# Free convection heat transfer inside square water-filled shallow enclosures

**DOI:** 10.1371/journal.pone.0204251

**Published:** 2018-10-31

**Authors:** Mohamed El-Sayed Ali, Abdullah O. Nuhait, Abdullah Alabdulkarem, Redhwan Almuzaiqer

**Affiliations:** Mechanical Engineering Department, College of Engineering, King Saud University, Riyadh, Saudi Arabia; Oak Ridge National Laboratory, UNITED STATES

## Abstract

Experimental study on free convection heat transfer was carried out inside shallow square enclosures filled with water. Two enclosures were used with size L × L × H (m^3^), where L and H are the side length and the inside thickness of the enclosure, respectively. Two different aspect ratios, κ = L/H, 7.143 and 12.0 were used. Constant heat flux boundary condition was applied at the bottom surface and stream of ambient air was applied at the top surface. Different values of constant heat fluxes were used as boundary conditions. For each aspect ratio of the enclosure, average Nusselt numbers were developed and correlated with the modified Rayleigh number. The results show that the heat transfer coefficient increases with the modified Rayleigh number with observed overlapping region between the two aspect ratios. A general overall correlation was developed using the aspect ratio as a parameter. The results also show that at the overlapping zone, the Nusselt number decreases as the aspect ratio increases for the fixed modified Rayleigh number.

## Introduction

Solar collectors, cooling and heating of buildings, windows with double pane and many other are applications of free convection in enclosures. Ostrach [1] reported a comprehensive review about the applications of free convection in enclosures. Natural convection in an inclined rectangular air layer for modified Rayleigh numbers, up to 2 × 10^6^, and different aspect ratios were reported experimentally by Inaba [2]. He presented different correlations for different Rayleigh numbers range and for several angles of inclination. Markatos and Pericleous [3] presented a computational method to solve the laminar or turbulent natural convection in a closed square cavity with differentially heated sidewalls. Square enclosures filled with water and heated from bottom and cooled from one vertical side was studied analytically and numerically by November and Nansteel [4]. Numerical simulations of free convection flow in an inclined square enclosure that has various orientations and different Rayleigh numbers were presented by Kuyper et al. [5]. They have developed free convection correlations for different inclinations and Rayleigh numbers. Rectangular enclosures cooled from one side and heated from bottom was numerically studied by Ganzarolli and Milanez [6]. Their study was done for isothermal or uniform heat flux boundary conditions and for different aspect ratios. They showed that, the Nusselt number correlation can be scaled as a function of the aspect ratio and the Rayleigh numbers to the power of 1/5 for the uniform heat flux boundary condition. Cioni et al. [7] presented an experimental study of high Rayleigh numbers turbulent regime in a cylindrical container of aspect ratio of one filled with water or mercury. Their results indicated a new transition to turbulent regime for Rayleigh number higher than 2 × 10^9^ with the increase of the Nusselt number. Numerical computation of transient three dimensional natural convection in a low Prandtl number fluids and in a shallow rectangular enclosure heated from bottom and cooled from top was reported by Nakano et al. [8]. Experimental natural convection Nusselt numbers were reported by Leong et al. [9] for an inclined cubical air-filled cavity that has one pair of isothermal opposing faces at different temperatures. Their results have shown Nusselt numbers versus Rayleigh numbers correlations for three different inclination angles. Aydin et al. [10] reported free convection in rectangular cavity cooled from the ceiling and heated from one side numerically. In their study, the effects of Rayleigh number and the aspect ratios on the flow and energy transport were investigated. Investigation on free convection in rectangular cavity cooled from top and heated from bottom with different specified thermal boundary conditions at the sidewalls was reported numerically by Corcione [11]. His results showed various correlations for different thermal configuration of the sidewalls. Square enclosures subject to discrete heater located on the lower wall and cooled from the lateral walls was studied numerically and experimentally by Calcagni et al. [12]. Deng [13] reported laminar natural convection in a square cavity with discrete heat source-sink pairs with different arrangements on the vertical sidewalls numerically. Recently, Ali at el. [14] presented experimental study on natural convection heat transfer inside a vertical circular enclosure filled with Al_2_O_3_ water based nanofluid. The effect of volume fraction of nanoparticles on the heat transfer was reported in details.

The earlier literature survey, which is mainly relied on numerical simulations, showed that Nusselt number in a shallow cavity depends on the Rayleigh number, aspect ratio, Prandtl number, and the sidewall boundary conditions. Even for a given specification of these parameters, the flow structure might not be unique [9]. To insure the flow structure and to obtain the overall correlations for the shallow cavity, experimental research is still needed on the structure of natural convection heat transfer in a shallow cavity with aspect ratio greater than unity and for high Rayleigh numbers. Therefore, the current experimental work was carried out to determine the behavior of free convection in shallow enclosures filled with water where the gap aspect ratio is greater than unity and adapting similar technique of [14]. Heat transfer coefficients were developed and a general correlation was developed for Nusselt numbers versus the modified Rayleigh numbers using aspect ratio as a parameter.

## Experimental setup

**Fig 1:**
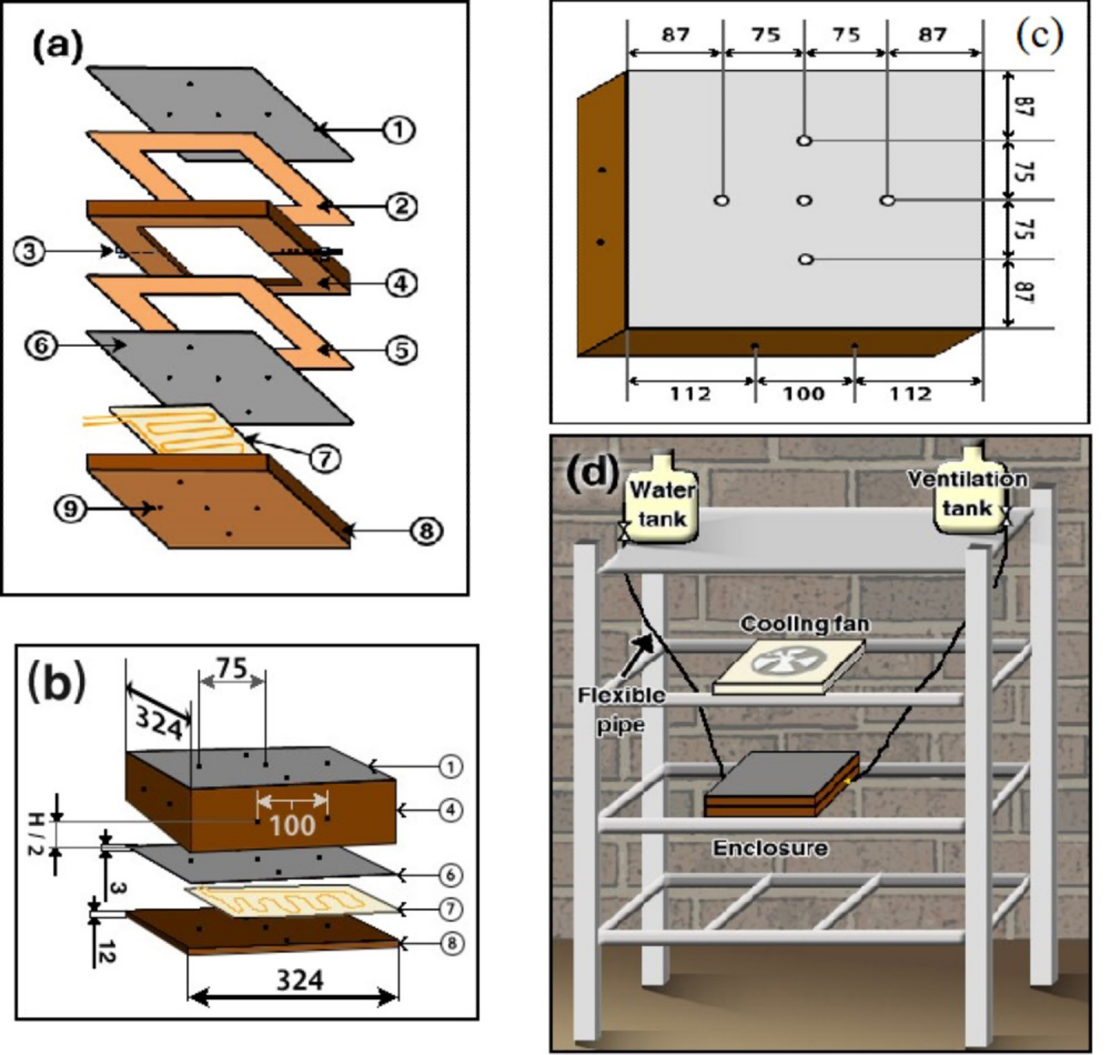
Experimental setup of the enclosure; (a and b) enclosure details, (1) top stainless cold plate, (2) gasket sheet, (3) two-way valve, (4) square enclosure side walls, (5) gasket sheet, (6) bottom stainless hot plate, (7) heater, (8) Bakelite insulator plate, (9) thermocouples, (c) Thermocouple locations (all dimensions are in mm), and (d) complete setup.

The experimental setup consists of two horizontal square enclosures (with vertical axis) of inside size 0.3 × 0.3 × H m^3^, where H is the inside thickness of the enclosure. Therefore, the enclosures have two different aspect ratios (κ = L/H = 7.143 and 12.0). It should be noted that the length and width of the enclosures were selected arbitrary such that they are suitable for laboratory measurements where any other dimensions could be used such that the cavity stay shallow. The circumference side wall of the enclosures are made of Bakelite insulating material (4) (k = 0.15 W/(m. K) [15]) as seen in details in Fig 1A and 1B. The enclosures were closed by two horizontal stainless steel square covers (1) and (6) of 0.003 m thickness each and having thermal conductivity of 16.3 W/(m. K) [15]. Two gasket sheets (2) and (5) are used to prevent any leakage. Flexible foil heater type (7) (0.3 × 0.3 m^2^ with a maximum thickness of 2.54 × 10^−4^ m (0.01in), and a maximum power of 1.55 × 10^4^ W/m^2^ (10 W/in^2^)) was. used to heat the lower stainless steel cover (6) as shown in Fig 1A and 1B. The heater was insulated using a Bakelite plate (8) of thickness 0.012 m. Five, type- K, self-adhesive surface temperature thermocouples were installed on the surface of the top and bottom stainless steel cover plates (1) and (6) respectively (shown as dots in Fig 1A–1C). Another five thermocouples (9) were installed on the lower surface of the bottom Bakelite (8). Eight more thermocouples were distributed around the sidewalls (4) of each enclosure, two at each side with eight inserted thermocouples to measure the heat loss by conduction through the sides. The thermocouple locations are shown in Fig 1C. The assembled enclosure was fixed in a frame as seen in Fig 1D. Fig 1D shows also two-way valves used for filling and ventilation. It should be mentioned that the top cold stainless steel plate (1) was subject to a stream of ambient air of 5 m/s as shown in Fig 1D. Voltage controller was used to control the input power to the heater and Wattmeter was used to measure the power consumption, which is distributed uniformly. The heat flux was calculated in a regular way following [14]. The voltage regulator was used to change the input power to the enclosure such that at least five input different powers are used. Those five input powers were chosen such that the maximum plate temperature does not exceed 90^o^ C to avoid boiling of water.

## Experimental procedure

**Fig 2:**
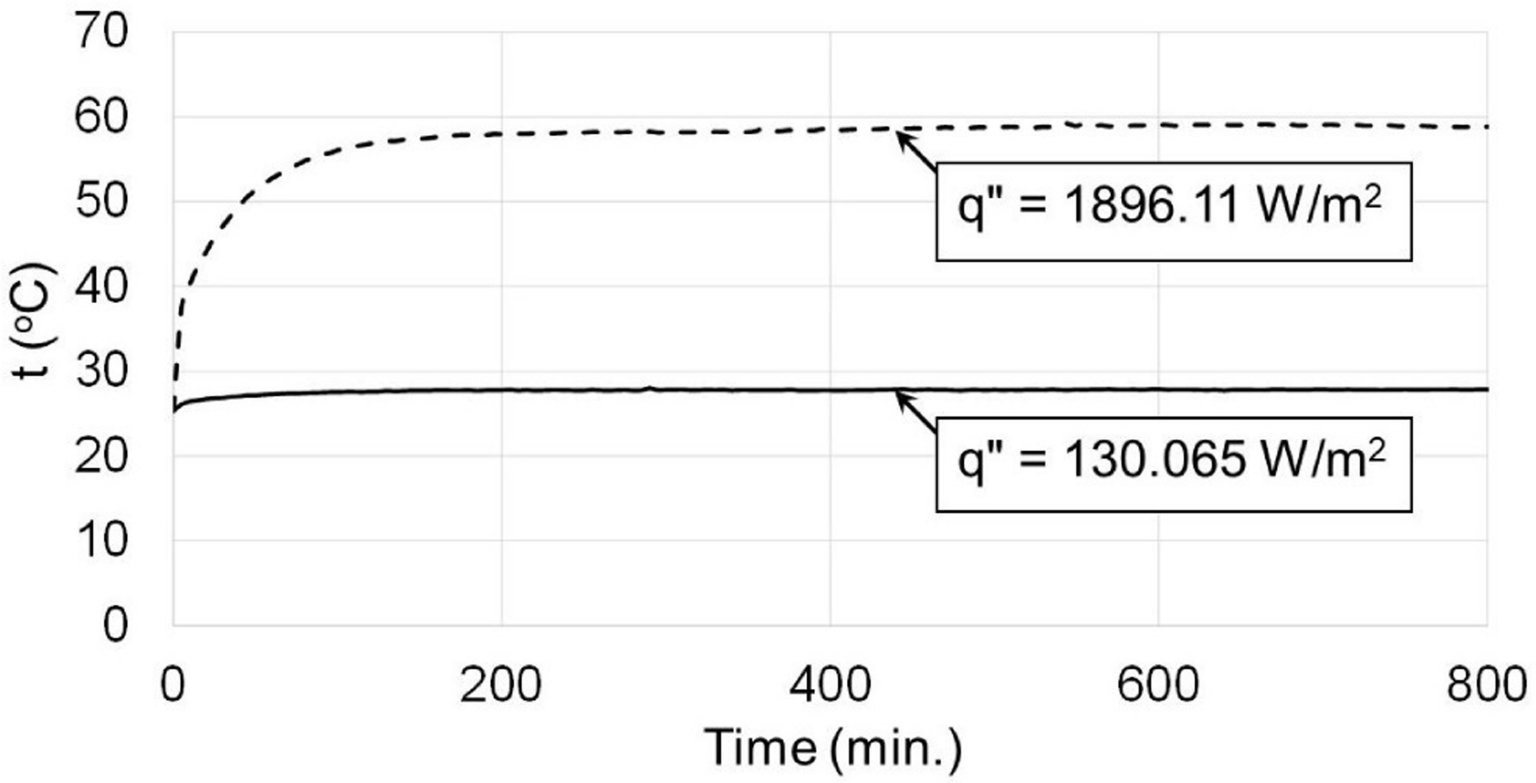
Temperature profiles at two different heat fluxes showing the condition of steady state, which reached after about 200 minutes.

The enclosure was filled with water using the two-way valves used for filling and ventilation as seen in Fig 1D. Voltage regulator is used to set the required heat flux which was measured by the wattmeter. The heater and the cooling fan are then turned on. The experiment was run until it reaches a steady state which usually takes about two to three hours based on the heat flux as seen in Fig 2. All thermocouples were connected to a computer via a data acquisition system where the measured temperatures were stored for heat transfer analyses.

## Experimental analyses

Most of the electrical power supplied to the foil heater dissipates heat through the stainless steel hot surface by conduction then to the water by convection. The minimum other part of the heat (losses) are transferred by conduction through the Bakelite sides and the lower Bakelite surface. Since the maximum temperature of the water does not exceed 90°C, the radiation heat transfer was neglected. Therefore, the total power and dissipated heat were calculated by:

$$Q_{total}=IV=Q_{Bkp}+Q_{Bks}+Q_{cv}$$ (1)

$$Q_{Bkp}=A_{Bkp}k_{Bk}\frac{{\bar{t}}_{h}-{\bar{t}}_{Bk}}{\delta_{Bkp}}$$ (2)

$$Q_{Bks}=A_{Bks}k_{Bk}\frac{{\bar{t}}_{si}-{\bar{t}}_{so}}{\delta_{Bks}}$$ (3)

$$Q_{cv}=IV-Q_{Bkp}-Q_{Bks}$$ (4)

It should be noted that Eqs (1–4) are valid for stationary staedy state conditions and one-dimensional heat transfer. All symboled defined in Eqs (1–4) are listed in the nomenclature. The water physical properties were evaluated at the average temperature ${\bar{t}}_{f}=\left({\bar{t}}_{h}+{\bar{t}}_{c}\right)/2$. Where ${\bar{t}}_{c}$, ${\bar{t}}_{h}$, ${\bar{t}}_{Bk}$ are the average surface temperatures of the top and bottom stainless steel surfaces and the bottom Bakelite surface respectively, ${\bar{t}}_{si}$ and ${\bar{t}}_{so}$ are the Bakelite side average inside and outside temperature respectively. The percentage of the lost heat through the bottom and sides of the Bakelite surfaces were calculated to be 4.4% and 1.8%, respectively at most.

## Average heat transfer coefficient h

At steady state condition, the heat transfer rate through the lower stainless plate, through the inside water and through the upper plate is equal. Therefore, the convection heat transfer Q_cv_ through the water can be calculated at each input power as:

$$Q_{cv}=\frac{{\bar{t}}_{h}-{\bar{t}}_{c}}{\sum R}$$ (5)

Where ∑R is the cavity thermal resistance:

$$\sum R=R_{ss}+R_{w}+R_{ss}$$ (6)

$$R_{ss}=\frac{\Delta x_{ss}}{A_{ss}k_{ss}},R_{w}=\frac{1}{Ah}$$ (7)

Where the subscripts ss and w stand for stainless steel and water respectively, and the area A_ss_ is the convection surface area A of the enclosure, *k*_*ss*_ = 16.3 W/(m. K) [15], and $\Delta x_{ss}$ is the thickness of the stainless steel surface. Plugging Eq (7) into (6) then into (5) we get:

$$Q_{cv}=\frac{{\bar{t}}_{h}-{\bar{t}}_{c}}{2\frac{\Delta x_{ss}}{A_{ss}k_{ss}}+\frac{1}{Ah}}$$ (8)

Then the average heat transfer coefficient can be obtained using the average hot and cold surface temperatures as:

$$h=\frac{1}{A\left[\frac{{\bar{t}}_{h}-{\bar{t}}_{c}}{Q_{cv}}-2\frac{\Delta x_{ss}}{A_{ss}k_{ss}}\right]}$$ (9)

The dimensionless average Nusselt and the modified Rayleigh numbers [16] are:

$$Nu=\frac{hH}{k}$$ (10)

$$Ra^{*}=\frac{g\beta Q_{cv}{\text{H}}^{4}}{k\upsilon \alpha A}$$ (11)

It should be noted that the cavity inside thickness H is used in expressions (10) and (11) as a characteristic length.

## Experimental uncertainty

The uncertainty of the experimental was calculated on the basis of the uncertainties in the primary measurements. It should be noted that, some of the experiments had to be repeated in order to check the calculated results and the general trends of the data. The error in determining the surface area and in measuring the temperature was ± 1.57 × 10^−5^ m^2^ and ± 0.2^o^ C, respectively. The manufacturer of the wattmeter provided the accuracy in measuring the voltage and the current as 0.5% of reading ±2 counts with a resolution of 0.1 V and 0.7% of the reading ± 5 counts + 1 mA with a resolution of 1 mA, respectively. At each temperature measurement, forty signals are obtained and transferred to the computer through the data acquisition system where the average was obtained. It should be noted that the uncertainty in the calculated result was calculated using the method recommended by Kline and McClintock [17] and Moffat [18] and a computer program was written to do that. Table 1 shows the maximum uncertainties of the calculated results.

**Table 1:** The uncertainties percentage of different parameters.

Parameters	Range (%)
Q_total_	3.41
$q_{BK}=\frac{Q_{BK}}{A_{BK}}$	6.77
$q^{''}$	5.07
*Nu*	7.48
h	6.32
Ra*	6.74

## Results and discussion

**Fig 3:**
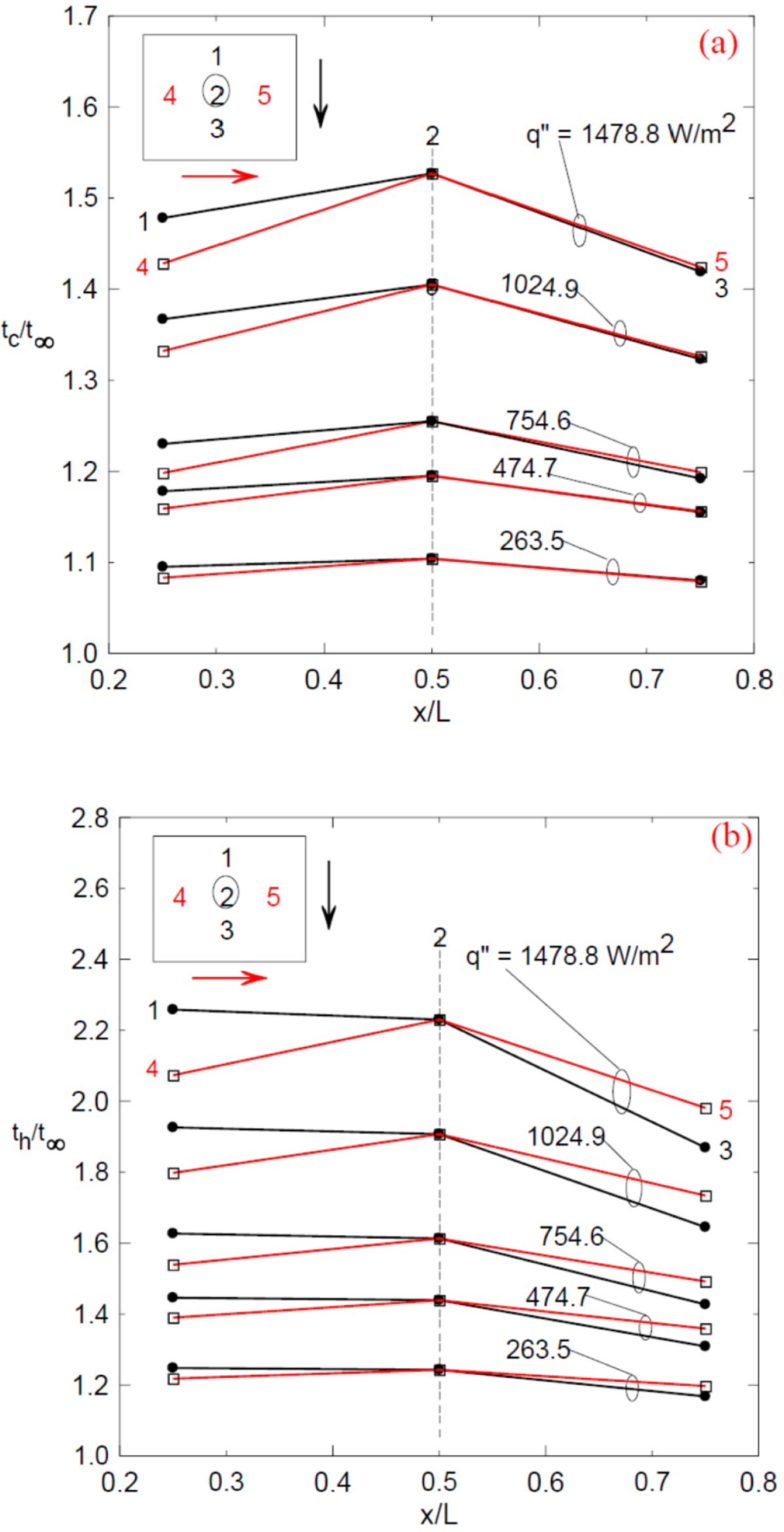
Temperature profiles along two different directions as specified by the arrows on the stainless steel surfaces; (a) the cold top surface and (b) the hot bottom surface.

The steady state condition at which the temperatures are measured are usually obtained after 400 minutes of running the experiment as seen in Fig 2 for two different heat fluxes. Samples of the temperature profiles across the distributed thermocouples normalized by the ambient temperature are shown in Fig 3A and 3B for enclosure having aspect ratio κ = 7.143 (H = 0.042 m) and for different values of heat flux. Fig 3A and 3B show the temperature profiles at different points of the cold and hot stainless steel surfaces, respectively following the arrows direction.

**Fig 4:**
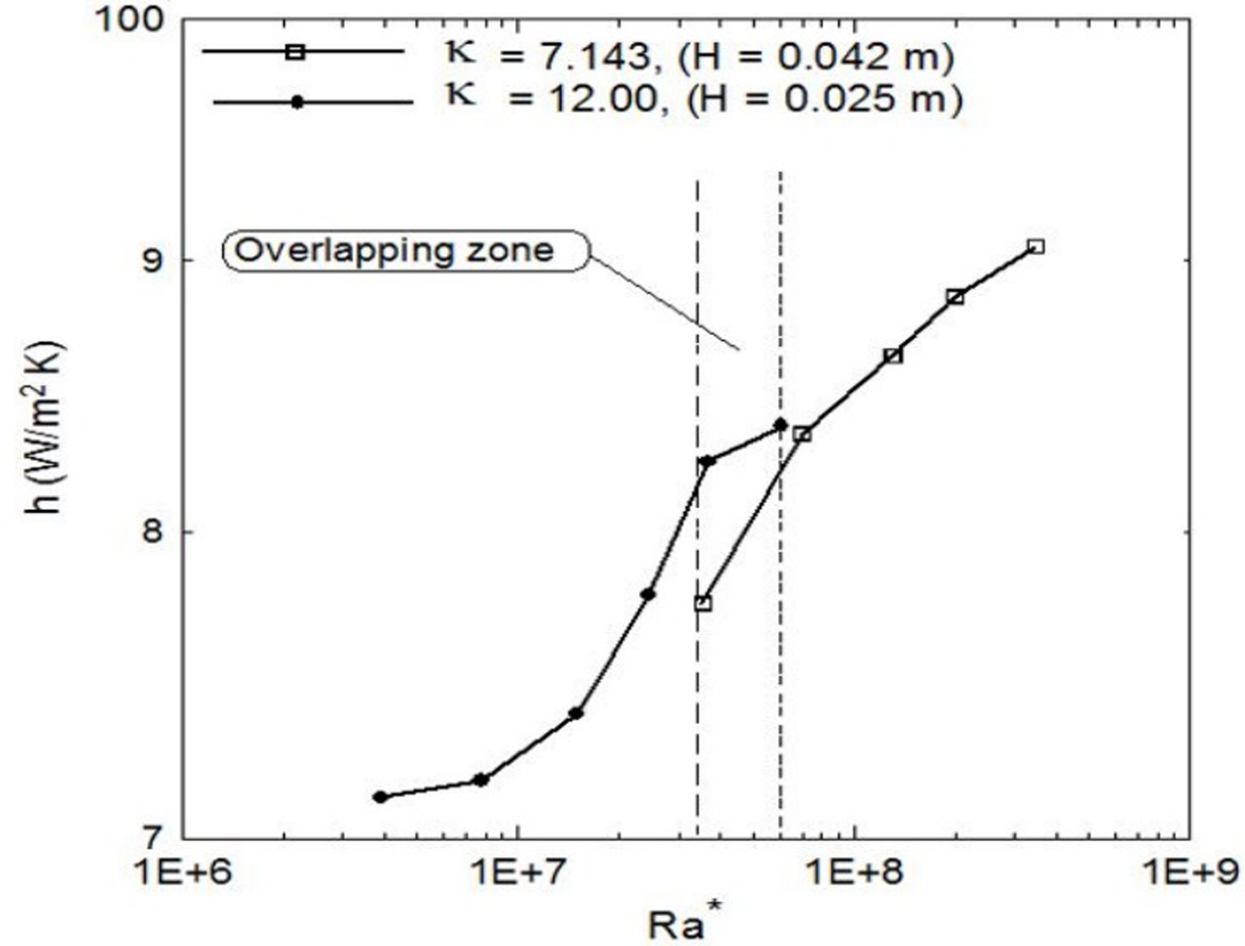
Average heat transfer coefficients profiles for two different aspect ratios versus the modified Rayleigh numbers showing the overlapping region.

**Fig 5:**
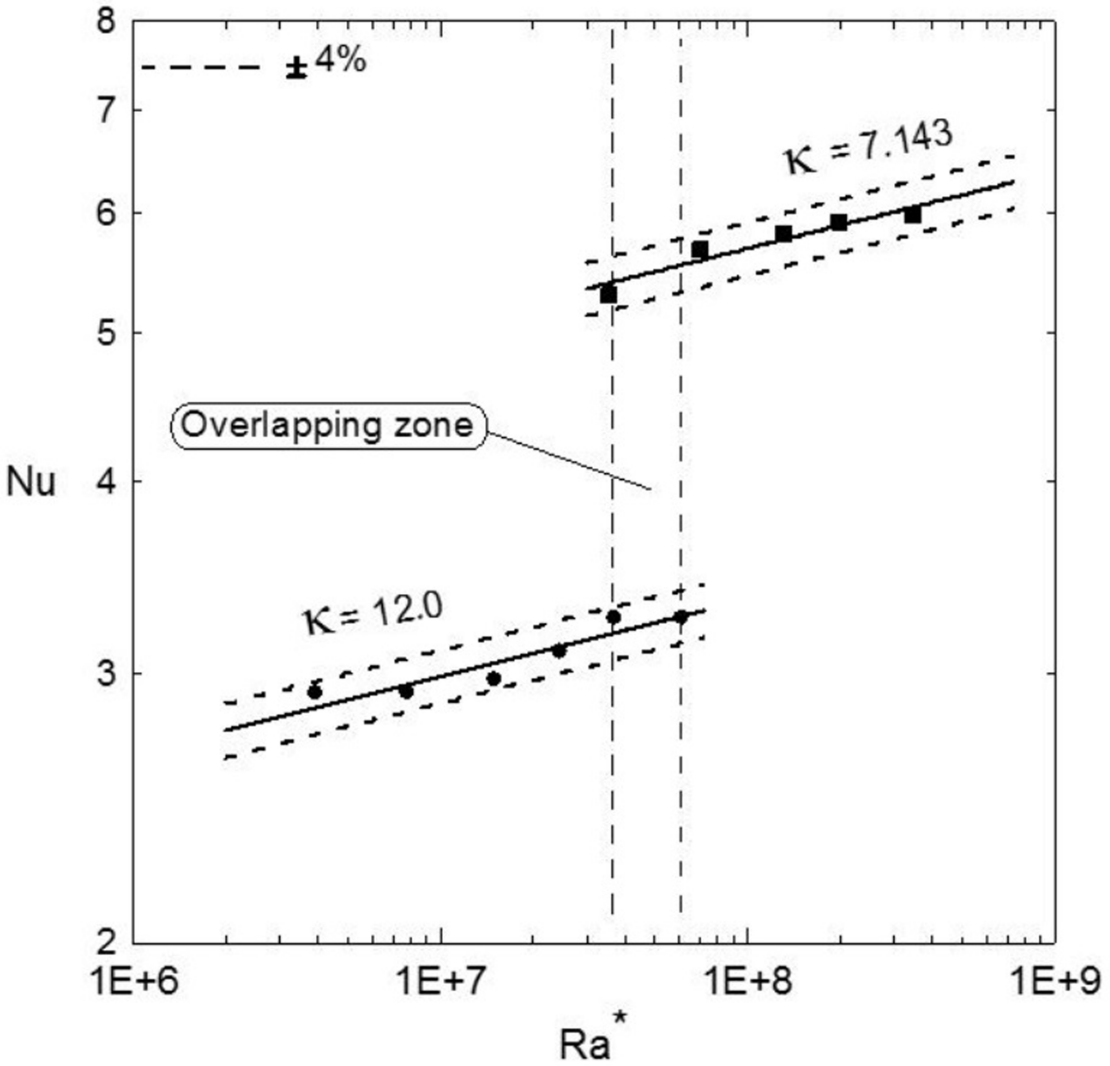
Average Nusselt numbers versus the modified Rayleigh numbers; solid lines showing the fitting through the data given by Eqs (12) and (13) or by Eq (14) using the aspect ratio as a parameter.

It can be seen that the temperature increases as the heat flux increases and the maximum temperature is almost at the middle of the plates at point 2. It should be mentioned that the other enclosure gives similar profiles. Fig 4 shows the heat transfer coefficient for the two enclosures versus the modified Rayleigh numbers on a logarithmic scale. It is also clear that as the aspect ratio decreases (larger gap thickness, H) the heat transfer coefficient increases as the Ra* increases; however, there is a region of overlapping zone occurs as shown by the vertical dashed lines where Ra* is the same for both enclosures. At this overlapping region, one could expect similar performance for the two enclosures where the heating effect overcomes the effect of increasing the size of the enclosure due to increasing its thickness H. The Nusselt numbers distributions against the modified Rayleigh numbers for two different aspect ratios are shown in Fig 5 for two different aspect ratios. It is clear that Nusselt numbers increase as the Ra* increases where the solid lines present the fitting correlation through the data and the dashed lines show that all the data lie between an error bandwidth of ± 4%. The following correlations are obtained for the aspect ratio κ = 12 and 7.143, respectively.

$$Nu=1.37{\left(Ra^{*}\right)}^{0.048},4\times 10^{6}<Ra^{*}<6\times 10^{7}$$ (12)

$$Nu=2.19{\left(Ra^{*}\right)}^{0.052},3.5\times 10^{7}<Ra^{*}<3.5\times 10^{8}$$ (13)

The coefficient of determination, R^2^, for Eqs (12) and (13) are 87% and 91%, respectively. The more general correlation including the aspect ratio as a parameter is obtained as:

$$Nu=16.676{\left(Ra^{*}\right)}^{0.0502}\kappa^{-1.018},4\times 10^{6}<Ra^{*}<3.5\times 10^{8}$$ (14)

By inspection, Eq (14) shows that the average heat transfer coefficient can be scaled as

$$h\propto {\left(H\right)}^{0}{}^{.218}$$ (15)

Eq (15) indicates that, as the thickness H of the enclosure increases the average heat transfer coefficient increases which confirms with the h profiles obtained in Fig 4. Furthermore, the overlapping zone between the vertical dashed lines for constant Ra* suggests that

$$\text{Nu}\propto {\left(\kappa \right)}^{-1.018}$$ (16)

**Fig 6:**
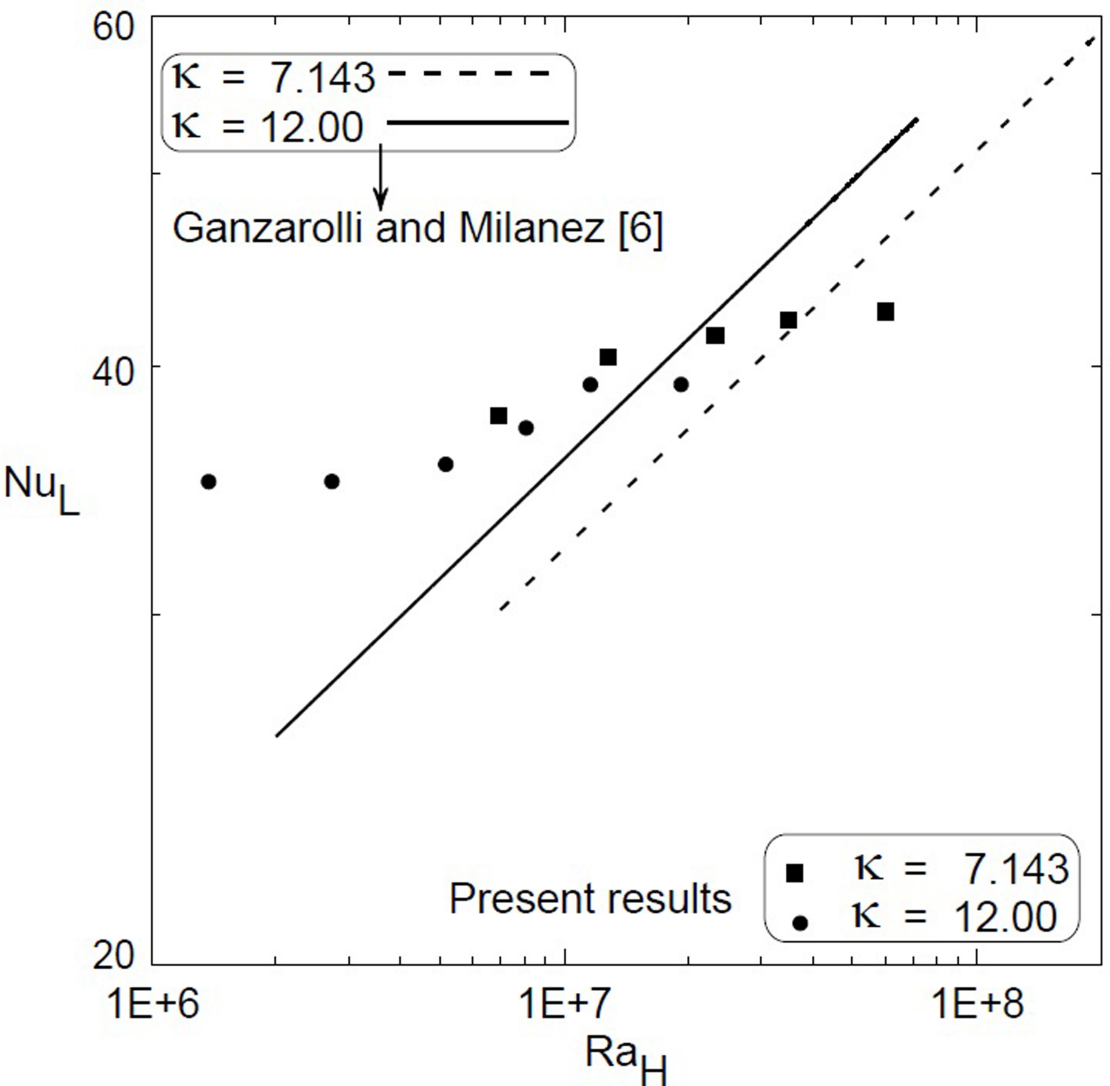
Comparison with [6] of Nusselt and Rayleigh numbers for two different aspect ratios; solid and dashed lines present the correlations given by [6] at the same aspect ratios and symbols present the current data.

Which confirms the finding that Nu decreases as the aspect ratio increases. It should be noted that Nusselt number in a shallow cavity depends on the Rayleigh number [2] and [9], aspect ratio of the cavity [2], [6] and [9], Prandtl number of the fluid [7– 9], and the sidewall boundary conditions [9]. Furthermore, even for a given specification of these parameters, the flow structure may still not be unique as described by [9]. Therefore, Fig 6 was constructed to compare the present results with those of [6] where the characteristic length used in Nusselt numbers is the side length L and that in Rayleigh numbers is the thickness of the cavity H to be consistent with [6]. In spite of the difference in the aspect ratio and the sidewalls boundary conditions, the current data show that most of the present results are in the range of the correlations obtained by [6] and presented in Fig 6 as solid and dashed lines.

## Conclusions

Free convection heat transfer inside vertical cavity filled with water, cooled from top and heated from bottom was experimentally investigated for two different gap aspect ratios, κ = 12 and 7.143. Experimental results show that the average heat transfer coefficient increases as the modified Rayleigh number increases for both aspect ratios. An overlapping region of the average heat transfer coefficient was developed at specified Ra* for the two aspect ratios. Correlations were developed for Nusselt numbers versus the modified Rayleigh numbers for each enclosure (Eqs (12) and (13)) and a more general correlation was developed using the aspect ratio as a parameter (Eq (14)). Comparison with the previous literature shows a qualitative agreement.

## Supporting information

S1 File Abbreviations. (DOCX)

S1 Table Data used for developing Fig 3A and 3B. (DOCX)

S2 Table Data used for developing Fig 4. (DOCX)

S3 Table Data used for developing Fig 5. (DOCX)

S4 Table Data used for developing Fig 6. (DOCX)
