# Supplementary material for: Free convection heat transfer inside square water-filled shallow enclosures
Source: PLoS One. 2018 Oct 31;13(10):e0204251. doi: 10.1371/journal.pone.0204251 (PMC6209140; doi:10.1371/journal.pone.0204251)
Supplement: S1 File — (DOCX) [file pone.0204251.s001.docx]

**Nomenclature**

*A Convection surface area, m^2^*

*A*_Bks_ Surface area of the Bakelite sidewalls, m^2^

A_Bkp_ Surface area of the Bakelite plates, m^2^

*h* Average heat transfer coefficient,$W m^{-2}K^{-1}$

*H* Gap inside thickness, (m)

*I* Electric current, A

*k* Thermal conductivity, $W m^{-1}K^{-1}$

*L* Square side length, (0.3 m)

*Nu* Nusselt number, h H / k or h L / k

*Q­­_total_* Electrical input power, W

$q''$ Convection heat flux, W/m^2^

*Q_Bkp_* Heat transfer rate lost by conduction through the bottom Bakelite plate, W

*Q_Bks_* Heat transfer rate lost by conduction through the Bakelite sidewalls, W

*Q_cv_* Convection heat transfer rate through the water, W

$Ra^{*}$ The modified Rayleigh number, $g \beta Q_{cv}H^{4}\upsilon^{-1}\alpha^{-1} k^{-1}A^{-1}$

Ra Rayleigh number, $g \beta\Delta TL^{3}\upsilon^{-1}\alpha^{-1}$

R Thermal resistance, K/W

*t* Temperature, ^o^C

*t*_si_ Inside surface temperature of the Bakelite sidewalls

*t*­_so_ Outside surface temperature of the Bakelite sidewalls

*V* Electric voltage, V

*Δ x* Stainless steel plate thickness, m

**Greek symbols**

α Thermal diffusivity, $m^{2}s^{-1}$

β Coefficient for thermal expansion, $K^{-1}$

δ Bakelite thickness

$\upsilon$ Kinematics viscosity,$m^{2}s^{-1}$

κ Aspect ratio, (L/H)

**Subscripts**

Bk Bakelite

c Cold

f Fluid

h Hot

H Characteristic length

L Characteristic length

**Superscripts**

- Averaged quantity
