# Supplementary material for: Free convection heat transfer inside square water-filled shallow enclosures
Source: PLoS One. 2018 Oct 31;13(10):e0204251. doi: 10.1371/journal.pone.0204251 (PMC6209140; doi:10.1371/journal.pone.0204251)
Supplement: S1 Table — (DOCX) [file pone.0204251.s002.docx]

Data for Fig. 3(a, b)

| $q^{"}$= 1478.8 W/m^2^, black, following 1 2 3 arrow direction. | | | $q^{"}$= 1478.8 W/m^2^, red, following 4 2 5 arrow direction. | | |
| --- | --- | --- | --- | --- | --- |
| Distance | Cold surface Fig. 3(a) | Hot surface Fig. 3(b) | Distance | Cold surface Fig. 3(a) | Hot surface Fig. 3(b) |
| X/D | t_c_/t_∞_ | T_h_/t_∞_ | X/D | t_c_/t_∞_ | T_h_/t_∞_ |
| 0.25 | 1.478 | 2.258 | 0.25 | 1.428 | 2.073 |
| 0.50 | 1.527 | 2.230 | 0.50 | 1.428 | 2.230 |
| 0.75 | 1.419 | 1.869 | 0.75 | 1.424 | 1.981 |
| $q^{"}$= 1024.9 W/m^2^, black, following 1 2 3 arrow direction. | | | $q^{"}$= 1024.9 W/m^2^, red, following 4 2 5 arrow direction. | | |
| X/D | t_c_/t_∞_ | T_h_/t_∞_ | X/D | t_c_/t_∞_ | T_h_/t_∞_ |
| 0.25 | 1.367 | 1.926 | 0.25 | 1.332 | 1.798 |
| 0.50 | 1.405 | 1.907 | 0.50 | 1.405 | 1.907 |
| 0.75 | 1.323 | 1.645 | 0.75 | 1.326 | 1.734 |
| $q^{"}$= 754.6 W/m^2^, black, following 1 2 3 arrow direction. | | | $q^{"}$= 754.6 W/m^2^, red, following 4 2 5 arrow direction. | | |
| X/D | t_c_/t_∞_ | T_h_/t_∞_ | X/D | t_c_/t_∞_ | T_h_/t_∞_ |
| 0.25 | 1.230 | 1.627 | 0.25 | 1.198 | 1.539 |
| 0.50 | 1.255 | 1.613 | 0.50 | 1.255 | 1.613 |
| 0.75 | 1.192 | 1.427 | 0.75 | 1.199 | 1.492 |
| $q^{"}$= 474.7 W/m^2^, black, following 1 2 3 arrow direction. | | | $q^{"}$= 474.7 W/m^2^, red, following 4 2 5 arrow direction. | | |
| X/D | t_c_/t_∞_ | T_h_/t_∞_ | X/D | t_c_/t_∞_ | T_h_/t_∞_ |
| 0.25 | 1.178 | 1.446 | 0.25 | 1.159 | 1.390 |
| 0.50 | 1.195 | 1.439 | 0.50 | 1.195 | 1.439 |
| 0.75 | 1.155 | 1.309 | 0.75 | 1.156 | 1.359 |
| $q^{"}$= 263.5 W/m^2^, black, following 1 2 3 arrow direction. | | | $q^{"}$= 263.5 W/m^2^, red, following 4 2 5 arrow direction. | | |
| X/D | t_c_/t_∞_ | T_h_/t_∞_ | X/D | t_c_/t_∞_ | T_h_/t_∞_ |
| 0.25 | 1.095 | 1.248 | 0.25 | 1.083 | 1.218 |
| 0.50 | 1.104 | 1.243 | 0.50 | 1.104 | 1.243 |
| 0.75 | 1.080 | 1.168 | 0.75 | 1.079 | 1.197 |
