# Supplementary material for: Free convection heat transfer inside square water-filled shallow enclosures
Source: PLoS One. 2018 Oct 31;13(10):e0204251. doi: 10.1371/journal.pone.0204251 (PMC6209140; doi:10.1371/journal.pone.0204251)
Supplement: S2 Table — (DOCX) [file pone.0204251.s003.docx]

Data for Fig. 4.

| For κ = 7.143 (H = 0.042 m) | |
| --- | --- |
| Ra* | h(W/m^2^K) |
| 35477212.760 | 77.547 |
| 70074119.433 | 83.498 |
| 130340762.423 | 86.360 |
| 199471881.880 | 88.624 |
| 345717810.784 | 90.561 |
| For κ = 12.00 (H = 0.025 m) | |
| Ra* | h(W/m^2^K) |
| 3916613.415 | 71.243 |
| 7797637.704 | 71.781 |
| 15005242.688 | 73.901 |
| 24472033.777 | 77.807 |
| 36801541.519 | 82.473 |
| 61062761.349 | 83.738 |
