# Supplementary material for: Free convection heat transfer inside square water-filled shallow enclosures
Source: PLoS One. 2018 Oct 31;13(10):e0204251. doi: 10.1371/journal.pone.0204251 (PMC6209140; doi:10.1371/journal.pone.0204251)
Supplement: S3 Table — (DOCX) [file pone.0204251.s004.docx]

Fitting data for Fig. 5 for κ= 12 7.143, values of Nu12p4%, Nu12n4%, Nu7.14p4% and Nu7.14n4% stand for NU at κ= 12 plus increase of 4%, NU at κ= 12 mines decrease of 4%, NU at κ= 7.14 plus increase of 4%, and NU at κ= 7.14 mines decrease of 4%.

Ra^*^(κ=12) Nu(κ=12) Ra^*^(κ=7.143) Nu(κ=7.143) Nu12p4% Nu12n4% Nu7.14p4% Nu7.14n4%

2000000.000 2.75500 30000000.000 5.35 2.87 2.64 5.56 5.14

2007000.000 2.75508 30070000.000 5.35 2.87 2.64 5.56 5.14

2014000.000 2.75557 30140000.000 5.35 2.87 2.65 5.56 5.14

2021000.000 2.75604 30210000.000 5.35 2.87 2.65 5.57 5.14

2028000.000 2.75652 30280000.000 5.35 2.87 2.65 5.57 5.14

2035000.000 2.75700 30350000.000 5.35 2.87 2.65 5.57 5.14

2042000.000 2.75747 30420000.000 5.35 2.87 2.65 5.57 5.14

2049000.000 2.75795 30490000.000 5.35 2.87 2.65 5.57 5.14

2056000.000 2.75842 30560000.000 5.35 2.87 2.65 5.57 5.14

2063000.000 2.75889 30630000.000 5.35 2.87 2.65 5.57 5.14

2070000.000 2.75936 30700000.000 5.36 2.87 2.65 5.57 5.14

2077000.000 2.75983 30770000.000 5.36 2.87 2.65 5.57 5.14

2084000.000 2.76029 30840000.000 5.36 2.87 2.65 5.57 5.14

2091000.000 2.76076 30910000.000 5.36 2.87 2.65 5.57 5.14

2098000.000 2.76122 30980000.000 5.36 2.87 2.65 5.57 5.14

2105000.000 2.76168 31050000.000 5.36 2.87 2.65 5.57 5.14

2112000.000 2.76214 31120000.000 5.36 2.87 2.65 5.57 5.14

2119000.000 2.76260 31190000.000 5.36 2.87 2.65 5.57 5.15

2126000.000 2.76306 31260000.000 5.36 2.87 2.65 5.57 5.15

2133000.000 2.76351 31330000.000 5.36 2.87 2.65 5.58 5.15

2140000.000 2.76397 31400000.000 5.36 2.87 2.65 5.58 5.15

2147000.000 2.76442 31470000.000 5.36 2.87 2.65 5.58 5.15

2154000.000 2.76487 31540000.000 5.36 2.88 2.65 5.58 5.15

2161000.000 2.76532 31610000.000 5.36 2.88 2.65 5.58 5.15

2168000.000 2.76577 31680000.000 5.36 2.88 2.66 5.58 5.15

2175000.000 2.76622 31750000.000 5.36 2.88 2.66 5.58 5.15

2182000.000 2.76666 31820000.000 5.37 2.88 2.66 5.58 5.15

2189000.000 2.76711 31890000.000 5.37 2.88 2.66 5.58 5.15

2196000.000 2.76755 31960000.000 5.37 2.88 2.66 5.58 5.15

2203000.000 2.76799 32030000.000 5.37 2.88 2.66 5.58 5.15

2210000.000 2.76843 32100000.000 5.37 2.88 2.66 5.58 5.15

2217000.000 2.76887 32170000.000 5.37 2.88 2.66 5.58 5.15

2224000.000 2.76931 32240000.000 5.37 2.88 2.66 5.58 5.15

2231000.000 2.76975 32310000.000 5.37 2.88 2.66 5.58 5.15

2238000.000 2.77018 32380000.000 5.37 2.88 2.66 5.58 5.16

2245000.000 2.77061 32450000.000 5.37 2.88 2.66 5.59 5.16

2252000.000 2.77105 32520000.000 5.37 2.88 2.66 5.59 5.16

2259000.000 2.77148 32590000.000 5.37 2.88 2.66 5.59 5.16

2266000.000 2.77191 32660000.000 5.37 2.88 2.66 5.59 5.16

2273000.000 2.77234 32730000.000 5.37 2.88 2.66 5.59 5.16

2280000.000 2.77276 32800000.000 5.37 2.88 2.66 5.59 5.16

2287000.000 2.77319 32870000.000 5.37 2.88 2.66 5.59 5.16

2294000.000 2.77362 32940000.000 5.37 2.88 2.66 5.59 5.16

2301000.000 2.77404 33010000.000 5.38 2.89 2.66 5.59 5.16

2308000.000 2.77446 33080000.000 5.38 2.89 2.66 5.59 5.16

2315000.000 2.77488 33150000.000 5.38 2.89 2.66 5.59 5.16

2322000.000 2.77530 33220000.000 5.38 2.89 2.66 5.59 5.16

2329000.000 2.77572 33290000.000 5.38 2.89 2.66 5.59 5.16

2336000.000 2.77614 33360000.000 5.38 2.89 2.67 5.59 5.16

2343000.000 2.77656 33430000.000 5.38 2.89 2.67 5.59 5.16

2350000.000 2.77697 33500000.000 5.38 2.89 2.67 5.59 5.16

2357000.000 2.77739 33570000.000 5.38 2.89 2.67 5.59 5.16

2364000.000 2.77780 33640000.000 5.38 2.89 2.67 5.60 5.16

2371000.000 2.77821 33710000.000 5.38 2.89 2.67 5.60 5.17

2378000.000 2.77862 33780000.000 5.38 2.89 2.67 5.60 5.17

2385000.000 2.77903 33850000.000 5.38 2.89 2.67 5.60 5.17

2392000.000 2.77944 33920000.000 5.38 2.89 2.67 5.60 5.17

2399000.000 2.77985 33990000.000 5.38 2.89 2.67 5.60 5.17

2406000.000 2.78026 34060000.000 5.38 2.89 2.67 5.60 5.17

2413000.000 2.78066 34130000.000 5.38 2.89 2.67 5.60 5.17

2420000.000 2.78107 34200000.000 5.38 2.89 2.67 5.60 5.17

2427000.000 2.78147 34270000.000 5.39 2.89 2.67 5.60 5.17

2434000.000 2.78187 34340000.000 5.39 2.89 2.67 5.60 5.17

2441000.000 2.78227 34410000.000 5.39 2.89 2.67 5.60 5.17

2448000.000 2.78267 34480000.000 5.39 2.89 2.67 5.60 5.17

2455000.000 2.78307 34550000.000 5.39 2.89 2.67 5.60 5.17

2462000.000 2.78347 34620000.000 5.39 2.89 2.67 5.60 5.17

2469000.000 2.78386 34690000.000 5.39 2.90 2.67 5.60 5.17

2476000.000 2.78426 34760000.000 5.39 2.90 2.67 5.60 5.17

2483000.000 2.78465 34830000.000 5.39 2.90 2.67 5.61 5.17

2490000.000 2.78505 34900000.000 5.39 2.90 2.67 5.61 5.17

2497000.000 2.78544 34970000.000 5.39 2.90 2.67 5.61 5.18

2504000.000 2.78583 35040000.000 5.39 2.90 2.67 5.61 5.18

2511000.000 2.78622 35110000.000 5.39 2.90 2.67 5.61 5.18

2518000.000 2.78661 35180000.000 5.39 2.90 2.68 5.61 5.18

2525000.000 2.78700 35250000.000 5.39 2.90 2.68 5.61 5.18

2532000.000 2.78738 35320000.000 5.39 2.90 2.68 5.61 5.18

2539000.000 2.78777 35390000.000 5.39 2.90 2.68 5.61 5.18

2546000.000 2.78815 35460000.000 5.39 2.90 2.68 5.61 5.18

2553000.000 2.78854 35530000.000 5.39 2.90 2.68 5.61 5.18

2560000.000 2.78892 35600000.000 5.40 2.90 2.68 5.61 5.18

2567000.000 2.78930 35670000.000 5.40 2.90 2.68 5.61 5.18

2574000.000 2.78969 35740000.000 5.40 2.90 2.68 5.61 5.18

2581000.000 2.79007 35810000.000 5.40 2.90 2.68 5.61 5.18

2588000.000 2.79044 35880000.000 5.40 2.90 2.68 5.61 5.18

2595000.000 2.79082 35950000.000 5.40 2.90 2.68 5.61 5.18

2602000.000 2.79120 36020000.000 5.40 2.90 2.68 5.61 5.18

2609000.000 2.79158 36090000.000 5.40 2.90 2.68 5.62 5.18

2616000.000 2.79195 36160000.000 5.40 2.90 2.68 5.62 5.18

2623000.000 2.79233 36230000.000 5.40 2.90 2.68 5.62 5.18

2630000.000 2.79270 36300000.000 5.40 2.90 2.68 5.62 5.18

2637000.000 2.79307 36370000.000 5.40 2.90 2.68 5.62 5.19

2644000.000 2.79344 36440000.000 5.40 2.91 2.68 5.62 5.19

2651000.000 2.79381 36510000.000 5.40 2.91 2.68 5.62 5.19

2658000.000 2.79418 36580000.000 5.40 2.91 2.68 5.62 5.19

2665000.000 2.79455 36650000.000 5.40 2.91 2.68 5.62 5.19

2672000.000 2.79492 36720000.000 5.40 2.91 2.68 5.62 5.19

2679000.000 2.79529 36790000.000 5.40 2.91 2.68 5.62 5.19

2686000.000 2.79565 36860000.000 5.40 2.91 2.68 5.62 5.19

2693000.000 2.79602 36930000.000 5.41 2.91 2.68 5.62 5.19

2700000.000 2.79638 37000000.000 5.41 2.91 2.68 5.62 5.19

2707000.000 2.79674 37070000.000 5.41 2.91 2.68 5.62 5.19

2714000.000 2.79711 37140000.000 5.41 2.91 2.69 5.62 5.19

2721000.000 2.79747 37210000.000 5.41 2.91 2.69 5.62 5.19

2728000.000 2.79783 37280000.000 5.41 2.91 2.69 5.62 5.19

2735000.000 2.79819 37350000.000 5.41 2.91 2.69 5.62 5.19

2742000.000 2.79855 37420000.000 5.41 2.91 2.69 5.63 5.19

2749000.000 2.79890 37490000.000 5.41 2.91 2.69 5.63 5.19

2756000.000 2.79926 37560000.000 5.41 2.91 2.69 5.63 5.19

2763000.000 2.79962 37630000.000 5.41 2.91 2.69 5.63 5.19

2770000.000 2.79997 37700000.000 5.41 2.91 2.69 5.63 5.19

2777000.000 2.80033 37770000.000 5.41 2.91 2.69 5.63 5.20

2784000.000 2.80068 37840000.000 5.41 2.91 2.69 5.63 5.20

2791000.000 2.80103 37910000.000 5.41 2.91 2.69 5.63 5.20

2798000.000 2.80139 37980000.000 5.41 2.91 2.69 5.63 5.20

2805000.000 2.80174 38050000.000 5.41 2.91 2.69 5.63 5.20

2812000.000 2.80209 38120000.000 5.41 2.91 2.69 5.63 5.20

2819000.000 2.80244 38190000.000 5.41 2.91 2.69 5.63 5.20

2826000.000 2.80279 38260000.000 5.41 2.91 2.69 5.63 5.20

2833000.000 2.80313 38330000.000 5.42 2.92 2.69 5.63 5.20

2840000.000 2.80348 38400000.000 5.42 2.92 2.69 5.63 5.20

2847000.000 2.80383 38470000.000 5.42 2.92 2.69 5.63 5.20

2854000.000 2.80417 38540000.000 5.42 2.92 2.69 5.63 5.20

2861000.000 2.80452 38610000.000 5.42 2.92 2.69 5.63 5.20

2868000.000 2.80486 38680000.000 5.42 2.92 2.69 5.63 5.20

2875000.000 2.80520 38750000.000 5.42 2.92 2.69 5.64 5.20

2882000.000 2.80555 38820000.000 5.42 2.92 2.69 5.64 5.20

2889000.000 2.80589 38890000.000 5.42 2.92 2.69 5.64 5.20

2896000.000 2.80623 38960000.000 5.42 2.92 2.69 5.64 5.20

2903000.000 2.80657 39030000.000 5.42 2.92 2.69 5.64 5.20

2910000.000 2.80691 39100000.000 5.42 2.92 2.69 5.64 5.20

2917000.000 2.80724 39170000.000 5.42 2.92 2.69 5.64 5.20

2924000.000 2.80758 39240000.000 5.42 2.92 2.70 5.64 5.20

2931000.000 2.80792 39310000.000 5.42 2.92 2.70 5.64 5.21

2938000.000 2.80825 39380000.000 5.42 2.92 2.70 5.64 5.21

2945000.000 2.80859 39450000.000 5.42 2.92 2.70 5.64 5.21

2952000.000 2.80892 39520000.000 5.42 2.92 2.70 5.64 5.21

2959000.000 2.80926 39590000.000 5.42 2.92 2.70 5.64 5.21

2966000.000 2.80959 39660000.000 5.42 2.92 2.70 5.64 5.21

2973000.000 2.80992 39730000.000 5.43 2.92 2.70 5.64 5.21

2980000.000 2.81026 39800000.000 5.43 2.92 2.70 5.64 5.21

2987000.000 2.81059 39870000.000 5.43 2.92 2.70 5.64 5.21

2994000.000 2.81092 39940000.000 5.43 2.92 2.70 5.64 5.21

3001000.000 2.81125 40010000.000 5.43 2.92 2.70 5.64 5.21

3008000.000 2.81157 40080000.000 5.43 2.92 2.70 5.64 5.21

3015000.000 2.81190 40150000.000 5.43 2.92 2.70 5.65 5.21

3022000.000 2.81223 40220000.000 5.43 2.92 2.70 5.65 5.21

3029000.000 2.81255 40290000.000 5.43 2.93 2.70 5.65 5.21

3036000.000 2.81288 40360000.000 5.43 2.93 2.70 5.65 5.21

3043000.000 2.81321 40430000.000 5.43 2.93 2.70 5.65 5.21

3050000.000 2.81353 40500000.000 5.43 2.93 2.70 5.65 5.21

3057000.000 2.81385 40570000.000 5.43 2.93 2.70 5.65 5.21

3064000.000 2.81418 40640000.000 5.43 2.93 2.70 5.65 5.21

3071000.000 2.81450 40710000.000 5.43 2.93 2.70 5.65 5.21

3078000.000 2.81482 40780000.000 5.43 2.93 2.70 5.65 5.22

3085000.000 2.81514 40850000.000 5.43 2.93 2.70 5.65 5.22

3092000.000 2.81546 40920000.000 5.43 2.93 2.70 5.65 5.22

3099000.000 2.81578 40990000.000 5.43 2.93 2.70 5.65 5.22

3106000.000 2.81610 41060000.000 5.43 2.93 2.70 5.65 5.22

3113000.000 2.81642 41130000.000 5.43 2.93 2.70 5.65 5.22

3120000.000 2.81673 41200000.000 5.44 2.93 2.70 5.65 5.22

3127000.000 2.81705 41270000.000 5.44 2.93 2.70 5.65 5.22

3134000.000 2.81737 41340000.000 5.44 2.93 2.70 5.65 5.22

3141000.000 2.81768 41410000.000 5.44 2.93 2.70 5.65 5.22

3148000.000 2.81800 41480000.000 5.44 2.93 2.71 5.65 5.22

3155000.000 2.81831 41550000.000 5.44 2.93 2.71 5.65 5.22

3162000.000 2.81862 41620000.000 5.44 2.93 2.71 5.66 5.22

3169000.000 2.81894 41690000.000 5.44 2.93 2.71 5.66 5.22

3176000.000 2.81925 41760000.000 5.44 2.93 2.71 5.66 5.22

3183000.000 2.81956 41830000.000 5.44 2.93 2.71 5.66 5.22

3190000.000 2.81987 41900000.000 5.44 2.93 2.71 5.66 5.22

3197000.000 2.82018 41970000.000 5.44 2.93 2.71 5.66 5.22

3204000.000 2.82049 42040000.000 5.44 2.93 2.71 5.66 5.22

3211000.000 2.82080 42110000.000 5.44 2.93 2.71 5.66 5.22

3218000.000 2.82111 42180000.000 5.44 2.93 2.71 5.66 5.22

3225000.000 2.82141 42250000.000 5.44 2.93 2.71 5.66 5.22

3232000.000 2.82172 42320000.000 5.44 2.93 2.71 5.66 5.22

3239000.000 2.82203 42390000.000 5.44 2.93 2.71 5.66 5.23

3246000.000 2.82233 42460000.000 5.44 2.94 2.71 5.66 5.23

3253000.000 2.82264 42530000.000 5.44 2.94 2.71 5.66 5.23

3260000.000 2.82294 42600000.000 5.44 2.94 2.71 5.66 5.23

3267000.000 2.82325 42670000.000 5.44 2.94 2.71 5.66 5.23

3274000.000 2.82355 42740000.000 5.45 2.94 2.71 5.66 5.23

3281000.000 2.82385 42810000.000 5.45 2.94 2.71 5.66 5.23

3288000.000 2.82415 42880000.000 5.45 2.94 2.71 5.66 5.23

3295000.000 2.82446 42950000.000 5.45 2.94 2.71 5.66 5.23

3302000.000 2.82476 43020000.000 5.45 2.94 2.71 5.66 5.23

3309000.000 2.82506 43090000.000 5.45 2.94 2.71 5.67 5.23

3316000.000 2.82536 43160000.000 5.45 2.94 2.71 5.67 5.23

3323000.000 2.82565 43230000.000 5.45 2.94 2.71 5.67 5.23

3330000.000 2.82595 43300000.000 5.45 2.94 2.71 5.67 5.23

3337000.000 2.82625 43370000.000 5.45 2.94 2.71 5.67 5.23

3344000.000 2.82655 43440000.000 5.45 2.94 2.71 5.67 5.23

3351000.000 2.82684 43510000.000 5.45 2.94 2.71 5.67 5.23

3358000.000 2.82714 43580000.000 5.45 2.94 2.71 5.67 5.23

3365000.000 2.82743 43650000.000 5.45 2.94 2.71 5.67 5.23

3372000.000 2.82773 43720000.000 5.45 2.94 2.71 5.67 5.23

3379000.000 2.82802 43790000.000 5.45 2.94 2.71 5.67 5.23

3386000.000 2.82832 43860000.000 5.45 2.94 2.72 5.67 5.23

3393000.000 2.82861 43930000.000 5.45 2.94 2.72 5.67 5.23

3400000.000 2.82890 44000000.000 5.45 2.94 2.72 5.67 5.23

3407000.000 2.82919 44070000.000 5.45 2.94 2.72 5.67 5.24

3414000.000 2.82949 44140000.000 5.45 2.94 2.72 5.67 5.24

3421000.000 2.82978 44210000.000 5.45 2.94 2.72 5.67 5.24

3428000.000 2.83007 44280000.000 5.45 2.94 2.72 5.67 5.24

3435000.000 2.83036 44350000.000 5.46 2.94 2.72 5.67 5.24

3442000.000 2.83065 44420000.000 5.46 2.94 2.72 5.67 5.24

3449000.000 2.83093 44490000.000 5.46 2.94 2.72 5.67 5.24

3456000.000 2.83122 44560000.000 5.46 2.94 2.72 5.67 5.24

3463000.000 2.83151 44630000.000 5.46 2.94 2.72 5.68 5.24

3470000.000 2.83180 44700000.000 5.46 2.95 2.72 5.68 5.24

3477000.000 2.83208 44770000.000 5.46 2.95 2.72 5.68 5.24

3484000.000 2.83237 44840000.000 5.46 2.95 2.72 5.68 5.24

3491000.000 2.83265 44910000.000 5.46 2.95 2.72 5.68 5.24

3498000.000 2.83294 44980000.000 5.46 2.95 2.72 5.68 5.24

3505000.000 2.83322 45050000.000 5.46 2.95 2.72 5.68 5.24

3512000.000 2.83351 45120000.000 5.46 2.95 2.72 5.68 5.24

3519000.000 2.83379 45190000.000 5.46 2.95 2.72 5.68 5.24

3526000.000 2.83407 45260000.000 5.46 2.95 2.72 5.68 5.24

3533000.000 2.83435 45330000.000 5.46 2.95 2.72 5.68 5.24

3540000.000 2.83463 45400000.000 5.46 2.95 2.72 5.68 5.24

3547000.000 2.83492 45470000.000 5.46 2.95 2.72 5.68 5.24

3554000.000 2.83520 45540000.000 5.46 2.95 2.72 5.68 5.24

3561000.000 2.83548 45610000.000 5.46 2.95 2.72 5.68 5.24

3568000.000 2.83575 45680000.000 5.46 2.95 2.72 5.68 5.24

3575000.000 2.83603 45750000.000 5.46 2.95 2.72 5.68 5.25

3582000.000 2.83631 45820000.000 5.46 2.95 2.72 5.68 5.25

3589000.000 2.83659 45890000.000 5.46 2.95 2.72 5.68 5.25

3596000.000 2.83687 45960000.000 5.47 2.95 2.72 5.68 5.25

3603000.000 2.83714 46030000.000 5.47 2.95 2.72 5.68 5.25

3610000.000 2.83742 46100000.000 5.47 2.95 2.72 5.68 5.25

3617000.000 2.83770 46170000.000 5.47 2.95 2.72 5.68 5.25

3624000.000 2.83797 46240000.000 5.47 2.95 2.72 5.69 5.25

3631000.000 2.83825 46310000.000 5.47 2.95 2.72 5.69 5.25

3638000.000 2.83852 46380000.000 5.47 2.95 2.72 5.69 5.25

3645000.000 2.83879 46450000.000 5.47 2.95 2.73 5.69 5.25

3652000.000 2.83907 46520000.000 5.47 2.95 2.73 5.69 5.25

3659000.000 2.83934 46590000.000 5.47 2.95 2.73 5.69 5.25

3666000.000 2.83961 46660000.000 5.47 2.95 2.73 5.69 5.25

3673000.000 2.83988 46730000.000 5.47 2.95 2.73 5.69 5.25

3680000.000 2.84015 46800000.000 5.47 2.95 2.73 5.69 5.25

3687000.000 2.84043 46870000.000 5.47 2.95 2.73 5.69 5.25

3694000.000 2.84070 46940000.000 5.47 2.95 2.73 5.69 5.25

3701000.000 2.84097 47010000.000 5.47 2.95 2.73 5.69 5.25

3708000.000 2.84123 47080000.000 5.47 2.95 2.73 5.69 5.25

3715000.000 2.84150 47150000.000 5.47 2.96 2.73 5.69 5.25

3722000.000 2.84177 47220000.000 5.47 2.96 2.73 5.69 5.25

3729000.000 2.84204 47290000.000 5.47 2.96 2.73 5.69 5.25

3736000.000 2.84231 47360000.000 5.47 2.96 2.73 5.69 5.25

3743000.000 2.84257 47430000.000 5.47 2.96 2.73 5.69 5.25

3750000.000 2.84284 47500000.000 5.47 2.96 2.73 5.69 5.26

3757000.000 2.84311 47570000.000 5.47 2.96 2.73 5.69 5.26

3764000.000 2.84337 47640000.000 5.47 2.96 2.73 5.69 5.26

3771000.000 2.84364 47710000.000 5.48 2.96 2.73 5.69 5.26

3778000.000 2.84390 47780000.000 5.48 2.96 2.73 5.69 5.26

3785000.000 2.84417 47850000.000 5.48 2.96 2.73 5.70 5.26

3792000.000 2.84443 47920000.000 5.48 2.96 2.73 5.70 5.26

3799000.000 2.84469 47990000.000 5.48 2.96 2.73 5.70 5.26

3806000.000 2.84495 48060000.000 5.48 2.96 2.73 5.70 5.26

3813000.000 2.84522 48130000.000 5.48 2.96 2.73 5.70 5.26

3820000.000 2.84548 48200000.000 5.48 2.96 2.73 5.70 5.26

3827000.000 2.84574 48270000.000 5.48 2.96 2.73 5.70 5.26

3834000.000 2.84600 48340000.000 5.48 2.96 2.73 5.70 5.26

3841000.000 2.84626 48410000.000 5.48 2.96 2.73 5.70 5.26

3848000.000 2.84652 48480000.000 5.48 2.96 2.73 5.70 5.26

3855000.000 2.84678 48550000.000 5.48 2.96 2.73 5.70 5.26

3862000.000 2.84704 48620000.000 5.48 2.96 2.73 5.70 5.26

3869000.000 2.84730 48690000.000 5.48 2.96 2.73 5.70 5.26

3876000.000 2.84756 48760000.000 5.48 2.96 2.73 5.70 5.26

3883000.000 2.84781 48830000.000 5.48 2.96 2.73 5.70 5.26

3890000.000 2.84807 48900000.000 5.48 2.96 2.73 5.70 5.26

3897000.000 2.84833 48970000.000 5.48 2.96 2.73 5.70 5.26

3904000.000 2.84858 49040000.000 5.48 2.96 2.73 5.70 5.26

3911000.000 2.84884 49110000.000 5.48 2.96 2.73 5.70 5.26

3918000.000 2.84910 49180000.000 5.48 2.96 2.74 5.70 5.26

3925000.000 2.84935 49250000.000 5.48 2.96 2.74 5.70 5.26

3932000.000 2.84961 49320000.000 5.48 2.96 2.74 5.70 5.27

3939000.000 2.84986 49390000.000 5.48 2.96 2.74 5.70 5.27

3946000.000 2.85011 49460000.000 5.49 2.96 2.74 5.70 5.27

3953000.000 2.85037 49530000.000 5.49 2.96 2.74 5.71 5.27

3960000.000 2.85062 49600000.000 5.49 2.96 2.74 5.71 5.27

3967000.000 2.85087 49670000.000 5.49 2.96 2.74 5.71 5.27

3974000.000 2.85113 49740000.000 5.49 2.97 2.74 5.71 5.27

3981000.000 2.85138 49810000.000 5.49 2.97 2.74 5.71 5.27

3988000.000 2.85163 49880000.000 5.49 2.97 2.74 5.71 5.27

3995000.000 2.85188 49950000.000 5.49 2.97 2.74 5.71 5.27

4002000.000 2.85213 50020000.000 5.49 2.97 2.74 5.71 5.27

4009000.000 2.85238 50090000.000 5.49 2.97 2.74 5.71 5.27

4016000.000 2.85263 50160000.000 5.49 2.97 2.74 5.71 5.27

4023000.000 2.85288 50230000.000 5.49 2.97 2.74 5.71 5.27

4030000.000 2.85313 50300000.000 5.49 2.97 2.74 5.71 5.27

4037000.000 2.85338 50370000.000 5.49 2.97 2.74 5.71 5.27

4044000.000 2.85362 50440000.000 5.49 2.97 2.74 5.71 5.27

4051000.000 2.85387 50510000.000 5.49 2.97 2.74 5.71 5.27

4058000.000 2.85412 50580000.000 5.49 2.97 2.74 5.71 5.27

4065000.000 2.85437 50650000.000 5.49 2.97 2.74 5.71 5.27

4072000.000 2.85461 50720000.000 5.49 2.97 2.74 5.71 5.27

4079000.000 2.85486 50790000.000 5.49 2.97 2.74 5.71 5.27

4086000.000 2.85510 50860000.000 5.49 2.97 2.74 5.71 5.27

4093000.000 2.85535 50930000.000 5.49 2.97 2.74 5.71 5.27

4100000.000 2.85559 51000000.000 5.49 2.97 2.74 5.71 5.27

4107000.000 2.85584 51070000.000 5.49 2.97 2.74 5.71 5.27

4114000.000 2.85608 51140000.000 5.49 2.97 2.74 5.71 5.27

4121000.000 2.85632 51210000.000 5.49 2.97 2.74 5.71 5.27

4128000.000 2.85657 51280000.000 5.50 2.97 2.74 5.71 5.28

4135000.000 2.85681 51350000.000 5.50 2.97 2.74 5.72 5.28

4142000.000 2.85705 51420000.000 5.50 2.97 2.74 5.72 5.28

4149000.000 2.85730 51490000.000 5.50 2.97 2.74 5.72 5.28

4156000.000 2.85754 51560000.000 5.50 2.97 2.74 5.72 5.28

4163000.000 2.85778 51630000.000 5.50 2.97 2.74 5.72 5.28

4170000.000 2.85802 51700000.000 5.50 2.97 2.74 5.72 5.28

4177000.000 2.85826 51770000.000 5.50 2.97 2.74 5.72 5.28

4184000.000 2.85850 51840000.000 5.50 2.97 2.74 5.72 5.28

4191000.000 2.85874 51910000.000 5.50 2.97 2.74 5.72 5.28

4198000.000 2.85898 51980000.000 5.50 2.97 2.74 5.72 5.28

4205000.000 2.85922 52050000.000 5.50 2.97 2.74 5.72 5.28

4212000.000 2.85946 52120000.000 5.50 2.97 2.75 5.72 5.28

4219000.000 2.85969 52190000.000 5.50 2.97 2.75 5.72 5.28

4226000.000 2.85993 52260000.000 5.50 2.97 2.75 5.72 5.28

4233000.000 2.86017 52330000.000 5.50 2.97 2.75 5.72 5.28

4240000.000 2.86041 52400000.000 5.50 2.97 2.75 5.72 5.28

4247000.000 2.86064 52470000.000 5.50 2.98 2.75 5.72 5.28

4254000.000 2.86088 52540000.000 5.50 2.98 2.75 5.72 5.28

4261000.000 2.86112 52610000.000 5.50 2.98 2.75 5.72 5.28

4268000.000 2.86135 52680000.000 5.50 2.98 2.75 5.72 5.28

4275000.000 2.86159 52750000.000 5.50 2.98 2.75 5.72 5.28

4282000.000 2.86182 52820000.000 5.50 2.98 2.75 5.72 5.28

4289000.000 2.86206 52890000.000 5.50 2.98 2.75 5.72 5.28

4296000.000 2.86229 52960000.000 5.50 2.98 2.75 5.72 5.28

4303000.000 2.86252 53030000.000 5.50 2.98 2.75 5.72 5.28

4310000.000 2.86276 53100000.000 5.50 2.98 2.75 5.72 5.28

4317000.000 2.86299 53170000.000 5.51 2.98 2.75 5.73 5.28

4324000.000 2.86322 53240000.000 5.51 2.98 2.75 5.73 5.29

4331000.000 2.86345 53310000.000 5.51 2.98 2.75 5.73 5.29

4338000.000 2.86369 53380000.000 5.51 2.98 2.75 5.73 5.29

4345000.000 2.86392 53450000.000 5.51 2.98 2.75 5.73 5.29

4352000.000 2.86415 53520000.000 5.51 2.98 2.75 5.73 5.29

4359000.000 2.86438 53590000.000 5.51 2.98 2.75 5.73 5.29

4366000.000 2.86461 53660000.000 5.51 2.98 2.75 5.73 5.29

4373000.000 2.86484 53730000.000 5.51 2.98 2.75 5.73 5.29

4380000.000 2.86507 53800000.000 5.51 2.98 2.75 5.73 5.29

4387000.000 2.86530 53870000.000 5.51 2.98 2.75 5.73 5.29

4394000.000 2.86553 53940000.000 5.51 2.98 2.75 5.73 5.29

4401000.000 2.86576 54010000.000 5.51 2.98 2.75 5.73 5.29

4408000.000 2.86599 54080000.000 5.51 2.98 2.75 5.73 5.29

4415000.000 2.86622 54150000.000 5.51 2.98 2.75 5.73 5.29

4422000.000 2.86644 54220000.000 5.51 2.98 2.75 5.73 5.29

4429000.000 2.86667 54290000.000 5.51 2.98 2.75 5.73 5.29

4436000.000 2.86690 54360000.000 5.51 2.98 2.75 5.73 5.29

4443000.000 2.86712 54430000.000 5.51 2.98 2.75 5.73 5.29

4450000.000 2.86735 54500000.000 5.51 2.98 2.75 5.73 5.29

4457000.000 2.86758 54570000.000 5.51 2.98 2.75 5.73 5.29

4464000.000 2.86780 54640000.000 5.51 2.98 2.75 5.73 5.29

4471000.000 2.86803 54710000.000 5.51 2.98 2.75 5.73 5.29

4478000.000 2.86825 54780000.000 5.51 2.98 2.75 5.73 5.29

4485000.000 2.86848 54850000.000 5.51 2.98 2.75 5.73 5.29

4492000.000 2.86870 54920000.000 5.51 2.98 2.75 5.73 5.29

4499000.000 2.86893 54990000.000 5.51 2.98 2.75 5.74 5.29

4506000.000 2.86915 55060000.000 5.51 2.98 2.75 5.74 5.29

4513000.000 2.86937 55130000.000 5.52 2.98 2.75 5.74 5.29

4520000.000 2.86960 55200000.000 5.52 2.98 2.75 5.74 5.29

4527000.000 2.86982 55270000.000 5.52 2.98 2.76 5.74 5.30

4534000.000 2.87004 55340000.000 5.52 2.98 2.76 5.74 5.30

4541000.000 2.87026 55410000.000 5.52 2.99 2.76 5.74 5.30

4548000.000 2.87049 55480000.000 5.52 2.99 2.76 5.74 5.30

4555000.000 2.87071 55550000.000 5.52 2.99 2.76 5.74 5.30

4562000.000 2.87093 55620000.000 5.52 2.99 2.76 5.74 5.30

4569000.000 2.87115 55690000.000 5.52 2.99 2.76 5.74 5.30

4576000.000 2.87137 55760000.000 5.52 2.99 2.76 5.74 5.30

4583000.000 2.87159 55830000.000 5.52 2.99 2.76 5.74 5.30

4590000.000 2.87181 55900000.000 5.52 2.99 2.76 5.74 5.30

4597000.000 2.87203 55970000.000 5.52 2.99 2.76 5.74 5.30

4604000.000 2.87225 56040000.000 5.52 2.99 2.76 5.74 5.30

4611000.000 2.87247 56110000.000 5.52 2.99 2.76 5.74 5.30

4618000.000 2.87269 56180000.000 5.52 2.99 2.76 5.74 5.30

4625000.000 2.87290 56250000.000 5.52 2.99 2.76 5.74 5.30

4632000.000 2.87312 56320000.000 5.52 2.99 2.76 5.74 5.30

4639000.000 2.87334 56390000.000 5.52 2.99 2.76 5.74 5.30

4646000.000 2.87356 56460000.000 5.52 2.99 2.76 5.74 5.30

4653000.000 2.87377 56530000.000 5.52 2.99 2.76 5.74 5.30

4660000.000 2.87399 56600000.000 5.52 2.99 2.76 5.74 5.30

4667000.000 2.87421 56670000.000 5.52 2.99 2.76 5.74 5.30

4674000.000 2.87442 56740000.000 5.52 2.99 2.76 5.74 5.30

4681000.000 2.87464 56810000.000 5.52 2.99 2.76 5.74 5.30

4688000.000 2.87485 56880000.000 5.52 2.99 2.76 5.74 5.30

4695000.000 2.87507 56950000.000 5.52 2.99 2.76 5.75 5.30

4702000.000 2.87528 57020000.000 5.52 2.99 2.76 5.75 5.30

4709000.000 2.87550 57090000.000 5.52 2.99 2.76 5.75 5.30

4716000.000 2.87571 57160000.000 5.53 2.99 2.76 5.75 5.30

4723000.000 2.87593 57230000.000 5.53 2.99 2.76 5.75 5.30

4730000.000 2.87614 57300000.000 5.53 2.99 2.76 5.75 5.30

4737000.000 2.87635 57370000.000 5.53 2.99 2.76 5.75 5.31

4744000.000 2.87657 57440000.000 5.53 2.99 2.76 5.75 5.31

4751000.000 2.87678 57510000.000 5.53 2.99 2.76 5.75 5.31

4758000.000 2.87699 57580000.000 5.53 2.99 2.76 5.75 5.31

4765000.000 2.87720 57650000.000 5.53 2.99 2.76 5.75 5.31

4772000.000 2.87742 57720000.000 5.53 2.99 2.76 5.75 5.31

4779000.000 2.87763 57790000.000 5.53 2.99 2.76 5.75 5.31

4786000.000 2.87784 57860000.000 5.53 2.99 2.76 5.75 5.31

4793000.000 2.87805 57930000.000 5.53 2.99 2.76 5.75 5.31

4800000.000 2.87826 58000000.000 5.53 2.99 2.76 5.75 5.31

4807000.000 2.87847 58070000.000 5.53 2.99 2.76 5.75 5.31

4814000.000 2.87868 58140000.000 5.53 2.99 2.76 5.75 5.31

4821000.000 2.87889 58210000.000 5.53 2.99 2.76 5.75 5.31

4828000.000 2.87910 58280000.000 5.53 2.99 2.76 5.75 5.31

4835000.000 2.87931 58350000.000 5.53 2.99 2.76 5.75 5.31

4842000.000 2.87952 58420000.000 5.53 2.99 2.76 5.75 5.31

4849000.000 2.87973 58490000.000 5.53 2.99 2.76 5.75 5.31

4856000.000 2.87994 58560000.000 5.53 3.00 2.76 5.75 5.31

4863000.000 2.88014 58630000.000 5.53 3.00 2.76 5.75 5.31

4870000.000 2.88035 58700000.000 5.53 3.00 2.77 5.75 5.31

4877000.000 2.88056 58770000.000 5.53 3.00 2.77 5.75 5.31

4884000.000 2.88077 58840000.000 5.53 3.00 2.77 5.75 5.31

4891000.000 2.88097 58910000.000 5.53 3.00 2.77 5.75 5.31

4898000.000 2.88118 58980000.000 5.53 3.00 2.77 5.76 5.31

4905000.000 2.88139 59050000.000 5.53 3.00 2.77 5.76 5.31

4912000.000 2.88159 59120000.000 5.53 3.00 2.77 5.76 5.31

4919000.000 2.88180 59190000.000 5.53 3.00 2.77 5.76 5.31

4926000.000 2.88200 59260000.000 5.54 3.00 2.77 5.76 5.31

4933000.000 2.88221 59330000.000 5.54 3.00 2.77 5.76 5.31

4940000.000 2.88241 59400000.000 5.54 3.00 2.77 5.76 5.31

4947000.000 2.88262 59470000.000 5.54 3.00 2.77 5.76 5.31

4954000.000 2.88282 59540000.000 5.54 3.00 2.77 5.76 5.32

4961000.000 2.88303 59610000.000 5.54 3.00 2.77 5.76 5.32

4968000.000 2.88323 59680000.000 5.54 3.00 2.77 5.76 5.32

4975000.000 2.88344 59750000.000 5.54 3.00 2.77 5.76 5.32

4982000.000 2.88364 59820000.000 5.54 3.00 2.77 5.76 5.32

4989000.000 2.88384 59890000.000 5.54 3.00 2.77 5.76 5.32

4996000.000 2.88404 59960000.000 5.54 3.00 2.77 5.76 5.32

5003000.000 2.88425 60030000.000 5.54 3.00 2.77 5.76 5.32

5010000.000 2.88445 60100000.000 5.54 3.00 2.77 5.76 5.32

5017000.000 2.88465 60170000.000 5.54 3.00 2.77 5.76 5.32

5024000.000 2.88485 60240000.000 5.54 3.00 2.77 5.76 5.32

5031000.000 2.88505 60310000.000 5.54 3.00 2.77 5.76 5.32

5038000.000 2.88526 60380000.000 5.54 3.00 2.77 5.76 5.32

5045000.000 2.88546 60450000.000 5.54 3.00 2.77 5.76 5.32

5052000.000 2.88566 60520000.000 5.54 3.00 2.77 5.76 5.32

5059000.000 2.88586 60590000.000 5.54 3.00 2.77 5.76 5.32

5066000.000 2.88606 60660000.000 5.54 3.00 2.77 5.76 5.32

5073000.000 2.88626 60730000.000 5.54 3.00 2.77 5.76 5.32

5080000.000 2.88646 60800000.000 5.54 3.00 2.77 5.76 5.32

5087000.000 2.88666 60870000.000 5.54 3.00 2.77 5.76 5.32

5094000.000 2.88686 60940000.000 5.54 3.00 2.77 5.76 5.32

5101000.000 2.88705 61010000.000 5.54 3.00 2.77 5.76 5.32

5108000.000 2.88725 61080000.000 5.54 3.00 2.77 5.77 5.32

5115000.000 2.88745 61150000.000 5.54 3.00 2.77 5.77 5.32

5122000.000 2.88765 61220000.000 5.54 3.00 2.77 5.77 5.32

5129000.000 2.88785 61290000.000 5.54 3.00 2.77 5.77 5.32

5136000.000 2.88805 61360000.000 5.54 3.00 2.77 5.77 5.32

5143000.000 2.88824 61430000.000 5.55 3.00 2.77 5.77 5.32

5150000.000 2.88844 61500000.000 5.55 3.00 2.77 5.77 5.32

5157000.000 2.88864 61570000.000 5.55 3.00 2.77 5.77 5.32

5164000.000 2.88883 61640000.000 5.55 3.00 2.77 5.77 5.32

5171000.000 2.88903 61710000.000 5.55 3.00 2.77 5.77 5.32

5178000.000 2.88923 61780000.000 5.55 3.00 2.77 5.77 5.32

5185000.000 2.88942 61850000.000 5.55 3.00 2.77 5.77 5.33

5192000.000 2.88962 61920000.000 5.55 3.01 2.77 5.77 5.33

5199000.000 2.88981 61990000.000 5.55 3.01 2.77 5.77 5.33

5206000.000 2.89001 62060000.000 5.55 3.01 2.77 5.77 5.33

5213000.000 2.89020 62130000.000 5.55 3.01 2.77 5.77 5.33

5220000.000 2.89040 62200000.000 5.55 3.01 2.77 5.77 5.33

5227000.000 2.89059 62270000.000 5.55 3.01 2.77 5.77 5.33

5234000.000 2.89078 62340000.000 5.55 3.01 2.78 5.77 5.33

5241000.000 2.89098 62410000.000 5.55 3.01 2.78 5.77 5.33

5248000.000 2.89117 62480000.000 5.55 3.01 2.78 5.77 5.33

5255000.000 2.89137 62550000.000 5.55 3.01 2.78 5.77 5.33

5262000.000 2.89156 62620000.000 5.55 3.01 2.78 5.77 5.33

5269000.000 2.89175 62690000.000 5.55 3.01 2.78 5.77 5.33

5276000.000 2.89194 62760000.000 5.55 3.01 2.78 5.77 5.33

5283000.000 2.89214 62830000.000 5.55 3.01 2.78 5.77 5.33

5290000.000 2.89233 62900000.000 5.55 3.01 2.78 5.77 5.33

5297000.000 2.89252 62970000.000 5.55 3.01 2.78 5.77 5.33

5304000.000 2.89271 63040000.000 5.55 3.01 2.78 5.77 5.33

5311000.000 2.89290 63110000.000 5.55 3.01 2.78 5.77 5.33

5318000.000 2.89309 63180000.000 5.55 3.01 2.78 5.78 5.33

5325000.000 2.89329 63250000.000 5.55 3.01 2.78 5.78 5.33

5332000.000 2.89348 63320000.000 5.55 3.01 2.78 5.78 5.33

5339000.000 2.89367 63390000.000 5.55 3.01 2.78 5.78 5.33

5346000.000 2.89386 63460000.000 5.55 3.01 2.78 5.78 5.33

5353000.000 2.89405 63530000.000 5.55 3.01 2.78 5.78 5.33

5360000.000 2.89424 63600000.000 5.55 3.01 2.78 5.78 5.33

5367000.000 2.89443 63670000.000 5.56 3.01 2.78 5.78 5.33

5374000.000 2.89461 63740000.000 5.56 3.01 2.78 5.78 5.33

5381000.000 2.89480 63810000.000 5.56 3.01 2.78 5.78 5.33

5388000.000 2.89499 63880000.000 5.56 3.01 2.78 5.78 5.33

5395000.000 2.89518 63950000.000 5.56 3.01 2.78 5.78 5.33

5402000.000 2.89537 64020000.000 5.56 3.01 2.78 5.78 5.33

5409000.000 2.89556 64090000.000 5.56 3.01 2.78 5.78 5.33

5416000.000 2.89575 64160000.000 5.56 3.01 2.78 5.78 5.33

5423000.000 2.89593 64230000.000 5.56 3.01 2.78 5.78 5.34

5430000.000 2.89612 64300000.000 5.56 3.01 2.78 5.78 5.34

5437000.000 2.89631 64370000.000 5.56 3.01 2.78 5.78 5.34

5444000.000 2.89649 64440000.000 5.56 3.01 2.78 5.78 5.34

5451000.000 2.89668 64510000.000 5.56 3.01 2.78 5.78 5.34

5458000.000 2.89687 64580000.000 5.56 3.01 2.78 5.78 5.34

5465000.000 2.89705 64650000.000 5.56 3.01 2.78 5.78 5.34

5472000.000 2.89724 64720000.000 5.56 3.01 2.78 5.78 5.34

5479000.000 2.89743 64790000.000 5.56 3.01 2.78 5.78 5.34

5486000.000 2.89761 64860000.000 5.56 3.01 2.78 5.78 5.34

5493000.000 2.89780 64930000.000 5.56 3.01 2.78 5.78 5.34

5500000.000 2.89798 65000000.000 5.56 3.01 2.78 5.78 5.34

5507000.000 2.89817 65070000.000 5.56 3.01 2.78 5.78 5.34

5514000.000 2.89835 65140000.000 5.56 3.01 2.78 5.78 5.34

5521000.000 2.89854 65210000.000 5.56 3.01 2.78 5.78 5.34

5528000.000 2.89872 65280000.000 5.56 3.01 2.78 5.78 5.34

5535000.000 2.89890 65350000.000 5.56 3.01 2.78 5.78 5.34

5542000.000 2.89909 65420000.000 5.56 3.02 2.78 5.79 5.34

5549000.000 2.89927 65490000.000 5.56 3.02 2.78 5.79 5.34

5556000.000 2.89945 65560000.000 5.56 3.02 2.78 5.79 5.34

5563000.000 2.89964 65630000.000 5.56 3.02 2.78 5.79 5.34

5570000.000 2.89982 65700000.000 5.56 3.02 2.78 5.79 5.34

5577000.000 2.90000 65770000.000 5.56 3.02 2.78 5.79 5.34

5584000.000 2.90019 65840000.000 5.56 3.02 2.78 5.79 5.34

5591000.000 2.90037 65910000.000 5.56 3.02 2.78 5.79 5.34

5598000.000 2.90055 65980000.000 5.57 3.02 2.78 5.79 5.34

5605000.000 2.90073 66050000.000 5.57 3.02 2.78 5.79 5.34

5612000.000 2.90091 66120000.000 5.57 3.02 2.78 5.79 5.34

5619000.000 2.90109 66190000.000 5.57 3.02 2.79 5.79 5.34

5626000.000 2.90128 66260000.000 5.57 3.02 2.79 5.79 5.34

5633000.000 2.90146 66330000.000 5.57 3.02 2.79 5.79 5.34

5640000.000 2.90164 66400000.000 5.57 3.02 2.79 5.79 5.34

5647000.000 2.90182 66470000.000 5.57 3.02 2.79 5.79 5.34

5654000.000 2.90200 66540000.000 5.57 3.02 2.79 5.79 5.34

5661000.000 2.90218 66610000.000 5.57 3.02 2.79 5.79 5.35

5668000.000 2.90236 66680000.000 5.57 3.02 2.79 5.79 5.35

5675000.000 2.90254 66750000.000 5.57 3.02 2.79 5.79 5.35

5682000.000 2.90272 66820000.000 5.57 3.02 2.79 5.79 5.35

5689000.000 2.90290 66890000.000 5.57 3.02 2.79 5.79 5.35

5696000.000 2.90308 66960000.000 5.57 3.02 2.79 5.79 5.35

5703000.000 2.90326 67030000.000 5.57 3.02 2.79 5.79 5.35

5710000.000 2.90343 67100000.000 5.57 3.02 2.79 5.79 5.35

5717000.000 2.90361 67170000.000 5.57 3.02 2.79 5.79 5.35

5724000.000 2.90379 67240000.000 5.57 3.02 2.79 5.79 5.35

5731000.000 2.90397 67310000.000 5.57 3.02 2.79 5.79 5.35

5738000.000 2.90415 67380000.000 5.57 3.02 2.79 5.79 5.35

5745000.000 2.90432 67450000.000 5.57 3.02 2.79 5.79 5.35

5752000.000 2.90450 67520000.000 5.57 3.02 2.79 5.79 5.35

5759000.000 2.90468 67590000.000 5.57 3.02 2.79 5.79 5.35

5766000.000 2.90486 67660000.000 5.57 3.02 2.79 5.79 5.35

5773000.000 2.90503 67730000.000 5.57 3.02 2.79 5.80 5.35

5780000.000 2.90521 67800000.000 5.57 3.02 2.79 5.80 5.35

5787000.000 2.90539 67870000.000 5.57 3.02 2.79 5.80 5.35

5794000.000 2.90556 67940000.000 5.57 3.02 2.79 5.80 5.35

5801000.000 2.90574 68010000.000 5.57 3.02 2.79 5.80 5.35

5808000.000 2.90591 68080000.000 5.57 3.02 2.79 5.80 5.35

5815000.000 2.90609 68150000.000 5.57 3.02 2.79 5.80 5.35

5822000.000 2.90626 68220000.000 5.57 3.02 2.79 5.80 5.35

5829000.000 2.90644 68290000.000 5.57 3.02 2.79 5.80 5.35

5836000.000 2.90661 68360000.000 5.57 3.02 2.79 5.80 5.35

5843000.000 2.90679 68430000.000 5.58 3.02 2.79 5.80 5.35

5850000.000 2.90696 68500000.000 5.58 3.02 2.79 5.80 5.35

5857000.000 2.90714 68570000.000 5.58 3.02 2.79 5.80 5.35

5864000.000 2.90731 68640000.000 5.58 3.02 2.79 5.80 5.35

5871000.000 2.90749 68710000.000 5.58 3.02 2.79 5.80 5.35

5878000.000 2.90766 68780000.000 5.58 3.02 2.79 5.80 5.35

5885000.000 2.90783 68850000.000 5.58 3.02 2.79 5.80 5.35

5892000.000 2.90801 68920000.000 5.58 3.02 2.79 5.80 5.35

5899000.000 2.90818 68990000.000 5.58 3.02 2.79 5.80 5.35

5906000.000 2.90835 69060000.000 5.58 3.02 2.79 5.80 5.35

5913000.000 2.90853 69130000.000 5.58 3.02 2.79 5.80 5.35

5920000.000 2.90870 69200000.000 5.58 3.03 2.79 5.80 5.36

5927000.000 2.90887 69270000.000 5.58 3.03 2.79 5.80 5.36

5934000.000 2.90904 69340000.000 5.58 3.03 2.79 5.80 5.36

5941000.000 2.90922 69410000.000 5.58 3.03 2.79 5.80 5.36

5948000.000 2.90939 69480000.000 5.58 3.03 2.79 5.80 5.36

5955000.000 2.90956 69550000.000 5.58 3.03 2.79 5.80 5.36

5962000.000 2.90973 69620000.000 5.58 3.03 2.79 5.80 5.36

5969000.000 2.90990 69690000.000 5.58 3.03 2.79 5.80 5.36

5976000.000 2.91007 69760000.000 5.58 3.03 2.79 5.80 5.36

5983000.000 2.91024 69830000.000 5.58 3.03 2.79 5.80 5.36

5990000.000 2.91041 69900000.000 5.58 3.03 2.79 5.80 5.36

5997000.000 2.91058 69970000.000 5.58 3.03 2.79 5.80 5.36

6004000.000 2.91075 70040000.000 5.58 3.03 2.79 5.81 5.36

6011000.000 2.91093 70110000.000 5.58 3.03 2.79 5.81 5.36

6018000.000 2.91109 70180000.000 5.58 3.03 2.79 5.81 5.36

6025000.000 2.91126 70250000.000 5.58 3.03 2.79 5.81 5.36

6032000.000 2.91143 70320000.000 5.58 3.03 2.79 5.81 5.36

6039000.000 2.91160 70390000.000 5.58 3.03 2.80 5.81 5.36

6046000.000 2.91177 70460000.000 5.58 3.03 2.80 5.81 5.36

6053000.000 2.91194 70530000.000 5.58 3.03 2.80 5.81 5.36

6060000.000 2.91211 70600000.000 5.58 3.03 2.80 5.81 5.36

6067000.000 2.91228 70670000.000 5.58 3.03 2.80 5.81 5.36

6074000.000 2.91245 70740000.000 5.58 3.03 2.80 5.81 5.36

6081000.000 2.91262 70810000.000 5.58 3.03 2.80 5.81 5.36

6088000.000 2.91278 70880000.000 5.59 3.03 2.80 5.81 5.36

6095000.000 2.91295 70950000.000 5.59 3.03 2.80 5.81 5.36

6102000.000 2.91312 71020000.000 5.59 3.03 2.80 5.81 5.36

6109000.000 2.91329 71090000.000 5.59 3.03 2.80 5.81 5.36

6116000.000 2.91345 71160000.000 5.59 3.03 2.80 5.81 5.36

6123000.000 2.91362 71230000.000 5.59 3.03 2.80 5.81 5.36

6130000.000 2.91379 71300000.000 5.59 3.03 2.80 5.81 5.36

6137000.000 2.91396 71370000.000 5.59 3.03 2.80 5.81 5.36

6144000.000 2.91412 71440000.000 5.59 3.03 2.80 5.81 5.36

6151000.000 2.91429 71510000.000 5.59 3.03 2.80 5.81 5.36

6158000.000 2.91445 71580000.000 5.59 3.03 2.80 5.81 5.36

6165000.000 2.91462 71650000.000 5.59 3.03 2.80 5.81 5.36

6172000.000 2.91479 71720000.000 5.59 3.03 2.80 5.81 5.36

6179000.000 2.91495 71790000.000 5.59 3.03 2.80 5.81 5.37

6186000.000 2.91512 71860000.000 5.59 3.03 2.80 5.81 5.37

6193000.000 2.91528 71930000.000 5.59 3.03 2.80 5.81 5.37

6200000.000 2.91545 72000000.000 5.59 3.03 2.80 5.81 5.37

6207000.000 2.91561 72070000.000 5.59 3.03 2.80 5.81 5.37

6214000.000 2.91578 72140000.000 5.59 3.03 2.80 5.81 5.37

6221000.000 2.91594 72210000.000 5.59 3.03 2.80 5.81 5.37

6228000.000 2.91611 72280000.000 5.59 3.03 2.80 5.81 5.37

6235000.000 2.91627 72350000.000 5.59 3.03 2.80 5.81 5.37

6242000.000 2.91644 72420000.000 5.59 3.03 2.80 5.81 5.37

6249000.000 2.91660 72490000.000 5.59 3.03 2.80 5.82 5.37

6256000.000 2.91676 72560000.000 5.59 3.03 2.80 5.82 5.37

6263000.000 2.91693 72630000.000 5.59 3.03 2.80 5.82 5.37

6270000.000 2.91709 72700000.000 5.59 3.03 2.80 5.82 5.37

6277000.000 2.91725 72770000.000 5.59 3.03 2.80 5.82 5.37

6284000.000 2.91742 72840000.000 5.59 3.03 2.80 5.82 5.37

6291000.000 2.91758 72910000.000 5.59 3.03 2.80 5.82 5.37

6298000.000 2.91774 72980000.000 5.59 3.03 2.80 5.82 5.37

6305000.000 2.91791 73050000.000 5.59 3.03 2.80 5.82 5.37

6312000.000 2.91807 73120000.000 5.59 3.03 2.80 5.82 5.37

6319000.000 2.91823 73190000.000 5.59 3.03 2.80 5.82 5.37

6326000.000 2.91839 73260000.000 5.59 3.04 2.80 5.82 5.37

6333000.000 2.91855 73330000.000 5.59 3.04 2.80 5.82 5.37

6340000.000 2.91872 73400000.000 5.59 3.04 2.80 5.82 5.37

6347000.000 2.91888 73470000.000 5.60 3.04 2.80 5.82 5.37

6354000.000 2.91904 73540000.000 5.60 3.04 2.80 5.82 5.37

6361000.000 2.91920 73610000.000 5.60 3.04 2.80 5.82 5.37

6368000.000 2.91936 73680000.000 5.60 3.04 2.80 5.82 5.37

6375000.000 2.91952 73750000.000 5.60 3.04 2.80 5.82 5.37

6382000.000 2.91968 73820000.000 5.60 3.04 2.80 5.82 5.37

6389000.000 2.91984 73890000.000 5.60 3.04 2.80 5.82 5.37

6396000.000 2.92000 73960000.000 5.60 3.04 2.80 5.82 5.37

6403000.000 2.92016 74030000.000 5.60 3.04 2.80 5.82 5.37

6410000.000 2.92032 74100000.000 5.60 3.04 2.80 5.82 5.37

6417000.000 2.92048 74170000.000 5.60 3.04 2.80 5.82 5.37

6424000.000 2.92064 74240000.000 5.60 3.04 2.80 5.82 5.37

6431000.000 2.92080 74310000.000 5.60 3.04 2.80 5.82 5.37

6438000.000 2.92096 74380000.000 5.60 3.04 2.80 5.82 5.37

6445000.000 2.92112 74450000.000 5.60 3.04 2.80 5.82 5.37

6452000.000 2.92128 74520000.000 5.60 3.04 2.80 5.82 5.38

6459000.000 2.92144 74590000.000 5.60 3.04 2.80 5.82 5.38

6466000.000 2.92160 74660000.000 5.60 3.04 2.80 5.82 5.38

6473000.000 2.92176 74730000.000 5.60 3.04 2.80 5.82 5.38

6480000.000 2.92192 74800000.000 5.60 3.04 2.81 5.82 5.38

6487000.000 2.92207 74870000.000 5.60 3.04 2.81 5.82 5.38

6494000.000 2.92223 74940000.000 5.60 3.04 2.81 5.82 5.38

6501000.000 2.92239 75010000.000 5.60 3.04 2.81 5.83 5.38

6508000.000 2.92255 75080000.000 5.60 3.04 2.81 5.83 5.38

6515000.000 2.92271 75150000.000 5.60 3.04 2.81 5.83 5.38

6522000.000 2.92286 75220000.000 5.60 3.04 2.81 5.83 5.38

6529000.000 2.92302 75290000.000 5.60 3.04 2.81 5.83 5.38

6536000.000 2.92318 75360000.000 5.60 3.04 2.81 5.83 5.38

6543000.000 2.92333 75430000.000 5.60 3.04 2.81 5.83 5.38

6550000.000 2.92349 75500000.000 5.60 3.04 2.81 5.83 5.38

6557000.000 2.92365 75570000.000 5.60 3.04 2.81 5.83 5.38

6564000.000 2.92380 75640000.000 5.60 3.04 2.81 5.83 5.38

6571000.000 2.92396 75710000.000 5.60 3.04 2.81 5.83 5.38

6578000.000 2.92412 75780000.000 5.60 3.04 2.81 5.83 5.38

6585000.000 2.92427 75850000.000 5.60 3.04 2.81 5.83 5.38

6592000.000 2.92443 75920000.000 5.60 3.04 2.81 5.83 5.38

6599000.000 2.92458 75990000.000 5.60 3.04 2.81 5.83 5.38

6606000.000 2.92474 76060000.000 5.60 3.04 2.81 5.83 5.38

6613000.000 2.92489 76130000.000 5.61 3.04 2.81 5.83 5.38

6620000.000 2.92505 76200000.000 5.61 3.04 2.81 5.83 5.38

6627000.000 2.92521 76270000.000 5.61 3.04 2.81 5.83 5.38

6634000.000 2.92536 76340000.000 5.61 3.04 2.81 5.83 5.38

6641000.000 2.92551 76410000.000 5.61 3.04 2.81 5.83 5.38

6648000.000 2.92567 76480000.000 5.61 3.04 2.81 5.83 5.38

6655000.000 2.92582 76550000.000 5.61 3.04 2.81 5.83 5.38

6662000.000 2.92598 76620000.000 5.61 3.04 2.81 5.83 5.38

6669000.000 2.92613 76690000.000 5.61 3.04 2.81 5.83 5.38

6676000.000 2.92629 76760000.000 5.61 3.04 2.81 5.83 5.38

6683000.000 2.92644 76830000.000 5.61 3.04 2.81 5.83 5.38

6690000.000 2.92659 76900000.000 5.61 3.04 2.81 5.83 5.38

6697000.000 2.92675 76970000.000 5.61 3.04 2.81 5.83 5.38

6704000.000 2.92690 77040000.000 5.61 3.04 2.81 5.83 5.38

6711000.000 2.92705 77110000.000 5.61 3.04 2.81 5.83 5.38

6718000.000 2.92721 77180000.000 5.61 3.04 2.81 5.83 5.38

6725000.000 2.92736 77250000.000 5.61 3.04 2.81 5.83 5.38

6732000.000 2.92751 77320000.000 5.61 3.04 2.81 5.83 5.39

6739000.000 2.92767 77390000.000 5.61 3.04 2.81 5.83 5.39

6746000.000 2.92782 77460000.000 5.61 3.04 2.81 5.83 5.39

6753000.000 2.92797 77530000.000 5.61 3.05 2.81 5.83 5.39

6760000.000 2.92812 77600000.000 5.61 3.05 2.81 5.83 5.39

6767000.000 2.92827 77670000.000 5.61 3.05 2.81 5.84 5.39

6774000.000 2.92843 77740000.000 5.61 3.05 2.81 5.84 5.39

6781000.000 2.92858 77810000.000 5.61 3.05 2.81 5.84 5.39

6788000.000 2.92873 77880000.000 5.61 3.05 2.81 5.84 5.39

6795000.000 2.92888 77950000.000 5.61 3.05 2.81 5.84 5.39

6802000.000 2.92903 78020000.000 5.61 3.05 2.81 5.84 5.39

6809000.000 2.92918 78090000.000 5.61 3.05 2.81 5.84 5.39

6816000.000 2.92933 78160000.000 5.61 3.05 2.81 5.84 5.39

6823000.000 2.92948 78230000.000 5.61 3.05 2.81 5.84 5.39

6830000.000 2.92964 78300000.000 5.61 3.05 2.81 5.84 5.39

6837000.000 2.92979 78370000.000 5.61 3.05 2.81 5.84 5.39

6844000.000 2.92994 78440000.000 5.61 3.05 2.81 5.84 5.39

6851000.000 2.93009 78510000.000 5.61 3.05 2.81 5.84 5.39

6858000.000 2.93024 78580000.000 5.61 3.05 2.81 5.84 5.39

6865000.000 2.93039 78650000.000 5.61 3.05 2.81 5.84 5.39

6872000.000 2.93054 78720000.000 5.61 3.05 2.81 5.84 5.39

6879000.000 2.93069 78790000.000 5.61 3.05 2.81 5.84 5.39

6886000.000 2.93084 78860000.000 5.62 3.05 2.81 5.84 5.39

6893000.000 2.93099 78930000.000 5.62 3.05 2.81 5.84 5.39

6900000.000 2.93113 79000000.000 5.62 3.05 2.81 5.84 5.39

6907000.000 2.93128 79070000.000 5.62 3.05 2.81 5.84 5.39

6914000.000 2.93143 79140000.000 5.62 3.05 2.81 5.84 5.39

6921000.000 2.93158 79210000.000 5.62 3.05 2.81 5.84 5.39

6928000.000 2.93173 79280000.000 5.62 3.05 2.81 5.84 5.39

6935000.000 2.93188 79350000.000 5.62 3.05 2.81 5.84 5.39

6942000.000 2.93203 79420000.000 5.62 3.05 2.81 5.84 5.39

6949000.000 2.93217 79490000.000 5.62 3.05 2.81 5.84 5.39

6956000.000 2.93232 79560000.000 5.62 3.05 2.82 5.84 5.39

6963000.000 2.93247 79630000.000 5.62 3.05 2.82 5.84 5.39

6970000.000 2.93262 79700000.000 5.62 3.05 2.82 5.84 5.39

6977000.000 2.93277 79770000.000 5.62 3.05 2.82 5.84 5.39

6984000.000 2.93291 79840000.000 5.62 3.05 2.82 5.84 5.39

6991000.000 2.93306 79910000.000 5.62 3.05 2.82 5.84 5.39

6998000.000 2.93321 79980000.000 5.62 3.05 2.82 5.84 5.39

7005000.000 2.93336 80050000.000 5.62 3.05 2.82 5.84 5.39

7012000.000 2.93350 80120000.000 5.62 3.05 2.82 5.84 5.39

7019000.000 2.93365 80190000.000 5.62 3.05 2.82 5.84 5.39

7026000.000 2.93380 80260000.000 5.62 3.05 2.82 5.84 5.40

7033000.000 2.93394 80330000.000 5.62 3.05 2.82 5.85 5.40

7040000.000 2.93409 80400000.000 5.62 3.05 2.82 5.85 5.40

7047000.000 2.93424 80470000.000 5.62 3.05 2.82 5.85 5.40

7054000.000 2.93438 80540000.000 5.62 3.05 2.82 5.85 5.40

7061000.000 2.93453 80610000.000 5.62 3.05 2.82 5.85 5.40

7068000.000 2.93467 80680000.000 5.62 3.05 2.82 5.85 5.40

7075000.000 2.93482 80750000.000 5.62 3.05 2.82 5.85 5.40

7082000.000 2.93496 80820000.000 5.62 3.05 2.82 5.85 5.40

7089000.000 2.93511 80890000.000 5.62 3.05 2.82 5.85 5.40

7096000.000 2.93526 80960000.000 5.62 3.05 2.82 5.85 5.40

7103000.000 2.93540 81030000.000 5.62 3.05 2.82 5.85 5.40

7110000.000 2.93555 81100000.000 5.62 3.05 2.82 5.85 5.40

7117000.000 2.93569 81170000.000 5.62 3.05 2.82 5.85 5.40

7124000.000 2.93584 81240000.000 5.62 3.05 2.82 5.85 5.40

7131000.000 2.93598 81310000.000 5.62 3.05 2.82 5.85 5.40

7138000.000 2.93612 81380000.000 5.62 3.05 2.82 5.85 5.40

7145000.000 2.93627 81450000.000 5.62 3.05 2.82 5.85 5.40

7152000.000 2.93641 81520000.000 5.62 3.05 2.82 5.85 5.40

7159000.000 2.93656 81590000.000 5.62 3.05 2.82 5.85 5.40

7166000.000 2.93670 81660000.000 5.62 3.05 2.82 5.85 5.40

7173000.000 2.93684 81730000.000 5.63 3.05 2.82 5.85 5.40

7180000.000 2.93699 81800000.000 5.63 3.05 2.82 5.85 5.40

7187000.000 2.93713 81870000.000 5.63 3.05 2.82 5.85 5.40

7194000.000 2.93728 81940000.000 5.63 3.05 2.82 5.85 5.40

7201000.000 2.93742 82010000.000 5.63 3.05 2.82 5.85 5.40

7208000.000 2.93756 82080000.000 5.63 3.06 2.82 5.85 5.40

7215000.000 2.93771 82150000.000 5.63 3.06 2.82 5.85 5.40

7222000.000 2.93785 82220000.000 5.63 3.06 2.82 5.85 5.40

7229000.000 2.93799 82290000.000 5.63 3.06 2.82 5.85 5.40

7236000.000 2.93813 82360000.000 5.63 3.06 2.82 5.85 5.40

7243000.000 2.93828 82430000.000 5.63 3.06 2.82 5.85 5.40

7250000.000 2.93842 82500000.000 5.63 3.06 2.82 5.85 5.40

7257000.000 2.93856 82570000.000 5.63 3.06 2.82 5.85 5.40

7264000.000 2.93870 82640000.000 5.63 3.06 2.82 5.85 5.40

7271000.000 2.93884 82710000.000 5.63 3.06 2.82 5.85 5.40

7278000.000 2.93899 82780000.000 5.63 3.06 2.82 5.85 5.40

7285000.000 2.93913 82850000.000 5.63 3.06 2.82 5.85 5.40

7292000.000 2.93927 82920000.000 5.63 3.06 2.82 5.85 5.40

7299000.000 2.93941 82990000.000 5.63 3.06 2.82 5.85 5.40

7306000.000 2.93955 83060000.000 5.63 3.06 2.82 5.85 5.40

7313000.000 2.93969 83130000.000 5.63 3.06 2.82 5.86 5.40

7320000.000 2.93983 83200000.000 5.63 3.06 2.82 5.86 5.40

7327000.000 2.93998 83270000.000 5.63 3.06 2.82 5.86 5.41

7334000.000 2.94012 83340000.000 5.63 3.06 2.82 5.86 5.41

7341000.000 2.94026 83410000.000 5.63 3.06 2.82 5.86 5.41

7348000.000 2.94040 83480000.000 5.63 3.06 2.82 5.86 5.41

7355000.000 2.94054 83550000.000 5.63 3.06 2.82 5.86 5.41

7362000.000 2.94068 83620000.000 5.63 3.06 2.82 5.86 5.41

7369000.000 2.94082 83690000.000 5.63 3.06 2.82 5.86 5.41

7376000.000 2.94096 83760000.000 5.63 3.06 2.82 5.86 5.41

7383000.000 2.94110 83830000.000 5.63 3.06 2.82 5.86 5.41

7390000.000 2.94124 83900000.000 5.63 3.06 2.82 5.86 5.41

7397000.000 2.94138 83970000.000 5.63 3.06 2.82 5.86 5.41

7404000.000 2.94152 84040000.000 5.63 3.06 2.82 5.86 5.41

7411000.000 2.94166 84110000.000 5.63 3.06 2.82 5.86 5.41

7418000.000 2.94180 84180000.000 5.63 3.06 2.82 5.86 5.41

7425000.000 2.94194 84250000.000 5.63 3.06 2.82 5.86 5.41

7432000.000 2.94207 84320000.000 5.63 3.06 2.82 5.86 5.41

7439000.000 2.94221 84390000.000 5.63 3.06 2.82 5.86 5.41

7446000.000 2.94235 84460000.000 5.63 3.06 2.82 5.86 5.41

7453000.000 2.94249 84530000.000 5.63 3.06 2.82 5.86 5.41

7460000.000 2.94263 84600000.000 5.63 3.06 2.82 5.86 5.41

7467000.000 2.94277 84670000.000 5.64 3.06 2.83 5.86 5.41

7474000.000 2.94291 84740000.000 5.64 3.06 2.83 5.86 5.41

7481000.000 2.94304 84810000.000 5.64 3.06 2.83 5.86 5.41

7488000.000 2.94318 84880000.000 5.64 3.06 2.83 5.86 5.41

7495000.000 2.94332 84950000.000 5.64 3.06 2.83 5.86 5.41

7502000.000 2.94346 85020000.000 5.64 3.06 2.83 5.86 5.41

7509000.000 2.94360 85090000.000 5.64 3.06 2.83 5.86 5.41

7516000.000 2.94373 85160000.000 5.64 3.06 2.83 5.86 5.41

7523000.000 2.94387 85230000.000 5.64 3.06 2.83 5.86 5.41

7530000.000 2.94401 85300000.000 5.64 3.06 2.83 5.86 5.41

7537000.000 2.94415 85370000.000 5.64 3.06 2.83 5.86 5.41

7544000.000 2.94428 85440000.000 5.64 3.06 2.83 5.86 5.41

7551000.000 2.94442 85510000.000 5.64 3.06 2.83 5.86 5.41

7558000.000 2.94456 85580000.000 5.64 3.06 2.83 5.86 5.41

7565000.000 2.94469 85650000.000 5.64 3.06 2.83 5.86 5.41

7572000.000 2.94483 85720000.000 5.64 3.06 2.83 5.86 5.41

7579000.000 2.94497 85790000.000 5.64 3.06 2.83 5.86 5.41

7586000.000 2.94510 85860000.000 5.64 3.06 2.83 5.86 5.41

7593000.000 2.94524 85930000.000 5.64 3.06 2.83 5.86 5.41

7600000.000 2.94538 86000000.000 5.64 3.06 2.83 5.87 5.41

7607000.000 2.94551 86070000.000 5.64 3.06 2.83 5.87 5.41

7614000.000 2.94565 86140000.000 5.64 3.06 2.83 5.87 5.41

7621000.000 2.94578 86210000.000 5.64 3.06 2.83 5.87 5.41

7628000.000 2.94592 86280000.000 5.64 3.06 2.83 5.87 5.41

7635000.000 2.94605 86350000.000 5.64 3.06 2.83 5.87 5.42

7642000.000 2.94619 86420000.000 5.64 3.06 2.83 5.87 5.42

7649000.000 2.94633 86490000.000 5.64 3.06 2.83 5.87 5.42

7656000.000 2.94646 86560000.000 5.64 3.06 2.83 5.87 5.42

7663000.000 2.94660 86630000.000 5.64 3.06 2.83 5.87 5.42

7670000.000 2.94673 86700000.000 5.64 3.06 2.83 5.87 5.42

7677000.000 2.94687 86770000.000 5.64 3.06 2.83 5.87 5.42

7684000.000 2.94700 86840000.000 5.64 3.06 2.83 5.87 5.42

7691000.000 2.94713 86910000.000 5.64 3.07 2.83 5.87 5.42

7698000.000 2.94727 86980000.000 5.64 3.07 2.83 5.87 5.42

7705000.000 2.94740 87050000.000 5.64 3.07 2.83 5.87 5.42

7712000.000 2.94754 87120000.000 5.64 3.07 2.83 5.87 5.42

7719000.000 2.94767 87190000.000 5.64 3.07 2.83 5.87 5.42

7726000.000 2.94781 87260000.000 5.64 3.07 2.83 5.87 5.42

7733000.000 2.94794 87330000.000 5.64 3.07 2.83 5.87 5.42

7740000.000 2.94807 87400000.000 5.64 3.07 2.83 5.87 5.42

7747000.000 2.94821 87470000.000 5.64 3.07 2.83 5.87 5.42

7754000.000 2.94834 87540000.000 5.64 3.07 2.83 5.87 5.42

7761000.000 2.94847 87610000.000 5.64 3.07 2.83 5.87 5.42

7768000.000 2.94861 87680000.000 5.64 3.07 2.83 5.87 5.42

7775000.000 2.94874 87750000.000 5.65 3.07 2.83 5.87 5.42

7782000.000 2.94887 87820000.000 5.65 3.07 2.83 5.87 5.42

7789000.000 2.94901 87890000.000 5.65 3.07 2.83 5.87 5.42

7796000.000 2.94914 87960000.000 5.65 3.07 2.83 5.87 5.42

7803000.000 2.94927 88030000.000 5.65 3.07 2.83 5.87 5.42

7810000.000 2.94941 88100000.000 5.65 3.07 2.83 5.87 5.42

7817000.000 2.94954 88170000.000 5.65 3.07 2.83 5.87 5.42

7824000.000 2.94967 88240000.000 5.65 3.07 2.83 5.87 5.42

7831000.000 2.94980 88310000.000 5.65 3.07 2.83 5.87 5.42

7838000.000 2.94993 88380000.000 5.65 3.07 2.83 5.87 5.42

7845000.000 2.95007 88450000.000 5.65 3.07 2.83 5.87 5.42

7852000.000 2.95020 88520000.000 5.65 3.07 2.83 5.87 5.42

7859000.000 2.95033 88590000.000 5.65 3.07 2.83 5.87 5.42

7866000.000 2.95046 88660000.000 5.65 3.07 2.83 5.87 5.42

7873000.000 2.95059 88730000.000 5.65 3.07 2.83 5.87 5.42

7880000.000 2.95073 88800000.000 5.65 3.07 2.83 5.87 5.42

7887000.000 2.95086 88870000.000 5.65 3.07 2.83 5.87 5.42

7894000.000 2.95099 88940000.000 5.65 3.07 2.83 5.88 5.42

7901000.000 2.95112 89010000.000 5.65 3.07 2.83 5.88 5.42

7908000.000 2.95125 89080000.000 5.65 3.07 2.83 5.88 5.42

7915000.000 2.95138 89150000.000 5.65 3.07 2.83 5.88 5.42

7922000.000 2.95151 89220000.000 5.65 3.07 2.83 5.88 5.42

7929000.000 2.95164 89290000.000 5.65 3.07 2.83 5.88 5.42

7936000.000 2.95177 89360000.000 5.65 3.07 2.83 5.88 5.42

7943000.000 2.95190 89430000.000 5.65 3.07 2.83 5.88 5.42

7950000.000 2.95203 89500000.000 5.65 3.07 2.83 5.88 5.42

7957000.000 2.95217 89570000.000 5.65 3.07 2.83 5.88 5.42

7964000.000 2.95230 89640000.000 5.65 3.07 2.83 5.88 5.43

7971000.000 2.95243 89710000.000 5.65 3.07 2.83 5.88 5.43

7978000.000 2.95256 89780000.000 5.65 3.07 2.83 5.88 5.43

7985000.000 2.95269 89850000.000 5.65 3.07 2.83 5.88 5.43

7992000.000 2.95282 89920000.000 5.65 3.07 2.83 5.88 5.43

7999000.000 2.95294 89990000.000 5.65 3.07 2.83 5.88 5.43

8006000.000 2.95307 90060000.000 5.65 3.07 2.83 5.88 5.43

8013000.000 2.95320 90130000.000 5.65 3.07 2.84 5.88 5.43

8020000.000 2.95333 90200000.000 5.65 3.07 2.84 5.88 5.43

8027000.000 2.95346 90270000.000 5.65 3.07 2.84 5.88 5.43

8034000.000 2.95359 90340000.000 5.65 3.07 2.84 5.88 5.43

8041000.000 2.95372 90410000.000 5.65 3.07 2.84 5.88 5.43

8048000.000 2.95385 90480000.000 5.65 3.07 2.84 5.88 5.43

8055000.000 2.95398 90550000.000 5.65 3.07 2.84 5.88 5.43

8062000.000 2.95411 90620000.000 5.65 3.07 2.84 5.88 5.43

8069000.000 2.95424 90690000.000 5.65 3.07 2.84 5.88 5.43

8076000.000 2.95436 90760000.000 5.65 3.07 2.84 5.88 5.43

8083000.000 2.95449 90830000.000 5.66 3.07 2.84 5.88 5.43

8090000.000 2.95462 90900000.000 5.66 3.07 2.84 5.88 5.43

8097000.000 2.95475 90970000.000 5.66 3.07 2.84 5.88 5.43

8104000.000 2.95488 91040000.000 5.66 3.07 2.84 5.88 5.43

8111000.000 2.95501 91110000.000 5.66 3.07 2.84 5.88 5.43

8118000.000 2.95513 91180000.000 5.66 3.07 2.84 5.88 5.43

8125000.000 2.95526 91250000.000 5.66 3.07 2.84 5.88 5.43

8132000.000 2.95539 91320000.000 5.66 3.07 2.84 5.88 5.43

8139000.000 2.95552 91390000.000 5.66 3.07 2.84 5.88 5.43

8146000.000 2.95564 91460000.000 5.66 3.07 2.84 5.88 5.43

8153000.000 2.95577 91530000.000 5.66 3.07 2.84 5.88 5.43

8160000.000 2.95590 91600000.000 5.66 3.07 2.84 5.88 5.43

8167000.000 2.95603 91670000.000 5.66 3.07 2.84 5.88 5.43

8174000.000 2.95615 91740000.000 5.66 3.07 2.84 5.88 5.43

8181000.000 2.95628 91810000.000 5.66 3.07 2.84 5.88 5.43

8188000.000 2.95641 91880000.000 5.66 3.07 2.84 5.88 5.43

8195000.000 2.95653 91950000.000 5.66 3.07 2.84 5.88 5.43

8202000.000 2.95666 92020000.000 5.66 3.07 2.84 5.89 5.43

8209000.000 2.95679 92090000.000 5.66 3.08 2.84 5.89 5.43

8216000.000 2.95691 92160000.000 5.66 3.08 2.84 5.89 5.43

8223000.000 2.95704 92230000.000 5.66 3.08 2.84 5.89 5.43

8230000.000 2.95716 92300000.000 5.66 3.08 2.84 5.89 5.43

8237000.000 2.95729 92370000.000 5.66 3.08 2.84 5.89 5.43

8244000.000 2.95742 92440000.000 5.66 3.08 2.84 5.89 5.43

8251000.000 2.95754 92510000.000 5.66 3.08 2.84 5.89 5.43

8258000.000 2.95767 92580000.000 5.66 3.08 2.84 5.89 5.43

8265000.000 2.95779 92650000.000 5.66 3.08 2.84 5.89 5.43

8272000.000 2.95792 92720000.000 5.66 3.08 2.84 5.89 5.43

8279000.000 2.95805 92790000.000 5.66 3.08 2.84 5.89 5.43

8286000.000 2.95817 92860000.000 5.66 3.08 2.84 5.89 5.43

8293000.000 2.95830 92930000.000 5.66 3.08 2.84 5.89 5.44

8300000.000 2.95842 93000000.000 5.66 3.08 2.84 5.89 5.44

8307000.000 2.95855 93070000.000 5.66 3.08 2.84 5.89 5.44

8314000.000 2.95867 93140000.000 5.66 3.08 2.84 5.89 5.44

8321000.000 2.95880 93210000.000 5.66 3.08 2.84 5.89 5.44

8328000.000 2.95892 93280000.000 5.66 3.08 2.84 5.89 5.44

8335000.000 2.95905 93350000.000 5.66 3.08 2.84 5.89 5.44

8342000.000 2.95917 93420000.000 5.66 3.08 2.84 5.89 5.44

8349000.000 2.95929 93490000.000 5.66 3.08 2.84 5.89 5.44

8356000.000 2.95942 93560000.000 5.66 3.08 2.84 5.89 5.44

8363000.000 2.95954 93630000.000 5.66 3.08 2.84 5.89 5.44

8370000.000 2.95967 93700000.000 5.66 3.08 2.84 5.89 5.44

8377000.000 2.95979 93770000.000 5.66 3.08 2.84 5.89 5.44

8384000.000 2.95992 93840000.000 5.66 3.08 2.84 5.89 5.44

8391000.000 2.96004 93910000.000 5.66 3.08 2.84 5.89 5.44

8398000.000 2.96016 93980000.000 5.66 3.08 2.84 5.89 5.44

8405000.000 2.96029 94050000.000 5.66 3.08 2.84 5.89 5.44

8412000.000 2.96041 94120000.000 5.67 3.08 2.84 5.89 5.44

8419000.000 2.96053 94190000.000 5.67 3.08 2.84 5.89 5.44

8426000.000 2.96066 94260000.000 5.67 3.08 2.84 5.89 5.44

8433000.000 2.96078 94330000.000 5.67 3.08 2.84 5.89 5.44

8440000.000 2.96090 94400000.000 5.67 3.08 2.84 5.89 5.44

8447000.000 2.96103 94470000.000 5.67 3.08 2.84 5.89 5.44

8454000.000 2.96115 94540000.000 5.67 3.08 2.84 5.89 5.44

8461000.000 2.96127 94610000.000 5.67 3.08 2.84 5.89 5.44

8468000.000 2.96140 94680000.000 5.67 3.08 2.84 5.89 5.44

8475000.000 2.96152 94750000.000 5.67 3.08 2.84 5.89 5.44

8482000.000 2.96164 94820000.000 5.67 3.08 2.84 5.89 5.44

8489000.000 2.96176 94890000.000 5.67 3.08 2.84 5.89 5.44

8496000.000 2.96189 94960000.000 5.67 3.08 2.84 5.89 5.44

8503000.000 2.96201 95030000.000 5.67 3.08 2.84 5.89 5.44

8510000.000 2.96213 95100000.000 5.67 3.08 2.84 5.89 5.44

8517000.000 2.96225 95170000.000 5.67 3.08 2.84 5.89 5.44

8524000.000 2.96238 95240000.000 5.67 3.08 2.84 5.90 5.44

8531000.000 2.96250 95310000.000 5.67 3.08 2.84 5.90 5.44

8538000.000 2.96262 95380000.000 5.67 3.08 2.84 5.90 5.44

8545000.000 2.96274 95450000.000 5.67 3.08 2.84 5.90 5.44

8552000.000 2.96286 95520000.000 5.67 3.08 2.84 5.90 5.44

8559000.000 2.96298 95590000.000 5.67 3.08 2.84 5.90 5.44

8566000.000 2.96311 95660000.000 5.67 3.08 2.84 5.90 5.44

8573000.000 2.96323 95730000.000 5.67 3.08 2.84 5.90 5.44

8580000.000 2.96335 95800000.000 5.67 3.08 2.84 5.90 5.44

8587000.000 2.96347 95870000.000 5.67 3.08 2.84 5.90 5.44

8594000.000 2.96359 95940000.000 5.67 3.08 2.85 5.90 5.44

8601000.000 2.96371 96010000.000 5.67 3.08 2.85 5.90 5.44

8608000.000 2.96383 96080000.000 5.67 3.08 2.85 5.90 5.44

8615000.000 2.96395 96150000.000 5.67 3.08 2.85 5.90 5.44

8622000.000 2.96407 96220000.000 5.67 3.08 2.85 5.90 5.44

8629000.000 2.96420 96290000.000 5.67 3.08 2.85 5.90 5.44

8636000.000 2.96432 96360000.000 5.67 3.08 2.85 5.90 5.44

8643000.000 2.96444 96430000.000 5.67 3.08 2.85 5.90 5.45

8650000.000 2.96456 96500000.000 5.67 3.08 2.85 5.90 5.45

8657000.000 2.96468 96570000.000 5.67 3.08 2.85 5.90 5.45

8664000.000 2.96480 96640000.000 5.67 3.08 2.85 5.90 5.45

8671000.000 2.96492 96710000.000 5.67 3.08 2.85 5.90 5.45

8678000.000 2.96504 96780000.000 5.67 3.08 2.85 5.90 5.45

8685000.000 2.96516 96850000.000 5.67 3.08 2.85 5.90 5.45

8692000.000 2.96528 96920000.000 5.67 3.08 2.85 5.90 5.45

8699000.000 2.96540 96990000.000 5.67 3.08 2.85 5.90 5.45

8706000.000 2.96552 97060000.000 5.67 3.08 2.85 5.90 5.45

8713000.000 2.96564 97130000.000 5.67 3.08 2.85 5.90 5.45

8720000.000 2.96576 97200000.000 5.67 3.08 2.85 5.90 5.45

8727000.000 2.96587 97270000.000 5.67 3.08 2.85 5.90 5.45

8734000.000 2.96599 97340000.000 5.67 3.08 2.85 5.90 5.45

8741000.000 2.96611 97410000.000 5.67 3.08 2.85 5.90 5.45

8748000.000 2.96623 97480000.000 5.68 3.08 2.85 5.90 5.45

8755000.000 2.96635 97550000.000 5.68 3.09 2.85 5.90 5.45

8762000.000 2.96647 97620000.000 5.68 3.09 2.85 5.90 5.45

8769000.000 2.96659 97690000.000 5.68 3.09 2.85 5.90 5.45

8776000.000 2.96671 97760000.000 5.68 3.09 2.85 5.90 5.45

8783000.000 2.96683 97830000.000 5.68 3.09 2.85 5.90 5.45

8790000.000 2.96695 97900000.000 5.68 3.09 2.85 5.90 5.45

8797000.000 2.96706 97970000.000 5.68 3.09 2.85 5.90 5.45

8804000.000 2.96718 98040000.000 5.68 3.09 2.85 5.90 5.45

8811000.000 2.96730 98110000.000 5.68 3.09 2.85 5.90 5.45

8818000.000 2.96742 98180000.000 5.68 3.09 2.85 5.90 5.45

8825000.000 2.96754 98250000.000 5.68 3.09 2.85 5.90 5.45

8832000.000 2.96765 98320000.000 5.68 3.09 2.85 5.90 5.45

8839000.000 2.96777 98390000.000 5.68 3.09 2.85 5.90 5.45

8846000.000 2.96789 98460000.000 5.68 3.09 2.85 5.91 5.45

8853000.000 2.96801 98530000.000 5.68 3.09 2.85 5.91 5.45

8860000.000 2.96813 98600000.000 5.68 3.09 2.85 5.91 5.45

8867000.000 2.96824 98670000.000 5.68 3.09 2.85 5.91 5.45

8874000.000 2.96836 98740000.000 5.68 3.09 2.85 5.91 5.45

8881000.000 2.96848 98810000.000 5.68 3.09 2.85 5.91 5.45

8888000.000 2.96860 98880000.000 5.68 3.09 2.85 5.91 5.45

8895000.000 2.96871 98950000.000 5.68 3.09 2.85 5.91 5.45

8902000.000 2.96883 99020000.000 5.68 3.09 2.85 5.91 5.45

8909000.000 2.96895 99090000.000 5.68 3.09 2.85 5.91 5.45

8916000.000 2.96906 99160000.000 5.68 3.09 2.85 5.91 5.45

8923000.000 2.96918 99230000.000 5.68 3.09 2.85 5.91 5.45

8930000.000 2.96930 99300000.000 5.68 3.09 2.85 5.91 5.45

8937000.000 2.96941 99370000.000 5.68 3.09 2.85 5.91 5.45

8944000.000 2.96953 99440000.000 5.68 3.09 2.85 5.91 5.45

8951000.000 2.96965 99510000.000 5.68 3.09 2.85 5.91 5.45

8958000.000 2.96976 99580000.000 5.68 3.09 2.85 5.91 5.45

8965000.000 2.96988 99650000.000 5.68 3.09 2.85 5.91 5.45

8972000.000 2.97000 99720000.000 5.68 3.09 2.85 5.91 5.45

8979000.000 2.97011 99790000.000 5.68 3.09 2.85 5.91 5.45

8986000.000 2.97023 99860000.000 5.68 3.09 2.85 5.91 5.45

8993000.000 2.97035 99930000.000 5.68 3.09 2.85 5.91 5.45

9000000.000 2.97046 100000000.000 5.68 3.09 2.85 5.91 5.46

9007000.000 2.97058 100070000.000 5.68 3.09 2.85 5.91 5.46

9014000.000 2.97069 100140000.000 5.68 3.09 2.85 5.91 5.46

9021000.000 2.97081 100210000.000 5.68 3.09 2.85 5.91 5.46

9028000.000 2.97092 100280000.000 5.68 3.09 2.85 5.91 5.46

9035000.000 2.97104 100350000.000 5.68 3.09 2.85 5.91 5.46

9042000.000 2.97115 100420000.000 5.68 3.09 2.85 5.91 5.46

9049000.000 2.97127 100490000.000 5.68 3.09 2.85 5.91 5.46

9056000.000 2.97139 100560000.000 5.68 3.09 2.85 5.91 5.46

9063000.000 2.97150 100630000.000 5.68 3.09 2.85 5.91 5.46

9070000.000 2.97162 100700000.000 5.68 3.09 2.85 5.91 5.46

9077000.000 2.97173 100770000.000 5.68 3.09 2.85 5.91 5.46

9084000.000 2.97185 100840000.000 5.68 3.09 2.85 5.91 5.46

9091000.000 2.97196 100910000.000 5.68 3.09 2.85 5.91 5.46

9098000.000 2.97208 100980000.000 5.69 3.09 2.85 5.91 5.46

9105000.000 2.97219 101050000.000 5.69 3.09 2.85 5.91 5.46

9112000.000 2.97230 101120000.000 5.69 3.09 2.85 5.91 5.46

9119000.000 2.97242 101190000.000 5.69 3.09 2.85 5.91 5.46

9126000.000 2.97253 101260000.000 5.69 3.09 2.85 5.91 5.46

9133000.000 2.97265 101330000.000 5.69 3.09 2.85 5.91 5.46

9140000.000 2.97276 101400000.000 5.69 3.09 2.85 5.91 5.46

9147000.000 2.97288 101470000.000 5.69 3.09 2.85 5.91 5.46

9154000.000 2.97299 101540000.000 5.69 3.09 2.85 5.91 5.46

9161000.000 2.97310 101610000.000 5.69 3.09 2.85 5.91 5.46

9168000.000 2.97322 101680000.000 5.69 3.09 2.85 5.91 5.46

9175000.000 2.97333 101750000.000 5.69 3.09 2.85 5.91 5.46

9182000.000 2.97345 101820000.000 5.69 3.09 2.85 5.91 5.46

9189000.000 2.97356 101890000.000 5.69 3.09 2.85 5.92 5.46

9196000.000 2.97367 101960000.000 5.69 3.09 2.85 5.92 5.46

9203000.000 2.97379 102030000.000 5.69 3.09 2.85 5.92 5.46

9210000.000 2.97390 102100000.000 5.69 3.09 2.85 5.92 5.46

9217000.000 2.97401 102170000.000 5.69 3.09 2.86 5.92 5.46

9224000.000 2.97413 102240000.000 5.69 3.09 2.86 5.92 5.46

9231000.000 2.97424 102310000.000 5.69 3.09 2.86 5.92 5.46

9238000.000 2.97435 102380000.000 5.69 3.09 2.86 5.92 5.46

9245000.000 2.97447 102450000.000 5.69 3.09 2.86 5.92 5.46

9252000.000 2.97458 102520000.000 5.69 3.09 2.86 5.92 5.46

9259000.000 2.97469 102590000.000 5.69 3.09 2.86 5.92 5.46

9266000.000 2.97480 102660000.000 5.69 3.09 2.86 5.92 5.46

9273000.000 2.97492 102730000.000 5.69 3.09 2.86 5.92 5.46

9280000.000 2.97503 102800000.000 5.69 3.09 2.86 5.92 5.46

9287000.000 2.97514 102870000.000 5.69 3.09 2.86 5.92 5.46

9294000.000 2.97525 102940000.000 5.69 3.09 2.86 5.92 5.46

9301000.000 2.97537 103010000.000 5.69 3.09 2.86 5.92 5.46

9308000.000 2.97548 103080000.000 5.69 3.09 2.86 5.92 5.46

9315000.000 2.97559 103150000.000 5.69 3.09 2.86 5.92 5.46

9322000.000 2.97570 103220000.000 5.69 3.09 2.86 5.92 5.46

9329000.000 2.97582 103290000.000 5.69 3.09 2.86 5.92 5.46

9336000.000 2.97593 103360000.000 5.69 3.09 2.86 5.92 5.46

9343000.000 2.97604 103430000.000 5.69 3.10 2.86 5.92 5.46

9350000.000 2.97615 103500000.000 5.69 3.10 2.86 5.92 5.46

9357000.000 2.97626 103570000.000 5.69 3.10 2.86 5.92 5.46

9364000.000 2.97637 103640000.000 5.69 3.10 2.86 5.92 5.46

9371000.000 2.97649 103710000.000 5.69 3.10 2.86 5.92 5.47

9378000.000 2.97660 103780000.000 5.69 3.10 2.86 5.92 5.47

9385000.000 2.97671 103850000.000 5.69 3.10 2.86 5.92 5.47

9392000.000 2.97682 103920000.000 5.69 3.10 2.86 5.92 5.47

9399000.000 2.97693 103990000.000 5.69 3.10 2.86 5.92 5.47

9406000.000 2.97704 104060000.000 5.69 3.10 2.86 5.92 5.47

9413000.000 2.97715 104130000.000 5.69 3.10 2.86 5.92 5.47

9420000.000 2.97726 104200000.000 5.69 3.10 2.86 5.92 5.47

9427000.000 2.97738 104270000.000 5.69 3.10 2.86 5.92 5.47

9434000.000 2.97749 104340000.000 5.69 3.10 2.86 5.92 5.47

9441000.000 2.97760 104410000.000 5.69 3.10 2.86 5.92 5.47

9448000.000 2.97771 104480000.000 5.69 3.10 2.86 5.92 5.47

9455000.000 2.97782 104550000.000 5.70 3.10 2.86 5.92 5.47

9462000.000 2.97793 104620000.000 5.70 3.10 2.86 5.92 5.47

9469000.000 2.97804 104690000.000 5.70 3.10 2.86 5.92 5.47

9476000.000 2.97815 104760000.000 5.70 3.10 2.86 5.92 5.47

9483000.000 2.97826 104830000.000 5.70 3.10 2.86 5.92 5.47

9490000.000 2.97837 104900000.000 5.70 3.10 2.86 5.92 5.47

9497000.000 2.97848 104970000.000 5.70 3.10 2.86 5.92 5.47

9504000.000 2.97859 105040000.000 5.70 3.10 2.86 5.92 5.47

9511000.000 2.97870 105110000.000 5.70 3.10 2.86 5.92 5.47

9518000.000 2.97881 105180000.000 5.70 3.10 2.86 5.92 5.47

9525000.000 2.97892 105250000.000 5.70 3.10 2.86 5.92 5.47

9532000.000 2.97903 105320000.000 5.70 3.10 2.86 5.93 5.47

9539000.000 2.97914 105390000.000 5.70 3.10 2.86 5.93 5.47

9546000.000 2.97925 105460000.000 5.70 3.10 2.86 5.93 5.47

9553000.000 2.97936 105530000.000 5.70 3.10 2.86 5.93 5.47

9560000.000 2.97947 105600000.000 5.70 3.10 2.86 5.93 5.47

9567000.000 2.97958 105670000.000 5.70 3.10 2.86 5.93 5.47

9574000.000 2.97969 105740000.000 5.70 3.10 2.86 5.93 5.47

9581000.000 2.97980 105810000.000 5.70 3.10 2.86 5.93 5.47

9588000.000 2.97991 105880000.000 5.70 3.10 2.86 5.93 5.47

9595000.000 2.98001 105950000.000 5.70 3.10 2.86 5.93 5.47

9602000.000 2.98012 106020000.000 5.70 3.10 2.86 5.93 5.47

9609000.000 2.98023 106090000.000 5.70 3.10 2.86 5.93 5.47

9616000.000 2.98034 106160000.000 5.70 3.10 2.86 5.93 5.47

9623000.000 2.98045 106230000.000 5.70 3.10 2.86 5.93 5.47

9630000.000 2.98056 106300000.000 5.70 3.10 2.86 5.93 5.47

9637000.000 2.98067 106370000.000 5.70 3.10 2.86 5.93 5.47

9644000.000 2.98078 106440000.000 5.70 3.10 2.86 5.93 5.47

9651000.000 2.98088 106510000.000 5.70 3.10 2.86 5.93 5.47

9658000.000 2.98099 106580000.000 5.70 3.10 2.86 5.93 5.47

9665000.000 2.98110 106650000.000 5.70 3.10 2.86 5.93 5.47

9672000.000 2.98121 106720000.000 5.70 3.10 2.86 5.93 5.47

9679000.000 2.98132 106790000.000 5.70 3.10 2.86 5.93 5.47

9686000.000 2.98143 106860000.000 5.70 3.10 2.86 5.93 5.47

9693000.000 2.98153 106930000.000 5.70 3.10 2.86 5.93 5.47

9700000.000 2.98164 107000000.000 5.70 3.10 2.86 5.93 5.47

9707000.000 2.98175 107070000.000 5.70 3.10 2.86 5.93 5.47

9714000.000 2.98186 107140000.000 5.70 3.10 2.86 5.93 5.47

9721000.000 2.98197 107210000.000 5.70 3.10 2.86 5.93 5.47

9728000.000 2.98207 107280000.000 5.70 3.10 2.86 5.93 5.47

9735000.000 2.98218 107350000.000 5.70 3.10 2.86 5.93 5.47

9742000.000 2.98229 107420000.000 5.70 3.10 2.86 5.93 5.47

9749000.000 2.98240 107490000.000 5.70 3.10 2.86 5.93 5.47

9756000.000 2.98250 107560000.000 5.70 3.10 2.86 5.93 5.48

9763000.000 2.98261 107630000.000 5.70 3.10 2.86 5.93 5.48

9770000.000 2.98272 107700000.000 5.70 3.10 2.86 5.93 5.48

9777000.000 2.98282 107770000.000 5.70 3.10 2.86 5.93 5.48

9784000.000 2.98293 107840000.000 5.70 3.10 2.86 5.93 5.48

9791000.000 2.98304 107910000.000 5.70 3.10 2.86 5.93 5.48

9798000.000 2.98315 107980000.000 5.70 3.10 2.86 5.93 5.48

9805000.000 2.98325 108050000.000 5.70 3.10 2.86 5.93 5.48

9812000.000 2.98336 108120000.000 5.70 3.10 2.86 5.93 5.48

9819000.000 2.98347 108190000.000 5.70 3.10 2.86 5.93 5.48

9826000.000 2.98357 108260000.000 5.71 3.10 2.86 5.93 5.48

9833000.000 2.98368 108330000.000 5.71 3.10 2.86 5.93 5.48

9840000.000 2.98379 108400000.000 5.71 3.10 2.86 5.93 5.48

9847000.000 2.98389 108470000.000 5.71 3.10 2.86 5.93 5.48

9854000.000 2.98400 108540000.000 5.71 3.10 2.86 5.93 5.48

9861000.000 2.98411 108610000.000 5.71 3.10 2.86 5.93 5.48

9868000.000 2.98421 108680000.000 5.71 3.10 2.86 5.93 5.48

9875000.000 2.98432 108750000.000 5.71 3.10 2.86 5.93 5.48

9882000.000 2.98442 108820000.000 5.71 3.10 2.87 5.93 5.48

9889000.000 2.98453 108890000.000 5.71 3.10 2.87 5.93 5.48

9896000.000 2.98464 108960000.000 5.71 3.10 2.87 5.94 5.48

9903000.000 2.98474 109030000.000 5.71 3.10 2.87 5.94 5.48

9910000.000 2.98485 109100000.000 5.71 3.10 2.87 5.94 5.48

9917000.000 2.98495 109170000.000 5.71 3.10 2.87 5.94 5.48

9924000.000 2.98506 109240000.000 5.71 3.10 2.87 5.94 5.48

9931000.000 2.98516 109310000.000 5.71 3.10 2.87 5.94 5.48

9938000.000 2.98527 109380000.000 5.71 3.10 2.87 5.94 5.48

9945000.000 2.98538 109450000.000 5.71 3.10 2.87 5.94 5.48

9952000.000 2.98548 109520000.000 5.71 3.10 2.87 5.94 5.48

9959000.000 2.98559 109590000.000 5.71 3.11 2.87 5.94 5.48

9966000.000 2.98569 109660000.000 5.71 3.11 2.87 5.94 5.48

9973000.000 2.98580 109730000.000 5.71 3.11 2.87 5.94 5.48

9980000.000 2.98590 109800000.000 5.71 3.11 2.87 5.94 5.48

9987000.000 2.98601 109870000.000 5.71 3.11 2.87 5.94 5.48

9994000.000 2.98611 109940000.000 5.71 3.11 2.87 5.94 5.48

10001000.000 2.98622 110010000.000 5.71 3.11 2.87 5.94 5.48

10008000.000 2.98632 110080000.000 5.71 3.11 2.87 5.94 5.48

10015000.000 2.98643 110150000.000 5.71 3.11 2.87 5.94 5.48

10022000.000 2.98653 110220000.000 5.71 3.11 2.87 5.94 5.48

10029000.000 2.98663 110290000.000 5.71 3.11 2.87 5.94 5.48

10036000.000 2.98674 110360000.000 5.71 3.11 2.87 5.94 5.48

10043000.000 2.98684 110430000.000 5.71 3.11 2.87 5.94 5.48

10050000.000 2.98695 110500000.000 5.71 3.11 2.87 5.94 5.48

10057000.000 2.98705 110570000.000 5.71 3.11 2.87 5.94 5.48

10064000.000 2.98716 110640000.000 5.71 3.11 2.87 5.94 5.48

10071000.000 2.98726 110710000.000 5.71 3.11 2.87 5.94 5.48

10078000.000 2.98737 110780000.000 5.71 3.11 2.87 5.94 5.48

10085000.000 2.98747 110850000.000 5.71 3.11 2.87 5.94 5.48

10092000.000 2.98757 110920000.000 5.71 3.11 2.87 5.94 5.48

10099000.000 2.98768 110990000.000 5.71 3.11 2.87 5.94 5.48

10106000.000 2.98778 111060000.000 5.71 3.11 2.87 5.94 5.48

10113000.000 2.98788 111130000.000 5.71 3.11 2.87 5.94 5.48

10120000.000 2.98799 111200000.000 5.71 3.11 2.87 5.94 5.48

10127000.000 2.98809 111270000.000 5.71 3.11 2.87 5.94 5.48

10134000.000 2.98820 111340000.000 5.71 3.11 2.87 5.94 5.48

10141000.000 2.98830 111410000.000 5.71 3.11 2.87 5.94 5.48

10148000.000 2.98840 111480000.000 5.71 3.11 2.87 5.94 5.48

10155000.000 2.98851 111550000.000 5.71 3.11 2.87 5.94 5.49

10162000.000 2.98861 111620000.000 5.71 3.11 2.87 5.94 5.49

10169000.000 2.98871 111690000.000 5.71 3.11 2.87 5.94 5.49

10176000.000 2.98882 111760000.000 5.71 3.11 2.87 5.94 5.49

10183000.000 2.98892 111830000.000 5.71 3.11 2.87 5.94 5.49

10190000.000 2.98902 111900000.000 5.71 3.11 2.87 5.94 5.49

10197000.000 2.98912 111970000.000 5.71 3.11 2.87 5.94 5.49

10204000.000 2.98923 112040000.000 5.71 3.11 2.87 5.94 5.49

10211000.000 2.98933 112110000.000 5.72 3.11 2.87 5.94 5.49

10218000.000 2.98943 112180000.000 5.72 3.11 2.87 5.94 5.49

10225000.000 2.98954 112250000.000 5.72 3.11 2.87 5.94 5.49

10232000.000 2.98964 112320000.000 5.72 3.11 2.87 5.94 5.49

10239000.000 2.98974 112390000.000 5.72 3.11 2.87 5.94 5.49

10246000.000 2.98984 112460000.000 5.72 3.11 2.87 5.94 5.49

10253000.000 2.98995 112530000.000 5.72 3.11 2.87 5.94 5.49

10260000.000 2.99005 112600000.000 5.72 3.11 2.87 5.94 5.49

10267000.000 2.99015 112670000.000 5.72 3.11 2.87 5.95 5.49

10274000.000 2.99025 112740000.000 5.72 3.11 2.87 5.95 5.49

10281000.000 2.99035 112810000.000 5.72 3.11 2.87 5.95 5.49

10288000.000 2.99046 112880000.000 5.72 3.11 2.87 5.95 5.49

10295000.000 2.99056 112950000.000 5.72 3.11 2.87 5.95 5.49

10302000.000 2.99066 113020000.000 5.72 3.11 2.87 5.95 5.49

10309000.000 2.99076 113090000.000 5.72 3.11 2.87 5.95 5.49

10316000.000 2.99086 113160000.000 5.72 3.11 2.87 5.95 5.49

10323000.000 2.99097 113230000.000 5.72 3.11 2.87 5.95 5.49

10330000.000 2.99107 113300000.000 5.72 3.11 2.87 5.95 5.49

10337000.000 2.99117 113370000.000 5.72 3.11 2.87 5.95 5.49

10344000.000 2.99127 113440000.000 5.72 3.11 2.87 5.95 5.49

10351000.000 2.99137 113510000.000 5.72 3.11 2.87 5.95 5.49

10358000.000 2.99147 113580000.000 5.72 3.11 2.87 5.95 5.49

10365000.000 2.99158 113650000.000 5.72 3.11 2.87 5.95 5.49

10372000.000 2.99168 113720000.000 5.72 3.11 2.87 5.95 5.49

10379000.000 2.99178 113790000.000 5.72 3.11 2.87 5.95 5.49

10386000.000 2.99188 113860000.000 5.72 3.11 2.87 5.95 5.49

10393000.000 2.99198 113930000.000 5.72 3.11 2.87 5.95 5.49

10400000.000 2.99208 114000000.000 5.72 3.11 2.87 5.95 5.49

10407000.000 2.99218 114070000.000 5.72 3.11 2.87 5.95 5.49

10414000.000 2.99228 114140000.000 5.72 3.11 2.87 5.95 5.49

10421000.000 2.99238 114210000.000 5.72 3.11 2.87 5.95 5.49

10428000.000 2.99249 114280000.000 5.72 3.11 2.87 5.95 5.49

10435000.000 2.99259 114350000.000 5.72 3.11 2.87 5.95 5.49

10442000.000 2.99269 114420000.000 5.72 3.11 2.87 5.95 5.49

10449000.000 2.99279 114490000.000 5.72 3.11 2.87 5.95 5.49

10456000.000 2.99289 114560000.000 5.72 3.11 2.87 5.95 5.49

10463000.000 2.99299 114630000.000 5.72 3.11 2.87 5.95 5.49

10470000.000 2.99309 114700000.000 5.72 3.11 2.87 5.95 5.49

10477000.000 2.99319 114770000.000 5.72 3.11 2.87 5.95 5.49

10484000.000 2.99329 114840000.000 5.72 3.11 2.87 5.95 5.49

10491000.000 2.99339 114910000.000 5.72 3.11 2.87 5.95 5.49

10498000.000 2.99349 114980000.000 5.72 3.11 2.87 5.95 5.49

10505000.000 2.99359 115050000.000 5.72 3.11 2.87 5.95 5.49

10512000.000 2.99369 115120000.000 5.72 3.11 2.87 5.95 5.49

10519000.000 2.99379 115190000.000 5.72 3.11 2.87 5.95 5.49

10526000.000 2.99389 115260000.000 5.72 3.11 2.87 5.95 5.49

10533000.000 2.99399 115330000.000 5.72 3.11 2.87 5.95 5.49

10540000.000 2.99409 115400000.000 5.72 3.11 2.87 5.95 5.49

10547000.000 2.99419 115470000.000 5.72 3.11 2.87 5.95 5.49

10554000.000 2.99429 115540000.000 5.72 3.11 2.87 5.95 5.49

10561000.000 2.99439 115610000.000 5.72 3.11 2.87 5.95 5.49

10568000.000 2.99449 115680000.000 5.72 3.11 2.87 5.95 5.50

10575000.000 2.99459 115750000.000 5.72 3.11 2.87 5.95 5.50

10582000.000 2.99469 115820000.000 5.72 3.11 2.87 5.95 5.50

10589000.000 2.99479 115890000.000 5.72 3.11 2.87 5.95 5.50

10596000.000 2.99489 115960000.000 5.72 3.11 2.88 5.95 5.50

10603000.000 2.99498 116030000.000 5.72 3.11 2.88 5.95 5.50

10610000.000 2.99508 116100000.000 5.73 3.11 2.88 5.95 5.50

10617000.000 2.99518 116170000.000 5.73 3.11 2.88 5.95 5.50

10624000.000 2.99528 116240000.000 5.73 3.12 2.88 5.95 5.50

10631000.000 2.99538 116310000.000 5.73 3.12 2.88 5.95 5.50

10638000.000 2.99548 116380000.000 5.73 3.12 2.88 5.95 5.50

10645000.000 2.99558 116450000.000 5.73 3.12 2.88 5.95 5.50

10652000.000 2.99568 116520000.000 5.73 3.12 2.88 5.96 5.50

10659000.000 2.99578 116590000.000 5.73 3.12 2.88 5.96 5.50

10666000.000 2.99587 116660000.000 5.73 3.12 2.88 5.96 5.50

10673000.000 2.99597 116730000.000 5.73 3.12 2.88 5.96 5.50

10680000.000 2.99607 116800000.000 5.73 3.12 2.88 5.96 5.50

10687000.000 2.99617 116870000.000 5.73 3.12 2.88 5.96 5.50

10694000.000 2.99627 116940000.000 5.73 3.12 2.88 5.96 5.50

10701000.000 2.99637 117010000.000 5.73 3.12 2.88 5.96 5.50

10708000.000 2.99647 117080000.000 5.73 3.12 2.88 5.96 5.50

10715000.000 2.99656 117150000.000 5.73 3.12 2.88 5.96 5.50

10722000.000 2.99666 117220000.000 5.73 3.12 2.88 5.96 5.50

10729000.000 2.99676 117290000.000 5.73 3.12 2.88 5.96 5.50

10736000.000 2.99686 117360000.000 5.73 3.12 2.88 5.96 5.50

10743000.000 2.99696 117430000.000 5.73 3.12 2.88 5.96 5.50

10750000.000 2.99705 117500000.000 5.73 3.12 2.88 5.96 5.50

10757000.000 2.99715 117570000.000 5.73 3.12 2.88 5.96 5.50

10764000.000 2.99725 117640000.000 5.73 3.12 2.88 5.96 5.50

10771000.000 2.99735 117710000.000 5.73 3.12 2.88 5.96 5.50

10778000.000 2.99744 117780000.000 5.73 3.12 2.88 5.96 5.50

10785000.000 2.99754 117850000.000 5.73 3.12 2.88 5.96 5.50

10792000.000 2.99764 117920000.000 5.73 3.12 2.88 5.96 5.50

10799000.000 2.99774 117990000.000 5.73 3.12 2.88 5.96 5.50

10806000.000 2.99783 118060000.000 5.73 3.12 2.88 5.96 5.50

10813000.000 2.99793 118130000.000 5.73 3.12 2.88 5.96 5.50

10820000.000 2.99803 118200000.000 5.73 3.12 2.88 5.96 5.50

10827000.000 2.99813 118270000.000 5.73 3.12 2.88 5.96 5.50

10834000.000 2.99822 118340000.000 5.73 3.12 2.88 5.96 5.50

10841000.000 2.99832 118410000.000 5.73 3.12 2.88 5.96 5.50

10848000.000 2.99842 118480000.000 5.73 3.12 2.88 5.96 5.50

10855000.000 2.99852 118550000.000 5.73 3.12 2.88 5.96 5.50

10862000.000 2.99861 118620000.000 5.73 3.12 2.88 5.96 5.50

10869000.000 2.99871 118690000.000 5.73 3.12 2.88 5.96 5.50

10876000.000 2.99881 118760000.000 5.73 3.12 2.88 5.96 5.50

10883000.000 2.99890 118830000.000 5.73 3.12 2.88 5.96 5.50

10890000.000 2.99900 118900000.000 5.73 3.12 2.88 5.96 5.50

10897000.000 2.99910 118970000.000 5.73 3.12 2.88 5.96 5.50

10904000.000 2.99919 119040000.000 5.73 3.12 2.88 5.96 5.50

10911000.000 2.99929 119110000.000 5.73 3.12 2.88 5.96 5.50

10918000.000 2.99939 119180000.000 5.73 3.12 2.88 5.96 5.50

10925000.000 2.99948 119250000.000 5.73 3.12 2.88 5.96 5.50

10932000.000 2.99958 119320000.000 5.73 3.12 2.88 5.96 5.50

10939000.000 2.99967 119390000.000 5.73 3.12 2.88 5.96 5.50

10946000.000 2.99977 119460000.000 5.73 3.12 2.88 5.96 5.50

10953000.000 2.99987 119530000.000 5.73 3.12 2.88 5.96 5.50

10960000.000 2.99996 119600000.000 5.73 3.12 2.88 5.96 5.50

10967000.000 3.00006 119670000.000 5.73 3.12 2.88 5.96 5.50

10974000.000 3.00016 119740000.000 5.73 3.12 2.88 5.96 5.50

10981000.000 3.00025 119810000.000 5.73 3.12 2.88 5.96 5.50

10988000.000 3.00035 119880000.000 5.73 3.12 2.88 5.96 5.50

10995000.000 3.00044 119950000.000 5.73 3.12 2.88 5.96 5.51

11002000.000 3.00054 120020000.000 5.73 3.12 2.88 5.96 5.51

11009000.000 3.00063 120090000.000 5.73 3.12 2.88 5.96 5.51

11016000.000 3.00073 120160000.000 5.73 3.12 2.88 5.96 5.51

11023000.000 3.00083 120230000.000 5.74 3.12 2.88 5.96 5.51

11030000.000 3.00092 120300000.000 5.74 3.12 2.88 5.96 5.51

11037000.000 3.00102 120370000.000 5.74 3.12 2.88 5.96 5.51

11044000.000 3.00111 120440000.000 5.74 3.12 2.88 5.97 5.51

11051000.000 3.00121 120510000.000 5.74 3.12 2.88 5.97 5.51

11058000.000 3.00130 120580000.000 5.74 3.12 2.88 5.97 5.51

11065000.000 3.00140 120650000.000 5.74 3.12 2.88 5.97 5.51

11072000.000 3.00149 120720000.000 5.74 3.12 2.88 5.97 5.51

11079000.000 3.00159 120790000.000 5.74 3.12 2.88 5.97 5.51

11086000.000 3.00168 120860000.000 5.74 3.12 2.88 5.97 5.51

11093000.000 3.00178 120930000.000 5.74 3.12 2.88 5.97 5.51

11100000.000 3.00187 121000000.000 5.74 3.12 2.88 5.97 5.51

11107000.000 3.00197 121070000.000 5.74 3.12 2.88 5.97 5.51

11114000.000 3.00206 121140000.000 5.74 3.12 2.88 5.97 5.51

11121000.000 3.00216 121210000.000 5.74 3.12 2.88 5.97 5.51

11128000.000 3.00225 121280000.000 5.74 3.12 2.88 5.97 5.51

11135000.000 3.00235 121350000.000 5.74 3.12 2.88 5.97 5.51

11142000.000 3.00244 121420000.000 5.74 3.12 2.88 5.97 5.51

11149000.000 3.00254 121490000.000 5.74 3.12 2.88 5.97 5.51

11156000.000 3.00263 121560000.000 5.74 3.12 2.88 5.97 5.51

11163000.000 3.00273 121630000.000 5.74 3.12 2.88 5.97 5.51

11170000.000 3.00282 121700000.000 5.74 3.12 2.88 5.97 5.51

11177000.000 3.00292 121770000.000 5.74 3.12 2.88 5.97 5.51

11184000.000 3.00301 121840000.000 5.74 3.12 2.88 5.97 5.51

11191000.000 3.00310 121910000.000 5.74 3.12 2.88 5.97 5.51

11198000.000 3.00320 121980000.000 5.74 3.12 2.88 5.97 5.51

11205000.000 3.00329 122050000.000 5.74 3.12 2.88 5.97 5.51

11212000.000 3.00339 122120000.000 5.74 3.12 2.88 5.97 5.51

11219000.000 3.00348 122190000.000 5.74 3.12 2.88 5.97 5.51

11226000.000 3.00357 122260000.000 5.74 3.12 2.88 5.97 5.51

11233000.000 3.00367 122330000.000 5.74 3.12 2.88 5.97 5.51

11240000.000 3.00376 122400000.000 5.74 3.12 2.88 5.97 5.51

11247000.000 3.00386 122470000.000 5.74 3.12 2.88 5.97 5.51

11254000.000 3.00395 122540000.000 5.74 3.12 2.88 5.97 5.51

11261000.000 3.00404 122610000.000 5.74 3.12 2.88 5.97 5.51

11268000.000 3.00414 122680000.000 5.74 3.12 2.88 5.97 5.51

11275000.000 3.00423 122750000.000 5.74 3.12 2.88 5.97 5.51

11282000.000 3.00432 122820000.000 5.74 3.12 2.88 5.97 5.51

11289000.000 3.00442 122890000.000 5.74 3.12 2.88 5.97 5.51

11296000.000 3.00451 122960000.000 5.74 3.12 2.88 5.97 5.51

11303000.000 3.00460 123030000.000 5.74 3.12 2.88 5.97 5.51

11310000.000 3.00470 123100000.000 5.74 3.12 2.88 5.97 5.51

11317000.000 3.00479 123170000.000 5.74 3.12 2.88 5.97 5.51

11324000.000 3.00488 123240000.000 5.74 3.13 2.88 5.97 5.51

11331000.000 3.00498 123310000.000 5.74 3.13 2.88 5.97 5.51

11338000.000 3.00507 123380000.000 5.74 3.13 2.88 5.97 5.51

11345000.000 3.00516 123450000.000 5.74 3.13 2.88 5.97 5.51

11352000.000 3.00526 123520000.000 5.74 3.13 2.89 5.97 5.51

11359000.000 3.00535 123590000.000 5.74 3.13 2.89 5.97 5.51

11366000.000 3.00544 123660000.000 5.74 3.13 2.89 5.97 5.51

11373000.000 3.00553 123730000.000 5.74 3.13 2.89 5.97 5.51

11380000.000 3.00563 123800000.000 5.74 3.13 2.89 5.97 5.51

11387000.000 3.00572 123870000.000 5.74 3.13 2.89 5.97 5.51

11394000.000 3.00581 123940000.000 5.74 3.13 2.89 5.97 5.51

11401000.000 3.00591 124010000.000 5.74 3.13 2.89 5.97 5.51

11408000.000 3.00600 124080000.000 5.74 3.13 2.89 5.97 5.51

11415000.000 3.00609 124150000.000 5.74 3.13 2.89 5.97 5.51

11422000.000 3.00618 124220000.000 5.74 3.13 2.89 5.97 5.51

11429000.000 3.00628 124290000.000 5.74 3.13 2.89 5.97 5.51

11436000.000 3.00637 124360000.000 5.74 3.13 2.89 5.97 5.52

11443000.000 3.00646 124430000.000 5.74 3.13 2.89 5.97 5.52

11450000.000 3.00655 124500000.000 5.75 3.13 2.89 5.97 5.52

11457000.000 3.00664 124570000.000 5.75 3.13 2.89 5.98 5.52

11464000.000 3.00674 124640000.000 5.75 3.13 2.89 5.98 5.52

11471000.000 3.00683 124710000.000 5.75 3.13 2.89 5.98 5.52

11478000.000 3.00692 124780000.000 5.75 3.13 2.89 5.98 5.52

11485000.000 3.00701 124850000.000 5.75 3.13 2.89 5.98 5.52

11492000.000 3.00710 124920000.000 5.75 3.13 2.89 5.98 5.52

11499000.000 3.00720 124990000.000 5.75 3.13 2.89 5.98 5.52

11506000.000 3.00729 125060000.000 5.75 3.13 2.89 5.98 5.52

11513000.000 3.00738 125130000.000 5.75 3.13 2.89 5.98 5.52

11520000.000 3.00747 125200000.000 5.75 3.13 2.89 5.98 5.52

11527000.000 3.00756 125270000.000 5.75 3.13 2.89 5.98 5.52

11534000.000 3.00765 125340000.000 5.75 3.13 2.89 5.98 5.52

11541000.000 3.00775 125410000.000 5.75 3.13 2.89 5.98 5.52

11548000.000 3.00784 125480000.000 5.75 3.13 2.89 5.98 5.52

11555000.000 3.00793 125550000.000 5.75 3.13 2.89 5.98 5.52

11562000.000 3.00802 125620000.000 5.75 3.13 2.89 5.98 5.52

11569000.000 3.00811 125690000.000 5.75 3.13 2.89 5.98 5.52

11576000.000 3.00820 125760000.000 5.75 3.13 2.89 5.98 5.52

11583000.000 3.00829 125830000.000 5.75 3.13 2.89 5.98 5.52

11590000.000 3.00839 125900000.000 5.75 3.13 2.89 5.98 5.52

11597000.000 3.00848 125970000.000 5.75 3.13 2.89 5.98 5.52

11604000.000 3.00857 126040000.000 5.75 3.13 2.89 5.98 5.52

11611000.000 3.00866 126110000.000 5.75 3.13 2.89 5.98 5.52

11618000.000 3.00875 126180000.000 5.75 3.13 2.89 5.98 5.52

11625000.000 3.00884 126250000.000 5.75 3.13 2.89 5.98 5.52

11632000.000 3.00893 126320000.000 5.75 3.13 2.89 5.98 5.52

11639000.000 3.00902 126390000.000 5.75 3.13 2.89 5.98 5.52

11646000.000 3.00911 126460000.000 5.75 3.13 2.89 5.98 5.52

11653000.000 3.00920 126530000.000 5.75 3.13 2.89 5.98 5.52

11660000.000 3.00929 126600000.000 5.75 3.13 2.89 5.98 5.52

11667000.000 3.00938 126670000.000 5.75 3.13 2.89 5.98 5.52

11674000.000 3.00948 126740000.000 5.75 3.13 2.89 5.98 5.52

11681000.000 3.00957 126810000.000 5.75 3.13 2.89 5.98 5.52

11688000.000 3.00966 126880000.000 5.75 3.13 2.89 5.98 5.52

11695000.000 3.00975 126950000.000 5.75 3.13 2.89 5.98 5.52

11702000.000 3.00984 127020000.000 5.75 3.13 2.89 5.98 5.52

11709000.000 3.00993 127090000.000 5.75 3.13 2.89 5.98 5.52

11716000.000 3.01002 127160000.000 5.75 3.13 2.89 5.98 5.52

11723000.000 3.01011 127230000.000 5.75 3.13 2.89 5.98 5.52

11730000.000 3.01020 127300000.000 5.75 3.13 2.89 5.98 5.52

11737000.000 3.01029 127370000.000 5.75 3.13 2.89 5.98 5.52

11744000.000 3.01038 127440000.000 5.75 3.13 2.89 5.98 5.52

11751000.000 3.01047 127510000.000 5.75 3.13 2.89 5.98 5.52

11758000.000 3.01056 127580000.000 5.75 3.13 2.89 5.98 5.52

11765000.000 3.01065 127650000.000 5.75 3.13 2.89 5.98 5.52

11772000.000 3.01074 127720000.000 5.75 3.13 2.89 5.98 5.52

11779000.000 3.01083 127790000.000 5.75 3.13 2.89 5.98 5.52

11786000.000 3.01092 127860000.000 5.75 3.13 2.89 5.98 5.52

11793000.000 3.01101 127930000.000 5.75 3.13 2.89 5.98 5.52

11800000.000 3.01110 128000000.000 5.75 3.13 2.89 5.98 5.52

11807000.000 3.01119 128070000.000 5.75 3.13 2.89 5.98 5.52

11814000.000 3.01128 128140000.000 5.75 3.13 2.89 5.98 5.52

11821000.000 3.01136 128210000.000 5.75 3.13 2.89 5.98 5.52

11828000.000 3.01145 128280000.000 5.75 3.13 2.89 5.98 5.52

11835000.000 3.01154 128350000.000 5.75 3.13 2.89 5.98 5.52

11842000.000 3.01163 128420000.000 5.75 3.13 2.89 5.98 5.52

11849000.000 3.01172 128490000.000 5.75 3.13 2.89 5.98 5.52

11856000.000 3.01181 128560000.000 5.75 3.13 2.89 5.98 5.52

11863000.000 3.01190 128630000.000 5.75 3.13 2.89 5.98 5.52

11870000.000 3.01199 128700000.000 5.75 3.13 2.89 5.98 5.52

11877000.000 3.01208 128770000.000 5.75 3.13 2.89 5.99 5.52

11884000.000 3.01217 128840000.000 5.76 3.13 2.89 5.99 5.52

11891000.000 3.01226 128910000.000 5.76 3.13 2.89 5.99 5.52

11898000.000 3.01235 128980000.000 5.76 3.13 2.89 5.99 5.53

11905000.000 3.01243 129050000.000 5.76 3.13 2.89 5.99 5.53

11912000.000 3.01252 129120000.000 5.76 3.13 2.89 5.99 5.53

11919000.000 3.01261 129190000.000 5.76 3.13 2.89 5.99 5.53

11926000.000 3.01270 129260000.000 5.76 3.13 2.89 5.99 5.53

11933000.000 3.01279 129330000.000 5.76 3.13 2.89 5.99 5.53

11940000.000 3.01288 129400000.000 5.76 3.13 2.89 5.99 5.53

11947000.000 3.01297 129470000.000 5.76 3.13 2.89 5.99 5.53

11954000.000 3.01306 129540000.000 5.76 3.13 2.89 5.99 5.53

11961000.000 3.01314 129610000.000 5.76 3.13 2.89 5.99 5.53

11968000.000 3.01323 129680000.000 5.76 3.13 2.89 5.99 5.53

11975000.000 3.01332 129750000.000 5.76 3.13 2.89 5.99 5.53

11982000.000 3.01341 129820000.000 5.76 3.13 2.89 5.99 5.53

11989000.000 3.01350 129890000.000 5.76 3.13 2.89 5.99 5.53

11996000.000 3.01359 129960000.000 5.76 3.13 2.89 5.99 5.53

12003000.000 3.01367 130030000.000 5.76 3.13 2.89 5.99 5.53

12010000.000 3.01376 130100000.000 5.76 3.13 2.89 5.99 5.53

12017000.000 3.01385 130170000.000 5.76 3.13 2.89 5.99 5.53

12024000.000 3.01394 130240000.000 5.76 3.13 2.89 5.99 5.53

12031000.000 3.01403 130310000.000 5.76 3.13 2.89 5.99 5.53

12038000.000 3.01411 130380000.000 5.76 3.13 2.89 5.99 5.53

12045000.000 3.01420 130450000.000 5.76 3.13 2.89 5.99 5.53

12052000.000 3.01429 130520000.000 5.76 3.13 2.89 5.99 5.53

12059000.000 3.01438 130590000.000 5.76 3.13 2.89 5.99 5.53

12066000.000 3.01447 130660000.000 5.76 3.14 2.89 5.99 5.53

12073000.000 3.01455 130730000.000 5.76 3.14 2.89 5.99 5.53

12080000.000 3.01464 130800000.000 5.76 3.14 2.89 5.99 5.53

12087000.000 3.01473 130870000.000 5.76 3.14 2.89 5.99 5.53

12094000.000 3.01482 130940000.000 5.76 3.14 2.89 5.99 5.53

12101000.000 3.01490 131010000.000 5.76 3.14 2.89 5.99 5.53

12108000.000 3.01499 131080000.000 5.76 3.14 2.89 5.99 5.53

12115000.000 3.01508 131150000.000 5.76 3.14 2.89 5.99 5.53

12122000.000 3.01517 131220000.000 5.76 3.14 2.89 5.99 5.53

12129000.000 3.01525 131290000.000 5.76 3.14 2.89 5.99 5.53

12136000.000 3.01534 131360000.000 5.76 3.14 2.89 5.99 5.53

12143000.000 3.01543 131430000.000 5.76 3.14 2.89 5.99 5.53

12150000.000 3.01551 131500000.000 5.76 3.14 2.89 5.99 5.53

12157000.000 3.01560 131570000.000 5.76 3.14 2.89 5.99 5.53

12164000.000 3.01569 131640000.000 5.76 3.14 2.90 5.99 5.53

12171000.000 3.01578 131710000.000 5.76 3.14 2.90 5.99 5.53

12178000.000 3.01586 131780000.000 5.76 3.14 2.90 5.99 5.53

12185000.000 3.01595 131850000.000 5.76 3.14 2.90 5.99 5.53

12192000.000 3.01604 131920000.000 5.76 3.14 2.90 5.99 5.53

12199000.000 3.01612 131990000.000 5.76 3.14 2.90 5.99 5.53

12206000.000 3.01621 132060000.000 5.76 3.14 2.90 5.99 5.53

12213000.000 3.01630 132130000.000 5.76 3.14 2.90 5.99 5.53

12220000.000 3.01638 132200000.000 5.76 3.14 2.90 5.99 5.53

12227000.000 3.01647 132270000.000 5.76 3.14 2.90 5.99 5.53

12234000.000 3.01656 132340000.000 5.76 3.14 2.90 5.99 5.53

12241000.000 3.01664 132410000.000 5.76 3.14 2.90 5.99 5.53

12248000.000 3.01673 132480000.000 5.76 3.14 2.90 5.99 5.53

12255000.000 3.01682 132550000.000 5.76 3.14 2.90 5.99 5.53

12262000.000 3.01690 132620000.000 5.76 3.14 2.90 5.99 5.53

12269000.000 3.01699 132690000.000 5.76 3.14 2.90 5.99 5.53

12276000.000 3.01708 132760000.000 5.76 3.14 2.90 5.99 5.53

12283000.000 3.01716 132830000.000 5.76 3.14 2.90 5.99 5.53

12290000.000 3.01725 132900000.000 5.76 3.14 2.90 5.99 5.53

12297000.000 3.01733 132970000.000 5.76 3.14 2.90 5.99 5.53

12304000.000 3.01742 133040000.000 5.76 3.14 2.90 5.99 5.53

12311000.000 3.01751 133110000.000 5.76 3.14 2.90 6.00 5.53

12318000.000 3.01759 133180000.000 5.76 3.14 2.90 6.00 5.53

12325000.000 3.01768 133250000.000 5.76 3.14 2.90 6.00 5.53

12332000.000 3.01776 133320000.000 5.76 3.14 2.90 6.00 5.53

12339000.000 3.01785 133390000.000 5.77 3.14 2.90 6.00 5.53

12346000.000 3.01794 133460000.000 5.77 3.14 2.90 6.00 5.53

12353000.000 3.01802 133530000.000 5.77 3.14 2.90 6.00 5.53

12360000.000 3.01811 133600000.000 5.77 3.14 2.90 6.00 5.53

12367000.000 3.01819 133670000.000 5.77 3.14 2.90 6.00 5.54

12374000.000 3.01828 133740000.000 5.77 3.14 2.90 6.00 5.54

12381000.000 3.01836 133810000.000 5.77 3.14 2.90 6.00 5.54

12388000.000 3.01845 133880000.000 5.77 3.14 2.90 6.00 5.54

12395000.000 3.01854 133950000.000 5.77 3.14 2.90 6.00 5.54

12402000.000 3.01862 134020000.000 5.77 3.14 2.90 6.00 5.54

12409000.000 3.01871 134090000.000 5.77 3.14 2.90 6.00 5.54

12416000.000 3.01879 134160000.000 5.77 3.14 2.90 6.00 5.54

12423000.000 3.01888 134230000.000 5.77 3.14 2.90 6.00 5.54

12430000.000 3.01896 134300000.000 5.77 3.14 2.90 6.00 5.54

12437000.000 3.01905 134370000.000 5.77 3.14 2.90 6.00 5.54

12444000.000 3.01913 134440000.000 5.77 3.14 2.90 6.00 5.54

12451000.000 3.01922 134510000.000 5.77 3.14 2.90 6.00 5.54

12458000.000 3.01930 134580000.000 5.77 3.14 2.90 6.00 5.54

12465000.000 3.01939 134650000.000 5.77 3.14 2.90 6.00 5.54

12472000.000 3.01947 134720000.000 5.77 3.14 2.90 6.00 5.54

12479000.000 3.01956 134790000.000 5.77 3.14 2.90 6.00 5.54

12486000.000 3.01964 134860000.000 5.77 3.14 2.90 6.00 5.54

12493000.000 3.01973 134930000.000 5.77 3.14 2.90 6.00 5.54

12500000.000 3.01981 135000000.000 5.77 3.14 2.90 6.00 5.54

12507000.000 3.01990 135070000.000 5.77 3.14 2.90 6.00 5.54

12514000.000 3.01998 135140000.000 5.77 3.14 2.90 6.00 5.54

12521000.000 3.02007 135210000.000 5.77 3.14 2.90 6.00 5.54

12528000.000 3.02015 135280000.000 5.77 3.14 2.90 6.00 5.54

12535000.000 3.02024 135350000.000 5.77 3.14 2.90 6.00 5.54

12542000.000 3.02032 135420000.000 5.77 3.14 2.90 6.00 5.54

12549000.000 3.02041 135490000.000 5.77 3.14 2.90 6.00 5.54

12556000.000 3.02049 135560000.000 5.77 3.14 2.90 6.00 5.54

12563000.000 3.02057 135630000.000 5.77 3.14 2.90 6.00 5.54

12570000.000 3.02066 135700000.000 5.77 3.14 2.90 6.00 5.54

12577000.000 3.02074 135770000.000 5.77 3.14 2.90 6.00 5.54

12584000.000 3.02083 135840000.000 5.77 3.14 2.90 6.00 5.54

12591000.000 3.02091 135910000.000 5.77 3.14 2.90 6.00 5.54

12598000.000 3.02100 135980000.000 5.77 3.14 2.90 6.00 5.54

12605000.000 3.02108 136050000.000 5.77 3.14 2.90 6.00 5.54

12612000.000 3.02116 136120000.000 5.77 3.14 2.90 6.00 5.54

12619000.000 3.02125 136190000.000 5.77 3.14 2.90 6.00 5.54

12626000.000 3.02133 136260000.000 5.77 3.14 2.90 6.00 5.54

12633000.000 3.02142 136330000.000 5.77 3.14 2.90 6.00 5.54

12640000.000 3.02150 136400000.000 5.77 3.14 2.90 6.00 5.54

12647000.000 3.02158 136470000.000 5.77 3.14 2.90 6.00 5.54

12654000.000 3.02167 136540000.000 5.77 3.14 2.90 6.00 5.54

12661000.000 3.02175 136610000.000 5.77 3.14 2.90 6.00 5.54

12668000.000 3.02184 136680000.000 5.77 3.14 2.90 6.00 5.54

12675000.000 3.02192 136750000.000 5.77 3.14 2.90 6.00 5.54

12682000.000 3.02200 136820000.000 5.77 3.14 2.90 6.00 5.54

12689000.000 3.02209 136890000.000 5.77 3.14 2.90 6.00 5.54

12696000.000 3.02217 136960000.000 5.77 3.14 2.90 6.00 5.54

12703000.000 3.02225 137030000.000 5.77 3.14 2.90 6.00 5.54

12710000.000 3.02234 137100000.000 5.77 3.14 2.90 6.00 5.54

12717000.000 3.02242 137170000.000 5.77 3.14 2.90 6.00 5.54

12724000.000 3.02250 137240000.000 5.77 3.14 2.90 6.00 5.54

12731000.000 3.02259 137310000.000 5.77 3.14 2.90 6.00 5.54

12738000.000 3.02267 137380000.000 5.77 3.14 2.90 6.00 5.54

12745000.000 3.02275 137450000.000 5.77 3.14 2.90 6.00 5.54

12752000.000 3.02284 137520000.000 5.77 3.14 2.90 6.00 5.54

12759000.000 3.02292 137590000.000 5.77 3.14 2.90 6.00 5.54

12766000.000 3.02300 137660000.000 5.77 3.14 2.90 6.01 5.54

12773000.000 3.02309 137730000.000 5.77 3.14 2.90 6.01 5.54

12780000.000 3.02317 137800000.000 5.77 3.14 2.90 6.01 5.54

12787000.000 3.02325 137870000.000 5.77 3.14 2.90 6.01 5.54

12794000.000 3.02334 137940000.000 5.77 3.14 2.90 6.01 5.54

12801000.000 3.02342 138010000.000 5.77 3.14 2.90 6.01 5.54

12808000.000 3.02350 138080000.000 5.78 3.14 2.90 6.01 5.54

12815000.000 3.02359 138150000.000 5.78 3.14 2.90 6.01 5.54

12822000.000 3.02367 138220000.000 5.78 3.14 2.90 6.01 5.54

12829000.000 3.02375 138290000.000 5.78 3.14 2.90 6.01 5.54

12836000.000 3.02383 138360000.000 5.78 3.14 2.90 6.01 5.54

12843000.000 3.02392 138430000.000 5.78 3.14 2.90 6.01 5.54

12850000.000 3.02400 138500000.000 5.78 3.14 2.90 6.01 5.54

12857000.000 3.02408 138570000.000 5.78 3.15 2.90 6.01 5.55

12864000.000 3.02416 138640000.000 5.78 3.15 2.90 6.01 5.55

12871000.000 3.02425 138710000.000 5.78 3.15 2.90 6.01 5.55

12878000.000 3.02433 138780000.000 5.78 3.15 2.90 6.01 5.55

12885000.000 3.02441 138850000.000 5.78 3.15 2.90 6.01 5.55

12892000.000 3.02449 138920000.000 5.78 3.15 2.90 6.01 5.55

12899000.000 3.02458 138990000.000 5.78 3.15 2.90 6.01 5.55

12906000.000 3.02466 139060000.000 5.78 3.15 2.90 6.01 5.55

12913000.000 3.02474 139130000.000 5.78 3.15 2.90 6.01 5.55

12920000.000 3.02482 139200000.000 5.78 3.15 2.90 6.01 5.55

12927000.000 3.02491 139270000.000 5.78 3.15 2.90 6.01 5.55

12934000.000 3.02499 139340000.000 5.78 3.15 2.90 6.01 5.55

12941000.000 3.02507 139410000.000 5.78 3.15 2.90 6.01 5.55

12948000.000 3.02515 139480000.000 5.78 3.15 2.90 6.01 5.55

12955000.000 3.02523 139550000.000 5.78 3.15 2.90 6.01 5.55

12962000.000 3.02532 139620000.000 5.78 3.15 2.90 6.01 5.55

12969000.000 3.02540 139690000.000 5.78 3.15 2.90 6.01 5.55

12976000.000 3.02548 139760000.000 5.78 3.15 2.90 6.01 5.55

12983000.000 3.02556 139830000.000 5.78 3.15 2.90 6.01 5.55

12990000.000 3.02564 139900000.000 5.78 3.15 2.90 6.01 5.55

12997000.000 3.02572 139970000.000 5.78 3.15 2.90 6.01 5.55

13004000.000 3.02581 140040000.000 5.78 3.15 2.90 6.01 5.55

13011000.000 3.02589 140110000.000 5.78 3.15 2.90 6.01 5.55

13018000.000 3.02597 140180000.000 5.78 3.15 2.90 6.01 5.55

13025000.000 3.02605 140250000.000 5.78 3.15 2.91 6.01 5.55

13032000.000 3.02613 140320000.000 5.78 3.15 2.91 6.01 5.55

13039000.000 3.02621 140390000.000 5.78 3.15 2.91 6.01 5.55

13046000.000 3.02630 140460000.000 5.78 3.15 2.91 6.01 5.55

13053000.000 3.02638 140530000.000 5.78 3.15 2.91 6.01 5.55

13060000.000 3.02646 140600000.000 5.78 3.15 2.91 6.01 5.55

13067000.000 3.02654 140670000.000 5.78 3.15 2.91 6.01 5.55

13074000.000 3.02662 140740000.000 5.78 3.15 2.91 6.01 5.55

13081000.000 3.02670 140810000.000 5.78 3.15 2.91 6.01 5.55

13088000.000 3.02678 140880000.000 5.78 3.15 2.91 6.01 5.55

13095000.000 3.02687 140950000.000 5.78 3.15 2.91 6.01 5.55

13102000.000 3.02695 141020000.000 5.78 3.15 2.91 6.01 5.55

13109000.000 3.02703 141090000.000 5.78 3.15 2.91 6.01 5.55

13116000.000 3.02711 141160000.000 5.78 3.15 2.91 6.01 5.55

13123000.000 3.02719 141230000.000 5.78 3.15 2.91 6.01 5.55

13130000.000 3.02727 141300000.000 5.78 3.15 2.91 6.01 5.55

13137000.000 3.02735 141370000.000 5.78 3.15 2.91 6.01 5.55

13144000.000 3.02743 141440000.000 5.78 3.15 2.91 6.01 5.55

13151000.000 3.02751 141510000.000 5.78 3.15 2.91 6.01 5.55

13158000.000 3.02759 141580000.000 5.78 3.15 2.91 6.01 5.55

13165000.000 3.02767 141650000.000 5.78 3.15 2.91 6.01 5.55

13172000.000 3.02776 141720000.000 5.78 3.15 2.91 6.01 5.55

13179000.000 3.02784 141790000.000 5.78 3.15 2.91 6.01 5.55

13186000.000 3.02792 141860000.000 5.78 3.15 2.91 6.01 5.55

13193000.000 3.02800 141930000.000 5.78 3.15 2.91 6.01 5.55

13200000.000 3.02808 142000000.000 5.78 3.15 2.91 6.01 5.55

13207000.000 3.02816 142070000.000 5.78 3.15 2.91 6.01 5.55

13214000.000 3.02824 142140000.000 5.78 3.15 2.91 6.01 5.55

13221000.000 3.02832 142210000.000 5.78 3.15 2.91 6.01 5.55

13228000.000 3.02840 142280000.000 5.78 3.15 2.91 6.02 5.55

13235000.000 3.02848 142350000.000 5.78 3.15 2.91 6.02 5.55

13242000.000 3.02856 142420000.000 5.78 3.15 2.91 6.02 5.55

13249000.000 3.02864 142490000.000 5.78 3.15 2.91 6.02 5.55

13256000.000 3.02872 142560000.000 5.78 3.15 2.91 6.02 5.55

13263000.000 3.02880 142630000.000 5.78 3.15 2.91 6.02 5.55

13270000.000 3.02888 142700000.000 5.78 3.15 2.91 6.02 5.55

13277000.000 3.02896 142770000.000 5.78 3.15 2.91 6.02 5.55

13284000.000 3.02904 142840000.000 5.78 3.15 2.91 6.02 5.55

13291000.000 3.02912 142910000.000 5.79 3.15 2.91 6.02 5.55

13298000.000 3.02920 142980000.000 5.79 3.15 2.91 6.02 5.55

13305000.000 3.02928 143050000.000 5.79 3.15 2.91 6.02 5.55

13312000.000 3.02936 143120000.000 5.79 3.15 2.91 6.02 5.55

13319000.000 3.02944 143190000.000 5.79 3.15 2.91 6.02 5.55

13326000.000 3.02952 143260000.000 5.79 3.15 2.91 6.02 5.55

13333000.000 3.02960 143330000.000 5.79 3.15 2.91 6.02 5.55

13340000.000 3.02968 143400000.000 5.79 3.15 2.91 6.02 5.55

13347000.000 3.02976 143470000.000 5.79 3.15 2.91 6.02 5.55

13354000.000 3.02984 143540000.000 5.79 3.15 2.91 6.02 5.55

13361000.000 3.02992 143610000.000 5.79 3.15 2.91 6.02 5.55

13368000.000 3.03000 143680000.000 5.79 3.15 2.91 6.02 5.56

13375000.000 3.03008 143750000.000 5.79 3.15 2.91 6.02 5.56

13382000.000 3.03016 143820000.000 5.79 3.15 2.91 6.02 5.56

13389000.000 3.03024 143890000.000 5.79 3.15 2.91 6.02 5.56

13396000.000 3.03032 143960000.000 5.79 3.15 2.91 6.02 5.56

13403000.000 3.03040 144030000.000 5.79 3.15 2.91 6.02 5.56

13410000.000 3.03048 144100000.000 5.79 3.15 2.91 6.02 5.56

13417000.000 3.03056 144170000.000 5.79 3.15 2.91 6.02 5.56

13424000.000 3.03063 144240000.000 5.79 3.15 2.91 6.02 5.56

13431000.000 3.03071 144310000.000 5.79 3.15 2.91 6.02 5.56

13438000.000 3.03079 144380000.000 5.79 3.15 2.91 6.02 5.56

13445000.000 3.03087 144450000.000 5.79 3.15 2.91 6.02 5.56

13452000.000 3.03095 144520000.000 5.79 3.15 2.91 6.02 5.56

13459000.000 3.03103 144590000.000 5.79 3.15 2.91 6.02 5.56

13466000.000 3.03111 144660000.000 5.79 3.15 2.91 6.02 5.56

13473000.000 3.03119 144730000.000 5.79 3.15 2.91 6.02 5.56

13480000.000 3.03127 144800000.000 5.79 3.15 2.91 6.02 5.56

13487000.000 3.03135 144870000.000 5.79 3.15 2.91 6.02 5.56

13494000.000 3.03143 144940000.000 5.79 3.15 2.91 6.02 5.56

13501000.000 3.03150 145010000.000 5.79 3.15 2.91 6.02 5.56

13508000.000 3.03158 145080000.000 5.79 3.15 2.91 6.02 5.56

13515000.000 3.03166 145150000.000 5.79 3.15 2.91 6.02 5.56

13522000.000 3.03174 145220000.000 5.79 3.15 2.91 6.02 5.56

13529000.000 3.03182 145290000.000 5.79 3.15 2.91 6.02 5.56

13536000.000 3.03190 145360000.000 5.79 3.15 2.91 6.02 5.56

13543000.000 3.03198 145430000.000 5.79 3.15 2.91 6.02 5.56

13550000.000 3.03206 145500000.000 5.79 3.15 2.91 6.02 5.56

13557000.000 3.03213 145570000.000 5.79 3.15 2.91 6.02 5.56

13564000.000 3.03221 145640000.000 5.79 3.15 2.91 6.02 5.56

13571000.000 3.03229 145710000.000 5.79 3.15 2.91 6.02 5.56

13578000.000 3.03237 145780000.000 5.79 3.15 2.91 6.02 5.56

13585000.000 3.03245 145850000.000 5.79 3.15 2.91 6.02 5.56

13592000.000 3.03253 145920000.000 5.79 3.15 2.91 6.02 5.56

13599000.000 3.03260 145990000.000 5.79 3.15 2.91 6.02 5.56

13606000.000 3.03268 146060000.000 5.79 3.15 2.91 6.02 5.56

13613000.000 3.03276 146130000.000 5.79 3.15 2.91 6.02 5.56

13620000.000 3.03284 146200000.000 5.79 3.15 2.91 6.02 5.56

13627000.000 3.03292 146270000.000 5.79 3.15 2.91 6.02 5.56

13634000.000 3.03300 146340000.000 5.79 3.15 2.91 6.02 5.56

13641000.000 3.03307 146410000.000 5.79 3.15 2.91 6.02 5.56

13648000.000 3.03315 146480000.000 5.79 3.15 2.91 6.02 5.56

13655000.000 3.03323 146550000.000 5.79 3.15 2.91 6.02 5.56

13662000.000 3.03331 146620000.000 5.79 3.15 2.91 6.02 5.56

13669000.000 3.03339 146690000.000 5.79 3.15 2.91 6.02 5.56

13676000.000 3.03346 146760000.000 5.79 3.15 2.91 6.02 5.56

13683000.000 3.03354 146830000.000 5.79 3.15 2.91 6.02 5.56

13690000.000 3.03362 146900000.000 5.79 3.15 2.91 6.02 5.56

13697000.000 3.03370 146970000.000 5.79 3.16 2.91 6.02 5.56

13704000.000 3.03377 147040000.000 5.79 3.16 2.91 6.03 5.56

13711000.000 3.03385 147110000.000 5.79 3.16 2.91 6.03 5.56

13718000.000 3.03393 147180000.000 5.79 3.16 2.91 6.03 5.56

13725000.000 3.03401 147250000.000 5.79 3.16 2.91 6.03 5.56

13732000.000 3.03409 147320000.000 5.79 3.16 2.91 6.03 5.56

13739000.000 3.03416 147390000.000 5.79 3.16 2.91 6.03 5.56

13746000.000 3.03424 147460000.000 5.79 3.16 2.91 6.03 5.56

13753000.000 3.03432 147530000.000 5.79 3.16 2.91 6.03 5.56

13760000.000 3.03440 147600000.000 5.79 3.16 2.91 6.03 5.56

13767000.000 3.03447 147670000.000 5.79 3.16 2.91 6.03 5.56

13774000.000 3.03455 147740000.000 5.79 3.16 2.91 6.03 5.56

13781000.000 3.03463 147810000.000 5.79 3.16 2.91 6.03 5.56

13788000.000 3.03470 147880000.000 5.79 3.16 2.91 6.03 5.56

13795000.000 3.03478 147950000.000 5.80 3.16 2.91 6.03 5.56

13802000.000 3.03486 148020000.000 5.80 3.16 2.91 6.03 5.56

13809000.000 3.03494 148090000.000 5.80 3.16 2.91 6.03 5.56

13816000.000 3.03501 148160000.000 5.80 3.16 2.91 6.03 5.56

13823000.000 3.03509 148230000.000 5.80 3.16 2.91 6.03 5.56

13830000.000 3.03517 148300000.000 5.80 3.16 2.91 6.03 5.56

13837000.000 3.03524 148370000.000 5.80 3.16 2.91 6.03 5.56

13844000.000 3.03532 148440000.000 5.80 3.16 2.91 6.03 5.56

13851000.000 3.03540 148510000.000 5.80 3.16 2.91 6.03 5.56

13858000.000 3.03548 148580000.000 5.80 3.16 2.91 6.03 5.56

13865000.000 3.03555 148650000.000 5.80 3.16 2.91 6.03 5.56

13872000.000 3.03563 148720000.000 5.80 3.16 2.91 6.03 5.56

13879000.000 3.03571 148790000.000 5.80 3.16 2.91 6.03 5.56

13886000.000 3.03578 148860000.000 5.80 3.16 2.91 6.03 5.57

13893000.000 3.03586 148930000.000 5.80 3.16 2.91 6.03 5.57

13900000.000 3.03594 149000000.000 5.80 3.16 2.91 6.03 5.57

13907000.000 3.03601 149070000.000 5.80 3.16 2.91 6.03 5.57

13914000.000 3.03609 149140000.000 5.80 3.16 2.91 6.03 5.57

13921000.000 3.03617 149210000.000 5.80 3.16 2.91 6.03 5.57

13928000.000 3.03624 149280000.000 5.80 3.16 2.91 6.03 5.57

13935000.000 3.03632 149350000.000 5.80 3.16 2.91 6.03 5.57

13942000.000 3.03640 149420000.000 5.80 3.16 2.91 6.03 5.57

13949000.000 3.03647 149490000.000 5.80 3.16 2.92 6.03 5.57

13956000.000 3.03655 149560000.000 5.80 3.16 2.92 6.03 5.57

13963000.000 3.03663 149630000.000 5.80 3.16 2.92 6.03 5.57

13970000.000 3.03670 149700000.000 5.80 3.16 2.92 6.03 5.57

13977000.000 3.03678 149770000.000 5.80 3.16 2.92 6.03 5.57

13984000.000 3.03685 149840000.000 5.80 3.16 2.92 6.03 5.57

13991000.000 3.03693 149910000.000 5.80 3.16 2.92 6.03 5.57

13998000.000 3.03701 149980000.000 5.80 3.16 2.92 6.03 5.57

14005000.000 3.03708 150050000.000 5.80 3.16 2.92 6.03 5.57

14012000.000 3.03716 150120000.000 5.80 3.16 2.92 6.03 5.57

14019000.000 3.03723 150190000.000 5.80 3.16 2.92 6.03 5.57

14026000.000 3.03731 150260000.000 5.80 3.16 2.92 6.03 5.57

14033000.000 3.03739 150330000.000 5.80 3.16 2.92 6.03 5.57

14040000.000 3.03746 150400000.000 5.80 3.16 2.92 6.03 5.57

14047000.000 3.03754 150470000.000 5.80 3.16 2.92 6.03 5.57

14054000.000 3.03761 150540000.000 5.80 3.16 2.92 6.03 5.57

14061000.000 3.03769 150610000.000 5.80 3.16 2.92 6.03 5.57

14068000.000 3.03777 150680000.000 5.80 3.16 2.92 6.03 5.57

14075000.000 3.03784 150750000.000 5.80 3.16 2.92 6.03 5.57

14082000.000 3.03792 150820000.000 5.80 3.16 2.92 6.03 5.57

14089000.000 3.03799 150890000.000 5.80 3.16 2.92 6.03 5.57

14096000.000 3.03807 150960000.000 5.80 3.16 2.92 6.03 5.57

14103000.000 3.03815 151030000.000 5.80 3.16 2.92 6.03 5.57

14110000.000 3.03822 151100000.000 5.80 3.16 2.92 6.03 5.57

14117000.000 3.03830 151170000.000 5.80 3.16 2.92 6.03 5.57

14124000.000 3.03837 151240000.000 5.80 3.16 2.92 6.03 5.57

14131000.000 3.03845 151310000.000 5.80 3.16 2.92 6.03 5.57

14138000.000 3.03852 151380000.000 5.80 3.16 2.92 6.03 5.57

14145000.000 3.03860 151450000.000 5.80 3.16 2.92 6.03 5.57

14152000.000 3.03867 151520000.000 5.80 3.16 2.92 6.03 5.57

14159000.000 3.03875 151590000.000 5.80 3.16 2.92 6.03 5.57

14166000.000 3.03882 151660000.000 5.80 3.16 2.92 6.03 5.57

14173000.000 3.03890 151730000.000 5.80 3.16 2.92 6.03 5.57

14180000.000 3.03898 151800000.000 5.80 3.16 2.92 6.03 5.57

14187000.000 3.03905 151870000.000 5.80 3.16 2.92 6.03 5.57

14194000.000 3.03913 151940000.000 5.80 3.16 2.92 6.03 5.57

14201000.000 3.03920 152010000.000 5.80 3.16 2.92 6.04 5.57

14208000.000 3.03928 152080000.000 5.80 3.16 2.92 6.04 5.57

14215000.000 3.03935 152150000.000 5.80 3.16 2.92 6.04 5.57

14222000.000 3.03943 152220000.000 5.80 3.16 2.92 6.04 5.57

14229000.000 3.03950 152290000.000 5.80 3.16 2.92 6.04 5.57

14236000.000 3.03958 152360000.000 5.80 3.16 2.92 6.04 5.57

14243000.000 3.03965 152430000.000 5.80 3.16 2.92 6.04 5.57

14250000.000 3.03973 152500000.000 5.80 3.16 2.92 6.04 5.57

14257000.000 3.03980 152570000.000 5.80 3.16 2.92 6.04 5.57

14264000.000 3.03988 152640000.000 5.80 3.16 2.92 6.04 5.57

14271000.000 3.03995 152710000.000 5.80 3.16 2.92 6.04 5.57

14278000.000 3.04003 152780000.000 5.80 3.16 2.92 6.04 5.57

14285000.000 3.04010 152850000.000 5.80 3.16 2.92 6.04 5.57

14292000.000 3.04017 152920000.000 5.80 3.16 2.92 6.04 5.57

14299000.000 3.04025 152990000.000 5.80 3.16 2.92 6.04 5.57

14306000.000 3.04032 153060000.000 5.80 3.16 2.92 6.04 5.57

14313000.000 3.04040 153130000.000 5.81 3.16 2.92 6.04 5.57

14320000.000 3.04047 153200000.000 5.81 3.16 2.92 6.04 5.57

14327000.000 3.04055 153270000.000 5.81 3.16 2.92 6.04 5.57

14334000.000 3.04062 153340000.000 5.81 3.16 2.92 6.04 5.57

14341000.000 3.04070 153410000.000 5.81 3.16 2.92 6.04 5.57

14348000.000 3.04077 153480000.000 5.81 3.16 2.92 6.04 5.57

14355000.000 3.04085 153550000.000 5.81 3.16 2.92 6.04 5.57

14362000.000 3.04092 153620000.000 5.81 3.16 2.92 6.04 5.57

14369000.000 3.04099 153690000.000 5.81 3.16 2.92 6.04 5.57

14376000.000 3.04107 153760000.000 5.81 3.16 2.92 6.04 5.57

14383000.000 3.04114 153830000.000 5.81 3.16 2.92 6.04 5.57

14390000.000 3.04122 153900000.000 5.81 3.16 2.92 6.04 5.57

14397000.000 3.04129 153970000.000 5.81 3.16 2.92 6.04 5.57

14404000.000 3.04137 154040000.000 5.81 3.16 2.92 6.04 5.57

14411000.000 3.04144 154110000.000 5.81 3.16 2.92 6.04 5.57

14418000.000 3.04151 154180000.000 5.81 3.16 2.92 6.04 5.57

14425000.000 3.04159 154250000.000 5.81 3.16 2.92 6.04 5.57

14432000.000 3.04166 154320000.000 5.81 3.16 2.92 6.04 5.58

14439000.000 3.04174 154390000.000 5.81 3.16 2.92 6.04 5.58

14446000.000 3.04181 154460000.000 5.81 3.16 2.92 6.04 5.58

14453000.000 3.04188 154530000.000 5.81 3.16 2.92 6.04 5.58

14460000.000 3.04196 154600000.000 5.81 3.16 2.92 6.04 5.58

14467000.000 3.04203 154670000.000 5.81 3.16 2.92 6.04 5.58

14474000.000 3.04210 154740000.000 5.81 3.16 2.92 6.04 5.58

14481000.000 3.04218 154810000.000 5.81 3.16 2.92 6.04 5.58

14488000.000 3.04225 154880000.000 5.81 3.16 2.92 6.04 5.58

14495000.000 3.04233 154950000.000 5.81 3.16 2.92 6.04 5.58

14502000.000 3.04240 155020000.000 5.81 3.16 2.92 6.04 5.58

14509000.000 3.04247 155090000.000 5.81 3.16 2.92 6.04 5.58

14516000.000 3.04255 155160000.000 5.81 3.16 2.92 6.04 5.58

14523000.000 3.04262 155230000.000 5.81 3.16 2.92 6.04 5.58

14530000.000 3.04269 155300000.000 5.81 3.16 2.92 6.04 5.58

14537000.000 3.04277 155370000.000 5.81 3.16 2.92 6.04 5.58

14544000.000 3.04284 155440000.000 5.81 3.16 2.92 6.04 5.58

14551000.000 3.04291 155510000.000 5.81 3.16 2.92 6.04 5.58

14558000.000 3.04299 155580000.000 5.81 3.16 2.92 6.04 5.58

14565000.000 3.04306 155650000.000 5.81 3.16 2.92 6.04 5.58

14572000.000 3.04313 155720000.000 5.81 3.16 2.92 6.04 5.58

14579000.000 3.04321 155790000.000 5.81 3.16 2.92 6.04 5.58

14586000.000 3.04328 155860000.000 5.81 3.17 2.92 6.04 5.58

14593000.000 3.04335 155930000.000 5.81 3.17 2.92 6.04 5.58

14600000.000 3.04343 156000000.000 5.81 3.17 2.92 6.04 5.58

14607000.000 3.04350 156070000.000 5.81 3.17 2.92 6.04 5.58

14614000.000 3.04357 156140000.000 5.81 3.17 2.92 6.04 5.58

14621000.000 3.04365 156210000.000 5.81 3.17 2.92 6.04 5.58

14628000.000 3.04372 156280000.000 5.81 3.17 2.92 6.04 5.58

14635000.000 3.04379 156350000.000 5.81 3.17 2.92 6.04 5.58

14642000.000 3.04387 156420000.000 5.81 3.17 2.92 6.04 5.58

14649000.000 3.04394 156490000.000 5.81 3.17 2.92 6.04 5.58

14656000.000 3.04401 156560000.000 5.81 3.17 2.92 6.04 5.58

14663000.000 3.04409 156630000.000 5.81 3.17 2.92 6.04 5.58

14670000.000 3.04416 156700000.000 5.81 3.17 2.92 6.04 5.58

14677000.000 3.04423 156770000.000 5.81 3.17 2.92 6.04 5.58

14684000.000 3.04430 156840000.000 5.81 3.17 2.92 6.04 5.58

14691000.000 3.04438 156910000.000 5.81 3.17 2.92 6.04 5.58

14698000.000 3.04445 156980000.000 5.81 3.17 2.92 6.04 5.58

14705000.000 3.04452 157050000.000 5.81 3.17 2.92 6.04 5.58

14712000.000 3.04459 157120000.000 5.81 3.17 2.92 6.05 5.58

14719000.000 3.04467 157190000.000 5.81 3.17 2.92 6.05 5.58

14726000.000 3.04474 157260000.000 5.81 3.17 2.92 6.05 5.58

14733000.000 3.04481 157330000.000 5.81 3.17 2.92 6.05 5.58

14740000.000 3.04488 157400000.000 5.81 3.17 2.92 6.05 5.58

14747000.000 3.04496 157470000.000 5.81 3.17 2.92 6.05 5.58

14754000.000 3.04503 157540000.000 5.81 3.17 2.92 6.05 5.58

14761000.000 3.04510 157610000.000 5.81 3.17 2.92 6.05 5.58

14768000.000 3.04517 157680000.000 5.81 3.17 2.92 6.05 5.58

14775000.000 3.04525 157750000.000 5.81 3.17 2.92 6.05 5.58

14782000.000 3.04532 157820000.000 5.81 3.17 2.92 6.05 5.58

14789000.000 3.04539 157890000.000 5.81 3.17 2.92 6.05 5.58

14796000.000 3.04546 157960000.000 5.81 3.17 2.92 6.05 5.58

14803000.000 3.04554 158030000.000 5.81 3.17 2.92 6.05 5.58

14810000.000 3.04561 158100000.000 5.81 3.17 2.92 6.05 5.58

14817000.000 3.04568 158170000.000 5.81 3.17 2.92 6.05 5.58

14824000.000 3.04575 158240000.000 5.81 3.17 2.92 6.05 5.58

14831000.000 3.04583 158310000.000 5.81 3.17 2.92 6.05 5.58

14838000.000 3.04590 158380000.000 5.81 3.17 2.92 6.05 5.58

14845000.000 3.04597 158450000.000 5.82 3.17 2.92 6.05 5.58

14852000.000 3.04604 158520000.000 5.82 3.17 2.92 6.05 5.58

14859000.000 3.04611 158590000.000 5.82 3.17 2.92 6.05 5.58

14866000.000 3.04619 158660000.000 5.82 3.17 2.92 6.05 5.58

14873000.000 3.04626 158730000.000 5.82 3.17 2.92 6.05 5.58

14880000.000 3.04633 158800000.000 5.82 3.17 2.92 6.05 5.58

14887000.000 3.04640 158870000.000 5.82 3.17 2.92 6.05 5.58

14894000.000 3.04647 158940000.000 5.82 3.17 2.92 6.05 5.58

14901000.000 3.04654 159010000.000 5.82 3.17 2.92 6.05 5.58

14908000.000 3.04662 159080000.000 5.82 3.17 2.92 6.05 5.58

14915000.000 3.04669 159150000.000 5.82 3.17 2.92 6.05 5.58

14922000.000 3.04676 159220000.000 5.82 3.17 2.92 6.05 5.58

14929000.000 3.04683 159290000.000 5.82 3.17 2.92 6.05 5.58

14936000.000 3.04690 159360000.000 5.82 3.17 2.93 6.05 5.58

14943000.000 3.04697 159430000.000 5.82 3.17 2.93 6.05 5.58

14950000.000 3.04705 159500000.000 5.82 3.17 2.93 6.05 5.58

14957000.000 3.04712 159570000.000 5.82 3.17 2.93 6.05 5.58

14964000.000 3.04719 159640000.000 5.82 3.17 2.93 6.05 5.58

14971000.000 3.04726 159710000.000 5.82 3.17 2.93 6.05 5.58

14978000.000 3.04733 159780000.000 5.82 3.17 2.93 6.05 5.58

14985000.000 3.04740 159850000.000 5.82 3.17 2.93 6.05 5.58

14992000.000 3.04748 159920000.000 5.82 3.17 2.93 6.05 5.59

14999000.000 3.04755 159990000.000 5.82 3.17 2.93 6.05 5.59

15006000.000 3.04762 160060000.000 5.82 3.17 2.93 6.05 5.59

15013000.000 3.04769 160130000.000 5.82 3.17 2.93 6.05 5.59

15020000.000 3.04776 160200000.000 5.82 3.17 2.93 6.05 5.59

15027000.000 3.04783 160270000.000 5.82 3.17 2.93 6.05 5.59

15034000.000 3.04790 160340000.000 5.82 3.17 2.93 6.05 5.59

15041000.000 3.04797 160410000.000 5.82 3.17 2.93 6.05 5.59

15048000.000 3.04805 160480000.000 5.82 3.17 2.93 6.05 5.59

15055000.000 3.04812 160550000.000 5.82 3.17 2.93 6.05 5.59

15062000.000 3.04819 160620000.000 5.82 3.17 2.93 6.05 5.59

15069000.000 3.04826 160690000.000 5.82 3.17 2.93 6.05 5.59

15076000.000 3.04833 160760000.000 5.82 3.17 2.93 6.05 5.59

15083000.000 3.04840 160830000.000 5.82 3.17 2.93 6.05 5.59

15090000.000 3.04847 160900000.000 5.82 3.17 2.93 6.05 5.59

15097000.000 3.04854 160970000.000 5.82 3.17 2.93 6.05 5.59

15104000.000 3.04861 161040000.000 5.82 3.17 2.93 6.05 5.59

15111000.000 3.04868 161110000.000 5.82 3.17 2.93 6.05 5.59

15118000.000 3.04875 161180000.000 5.82 3.17 2.93 6.05 5.59

15125000.000 3.04883 161250000.000 5.82 3.17 2.93 6.05 5.59

15132000.000 3.04890 161320000.000 5.82 3.17 2.93 6.05 5.59

15139000.000 3.04897 161390000.000 5.82 3.17 2.93 6.05 5.59

15146000.000 3.04904 161460000.000 5.82 3.17 2.93 6.05 5.59

15153000.000 3.04911 161530000.000 5.82 3.17 2.93 6.05 5.59

15160000.000 3.04918 161600000.000 5.82 3.17 2.93 6.05 5.59

15167000.000 3.04925 161670000.000 5.82 3.17 2.93 6.05 5.59

15174000.000 3.04932 161740000.000 5.82 3.17 2.93 6.05 5.59

15181000.000 3.04939 161810000.000 5.82 3.17 2.93 6.05 5.59

15188000.000 3.04946 161880000.000 5.82 3.17 2.93 6.05 5.59

15195000.000 3.04953 161950000.000 5.82 3.17 2.93 6.05 5.59

15202000.000 3.04960 162020000.000 5.82 3.17 2.93 6.05 5.59

15209000.000 3.04967 162090000.000 5.82 3.17 2.93 6.05 5.59

15216000.000 3.04974 162160000.000 5.82 3.17 2.93 6.05 5.59

15223000.000 3.04981 162230000.000 5.82 3.17 2.93 6.05 5.59

15230000.000 3.04988 162300000.000 5.82 3.17 2.93 6.05 5.59

15237000.000 3.04995 162370000.000 5.82 3.17 2.93 6.06 5.59

15244000.000 3.05002 162440000.000 5.82 3.17 2.93 6.06 5.59

15251000.000 3.05009 162510000.000 5.82 3.17 2.93 6.06 5.59

15258000.000 3.05016 162580000.000 5.82 3.17 2.93 6.06 5.59

15265000.000 3.05023 162650000.000 5.82 3.17 2.93 6.06 5.59

15272000.000 3.05031 162720000.000 5.82 3.17 2.93 6.06 5.59

15279000.000 3.05038 162790000.000 5.82 3.17 2.93 6.06 5.59

15286000.000 3.05045 162860000.000 5.82 3.17 2.93 6.06 5.59

15293000.000 3.05052 162930000.000 5.82 3.17 2.93 6.06 5.59

15300000.000 3.05059 163000000.000 5.82 3.17 2.93 6.06 5.59

15307000.000 3.05066 163070000.000 5.82 3.17 2.93 6.06 5.59

15314000.000 3.05073 163140000.000 5.82 3.17 2.93 6.06 5.59

15321000.000 3.05080 163210000.000 5.82 3.17 2.93 6.06 5.59

15328000.000 3.05087 163280000.000 5.82 3.17 2.93 6.06 5.59

15335000.000 3.05093 163350000.000 5.82 3.17 2.93 6.06 5.59

15342000.000 3.05100 163420000.000 5.82 3.17 2.93 6.06 5.59

15349000.000 3.05107 163490000.000 5.82 3.17 2.93 6.06 5.59

15356000.000 3.05114 163560000.000 5.82 3.17 2.93 6.06 5.59

15363000.000 3.05121 163630000.000 5.82 3.17 2.93 6.06 5.59

15370000.000 3.05128 163700000.000 5.82 3.17 2.93 6.06 5.59

15377000.000 3.05135 163770000.000 5.82 3.17 2.93 6.06 5.59

15384000.000 3.05142 163840000.000 5.82 3.17 2.93 6.06 5.59

15391000.000 3.05149 163910000.000 5.82 3.17 2.93 6.06 5.59

15398000.000 3.05156 163980000.000 5.83 3.17 2.93 6.06 5.59

15405000.000 3.05163 164050000.000 5.83 3.17 2.93 6.06 5.59

15412000.000 3.05170 164120000.000 5.83 3.17 2.93 6.06 5.59

15419000.000 3.05177 164190000.000 5.83 3.17 2.93 6.06 5.59

15426000.000 3.05184 164260000.000 5.83 3.17 2.93 6.06 5.59

15433000.000 3.05191 164330000.000 5.83 3.17 2.93 6.06 5.59

15440000.000 3.05198 164400000.000 5.83 3.17 2.93 6.06 5.59

15447000.000 3.05205 164470000.000 5.83 3.17 2.93 6.06 5.59

15454000.000 3.05212 164540000.000 5.83 3.17 2.93 6.06 5.59

15461000.000 3.05219 164610000.000 5.83 3.17 2.93 6.06 5.59

15468000.000 3.05226 164680000.000 5.83 3.17 2.93 6.06 5.59

15475000.000 3.05233 164750000.000 5.83 3.17 2.93 6.06 5.59

15482000.000 3.05240 164820000.000 5.83 3.17 2.93 6.06 5.59

15489000.000 3.05246 164890000.000 5.83 3.17 2.93 6.06 5.59

15496000.000 3.05253 164960000.000 5.83 3.17 2.93 6.06 5.59

15503000.000 3.05260 165030000.000 5.83 3.17 2.93 6.06 5.59

15510000.000 3.05267 165100000.000 5.83 3.17 2.93 6.06 5.59

15517000.000 3.05274 165170000.000 5.83 3.17 2.93 6.06 5.59

15524000.000 3.05281 165240000.000 5.83 3.17 2.93 6.06 5.59

15531000.000 3.05288 165310000.000 5.83 3.17 2.93 6.06 5.59

15538000.000 3.05295 165380000.000 5.83 3.18 2.93 6.06 5.59

15545000.000 3.05302 165450000.000 5.83 3.18 2.93 6.06 5.59

15552000.000 3.05309 165520000.000 5.83 3.18 2.93 6.06 5.59

15559000.000 3.05315 165590000.000 5.83 3.18 2.93 6.06 5.59

15566000.000 3.05322 165660000.000 5.83 3.18 2.93 6.06 5.59

15573000.000 3.05329 165730000.000 5.83 3.18 2.93 6.06 5.60

15580000.000 3.05336 165800000.000 5.83 3.18 2.93 6.06 5.60

15587000.000 3.05343 165870000.000 5.83 3.18 2.93 6.06 5.60

15594000.000 3.05350 165940000.000 5.83 3.18 2.93 6.06 5.60

15601000.000 3.05357 166010000.000 5.83 3.18 2.93 6.06 5.60

15608000.000 3.05364 166080000.000 5.83 3.18 2.93 6.06 5.60

15615000.000 3.05371 166150000.000 5.83 3.18 2.93 6.06 5.60

15622000.000 3.05377 166220000.000 5.83 3.18 2.93 6.06 5.60

15629000.000 3.05384 166290000.000 5.83 3.18 2.93 6.06 5.60

15636000.000 3.05391 166360000.000 5.83 3.18 2.93 6.06 5.60

15643000.000 3.05398 166430000.000 5.83 3.18 2.93 6.06 5.60

15650000.000 3.05405 166500000.000 5.83 3.18 2.93 6.06 5.60

15657000.000 3.05412 166570000.000 5.83 3.18 2.93 6.06 5.60

15664000.000 3.05419 166640000.000 5.83 3.18 2.93 6.06 5.60

15671000.000 3.05425 166710000.000 5.83 3.18 2.93 6.06 5.60

15678000.000 3.05432 166780000.000 5.83 3.18 2.93 6.06 5.60

15685000.000 3.05439 166850000.000 5.83 3.18 2.93 6.06 5.60

15692000.000 3.05446 166920000.000 5.83 3.18 2.93 6.06 5.60

15699000.000 3.05453 166990000.000 5.83 3.18 2.93 6.06 5.60

15706000.000 3.05460 167060000.000 5.83 3.18 2.93 6.06 5.60

15713000.000 3.05466 167130000.000 5.83 3.18 2.93 6.06 5.60

15720000.000 3.05473 167200000.000 5.83 3.18 2.93 6.06 5.60

15727000.000 3.05480 167270000.000 5.83 3.18 2.93 6.06 5.60

15734000.000 3.05487 167340000.000 5.83 3.18 2.93 6.06 5.60

15741000.000 3.05494 167410000.000 5.83 3.18 2.93 6.06 5.60

15748000.000 3.05500 167480000.000 5.83 3.18 2.93 6.06 5.60

15755000.000 3.05507 167550000.000 5.83 3.18 2.93 6.06 5.60

15762000.000 3.05514 167620000.000 5.83 3.18 2.93 6.06 5.60

15769000.000 3.05521 167690000.000 5.83 3.18 2.93 6.06 5.60

15776000.000 3.05528 167760000.000 5.83 3.18 2.93 6.07 5.60

15783000.000 3.05534 167830000.000 5.83 3.18 2.93 6.07 5.60

15790000.000 3.05541 167900000.000 5.83 3.18 2.93 6.07 5.60

15797000.000 3.05548 167970000.000 5.83 3.18 2.93 6.07 5.60

15804000.000 3.05555 168040000.000 5.83 3.18 2.93 6.07 5.60

15811000.000 3.05562 168110000.000 5.83 3.18 2.93 6.07 5.60

15818000.000 3.05568 168180000.000 5.83 3.18 2.93 6.07 5.60

15825000.000 3.05575 168250000.000 5.83 3.18 2.93 6.07 5.60

15832000.000 3.05582 168320000.000 5.83 3.18 2.93 6.07 5.60

15839000.000 3.05589 168390000.000 5.83 3.18 2.93 6.07 5.60

15846000.000 3.05596 168460000.000 5.83 3.18 2.93 6.07 5.60

15853000.000 3.05602 168530000.000 5.83 3.18 2.93 6.07 5.60

15860000.000 3.05609 168600000.000 5.83 3.18 2.93 6.07 5.60

15867000.000 3.05616 168670000.000 5.83 3.18 2.93 6.07 5.60

15874000.000 3.05623 168740000.000 5.83 3.18 2.93 6.07 5.60

15881000.000 3.05629 168810000.000 5.83 3.18 2.93 6.07 5.60

15888000.000 3.05636 168880000.000 5.83 3.18 2.93 6.07 5.60

15895000.000 3.05643 168950000.000 5.83 3.18 2.93 6.07 5.60

15902000.000 3.05650 169020000.000 5.83 3.18 2.93 6.07 5.60

15909000.000 3.05656 169090000.000 5.83 3.18 2.93 6.07 5.60

15916000.000 3.05663 169160000.000 5.83 3.18 2.93 6.07 5.60

15923000.000 3.05670 169230000.000 5.83 3.18 2.93 6.07 5.60

15930000.000 3.05677 169300000.000 5.83 3.18 2.93 6.07 5.60

15937000.000 3.05683 169370000.000 5.83 3.18 2.93 6.07 5.60

15944000.000 3.05690 169440000.000 5.83 3.18 2.93 6.07 5.60

15951000.000 3.05697 169510000.000 5.83 3.18 2.93 6.07 5.60

15958000.000 3.05704 169580000.000 5.83 3.18 2.93 6.07 5.60

15965000.000 3.05710 169650000.000 5.84 3.18 2.93 6.07 5.60

15972000.000 3.05717 169720000.000 5.84 3.18 2.93 6.07 5.60

15979000.000 3.05724 169790000.000 5.84 3.18 2.93 6.07 5.60

15986000.000 3.05730 169860000.000 5.84 3.18 2.94 6.07 5.60

15993000.000 3.05737 169930000.000 5.84 3.18 2.94 6.07 5.60

16000000.000 3.05744 170000000.000 5.84 3.18 2.94 6.07 5.60

16007000.000 3.05751 170070000.000 5.84 3.18 2.94 6.07 5.60

16014000.000 3.05757 170140000.000 5.84 3.18 2.94 6.07 5.60

16021000.000 3.05764 170210000.000 5.84 3.18 2.94 6.07 5.60

16028000.000 3.05771 170280000.000 5.84 3.18 2.94 6.07 5.60

16035000.000 3.05777 170350000.000 5.84 3.18 2.94 6.07 5.60

16042000.000 3.05784 170420000.000 5.84 3.18 2.94 6.07 5.60

16049000.000 3.05791 170490000.000 5.84 3.18 2.94 6.07 5.60

16056000.000 3.05797 170560000.000 5.84 3.18 2.94 6.07 5.60

16063000.000 3.05804 170630000.000 5.84 3.18 2.94 6.07 5.60

16070000.000 3.05811 170700000.000 5.84 3.18 2.94 6.07 5.60

16077000.000 3.05817 170770000.000 5.84 3.18 2.94 6.07 5.60

16084000.000 3.05824 170840000.000 5.84 3.18 2.94 6.07 5.60

16091000.000 3.05831 170910000.000 5.84 3.18 2.94 6.07 5.60

16098000.000 3.05837 170980000.000 5.84 3.18 2.94 6.07 5.60

16105000.000 3.05844 171050000.000 5.84 3.18 2.94 6.07 5.60

16112000.000 3.05851 171120000.000 5.84 3.18 2.94 6.07 5.60

16119000.000 3.05857 171190000.000 5.84 3.18 2.94 6.07 5.60

16126000.000 3.05864 171260000.000 5.84 3.18 2.94 6.07 5.60

16133000.000 3.05871 171330000.000 5.84 3.18 2.94 6.07 5.60

16140000.000 3.05877 171400000.000 5.84 3.18 2.94 6.07 5.60

16147000.000 3.05884 171470000.000 5.84 3.18 2.94 6.07 5.60

16154000.000 3.05891 171540000.000 5.84 3.18 2.94 6.07 5.60

16161000.000 3.05897 171610000.000 5.84 3.18 2.94 6.07 5.60

16168000.000 3.05904 171680000.000 5.84 3.18 2.94 6.07 5.60

16175000.000 3.05911 171750000.000 5.84 3.18 2.94 6.07 5.61

16182000.000 3.05917 171820000.000 5.84 3.18 2.94 6.07 5.61

16189000.000 3.05924 171890000.000 5.84 3.18 2.94 6.07 5.61

16196000.000 3.05931 171960000.000 5.84 3.18 2.94 6.07 5.61

16203000.000 3.05937 172030000.000 5.84 3.18 2.94 6.07 5.61

16210000.000 3.05944 172100000.000 5.84 3.18 2.94 6.07 5.61

16217000.000 3.05951 172170000.000 5.84 3.18 2.94 6.07 5.61

16224000.000 3.05957 172240000.000 5.84 3.18 2.94 6.07 5.61

16231000.000 3.05964 172310000.000 5.84 3.18 2.94 6.07 5.61

16238000.000 3.05970 172380000.000 5.84 3.18 2.94 6.07 5.61

16245000.000 3.05977 172450000.000 5.84 3.18 2.94 6.07 5.61

16252000.000 3.05984 172520000.000 5.84 3.18 2.94 6.07 5.61

16259000.000 3.05990 172590000.000 5.84 3.18 2.94 6.07 5.61

16266000.000 3.05997 172660000.000 5.84 3.18 2.94 6.07 5.61

16273000.000 3.06003 172730000.000 5.84 3.18 2.94 6.07 5.61

16280000.000 3.06010 172800000.000 5.84 3.18 2.94 6.07 5.61

16287000.000 3.06017 172870000.000 5.84 3.18 2.94 6.07 5.61

16294000.000 3.06023 172940000.000 5.84 3.18 2.94 6.07 5.61

16301000.000 3.06030 173010000.000 5.84 3.18 2.94 6.07 5.61

16308000.000 3.06036 173080000.000 5.84 3.18 2.94 6.07 5.61

16315000.000 3.06043 173150000.000 5.84 3.18 2.94 6.07 5.61

16322000.000 3.06050 173220000.000 5.84 3.18 2.94 6.07 5.61

16329000.000 3.06056 173290000.000 5.84 3.18 2.94 6.07 5.61

16336000.000 3.06063 173360000.000 5.84 3.18 2.94 6.08 5.61

16343000.000 3.06069 173430000.000 5.84 3.18 2.94 6.08 5.61

16350000.000 3.06076 173500000.000 5.84 3.18 2.94 6.08 5.61

16357000.000 3.06082 173570000.000 5.84 3.18 2.94 6.08 5.61

16364000.000 3.06089 173640000.000 5.84 3.18 2.94 6.08 5.61

16371000.000 3.06096 173710000.000 5.84 3.18 2.94 6.08 5.61

16378000.000 3.06102 173780000.000 5.84 3.18 2.94 6.08 5.61

16385000.000 3.06109 173850000.000 5.84 3.18 2.94 6.08 5.61

16392000.000 3.06115 173920000.000 5.84 3.18 2.94 6.08 5.61

16399000.000 3.06122 173990000.000 5.84 3.18 2.94 6.08 5.61

16406000.000 3.06128 174060000.000 5.84 3.18 2.94 6.08 5.61

16413000.000 3.06135 174130000.000 5.84 3.18 2.94 6.08 5.61

16420000.000 3.06141 174200000.000 5.84 3.18 2.94 6.08 5.61

16427000.000 3.06148 174270000.000 5.84 3.18 2.94 6.08 5.61

16434000.000 3.06155 174340000.000 5.84 3.18 2.94 6.08 5.61

16441000.000 3.06161 174410000.000 5.84 3.18 2.94 6.08 5.61

16448000.000 3.06168 174480000.000 5.84 3.18 2.94 6.08 5.61

16455000.000 3.06174 174550000.000 5.84 3.18 2.94 6.08 5.61

16462000.000 3.06181 174620000.000 5.84 3.18 2.94 6.08 5.61

16469000.000 3.06187 174690000.000 5.84 3.18 2.94 6.08 5.61

16476000.000 3.06194 174760000.000 5.84 3.18 2.94 6.08 5.61

16483000.000 3.06200 174830000.000 5.84 3.18 2.94 6.08 5.61

16490000.000 3.06207 174900000.000 5.84 3.18 2.94 6.08 5.61

16497000.000 3.06213 174970000.000 5.84 3.18 2.94 6.08 5.61

16504000.000 3.06220 175040000.000 5.84 3.18 2.94 6.08 5.61

16511000.000 3.06226 175110000.000 5.84 3.18 2.94 6.08 5.61

16518000.000 3.06233 175180000.000 5.84 3.18 2.94 6.08 5.61

16525000.000 3.06239 175250000.000 5.84 3.18 2.94 6.08 5.61

16532000.000 3.06246 175320000.000 5.84 3.18 2.94 6.08 5.61

16539000.000 3.06252 175390000.000 5.84 3.19 2.94 6.08 5.61

16546000.000 3.06259 175460000.000 5.84 3.19 2.94 6.08 5.61

16553000.000 3.06265 175530000.000 5.84 3.19 2.94 6.08 5.61

16560000.000 3.06272 175600000.000 5.85 3.19 2.94 6.08 5.61

16567000.000 3.06278 175670000.000 5.85 3.19 2.94 6.08 5.61

16574000.000 3.06285 175740000.000 5.85 3.19 2.94 6.08 5.61

16581000.000 3.06291 175810000.000 5.85 3.19 2.94 6.08 5.61

16588000.000 3.06298 175880000.000 5.85 3.19 2.94 6.08 5.61

16595000.000 3.06304 175950000.000 5.85 3.19 2.94 6.08 5.61

16602000.000 3.06311 176020000.000 5.85 3.19 2.94 6.08 5.61

16609000.000 3.06317 176090000.000 5.85 3.19 2.94 6.08 5.61

16616000.000 3.06324 176160000.000 5.85 3.19 2.94 6.08 5.61

16623000.000 3.06330 176230000.000 5.85 3.19 2.94 6.08 5.61

16630000.000 3.06337 176300000.000 5.85 3.19 2.94 6.08 5.61

16637000.000 3.06343 176370000.000 5.85 3.19 2.94 6.08 5.61

16644000.000 3.06350 176440000.000 5.85 3.19 2.94 6.08 5.61

16651000.000 3.06356 176510000.000 5.85 3.19 2.94 6.08 5.61

16658000.000 3.06363 176580000.000 5.85 3.19 2.94 6.08 5.61

16665000.000 3.06369 176650000.000 5.85 3.19 2.94 6.08 5.61

16672000.000 3.06375 176720000.000 5.85 3.19 2.94 6.08 5.61

16679000.000 3.06382 176790000.000 5.85 3.19 2.94 6.08 5.61

16686000.000 3.06388 176860000.000 5.85 3.19 2.94 6.08 5.61

16693000.000 3.06395 176930000.000 5.85 3.19 2.94 6.08 5.61

16700000.000 3.06401 177000000.000 5.85 3.19 2.94 6.08 5.61

16707000.000 3.06408 177070000.000 5.85 3.19 2.94 6.08 5.61

16714000.000 3.06414 177140000.000 5.85 3.19 2.94 6.08 5.61

16721000.000 3.06421 177210000.000 5.85 3.19 2.94 6.08 5.61

16728000.000 3.06427 177280000.000 5.85 3.19 2.94 6.08 5.61

16735000.000 3.06433 177350000.000 5.85 3.19 2.94 6.08 5.61

16742000.000 3.06440 177420000.000 5.85 3.19 2.94 6.08 5.61

16749000.000 3.06446 177490000.000 5.85 3.19 2.94 6.08 5.61

16756000.000 3.06453 177560000.000 5.85 3.19 2.94 6.08 5.61

16763000.000 3.06459 177630000.000 5.85 3.19 2.94 6.08 5.61

16770000.000 3.06466 177700000.000 5.85 3.19 2.94 6.08 5.61

16777000.000 3.06472 177770000.000 5.85 3.19 2.94 6.08 5.61

16784000.000 3.06478 177840000.000 5.85 3.19 2.94 6.08 5.61

16791000.000 3.06485 177910000.000 5.85 3.19 2.94 6.08 5.61

16798000.000 3.06491 177980000.000 5.85 3.19 2.94 6.08 5.62

16805000.000 3.06498 178050000.000 5.85 3.19 2.94 6.08 5.62

16812000.000 3.06504 178120000.000 5.85 3.19 2.94 6.08 5.62

16819000.000 3.06510 178190000.000 5.85 3.19 2.94 6.08 5.62

16826000.000 3.06517 178260000.000 5.85 3.19 2.94 6.08 5.62

16833000.000 3.06523 178330000.000 5.85 3.19 2.94 6.08 5.62

16840000.000 3.06530 178400000.000 5.85 3.19 2.94 6.08 5.62

16847000.000 3.06536 178470000.000 5.85 3.19 2.94 6.08 5.62

16854000.000 3.06542 178540000.000 5.85 3.19 2.94 6.08 5.62

16861000.000 3.06549 178610000.000 5.85 3.19 2.94 6.08 5.62

16868000.000 3.06555 178680000.000 5.85 3.19 2.94 6.08 5.62

16875000.000 3.06561 178750000.000 5.85 3.19 2.94 6.08 5.62

16882000.000 3.06568 178820000.000 5.85 3.19 2.94 6.08 5.62

16889000.000 3.06574 178890000.000 5.85 3.19 2.94 6.08 5.62

16896000.000 3.06581 178960000.000 5.85 3.19 2.94 6.08 5.62

16903000.000 3.06587 179030000.000 5.85 3.19 2.94 6.08 5.62

16910000.000 3.06593 179100000.000 5.85 3.19 2.94 6.08 5.62

16917000.000 3.06600 179170000.000 5.85 3.19 2.94 6.09 5.62

16924000.000 3.06606 179240000.000 5.85 3.19 2.94 6.09 5.62

16931000.000 3.06612 179310000.000 5.85 3.19 2.94 6.09 5.62

16938000.000 3.06619 179380000.000 5.85 3.19 2.94 6.09 5.62

16945000.000 3.06625 179450000.000 5.85 3.19 2.94 6.09 5.62

16952000.000 3.06632 179520000.000 5.85 3.19 2.94 6.09 5.62

16959000.000 3.06638 179590000.000 5.85 3.19 2.94 6.09 5.62

16966000.000 3.06644 179660000.000 5.85 3.19 2.94 6.09 5.62

16973000.000 3.06651 179730000.000 5.85 3.19 2.94 6.09 5.62

16980000.000 3.06657 179800000.000 5.85 3.19 2.94 6.09 5.62

16987000.000 3.06663 179870000.000 5.85 3.19 2.94 6.09 5.62

16994000.000 3.06670 179940000.000 5.85 3.19 2.94 6.09 5.62

17001000.000 3.06676 180010000.000 5.85 3.19 2.94 6.09 5.62

17008000.000 3.06682 180080000.000 5.85 3.19 2.94 6.09 5.62

17015000.000 3.06689 180150000.000 5.85 3.19 2.94 6.09 5.62

17022000.000 3.06695 180220000.000 5.85 3.19 2.94 6.09 5.62

17029000.000 3.06701 180290000.000 5.85 3.19 2.94 6.09 5.62

17036000.000 3.06708 180360000.000 5.85 3.19 2.94 6.09 5.62

17043000.000 3.06714 180430000.000 5.85 3.19 2.94 6.09 5.62

17050000.000 3.06720 180500000.000 5.85 3.19 2.94 6.09 5.62

17057000.000 3.06726 180570000.000 5.85 3.19 2.94 6.09 5.62

17064000.000 3.06733 180640000.000 5.85 3.19 2.94 6.09 5.62

17071000.000 3.06739 180710000.000 5.85 3.19 2.94 6.09 5.62

17078000.000 3.06745 180780000.000 5.85 3.19 2.94 6.09 5.62

17085000.000 3.06752 180850000.000 5.85 3.19 2.94 6.09 5.62

17092000.000 3.06758 180920000.000 5.85 3.19 2.94 6.09 5.62

17099000.000 3.06764 180990000.000 5.85 3.19 2.94 6.09 5.62

17106000.000 3.06771 181060000.000 5.85 3.19 2.94 6.09 5.62

17113000.000 3.06777 181130000.000 5.85 3.19 2.95 6.09 5.62

17120000.000 3.06783 181200000.000 5.85 3.19 2.95 6.09 5.62

17127000.000 3.06790 181270000.000 5.85 3.19 2.95 6.09 5.62

17134000.000 3.06796 181340000.000 5.85 3.19 2.95 6.09 5.62

17141000.000 3.06802 181410000.000 5.85 3.19 2.95 6.09 5.62

17148000.000 3.06808 181480000.000 5.85 3.19 2.95 6.09 5.62

17155000.000 3.06815 181550000.000 5.85 3.19 2.95 6.09 5.62

17162000.000 3.06821 181620000.000 5.86 3.19 2.95 6.09 5.62

17169000.000 3.06827 181690000.000 5.86 3.19 2.95 6.09 5.62

17176000.000 3.06833 181760000.000 5.86 3.19 2.95 6.09 5.62

17183000.000 3.06840 181830000.000 5.86 3.19 2.95 6.09 5.62

17190000.000 3.06846 181900000.000 5.86 3.19 2.95 6.09 5.62

17197000.000 3.06852 181970000.000 5.86 3.19 2.95 6.09 5.62

17204000.000 3.06859 182040000.000 5.86 3.19 2.95 6.09 5.62

17211000.000 3.06865 182110000.000 5.86 3.19 2.95 6.09 5.62

17218000.000 3.06871 182180000.000 5.86 3.19 2.95 6.09 5.62

17225000.000 3.06877 182250000.000 5.86 3.19 2.95 6.09 5.62

17232000.000 3.06884 182320000.000 5.86 3.19 2.95 6.09 5.62

17239000.000 3.06890 182390000.000 5.86 3.19 2.95 6.09 5.62

17246000.000 3.06896 182460000.000 5.86 3.19 2.95 6.09 5.62

17253000.000 3.06902 182530000.000 5.86 3.19 2.95 6.09 5.62

17260000.000 3.06909 182600000.000 5.86 3.19 2.95 6.09 5.62

17267000.000 3.06915 182670000.000 5.86 3.19 2.95 6.09 5.62

17274000.000 3.06921 182740000.000 5.86 3.19 2.95 6.09 5.62

17281000.000 3.06927 182810000.000 5.86 3.19 2.95 6.09 5.62

17288000.000 3.06934 182880000.000 5.86 3.19 2.95 6.09 5.62

17295000.000 3.06940 182950000.000 5.86 3.19 2.95 6.09 5.62

17302000.000 3.06946 183020000.000 5.86 3.19 2.95 6.09 5.62

17309000.000 3.06952 183090000.000 5.86 3.19 2.95 6.09 5.62

17316000.000 3.06958 183160000.000 5.86 3.19 2.95 6.09 5.62

17323000.000 3.06965 183230000.000 5.86 3.19 2.95 6.09 5.62

17330000.000 3.06971 183300000.000 5.86 3.19 2.95 6.09 5.62

17337000.000 3.06977 183370000.000 5.86 3.19 2.95 6.09 5.62

17344000.000 3.06983 183440000.000 5.86 3.19 2.95 6.09 5.62

17351000.000 3.06990 183510000.000 5.86 3.19 2.95 6.09 5.62

17358000.000 3.06996 183580000.000 5.86 3.19 2.95 6.09 5.62

17365000.000 3.07002 183650000.000 5.86 3.19 2.95 6.09 5.62

17372000.000 3.07008 183720000.000 5.86 3.19 2.95 6.09 5.62

17379000.000 3.07014 183790000.000 5.86 3.19 2.95 6.09 5.62

17386000.000 3.07021 183860000.000 5.86 3.19 2.95 6.09 5.62

17393000.000 3.07027 183930000.000 5.86 3.19 2.95 6.09 5.62

17400000.000 3.07033 184000000.000 5.86 3.19 2.95 6.09 5.62

17407000.000 3.07039 184070000.000 5.86 3.19 2.95 6.09 5.62

17414000.000 3.07045 184140000.000 5.86 3.19 2.95 6.09 5.62

17421000.000 3.07052 184210000.000 5.86 3.19 2.95 6.09 5.62

17428000.000 3.07058 184280000.000 5.86 3.19 2.95 6.09 5.62

17435000.000 3.07064 184350000.000 5.86 3.19 2.95 6.09 5.63

17442000.000 3.07070 184420000.000 5.86 3.19 2.95 6.09 5.63

17449000.000 3.07076 184490000.000 5.86 3.19 2.95 6.09 5.63

17456000.000 3.07082 184560000.000 5.86 3.19 2.95 6.09 5.63

17463000.000 3.07089 184630000.000 5.86 3.19 2.95 6.09 5.63

17470000.000 3.07095 184700000.000 5.86 3.19 2.95 6.09 5.63

17477000.000 3.07101 184770000.000 5.86 3.19 2.95 6.09 5.63

17484000.000 3.07107 184840000.000 5.86 3.19 2.95 6.09 5.63

17491000.000 3.07113 184910000.000 5.86 3.19 2.95 6.09 5.63

17498000.000 3.07119 184980000.000 5.86 3.19 2.95 6.09 5.63

17505000.000 3.07126 185050000.000 5.86 3.19 2.95 6.09 5.63

17512000.000 3.07132 185120000.000 5.86 3.19 2.95 6.10 5.63

17519000.000 3.07138 185190000.000 5.86 3.19 2.95 6.10 5.63

17526000.000 3.07144 185260000.000 5.86 3.19 2.95 6.10 5.63

17533000.000 3.07150 185330000.000 5.86 3.19 2.95 6.10 5.63

17540000.000 3.07156 185400000.000 5.86 3.19 2.95 6.10 5.63

17547000.000 3.07163 185470000.000 5.86 3.19 2.95 6.10 5.63

17554000.000 3.07169 185540000.000 5.86 3.19 2.95 6.10 5.63

17561000.000 3.07175 185610000.000 5.86 3.19 2.95 6.10 5.63

17568000.000 3.07181 185680000.000 5.86 3.19 2.95 6.10 5.63

17575000.000 3.07187 185750000.000 5.86 3.19 2.95 6.10 5.63

17582000.000 3.07193 185820000.000 5.86 3.19 2.95 6.10 5.63

17589000.000 3.07199 185890000.000 5.86 3.19 2.95 6.10 5.63

17596000.000 3.07206 185960000.000 5.86 3.19 2.95 6.10 5.63

17603000.000 3.07212 186030000.000 5.86 3.20 2.95 6.10 5.63

17610000.000 3.07218 186100000.000 5.86 3.20 2.95 6.10 5.63

17617000.000 3.07224 186170000.000 5.86 3.20 2.95 6.10 5.63

17624000.000 3.07230 186240000.000 5.86 3.20 2.95 6.10 5.63

17631000.000 3.07236 186310000.000 5.86 3.20 2.95 6.10 5.63

17638000.000 3.07242 186380000.000 5.86 3.20 2.95 6.10 5.63

17645000.000 3.07248 186450000.000 5.86 3.20 2.95 6.10 5.63

17652000.000 3.07254 186520000.000 5.86 3.20 2.95 6.10 5.63

17659000.000 3.07261 186590000.000 5.86 3.20 2.95 6.10 5.63

17666000.000 3.07267 186660000.000 5.86 3.20 2.95 6.10 5.63

17673000.000 3.07273 186730000.000 5.86 3.20 2.95 6.10 5.63

17680000.000 3.07279 186800000.000 5.86 3.20 2.95 6.10 5.63

17687000.000 3.07285 186870000.000 5.86 3.20 2.95 6.10 5.63

17694000.000 3.07291 186940000.000 5.86 3.20 2.95 6.10 5.63

17701000.000 3.07297 187010000.000 5.86 3.20 2.95 6.10 5.63

17708000.000 3.07303 187080000.000 5.86 3.20 2.95 6.10 5.63

17715000.000 3.07309 187150000.000 5.86 3.20 2.95 6.10 5.63

17722000.000 3.07315 187220000.000 5.86 3.20 2.95 6.10 5.63

17729000.000 3.07322 187290000.000 5.86 3.20 2.95 6.10 5.63

17736000.000 3.07328 187360000.000 5.86 3.20 2.95 6.10 5.63

17743000.000 3.07334 187430000.000 5.86 3.20 2.95 6.10 5.63

17750000.000 3.07340 187500000.000 5.86 3.20 2.95 6.10 5.63

17757000.000 3.07346 187570000.000 5.86 3.20 2.95 6.10 5.63

17764000.000 3.07352 187640000.000 5.86 3.20 2.95 6.10 5.63

17771000.000 3.07358 187710000.000 5.86 3.20 2.95 6.10 5.63

17778000.000 3.07364 187780000.000 5.86 3.20 2.95 6.10 5.63

17785000.000 3.07370 187850000.000 5.86 3.20 2.95 6.10 5.63

17792000.000 3.07376 187920000.000 5.87 3.20 2.95 6.10 5.63

17799000.000 3.07382 187990000.000 5.87 3.20 2.95 6.10 5.63

17806000.000 3.07388 188060000.000 5.87 3.20 2.95 6.10 5.63

17813000.000 3.07394 188130000.000 5.87 3.20 2.95 6.10 5.63

17820000.000 3.07401 188200000.000 5.87 3.20 2.95 6.10 5.63

17827000.000 3.07407 188270000.000 5.87 3.20 2.95 6.10 5.63

17834000.000 3.07413 188340000.000 5.87 3.20 2.95 6.10 5.63

17841000.000 3.07419 188410000.000 5.87 3.20 2.95 6.10 5.63

17848000.000 3.07425 188480000.000 5.87 3.20 2.95 6.10 5.63

17855000.000 3.07431 188550000.000 5.87 3.20 2.95 6.10 5.63

17862000.000 3.07437 188620000.000 5.87 3.20 2.95 6.10 5.63

17869000.000 3.07443 188690000.000 5.87 3.20 2.95 6.10 5.63

17876000.000 3.07449 188760000.000 5.87 3.20 2.95 6.10 5.63

17883000.000 3.07455 188830000.000 5.87 3.20 2.95 6.10 5.63

17890000.000 3.07461 188900000.000 5.87 3.20 2.95 6.10 5.63

17897000.000 3.07467 188970000.000 5.87 3.20 2.95 6.10 5.63

17904000.000 3.07473 189040000.000 5.87 3.20 2.95 6.10 5.63

17911000.000 3.07479 189110000.000 5.87 3.20 2.95 6.10 5.63

17918000.000 3.07485 189180000.000 5.87 3.20 2.95 6.10 5.63

17925000.000 3.07491 189250000.000 5.87 3.20 2.95 6.10 5.63

17932000.000 3.07497 189320000.000 5.87 3.20 2.95 6.10 5.63

17939000.000 3.07503 189390000.000 5.87 3.20 2.95 6.10 5.63

17946000.000 3.07509 189460000.000 5.87 3.20 2.95 6.10 5.63

17953000.000 3.07515 189530000.000 5.87 3.20 2.95 6.10 5.63

17960000.000 3.07521 189600000.000 5.87 3.20 2.95 6.10 5.63

17967000.000 3.07527 189670000.000 5.87 3.20 2.95 6.10 5.63

17974000.000 3.07533 189740000.000 5.87 3.20 2.95 6.10 5.63

17981000.000 3.07539 189810000.000 5.87 3.20 2.95 6.10 5.63

17988000.000 3.07545 189880000.000 5.87 3.20 2.95 6.10 5.63

17995000.000 3.07551 189950000.000 5.87 3.20 2.95 6.10 5.63

18002000.000 3.07557 190020000.000 5.87 3.20 2.95 6.10 5.63

18009000.000 3.07563 190090000.000 5.87 3.20 2.95 6.10 5.63

18016000.000 3.07569 190160000.000 5.87 3.20 2.95 6.10 5.63

18023000.000 3.07575 190230000.000 5.87 3.20 2.95 6.10 5.63

18030000.000 3.07581 190300000.000 5.87 3.20 2.95 6.10 5.63

18037000.000 3.07587 190370000.000 5.87 3.20 2.95 6.10 5.63

18044000.000 3.07593 190440000.000 5.87 3.20 2.95 6.10 5.63

18051000.000 3.07599 190510000.000 5.87 3.20 2.95 6.10 5.63

18058000.000 3.07605 190580000.000 5.87 3.20 2.95 6.10 5.63

18065000.000 3.07611 190650000.000 5.87 3.20 2.95 6.10 5.63

18072000.000 3.07617 190720000.000 5.87 3.20 2.95 6.10 5.63

18079000.000 3.07623 190790000.000 5.87 3.20 2.95 6.10 5.63

18086000.000 3.07629 190860000.000 5.87 3.20 2.95 6.10 5.63

18093000.000 3.07635 190930000.000 5.87 3.20 2.95 6.10 5.63

18100000.000 3.07641 191000000.000 5.87 3.20 2.95 6.10 5.64

18107000.000 3.07647 191070000.000 5.87 3.20 2.95 6.10 5.64

18114000.000 3.07653 191140000.000 5.87 3.20 2.95 6.10 5.64

18121000.000 3.07659 191210000.000 5.87 3.20 2.95 6.10 5.64

18128000.000 3.07665 191280000.000 5.87 3.20 2.95 6.11 5.64

18135000.000 3.07671 191350000.000 5.87 3.20 2.95 6.11 5.64

18142000.000 3.07677 191420000.000 5.87 3.20 2.95 6.11 5.64

18149000.000 3.07683 191490000.000 5.87 3.20 2.95 6.11 5.64

18156000.000 3.07689 191560000.000 5.87 3.20 2.95 6.11 5.64

18163000.000 3.07695 191630000.000 5.87 3.20 2.95 6.11 5.64

18170000.000 3.07701 191700000.000 5.87 3.20 2.95 6.11 5.64

18177000.000 3.07707 191770000.000 5.87 3.20 2.95 6.11 5.64

18184000.000 3.07712 191840000.000 5.87 3.20 2.95 6.11 5.64

18191000.000 3.07718 191910000.000 5.87 3.20 2.95 6.11 5.64

18198000.000 3.07724 191980000.000 5.87 3.20 2.95 6.11 5.64

18205000.000 3.07730 192050000.000 5.87 3.20 2.95 6.11 5.64

18212000.000 3.07736 192120000.000 5.87 3.20 2.95 6.11 5.64

18219000.000 3.07742 192190000.000 5.87 3.20 2.95 6.11 5.64

18226000.000 3.07748 192260000.000 5.87 3.20 2.95 6.11 5.64

18233000.000 3.07754 192330000.000 5.87 3.20 2.95 6.11 5.64

18240000.000 3.07760 192400000.000 5.87 3.20 2.95 6.11 5.64

18247000.000 3.07766 192470000.000 5.87 3.20 2.95 6.11 5.64

18254000.000 3.07772 192540000.000 5.87 3.20 2.95 6.11 5.64

18261000.000 3.07778 192610000.000 5.87 3.20 2.95 6.11 5.64

18268000.000 3.07784 192680000.000 5.87 3.20 2.95 6.11 5.64

18275000.000 3.07790 192750000.000 5.87 3.20 2.95 6.11 5.64

18282000.000 3.07795 192820000.000 5.87 3.20 2.95 6.11 5.64

18289000.000 3.07801 192890000.000 5.87 3.20 2.95 6.11 5.64

18296000.000 3.07807 192960000.000 5.87 3.20 2.95 6.11 5.64

18303000.000 3.07813 193030000.000 5.87 3.20 2.96 6.11 5.64

18310000.000 3.07819 193100000.000 5.87 3.20 2.96 6.11 5.64

18317000.000 3.07825 193170000.000 5.87 3.20 2.96 6.11 5.64

18324000.000 3.07831 193240000.000 5.87 3.20 2.96 6.11 5.64

18331000.000 3.07837 193310000.000 5.87 3.20 2.96 6.11 5.64

18338000.000 3.07843 193380000.000 5.87 3.20 2.96 6.11 5.64

18345000.000 3.07849 193450000.000 5.87 3.20 2.96 6.11 5.64

18352000.000 3.07854 193520000.000 5.87 3.20 2.96 6.11 5.64

18359000.000 3.07860 193590000.000 5.87 3.20 2.96 6.11 5.64

18366000.000 3.07866 193660000.000 5.87 3.20 2.96 6.11 5.64

18373000.000 3.07872 193730000.000 5.87 3.20 2.96 6.11 5.64

18380000.000 3.07878 193800000.000 5.87 3.20 2.96 6.11 5.64

18387000.000 3.07884 193870000.000 5.87 3.20 2.96 6.11 5.64

18394000.000 3.07890 193940000.000 5.87 3.20 2.96 6.11 5.64

18401000.000 3.07896 194010000.000 5.87 3.20 2.96 6.11 5.64

18408000.000 3.07901 194080000.000 5.87 3.20 2.96 6.11 5.64

18415000.000 3.07907 194150000.000 5.87 3.20 2.96 6.11 5.64

18422000.000 3.07913 194220000.000 5.87 3.20 2.96 6.11 5.64

18429000.000 3.07919 194290000.000 5.87 3.20 2.96 6.11 5.64

18436000.000 3.07925 194360000.000 5.87 3.20 2.96 6.11 5.64

18443000.000 3.07931 194430000.000 5.88 3.20 2.96 6.11 5.64

18450000.000 3.07937 194500000.000 5.88 3.20 2.96 6.11 5.64

18457000.000 3.07943 194570000.000 5.88 3.20 2.96 6.11 5.64

18464000.000 3.07948 194640000.000 5.88 3.20 2.96 6.11 5.64

18471000.000 3.07954 194710000.000 5.88 3.20 2.96 6.11 5.64

18478000.000 3.07960 194780000.000 5.88 3.20 2.96 6.11 5.64

18485000.000 3.07966 194850000.000 5.88 3.20 2.96 6.11 5.64

18492000.000 3.07972 194920000.000 5.88 3.20 2.96 6.11 5.64

18499000.000 3.07978 194990000.000 5.88 3.20 2.96 6.11 5.64

18506000.000 3.07983 195060000.000 5.88 3.20 2.96 6.11 5.64

18513000.000 3.07989 195130000.000 5.88 3.20 2.96 6.11 5.64

18520000.000 3.07995 195200000.000 5.88 3.20 2.96 6.11 5.64

18527000.000 3.08001 195270000.000 5.88 3.20 2.96 6.11 5.64

18534000.000 3.08007 195340000.000 5.88 3.20 2.96 6.11 5.64

18541000.000 3.08013 195410000.000 5.88 3.20 2.96 6.11 5.64

18548000.000 3.08019 195480000.000 5.88 3.20 2.96 6.11 5.64

18555000.000 3.08024 195550000.000 5.88 3.20 2.96 6.11 5.64

18562000.000 3.08030 195620000.000 5.88 3.20 2.96 6.11 5.64

18569000.000 3.08036 195690000.000 5.88 3.20 2.96 6.11 5.64

18576000.000 3.08042 195760000.000 5.88 3.20 2.96 6.11 5.64

18583000.000 3.08048 195830000.000 5.88 3.20 2.96 6.11 5.64

18590000.000 3.08053 195900000.000 5.88 3.20 2.96 6.11 5.64

18597000.000 3.08059 195970000.000 5.88 3.20 2.96 6.11 5.64

18604000.000 3.08065 196040000.000 5.88 3.20 2.96 6.11 5.64

18611000.000 3.08071 196110000.000 5.88 3.20 2.96 6.11 5.64

18618000.000 3.08077 196180000.000 5.88 3.20 2.96 6.11 5.64

18625000.000 3.08083 196250000.000 5.88 3.20 2.96 6.11 5.64

18632000.000 3.08088 196320000.000 5.88 3.20 2.96 6.11 5.64

18639000.000 3.08094 196390000.000 5.88 3.20 2.96 6.11 5.64

18646000.000 3.08100 196460000.000 5.88 3.20 2.96 6.11 5.64

18653000.000 3.08106 196530000.000 5.88 3.20 2.96 6.11 5.64

18660000.000 3.08112 196600000.000 5.88 3.20 2.96 6.11 5.64

18667000.000 3.08117 196670000.000 5.88 3.20 2.96 6.11 5.64

18674000.000 3.08123 196740000.000 5.88 3.20 2.96 6.11 5.64

18681000.000 3.08129 196810000.000 5.88 3.20 2.96 6.11 5.64

18688000.000 3.08135 196880000.000 5.88 3.20 2.96 6.11 5.64

18695000.000 3.08141 196950000.000 5.88 3.20 2.96 6.11 5.64

18702000.000 3.08146 197020000.000 5.88 3.20 2.96 6.11 5.64

18709000.000 3.08152 197090000.000 5.88 3.20 2.96 6.11 5.64

18716000.000 3.08158 197160000.000 5.88 3.20 2.96 6.11 5.64

18723000.000 3.08164 197230000.000 5.88 3.20 2.96 6.11 5.64

18730000.000 3.08169 197300000.000 5.88 3.20 2.96 6.11 5.64

18737000.000 3.08175 197370000.000 5.88 3.21 2.96 6.11 5.64

18744000.000 3.08181 197440000.000 5.88 3.21 2.96 6.11 5.64

18751000.000 3.08187 197510000.000 5.88 3.21 2.96 6.11 5.64

18758000.000 3.08193 197580000.000 5.88 3.21 2.96 6.11 5.64

18765000.000 3.08198 197650000.000 5.88 3.21 2.96 6.12 5.64

18772000.000 3.08204 197720000.000 5.88 3.21 2.96 6.12 5.64

18779000.000 3.08210 197790000.000 5.88 3.21 2.96 6.12 5.64

18786000.000 3.08216 197860000.000 5.88 3.21 2.96 6.12 5.65

18793000.000 3.08221 197930000.000 5.88 3.21 2.96 6.12 5.65

18800000.000 3.08227 198000000.000 5.88 3.21 2.96 6.12 5.65

18807000.000 3.08233 198070000.000 5.88 3.21 2.96 6.12 5.65

18814000.000 3.08239 198140000.000 5.88 3.21 2.96 6.12 5.65

18821000.000 3.08244 198210000.000 5.88 3.21 2.96 6.12 5.65

18828000.000 3.08250 198280000.000 5.88 3.21 2.96 6.12 5.65

18835000.000 3.08256 198350000.000 5.88 3.21 2.96 6.12 5.65

18842000.000 3.08262 198420000.000 5.88 3.21 2.96 6.12 5.65

18849000.000 3.08267 198490000.000 5.88 3.21 2.96 6.12 5.65

18856000.000 3.08273 198560000.000 5.88 3.21 2.96 6.12 5.65

18863000.000 3.08279 198630000.000 5.88 3.21 2.96 6.12 5.65

18870000.000 3.08285 198700000.000 5.88 3.21 2.96 6.12 5.65

18877000.000 3.08290 198770000.000 5.88 3.21 2.96 6.12 5.65

18884000.000 3.08296 198840000.000 5.88 3.21 2.96 6.12 5.65

18891000.000 3.08302 198910000.000 5.88 3.21 2.96 6.12 5.65

18898000.000 3.08307 198980000.000 5.88 3.21 2.96 6.12 5.65

18905000.000 3.08313 199050000.000 5.88 3.21 2.96 6.12 5.65

18912000.000 3.08319 199120000.000 5.88 3.21 2.96 6.12 5.65

18919000.000 3.08325 199190000.000 5.88 3.21 2.96 6.12 5.65

18926000.000 3.08330 199260000.000 5.88 3.21 2.96 6.12 5.65

18933000.000 3.08336 199330000.000 5.88 3.21 2.96 6.12 5.65

18940000.000 3.08342 199400000.000 5.88 3.21 2.96 6.12 5.65

18947000.000 3.08348 199470000.000 5.88 3.21 2.96 6.12 5.65

18954000.000 3.08353 199540000.000 5.88 3.21 2.96 6.12 5.65

18961000.000 3.08359 199610000.000 5.88 3.21 2.96 6.12 5.65

18968000.000 3.08365 199680000.000 5.88 3.21 2.96 6.12 5.65

18975000.000 3.08370 199750000.000 5.88 3.21 2.96 6.12 5.65

18982000.000 3.08376 199820000.000 5.88 3.21 2.96 6.12 5.65

18989000.000 3.08382 199890000.000 5.88 3.21 2.96 6.12 5.65

18996000.000 3.08387 199960000.000 5.88 3.21 2.96 6.12 5.65

19003000.000 3.08393 200030000.000 5.88 3.21 2.96 6.12 5.65

19010000.000 3.08399 200100000.000 5.88 3.21 2.96 6.12 5.65

19017000.000 3.08405 200170000.000 5.88 3.21 2.96 6.12 5.65

19024000.000 3.08410 200240000.000 5.88 3.21 2.96 6.12 5.65

19031000.000 3.08416 200310000.000 5.88 3.21 2.96 6.12 5.65

19038000.000 3.08422 200380000.000 5.88 3.21 2.96 6.12 5.65

19045000.000 3.08427 200450000.000 5.88 3.21 2.96 6.12 5.65

19052000.000 3.08433 200520000.000 5.88 3.21 2.96 6.12 5.65

19059000.000 3.08439 200590000.000 5.88 3.21 2.96 6.12 5.65

19066000.000 3.08444 200660000.000 5.88 3.21 2.96 6.12 5.65

19073000.000 3.08450 200730000.000 5.88 3.21 2.96 6.12 5.65

19080000.000 3.08456 200800000.000 5.88 3.21 2.96 6.12 5.65

19087000.000 3.08461 200870000.000 5.88 3.21 2.96 6.12 5.65

19094000.000 3.08467 200940000.000 5.88 3.21 2.96 6.12 5.65

19101000.000 3.08473 201010000.000 5.88 3.21 2.96 6.12 5.65

19108000.000 3.08478 201080000.000 5.88 3.21 2.96 6.12 5.65

19115000.000 3.08484 201150000.000 5.89 3.21 2.96 6.12 5.65

19122000.000 3.08490 201220000.000 5.89 3.21 2.96 6.12 5.65

19129000.000 3.08495 201290000.000 5.89 3.21 2.96 6.12 5.65

19136000.000 3.08501 201360000.000 5.89 3.21 2.96 6.12 5.65

19143000.000 3.08507 201430000.000 5.89 3.21 2.96 6.12 5.65

19150000.000 3.08512 201500000.000 5.89 3.21 2.96 6.12 5.65

19157000.000 3.08518 201570000.000 5.89 3.21 2.96 6.12 5.65

19164000.000 3.08524 201640000.000 5.89 3.21 2.96 6.12 5.65

19171000.000 3.08529 201710000.000 5.89 3.21 2.96 6.12 5.65

19178000.000 3.08535 201780000.000 5.89 3.21 2.96 6.12 5.65

19185000.000 3.08541 201850000.000 5.89 3.21 2.96 6.12 5.65

19192000.000 3.08546 201920000.000 5.89 3.21 2.96 6.12 5.65

19199000.000 3.08552 201990000.000 5.89 3.21 2.96 6.12 5.65

19206000.000 3.08558 202060000.000 5.89 3.21 2.96 6.12 5.65

19213000.000 3.08563 202130000.000 5.89 3.21 2.96 6.12 5.65

19220000.000 3.08569 202200000.000 5.89 3.21 2.96 6.12 5.65

19227000.000 3.08575 202270000.000 5.89 3.21 2.96 6.12 5.65

19234000.000 3.08580 202340000.000 5.89 3.21 2.96 6.12 5.65

19241000.000 3.08586 202410000.000 5.89 3.21 2.96 6.12 5.65

19248000.000 3.08591 202480000.000 5.89 3.21 2.96 6.12 5.65

19255000.000 3.08597 202550000.000 5.89 3.21 2.96 6.12 5.65

19262000.000 3.08603 202620000.000 5.89 3.21 2.96 6.12 5.65

19269000.000 3.08608 202690000.000 5.89 3.21 2.96 6.12 5.65

19276000.000 3.08614 202760000.000 5.89 3.21 2.96 6.12 5.65

19283000.000 3.08620 202830000.000 5.89 3.21 2.96 6.12 5.65

19290000.000 3.08625 202900000.000 5.89 3.21 2.96 6.12 5.65

19297000.000 3.08631 202970000.000 5.89 3.21 2.96 6.12 5.65

19304000.000 3.08636 203040000.000 5.89 3.21 2.96 6.12 5.65

19311000.000 3.08642 203110000.000 5.89 3.21 2.96 6.12 5.65

19318000.000 3.08648 203180000.000 5.89 3.21 2.96 6.12 5.65

19325000.000 3.08653 203250000.000 5.89 3.21 2.96 6.12 5.65

19332000.000 3.08659 203320000.000 5.89 3.21 2.96 6.12 5.65

19339000.000 3.08664 203390000.000 5.89 3.21 2.96 6.12 5.65

19346000.000 3.08670 203460000.000 5.89 3.21 2.96 6.12 5.65

19353000.000 3.08676 203530000.000 5.89 3.21 2.96 6.12 5.65

19360000.000 3.08681 203600000.000 5.89 3.21 2.96 6.12 5.65

19367000.000 3.08687 203670000.000 5.89 3.21 2.96 6.12 5.65

19374000.000 3.08692 203740000.000 5.89 3.21 2.96 6.12 5.65

19381000.000 3.08698 203810000.000 5.89 3.21 2.96 6.12 5.65

19388000.000 3.08704 203880000.000 5.89 3.21 2.96 6.12 5.65

19395000.000 3.08709 203950000.000 5.89 3.21 2.96 6.12 5.65

19402000.000 3.08715 204020000.000 5.89 3.21 2.96 6.12 5.65

19409000.000 3.08720 204090000.000 5.89 3.21 2.96 6.12 5.65

19416000.000 3.08726 204160000.000 5.89 3.21 2.96 6.13 5.65

19423000.000 3.08732 204230000.000 5.89 3.21 2.96 6.13 5.65

19430000.000 3.08737 204300000.000 5.89 3.21 2.96 6.13 5.65

19437000.000 3.08743 204370000.000 5.89 3.21 2.96 6.13 5.65

19444000.000 3.08748 204440000.000 5.89 3.21 2.96 6.13 5.65

19451000.000 3.08754 204510000.000 5.89 3.21 2.96 6.13 5.65

19458000.000 3.08759 204580000.000 5.89 3.21 2.96 6.13 5.65

19465000.000 3.08765 204650000.000 5.89 3.21 2.96 6.13 5.65

19472000.000 3.08771 204720000.000 5.89 3.21 2.96 6.13 5.65

19479000.000 3.08776 204790000.000 5.89 3.21 2.96 6.13 5.65

19486000.000 3.08782 204860000.000 5.89 3.21 2.96 6.13 5.65

19493000.000 3.08787 204930000.000 5.89 3.21 2.96 6.13 5.65

19500000.000 3.08793 205000000.000 5.89 3.21 2.96 6.13 5.66

19507000.000 3.08798 205070000.000 5.89 3.21 2.96 6.13 5.66

19514000.000 3.08804 205140000.000 5.89 3.21 2.96 6.13 5.66

19521000.000 3.08809 205210000.000 5.89 3.21 2.96 6.13 5.66

19528000.000 3.08815 205280000.000 5.89 3.21 2.96 6.13 5.66

19535000.000 3.08821 205350000.000 5.89 3.21 2.96 6.13 5.66

19542000.000 3.08826 205420000.000 5.89 3.21 2.96 6.13 5.66

19549000.000 3.08832 205490000.000 5.89 3.21 2.96 6.13 5.66

19556000.000 3.08837 205560000.000 5.89 3.21 2.96 6.13 5.66

19563000.000 3.08843 205630000.000 5.89 3.21 2.96 6.13 5.66

19570000.000 3.08848 205700000.000 5.89 3.21 2.96 6.13 5.66

19577000.000 3.08854 205770000.000 5.89 3.21 2.96 6.13 5.66

19584000.000 3.08859 205840000.000 5.89 3.21 2.97 6.13 5.66

19591000.000 3.08865 205910000.000 5.89 3.21 2.97 6.13 5.66

19598000.000 3.08870 205980000.000 5.89 3.21 2.97 6.13 5.66

19605000.000 3.08876 206050000.000 5.89 3.21 2.97 6.13 5.66

19612000.000 3.08882 206120000.000 5.89 3.21 2.97 6.13 5.66

19619000.000 3.08887 206190000.000 5.89 3.21 2.97 6.13 5.66

19626000.000 3.08893 206260000.000 5.89 3.21 2.97 6.13 5.66

19633000.000 3.08898 206330000.000 5.89 3.21 2.97 6.13 5.66

19640000.000 3.08904 206400000.000 5.89 3.21 2.97 6.13 5.66

19647000.000 3.08909 206470000.000 5.89 3.21 2.97 6.13 5.66

19654000.000 3.08915 206540000.000 5.89 3.21 2.97 6.13 5.66

19661000.000 3.08920 206610000.000 5.89 3.21 2.97 6.13 5.66

19668000.000 3.08926 206680000.000 5.89 3.21 2.97 6.13 5.66

19675000.000 3.08931 206750000.000 5.89 3.21 2.97 6.13 5.66

19682000.000 3.08937 206820000.000 5.89 3.21 2.97 6.13 5.66

19689000.000 3.08942 206890000.000 5.89 3.21 2.97 6.13 5.66

19696000.000 3.08948 206960000.000 5.89 3.21 2.97 6.13 5.66

19703000.000 3.08953 207030000.000 5.89 3.21 2.97 6.13 5.66

19710000.000 3.08959 207100000.000 5.89 3.21 2.97 6.13 5.66

19717000.000 3.08964 207170000.000 5.89 3.21 2.97 6.13 5.66

19724000.000 3.08970 207240000.000 5.89 3.21 2.97 6.13 5.66

19731000.000 3.08975 207310000.000 5.89 3.21 2.97 6.13 5.66

19738000.000 3.08981 207380000.000 5.89 3.21 2.97 6.13 5.66

19745000.000 3.08986 207450000.000 5.89 3.21 2.97 6.13 5.66

19752000.000 3.08992 207520000.000 5.89 3.21 2.97 6.13 5.66

19759000.000 3.08997 207590000.000 5.89 3.21 2.97 6.13 5.66

19766000.000 3.09003 207660000.000 5.89 3.21 2.97 6.13 5.66

19773000.000 3.09008 207730000.000 5.89 3.21 2.97 6.13 5.66

19780000.000 3.09014 207800000.000 5.89 3.21 2.97 6.13 5.66

19787000.000 3.09019 207870000.000 5.89 3.21 2.97 6.13 5.66

19794000.000 3.09025 207940000.000 5.89 3.21 2.97 6.13 5.66

19801000.000 3.09030 208010000.000 5.89 3.21 2.97 6.13 5.66

19808000.000 3.09036 208080000.000 5.90 3.21 2.97 6.13 5.66

19815000.000 3.09041 208150000.000 5.90 3.21 2.97 6.13 5.66

19822000.000 3.09047 208220000.000 5.90 3.21 2.97 6.13 5.66

19829000.000 3.09052 208290000.000 5.90 3.21 2.97 6.13 5.66

19836000.000 3.09058 208360000.000 5.90 3.21 2.97 6.13 5.66

19843000.000 3.09063 208430000.000 5.90 3.21 2.97 6.13 5.66

19850000.000 3.09068 208500000.000 5.90 3.21 2.97 6.13 5.66

19857000.000 3.09074 208570000.000 5.90 3.21 2.97 6.13 5.66

19864000.000 3.09079 208640000.000 5.90 3.21 2.97 6.13 5.66

19871000.000 3.09085 208710000.000 5.90 3.21 2.97 6.13 5.66

19878000.000 3.09090 208780000.000 5.90 3.21 2.97 6.13 5.66

19885000.000 3.09096 208850000.000 5.90 3.21 2.97 6.13 5.66

19892000.000 3.09101 208920000.000 5.90 3.21 2.97 6.13 5.66

19899000.000 3.09107 208990000.000 5.90 3.21 2.97 6.13 5.66

19906000.000 3.09112 209060000.000 5.90 3.21 2.97 6.13 5.66

19913000.000 3.09118 209130000.000 5.90 3.21 2.97 6.13 5.66

19920000.000 3.09123 209200000.000 5.90 3.21 2.97 6.13 5.66

19927000.000 3.09129 209270000.000 5.90 3.21 2.97 6.13 5.66

19934000.000 3.09134 209340000.000 5.90 3.21 2.97 6.13 5.66

19941000.000 3.09139 209410000.000 5.90 3.22 2.97 6.13 5.66

19948000.000 3.09145 209480000.000 5.90 3.22 2.97 6.13 5.66

19955000.000 3.09150 209550000.000 5.90 3.22 2.97 6.13 5.66

19962000.000 3.09156 209620000.000 5.90 3.22 2.97 6.13 5.66

19969000.000 3.09161 209690000.000 5.90 3.22 2.97 6.13 5.66

19976000.000 3.09167 209760000.000 5.90 3.22 2.97 6.13 5.66

19983000.000 3.09172 209830000.000 5.90 3.22 2.97 6.13 5.66

19990000.000 3.09177 209900000.000 5.90 3.22 2.97 6.13 5.66

19997000.000 3.09183 209970000.000 5.90 3.22 2.97 6.13 5.66

20004000.000 3.09188 210040000.000 5.90 3.22 2.97 6.13 5.66

20011000.000 3.09194 210110000.000 5.90 3.22 2.97 6.13 5.66

20018000.000 3.09199 210180000.000 5.90 3.22 2.97 6.13 5.66

20025000.000 3.09205 210250000.000 5.90 3.22 2.97 6.13 5.66

20032000.000 3.09210 210320000.000 5.90 3.22 2.97 6.13 5.66

20039000.000 3.09215 210390000.000 5.90 3.22 2.97 6.13 5.66

20046000.000 3.09221 210460000.000 5.90 3.22 2.97 6.13 5.66

20053000.000 3.09226 210530000.000 5.90 3.22 2.97 6.13 5.66

20060000.000 3.09232 210600000.000 5.90 3.22 2.97 6.13 5.66

20067000.000 3.09237 210670000.000 5.90 3.22 2.97 6.13 5.66

20074000.000 3.09242 210740000.000 5.90 3.22 2.97 6.13 5.66

20081000.000 3.09248 210810000.000 5.90 3.22 2.97 6.13 5.66

20088000.000 3.09253 210880000.000 5.90 3.22 2.97 6.14 5.66

20095000.000 3.09259 210950000.000 5.90 3.22 2.97 6.14 5.66

20102000.000 3.09264 211020000.000 5.90 3.22 2.97 6.14 5.66

20109000.000 3.09270 211090000.000 5.90 3.22 2.97 6.14 5.66

20116000.000 3.09275 211160000.000 5.90 3.22 2.97 6.14 5.66

20123000.000 3.09280 211230000.000 5.90 3.22 2.97 6.14 5.66

20130000.000 3.09286 211300000.000 5.90 3.22 2.97 6.14 5.66

20137000.000 3.09291 211370000.000 5.90 3.22 2.97 6.14 5.66

20144000.000 3.09296 211440000.000 5.90 3.22 2.97 6.14 5.66

20151000.000 3.09302 211510000.000 5.90 3.22 2.97 6.14 5.66

20158000.000 3.09307 211580000.000 5.90 3.22 2.97 6.14 5.66

20165000.000 3.09313 211650000.000 5.90 3.22 2.97 6.14 5.66

20172000.000 3.09318 211720000.000 5.90 3.22 2.97 6.14 5.66

20179000.000 3.09323 211790000.000 5.90 3.22 2.97 6.14 5.66

20186000.000 3.09329 211860000.000 5.90 3.22 2.97 6.14 5.66

20193000.000 3.09334 211930000.000 5.90 3.22 2.97 6.14 5.66

20200000.000 3.09340 212000000.000 5.90 3.22 2.97 6.14 5.66

20207000.000 3.09345 212070000.000 5.90 3.22 2.97 6.14 5.66

20214000.000 3.09350 212140000.000 5.90 3.22 2.97 6.14 5.66

20221000.000 3.09356 212210000.000 5.90 3.22 2.97 6.14 5.66

20228000.000 3.09361 212280000.000 5.90 3.22 2.97 6.14 5.66

20235000.000 3.09366 212350000.000 5.90 3.22 2.97 6.14 5.67

20242000.000 3.09372 212420000.000 5.90 3.22 2.97 6.14 5.67

20249000.000 3.09377 212490000.000 5.90 3.22 2.97 6.14 5.67

20256000.000 3.09383 212560000.000 5.90 3.22 2.97 6.14 5.67

20263000.000 3.09388 212630000.000 5.90 3.22 2.97 6.14 5.67

20270000.000 3.09393 212700000.000 5.90 3.22 2.97 6.14 5.67

20277000.000 3.09399 212770000.000 5.90 3.22 2.97 6.14 5.67

20284000.000 3.09404 212840000.000 5.90 3.22 2.97 6.14 5.67

20291000.000 3.09409 212910000.000 5.90 3.22 2.97 6.14 5.67

20298000.000 3.09415 212980000.000 5.90 3.22 2.97 6.14 5.67

20305000.000 3.09420 213050000.000 5.90 3.22 2.97 6.14 5.67

20312000.000 3.09425 213120000.000 5.90 3.22 2.97 6.14 5.67

20319000.000 3.09431 213190000.000 5.90 3.22 2.97 6.14 5.67

20326000.000 3.09436 213260000.000 5.90 3.22 2.97 6.14 5.67

20333000.000 3.09441 213330000.000 5.90 3.22 2.97 6.14 5.67

20340000.000 3.09447 213400000.000 5.90 3.22 2.97 6.14 5.67

20347000.000 3.09452 213470000.000 5.90 3.22 2.97 6.14 5.67

20354000.000 3.09457 213540000.000 5.90 3.22 2.97 6.14 5.67

20361000.000 3.09463 213610000.000 5.90 3.22 2.97 6.14 5.67

20368000.000 3.09468 213680000.000 5.90 3.22 2.97 6.14 5.67

20375000.000 3.09473 213750000.000 5.90 3.22 2.97 6.14 5.67

20382000.000 3.09479 213820000.000 5.90 3.22 2.97 6.14 5.67

20389000.000 3.09484 213890000.000 5.90 3.22 2.97 6.14 5.67

20396000.000 3.09489 213960000.000 5.90 3.22 2.97 6.14 5.67

20403000.000 3.09495 214030000.000 5.90 3.22 2.97 6.14 5.67

20410000.000 3.09500 214100000.000 5.90 3.22 2.97 6.14 5.67

20417000.000 3.09505 214170000.000 5.90 3.22 2.97 6.14 5.67

20424000.000 3.09511 214240000.000 5.90 3.22 2.97 6.14 5.67

20431000.000 3.09516 214310000.000 5.90 3.22 2.97 6.14 5.67

20438000.000 3.09521 214380000.000 5.90 3.22 2.97 6.14 5.67

20445000.000 3.09527 214450000.000 5.90 3.22 2.97 6.14 5.67

20452000.000 3.09532 214520000.000 5.90 3.22 2.97 6.14 5.67

20459000.000 3.09537 214590000.000 5.90 3.22 2.97 6.14 5.67

20466000.000 3.09543 214660000.000 5.90 3.22 2.97 6.14 5.67

20473000.000 3.09548 214730000.000 5.90 3.22 2.97 6.14 5.67

20480000.000 3.09553 214800000.000 5.90 3.22 2.97 6.14 5.67

20487000.000 3.09559 214870000.000 5.90 3.22 2.97 6.14 5.67

20494000.000 3.09564 214940000.000 5.90 3.22 2.97 6.14 5.67

20501000.000 3.09569 215010000.000 5.90 3.22 2.97 6.14 5.67

20508000.000 3.09574 215080000.000 5.90 3.22 2.97 6.14 5.67

20515000.000 3.09580 215150000.000 5.90 3.22 2.97 6.14 5.67

20522000.000 3.09585 215220000.000 5.91 3.22 2.97 6.14 5.67

20529000.000 3.09590 215290000.000 5.91 3.22 2.97 6.14 5.67

20536000.000 3.09596 215360000.000 5.91 3.22 2.97 6.14 5.67

20543000.000 3.09601 215430000.000 5.91 3.22 2.97 6.14 5.67

20550000.000 3.09606 215500000.000 5.91 3.22 2.97 6.14 5.67

20557000.000 3.09612 215570000.000 5.91 3.22 2.97 6.14 5.67

20564000.000 3.09617 215640000.000 5.91 3.22 2.97 6.14 5.67

20571000.000 3.09622 215710000.000 5.91 3.22 2.97 6.14 5.67

20578000.000 3.09627 215780000.000 5.91 3.22 2.97 6.14 5.67

20585000.000 3.09633 215850000.000 5.91 3.22 2.97 6.14 5.67

20592000.000 3.09638 215920000.000 5.91 3.22 2.97 6.14 5.67

20599000.000 3.09643 215990000.000 5.91 3.22 2.97 6.14 5.67

20606000.000 3.09648 216060000.000 5.91 3.22 2.97 6.14 5.67

20613000.000 3.09654 216130000.000 5.91 3.22 2.97 6.14 5.67

20620000.000 3.09659 216200000.000 5.91 3.22 2.97 6.14 5.67

20627000.000 3.09664 216270000.000 5.91 3.22 2.97 6.14 5.67

20634000.000 3.09670 216340000.000 5.91 3.22 2.97 6.14 5.67

20641000.000 3.09675 216410000.000 5.91 3.22 2.97 6.14 5.67

20648000.000 3.09680 216480000.000 5.91 3.22 2.97 6.14 5.67

20655000.000 3.09685 216550000.000 5.91 3.22 2.97 6.14 5.67

20662000.000 3.09691 216620000.000 5.91 3.22 2.97 6.14 5.67

20669000.000 3.09696 216690000.000 5.91 3.22 2.97 6.14 5.67

20676000.000 3.09701 216760000.000 5.91 3.22 2.97 6.14 5.67

20683000.000 3.09706 216830000.000 5.91 3.22 2.97 6.14 5.67

20690000.000 3.09712 216900000.000 5.91 3.22 2.97 6.14 5.67

20697000.000 3.09717 216970000.000 5.91 3.22 2.97 6.14 5.67

20704000.000 3.09722 217040000.000 5.91 3.22 2.97 6.14 5.67

20711000.000 3.09727 217110000.000 5.91 3.22 2.97 6.14 5.67

20718000.000 3.09733 217180000.000 5.91 3.22 2.97 6.14 5.67

20725000.000 3.09738 217250000.000 5.91 3.22 2.97 6.14 5.67

20732000.000 3.09743 217320000.000 5.91 3.22 2.97 6.14 5.67

20739000.000 3.09748 217390000.000 5.91 3.22 2.97 6.14 5.67

20746000.000 3.09754 217460000.000 5.91 3.22 2.97 6.14 5.67

20753000.000 3.09759 217530000.000 5.91 3.22 2.97 6.14 5.67

20760000.000 3.09764 217600000.000 5.91 3.22 2.97 6.14 5.67

20767000.000 3.09769 217670000.000 5.91 3.22 2.97 6.14 5.67

20774000.000 3.09775 217740000.000 5.91 3.22 2.97 6.14 5.67

20781000.000 3.09780 217810000.000 5.91 3.22 2.97 6.14 5.67

20788000.000 3.09785 217880000.000 5.91 3.22 2.97 6.15 5.67

20795000.000 3.09790 217950000.000 5.91 3.22 2.97 6.15 5.67

20802000.000 3.09796 218020000.000 5.91 3.22 2.97 6.15 5.67

20809000.000 3.09801 218090000.000 5.91 3.22 2.97 6.15 5.67

20816000.000 3.09806 218160000.000 5.91 3.22 2.97 6.15 5.67

20823000.000 3.09811 218230000.000 5.91 3.22 2.97 6.15 5.67

20830000.000 3.09816 218300000.000 5.91 3.22 2.97 6.15 5.67

20837000.000 3.09822 218370000.000 5.91 3.22 2.97 6.15 5.67

20844000.000 3.09827 218440000.000 5.91 3.22 2.97 6.15 5.67

20851000.000 3.09832 218510000.000 5.91 3.22 2.97 6.15 5.67

20858000.000 3.09837 218580000.000 5.91 3.22 2.97 6.15 5.67

20865000.000 3.09843 218650000.000 5.91 3.22 2.97 6.15 5.67

20872000.000 3.09848 218720000.000 5.91 3.22 2.97 6.15 5.67

20879000.000 3.09853 218790000.000 5.91 3.22 2.97 6.15 5.67

20886000.000 3.09858 218860000.000 5.91 3.22 2.97 6.15 5.67

20893000.000 3.09863 218930000.000 5.91 3.22 2.97 6.15 5.67

20900000.000 3.09869 219000000.000 5.91 3.22 2.97 6.15 5.67

20907000.000 3.09874 219070000.000 5.91 3.22 2.97 6.15 5.67

20914000.000 3.09879 219140000.000 5.91 3.22 2.97 6.15 5.67

20921000.000 3.09884 219210000.000 5.91 3.22 2.97 6.15 5.67

20928000.000 3.09889 219280000.000 5.91 3.22 2.97 6.15 5.67

20935000.000 3.09895 219350000.000 5.91 3.22 2.97 6.15 5.67

20942000.000 3.09900 219420000.000 5.91 3.22 2.98 6.15 5.67

20949000.000 3.09905 219490000.000 5.91 3.22 2.98 6.15 5.67

20956000.000 3.09910 219560000.000 5.91 3.22 2.98 6.15 5.67

20963000.000 3.09915 219630000.000 5.91 3.22 2.98 6.15 5.67

20970000.000 3.09921 219700000.000 5.91 3.22 2.98 6.15 5.67

20977000.000 3.09926 219770000.000 5.91 3.22 2.98 6.15 5.67

20984000.000 3.09931 219840000.000 5.91 3.22 2.98 6.15 5.67

20991000.000 3.09936 219910000.000 5.91 3.22 2.98 6.15 5.68

20998000.000 3.09941 219980000.000 5.91 3.22 2.98 6.15 5.68

21005000.000 3.09947 220050000.000 5.91 3.22 2.98 6.15 5.68

21012000.000 3.09952 220120000.000 5.91 3.22 2.98 6.15 5.68

21019000.000 3.09957 220190000.000 5.91 3.22 2.98 6.15 5.68

21026000.000 3.09962 220260000.000 5.91 3.22 2.98 6.15 5.68

21033000.000 3.09967 220330000.000 5.91 3.22 2.98 6.15 5.68

21040000.000 3.09972 220400000.000 5.91 3.22 2.98 6.15 5.68

21047000.000 3.09978 220470000.000 5.91 3.22 2.98 6.15 5.68

21054000.000 3.09983 220540000.000 5.91 3.22 2.98 6.15 5.68

21061000.000 3.09988 220610000.000 5.91 3.22 2.98 6.15 5.68

21068000.000 3.09993 220680000.000 5.91 3.22 2.98 6.15 5.68

21075000.000 3.09998 220750000.000 5.91 3.22 2.98 6.15 5.68

21082000.000 3.10003 220820000.000 5.91 3.22 2.98 6.15 5.68

21089000.000 3.10009 220890000.000 5.91 3.22 2.98 6.15 5.68

21096000.000 3.10014 220960000.000 5.91 3.22 2.98 6.15 5.68

21103000.000 3.10019 221030000.000 5.91 3.22 2.98 6.15 5.68

21110000.000 3.10024 221100000.000 5.91 3.22 2.98 6.15 5.68

21117000.000 3.10029 221170000.000 5.91 3.22 2.98 6.15 5.68

21124000.000 3.10034 221240000.000 5.91 3.22 2.98 6.15 5.68

21131000.000 3.10040 221310000.000 5.91 3.22 2.98 6.15 5.68

21138000.000 3.10045 221380000.000 5.91 3.22 2.98 6.15 5.68

21145000.000 3.10050 221450000.000 5.91 3.22 2.98 6.15 5.68

21152000.000 3.10055 221520000.000 5.91 3.22 2.98 6.15 5.68

21159000.000 3.10060 221590000.000 5.91 3.22 2.98 6.15 5.68

21166000.000 3.10065 221660000.000 5.91 3.22 2.98 6.15 5.68

21173000.000 3.10070 221730000.000 5.91 3.22 2.98 6.15 5.68

21180000.000 3.10076 221800000.000 5.91 3.22 2.98 6.15 5.68

21187000.000 3.10081 221870000.000 5.91 3.22 2.98 6.15 5.68

21194000.000 3.10086 221940000.000 5.91 3.22 2.98 6.15 5.68

21201000.000 3.10091 222010000.000 5.91 3.22 2.98 6.15 5.68

21208000.000 3.10096 222080000.000 5.91 3.22 2.98 6.15 5.68

21215000.000 3.10101 222150000.000 5.91 3.23 2.98 6.15 5.68

21222000.000 3.10106 222220000.000 5.91 3.23 2.98 6.15 5.68

21229000.000 3.10111 222290000.000 5.91 3.23 2.98 6.15 5.68

21236000.000 3.10117 222360000.000 5.91 3.23 2.98 6.15 5.68

21243000.000 3.10122 222430000.000 5.91 3.23 2.98 6.15 5.68

21250000.000 3.10127 222500000.000 5.91 3.23 2.98 6.15 5.68

21257000.000 3.10132 222570000.000 5.92 3.23 2.98 6.15 5.68

21264000.000 3.10137 222640000.000 5.92 3.23 2.98 6.15 5.68

21271000.000 3.10142 222710000.000 5.92 3.23 2.98 6.15 5.68

21278000.000 3.10147 222780000.000 5.92 3.23 2.98 6.15 5.68

21285000.000 3.10152 222850000.000 5.92 3.23 2.98 6.15 5.68

21292000.000 3.10158 222920000.000 5.92 3.23 2.98 6.15 5.68

21299000.000 3.10163 222990000.000 5.92 3.23 2.98 6.15 5.68

21306000.000 3.10168 223060000.000 5.92 3.23 2.98 6.15 5.68

21313000.000 3.10173 223130000.000 5.92 3.23 2.98 6.15 5.68

21320000.000 3.10178 223200000.000 5.92 3.23 2.98 6.15 5.68

21327000.000 3.10183 223270000.000 5.92 3.23 2.98 6.15 5.68

21334000.000 3.10188 223340000.000 5.92 3.23 2.98 6.15 5.68

21341000.000 3.10193 223410000.000 5.92 3.23 2.98 6.15 5.68

21348000.000 3.10198 223480000.000 5.92 3.23 2.98 6.15 5.68

21355000.000 3.10204 223550000.000 5.92 3.23 2.98 6.15 5.68

21362000.000 3.10209 223620000.000 5.92 3.23 2.98 6.15 5.68

21369000.000 3.10214 223690000.000 5.92 3.23 2.98 6.15 5.68

21376000.000 3.10219 223760000.000 5.92 3.23 2.98 6.15 5.68

21383000.000 3.10224 223830000.000 5.92 3.23 2.98 6.15 5.68

21390000.000 3.10229 223900000.000 5.92 3.23 2.98 6.15 5.68

21397000.000 3.10234 223970000.000 5.92 3.23 2.98 6.15 5.68

21404000.000 3.10239 224040000.000 5.92 3.23 2.98 6.15 5.68

21411000.000 3.10244 224110000.000 5.92 3.23 2.98 6.15 5.68

21418000.000 3.10249 224180000.000 5.92 3.23 2.98 6.15 5.68

21425000.000 3.10254 224250000.000 5.92 3.23 2.98 6.15 5.68

21432000.000 3.10260 224320000.000 5.92 3.23 2.98 6.15 5.68

21439000.000 3.10265 224390000.000 5.92 3.23 2.98 6.15 5.68

21446000.000 3.10270 224460000.000 5.92 3.23 2.98 6.15 5.68

21453000.000 3.10275 224530000.000 5.92 3.23 2.98 6.15 5.68

21460000.000 3.10280 224600000.000 5.92 3.23 2.98 6.15 5.68

21467000.000 3.10285 224670000.000 5.92 3.23 2.98 6.15 5.68

21474000.000 3.10290 224740000.000 5.92 3.23 2.98 6.15 5.68

21481000.000 3.10295 224810000.000 5.92 3.23 2.98 6.15 5.68

21488000.000 3.10300 224880000.000 5.92 3.23 2.98 6.15 5.68

21495000.000 3.10305 224950000.000 5.92 3.23 2.98 6.15 5.68

21502000.000 3.10310 225020000.000 5.92 3.23 2.98 6.16 5.68

21509000.000 3.10315 225090000.000 5.92 3.23 2.98 6.16 5.68

21516000.000 3.10320 225160000.000 5.92 3.23 2.98 6.16 5.68

21523000.000 3.10325 225230000.000 5.92 3.23 2.98 6.16 5.68

21530000.000 3.10331 225300000.000 5.92 3.23 2.98 6.16 5.68

21537000.000 3.10336 225370000.000 5.92 3.23 2.98 6.16 5.68

21544000.000 3.10341 225440000.000 5.92 3.23 2.98 6.16 5.68

21551000.000 3.10346 225510000.000 5.92 3.23 2.98 6.16 5.68

21558000.000 3.10351 225580000.000 5.92 3.23 2.98 6.16 5.68

21565000.000 3.10356 225650000.000 5.92 3.23 2.98 6.16 5.68

21572000.000 3.10361 225720000.000 5.92 3.23 2.98 6.16 5.68

21579000.000 3.10366 225790000.000 5.92 3.23 2.98 6.16 5.68

21586000.000 3.10371 225860000.000 5.92 3.23 2.98 6.16 5.68

21593000.000 3.10376 225930000.000 5.92 3.23 2.98 6.16 5.68

21600000.000 3.10381 226000000.000 5.92 3.23 2.98 6.16 5.68

21607000.000 3.10386 226070000.000 5.92 3.23 2.98 6.16 5.68

21614000.000 3.10391 226140000.000 5.92 3.23 2.98 6.16 5.68

21621000.000 3.10396 226210000.000 5.92 3.23 2.98 6.16 5.68

21628000.000 3.10401 226280000.000 5.92 3.23 2.98 6.16 5.68

21635000.000 3.10406 226350000.000 5.92 3.23 2.98 6.16 5.68

21642000.000 3.10411 226420000.000 5.92 3.23 2.98 6.16 5.68

21649000.000 3.10416 226490000.000 5.92 3.23 2.98 6.16 5.68

21656000.000 3.10421 226560000.000 5.92 3.23 2.98 6.16 5.68

21663000.000 3.10426 226630000.000 5.92 3.23 2.98 6.16 5.68

21670000.000 3.10431 226700000.000 5.92 3.23 2.98 6.16 5.68

21677000.000 3.10436 226770000.000 5.92 3.23 2.98 6.16 5.68

21684000.000 3.10442 226840000.000 5.92 3.23 2.98 6.16 5.68

21691000.000 3.10447 226910000.000 5.92 3.23 2.98 6.16 5.68

21698000.000 3.10452 226980000.000 5.92 3.23 2.98 6.16 5.68

21705000.000 3.10457 227050000.000 5.92 3.23 2.98 6.16 5.68

21712000.000 3.10462 227120000.000 5.92 3.23 2.98 6.16 5.68

21719000.000 3.10467 227190000.000 5.92 3.23 2.98 6.16 5.68

21726000.000 3.10472 227260000.000 5.92 3.23 2.98 6.16 5.68

21733000.000 3.10477 227330000.000 5.92 3.23 2.98 6.16 5.68

21740000.000 3.10482 227400000.000 5.92 3.23 2.98 6.16 5.68

21747000.000 3.10487 227470000.000 5.92 3.23 2.98 6.16 5.68

21754000.000 3.10492 227540000.000 5.92 3.23 2.98 6.16 5.68

21761000.000 3.10497 227610000.000 5.92 3.23 2.98 6.16 5.68

21768000.000 3.10502 227680000.000 5.92 3.23 2.98 6.16 5.68

21775000.000 3.10507 227750000.000 5.92 3.23 2.98 6.16 5.68

21782000.000 3.10512 227820000.000 5.92 3.23 2.98 6.16 5.69

21789000.000 3.10517 227890000.000 5.92 3.23 2.98 6.16 5.69

21796000.000 3.10522 227960000.000 5.92 3.23 2.98 6.16 5.69

21803000.000 3.10527 228030000.000 5.92 3.23 2.98 6.16 5.69

21810000.000 3.10532 228100000.000 5.92 3.23 2.98 6.16 5.69

21817000.000 3.10537 228170000.000 5.92 3.23 2.98 6.16 5.69

21824000.000 3.10542 228240000.000 5.92 3.23 2.98 6.16 5.69

21831000.000 3.10547 228310000.000 5.92 3.23 2.98 6.16 5.69

21838000.000 3.10552 228380000.000 5.92 3.23 2.98 6.16 5.69

21845000.000 3.10557 228450000.000 5.92 3.23 2.98 6.16 5.69

21852000.000 3.10562 228520000.000 5.92 3.23 2.98 6.16 5.69

21859000.000 3.10567 228590000.000 5.92 3.23 2.98 6.16 5.69

21866000.000 3.10572 228660000.000 5.92 3.23 2.98 6.16 5.69

21873000.000 3.10577 228730000.000 5.92 3.23 2.98 6.16 5.69

21880000.000 3.10582 228800000.000 5.92 3.23 2.98 6.16 5.69

21887000.000 3.10587 228870000.000 5.92 3.23 2.98 6.16 5.69

21894000.000 3.10592 228940000.000 5.92 3.23 2.98 6.16 5.69

21901000.000 3.10597 229010000.000 5.92 3.23 2.98 6.16 5.69

21908000.000 3.10602 229080000.000 5.92 3.23 2.98 6.16 5.69

21915000.000 3.10607 229150000.000 5.92 3.23 2.98 6.16 5.69

21922000.000 3.10612 229220000.000 5.92 3.23 2.98 6.16 5.69

21929000.000 3.10617 229290000.000 5.92 3.23 2.98 6.16 5.69

21936000.000 3.10621 229360000.000 5.92 3.23 2.98 6.16 5.69

21943000.000 3.10626 229430000.000 5.92 3.23 2.98 6.16 5.69

21950000.000 3.10631 229500000.000 5.92 3.23 2.98 6.16 5.69

21957000.000 3.10636 229570000.000 5.92 3.23 2.98 6.16 5.69

21964000.000 3.10641 229640000.000 5.92 3.23 2.98 6.16 5.69

21971000.000 3.10646 229710000.000 5.92 3.23 2.98 6.16 5.69

21978000.000 3.10651 229780000.000 5.92 3.23 2.98 6.16 5.69

21985000.000 3.10656 229850000.000 5.92 3.23 2.98 6.16 5.69

21992000.000 3.10661 229920000.000 5.92 3.23 2.98 6.16 5.69

21999000.000 3.10666 229990000.000 5.92 3.23 2.98 6.16 5.69

22006000.000 3.10671 230060000.000 5.92 3.23 2.98 6.16 5.69

22013000.000 3.10676 230130000.000 5.92 3.23 2.98 6.16 5.69

22020000.000 3.10681 230200000.000 5.93 3.23 2.98 6.16 5.69

22027000.000 3.10686 230270000.000 5.93 3.23 2.98 6.16 5.69

22034000.000 3.10691 230340000.000 5.93 3.23 2.98 6.16 5.69

22041000.000 3.10696 230410000.000 5.93 3.23 2.98 6.16 5.69

22048000.000 3.10701 230480000.000 5.93 3.23 2.98 6.16 5.69

22055000.000 3.10706 230550000.000 5.93 3.23 2.98 6.16 5.69

22062000.000 3.10711 230620000.000 5.93 3.23 2.98 6.16 5.69

22069000.000 3.10716 230690000.000 5.93 3.23 2.98 6.16 5.69

22076000.000 3.10721 230760000.000 5.93 3.23 2.98 6.16 5.69

22083000.000 3.10726 230830000.000 5.93 3.23 2.98 6.16 5.69

22090000.000 3.10731 230900000.000 5.93 3.23 2.98 6.16 5.69

22097000.000 3.10735 230970000.000 5.93 3.23 2.98 6.16 5.69

22104000.000 3.10740 231040000.000 5.93 3.23 2.98 6.16 5.69

22111000.000 3.10745 231110000.000 5.93 3.23 2.98 6.16 5.69

22118000.000 3.10750 231180000.000 5.93 3.23 2.98 6.16 5.69

22125000.000 3.10755 231250000.000 5.93 3.23 2.98 6.16 5.69

22132000.000 3.10760 231320000.000 5.93 3.23 2.98 6.16 5.69

22139000.000 3.10765 231390000.000 5.93 3.23 2.98 6.16 5.69

22146000.000 3.10770 231460000.000 5.93 3.23 2.98 6.16 5.69

22153000.000 3.10775 231530000.000 5.93 3.23 2.98 6.16 5.69

22160000.000 3.10780 231600000.000 5.93 3.23 2.98 6.16 5.69

22167000.000 3.10785 231670000.000 5.93 3.23 2.98 6.16 5.69

22174000.000 3.10790 231740000.000 5.93 3.23 2.98 6.16 5.69

22181000.000 3.10795 231810000.000 5.93 3.23 2.98 6.16 5.69

22188000.000 3.10800 231880000.000 5.93 3.23 2.98 6.16 5.69

22195000.000 3.10804 231950000.000 5.93 3.23 2.98 6.16 5.69

22202000.000 3.10809 232020000.000 5.93 3.23 2.98 6.16 5.69

22209000.000 3.10814 232090000.000 5.93 3.23 2.98 6.16 5.69

22216000.000 3.10819 232160000.000 5.93 3.23 2.98 6.16 5.69

22223000.000 3.10824 232230000.000 5.93 3.23 2.98 6.16 5.69

22230000.000 3.10829 232300000.000 5.93 3.23 2.98 6.16 5.69

22237000.000 3.10834 232370000.000 5.93 3.23 2.98 6.16 5.69

22244000.000 3.10839 232440000.000 5.93 3.23 2.98 6.17 5.69

22251000.000 3.10844 232510000.000 5.93 3.23 2.98 6.17 5.69

22258000.000 3.10849 232580000.000 5.93 3.23 2.98 6.17 5.69

22265000.000 3.10854 232650000.000 5.93 3.23 2.98 6.17 5.69

22272000.000 3.10858 232720000.000 5.93 3.23 2.98 6.17 5.69

22279000.000 3.10863 232790000.000 5.93 3.23 2.98 6.17 5.69

22286000.000 3.10868 232860000.000 5.93 3.23 2.98 6.17 5.69

22293000.000 3.10873 232930000.000 5.93 3.23 2.98 6.17 5.69

22300000.000 3.10878 233000000.000 5.93 3.23 2.98 6.17 5.69

22307000.000 3.10883 233070000.000 5.93 3.23 2.98 6.17 5.69

22314000.000 3.10888 233140000.000 5.93 3.23 2.98 6.17 5.69

22321000.000 3.10893 233210000.000 5.93 3.23 2.98 6.17 5.69

22328000.000 3.10898 233280000.000 5.93 3.23 2.98 6.17 5.69

22335000.000 3.10902 233350000.000 5.93 3.23 2.98 6.17 5.69

22342000.000 3.10907 233420000.000 5.93 3.23 2.98 6.17 5.69

22349000.000 3.10912 233490000.000 5.93 3.23 2.98 6.17 5.69

22356000.000 3.10917 233560000.000 5.93 3.23 2.98 6.17 5.69

22363000.000 3.10922 233630000.000 5.93 3.23 2.98 6.17 5.69

22370000.000 3.10927 233700000.000 5.93 3.23 2.98 6.17 5.69

22377000.000 3.10932 233770000.000 5.93 3.23 2.98 6.17 5.69

22384000.000 3.10937 233840000.000 5.93 3.23 2.98 6.17 5.69

22391000.000 3.10942 233910000.000 5.93 3.23 2.99 6.17 5.69

22398000.000 3.10946 233980000.000 5.93 3.23 2.99 6.17 5.69

22405000.000 3.10951 234050000.000 5.93 3.23 2.99 6.17 5.69

22412000.000 3.10956 234120000.000 5.93 3.23 2.99 6.17 5.69

22419000.000 3.10961 234190000.000 5.93 3.23 2.99 6.17 5.69

22426000.000 3.10966 234260000.000 5.93 3.23 2.99 6.17 5.69

22433000.000 3.10971 234330000.000 5.93 3.23 2.99 6.17 5.69

22440000.000 3.10976 234400000.000 5.93 3.23 2.99 6.17 5.69

22447000.000 3.10980 234470000.000 5.93 3.23 2.99 6.17 5.69

22454000.000 3.10985 234540000.000 5.93 3.23 2.99 6.17 5.69

22461000.000 3.10990 234610000.000 5.93 3.23 2.99 6.17 5.69

22468000.000 3.10995 234680000.000 5.93 3.23 2.99 6.17 5.69

22475000.000 3.11000 234750000.000 5.93 3.23 2.99 6.17 5.69

22482000.000 3.11005 234820000.000 5.93 3.23 2.99 6.17 5.69

22489000.000 3.11010 234890000.000 5.93 3.23 2.99 6.17 5.69

22496000.000 3.11015 234960000.000 5.93 3.23 2.99 6.17 5.69

22503000.000 3.11019 235030000.000 5.93 3.23 2.99 6.17 5.69

22510000.000 3.11024 235100000.000 5.93 3.23 2.99 6.17 5.69

22517000.000 3.11029 235170000.000 5.93 3.23 2.99 6.17 5.69

22524000.000 3.11034 235240000.000 5.93 3.23 2.99 6.17 5.69

22531000.000 3.11039 235310000.000 5.93 3.23 2.99 6.17 5.69

22538000.000 3.11044 235380000.000 5.93 3.23 2.99 6.17 5.69

22545000.000 3.11048 235450000.000 5.93 3.23 2.99 6.17 5.69

22552000.000 3.11053 235520000.000 5.93 3.23 2.99 6.17 5.69

22559000.000 3.11058 235590000.000 5.93 3.24 2.99 6.17 5.69

22566000.000 3.11063 235660000.000 5.93 3.24 2.99 6.17 5.69

22573000.000 3.11068 235730000.000 5.93 3.24 2.99 6.17 5.69

22580000.000 3.11073 235800000.000 5.93 3.24 2.99 6.17 5.69

22587000.000 3.11077 235870000.000 5.93 3.24 2.99 6.17 5.69

22594000.000 3.11082 235940000.000 5.93 3.24 2.99 6.17 5.70

22601000.000 3.11087 236010000.000 5.93 3.24 2.99 6.17 5.70

22608000.000 3.11092 236080000.000 5.93 3.24 2.99 6.17 5.70

22615000.000 3.11097 236150000.000 5.93 3.24 2.99 6.17 5.70

22622000.000 3.11102 236220000.000 5.93 3.24 2.99 6.17 5.70

22629000.000 3.11106 236290000.000 5.93 3.24 2.99 6.17 5.70

22636000.000 3.11111 236360000.000 5.93 3.24 2.99 6.17 5.70

22643000.000 3.11116 236430000.000 5.93 3.24 2.99 6.17 5.70

22650000.000 3.11121 236500000.000 5.93 3.24 2.99 6.17 5.70

22657000.000 3.11126 236570000.000 5.93 3.24 2.99 6.17 5.70

22664000.000 3.11131 236640000.000 5.93 3.24 2.99 6.17 5.70

22671000.000 3.11135 236710000.000 5.93 3.24 2.99 6.17 5.70

22678000.000 3.11140 236780000.000 5.93 3.24 2.99 6.17 5.70

22685000.000 3.11145 236850000.000 5.93 3.24 2.99 6.17 5.70

22692000.000 3.11150 236920000.000 5.93 3.24 2.99 6.17 5.70

22699000.000 3.11155 236990000.000 5.93 3.24 2.99 6.17 5.70

22706000.000 3.11159 237060000.000 5.93 3.24 2.99 6.17 5.70

22713000.000 3.11164 237130000.000 5.93 3.24 2.99 6.17 5.70

22720000.000 3.11169 237200000.000 5.93 3.24 2.99 6.17 5.70

22727000.000 3.11174 237270000.000 5.93 3.24 2.99 6.17 5.70

22734000.000 3.11179 237340000.000 5.93 3.24 2.99 6.17 5.70

22741000.000 3.11184 237410000.000 5.93 3.24 2.99 6.17 5.70

22748000.000 3.11188 237480000.000 5.93 3.24 2.99 6.17 5.70

22755000.000 3.11193 237550000.000 5.93 3.24 2.99 6.17 5.70

22762000.000 3.11198 237620000.000 5.93 3.24 2.99 6.17 5.70

22769000.000 3.11203 237690000.000 5.93 3.24 2.99 6.17 5.70

22776000.000 3.11208 237760000.000 5.93 3.24 2.99 6.17 5.70

22783000.000 3.11212 237830000.000 5.93 3.24 2.99 6.17 5.70

22790000.000 3.11217 237900000.000 5.93 3.24 2.99 6.17 5.70

22797000.000 3.11222 237970000.000 5.93 3.24 2.99 6.17 5.70

22804000.000 3.11227 238040000.000 5.93 3.24 2.99 6.17 5.70

22811000.000 3.11232 238110000.000 5.94 3.24 2.99 6.17 5.70

22818000.000 3.11236 238180000.000 5.94 3.24 2.99 6.17 5.70

22825000.000 3.11241 238250000.000 5.94 3.24 2.99 6.17 5.70

22832000.000 3.11246 238320000.000 5.94 3.24 2.99 6.17 5.70

22839000.000 3.11251 238390000.000 5.94 3.24 2.99 6.17 5.70

22846000.000 3.11255 238460000.000 5.94 3.24 2.99 6.17 5.70

22853000.000 3.11260 238530000.000 5.94 3.24 2.99 6.17 5.70

22860000.000 3.11265 238600000.000 5.94 3.24 2.99 6.17 5.70

22867000.000 3.11270 238670000.000 5.94 3.24 2.99 6.17 5.70

22874000.000 3.11275 238740000.000 5.94 3.24 2.99 6.17 5.70

22881000.000 3.11279 238810000.000 5.94 3.24 2.99 6.17 5.70

22888000.000 3.11284 238880000.000 5.94 3.24 2.99 6.17 5.70

22895000.000 3.11289 238950000.000 5.94 3.24 2.99 6.17 5.70

22902000.000 3.11294 239020000.000 5.94 3.24 2.99 6.17 5.70

22909000.000 3.11298 239090000.000 5.94 3.24 2.99 6.17 5.70

22916000.000 3.11303 239160000.000 5.94 3.24 2.99 6.17 5.70

22923000.000 3.11308 239230000.000 5.94 3.24 2.99 6.17 5.70

22930000.000 3.11313 239300000.000 5.94 3.24 2.99 6.17 5.70

22937000.000 3.11318 239370000.000 5.94 3.24 2.99 6.17 5.70

22944000.000 3.11322 239440000.000 5.94 3.24 2.99 6.17 5.70

22951000.000 3.11327 239510000.000 5.94 3.24 2.99 6.17 5.70

22958000.000 3.11332 239580000.000 5.94 3.24 2.99 6.17 5.70

22965000.000 3.11337 239650000.000 5.94 3.24 2.99 6.17 5.70

22972000.000 3.11341 239720000.000 5.94 3.24 2.99 6.17 5.70

22979000.000 3.11346 239790000.000 5.94 3.24 2.99 6.17 5.70

22986000.000 3.11351 239860000.000 5.94 3.24 2.99 6.17 5.70

22993000.000 3.11356 239930000.000 5.94 3.24 2.99 6.17 5.70

23000000.000 3.11360 240000000.000 5.94 3.24 2.99 6.17 5.70

23007000.000 3.11365 240070000.000 5.94 3.24 2.99 6.18 5.70

23014000.000 3.11370 240140000.000 5.94 3.24 2.99 6.18 5.70

23021000.000 3.11375 240210000.000 5.94 3.24 2.99 6.18 5.70

23028000.000 3.11379 240280000.000 5.94 3.24 2.99 6.18 5.70

23035000.000 3.11384 240350000.000 5.94 3.24 2.99 6.18 5.70

23042000.000 3.11389 240420000.000 5.94 3.24 2.99 6.18 5.70

23049000.000 3.11394 240490000.000 5.94 3.24 2.99 6.18 5.70

23056000.000 3.11398 240560000.000 5.94 3.24 2.99 6.18 5.70

23063000.000 3.11403 240630000.000 5.94 3.24 2.99 6.18 5.70

23070000.000 3.11408 240700000.000 5.94 3.24 2.99 6.18 5.70

23077000.000 3.11413 240770000.000 5.94 3.24 2.99 6.18 5.70

23084000.000 3.11417 240840000.000 5.94 3.24 2.99 6.18 5.70

23091000.000 3.11422 240910000.000 5.94 3.24 2.99 6.18 5.70

23098000.000 3.11427 240980000.000 5.94 3.24 2.99 6.18 5.70

23105000.000 3.11432 241050000.000 5.94 3.24 2.99 6.18 5.70

23112000.000 3.11436 241120000.000 5.94 3.24 2.99 6.18 5.70

23119000.000 3.11441 241190000.000 5.94 3.24 2.99 6.18 5.70

23126000.000 3.11446 241260000.000 5.94 3.24 2.99 6.18 5.70

23133000.000 3.11450 241330000.000 5.94 3.24 2.99 6.18 5.70

23140000.000 3.11455 241400000.000 5.94 3.24 2.99 6.18 5.70

23147000.000 3.11460 241470000.000 5.94 3.24 2.99 6.18 5.70

23154000.000 3.11465 241540000.000 5.94 3.24 2.99 6.18 5.70

23161000.000 3.11469 241610000.000 5.94 3.24 2.99 6.18 5.70

23168000.000 3.11474 241680000.000 5.94 3.24 2.99 6.18 5.70

23175000.000 3.11479 241750000.000 5.94 3.24 2.99 6.18 5.70

23182000.000 3.11483 241820000.000 5.94 3.24 2.99 6.18 5.70

23189000.000 3.11488 241890000.000 5.94 3.24 2.99 6.18 5.70

23196000.000 3.11493 241960000.000 5.94 3.24 2.99 6.18 5.70

23203000.000 3.11498 242030000.000 5.94 3.24 2.99 6.18 5.70

23210000.000 3.11502 242100000.000 5.94 3.24 2.99 6.18 5.70

23217000.000 3.11507 242170000.000 5.94 3.24 2.99 6.18 5.70

23224000.000 3.11512 242240000.000 5.94 3.24 2.99 6.18 5.70

23231000.000 3.11516 242310000.000 5.94 3.24 2.99 6.18 5.70

23238000.000 3.11521 242380000.000 5.94 3.24 2.99 6.18 5.70

23245000.000 3.11526 242450000.000 5.94 3.24 2.99 6.18 5.70

23252000.000 3.11531 242520000.000 5.94 3.24 2.99 6.18 5.70

23259000.000 3.11535 242590000.000 5.94 3.24 2.99 6.18 5.70

23266000.000 3.11540 242660000.000 5.94 3.24 2.99 6.18 5.70

23273000.000 3.11545 242730000.000 5.94 3.24 2.99 6.18 5.70

23280000.000 3.11549 242800000.000 5.94 3.24 2.99 6.18 5.70

23287000.000 3.11554 242870000.000 5.94 3.24 2.99 6.18 5.70

23294000.000 3.11559 242940000.000 5.94 3.24 2.99 6.18 5.70

23301000.000 3.11563 243010000.000 5.94 3.24 2.99 6.18 5.70

23308000.000 3.11568 243080000.000 5.94 3.24 2.99 6.18 5.70

23315000.000 3.11573 243150000.000 5.94 3.24 2.99 6.18 5.70

23322000.000 3.11578 243220000.000 5.94 3.24 2.99 6.18 5.70

23329000.000 3.11582 243290000.000 5.94 3.24 2.99 6.18 5.70

23336000.000 3.11587 243360000.000 5.94 3.24 2.99 6.18 5.70

23343000.000 3.11592 243430000.000 5.94 3.24 2.99 6.18 5.70

23350000.000 3.11596 243500000.000 5.94 3.24 2.99 6.18 5.70

23357000.000 3.11601 243570000.000 5.94 3.24 2.99 6.18 5.70

23364000.000 3.11606 243640000.000 5.94 3.24 2.99 6.18 5.70

23371000.000 3.11610 243710000.000 5.94 3.24 2.99 6.18 5.70

23378000.000 3.11615 243780000.000 5.94 3.24 2.99 6.18 5.70

23385000.000 3.11620 243850000.000 5.94 3.24 2.99 6.18 5.70

23392000.000 3.11624 243920000.000 5.94 3.24 2.99 6.18 5.70

23399000.000 3.11629 243990000.000 5.94 3.24 2.99 6.18 5.70

23406000.000 3.11634 244060000.000 5.94 3.24 2.99 6.18 5.70

23413000.000 3.11638 244130000.000 5.94 3.24 2.99 6.18 5.70

23420000.000 3.11643 244200000.000 5.94 3.24 2.99 6.18 5.70

23427000.000 3.11648 244270000.000 5.94 3.24 2.99 6.18 5.70

23434000.000 3.11652 244340000.000 5.94 3.24 2.99 6.18 5.71

23441000.000 3.11657 244410000.000 5.94 3.24 2.99 6.18 5.71

23448000.000 3.11662 244480000.000 5.94 3.24 2.99 6.18 5.71

23455000.000 3.11666 244550000.000 5.94 3.24 2.99 6.18 5.71

23462000.000 3.11671 244620000.000 5.94 3.24 2.99 6.18 5.71

23469000.000 3.11676 244690000.000 5.94 3.24 2.99 6.18 5.71

23476000.000 3.11680 244760000.000 5.94 3.24 2.99 6.18 5.71

23483000.000 3.11685 244830000.000 5.94 3.24 2.99 6.18 5.71

23490000.000 3.11690 244900000.000 5.94 3.24 2.99 6.18 5.71

23497000.000 3.11694 244970000.000 5.94 3.24 2.99 6.18 5.71

23504000.000 3.11699 245040000.000 5.94 3.24 2.99 6.18 5.71

23511000.000 3.11704 245110000.000 5.94 3.24 2.99 6.18 5.71

23518000.000 3.11708 245180000.000 5.94 3.24 2.99 6.18 5.71

23525000.000 3.11713 245250000.000 5.94 3.24 2.99 6.18 5.71

23532000.000 3.11718 245320000.000 5.94 3.24 2.99 6.18 5.71

23539000.000 3.11722 245390000.000 5.94 3.24 2.99 6.18 5.71

23546000.000 3.11727 245460000.000 5.94 3.24 2.99 6.18 5.71

23553000.000 3.11732 245530000.000 5.94 3.24 2.99 6.18 5.71

23560000.000 3.11736 245600000.000 5.94 3.24 2.99 6.18 5.71

23567000.000 3.11741 245670000.000 5.94 3.24 2.99 6.18 5.71

23574000.000 3.11746 245740000.000 5.94 3.24 2.99 6.18 5.71

23581000.000 3.11750 245810000.000 5.94 3.24 2.99 6.18 5.71

23588000.000 3.11755 245880000.000 5.94 3.24 2.99 6.18 5.71

23595000.000 3.11760 245950000.000 5.94 3.24 2.99 6.18 5.71

23602000.000 3.11764 246020000.000 5.94 3.24 2.99 6.18 5.71

23609000.000 3.11769 246090000.000 5.94 3.24 2.99 6.18 5.71

23616000.000 3.11773 246160000.000 5.94 3.24 2.99 6.18 5.71

23623000.000 3.11778 246230000.000 5.95 3.24 2.99 6.18 5.71

23630000.000 3.11783 246300000.000 5.95 3.24 2.99 6.18 5.71

23637000.000 3.11787 246370000.000 5.95 3.24 2.99 6.18 5.71

23644000.000 3.11792 246440000.000 5.95 3.24 2.99 6.18 5.71

23651000.000 3.11797 246510000.000 5.95 3.24 2.99 6.18 5.71

23658000.000 3.11801 246580000.000 5.95 3.24 2.99 6.18 5.71

23665000.000 3.11806 246650000.000 5.95 3.24 2.99 6.18 5.71

23672000.000 3.11810 246720000.000 5.95 3.24 2.99 6.18 5.71

23679000.000 3.11815 246790000.000 5.95 3.24 2.99 6.18 5.71

23686000.000 3.11820 246860000.000 5.95 3.24 2.99 6.18 5.71

23693000.000 3.11824 246930000.000 5.95 3.24 2.99 6.18 5.71

23700000.000 3.11829 247000000.000 5.95 3.24 2.99 6.18 5.71

23707000.000 3.11834 247070000.000 5.95 3.24 2.99 6.18 5.71

23714000.000 3.11838 247140000.000 5.95 3.24 2.99 6.18 5.71

23721000.000 3.11843 247210000.000 5.95 3.24 2.99 6.18 5.71

23728000.000 3.11847 247280000.000 5.95 3.24 2.99 6.18 5.71

23735000.000 3.11852 247350000.000 5.95 3.24 2.99 6.18 5.71

23742000.000 3.11857 247420000.000 5.95 3.24 2.99 6.18 5.71

23749000.000 3.11861 247490000.000 5.95 3.24 2.99 6.18 5.71

23756000.000 3.11866 247560000.000 5.95 3.24 2.99 6.18 5.71

23763000.000 3.11870 247630000.000 5.95 3.24 2.99 6.18 5.71

23770000.000 3.11875 247700000.000 5.95 3.24 2.99 6.18 5.71

23777000.000 3.11880 247770000.000 5.95 3.24 2.99 6.18 5.71

23784000.000 3.11884 247840000.000 5.95 3.24 2.99 6.18 5.71

23791000.000 3.11889 247910000.000 5.95 3.24 2.99 6.18 5.71

23798000.000 3.11893 247980000.000 5.95 3.24 2.99 6.19 5.71

23805000.000 3.11898 248050000.000 5.95 3.24 2.99 6.19 5.71

23812000.000 3.11903 248120000.000 5.95 3.24 2.99 6.19 5.71

23819000.000 3.11907 248190000.000 5.95 3.24 2.99 6.19 5.71

23826000.000 3.11912 248260000.000 5.95 3.24 2.99 6.19 5.71

23833000.000 3.11916 248330000.000 5.95 3.24 2.99 6.19 5.71

23840000.000 3.11921 248400000.000 5.95 3.24 2.99 6.19 5.71

23847000.000 3.11926 248470000.000 5.95 3.24 2.99 6.19 5.71

23854000.000 3.11930 248540000.000 5.95 3.24 2.99 6.19 5.71

23861000.000 3.11935 248610000.000 5.95 3.24 2.99 6.19 5.71

23868000.000 3.11939 248680000.000 5.95 3.24 2.99 6.19 5.71

23875000.000 3.11944 248750000.000 5.95 3.24 2.99 6.19 5.71

23882000.000 3.11949 248820000.000 5.95 3.24 2.99 6.19 5.71

23889000.000 3.11953 248890000.000 5.95 3.24 2.99 6.19 5.71

23896000.000 3.11958 248960000.000 5.95 3.24 2.99 6.19 5.71

23903000.000 3.11962 249030000.000 5.95 3.24 2.99 6.19 5.71

23910000.000 3.11967 249100000.000 5.95 3.24 2.99 6.19 5.71

23917000.000 3.11972 249170000.000 5.95 3.24 2.99 6.19 5.71

23924000.000 3.11976 249240000.000 5.95 3.24 2.99 6.19 5.71

23931000.000 3.11981 249310000.000 5.95 3.24 3.00 6.19 5.71

23938000.000 3.11985 249380000.000 5.95 3.24 3.00 6.19 5.71

23945000.000 3.11990 249450000.000 5.95 3.24 3.00 6.19 5.71

23952000.000 3.11994 249520000.000 5.95 3.24 3.00 6.19 5.71

23959000.000 3.11999 249590000.000 5.95 3.24 3.00 6.19 5.71

23966000.000 3.12004 249660000.000 5.95 3.24 3.00 6.19 5.71

23973000.000 3.12008 249730000.000 5.95 3.24 3.00 6.19 5.71

23980000.000 3.12013 249800000.000 5.95 3.24 3.00 6.19 5.71

23987000.000 3.12017 249870000.000 5.95 3.24 3.00 6.19 5.71

23994000.000 3.12022 249940000.000 5.95 3.25 3.00 6.19 5.71

24001000.000 3.12026 250010000.000 5.95 3.25 3.00 6.19 5.71

24008000.000 3.12031 250080000.000 5.95 3.25 3.00 6.19 5.71

24015000.000 3.12036 250150000.000 5.95 3.25 3.00 6.19 5.71

24022000.000 3.12040 250220000.000 5.95 3.25 3.00 6.19 5.71

24029000.000 3.12045 250290000.000 5.95 3.25 3.00 6.19 5.71

24036000.000 3.12049 250360000.000 5.95 3.25 3.00 6.19 5.71

24043000.000 3.12054 250430000.000 5.95 3.25 3.00 6.19 5.71

24050000.000 3.12058 250500000.000 5.95 3.25 3.00 6.19 5.71

24057000.000 3.12063 250570000.000 5.95 3.25 3.00 6.19 5.71

24064000.000 3.12067 250640000.000 5.95 3.25 3.00 6.19 5.71

24071000.000 3.12072 250710000.000 5.95 3.25 3.00 6.19 5.71

24078000.000 3.12077 250780000.000 5.95 3.25 3.00 6.19 5.71

24085000.000 3.12081 250850000.000 5.95 3.25 3.00 6.19 5.71

24092000.000 3.12086 250920000.000 5.95 3.25 3.00 6.19 5.71

24099000.000 3.12090 250990000.000 5.95 3.25 3.00 6.19 5.71

24106000.000 3.12095 251060000.000 5.95 3.25 3.00 6.19 5.71

24113000.000 3.12099 251130000.000 5.95 3.25 3.00 6.19 5.71

24120000.000 3.12104 251200000.000 5.95 3.25 3.00 6.19 5.71

24127000.000 3.12108 251270000.000 5.95 3.25 3.00 6.19 5.71

24134000.000 3.12113 251340000.000 5.95 3.25 3.00 6.19 5.71

24141000.000 3.12117 251410000.000 5.95 3.25 3.00 6.19 5.71

24148000.000 3.12122 251480000.000 5.95 3.25 3.00 6.19 5.71

24155000.000 3.12127 251550000.000 5.95 3.25 3.00 6.19 5.71

24162000.000 3.12131 251620000.000 5.95 3.25 3.00 6.19 5.71

24169000.000 3.12136 251690000.000 5.95 3.25 3.00 6.19 5.71

24176000.000 3.12140 251760000.000 5.95 3.25 3.00 6.19 5.71

24183000.000 3.12145 251830000.000 5.95 3.25 3.00 6.19 5.71

24190000.000 3.12149 251900000.000 5.95 3.25 3.00 6.19 5.71

24197000.000 3.12154 251970000.000 5.95 3.25 3.00 6.19 5.71

24204000.000 3.12158 252040000.000 5.95 3.25 3.00 6.19 5.71

24211000.000 3.12163 252110000.000 5.95 3.25 3.00 6.19 5.71

24218000.000 3.12167 252180000.000 5.95 3.25 3.00 6.19 5.71

24225000.000 3.12172 252250000.000 5.95 3.25 3.00 6.19 5.71

24232000.000 3.12176 252320000.000 5.95 3.25 3.00 6.19 5.71

24239000.000 3.12181 252390000.000 5.95 3.25 3.00 6.19 5.71

24246000.000 3.12185 252460000.000 5.95 3.25 3.00 6.19 5.71

24253000.000 3.12190 252530000.000 5.95 3.25 3.00 6.19 5.71

24260000.000 3.12194 252600000.000 5.95 3.25 3.00 6.19 5.71

24267000.000 3.12199 252670000.000 5.95 3.25 3.00 6.19 5.71

24274000.000 3.12203 252740000.000 5.95 3.25 3.00 6.19 5.71

24281000.000 3.12208 252810000.000 5.95 3.25 3.00 6.19 5.71

24288000.000 3.12213 252880000.000 5.95 3.25 3.00 6.19 5.71

24295000.000 3.12217 252950000.000 5.95 3.25 3.00 6.19 5.71

24302000.000 3.12222 253020000.000 5.95 3.25 3.00 6.19 5.72

24309000.000 3.12226 253090000.000 5.95 3.25 3.00 6.19 5.72

24316000.000 3.12231 253160000.000 5.95 3.25 3.00 6.19 5.72

24323000.000 3.12235 253230000.000 5.95 3.25 3.00 6.19 5.72

24330000.000 3.12240 253300000.000 5.95 3.25 3.00 6.19 5.72

24337000.000 3.12244 253370000.000 5.95 3.25 3.00 6.19 5.72

24344000.000 3.12249 253440000.000 5.95 3.25 3.00 6.19 5.72

24351000.000 3.12253 253510000.000 5.95 3.25 3.00 6.19 5.72

24358000.000 3.12258 253580000.000 5.95 3.25 3.00 6.19 5.72

24365000.000 3.12262 253650000.000 5.95 3.25 3.00 6.19 5.72

24372000.000 3.12267 253720000.000 5.95 3.25 3.00 6.19 5.72

24379000.000 3.12271 253790000.000 5.95 3.25 3.00 6.19 5.72

24386000.000 3.12276 253860000.000 5.95 3.25 3.00 6.19 5.72

24393000.000 3.12280 253930000.000 5.95 3.25 3.00 6.19 5.72

24400000.000 3.12285 254000000.000 5.95 3.25 3.00 6.19 5.72

24407000.000 3.12289 254070000.000 5.95 3.25 3.00 6.19 5.72

24414000.000 3.12294 254140000.000 5.95 3.25 3.00 6.19 5.72

24421000.000 3.12298 254210000.000 5.95 3.25 3.00 6.19 5.72

24428000.000 3.12303 254280000.000 5.95 3.25 3.00 6.19 5.72

24435000.000 3.12307 254350000.000 5.95 3.25 3.00 6.19 5.72

24442000.000 3.12312 254420000.000 5.95 3.25 3.00 6.19 5.72

24449000.000 3.12316 254490000.000 5.95 3.25 3.00 6.19 5.72

24456000.000 3.12320 254560000.000 5.96 3.25 3.00 6.19 5.72

24463000.000 3.12325 254630000.000 5.96 3.25 3.00 6.19 5.72

24470000.000 3.12329 254700000.000 5.96 3.25 3.00 6.19 5.72

24477000.000 3.12334 254770000.000 5.96 3.25 3.00 6.19 5.72

24484000.000 3.12338 254840000.000 5.96 3.25 3.00 6.19 5.72

24491000.000 3.12343 254910000.000 5.96 3.25 3.00 6.19 5.72

24498000.000 3.12347 254980000.000 5.96 3.25 3.00 6.19 5.72

24505000.000 3.12352 255050000.000 5.96 3.25 3.00 6.19 5.72

24512000.000 3.12356 255120000.000 5.96 3.25 3.00 6.19 5.72

24519000.000 3.12361 255190000.000 5.96 3.25 3.00 6.19 5.72

24526000.000 3.12365 255260000.000 5.96 3.25 3.00 6.19 5.72

24533000.000 3.12370 255330000.000 5.96 3.25 3.00 6.19 5.72

24540000.000 3.12374 255400000.000 5.96 3.25 3.00 6.19 5.72

24547000.000 3.12379 255470000.000 5.96 3.25 3.00 6.19 5.72

24554000.000 3.12383 255540000.000 5.96 3.25 3.00 6.19 5.72

24561000.000 3.12388 255610000.000 5.96 3.25 3.00 6.19 5.72

24568000.000 3.12392 255680000.000 5.96 3.25 3.00 6.19 5.72

24575000.000 3.12397 255750000.000 5.96 3.25 3.00 6.19 5.72

24582000.000 3.12401 255820000.000 5.96 3.25 3.00 6.19 5.72

24589000.000 3.12405 255890000.000 5.96 3.25 3.00 6.19 5.72

24596000.000 3.12410 255960000.000 5.96 3.25 3.00 6.19 5.72

24603000.000 3.12414 256030000.000 5.96 3.25 3.00 6.19 5.72

24610000.000 3.12419 256100000.000 5.96 3.25 3.00 6.20 5.72

24617000.000 3.12423 256170000.000 5.96 3.25 3.00 6.20 5.72

24624000.000 3.12428 256240000.000 5.96 3.25 3.00 6.20 5.72

24631000.000 3.12432 256310000.000 5.96 3.25 3.00 6.20 5.72

24638000.000 3.12437 256380000.000 5.96 3.25 3.00 6.20 5.72

24645000.000 3.12441 256450000.000 5.96 3.25 3.00 6.20 5.72

24652000.000 3.12446 256520000.000 5.96 3.25 3.00 6.20 5.72

24659000.000 3.12450 256590000.000 5.96 3.25 3.00 6.20 5.72

24666000.000 3.12454 256660000.000 5.96 3.25 3.00 6.20 5.72

24673000.000 3.12459 256730000.000 5.96 3.25 3.00 6.20 5.72

24680000.000 3.12463 256800000.000 5.96 3.25 3.00 6.20 5.72

24687000.000 3.12468 256870000.000 5.96 3.25 3.00 6.20 5.72

24694000.000 3.12472 256940000.000 5.96 3.25 3.00 6.20 5.72

24701000.000 3.12477 257010000.000 5.96 3.25 3.00 6.20 5.72

24708000.000 3.12481 257080000.000 5.96 3.25 3.00 6.20 5.72

24715000.000 3.12486 257150000.000 5.96 3.25 3.00 6.20 5.72

24722000.000 3.12490 257220000.000 5.96 3.25 3.00 6.20 5.72

24729000.000 3.12494 257290000.000 5.96 3.25 3.00 6.20 5.72

24736000.000 3.12499 257360000.000 5.96 3.25 3.00 6.20 5.72

24743000.000 3.12503 257430000.000 5.96 3.25 3.00 6.20 5.72

24750000.000 3.12508 257500000.000 5.96 3.25 3.00 6.20 5.72

24757000.000 3.12512 257570000.000 5.96 3.25 3.00 6.20 5.72

24764000.000 3.12517 257640000.000 5.96 3.25 3.00 6.20 5.72

24771000.000 3.12521 257710000.000 5.96 3.25 3.00 6.20 5.72

24778000.000 3.12525 257780000.000 5.96 3.25 3.00 6.20 5.72

24785000.000 3.12530 257850000.000 5.96 3.25 3.00 6.20 5.72

24792000.000 3.12534 257920000.000 5.96 3.25 3.00 6.20 5.72

24799000.000 3.12539 257990000.000 5.96 3.25 3.00 6.20 5.72

24806000.000 3.12543 258060000.000 5.96 3.25 3.00 6.20 5.72

24813000.000 3.12548 258130000.000 5.96 3.25 3.00 6.20 5.72

24820000.000 3.12552 258200000.000 5.96 3.25 3.00 6.20 5.72

24827000.000 3.12556 258270000.000 5.96 3.25 3.00 6.20 5.72

24834000.000 3.12561 258340000.000 5.96 3.25 3.00 6.20 5.72

24841000.000 3.12565 258410000.000 5.96 3.25 3.00 6.20 5.72

24848000.000 3.12570 258480000.000 5.96 3.25 3.00 6.20 5.72

24855000.000 3.12574 258550000.000 5.96 3.25 3.00 6.20 5.72

24862000.000 3.12579 258620000.000 5.96 3.25 3.00 6.20 5.72

24869000.000 3.12583 258690000.000 5.96 3.25 3.00 6.20 5.72

24876000.000 3.12587 258760000.000 5.96 3.25 3.00 6.20 5.72

24883000.000 3.12592 258830000.000 5.96 3.25 3.00 6.20 5.72

24890000.000 3.12596 258900000.000 5.96 3.25 3.00 6.20 5.72

24897000.000 3.12601 258970000.000 5.96 3.25 3.00 6.20 5.72

24904000.000 3.12605 259040000.000 5.96 3.25 3.00 6.20 5.72

24911000.000 3.12609 259110000.000 5.96 3.25 3.00 6.20 5.72

24918000.000 3.12614 259180000.000 5.96 3.25 3.00 6.20 5.72

24925000.000 3.12618 259250000.000 5.96 3.25 3.00 6.20 5.72

24932000.000 3.12623 259320000.000 5.96 3.25 3.00 6.20 5.72

24939000.000 3.12627 259390000.000 5.96 3.25 3.00 6.20 5.72

24946000.000 3.12631 259460000.000 5.96 3.25 3.00 6.20 5.72

24953000.000 3.12636 259530000.000 5.96 3.25 3.00 6.20 5.72

24960000.000 3.12640 259600000.000 5.96 3.25 3.00 6.20 5.72

24967000.000 3.12645 259670000.000 5.96 3.25 3.00 6.20 5.72

24974000.000 3.12649 259740000.000 5.96 3.25 3.00 6.20 5.72

24981000.000 3.12653 259810000.000 5.96 3.25 3.00 6.20 5.72

24988000.000 3.12658 259880000.000 5.96 3.25 3.00 6.20 5.72

24995000.000 3.12662 259950000.000 5.96 3.25 3.00 6.20 5.72

25002000.000 3.12667 260020000.000 5.96 3.25 3.00 6.20 5.72

25009000.000 3.12671 260090000.000 5.96 3.25 3.00 6.20 5.72

25016000.000 3.12675 260160000.000 5.96 3.25 3.00 6.20 5.72

25023000.000 3.12680 260230000.000 5.96 3.25 3.00 6.20 5.72

25030000.000 3.12684 260300000.000 5.96 3.25 3.00 6.20 5.72

25037000.000 3.12689 260370000.000 5.96 3.25 3.00 6.20 5.72

25044000.000 3.12693 260440000.000 5.96 3.25 3.00 6.20 5.72

25051000.000 3.12697 260510000.000 5.96 3.25 3.00 6.20 5.72

25058000.000 3.12702 260580000.000 5.96 3.25 3.00 6.20 5.72

25065000.000 3.12706 260650000.000 5.96 3.25 3.00 6.20 5.72

25072000.000 3.12710 260720000.000 5.96 3.25 3.00 6.20 5.72

25079000.000 3.12715 260790000.000 5.96 3.25 3.00 6.20 5.72

25086000.000 3.12719 260860000.000 5.96 3.25 3.00 6.20 5.72

25093000.000 3.12724 260930000.000 5.96 3.25 3.00 6.20 5.72

25100000.000 3.12728 261000000.000 5.96 3.25 3.00 6.20 5.72

25107000.000 3.12732 261070000.000 5.96 3.25 3.00 6.20 5.72

25114000.000 3.12737 261140000.000 5.96 3.25 3.00 6.20 5.72

25121000.000 3.12741 261210000.000 5.96 3.25 3.00 6.20 5.72

25128000.000 3.12745 261280000.000 5.96 3.25 3.00 6.20 5.72

25135000.000 3.12750 261350000.000 5.96 3.25 3.00 6.20 5.72

25142000.000 3.12754 261420000.000 5.96 3.25 3.00 6.20 5.72

25149000.000 3.12759 261490000.000 5.96 3.25 3.00 6.20 5.72

25156000.000 3.12763 261560000.000 5.96 3.25 3.00 6.20 5.72

25163000.000 3.12767 261630000.000 5.96 3.25 3.00 6.20 5.72

25170000.000 3.12772 261700000.000 5.96 3.25 3.00 6.20 5.72

25177000.000 3.12776 261770000.000 5.96 3.25 3.00 6.20 5.72

25184000.000 3.12780 261840000.000 5.96 3.25 3.00 6.20 5.72

25191000.000 3.12785 261910000.000 5.96 3.25 3.00 6.20 5.72

25198000.000 3.12789 261980000.000 5.96 3.25 3.00 6.20 5.73

25205000.000 3.12793 262050000.000 5.96 3.25 3.00 6.20 5.73

25212000.000 3.12798 262120000.000 5.96 3.25 3.00 6.20 5.73

25219000.000 3.12802 262190000.000 5.96 3.25 3.00 6.20 5.73

25226000.000 3.12806 262260000.000 5.96 3.25 3.00 6.20 5.73

25233000.000 3.12811 262330000.000 5.96 3.25 3.00 6.20 5.73

25240000.000 3.12815 262400000.000 5.96 3.25 3.00 6.20 5.73

25247000.000 3.12820 262470000.000 5.96 3.25 3.00 6.20 5.73

25254000.000 3.12824 262540000.000 5.96 3.25 3.00 6.20 5.73

25261000.000 3.12828 262610000.000 5.96 3.25 3.00 6.20 5.73

25268000.000 3.12833 262680000.000 5.96 3.25 3.00 6.20 5.73

25275000.000 3.12837 262750000.000 5.96 3.25 3.00 6.20 5.73

25282000.000 3.12841 262820000.000 5.96 3.25 3.00 6.20 5.73

25289000.000 3.12846 262890000.000 5.96 3.25 3.00 6.20 5.73

25296000.000 3.12850 262960000.000 5.96 3.25 3.00 6.20 5.73

25303000.000 3.12854 263030000.000 5.96 3.25 3.00 6.20 5.73

25310000.000 3.12859 263100000.000 5.96 3.25 3.00 6.20 5.73

25317000.000 3.12863 263170000.000 5.96 3.25 3.00 6.20 5.73

25324000.000 3.12867 263240000.000 5.97 3.25 3.00 6.20 5.73

25331000.000 3.12872 263310000.000 5.97 3.25 3.00 6.20 5.73

25338000.000 3.12876 263380000.000 5.97 3.25 3.00 6.20 5.73

25345000.000 3.12880 263450000.000 5.97 3.25 3.00 6.20 5.73

25352000.000 3.12885 263520000.000 5.97 3.25 3.00 6.20 5.73

25359000.000 3.12889 263590000.000 5.97 3.25 3.00 6.20 5.73

25366000.000 3.12893 263660000.000 5.97 3.25 3.00 6.20 5.73

25373000.000 3.12898 263730000.000 5.97 3.25 3.00 6.20 5.73

25380000.000 3.12902 263800000.000 5.97 3.25 3.00 6.20 5.73

25387000.000 3.12906 263870000.000 5.97 3.25 3.00 6.20 5.73

25394000.000 3.12911 263940000.000 5.97 3.25 3.00 6.20 5.73

25401000.000 3.12915 264010000.000 5.97 3.25 3.00 6.20 5.73

25408000.000 3.12919 264080000.000 5.97 3.25 3.00 6.20 5.73

25415000.000 3.12924 264150000.000 5.97 3.25 3.00 6.20 5.73

25422000.000 3.12928 264220000.000 5.97 3.25 3.00 6.20 5.73

25429000.000 3.12932 264290000.000 5.97 3.25 3.00 6.20 5.73

25436000.000 3.12937 264360000.000 5.97 3.25 3.00 6.20 5.73

25443000.000 3.12941 264430000.000 5.97 3.25 3.00 6.21 5.73

25450000.000 3.12945 264500000.000 5.97 3.25 3.00 6.21 5.73

25457000.000 3.12950 264570000.000 5.97 3.25 3.00 6.21 5.73

25464000.000 3.12954 264640000.000 5.97 3.25 3.00 6.21 5.73

25471000.000 3.12958 264710000.000 5.97 3.25 3.00 6.21 5.73

25478000.000 3.12962 264780000.000 5.97 3.25 3.00 6.21 5.73

25485000.000 3.12967 264850000.000 5.97 3.25 3.00 6.21 5.73

25492000.000 3.12971 264920000.000 5.97 3.25 3.00 6.21 5.73

25499000.000 3.12975 264990000.000 5.97 3.25 3.00 6.21 5.73

25506000.000 3.12980 265060000.000 5.97 3.25 3.00 6.21 5.73

25513000.000 3.12984 265130000.000 5.97 3.26 3.00 6.21 5.73

25520000.000 3.12988 265200000.000 5.97 3.26 3.00 6.21 5.73

25527000.000 3.12993 265270000.000 5.97 3.26 3.00 6.21 5.73

25534000.000 3.12997 265340000.000 5.97 3.26 3.00 6.21 5.73

25541000.000 3.13001 265410000.000 5.97 3.26 3.00 6.21 5.73

25548000.000 3.13006 265480000.000 5.97 3.26 3.00 6.21 5.73

25555000.000 3.13010 265550000.000 5.97 3.26 3.00 6.21 5.73

25562000.000 3.13014 265620000.000 5.97 3.26 3.00 6.21 5.73

25569000.000 3.13018 265690000.000 5.97 3.26 3.00 6.21 5.73

25576000.000 3.13023 265760000.000 5.97 3.26 3.01 6.21 5.73

25583000.000 3.13027 265830000.000 5.97 3.26 3.01 6.21 5.73

25590000.000 3.13031 265900000.000 5.97 3.26 3.01 6.21 5.73

25597000.000 3.13036 265970000.000 5.97 3.26 3.01 6.21 5.73

25604000.000 3.13040 266040000.000 5.97 3.26 3.01 6.21 5.73

25611000.000 3.13044 266110000.000 5.97 3.26 3.01 6.21 5.73

25618000.000 3.13049 266180000.000 5.97 3.26 3.01 6.21 5.73

25625000.000 3.13053 266250000.000 5.97 3.26 3.01 6.21 5.73

25632000.000 3.13057 266320000.000 5.97 3.26 3.01 6.21 5.73

25639000.000 3.13061 266390000.000 5.97 3.26 3.01 6.21 5.73

25646000.000 3.13066 266460000.000 5.97 3.26 3.01 6.21 5.73

25653000.000 3.13070 266530000.000 5.97 3.26 3.01 6.21 5.73

25660000.000 3.13074 266600000.000 5.97 3.26 3.01 6.21 5.73

25667000.000 3.13079 266670000.000 5.97 3.26 3.01 6.21 5.73

25674000.000 3.13083 266740000.000 5.97 3.26 3.01 6.21 5.73

25681000.000 3.13087 266810000.000 5.97 3.26 3.01 6.21 5.73

25688000.000 3.13091 266880000.000 5.97 3.26 3.01 6.21 5.73

25695000.000 3.13096 266950000.000 5.97 3.26 3.01 6.21 5.73

25702000.000 3.13100 267020000.000 5.97 3.26 3.01 6.21 5.73

25709000.000 3.13104 267090000.000 5.97 3.26 3.01 6.21 5.73

25716000.000 3.13108 267160000.000 5.97 3.26 3.01 6.21 5.73

25723000.000 3.13113 267230000.000 5.97 3.26 3.01 6.21 5.73

25730000.000 3.13117 267300000.000 5.97 3.26 3.01 6.21 5.73

25737000.000 3.13121 267370000.000 5.97 3.26 3.01 6.21 5.73

25744000.000 3.13126 267440000.000 5.97 3.26 3.01 6.21 5.73

25751000.000 3.13130 267510000.000 5.97 3.26 3.01 6.21 5.73

25758000.000 3.13134 267580000.000 5.97 3.26 3.01 6.21 5.73

25765000.000 3.13138 267650000.000 5.97 3.26 3.01 6.21 5.73

25772000.000 3.13143 267720000.000 5.97 3.26 3.01 6.21 5.73

25779000.000 3.13147 267790000.000 5.97 3.26 3.01 6.21 5.73

25786000.000 3.13151 267860000.000 5.97 3.26 3.01 6.21 5.73

25793000.000 3.13155 267930000.000 5.97 3.26 3.01 6.21 5.73

25800000.000 3.13160 268000000.000 5.97 3.26 3.01 6.21 5.73

25807000.000 3.13164 268070000.000 5.97 3.26 3.01 6.21 5.73

25814000.000 3.13168 268140000.000 5.97 3.26 3.01 6.21 5.73

25821000.000 3.13172 268210000.000 5.97 3.26 3.01 6.21 5.73

25828000.000 3.13177 268280000.000 5.97 3.26 3.01 6.21 5.73

25835000.000 3.13181 268350000.000 5.97 3.26 3.01 6.21 5.73

25842000.000 3.13185 268420000.000 5.97 3.26 3.01 6.21 5.73

25849000.000 3.13190 268490000.000 5.97 3.26 3.01 6.21 5.73

25856000.000 3.13194 268560000.000 5.97 3.26 3.01 6.21 5.73

25863000.000 3.13198 268630000.000 5.97 3.26 3.01 6.21 5.73

25870000.000 3.13202 268700000.000 5.97 3.26 3.01 6.21 5.73

25877000.000 3.13207 268770000.000 5.97 3.26 3.01 6.21 5.73

25884000.000 3.13211 268840000.000 5.97 3.26 3.01 6.21 5.73

25891000.000 3.13215 268910000.000 5.97 3.26 3.01 6.21 5.73

25898000.000 3.13219 268980000.000 5.97 3.26 3.01 6.21 5.73

25905000.000 3.13224 269050000.000 5.97 3.26 3.01 6.21 5.73

25912000.000 3.13228 269120000.000 5.97 3.26 3.01 6.21 5.73

25919000.000 3.13232 269190000.000 5.97 3.26 3.01 6.21 5.73

25926000.000 3.13236 269260000.000 5.97 3.26 3.01 6.21 5.73

25933000.000 3.13240 269330000.000 5.97 3.26 3.01 6.21 5.73

25940000.000 3.13245 269400000.000 5.97 3.26 3.01 6.21 5.73

25947000.000 3.13249 269470000.000 5.97 3.26 3.01 6.21 5.73

25954000.000 3.13253 269540000.000 5.97 3.26 3.01 6.21 5.73

25961000.000 3.13257 269610000.000 5.97 3.26 3.01 6.21 5.73

25968000.000 3.13262 269680000.000 5.97 3.26 3.01 6.21 5.73

25975000.000 3.13266 269750000.000 5.97 3.26 3.01 6.21 5.73

25982000.000 3.13270 269820000.000 5.97 3.26 3.01 6.21 5.73

25989000.000 3.13274 269890000.000 5.97 3.26 3.01 6.21 5.73

25996000.000 3.13279 269960000.000 5.97 3.26 3.01 6.21 5.73

26003000.000 3.13283 270030000.000 5.97 3.26 3.01 6.21 5.73

26010000.000 3.13287 270100000.000 5.97 3.26 3.01 6.21 5.73

26017000.000 3.13291 270170000.000 5.97 3.26 3.01 6.21 5.73

26024000.000 3.13296 270240000.000 5.97 3.26 3.01 6.21 5.73

26031000.000 3.13300 270310000.000 5.97 3.26 3.01 6.21 5.73

26038000.000 3.13304 270380000.000 5.97 3.26 3.01 6.21 5.73

26045000.000 3.13308 270450000.000 5.97 3.26 3.01 6.21 5.73

26052000.000 3.13312 270520000.000 5.97 3.26 3.01 6.21 5.73

26059000.000 3.13317 270590000.000 5.97 3.26 3.01 6.21 5.73

26066000.000 3.13321 270660000.000 5.97 3.26 3.01 6.21 5.73

26073000.000 3.13325 270730000.000 5.97 3.26 3.01 6.21 5.73

26080000.000 3.13329 270800000.000 5.97 3.26 3.01 6.21 5.73

26087000.000 3.13334 270870000.000 5.97 3.26 3.01 6.21 5.73

26094000.000 3.13338 270940000.000 5.97 3.26 3.01 6.21 5.73

26101000.000 3.13342 271010000.000 5.97 3.26 3.01 6.21 5.73

26108000.000 3.13346 271080000.000 5.97 3.26 3.01 6.21 5.73

26115000.000 3.13350 271150000.000 5.97 3.26 3.01 6.21 5.73

26122000.000 3.13355 271220000.000 5.97 3.26 3.01 6.21 5.74

26129000.000 3.13359 271290000.000 5.97 3.26 3.01 6.21 5.74

26136000.000 3.13363 271360000.000 5.97 3.26 3.01 6.21 5.74

26143000.000 3.13367 271430000.000 5.97 3.26 3.01 6.21 5.74

26150000.000 3.13371 271500000.000 5.97 3.26 3.01 6.21 5.74

26157000.000 3.13376 271570000.000 5.97 3.26 3.01 6.21 5.74

26164000.000 3.13380 271640000.000 5.97 3.26 3.01 6.21 5.74

26171000.000 3.13384 271710000.000 5.97 3.26 3.01 6.21 5.74

26178000.000 3.13388 271780000.000 5.97 3.26 3.01 6.21 5.74

26185000.000 3.13392 271850000.000 5.97 3.26 3.01 6.21 5.74

26192000.000 3.13397 271920000.000 5.97 3.26 3.01 6.21 5.74

26199000.000 3.13401 271990000.000 5.97 3.26 3.01 6.21 5.74

26206000.000 3.13405 272060000.000 5.97 3.26 3.01 6.21 5.74

26213000.000 3.13409 272130000.000 5.97 3.26 3.01 6.21 5.74

26220000.000 3.13413 272200000.000 5.98 3.26 3.01 6.21 5.74

26227000.000 3.13418 272270000.000 5.98 3.26 3.01 6.21 5.74

26234000.000 3.13422 272340000.000 5.98 3.26 3.01 6.21 5.74

26241000.000 3.13426 272410000.000 5.98 3.26 3.01 6.21 5.74

26248000.000 3.13430 272480000.000 5.98 3.26 3.01 6.21 5.74

26255000.000 3.13434 272550000.000 5.98 3.26 3.01 6.21 5.74

26262000.000 3.13439 272620000.000 5.98 3.26 3.01 6.21 5.74

26269000.000 3.13443 272690000.000 5.98 3.26 3.01 6.21 5.74

26276000.000 3.13447 272760000.000 5.98 3.26 3.01 6.21 5.74

26283000.000 3.13451 272830000.000 5.98 3.26 3.01 6.21 5.74

26290000.000 3.13455 272900000.000 5.98 3.26 3.01 6.21 5.74

26297000.000 3.13460 272970000.000 5.98 3.26 3.01 6.21 5.74

26304000.000 3.13464 273040000.000 5.98 3.26 3.01 6.22 5.74

26311000.000 3.13468 273110000.000 5.98 3.26 3.01 6.22 5.74

26318000.000 3.13472 273180000.000 5.98 3.26 3.01 6.22 5.74

26325000.000 3.13476 273250000.000 5.98 3.26 3.01 6.22 5.74

26332000.000 3.13480 273320000.000 5.98 3.26 3.01 6.22 5.74

26339000.000 3.13485 273390000.000 5.98 3.26 3.01 6.22 5.74

26346000.000 3.13489 273460000.000 5.98 3.26 3.01 6.22 5.74

26353000.000 3.13493 273530000.000 5.98 3.26 3.01 6.22 5.74

26360000.000 3.13497 273600000.000 5.98 3.26 3.01 6.22 5.74

26367000.000 3.13501 273670000.000 5.98 3.26 3.01 6.22 5.74

26374000.000 3.13506 273740000.000 5.98 3.26 3.01 6.22 5.74

26381000.000 3.13510 273810000.000 5.98 3.26 3.01 6.22 5.74

26388000.000 3.13514 273880000.000 5.98 3.26 3.01 6.22 5.74

26395000.000 3.13518 273950000.000 5.98 3.26 3.01 6.22 5.74

26402000.000 3.13522 274020000.000 5.98 3.26 3.01 6.22 5.74

26409000.000 3.13526 274090000.000 5.98 3.26 3.01 6.22 5.74

26416000.000 3.13531 274160000.000 5.98 3.26 3.01 6.22 5.74

26423000.000 3.13535 274230000.000 5.98 3.26 3.01 6.22 5.74

26430000.000 3.13539 274300000.000 5.98 3.26 3.01 6.22 5.74

26437000.000 3.13543 274370000.000 5.98 3.26 3.01 6.22 5.74

26444000.000 3.13547 274440000.000 5.98 3.26 3.01 6.22 5.74

26451000.000 3.13551 274510000.000 5.98 3.26 3.01 6.22 5.74

26458000.000 3.13556 274580000.000 5.98 3.26 3.01 6.22 5.74

26465000.000 3.13560 274650000.000 5.98 3.26 3.01 6.22 5.74

26472000.000 3.13564 274720000.000 5.98 3.26 3.01 6.22 5.74

26479000.000 3.13568 274790000.000 5.98 3.26 3.01 6.22 5.74

26486000.000 3.13572 274860000.000 5.98 3.26 3.01 6.22 5.74

26493000.000 3.13576 274930000.000 5.98 3.26 3.01 6.22 5.74

26500000.000 3.13581 275000000.000 5.98 3.26 3.01 6.22 5.74

26507000.000 3.13585 275070000.000 5.98 3.26 3.01 6.22 5.74

26514000.000 3.13589 275140000.000 5.98 3.26 3.01 6.22 5.74

26521000.000 3.13593 275210000.000 5.98 3.26 3.01 6.22 5.74

26528000.000 3.13597 275280000.000 5.98 3.26 3.01 6.22 5.74

26535000.000 3.13601 275350000.000 5.98 3.26 3.01 6.22 5.74

26542000.000 3.13605 275420000.000 5.98 3.26 3.01 6.22 5.74

26549000.000 3.13610 275490000.000 5.98 3.26 3.01 6.22 5.74

26556000.000 3.13614 275560000.000 5.98 3.26 3.01 6.22 5.74

26563000.000 3.13618 275630000.000 5.98 3.26 3.01 6.22 5.74

26570000.000 3.13622 275700000.000 5.98 3.26 3.01 6.22 5.74

26577000.000 3.13626 275770000.000 5.98 3.26 3.01 6.22 5.74

26584000.000 3.13630 275840000.000 5.98 3.26 3.01 6.22 5.74

26591000.000 3.13634 275910000.000 5.98 3.26 3.01 6.22 5.74

26598000.000 3.13639 275980000.000 5.98 3.26 3.01 6.22 5.74

26605000.000 3.13643 276050000.000 5.98 3.26 3.01 6.22 5.74

26612000.000 3.13647 276120000.000 5.98 3.26 3.01 6.22 5.74

26619000.000 3.13651 276190000.000 5.98 3.26 3.01 6.22 5.74

26626000.000 3.13655 276260000.000 5.98 3.26 3.01 6.22 5.74

26633000.000 3.13659 276330000.000 5.98 3.26 3.01 6.22 5.74

26640000.000 3.13663 276400000.000 5.98 3.26 3.01 6.22 5.74

26647000.000 3.13668 276470000.000 5.98 3.26 3.01 6.22 5.74

26654000.000 3.13672 276540000.000 5.98 3.26 3.01 6.22 5.74

26661000.000 3.13676 276610000.000 5.98 3.26 3.01 6.22 5.74

26668000.000 3.13680 276680000.000 5.98 3.26 3.01 6.22 5.74

26675000.000 3.13684 276750000.000 5.98 3.26 3.01 6.22 5.74

26682000.000 3.13688 276820000.000 5.98 3.26 3.01 6.22 5.74

26689000.000 3.13692 276890000.000 5.98 3.26 3.01 6.22 5.74

26696000.000 3.13696 276960000.000 5.98 3.26 3.01 6.22 5.74

26703000.000 3.13701 277030000.000 5.98 3.26 3.01 6.22 5.74

26710000.000 3.13705 277100000.000 5.98 3.26 3.01 6.22 5.74

26717000.000 3.13709 277170000.000 5.98 3.26 3.01 6.22 5.74

26724000.000 3.13713 277240000.000 5.98 3.26 3.01 6.22 5.74

26731000.000 3.13717 277310000.000 5.98 3.26 3.01 6.22 5.74

26738000.000 3.13721 277380000.000 5.98 3.26 3.01 6.22 5.74

26745000.000 3.13725 277450000.000 5.98 3.26 3.01 6.22 5.74

26752000.000 3.13729 277520000.000 5.98 3.26 3.01 6.22 5.74

26759000.000 3.13734 277590000.000 5.98 3.26 3.01 6.22 5.74

26766000.000 3.13738 277660000.000 5.98 3.26 3.01 6.22 5.74

26773000.000 3.13742 277730000.000 5.98 3.26 3.01 6.22 5.74

26780000.000 3.13746 277800000.000 5.98 3.26 3.01 6.22 5.74

26787000.000 3.13750 277870000.000 5.98 3.26 3.01 6.22 5.74

26794000.000 3.13754 277940000.000 5.98 3.26 3.01 6.22 5.74

26801000.000 3.13758 278010000.000 5.98 3.26 3.01 6.22 5.74

26808000.000 3.13762 278080000.000 5.98 3.26 3.01 6.22 5.74

26815000.000 3.13766 278150000.000 5.98 3.26 3.01 6.22 5.74

26822000.000 3.13771 278220000.000 5.98 3.26 3.01 6.22 5.74

26829000.000 3.13775 278290000.000 5.98 3.26 3.01 6.22 5.74

26836000.000 3.13779 278360000.000 5.98 3.26 3.01 6.22 5.74

26843000.000 3.13783 278430000.000 5.98 3.26 3.01 6.22 5.74

26850000.000 3.13787 278500000.000 5.98 3.26 3.01 6.22 5.74

26857000.000 3.13791 278570000.000 5.98 3.26 3.01 6.22 5.74

26864000.000 3.13795 278640000.000 5.98 3.26 3.01 6.22 5.74

26871000.000 3.13799 278710000.000 5.98 3.26 3.01 6.22 5.74

26878000.000 3.13803 278780000.000 5.98 3.26 3.01 6.22 5.74

26885000.000 3.13807 278850000.000 5.98 3.26 3.01 6.22 5.74

26892000.000 3.13812 278920000.000 5.98 3.26 3.01 6.22 5.74

26899000.000 3.13816 278990000.000 5.98 3.26 3.01 6.22 5.74

26906000.000 3.13820 279060000.000 5.98 3.26 3.01 6.22 5.74

26913000.000 3.13824 279130000.000 5.98 3.26 3.01 6.22 5.74

26920000.000 3.13828 279200000.000 5.98 3.26 3.01 6.22 5.74

26927000.000 3.13832 279270000.000 5.98 3.26 3.01 6.22 5.74

26934000.000 3.13836 279340000.000 5.98 3.26 3.01 6.22 5.74

26941000.000 3.13840 279410000.000 5.98 3.26 3.01 6.22 5.74

26948000.000 3.13844 279480000.000 5.98 3.26 3.01 6.22 5.74

26955000.000 3.13848 279550000.000 5.98 3.26 3.01 6.22 5.74

26962000.000 3.13852 279620000.000 5.98 3.26 3.01 6.22 5.74

26969000.000 3.13857 279690000.000 5.98 3.26 3.01 6.22 5.74

26976000.000 3.13861 279760000.000 5.98 3.26 3.01 6.22 5.74

26983000.000 3.13865 279830000.000 5.98 3.26 3.01 6.22 5.74

26990000.000 3.13869 279900000.000 5.98 3.26 3.01 6.22 5.74

26997000.000 3.13873 279970000.000 5.98 3.26 3.01 6.22 5.74

27004000.000 3.13877 280040000.000 5.98 3.26 3.01 6.22 5.74

27011000.000 3.13881 280110000.000 5.98 3.26 3.01 6.22 5.74

27018000.000 3.13885 280180000.000 5.98 3.26 3.01 6.22 5.74

27025000.000 3.13889 280250000.000 5.98 3.26 3.01 6.22 5.74

27032000.000 3.13893 280320000.000 5.98 3.26 3.01 6.22 5.74

27039000.000 3.13897 280390000.000 5.98 3.26 3.01 6.22 5.74

27046000.000 3.13901 280460000.000 5.98 3.26 3.01 6.22 5.74

27053000.000 3.13906 280530000.000 5.98 3.26 3.01 6.22 5.74

27060000.000 3.13910 280600000.000 5.98 3.26 3.01 6.22 5.74

27067000.000 3.13914 280670000.000 5.98 3.26 3.01 6.22 5.74

27074000.000 3.13918 280740000.000 5.98 3.26 3.01 6.22 5.74

27081000.000 3.13922 280810000.000 5.98 3.26 3.01 6.22 5.75

27088000.000 3.13926 280880000.000 5.98 3.26 3.01 6.22 5.75

27095000.000 3.13930 280950000.000 5.98 3.26 3.01 6.22 5.75

27102000.000 3.13934 281020000.000 5.98 3.26 3.01 6.22 5.75

27109000.000 3.13938 281090000.000 5.98 3.26 3.01 6.22 5.75

27116000.000 3.13942 281160000.000 5.98 3.26 3.01 6.22 5.75

27123000.000 3.13946 281230000.000 5.98 3.27 3.01 6.22 5.75

27130000.000 3.13950 281300000.000 5.98 3.27 3.01 6.22 5.75

27137000.000 3.13954 281370000.000 5.98 3.27 3.01 6.22 5.75

27144000.000 3.13958 281440000.000 5.99 3.27 3.01 6.22 5.75

27151000.000 3.13962 281510000.000 5.99 3.27 3.01 6.22 5.75

27158000.000 3.13967 281580000.000 5.99 3.27 3.01 6.22 5.75

27165000.000 3.13971 281650000.000 5.99 3.27 3.01 6.22 5.75

27172000.000 3.13975 281720000.000 5.99 3.27 3.01 6.22 5.75

27179000.000 3.13979 281790000.000 5.99 3.27 3.01 6.22 5.75

27186000.000 3.13983 281860000.000 5.99 3.27 3.01 6.22 5.75

27193000.000 3.13987 281930000.000 5.99 3.27 3.01 6.23 5.75

27200000.000 3.13991 282000000.000 5.99 3.27 3.01 6.23 5.75

27207000.000 3.13995 282070000.000 5.99 3.27 3.01 6.23 5.75

27214000.000 3.13999 282140000.000 5.99 3.27 3.01 6.23 5.75

27221000.000 3.14003 282210000.000 5.99 3.27 3.01 6.23 5.75

27228000.000 3.14007 282280000.000 5.99 3.27 3.01 6.23 5.75

27235000.000 3.14011 282350000.000 5.99 3.27 3.01 6.23 5.75

27242000.000 3.14015 282420000.000 5.99 3.27 3.01 6.23 5.75

27249000.000 3.14019 282490000.000 5.99 3.27 3.01 6.23 5.75

27256000.000 3.14023 282560000.000 5.99 3.27 3.01 6.23 5.75

27263000.000 3.14027 282630000.000 5.99 3.27 3.01 6.23 5.75

27270000.000 3.14031 282700000.000 5.99 3.27 3.01 6.23 5.75

27277000.000 3.14035 282770000.000 5.99 3.27 3.01 6.23 5.75

27284000.000 3.14039 282840000.000 5.99 3.27 3.01 6.23 5.75

27291000.000 3.14043 282910000.000 5.99 3.27 3.01 6.23 5.75

27298000.000 3.14048 282980000.000 5.99 3.27 3.01 6.23 5.75

27305000.000 3.14052 283050000.000 5.99 3.27 3.01 6.23 5.75

27312000.000 3.14056 283120000.000 5.99 3.27 3.01 6.23 5.75

27319000.000 3.14060 283190000.000 5.99 3.27 3.01 6.23 5.75

27326000.000 3.14064 283260000.000 5.99 3.27 3.02 6.23 5.75

27333000.000 3.14068 283330000.000 5.99 3.27 3.02 6.23 5.75

27340000.000 3.14072 283400000.000 5.99 3.27 3.02 6.23 5.75

27347000.000 3.14076 283470000.000 5.99 3.27 3.02 6.23 5.75

27354000.000 3.14080 283540000.000 5.99 3.27 3.02 6.23 5.75

27361000.000 3.14084 283610000.000 5.99 3.27 3.02 6.23 5.75

27368000.000 3.14088 283680000.000 5.99 3.27 3.02 6.23 5.75

27375000.000 3.14092 283750000.000 5.99 3.27 3.02 6.23 5.75

27382000.000 3.14096 283820000.000 5.99 3.27 3.02 6.23 5.75

27389000.000 3.14100 283890000.000 5.99 3.27 3.02 6.23 5.75

27396000.000 3.14104 283960000.000 5.99 3.27 3.02 6.23 5.75

27403000.000 3.14108 284030000.000 5.99 3.27 3.02 6.23 5.75

27410000.000 3.14112 284100000.000 5.99 3.27 3.02 6.23 5.75

27417000.000 3.14116 284170000.000 5.99 3.27 3.02 6.23 5.75

27424000.000 3.14120 284240000.000 5.99 3.27 3.02 6.23 5.75

27431000.000 3.14124 284310000.000 5.99 3.27 3.02 6.23 5.75

27438000.000 3.14128 284380000.000 5.99 3.27 3.02 6.23 5.75

27445000.000 3.14132 284450000.000 5.99 3.27 3.02 6.23 5.75

27452000.000 3.14136 284520000.000 5.99 3.27 3.02 6.23 5.75

27459000.000 3.14140 284590000.000 5.99 3.27 3.02 6.23 5.75

27466000.000 3.14144 284660000.000 5.99 3.27 3.02 6.23 5.75

27473000.000 3.14148 284730000.000 5.99 3.27 3.02 6.23 5.75

27480000.000 3.14152 284800000.000 5.99 3.27 3.02 6.23 5.75

27487000.000 3.14156 284870000.000 5.99 3.27 3.02 6.23 5.75

27494000.000 3.14160 284940000.000 5.99 3.27 3.02 6.23 5.75

27501000.000 3.14164 285010000.000 5.99 3.27 3.02 6.23 5.75

27508000.000 3.14168 285080000.000 5.99 3.27 3.02 6.23 5.75

27515000.000 3.14172 285150000.000 5.99 3.27 3.02 6.23 5.75

27522000.000 3.14176 285220000.000 5.99 3.27 3.02 6.23 5.75

27529000.000 3.14180 285290000.000 5.99 3.27 3.02 6.23 5.75

27536000.000 3.14184 285360000.000 5.99 3.27 3.02 6.23 5.75

27543000.000 3.14188 285430000.000 5.99 3.27 3.02 6.23 5.75

27550000.000 3.14192 285500000.000 5.99 3.27 3.02 6.23 5.75

27557000.000 3.14196 285570000.000 5.99 3.27 3.02 6.23 5.75

27564000.000 3.14200 285640000.000 5.99 3.27 3.02 6.23 5.75

27571000.000 3.14204 285710000.000 5.99 3.27 3.02 6.23 5.75

27578000.000 3.14208 285780000.000 5.99 3.27 3.02 6.23 5.75

27585000.000 3.14212 285850000.000 5.99 3.27 3.02 6.23 5.75

27592000.000 3.14216 285920000.000 5.99 3.27 3.02 6.23 5.75

27599000.000 3.14220 285990000.000 5.99 3.27 3.02 6.23 5.75

27606000.000 3.14224 286060000.000 5.99 3.27 3.02 6.23 5.75

27613000.000 3.14228 286130000.000 5.99 3.27 3.02 6.23 5.75

27620000.000 3.14232 286200000.000 5.99 3.27 3.02 6.23 5.75

27627000.000 3.14236 286270000.000 5.99 3.27 3.02 6.23 5.75

27634000.000 3.14240 286340000.000 5.99 3.27 3.02 6.23 5.75

27641000.000 3.14244 286410000.000 5.99 3.27 3.02 6.23 5.75

27648000.000 3.14248 286480000.000 5.99 3.27 3.02 6.23 5.75

27655000.000 3.14252 286550000.000 5.99 3.27 3.02 6.23 5.75

27662000.000 3.14256 286620000.000 5.99 3.27 3.02 6.23 5.75

27669000.000 3.14260 286690000.000 5.99 3.27 3.02 6.23 5.75

27676000.000 3.14264 286760000.000 5.99 3.27 3.02 6.23 5.75

27683000.000 3.14268 286830000.000 5.99 3.27 3.02 6.23 5.75

27690000.000 3.14272 286900000.000 5.99 3.27 3.02 6.23 5.75

27697000.000 3.14276 286970000.000 5.99 3.27 3.02 6.23 5.75

27704000.000 3.14280 287040000.000 5.99 3.27 3.02 6.23 5.75

27711000.000 3.14284 287110000.000 5.99 3.27 3.02 6.23 5.75

27718000.000 3.14288 287180000.000 5.99 3.27 3.02 6.23 5.75

27725000.000 3.14292 287250000.000 5.99 3.27 3.02 6.23 5.75

27732000.000 3.14296 287320000.000 5.99 3.27 3.02 6.23 5.75

27739000.000 3.14300 287390000.000 5.99 3.27 3.02 6.23 5.75

27746000.000 3.14304 287460000.000 5.99 3.27 3.02 6.23 5.75

27753000.000 3.14308 287530000.000 5.99 3.27 3.02 6.23 5.75

27760000.000 3.14312 287600000.000 5.99 3.27 3.02 6.23 5.75

27767000.000 3.14316 287670000.000 5.99 3.27 3.02 6.23 5.75

27774000.000 3.14320 287740000.000 5.99 3.27 3.02 6.23 5.75

27781000.000 3.14324 287810000.000 5.99 3.27 3.02 6.23 5.75

27788000.000 3.14328 287880000.000 5.99 3.27 3.02 6.23 5.75

27795000.000 3.14332 287950000.000 5.99 3.27 3.02 6.23 5.75

27802000.000 3.14336 288020000.000 5.99 3.27 3.02 6.23 5.75

27809000.000 3.14340 288090000.000 5.99 3.27 3.02 6.23 5.75

27816000.000 3.14344 288160000.000 5.99 3.27 3.02 6.23 5.75

27823000.000 3.14348 288230000.000 5.99 3.27 3.02 6.23 5.75

27830000.000 3.14352 288300000.000 5.99 3.27 3.02 6.23 5.75

27837000.000 3.14356 288370000.000 5.99 3.27 3.02 6.23 5.75

27844000.000 3.14360 288440000.000 5.99 3.27 3.02 6.23 5.75

27851000.000 3.14364 288510000.000 5.99 3.27 3.02 6.23 5.75

27858000.000 3.14368 288580000.000 5.99 3.27 3.02 6.23 5.75

27865000.000 3.14372 288650000.000 5.99 3.27 3.02 6.23 5.75

27872000.000 3.14375 288720000.000 5.99 3.27 3.02 6.23 5.75

27879000.000 3.14379 288790000.000 5.99 3.27 3.02 6.23 5.75

27886000.000 3.14383 288860000.000 5.99 3.27 3.02 6.23 5.75

27893000.000 3.14387 288930000.000 5.99 3.27 3.02 6.23 5.75

27900000.000 3.14391 289000000.000 5.99 3.27 3.02 6.23 5.75

27907000.000 3.14395 289070000.000 5.99 3.27 3.02 6.23 5.75

27914000.000 3.14399 289140000.000 5.99 3.27 3.02 6.23 5.75

27921000.000 3.14403 289210000.000 5.99 3.27 3.02 6.23 5.75

27928000.000 3.14407 289280000.000 5.99 3.27 3.02 6.23 5.75

27935000.000 3.14411 289350000.000 5.99 3.27 3.02 6.23 5.75

27942000.000 3.14415 289420000.000 5.99 3.27 3.02 6.23 5.75

27949000.000 3.14419 289490000.000 5.99 3.27 3.02 6.23 5.75

27956000.000 3.14423 289560000.000 5.99 3.27 3.02 6.23 5.75

27963000.000 3.14427 289630000.000 5.99 3.27 3.02 6.23 5.75

27970000.000 3.14431 289700000.000 5.99 3.27 3.02 6.23 5.75

27977000.000 3.14435 289770000.000 5.99 3.27 3.02 6.23 5.75

27984000.000 3.14439 289840000.000 5.99 3.27 3.02 6.23 5.75

27991000.000 3.14443 289910000.000 5.99 3.27 3.02 6.23 5.75

27998000.000 3.14447 289980000.000 5.99 3.27 3.02 6.23 5.75

28005000.000 3.14451 290050000.000 5.99 3.27 3.02 6.23 5.75

28012000.000 3.14455 290120000.000 5.99 3.27 3.02 6.23 5.75

28019000.000 3.14458 290190000.000 5.99 3.27 3.02 6.23 5.75

28026000.000 3.14462 290260000.000 5.99 3.27 3.02 6.23 5.75

28033000.000 3.14466 290330000.000 5.99 3.27 3.02 6.23 5.75

28040000.000 3.14470 290400000.000 5.99 3.27 3.02 6.23 5.75

28047000.000 3.14474 290470000.000 5.99 3.27 3.02 6.23 5.75

28054000.000 3.14478 290540000.000 5.99 3.27 3.02 6.23 5.75

28061000.000 3.14482 290610000.000 5.99 3.27 3.02 6.23 5.75

28068000.000 3.14486 290680000.000 5.99 3.27 3.02 6.23 5.75

28075000.000 3.14490 290750000.000 5.99 3.27 3.02 6.23 5.76

28082000.000 3.14494 290820000.000 5.99 3.27 3.02 6.23 5.76

28089000.000 3.14498 290890000.000 5.99 3.27 3.02 6.23 5.76

28096000.000 3.14502 290960000.000 6.00 3.27 3.02 6.23 5.76

28103000.000 3.14506 291030000.000 6.00 3.27 3.02 6.23 5.76

28110000.000 3.14510 291100000.000 6.00 3.27 3.02 6.24 5.76

28117000.000 3.14514 291170000.000 6.00 3.27 3.02 6.24 5.76

28124000.000 3.14517 291240000.000 6.00 3.27 3.02 6.24 5.76

28131000.000 3.14521 291310000.000 6.00 3.27 3.02 6.24 5.76

28138000.000 3.14525 291380000.000 6.00 3.27 3.02 6.24 5.76

28145000.000 3.14529 291450000.000 6.00 3.27 3.02 6.24 5.76

28152000.000 3.14533 291520000.000 6.00 3.27 3.02 6.24 5.76

28159000.000 3.14537 291590000.000 6.00 3.27 3.02 6.24 5.76

28166000.000 3.14541 291660000.000 6.00 3.27 3.02 6.24 5.76

28173000.000 3.14545 291730000.000 6.00 3.27 3.02 6.24 5.76

28180000.000 3.14549 291800000.000 6.00 3.27 3.02 6.24 5.76

28187000.000 3.14553 291870000.000 6.00 3.27 3.02 6.24 5.76

28194000.000 3.14557 291940000.000 6.00 3.27 3.02 6.24 5.76

28201000.000 3.14561 292010000.000 6.00 3.27 3.02 6.24 5.76

28208000.000 3.14565 292080000.000 6.00 3.27 3.02 6.24 5.76

28215000.000 3.14568 292150000.000 6.00 3.27 3.02 6.24 5.76

28222000.000 3.14572 292220000.000 6.00 3.27 3.02 6.24 5.76

28229000.000 3.14576 292290000.000 6.00 3.27 3.02 6.24 5.76

28236000.000 3.14580 292360000.000 6.00 3.27 3.02 6.24 5.76

28243000.000 3.14584 292430000.000 6.00 3.27 3.02 6.24 5.76

28250000.000 3.14588 292500000.000 6.00 3.27 3.02 6.24 5.76

28257000.000 3.14592 292570000.000 6.00 3.27 3.02 6.24 5.76

28264000.000 3.14596 292640000.000 6.00 3.27 3.02 6.24 5.76

28271000.000 3.14600 292710000.000 6.00 3.27 3.02 6.24 5.76

28278000.000 3.14604 292780000.000 6.00 3.27 3.02 6.24 5.76

28285000.000 3.14608 292850000.000 6.00 3.27 3.02 6.24 5.76

28292000.000 3.14611 292920000.000 6.00 3.27 3.02 6.24 5.76

28299000.000 3.14615 292990000.000 6.00 3.27 3.02 6.24 5.76

28306000.000 3.14619 293060000.000 6.00 3.27 3.02 6.24 5.76

28313000.000 3.14623 293130000.000 6.00 3.27 3.02 6.24 5.76

28320000.000 3.14627 293200000.000 6.00 3.27 3.02 6.24 5.76

28327000.000 3.14631 293270000.000 6.00 3.27 3.02 6.24 5.76

28334000.000 3.14635 293340000.000 6.00 3.27 3.02 6.24 5.76

28341000.000 3.14639 293410000.000 6.00 3.27 3.02 6.24 5.76

28348000.000 3.14643 293480000.000 6.00 3.27 3.02 6.24 5.76

28355000.000 3.14647 293550000.000 6.00 3.27 3.02 6.24 5.76

28362000.000 3.14650 293620000.000 6.00 3.27 3.02 6.24 5.76

28369000.000 3.14654 293690000.000 6.00 3.27 3.02 6.24 5.76

28376000.000 3.14658 293760000.000 6.00 3.27 3.02 6.24 5.76

28383000.000 3.14662 293830000.000 6.00 3.27 3.02 6.24 5.76

28390000.000 3.14666 293900000.000 6.00 3.27 3.02 6.24 5.76

28397000.000 3.14670 293970000.000 6.00 3.27 3.02 6.24 5.76

28404000.000 3.14674 294040000.000 6.00 3.27 3.02 6.24 5.76

28411000.000 3.14678 294110000.000 6.00 3.27 3.02 6.24 5.76

28418000.000 3.14682 294180000.000 6.00 3.27 3.02 6.24 5.76

28425000.000 3.14685 294250000.000 6.00 3.27 3.02 6.24 5.76

28432000.000 3.14689 294320000.000 6.00 3.27 3.02 6.24 5.76

28439000.000 3.14693 294390000.000 6.00 3.27 3.02 6.24 5.76

28446000.000 3.14697 294460000.000 6.00 3.27 3.02 6.24 5.76

28453000.000 3.14701 294530000.000 6.00 3.27 3.02 6.24 5.76

28460000.000 3.14705 294600000.000 6.00 3.27 3.02 6.24 5.76

28467000.000 3.14709 294670000.000 6.00 3.27 3.02 6.24 5.76

28474000.000 3.14713 294740000.000 6.00 3.27 3.02 6.24 5.76

28481000.000 3.14717 294810000.000 6.00 3.27 3.02 6.24 5.76

28488000.000 3.14720 294880000.000 6.00 3.27 3.02 6.24 5.76

28495000.000 3.14724 294950000.000 6.00 3.27 3.02 6.24 5.76

28502000.000 3.14728 295020000.000 6.00 3.27 3.02 6.24 5.76

28509000.000 3.14732 295090000.000 6.00 3.27 3.02 6.24 5.76

28516000.000 3.14736 295160000.000 6.00 3.27 3.02 6.24 5.76

28523000.000 3.14740 295230000.000 6.00 3.27 3.02 6.24 5.76

28530000.000 3.14744 295300000.000 6.00 3.27 3.02 6.24 5.76

28537000.000 3.14748 295370000.000 6.00 3.27 3.02 6.24 5.76

28544000.000 3.14751 295440000.000 6.00 3.27 3.02 6.24 5.76

28551000.000 3.14755 295510000.000 6.00 3.27 3.02 6.24 5.76

28558000.000 3.14759 295580000.000 6.00 3.27 3.02 6.24 5.76

28565000.000 3.14763 295650000.000 6.00 3.27 3.02 6.24 5.76

28572000.000 3.14767 295720000.000 6.00 3.27 3.02 6.24 5.76

28579000.000 3.14771 295790000.000 6.00 3.27 3.02 6.24 5.76

28586000.000 3.14775 295860000.000 6.00 3.27 3.02 6.24 5.76

28593000.000 3.14778 295930000.000 6.00 3.27 3.02 6.24 5.76

28600000.000 3.14782 296000000.000 6.00 3.27 3.02 6.24 5.76

28607000.000 3.14786 296070000.000 6.00 3.27 3.02 6.24 5.76

28614000.000 3.14790 296140000.000 6.00 3.27 3.02 6.24 5.76

28621000.000 3.14794 296210000.000 6.00 3.27 3.02 6.24 5.76

28628000.000 3.14798 296280000.000 6.00 3.27 3.02 6.24 5.76

28635000.000 3.14802 296350000.000 6.00 3.27 3.02 6.24 5.76

28642000.000 3.14806 296420000.000 6.00 3.27 3.02 6.24 5.76

28649000.000 3.14809 296490000.000 6.00 3.27 3.02 6.24 5.76

28656000.000 3.14813 296560000.000 6.00 3.27 3.02 6.24 5.76

28663000.000 3.14817 296630000.000 6.00 3.27 3.02 6.24 5.76

28670000.000 3.14821 296700000.000 6.00 3.27 3.02 6.24 5.76

28677000.000 3.14825 296770000.000 6.00 3.27 3.02 6.24 5.76

28684000.000 3.14829 296840000.000 6.00 3.27 3.02 6.24 5.76

28691000.000 3.14833 296910000.000 6.00 3.27 3.02 6.24 5.76

28698000.000 3.14836 296980000.000 6.00 3.27 3.02 6.24 5.76

28705000.000 3.14840 297050000.000 6.00 3.27 3.02 6.24 5.76

28712000.000 3.14844 297120000.000 6.00 3.27 3.02 6.24 5.76

28719000.000 3.14848 297190000.000 6.00 3.27 3.02 6.24 5.76

28726000.000 3.14852 297260000.000 6.00 3.27 3.02 6.24 5.76

28733000.000 3.14856 297330000.000 6.00 3.27 3.02 6.24 5.76

28740000.000 3.14859 297400000.000 6.00 3.27 3.02 6.24 5.76

28747000.000 3.14863 297470000.000 6.00 3.27 3.02 6.24 5.76

28754000.000 3.14867 297540000.000 6.00 3.27 3.02 6.24 5.76

28761000.000 3.14871 297610000.000 6.00 3.27 3.02 6.24 5.76

28768000.000 3.14875 297680000.000 6.00 3.27 3.02 6.24 5.76

28775000.000 3.14879 297750000.000 6.00 3.27 3.02 6.24 5.76

28782000.000 3.14883 297820000.000 6.00 3.27 3.02 6.24 5.76

28789000.000 3.14886 297890000.000 6.00 3.27 3.02 6.24 5.76

28796000.000 3.14890 297960000.000 6.00 3.27 3.02 6.24 5.76

28803000.000 3.14894 298030000.000 6.00 3.27 3.02 6.24 5.76

28810000.000 3.14898 298100000.000 6.00 3.27 3.02 6.24 5.76

28817000.000 3.14902 298170000.000 6.00 3.27 3.02 6.24 5.76

28824000.000 3.14906 298240000.000 6.00 3.28 3.02 6.24 5.76

28831000.000 3.14909 298310000.000 6.00 3.28 3.02 6.24 5.76

28838000.000 3.14913 298380000.000 6.00 3.28 3.02 6.24 5.76

28845000.000 3.14917 298450000.000 6.00 3.28 3.02 6.24 5.76

28852000.000 3.14921 298520000.000 6.00 3.28 3.02 6.24 5.76

28859000.000 3.14925 298590000.000 6.00 3.28 3.02 6.24 5.76

28866000.000 3.14929 298660000.000 6.00 3.28 3.02 6.24 5.76

28873000.000 3.14932 298730000.000 6.00 3.28 3.02 6.24 5.76

28880000.000 3.14936 298800000.000 6.00 3.28 3.02 6.24 5.76

28887000.000 3.14940 298870000.000 6.00 3.28 3.02 6.24 5.76

28894000.000 3.14944 298940000.000 6.00 3.28 3.02 6.24 5.76

28901000.000 3.14948 299010000.000 6.00 3.28 3.02 6.24 5.76

28908000.000 3.14952 299080000.000 6.00 3.28 3.02 6.24 5.76

28915000.000 3.14955 299150000.000 6.00 3.28 3.02 6.24 5.76

28922000.000 3.14959 299220000.000 6.00 3.28 3.02 6.24 5.76

28929000.000 3.14963 299290000.000 6.00 3.28 3.02 6.24 5.76

28936000.000 3.14967 299360000.000 6.00 3.28 3.02 6.24 5.76

28943000.000 3.14971 299430000.000 6.00 3.28 3.02 6.24 5.76

28950000.000 3.14974 299500000.000 6.00 3.28 3.02 6.24 5.76

28957000.000 3.14978 299570000.000 6.00 3.28 3.02 6.24 5.76

28964000.000 3.14982 299640000.000 6.00 3.28 3.02 6.24 5.76

28971000.000 3.14986 299710000.000 6.00 3.28 3.02 6.24 5.76

28978000.000 3.14990 299780000.000 6.00 3.28 3.02 6.24 5.76

28985000.000 3.14994 299850000.000 6.00 3.28 3.02 6.24 5.76

28992000.000 3.14997 299920000.000 6.00 3.28 3.02 6.24 5.76

28999000.000 3.15001 299990000.000 6.00 3.28 3.02 6.24 5.76

29006000.000 3.15005 300060000.000 6.00 3.28 3.02 6.24 5.76

29013000.000 3.15009 300130000.000 6.00 3.28 3.02 6.24 5.76

29020000.000 3.15013 300200000.000 6.00 3.28 3.02 6.24 5.76

29027000.000 3.15016 300270000.000 6.00 3.28 3.02 6.24 5.76

29034000.000 3.15020 300340000.000 6.00 3.28 3.02 6.24 5.76

29041000.000 3.15024 300410000.000 6.00 3.28 3.02 6.24 5.76

29048000.000 3.15028 300480000.000 6.00 3.28 3.02 6.24 5.76

29055000.000 3.15032 300550000.000 6.00 3.28 3.02 6.25 5.76

29062000.000 3.15035 300620000.000 6.00 3.28 3.02 6.25 5.76

29069000.000 3.15039 300690000.000 6.00 3.28 3.02 6.25 5.76

29076000.000 3.15043 300760000.000 6.01 3.28 3.02 6.25 5.76

29083000.000 3.15047 300830000.000 6.01 3.28 3.02 6.25 5.76

29090000.000 3.15051 300900000.000 6.01 3.28 3.02 6.25 5.76

29097000.000 3.15054 300970000.000 6.01 3.28 3.02 6.25 5.77

29104000.000 3.15058 301040000.000 6.01 3.28 3.02 6.25 5.77

29111000.000 3.15062 301110000.000 6.01 3.28 3.02 6.25 5.77

29118000.000 3.15066 301180000.000 6.01 3.28 3.02 6.25 5.77

29125000.000 3.15070 301250000.000 6.01 3.28 3.02 6.25 5.77

29132000.000 3.15073 301320000.000 6.01 3.28 3.02 6.25 5.77

29139000.000 3.15077 301390000.000 6.01 3.28 3.02 6.25 5.77

29146000.000 3.15081 301460000.000 6.01 3.28 3.02 6.25 5.77

29153000.000 3.15085 301530000.000 6.01 3.28 3.02 6.25 5.77

29160000.000 3.15089 301600000.000 6.01 3.28 3.02 6.25 5.77

29167000.000 3.15092 301670000.000 6.01 3.28 3.02 6.25 5.77

29174000.000 3.15096 301740000.000 6.01 3.28 3.02 6.25 5.77

29181000.000 3.15100 301810000.000 6.01 3.28 3.02 6.25 5.77

29188000.000 3.15104 301880000.000 6.01 3.28 3.02 6.25 5.77

29195000.000 3.15108 301950000.000 6.01 3.28 3.03 6.25 5.77

29202000.000 3.15111 302020000.000 6.01 3.28 3.03 6.25 5.77

29209000.000 3.15115 302090000.000 6.01 3.28 3.03 6.25 5.77

29216000.000 3.15119 302160000.000 6.01 3.28 3.03 6.25 5.77

29223000.000 3.15123 302230000.000 6.01 3.28 3.03 6.25 5.77

29230000.000 3.15127 302300000.000 6.01 3.28 3.03 6.25 5.77

29237000.000 3.15130 302370000.000 6.01 3.28 3.03 6.25 5.77

29244000.000 3.15134 302440000.000 6.01 3.28 3.03 6.25 5.77

29251000.000 3.15138 302510000.000 6.01 3.28 3.03 6.25 5.77

29258000.000 3.15142 302580000.000 6.01 3.28 3.03 6.25 5.77

29265000.000 3.15145 302650000.000 6.01 3.28 3.03 6.25 5.77

29272000.000 3.15149 302720000.000 6.01 3.28 3.03 6.25 5.77

29279000.000 3.15153 302790000.000 6.01 3.28 3.03 6.25 5.77

29286000.000 3.15157 302860000.000 6.01 3.28 3.03 6.25 5.77

29293000.000 3.15161 302930000.000 6.01 3.28 3.03 6.25 5.77

29300000.000 3.15164 303000000.000 6.01 3.28 3.03 6.25 5.77

29307000.000 3.15168 303070000.000 6.01 3.28 3.03 6.25 5.77

29314000.000 3.15172 303140000.000 6.01 3.28 3.03 6.25 5.77

29321000.000 3.15176 303210000.000 6.01 3.28 3.03 6.25 5.77

29328000.000 3.15179 303280000.000 6.01 3.28 3.03 6.25 5.77

29335000.000 3.15183 303350000.000 6.01 3.28 3.03 6.25 5.77

29342000.000 3.15187 303420000.000 6.01 3.28 3.03 6.25 5.77

29349000.000 3.15191 303490000.000 6.01 3.28 3.03 6.25 5.77

29356000.000 3.15195 303560000.000 6.01 3.28 3.03 6.25 5.77

29363000.000 3.15198 303630000.000 6.01 3.28 3.03 6.25 5.77

29370000.000 3.15202 303700000.000 6.01 3.28 3.03 6.25 5.77

29377000.000 3.15206 303770000.000 6.01 3.28 3.03 6.25 5.77

29384000.000 3.15210 303840000.000 6.01 3.28 3.03 6.25 5.77

29391000.000 3.15213 303910000.000 6.01 3.28 3.03 6.25 5.77

29398000.000 3.15217 303980000.000 6.01 3.28 3.03 6.25 5.77

29405000.000 3.15221 304050000.000 6.01 3.28 3.03 6.25 5.77

29412000.000 3.15225 304120000.000 6.01 3.28 3.03 6.25 5.77

29419000.000 3.15228 304190000.000 6.01 3.28 3.03 6.25 5.77

29426000.000 3.15232 304260000.000 6.01 3.28 3.03 6.25 5.77

29433000.000 3.15236 304330000.000 6.01 3.28 3.03 6.25 5.77

29440000.000 3.15240 304400000.000 6.01 3.28 3.03 6.25 5.77

29447000.000 3.15244 304470000.000 6.01 3.28 3.03 6.25 5.77

29454000.000 3.15247 304540000.000 6.01 3.28 3.03 6.25 5.77

29461000.000 3.15251 304610000.000 6.01 3.28 3.03 6.25 5.77

29468000.000 3.15255 304680000.000 6.01 3.28 3.03 6.25 5.77

29475000.000 3.15259 304750000.000 6.01 3.28 3.03 6.25 5.77

29482000.000 3.15262 304820000.000 6.01 3.28 3.03 6.25 5.77

29489000.000 3.15266 304890000.000 6.01 3.28 3.03 6.25 5.77

29496000.000 3.15270 304960000.000 6.01 3.28 3.03 6.25 5.77

29503000.000 3.15274 305030000.000 6.01 3.28 3.03 6.25 5.77

29510000.000 3.15277 305100000.000 6.01 3.28 3.03 6.25 5.77

29517000.000 3.15281 305170000.000 6.01 3.28 3.03 6.25 5.77

29524000.000 3.15285 305240000.000 6.01 3.28 3.03 6.25 5.77

29531000.000 3.15289 305310000.000 6.01 3.28 3.03 6.25 5.77

29538000.000 3.15292 305380000.000 6.01 3.28 3.03 6.25 5.77

29545000.000 3.15296 305450000.000 6.01 3.28 3.03 6.25 5.77

29552000.000 3.15300 305520000.000 6.01 3.28 3.03 6.25 5.77

29559000.000 3.15304 305590000.000 6.01 3.28 3.03 6.25 5.77

29566000.000 3.15307 305660000.000 6.01 3.28 3.03 6.25 5.77

29573000.000 3.15311 305730000.000 6.01 3.28 3.03 6.25 5.77

29580000.000 3.15315 305800000.000 6.01 3.28 3.03 6.25 5.77

29587000.000 3.15319 305870000.000 6.01 3.28 3.03 6.25 5.77

29594000.000 3.15322 305940000.000 6.01 3.28 3.03 6.25 5.77

29601000.000 3.15326 306010000.000 6.01 3.28 3.03 6.25 5.77

29608000.000 3.15330 306080000.000 6.01 3.28 3.03 6.25 5.77

29615000.000 3.15333 306150000.000 6.01 3.28 3.03 6.25 5.77

29622000.000 3.15337 306220000.000 6.01 3.28 3.03 6.25 5.77

29629000.000 3.15341 306290000.000 6.01 3.28 3.03 6.25 5.77

29636000.000 3.15345 306360000.000 6.01 3.28 3.03 6.25 5.77

29643000.000 3.15348 306430000.000 6.01 3.28 3.03 6.25 5.77

29650000.000 3.15352 306500000.000 6.01 3.28 3.03 6.25 5.77

29657000.000 3.15356 306570000.000 6.01 3.28 3.03 6.25 5.77

29664000.000 3.15360 306640000.000 6.01 3.28 3.03 6.25 5.77

29671000.000 3.15363 306710000.000 6.01 3.28 3.03 6.25 5.77

29678000.000 3.15367 306780000.000 6.01 3.28 3.03 6.25 5.77

29685000.000 3.15371 306850000.000 6.01 3.28 3.03 6.25 5.77

29692000.000 3.15375 306920000.000 6.01 3.28 3.03 6.25 5.77

29699000.000 3.15378 306990000.000 6.01 3.28 3.03 6.25 5.77

29706000.000 3.15382 307060000.000 6.01 3.28 3.03 6.25 5.77

29713000.000 3.15386 307130000.000 6.01 3.28 3.03 6.25 5.77

29720000.000 3.15389 307200000.000 6.01 3.28 3.03 6.25 5.77

29727000.000 3.15393 307270000.000 6.01 3.28 3.03 6.25 5.77

29734000.000 3.15397 307340000.000 6.01 3.28 3.03 6.25 5.77

29741000.000 3.15401 307410000.000 6.01 3.28 3.03 6.25 5.77

29748000.000 3.15404 307480000.000 6.01 3.28 3.03 6.25 5.77

29755000.000 3.15408 307550000.000 6.01 3.28 3.03 6.25 5.77

29762000.000 3.15412 307620000.000 6.01 3.28 3.03 6.25 5.77

29769000.000 3.15416 307690000.000 6.01 3.28 3.03 6.25 5.77

29776000.000 3.15419 307760000.000 6.01 3.28 3.03 6.25 5.77

29783000.000 3.15423 307830000.000 6.01 3.28 3.03 6.25 5.77

29790000.000 3.15427 307900000.000 6.01 3.28 3.03 6.25 5.77

29797000.000 3.15430 307970000.000 6.01 3.28 3.03 6.25 5.77

29804000.000 3.15434 308040000.000 6.01 3.28 3.03 6.25 5.77

29811000.000 3.15438 308110000.000 6.01 3.28 3.03 6.25 5.77

29818000.000 3.15442 308180000.000 6.01 3.28 3.03 6.25 5.77

29825000.000 3.15445 308250000.000 6.01 3.28 3.03 6.25 5.77

29832000.000 3.15449 308320000.000 6.01 3.28 3.03 6.25 5.77

29839000.000 3.15453 308390000.000 6.01 3.28 3.03 6.25 5.77

29846000.000 3.15456 308460000.000 6.01 3.28 3.03 6.25 5.77

29853000.000 3.15460 308530000.000 6.01 3.28 3.03 6.25 5.77

29860000.000 3.15464 308600000.000 6.01 3.28 3.03 6.25 5.77

29867000.000 3.15468 308670000.000 6.01 3.28 3.03 6.25 5.77

29874000.000 3.15471 308740000.000 6.01 3.28 3.03 6.25 5.77

29881000.000 3.15475 308810000.000 6.01 3.28 3.03 6.25 5.77

29888000.000 3.15479 308880000.000 6.01 3.28 3.03 6.25 5.77

29895000.000 3.15482 308950000.000 6.01 3.28 3.03 6.25 5.77

29902000.000 3.15486 309020000.000 6.01 3.28 3.03 6.25 5.77

29909000.000 3.15490 309090000.000 6.01 3.28 3.03 6.25 5.77

29916000.000 3.15493 309160000.000 6.01 3.28 3.03 6.25 5.77

29923000.000 3.15497 309230000.000 6.01 3.28 3.03 6.25 5.77

29930000.000 3.15501 309300000.000 6.01 3.28 3.03 6.25 5.77

29937000.000 3.15505 309370000.000 6.01 3.28 3.03 6.25 5.77

29944000.000 3.15508 309440000.000 6.01 3.28 3.03 6.25 5.77

29951000.000 3.15512 309510000.000 6.01 3.28 3.03 6.25 5.77

29958000.000 3.15516 309580000.000 6.01 3.28 3.03 6.25 5.77

29965000.000 3.15519 309650000.000 6.01 3.28 3.03 6.25 5.77

29972000.000 3.15523 309720000.000 6.01 3.28 3.03 6.25 5.77

29979000.000 3.15527 309790000.000 6.01 3.28 3.03 6.25 5.77

29986000.000 3.15530 309860000.000 6.01 3.28 3.03 6.25 5.77

29993000.000 3.15534 309930000.000 6.01 3.28 3.03 6.25 5.77

30000000.000 3.15538 310000000.000 6.01 3.28 3.03 6.25 5.77

30007000.000 3.15542 310070000.000 6.01 3.28 3.03 6.25 5.77

30014000.000 3.15545 310140000.000 6.01 3.28 3.03 6.25 5.77

30021000.000 3.15549 310210000.000 6.01 3.28 3.03 6.25 5.77

30028000.000 3.15553 310280000.000 6.01 3.28 3.03 6.26 5.77

30035000.000 3.15556 310350000.000 6.01 3.28 3.03 6.26 5.77

30042000.000 3.15560 310420000.000 6.01 3.28 3.03 6.26 5.77

30049000.000 3.15564 310490000.000 6.01 3.28 3.03 6.26 5.77

30056000.000 3.15567 310560000.000 6.01 3.28 3.03 6.26 5.77

30063000.000 3.15571 310630000.000 6.01 3.28 3.03 6.26 5.77

30070000.000 3.15575 310700000.000 6.01 3.28 3.03 6.26 5.77

30077000.000 3.15578 310770000.000 6.01 3.28 3.03 6.26 5.77

30084000.000 3.15582 310840000.000 6.01 3.28 3.03 6.26 5.77

30091000.000 3.15586 310910000.000 6.02 3.28 3.03 6.26 5.77

30098000.000 3.15589 310980000.000 6.02 3.28 3.03 6.26 5.77

30105000.000 3.15593 311050000.000 6.02 3.28 3.03 6.26 5.77

30112000.000 3.15597 311120000.000 6.02 3.28 3.03 6.26 5.77

30119000.000 3.15601 311190000.000 6.02 3.28 3.03 6.26 5.77

30126000.000 3.15604 311260000.000 6.02 3.28 3.03 6.26 5.77

30133000.000 3.15608 311330000.000 6.02 3.28 3.03 6.26 5.77

30140000.000 3.15612 311400000.000 6.02 3.28 3.03 6.26 5.77

30147000.000 3.15615 311470000.000 6.02 3.28 3.03 6.26 5.77

30154000.000 3.15619 311540000.000 6.02 3.28 3.03 6.26 5.78

30161000.000 3.15623 311610000.000 6.02 3.28 3.03 6.26 5.78

30168000.000 3.15626 311680000.000 6.02 3.28 3.03 6.26 5.78

30175000.000 3.15630 311750000.000 6.02 3.28 3.03 6.26 5.78

30182000.000 3.15634 311820000.000 6.02 3.28 3.03 6.26 5.78

30189000.000 3.15637 311890000.000 6.02 3.28 3.03 6.26 5.78

30196000.000 3.15641 311960000.000 6.02 3.28 3.03 6.26 5.78

30203000.000 3.15645 312030000.000 6.02 3.28 3.03 6.26 5.78

30210000.000 3.15648 312100000.000 6.02 3.28 3.03 6.26 5.78

30217000.000 3.15652 312170000.000 6.02 3.28 3.03 6.26 5.78

30224000.000 3.15656 312240000.000 6.02 3.28 3.03 6.26 5.78

30231000.000 3.15659 312310000.000 6.02 3.28 3.03 6.26 5.78

30238000.000 3.15663 312380000.000 6.02 3.28 3.03 6.26 5.78

30245000.000 3.15667 312450000.000 6.02 3.28 3.03 6.26 5.78

30252000.000 3.15670 312520000.000 6.02 3.28 3.03 6.26 5.78

30259000.000 3.15674 312590000.000 6.02 3.28 3.03 6.26 5.78

30266000.000 3.15678 312660000.000 6.02 3.28 3.03 6.26 5.78

30273000.000 3.15681 312730000.000 6.02 3.28 3.03 6.26 5.78

30280000.000 3.15685 312800000.000 6.02 3.28 3.03 6.26 5.78

30287000.000 3.15689 312870000.000 6.02 3.28 3.03 6.26 5.78

30294000.000 3.15692 312940000.000 6.02 3.28 3.03 6.26 5.78

30301000.000 3.15696 313010000.000 6.02 3.28 3.03 6.26 5.78

30308000.000 3.15700 313080000.000 6.02 3.28 3.03 6.26 5.78

30315000.000 3.15703 313150000.000 6.02 3.28 3.03 6.26 5.78

30322000.000 3.15707 313220000.000 6.02 3.28 3.03 6.26 5.78

30329000.000 3.15711 313290000.000 6.02 3.28 3.03 6.26 5.78

30336000.000 3.15714 313360000.000 6.02 3.28 3.03 6.26 5.78

30343000.000 3.15718 313430000.000 6.02 3.28 3.03 6.26 5.78

30350000.000 3.15721 313500000.000 6.02 3.28 3.03 6.26 5.78

30357000.000 3.15725 313570000.000 6.02 3.28 3.03 6.26 5.78

30364000.000 3.15729 313640000.000 6.02 3.28 3.03 6.26 5.78

30371000.000 3.15732 313710000.000 6.02 3.28 3.03 6.26 5.78

30378000.000 3.15736 313780000.000 6.02 3.28 3.03 6.26 5.78

30385000.000 3.15740 313850000.000 6.02 3.28 3.03 6.26 5.78

30392000.000 3.15743 313920000.000 6.02 3.28 3.03 6.26 5.78

30399000.000 3.15747 313990000.000 6.02 3.28 3.03 6.26 5.78

30406000.000 3.15751 314060000.000 6.02 3.28 3.03 6.26 5.78

30413000.000 3.15754 314130000.000 6.02 3.28 3.03 6.26 5.78

30420000.000 3.15758 314200000.000 6.02 3.28 3.03 6.26 5.78

30427000.000 3.15762 314270000.000 6.02 3.28 3.03 6.26 5.78

30434000.000 3.15765 314340000.000 6.02 3.28 3.03 6.26 5.78

30441000.000 3.15769 314410000.000 6.02 3.28 3.03 6.26 5.78

30448000.000 3.15773 314480000.000 6.02 3.28 3.03 6.26 5.78

30455000.000 3.15776 314550000.000 6.02 3.28 3.03 6.26 5.78

30462000.000 3.15780 314620000.000 6.02 3.28 3.03 6.26 5.78

30469000.000 3.15783 314690000.000 6.02 3.28 3.03 6.26 5.78

30476000.000 3.15787 314760000.000 6.02 3.28 3.03 6.26 5.78

30483000.000 3.15791 314830000.000 6.02 3.28 3.03 6.26 5.78

30490000.000 3.15794 314900000.000 6.02 3.28 3.03 6.26 5.78

30497000.000 3.15798 314970000.000 6.02 3.28 3.03 6.26 5.78

30504000.000 3.15802 315040000.000 6.02 3.28 3.03 6.26 5.78

30511000.000 3.15805 315110000.000 6.02 3.28 3.03 6.26 5.78

30518000.000 3.15809 315180000.000 6.02 3.28 3.03 6.26 5.78

30525000.000 3.15813 315250000.000 6.02 3.28 3.03 6.26 5.78

30532000.000 3.15816 315320000.000 6.02 3.28 3.03 6.26 5.78

30539000.000 3.15820 315390000.000 6.02 3.28 3.03 6.26 5.78

30546000.000 3.15823 315460000.000 6.02 3.28 3.03 6.26 5.78

30553000.000 3.15827 315530000.000 6.02 3.28 3.03 6.26 5.78

30560000.000 3.15831 315600000.000 6.02 3.28 3.03 6.26 5.78

30567000.000 3.15834 315670000.000 6.02 3.28 3.03 6.26 5.78

30574000.000 3.15838 315740000.000 6.02 3.28 3.03 6.26 5.78

30581000.000 3.15842 315810000.000 6.02 3.28 3.03 6.26 5.78

30588000.000 3.15845 315880000.000 6.02 3.28 3.03 6.26 5.78

30595000.000 3.15849 315950000.000 6.02 3.28 3.03 6.26 5.78

30602000.000 3.15852 316020000.000 6.02 3.28 3.03 6.26 5.78

30609000.000 3.15856 316090000.000 6.02 3.28 3.03 6.26 5.78

30616000.000 3.15860 316160000.000 6.02 3.28 3.03 6.26 5.78

30623000.000 3.15863 316230000.000 6.02 3.28 3.03 6.26 5.78

30630000.000 3.15867 316300000.000 6.02 3.29 3.03 6.26 5.78

30637000.000 3.15871 316370000.000 6.02 3.29 3.03 6.26 5.78

30644000.000 3.15874 316440000.000 6.02 3.29 3.03 6.26 5.78

30651000.000 3.15878 316510000.000 6.02 3.29 3.03 6.26 5.78

30658000.000 3.15881 316580000.000 6.02 3.29 3.03 6.26 5.78

30665000.000 3.15885 316650000.000 6.02 3.29 3.03 6.26 5.78

30672000.000 3.15889 316720000.000 6.02 3.29 3.03 6.26 5.78

30679000.000 3.15892 316790000.000 6.02 3.29 3.03 6.26 5.78

30686000.000 3.15896 316860000.000 6.02 3.29 3.03 6.26 5.78

30693000.000 3.15900 316930000.000 6.02 3.29 3.03 6.26 5.78

30700000.000 3.15903 317000000.000 6.02 3.29 3.03 6.26 5.78

30707000.000 3.15907 317070000.000 6.02 3.29 3.03 6.26 5.78

30714000.000 3.15910 317140000.000 6.02 3.29 3.03 6.26 5.78

30721000.000 3.15914 317210000.000 6.02 3.29 3.03 6.26 5.78

30728000.000 3.15918 317280000.000 6.02 3.29 3.03 6.26 5.78

30735000.000 3.15921 317350000.000 6.02 3.29 3.03 6.26 5.78

30742000.000 3.15925 317420000.000 6.02 3.29 3.03 6.26 5.78

30749000.000 3.15928 317490000.000 6.02 3.29 3.03 6.26 5.78

30756000.000 3.15932 317560000.000 6.02 3.29 3.03 6.26 5.78

30763000.000 3.15936 317630000.000 6.02 3.29 3.03 6.26 5.78

30770000.000 3.15939 317700000.000 6.02 3.29 3.03 6.26 5.78

30777000.000 3.15943 317770000.000 6.02 3.29 3.03 6.26 5.78

30784000.000 3.15946 317840000.000 6.02 3.29 3.03 6.26 5.78

30791000.000 3.15950 317910000.000 6.02 3.29 3.03 6.26 5.78

30798000.000 3.15954 317980000.000 6.02 3.29 3.03 6.26 5.78

30805000.000 3.15957 318050000.000 6.02 3.29 3.03 6.26 5.78

30812000.000 3.15961 318120000.000 6.02 3.29 3.03 6.26 5.78

30819000.000 3.15964 318190000.000 6.02 3.29 3.03 6.26 5.78

30826000.000 3.15968 318260000.000 6.02 3.29 3.03 6.26 5.78

30833000.000 3.15972 318330000.000 6.02 3.29 3.03 6.26 5.78

30840000.000 3.15975 318400000.000 6.02 3.29 3.03 6.26 5.78

30847000.000 3.15979 318470000.000 6.02 3.29 3.03 6.26 5.78

30854000.000 3.15982 318540000.000 6.02 3.29 3.03 6.26 5.78

30861000.000 3.15986 318610000.000 6.02 3.29 3.03 6.26 5.78

30868000.000 3.15990 318680000.000 6.02 3.29 3.03 6.26 5.78

30875000.000 3.15993 318750000.000 6.02 3.29 3.03 6.26 5.78

30882000.000 3.15997 318820000.000 6.02 3.29 3.03 6.26 5.78

30889000.000 3.16000 318890000.000 6.02 3.29 3.03 6.26 5.78

30896000.000 3.16004 318960000.000 6.02 3.29 3.03 6.26 5.78

30903000.000 3.16008 319030000.000 6.02 3.29 3.03 6.26 5.78

30910000.000 3.16011 319100000.000 6.02 3.29 3.03 6.26 5.78

30917000.000 3.16015 319170000.000 6.02 3.29 3.03 6.26 5.78

30924000.000 3.16018 319240000.000 6.02 3.29 3.03 6.26 5.78

30931000.000 3.16022 319310000.000 6.02 3.29 3.03 6.26 5.78

30938000.000 3.16026 319380000.000 6.02 3.29 3.03 6.26 5.78

30945000.000 3.16029 319450000.000 6.02 3.29 3.03 6.26 5.78

30952000.000 3.16033 319520000.000 6.02 3.29 3.03 6.26 5.78

30959000.000 3.16036 319590000.000 6.02 3.29 3.03 6.26 5.78

30966000.000 3.16040 319660000.000 6.02 3.29 3.03 6.26 5.78

30973000.000 3.16043 319730000.000 6.02 3.29 3.03 6.26 5.78

30980000.000 3.16047 319800000.000 6.02 3.29 3.03 6.26 5.78

30987000.000 3.16051 319870000.000 6.02 3.29 3.03 6.26 5.78

30994000.000 3.16054 319940000.000 6.02 3.29 3.03 6.26 5.78

31001000.000 3.16058 320010000.000 6.02 3.29 3.03 6.26 5.78

31008000.000 3.16061 320080000.000 6.02 3.29 3.03 6.26 5.78

31015000.000 3.16065 320150000.000 6.02 3.29 3.03 6.26 5.78

31022000.000 3.16068 320220000.000 6.02 3.29 3.03 6.26 5.78

31029000.000 3.16072 320290000.000 6.02 3.29 3.03 6.26 5.78

31036000.000 3.16076 320360000.000 6.02 3.29 3.03 6.27 5.78

31043000.000 3.16079 320430000.000 6.02 3.29 3.03 6.27 5.78

31050000.000 3.16083 320500000.000 6.02 3.29 3.03 6.27 5.78

31057000.000 3.16086 320570000.000 6.02 3.29 3.03 6.27 5.78

31064000.000 3.16090 320640000.000 6.02 3.29 3.03 6.27 5.78

31071000.000 3.16094 320710000.000 6.02 3.29 3.03 6.27 5.78

31078000.000 3.16097 320780000.000 6.02 3.29 3.03 6.27 5.78

31085000.000 3.16101 320850000.000 6.02 3.29 3.03 6.27 5.78

31092000.000 3.16104 320920000.000 6.02 3.29 3.03 6.27 5.78

31099000.000 3.16108 320990000.000 6.02 3.29 3.03 6.27 5.78

31106000.000 3.16111 321060000.000 6.02 3.29 3.03 6.27 5.78

31113000.000 3.16115 321130000.000 6.02 3.29 3.03 6.27 5.78

31120000.000 3.16119 321200000.000 6.02 3.29 3.03 6.27 5.78

31127000.000 3.16122 321270000.000 6.02 3.29 3.03 6.27 5.78

31134000.000 3.16126 321340000.000 6.03 3.29 3.03 6.27 5.78

31141000.000 3.16129 321410000.000 6.03 3.29 3.03 6.27 5.78

31148000.000 3.16133 321480000.000 6.03 3.29 3.03 6.27 5.78

31155000.000 3.16136 321550000.000 6.03 3.29 3.03 6.27 5.78

31162000.000 3.16140 321620000.000 6.03 3.29 3.03 6.27 5.78

31169000.000 3.16143 321690000.000 6.03 3.29 3.03 6.27 5.78

31176000.000 3.16147 321760000.000 6.03 3.29 3.04 6.27 5.78

31183000.000 3.16151 321830000.000 6.03 3.29 3.04 6.27 5.78

31190000.000 3.16154 321900000.000 6.03 3.29 3.04 6.27 5.78

31197000.000 3.16158 321970000.000 6.03 3.29 3.04 6.27 5.78

31204000.000 3.16161 322040000.000 6.03 3.29 3.04 6.27 5.78

31211000.000 3.16165 322110000.000 6.03 3.29 3.04 6.27 5.78

31218000.000 3.16168 322180000.000 6.03 3.29 3.04 6.27 5.78

31225000.000 3.16172 322250000.000 6.03 3.29 3.04 6.27 5.78

31232000.000 3.16175 322320000.000 6.03 3.29 3.04 6.27 5.78

31239000.000 3.16179 322390000.000 6.03 3.29 3.04 6.27 5.78

31246000.000 3.16183 322460000.000 6.03 3.29 3.04 6.27 5.79

31253000.000 3.16186 322530000.000 6.03 3.29 3.04 6.27 5.79

31260000.000 3.16190 322600000.000 6.03 3.29 3.04 6.27 5.79

31267000.000 3.16193 322670000.000 6.03 3.29 3.04 6.27 5.79

31274000.000 3.16197 322740000.000 6.03 3.29 3.04 6.27 5.79

31281000.000 3.16200 322810000.000 6.03 3.29 3.04 6.27 5.79

31288000.000 3.16204 322880000.000 6.03 3.29 3.04 6.27 5.79

31295000.000 3.16207 322950000.000 6.03 3.29 3.04 6.27 5.79

31302000.000 3.16211 323020000.000 6.03 3.29 3.04 6.27 5.79

31309000.000 3.16215 323090000.000 6.03 3.29 3.04 6.27 5.79

31316000.000 3.16218 323160000.000 6.03 3.29 3.04 6.27 5.79

31323000.000 3.16222 323230000.000 6.03 3.29 3.04 6.27 5.79

31330000.000 3.16225 323300000.000 6.03 3.29 3.04 6.27 5.79

31337000.000 3.16229 323370000.000 6.03 3.29 3.04 6.27 5.79

31344000.000 3.16232 323440000.000 6.03 3.29 3.04 6.27 5.79

31351000.000 3.16236 323510000.000 6.03 3.29 3.04 6.27 5.79

31358000.000 3.16239 323580000.000 6.03 3.29 3.04 6.27 5.79

31365000.000 3.16243 323650000.000 6.03 3.29 3.04 6.27 5.79

31372000.000 3.16246 323720000.000 6.03 3.29 3.04 6.27 5.79

31379000.000 3.16250 323790000.000 6.03 3.29 3.04 6.27 5.79

31386000.000 3.16253 323860000.000 6.03 3.29 3.04 6.27 5.79

31393000.000 3.16257 323930000.000 6.03 3.29 3.04 6.27 5.79

31400000.000 3.16261 324000000.000 6.03 3.29 3.04 6.27 5.79

31407000.000 3.16264 324070000.000 6.03 3.29 3.04 6.27 5.79

31414000.000 3.16268 324140000.000 6.03 3.29 3.04 6.27 5.79

31421000.000 3.16271 324210000.000 6.03 3.29 3.04 6.27 5.79

31428000.000 3.16275 324280000.000 6.03 3.29 3.04 6.27 5.79

31435000.000 3.16278 324350000.000 6.03 3.29 3.04 6.27 5.79

31442000.000 3.16282 324420000.000 6.03 3.29 3.04 6.27 5.79

31449000.000 3.16285 324490000.000 6.03 3.29 3.04 6.27 5.79

31456000.000 3.16289 324560000.000 6.03 3.29 3.04 6.27 5.79

31463000.000 3.16292 324630000.000 6.03 3.29 3.04 6.27 5.79

31470000.000 3.16296 324700000.000 6.03 3.29 3.04 6.27 5.79

31477000.000 3.16299 324770000.000 6.03 3.29 3.04 6.27 5.79

31484000.000 3.16303 324840000.000 6.03 3.29 3.04 6.27 5.79

31491000.000 3.16306 324910000.000 6.03 3.29 3.04 6.27 5.79

31498000.000 3.16310 324980000.000 6.03 3.29 3.04 6.27 5.79

31505000.000 3.16314 325050000.000 6.03 3.29 3.04 6.27 5.79

31512000.000 3.16317 325120000.000 6.03 3.29 3.04 6.27 5.79

31519000.000 3.16321 325190000.000 6.03 3.29 3.04 6.27 5.79

31526000.000 3.16324 325260000.000 6.03 3.29 3.04 6.27 5.79

31533000.000 3.16328 325330000.000 6.03 3.29 3.04 6.27 5.79

31540000.000 3.16331 325400000.000 6.03 3.29 3.04 6.27 5.79

31547000.000 3.16335 325470000.000 6.03 3.29 3.04 6.27 5.79

31554000.000 3.16338 325540000.000 6.03 3.29 3.04 6.27 5.79

31561000.000 3.16342 325610000.000 6.03 3.29 3.04 6.27 5.79

31568000.000 3.16345 325680000.000 6.03 3.29 3.04 6.27 5.79

31575000.000 3.16349 325750000.000 6.03 3.29 3.04 6.27 5.79

31582000.000 3.16352 325820000.000 6.03 3.29 3.04 6.27 5.79

31589000.000 3.16356 325890000.000 6.03 3.29 3.04 6.27 5.79

31596000.000 3.16359 325960000.000 6.03 3.29 3.04 6.27 5.79

31603000.000 3.16363 326030000.000 6.03 3.29 3.04 6.27 5.79

31610000.000 3.16366 326100000.000 6.03 3.29 3.04 6.27 5.79

31617000.000 3.16370 326170000.000 6.03 3.29 3.04 6.27 5.79

31624000.000 3.16373 326240000.000 6.03 3.29 3.04 6.27 5.79

31631000.000 3.16377 326310000.000 6.03 3.29 3.04 6.27 5.79

31638000.000 3.16380 326380000.000 6.03 3.29 3.04 6.27 5.79

31645000.000 3.16384 326450000.000 6.03 3.29 3.04 6.27 5.79

31652000.000 3.16387 326520000.000 6.03 3.29 3.04 6.27 5.79

31659000.000 3.16391 326590000.000 6.03 3.29 3.04 6.27 5.79

31666000.000 3.16394 326660000.000 6.03 3.29 3.04 6.27 5.79

31673000.000 3.16398 326730000.000 6.03 3.29 3.04 6.27 5.79

31680000.000 3.16401 326800000.000 6.03 3.29 3.04 6.27 5.79

31687000.000 3.16405 326870000.000 6.03 3.29 3.04 6.27 5.79

31694000.000 3.16408 326940000.000 6.03 3.29 3.04 6.27 5.79

31701000.000 3.16412 327010000.000 6.03 3.29 3.04 6.27 5.79

31708000.000 3.16415 327080000.000 6.03 3.29 3.04 6.27 5.79

31715000.000 3.16419 327150000.000 6.03 3.29 3.04 6.27 5.79

31722000.000 3.16422 327220000.000 6.03 3.29 3.04 6.27 5.79

31729000.000 3.16426 327290000.000 6.03 3.29 3.04 6.27 5.79

31736000.000 3.16429 327360000.000 6.03 3.29 3.04 6.27 5.79

31743000.000 3.16433 327430000.000 6.03 3.29 3.04 6.27 5.79

31750000.000 3.16436 327500000.000 6.03 3.29 3.04 6.27 5.79

31757000.000 3.16440 327570000.000 6.03 3.29 3.04 6.27 5.79

31764000.000 3.16443 327640000.000 6.03 3.29 3.04 6.27 5.79

31771000.000 3.16447 327710000.000 6.03 3.29 3.04 6.27 5.79

31778000.000 3.16450 327780000.000 6.03 3.29 3.04 6.27 5.79

31785000.000 3.16454 327850000.000 6.03 3.29 3.04 6.27 5.79

31792000.000 3.16457 327920000.000 6.03 3.29 3.04 6.27 5.79

31799000.000 3.16461 327990000.000 6.03 3.29 3.04 6.27 5.79

31806000.000 3.16464 328060000.000 6.03 3.29 3.04 6.27 5.79

31813000.000 3.16468 328130000.000 6.03 3.29 3.04 6.27 5.79

31820000.000 3.16471 328200000.000 6.03 3.29 3.04 6.27 5.79

31827000.000 3.16475 328270000.000 6.03 3.29 3.04 6.27 5.79

31834000.000 3.16478 328340000.000 6.03 3.29 3.04 6.27 5.79

31841000.000 3.16482 328410000.000 6.03 3.29 3.04 6.27 5.79

31848000.000 3.16485 328480000.000 6.03 3.29 3.04 6.27 5.79

31855000.000 3.16489 328550000.000 6.03 3.29 3.04 6.27 5.79

31862000.000 3.16492 328620000.000 6.03 3.29 3.04 6.27 5.79

31869000.000 3.16496 328690000.000 6.03 3.29 3.04 6.27 5.79

31876000.000 3.16499 328760000.000 6.03 3.29 3.04 6.27 5.79

31883000.000 3.16503 328830000.000 6.03 3.29 3.04 6.27 5.79

31890000.000 3.16506 328900000.000 6.03 3.29 3.04 6.27 5.79

31897000.000 3.16510 328970000.000 6.03 3.29 3.04 6.27 5.79

31904000.000 3.16513 329040000.000 6.03 3.29 3.04 6.27 5.79

31911000.000 3.16517 329110000.000 6.03 3.29 3.04 6.27 5.79

31918000.000 3.16520 329180000.000 6.03 3.29 3.04 6.27 5.79

31925000.000 3.16524 329250000.000 6.03 3.29 3.04 6.27 5.79

31932000.000 3.16527 329320000.000 6.03 3.29 3.04 6.27 5.79

31939000.000 3.16531 329390000.000 6.03 3.29 3.04 6.27 5.79

31946000.000 3.16534 329460000.000 6.03 3.29 3.04 6.27 5.79

31953000.000 3.16538 329530000.000 6.03 3.29 3.04 6.27 5.79

31960000.000 3.16541 329600000.000 6.03 3.29 3.04 6.27 5.79

31967000.000 3.16545 329670000.000 6.03 3.29 3.04 6.27 5.79

31974000.000 3.16548 329740000.000 6.03 3.29 3.04 6.27 5.79

31981000.000 3.16552 329810000.000 6.03 3.29 3.04 6.27 5.79

31988000.000 3.16555 329880000.000 6.03 3.29 3.04 6.27 5.79

31995000.000 3.16558 329950000.000 6.03 3.29 3.04 6.27 5.79

32002000.000 3.16562 330020000.000 6.03 3.29 3.04 6.27 5.79

32009000.000 3.16565 330090000.000 6.03 3.29 3.04 6.27 5.79

32016000.000 3.16569 330160000.000 6.03 3.29 3.04 6.27 5.79

32023000.000 3.16572 330230000.000 6.03 3.29 3.04 6.27 5.79

32030000.000 3.16576 330300000.000 6.03 3.29 3.04 6.27 5.79

32037000.000 3.16579 330370000.000 6.03 3.29 3.04 6.27 5.79

32044000.000 3.16583 330440000.000 6.03 3.29 3.04 6.27 5.79

32051000.000 3.16586 330510000.000 6.03 3.29 3.04 6.27 5.79

32058000.000 3.16590 330580000.000 6.03 3.29 3.04 6.27 5.79

32065000.000 3.16593 330650000.000 6.03 3.29 3.04 6.27 5.79

32072000.000 3.16597 330720000.000 6.03 3.29 3.04 6.28 5.79

32079000.000 3.16600 330790000.000 6.03 3.29 3.04 6.28 5.79

32086000.000 3.16604 330860000.000 6.03 3.29 3.04 6.28 5.79

32093000.000 3.16607 330930000.000 6.03 3.29 3.04 6.28 5.79

32100000.000 3.16611 331000000.000 6.03 3.29 3.04 6.28 5.79

32107000.000 3.16614 331070000.000 6.03 3.29 3.04 6.28 5.79

32114000.000 3.16617 331140000.000 6.03 3.29 3.04 6.28 5.79

32121000.000 3.16621 331210000.000 6.03 3.29 3.04 6.28 5.79

32128000.000 3.16624 331280000.000 6.03 3.29 3.04 6.28 5.79

32135000.000 3.16628 331350000.000 6.03 3.29 3.04 6.28 5.79

32142000.000 3.16631 331420000.000 6.03 3.29 3.04 6.28 5.79

32149000.000 3.16635 331490000.000 6.03 3.29 3.04 6.28 5.79

32156000.000 3.16638 331560000.000 6.03 3.29 3.04 6.28 5.79

32163000.000 3.16642 331630000.000 6.03 3.29 3.04 6.28 5.79

32170000.000 3.16645 331700000.000 6.03 3.29 3.04 6.28 5.79

32177000.000 3.16649 331770000.000 6.03 3.29 3.04 6.28 5.79

32184000.000 3.16652 331840000.000 6.03 3.29 3.04 6.28 5.79

32191000.000 3.16655 331910000.000 6.03 3.29 3.04 6.28 5.79

32198000.000 3.16659 331980000.000 6.03 3.29 3.04 6.28 5.79

32205000.000 3.16662 332050000.000 6.03 3.29 3.04 6.28 5.79

32212000.000 3.16666 332120000.000 6.03 3.29 3.04 6.28 5.79

32219000.000 3.16669 332190000.000 6.04 3.29 3.04 6.28 5.79

32226000.000 3.16673 332260000.000 6.04 3.29 3.04 6.28 5.79

32233000.000 3.16676 332330000.000 6.04 3.29 3.04 6.28 5.79

32240000.000 3.16680 332400000.000 6.04 3.29 3.04 6.28 5.79

32247000.000 3.16683 332470000.000 6.04 3.29 3.04 6.28 5.79

32254000.000 3.16687 332540000.000 6.04 3.29 3.04 6.28 5.79

32261000.000 3.16690 332610000.000 6.04 3.29 3.04 6.28 5.79

32268000.000 3.16693 332680000.000 6.04 3.29 3.04 6.28 5.79

32275000.000 3.16697 332750000.000 6.04 3.29 3.04 6.28 5.79

32282000.000 3.16700 332820000.000 6.04 3.29 3.04 6.28 5.79

32289000.000 3.16704 332890000.000 6.04 3.29 3.04 6.28 5.79

32296000.000 3.16707 332960000.000 6.04 3.29 3.04 6.28 5.79

32303000.000 3.16711 333030000.000 6.04 3.29 3.04 6.28 5.79

32310000.000 3.16714 333100000.000 6.04 3.29 3.04 6.28 5.79

32317000.000 3.16718 333170000.000 6.04 3.29 3.04 6.28 5.79

32324000.000 3.16721 333240000.000 6.04 3.29 3.04 6.28 5.79

32331000.000 3.16724 333310000.000 6.04 3.29 3.04 6.28 5.79

32338000.000 3.16728 333380000.000 6.04 3.29 3.04 6.28 5.79

32345000.000 3.16731 333450000.000 6.04 3.29 3.04 6.28 5.79

32352000.000 3.16735 333520000.000 6.04 3.29 3.04 6.28 5.79

32359000.000 3.16738 333590000.000 6.04 3.29 3.04 6.28 5.79

32366000.000 3.16742 333660000.000 6.04 3.29 3.04 6.28 5.79

32373000.000 3.16745 333730000.000 6.04 3.29 3.04 6.28 5.79

32380000.000 3.16748 333800000.000 6.04 3.29 3.04 6.28 5.80

32387000.000 3.16752 333870000.000 6.04 3.29 3.04 6.28 5.80

32394000.000 3.16755 333940000.000 6.04 3.29 3.04 6.28 5.80

32401000.000 3.16759 334010000.000 6.04 3.29 3.04 6.28 5.80

32408000.000 3.16762 334080000.000 6.04 3.29 3.04 6.28 5.80

32415000.000 3.16766 334150000.000 6.04 3.29 3.04 6.28 5.80

32422000.000 3.16769 334220000.000 6.04 3.29 3.04 6.28 5.80

32429000.000 3.16773 334290000.000 6.04 3.29 3.04 6.28 5.80

32436000.000 3.16776 334360000.000 6.04 3.29 3.04 6.28 5.80

32443000.000 3.16779 334430000.000 6.04 3.29 3.04 6.28 5.80

32450000.000 3.16783 334500000.000 6.04 3.29 3.04 6.28 5.80

32457000.000 3.16786 334570000.000 6.04 3.29 3.04 6.28 5.80

32464000.000 3.16790 334640000.000 6.04 3.29 3.04 6.28 5.80

32471000.000 3.16793 334710000.000 6.04 3.29 3.04 6.28 5.80

32478000.000 3.16796 334780000.000 6.04 3.29 3.04 6.28 5.80

32485000.000 3.16800 334850000.000 6.04 3.29 3.04 6.28 5.80

32492000.000 3.16803 334920000.000 6.04 3.29 3.04 6.28 5.80

32499000.000 3.16807 334990000.000 6.04 3.29 3.04 6.28 5.80

32506000.000 3.16810 335060000.000 6.04 3.29 3.04 6.28 5.80

32513000.000 3.16814 335130000.000 6.04 3.29 3.04 6.28 5.80

32520000.000 3.16817 335200000.000 6.04 3.29 3.04 6.28 5.80

32527000.000 3.16820 335270000.000 6.04 3.29 3.04 6.28 5.80

32534000.000 3.16824 335340000.000 6.04 3.29 3.04 6.28 5.80

32541000.000 3.16827 335410000.000 6.04 3.30 3.04 6.28 5.80

32548000.000 3.16831 335480000.000 6.04 3.30 3.04 6.28 5.80

32555000.000 3.16834 335550000.000 6.04 3.30 3.04 6.28 5.80

32562000.000 3.16838 335620000.000 6.04 3.30 3.04 6.28 5.80

32569000.000 3.16841 335690000.000 6.04 3.30 3.04 6.28 5.80

32576000.000 3.16844 335760000.000 6.04 3.30 3.04 6.28 5.80

32583000.000 3.16848 335830000.000 6.04 3.30 3.04 6.28 5.80

32590000.000 3.16851 335900000.000 6.04 3.30 3.04 6.28 5.80

32597000.000 3.16855 335970000.000 6.04 3.30 3.04 6.28 5.80

32604000.000 3.16858 336040000.000 6.04 3.30 3.04 6.28 5.80

32611000.000 3.16861 336110000.000 6.04 3.30 3.04 6.28 5.80

32618000.000 3.16865 336180000.000 6.04 3.30 3.04 6.28 5.80

32625000.000 3.16868 336250000.000 6.04 3.30 3.04 6.28 5.80

32632000.000 3.16872 336320000.000 6.04 3.30 3.04 6.28 5.80

32639000.000 3.16875 336390000.000 6.04 3.30 3.04 6.28 5.80

32646000.000 3.16878 336460000.000 6.04 3.30 3.04 6.28 5.80

32653000.000 3.16882 336530000.000 6.04 3.30 3.04 6.28 5.80

32660000.000 3.16885 336600000.000 6.04 3.30 3.04 6.28 5.80

32667000.000 3.16889 336670000.000 6.04 3.30 3.04 6.28 5.80

32674000.000 3.16892 336740000.000 6.04 3.30 3.04 6.28 5.80

32681000.000 3.16896 336810000.000 6.04 3.30 3.04 6.28 5.80

32688000.000 3.16899 336880000.000 6.04 3.30 3.04 6.28 5.80

32695000.000 3.16902 336950000.000 6.04 3.30 3.04 6.28 5.80

32702000.000 3.16906 337020000.000 6.04 3.30 3.04 6.28 5.80

32709000.000 3.16909 337090000.000 6.04 3.30 3.04 6.28 5.80

32716000.000 3.16913 337160000.000 6.04 3.30 3.04 6.28 5.80

32723000.000 3.16916 337230000.000 6.04 3.30 3.04 6.28 5.80

32730000.000 3.16919 337300000.000 6.04 3.30 3.04 6.28 5.80

32737000.000 3.16923 337370000.000 6.04 3.30 3.04 6.28 5.80

32744000.000 3.16926 337440000.000 6.04 3.30 3.04 6.28 5.80

32751000.000 3.16930 337510000.000 6.04 3.30 3.04 6.28 5.80

32758000.000 3.16933 337580000.000 6.04 3.30 3.04 6.28 5.80

32765000.000 3.16936 337650000.000 6.04 3.30 3.04 6.28 5.80

32772000.000 3.16940 337720000.000 6.04 3.30 3.04 6.28 5.80

32779000.000 3.16943 337790000.000 6.04 3.30 3.04 6.28 5.80

32786000.000 3.16947 337860000.000 6.04 3.30 3.04 6.28 5.80

32793000.000 3.16950 337930000.000 6.04 3.30 3.04 6.28 5.80

32800000.000 3.16953 338000000.000 6.04 3.30 3.04 6.28 5.80

32807000.000 3.16957 338070000.000 6.04 3.30 3.04 6.28 5.80

32814000.000 3.16960 338140000.000 6.04 3.30 3.04 6.28 5.80

32821000.000 3.16963 338210000.000 6.04 3.30 3.04 6.28 5.80

32828000.000 3.16967 338280000.000 6.04 3.30 3.04 6.28 5.80

32835000.000 3.16970 338350000.000 6.04 3.30 3.04 6.28 5.80

32842000.000 3.16974 338420000.000 6.04 3.30 3.04 6.28 5.80

32849000.000 3.16977 338490000.000 6.04 3.30 3.04 6.28 5.80

32856000.000 3.16980 338560000.000 6.04 3.30 3.04 6.28 5.80

32863000.000 3.16984 338630000.000 6.04 3.30 3.04 6.28 5.80

32870000.000 3.16987 338700000.000 6.04 3.30 3.04 6.28 5.80

32877000.000 3.16991 338770000.000 6.04 3.30 3.04 6.28 5.80

32884000.000 3.16994 338840000.000 6.04 3.30 3.04 6.28 5.80

32891000.000 3.16997 338910000.000 6.04 3.30 3.04 6.28 5.80

32898000.000 3.17001 338980000.000 6.04 3.30 3.04 6.28 5.80

32905000.000 3.17004 339050000.000 6.04 3.30 3.04 6.28 5.80

32912000.000 3.17008 339120000.000 6.04 3.30 3.04 6.28 5.80

32919000.000 3.17011 339190000.000 6.04 3.30 3.04 6.28 5.80

32926000.000 3.17014 339260000.000 6.04 3.30 3.04 6.28 5.80

32933000.000 3.17018 339330000.000 6.04 3.30 3.04 6.28 5.80

32940000.000 3.17021 339400000.000 6.04 3.30 3.04 6.28 5.80

32947000.000 3.17024 339470000.000 6.04 3.30 3.04 6.28 5.80

32954000.000 3.17028 339540000.000 6.04 3.30 3.04 6.28 5.80

32961000.000 3.17031 339610000.000 6.04 3.30 3.04 6.28 5.80

32968000.000 3.17035 339680000.000 6.04 3.30 3.04 6.28 5.80

32975000.000 3.17038 339750000.000 6.04 3.30 3.04 6.28 5.80

32982000.000 3.17041 339820000.000 6.04 3.30 3.04 6.28 5.80

32989000.000 3.17045 339890000.000 6.04 3.30 3.04 6.28 5.80

32996000.000 3.17048 339960000.000 6.04 3.30 3.04 6.28 5.80

33003000.000 3.17051 340030000.000 6.04 3.30 3.04 6.28 5.80

33010000.000 3.17055 340100000.000 6.04 3.30 3.04 6.28 5.80

33017000.000 3.17058 340170000.000 6.04 3.30 3.04 6.28 5.80

33024000.000 3.17062 340240000.000 6.04 3.30 3.04 6.28 5.80

33031000.000 3.17065 340310000.000 6.04 3.30 3.04 6.28 5.80

33038000.000 3.17068 340380000.000 6.04 3.30 3.04 6.28 5.80

33045000.000 3.17072 340450000.000 6.04 3.30 3.04 6.28 5.80

33052000.000 3.17075 340520000.000 6.04 3.30 3.04 6.28 5.80

33059000.000 3.17078 340590000.000 6.04 3.30 3.04 6.28 5.80

33066000.000 3.17082 340660000.000 6.04 3.30 3.04 6.28 5.80

33073000.000 3.17085 340730000.000 6.04 3.30 3.04 6.28 5.80

33080000.000 3.17088 340800000.000 6.04 3.30 3.04 6.28 5.80

33087000.000 3.17092 340870000.000 6.04 3.30 3.04 6.28 5.80

33094000.000 3.17095 340940000.000 6.04 3.30 3.04 6.28 5.80

33101000.000 3.17099 341010000.000 6.04 3.30 3.04 6.28 5.80

33108000.000 3.17102 341080000.000 6.04 3.30 3.04 6.28 5.80

33115000.000 3.17105 341150000.000 6.04 3.30 3.04 6.28 5.80

33122000.000 3.17109 341220000.000 6.04 3.30 3.04 6.28 5.80

33129000.000 3.17112 341290000.000 6.04 3.30 3.04 6.28 5.80

33136000.000 3.17115 341360000.000 6.04 3.30 3.04 6.29 5.80

33143000.000 3.17119 341430000.000 6.04 3.30 3.04 6.29 5.80

33150000.000 3.17122 341500000.000 6.04 3.30 3.04 6.29 5.80

33157000.000 3.17125 341570000.000 6.04 3.30 3.04 6.29 5.80

33164000.000 3.17129 341640000.000 6.04 3.30 3.04 6.29 5.80

33171000.000 3.17132 341710000.000 6.04 3.30 3.04 6.29 5.80

33178000.000 3.17136 341780000.000 6.04 3.30 3.04 6.29 5.80

33185000.000 3.17139 341850000.000 6.04 3.30 3.04 6.29 5.80

33192000.000 3.17142 341920000.000 6.04 3.30 3.04 6.29 5.80

33199000.000 3.17146 341990000.000 6.04 3.30 3.04 6.29 5.80

33206000.000 3.17149 342060000.000 6.04 3.30 3.04 6.29 5.80

33213000.000 3.17152 342130000.000 6.04 3.30 3.04 6.29 5.80

33220000.000 3.17156 342200000.000 6.04 3.30 3.04 6.29 5.80

33227000.000 3.17159 342270000.000 6.04 3.30 3.04 6.29 5.80

33234000.000 3.17162 342340000.000 6.04 3.30 3.04 6.29 5.80

33241000.000 3.17166 342410000.000 6.04 3.30 3.04 6.29 5.80

33248000.000 3.17169 342480000.000 6.04 3.30 3.04 6.29 5.80

33255000.000 3.17172 342550000.000 6.04 3.30 3.04 6.29 5.80

33262000.000 3.17176 342620000.000 6.04 3.30 3.04 6.29 5.80

33269000.000 3.17179 342690000.000 6.04 3.30 3.04 6.29 5.80

33276000.000 3.17182 342760000.000 6.04 3.30 3.04 6.29 5.80

33283000.000 3.17186 342830000.000 6.04 3.30 3.04 6.29 5.80

33290000.000 3.17189 342900000.000 6.04 3.30 3.05 6.29 5.80

33297000.000 3.17192 342970000.000 6.04 3.30 3.05 6.29 5.80

33304000.000 3.17196 343040000.000 6.04 3.30 3.05 6.29 5.80

33311000.000 3.17199 343110000.000 6.04 3.30 3.05 6.29 5.80

33318000.000 3.17203 343180000.000 6.04 3.30 3.05 6.29 5.80

33325000.000 3.17206 343250000.000 6.04 3.30 3.05 6.29 5.80

33332000.000 3.17209 343320000.000 6.05 3.30 3.05 6.29 5.80

33339000.000 3.17213 343390000.000 6.05 3.30 3.05 6.29 5.80

33346000.000 3.17216 343460000.000 6.05 3.30 3.05 6.29 5.80

33353000.000 3.17219 343530000.000 6.05 3.30 3.05 6.29 5.80

33360000.000 3.17223 343600000.000 6.05 3.30 3.05 6.29 5.80

33367000.000 3.17226 343670000.000 6.05 3.30 3.05 6.29 5.80

33374000.000 3.17229 343740000.000 6.05 3.30 3.05 6.29 5.80

33381000.000 3.17233 343810000.000 6.05 3.30 3.05 6.29 5.80

33388000.000 3.17236 343880000.000 6.05 3.30 3.05 6.29 5.80

33395000.000 3.17239 343950000.000 6.05 3.30 3.05 6.29 5.80

33402000.000 3.17243 344020000.000 6.05 3.30 3.05 6.29 5.80

33409000.000 3.17246 344090000.000 6.05 3.30 3.05 6.29 5.80

33416000.000 3.17249 344160000.000 6.05 3.30 3.05 6.29 5.80

33423000.000 3.17253 344230000.000 6.05 3.30 3.05 6.29 5.80

33430000.000 3.17256 344300000.000 6.05 3.30 3.05 6.29 5.80

33437000.000 3.17259 344370000.000 6.05 3.30 3.05 6.29 5.80

33444000.000 3.17263 344440000.000 6.05 3.30 3.05 6.29 5.80

33451000.000 3.17266 344510000.000 6.05 3.30 3.05 6.29 5.80

33458000.000 3.17269 344580000.000 6.05 3.30 3.05 6.29 5.80

33465000.000 3.17273 344650000.000 6.05 3.30 3.05 6.29 5.80

33472000.000 3.17276 344720000.000 6.05 3.30 3.05 6.29 5.80

33479000.000 3.17279 344790000.000 6.05 3.30 3.05 6.29 5.80

33486000.000 3.17283 344860000.000 6.05 3.30 3.05 6.29 5.80

33493000.000 3.17286 344930000.000 6.05 3.30 3.05 6.29 5.80

33500000.000 3.17289 345000000.000 6.05 3.30 3.05 6.29 5.80

33507000.000 3.17293 345070000.000 6.05 3.30 3.05 6.29 5.80

33514000.000 3.17296 345140000.000 6.05 3.30 3.05 6.29 5.80

33521000.000 3.17299 345210000.000 6.05 3.30 3.05 6.29 5.80

33528000.000 3.17303 345280000.000 6.05 3.30 3.05 6.29 5.80

33535000.000 3.17306 345350000.000 6.05 3.30 3.05 6.29 5.80

33542000.000 3.17309 345420000.000 6.05 3.30 3.05 6.29 5.81

33549000.000 3.17312 345490000.000 6.05 3.30 3.05 6.29 5.81

33556000.000 3.17316 345560000.000 6.05 3.30 3.05 6.29 5.81

33563000.000 3.17319 345630000.000 6.05 3.30 3.05 6.29 5.81

33570000.000 3.17322 345700000.000 6.05 3.30 3.05 6.29 5.81

33577000.000 3.17326 345770000.000 6.05 3.30 3.05 6.29 5.81

33584000.000 3.17329 345840000.000 6.05 3.30 3.05 6.29 5.81

33591000.000 3.17332 345910000.000 6.05 3.30 3.05 6.29 5.81

33598000.000 3.17336 345980000.000 6.05 3.30 3.05 6.29 5.81

33605000.000 3.17339 346050000.000 6.05 3.30 3.05 6.29 5.81

33612000.000 3.17342 346120000.000 6.05 3.30 3.05 6.29 5.81

33619000.000 3.17346 346190000.000 6.05 3.30 3.05 6.29 5.81

33626000.000 3.17349 346260000.000 6.05 3.30 3.05 6.29 5.81

33633000.000 3.17352 346330000.000 6.05 3.30 3.05 6.29 5.81

33640000.000 3.17356 346400000.000 6.05 3.30 3.05 6.29 5.81

33647000.000 3.17359 346470000.000 6.05 3.30 3.05 6.29 5.81

33654000.000 3.17362 346540000.000 6.05 3.30 3.05 6.29 5.81

33661000.000 3.17366 346610000.000 6.05 3.30 3.05 6.29 5.81

33668000.000 3.17369 346680000.000 6.05 3.30 3.05 6.29 5.81

33675000.000 3.17372 346750000.000 6.05 3.30 3.05 6.29 5.81

33682000.000 3.17375 346820000.000 6.05 3.30 3.05 6.29 5.81

33689000.000 3.17379 346890000.000 6.05 3.30 3.05 6.29 5.81

33696000.000 3.17382 346960000.000 6.05 3.30 3.05 6.29 5.81

33703000.000 3.17385 347030000.000 6.05 3.30 3.05 6.29 5.81

33710000.000 3.17389 347100000.000 6.05 3.30 3.05 6.29 5.81

33717000.000 3.17392 347170000.000 6.05 3.30 3.05 6.29 5.81

33724000.000 3.17395 347240000.000 6.05 3.30 3.05 6.29 5.81

33731000.000 3.17399 347310000.000 6.05 3.30 3.05 6.29 5.81

33738000.000 3.17402 347380000.000 6.05 3.30 3.05 6.29 5.81

33745000.000 3.17405 347450000.000 6.05 3.30 3.05 6.29 5.81

33752000.000 3.17408 347520000.000 6.05 3.30 3.05 6.29 5.81

33759000.000 3.17412 347590000.000 6.05 3.30 3.05 6.29 5.81

33766000.000 3.17415 347660000.000 6.05 3.30 3.05 6.29 5.81

33773000.000 3.17418 347730000.000 6.05 3.30 3.05 6.29 5.81

33780000.000 3.17422 347800000.000 6.05 3.30 3.05 6.29 5.81

33787000.000 3.17425 347870000.000 6.05 3.30 3.05 6.29 5.81

33794000.000 3.17428 347940000.000 6.05 3.30 3.05 6.29 5.81

33801000.000 3.17432 348010000.000 6.05 3.30 3.05 6.29 5.81

33808000.000 3.17435 348080000.000 6.05 3.30 3.05 6.29 5.81

33815000.000 3.17438 348150000.000 6.05 3.30 3.05 6.29 5.81

33822000.000 3.17441 348220000.000 6.05 3.30 3.05 6.29 5.81

33829000.000 3.17445 348290000.000 6.05 3.30 3.05 6.29 5.81

33836000.000 3.17448 348360000.000 6.05 3.30 3.05 6.29 5.81

33843000.000 3.17451 348430000.000 6.05 3.30 3.05 6.29 5.81

33850000.000 3.17455 348500000.000 6.05 3.30 3.05 6.29 5.81

33857000.000 3.17458 348570000.000 6.05 3.30 3.05 6.29 5.81

33864000.000 3.17461 348640000.000 6.05 3.30 3.05 6.29 5.81

33871000.000 3.17465 348710000.000 6.05 3.30 3.05 6.29 5.81

33878000.000 3.17468 348780000.000 6.05 3.30 3.05 6.29 5.81

33885000.000 3.17471 348850000.000 6.05 3.30 3.05 6.29 5.81

33892000.000 3.17474 348920000.000 6.05 3.30 3.05 6.29 5.81

33899000.000 3.17478 348990000.000 6.05 3.30 3.05 6.29 5.81

33906000.000 3.17481 349060000.000 6.05 3.30 3.05 6.29 5.81

33913000.000 3.17484 349130000.000 6.05 3.30 3.05 6.29 5.81

33920000.000 3.17488 349200000.000 6.05 3.30 3.05 6.29 5.81

33927000.000 3.17491 349270000.000 6.05 3.30 3.05 6.29 5.81

33934000.000 3.17494 349340000.000 6.05 3.30 3.05 6.29 5.81

33941000.000 3.17497 349410000.000 6.05 3.30 3.05 6.29 5.81

33948000.000 3.17501 349480000.000 6.05 3.30 3.05 6.29 5.81

33955000.000 3.17504 349550000.000 6.05 3.30 3.05 6.29 5.81

33962000.000 3.17507 349620000.000 6.05 3.30 3.05 6.29 5.81

33969000.000 3.17511 349690000.000 6.05 3.30 3.05 6.29 5.81

33976000.000 3.17514 349760000.000 6.05 3.30 3.05 6.29 5.81

33983000.000 3.17517 349830000.000 6.05 3.30 3.05 6.29 5.81

33990000.000 3.17520 349900000.000 6.05 3.30 3.05 6.29 5.81

33997000.000 3.17524 349970000.000 6.05 3.30 3.05 6.29 5.81

34004000.000 3.17527 350040000.000 6.05 3.30 3.05 6.29 5.81

34011000.000 3.17530 350110000.000 6.05 3.30 3.05 6.29 5.81

34018000.000 3.17534 350180000.000 6.05 3.30 3.05 6.29 5.81

34025000.000 3.17537 350250000.000 6.05 3.30 3.05 6.29 5.81

34032000.000 3.17540 350320000.000 6.05 3.30 3.05 6.29 5.81

34039000.000 3.17543 350390000.000 6.05 3.30 3.05 6.29 5.81

34046000.000 3.17547 350460000.000 6.05 3.30 3.05 6.29 5.81

34053000.000 3.17550 350530000.000 6.05 3.30 3.05 6.29 5.81

34060000.000 3.17553 350600000.000 6.05 3.30 3.05 6.29 5.81

34067000.000 3.17556 350670000.000 6.05 3.30 3.05 6.29 5.81

34074000.000 3.17560 350740000.000 6.05 3.30 3.05 6.29 5.81

34081000.000 3.17563 350810000.000 6.05 3.30 3.05 6.29 5.81

34088000.000 3.17566 350880000.000 6.05 3.30 3.05 6.29 5.81

34095000.000 3.17570 350950000.000 6.05 3.30 3.05 6.29 5.81

34102000.000 3.17573 351020000.000 6.05 3.30 3.05 6.29 5.81

34109000.000 3.17576 351090000.000 6.05 3.30 3.05 6.29 5.81

34116000.000 3.17579 351160000.000 6.05 3.30 3.05 6.29 5.81

34123000.000 3.17583 351230000.000 6.05 3.30 3.05 6.29 5.81

34130000.000 3.17586 351300000.000 6.05 3.30 3.05 6.29 5.81

34137000.000 3.17589 351370000.000 6.05 3.30 3.05 6.29 5.81

34144000.000 3.17592 351440000.000 6.05 3.30 3.05 6.29 5.81

34151000.000 3.17596 351510000.000 6.05 3.30 3.05 6.29 5.81

34158000.000 3.17599 351580000.000 6.05 3.30 3.05 6.29 5.81

34165000.000 3.17602 351650000.000 6.05 3.30 3.05 6.29 5.81

34172000.000 3.17605 351720000.000 6.05 3.30 3.05 6.29 5.81

34179000.000 3.17609 351790000.000 6.05 3.30 3.05 6.29 5.81

34186000.000 3.17612 351860000.000 6.05 3.30 3.05 6.29 5.81

34193000.000 3.17615 351930000.000 6.05 3.30 3.05 6.29 5.81

34200000.000 3.17619 352000000.000 6.05 3.30 3.05 6.29 5.81

34207000.000 3.17622 352070000.000 6.05 3.30 3.05 6.29 5.81

34214000.000 3.17625 352140000.000 6.05 3.30 3.05 6.29 5.81

34221000.000 3.17628 352210000.000 6.05 3.30 3.05 6.29 5.81

34228000.000 3.17632 352280000.000 6.05 3.30 3.05 6.29 5.81

34235000.000 3.17635 352350000.000 6.05 3.30 3.05 6.30 5.81

34242000.000 3.17638 352420000.000 6.05 3.30 3.05 6.30 5.81

34249000.000 3.17641 352490000.000 6.05 3.30 3.05 6.30 5.81

34256000.000 3.17645 352560000.000 6.05 3.30 3.05 6.30 5.81

34263000.000 3.17648 352630000.000 6.05 3.30 3.05 6.30 5.81

34270000.000 3.17651 352700000.000 6.05 3.30 3.05 6.30 5.81

34277000.000 3.17654 352770000.000 6.05 3.30 3.05 6.30 5.81

34284000.000 3.17658 352840000.000 6.05 3.30 3.05 6.30 5.81

34291000.000 3.17661 352910000.000 6.05 3.30 3.05 6.30 5.81

34298000.000 3.17664 352980000.000 6.05 3.30 3.05 6.30 5.81

34305000.000 3.17667 353050000.000 6.05 3.30 3.05 6.30 5.81

34312000.000 3.17671 353120000.000 6.05 3.30 3.05 6.30 5.81

34319000.000 3.17674 353190000.000 6.05 3.30 3.05 6.30 5.81

34326000.000 3.17677 353260000.000 6.05 3.30 3.05 6.30 5.81

34333000.000 3.17680 353330000.000 6.05 3.30 3.05 6.30 5.81

34340000.000 3.17684 353400000.000 6.05 3.30 3.05 6.30 5.81

34347000.000 3.17687 353470000.000 6.05 3.30 3.05 6.30 5.81

34354000.000 3.17690 353540000.000 6.05 3.30 3.05 6.30 5.81

34361000.000 3.17693 353610000.000 6.05 3.30 3.05 6.30 5.81

34368000.000 3.17697 353680000.000 6.05 3.30 3.05 6.30 5.81

34375000.000 3.17700 353750000.000 6.05 3.30 3.05 6.30 5.81

34382000.000 3.17703 353820000.000 6.05 3.30 3.05 6.30 5.81

34389000.000 3.17706 353890000.000 6.05 3.30 3.05 6.30 5.81

34396000.000 3.17710 353960000.000 6.05 3.30 3.05 6.30 5.81

34403000.000 3.17713 354030000.000 6.05 3.30 3.05 6.30 5.81

34410000.000 3.17716 354100000.000 6.05 3.30 3.05 6.30 5.81

34417000.000 3.17719 354170000.000 6.05 3.30 3.05 6.30 5.81

34424000.000 3.17723 354240000.000 6.05 3.30 3.05 6.30 5.81

34431000.000 3.17726 354310000.000 6.05 3.30 3.05 6.30 5.81

34438000.000 3.17729 354380000.000 6.05 3.30 3.05 6.30 5.81

34445000.000 3.17732 354450000.000 6.05 3.30 3.05 6.30 5.81

34452000.000 3.17735 354520000.000 6.05 3.30 3.05 6.30 5.81

34459000.000 3.17739 354590000.000 6.05 3.30 3.05 6.30 5.81

34466000.000 3.17742 354660000.000 6.05 3.30 3.05 6.30 5.81

34473000.000 3.17745 354730000.000 6.05 3.30 3.05 6.30 5.81

34480000.000 3.17748 354800000.000 6.06 3.30 3.05 6.30 5.81

34487000.000 3.17752 354870000.000 6.06 3.30 3.05 6.30 5.81

34494000.000 3.17755 354940000.000 6.06 3.30 3.05 6.30 5.81

34501000.000 3.17758 355010000.000 6.06 3.30 3.05 6.30 5.81

34508000.000 3.17761 355080000.000 6.06 3.30 3.05 6.30 5.81

34515000.000 3.17765 355150000.000 6.06 3.30 3.05 6.30 5.81

34522000.000 3.17768 355220000.000 6.06 3.30 3.05 6.30 5.81

34529000.000 3.17771 355290000.000 6.06 3.30 3.05 6.30 5.81

34536000.000 3.17774 355360000.000 6.06 3.30 3.05 6.30 5.81

34543000.000 3.17778 355430000.000 6.06 3.30 3.05 6.30 5.81

34550000.000 3.17781 355500000.000 6.06 3.30 3.05 6.30 5.81

34557000.000 3.17784 355570000.000 6.06 3.30 3.05 6.30 5.81

34564000.000 3.17787 355640000.000 6.06 3.30 3.05 6.30 5.81

34571000.000 3.17790 355710000.000 6.06 3.31 3.05 6.30 5.81

34578000.000 3.17794 355780000.000 6.06 3.31 3.05 6.30 5.81

34585000.000 3.17797 355850000.000 6.06 3.31 3.05 6.30 5.81

34592000.000 3.17800 355920000.000 6.06 3.31 3.05 6.30 5.81

34599000.000 3.17803 355990000.000 6.06 3.31 3.05 6.30 5.81

34606000.000 3.17807 356060000.000 6.06 3.31 3.05 6.30 5.81

34613000.000 3.17810 356130000.000 6.06 3.31 3.05 6.30 5.81

34620000.000 3.17813 356200000.000 6.06 3.31 3.05 6.30 5.81

34627000.000 3.17816 356270000.000 6.06 3.31 3.05 6.30 5.81

34634000.000 3.17819 356340000.000 6.06 3.31 3.05 6.30 5.81

34641000.000 3.17823 356410000.000 6.06 3.31 3.05 6.30 5.81

34648000.000 3.17826 356480000.000 6.06 3.31 3.05 6.30 5.81

34655000.000 3.17829 356550000.000 6.06 3.31 3.05 6.30 5.81

34662000.000 3.17832 356620000.000 6.06 3.31 3.05 6.30 5.81

34669000.000 3.17836 356690000.000 6.06 3.31 3.05 6.30 5.81

34676000.000 3.17839 356760000.000 6.06 3.31 3.05 6.30 5.81

34683000.000 3.17842 356830000.000 6.06 3.31 3.05 6.30 5.81

34690000.000 3.17845 356900000.000 6.06 3.31 3.05 6.30 5.81

34697000.000 3.17848 356970000.000 6.06 3.31 3.05 6.30 5.81

34704000.000 3.17852 357040000.000 6.06 3.31 3.05 6.30 5.81

34711000.000 3.17855 357110000.000 6.06 3.31 3.05 6.30 5.81

34718000.000 3.17858 357180000.000 6.06 3.31 3.05 6.30 5.81

34725000.000 3.17861 357250000.000 6.06 3.31 3.05 6.30 5.81

34732000.000 3.17865 357320000.000 6.06 3.31 3.05 6.30 5.81

34739000.000 3.17868 357390000.000 6.06 3.31 3.05 6.30 5.81

34746000.000 3.17871 357460000.000 6.06 3.31 3.05 6.30 5.81

34753000.000 3.17874 357530000.000 6.06 3.31 3.05 6.30 5.82

34760000.000 3.17877 357600000.000 6.06 3.31 3.05 6.30 5.82

34767000.000 3.17881 357670000.000 6.06 3.31 3.05 6.30 5.82

34774000.000 3.17884 357740000.000 6.06 3.31 3.05 6.30 5.82

34781000.000 3.17887 357810000.000 6.06 3.31 3.05 6.30 5.82

34788000.000 3.17890 357880000.000 6.06 3.31 3.05 6.30 5.82

34795000.000 3.17893 357950000.000 6.06 3.31 3.05 6.30 5.82

34802000.000 3.17897 358020000.000 6.06 3.31 3.05 6.30 5.82

34809000.000 3.17900 358090000.000 6.06 3.31 3.05 6.30 5.82

34816000.000 3.17903 358160000.000 6.06 3.31 3.05 6.30 5.82

34823000.000 3.17906 358230000.000 6.06 3.31 3.05 6.30 5.82

34830000.000 3.17909 358300000.000 6.06 3.31 3.05 6.30 5.82

34837000.000 3.17913 358370000.000 6.06 3.31 3.05 6.30 5.82

34844000.000 3.17916 358440000.000 6.06 3.31 3.05 6.30 5.82

34851000.000 3.17919 358510000.000 6.06 3.31 3.05 6.30 5.82

34858000.000 3.17922 358580000.000 6.06 3.31 3.05 6.30 5.82

34865000.000 3.17925 358650000.000 6.06 3.31 3.05 6.30 5.82

34872000.000 3.17929 358720000.000 6.06 3.31 3.05 6.30 5.82

34879000.000 3.17932 358790000.000 6.06 3.31 3.05 6.30 5.82

34886000.000 3.17935 358860000.000 6.06 3.31 3.05 6.30 5.82

34893000.000 3.17938 358930000.000 6.06 3.31 3.05 6.30 5.82

34900000.000 3.17941 359000000.000 6.06 3.31 3.05 6.30 5.82

34907000.000 3.17945 359070000.000 6.06 3.31 3.05 6.30 5.82

34914000.000 3.17948 359140000.000 6.06 3.31 3.05 6.30 5.82

34921000.000 3.17951 359210000.000 6.06 3.31 3.05 6.30 5.82

34928000.000 3.17954 359280000.000 6.06 3.31 3.05 6.30 5.82

34935000.000 3.17957 359350000.000 6.06 3.31 3.05 6.30 5.82

34942000.000 3.17961 359420000.000 6.06 3.31 3.05 6.30 5.82

34949000.000 3.17964 359490000.000 6.06 3.31 3.05 6.30 5.82

34956000.000 3.17967 359560000.000 6.06 3.31 3.05 6.30 5.82

34963000.000 3.17970 359630000.000 6.06 3.31 3.05 6.30 5.82

34970000.000 3.17973 359700000.000 6.06 3.31 3.05 6.30 5.82

34977000.000 3.17977 359770000.000 6.06 3.31 3.05 6.30 5.82

34984000.000 3.17980 359840000.000 6.06 3.31 3.05 6.30 5.82

34991000.000 3.17983 359910000.000 6.06 3.31 3.05 6.30 5.82

34998000.000 3.17986 359980000.000 6.06 3.31 3.05 6.30 5.82

35005000.000 3.17989 360050000.000 6.06 3.31 3.05 6.30 5.82

35012000.000 3.17993 360120000.000 6.06 3.31 3.05 6.30 5.82

35019000.000 3.17996 360190000.000 6.06 3.31 3.05 6.30 5.82

35026000.000 3.17999 360260000.000 6.06 3.31 3.05 6.30 5.82

35033000.000 3.18002 360330000.000 6.06 3.31 3.05 6.30 5.82

35040000.000 3.18005 360400000.000 6.06 3.31 3.05 6.30 5.82

35047000.000 3.18009 360470000.000 6.06 3.31 3.05 6.30 5.82

35054000.000 3.18012 360540000.000 6.06 3.31 3.05 6.30 5.82

35061000.000 3.18015 360610000.000 6.06 3.31 3.05 6.30 5.82

35068000.000 3.18018 360680000.000 6.06 3.31 3.05 6.30 5.82

35075000.000 3.18021 360750000.000 6.06 3.31 3.05 6.30 5.82

35082000.000 3.18024 360820000.000 6.06 3.31 3.05 6.30 5.82

35089000.000 3.18028 360890000.000 6.06 3.31 3.05 6.30 5.82

35096000.000 3.18031 360960000.000 6.06 3.31 3.05 6.30 5.82

35103000.000 3.18034 361030000.000 6.06 3.31 3.05 6.30 5.82

35110000.000 3.18037 361100000.000 6.06 3.31 3.05 6.30 5.82

35117000.000 3.18040 361170000.000 6.06 3.31 3.05 6.30 5.82

35124000.000 3.18044 361240000.000 6.06 3.31 3.05 6.30 5.82

35131000.000 3.18047 361310000.000 6.06 3.31 3.05 6.30 5.82

35138000.000 3.18050 361380000.000 6.06 3.31 3.05 6.30 5.82

35145000.000 3.18053 361450000.000 6.06 3.31 3.05 6.30 5.82

35152000.000 3.18056 361520000.000 6.06 3.31 3.05 6.30 5.82

35159000.000 3.18059 361590000.000 6.06 3.31 3.05 6.30 5.82

35166000.000 3.18063 361660000.000 6.06 3.31 3.05 6.30 5.82

35173000.000 3.18066 361730000.000 6.06 3.31 3.05 6.30 5.82

35180000.000 3.18069 361800000.000 6.06 3.31 3.05 6.30 5.82

35187000.000 3.18072 361870000.000 6.06 3.31 3.05 6.30 5.82

35194000.000 3.18075 361940000.000 6.06 3.31 3.05 6.30 5.82

35201000.000 3.18078 362010000.000 6.06 3.31 3.05 6.30 5.82

35208000.000 3.18082 362080000.000 6.06 3.31 3.05 6.30 5.82

35215000.000 3.18085 362150000.000 6.06 3.31 3.05 6.30 5.82

35222000.000 3.18088 362220000.000 6.06 3.31 3.05 6.30 5.82

35229000.000 3.18091 362290000.000 6.06 3.31 3.05 6.30 5.82

35236000.000 3.18094 362360000.000 6.06 3.31 3.05 6.30 5.82

35243000.000 3.18097 362430000.000 6.06 3.31 3.05 6.30 5.82

35250000.000 3.18101 362500000.000 6.06 3.31 3.05 6.30 5.82

35257000.000 3.18104 362570000.000 6.06 3.31 3.05 6.30 5.82

35264000.000 3.18107 362640000.000 6.06 3.31 3.05 6.30 5.82

35271000.000 3.18110 362710000.000 6.06 3.31 3.05 6.30 5.82

35278000.000 3.18113 362780000.000 6.06 3.31 3.05 6.30 5.82

35285000.000 3.18116 362850000.000 6.06 3.31 3.05 6.30 5.82

35292000.000 3.18120 362920000.000 6.06 3.31 3.05 6.30 5.82

35299000.000 3.18123 362990000.000 6.06 3.31 3.05 6.30 5.82

35306000.000 3.18126 363060000.000 6.06 3.31 3.05 6.30 5.82

35313000.000 3.18129 363130000.000 6.06 3.31 3.05 6.30 5.82

35320000.000 3.18132 363200000.000 6.06 3.31 3.05 6.30 5.82

35327000.000 3.18135 363270000.000 6.06 3.31 3.05 6.30 5.82

35334000.000 3.18139 363340000.000 6.06 3.31 3.05 6.30 5.82

35341000.000 3.18142 363410000.000 6.06 3.31 3.05 6.30 5.82

35348000.000 3.18145 363480000.000 6.06 3.31 3.05 6.30 5.82

35355000.000 3.18148 363550000.000 6.06 3.31 3.05 6.30 5.82

35362000.000 3.18151 363620000.000 6.06 3.31 3.05 6.30 5.82

35369000.000 3.18154 363690000.000 6.06 3.31 3.05 6.31 5.82

35376000.000 3.18158 363760000.000 6.06 3.31 3.05 6.31 5.82

35383000.000 3.18161 363830000.000 6.06 3.31 3.05 6.31 5.82

35390000.000 3.18164 363900000.000 6.06 3.31 3.05 6.31 5.82

35397000.000 3.18167 363970000.000 6.06 3.31 3.05 6.31 5.82

35404000.000 3.18170 364040000.000 6.06 3.31 3.05 6.31 5.82

35411000.000 3.18173 364110000.000 6.06 3.31 3.05 6.31 5.82

35418000.000 3.18177 364180000.000 6.06 3.31 3.05 6.31 5.82

35425000.000 3.18180 364250000.000 6.06 3.31 3.05 6.31 5.82

35432000.000 3.18183 364320000.000 6.06 3.31 3.05 6.31 5.82

35439000.000 3.18186 364390000.000 6.06 3.31 3.05 6.31 5.82

35446000.000 3.18189 364460000.000 6.06 3.31 3.05 6.31 5.82

35453000.000 3.18192 364530000.000 6.06 3.31 3.05 6.31 5.82

35460000.000 3.18195 364600000.000 6.06 3.31 3.05 6.31 5.82

35467000.000 3.18199 364670000.000 6.06 3.31 3.05 6.31 5.82

35474000.000 3.18202 364740000.000 6.06 3.31 3.05 6.31 5.82

35481000.000 3.18205 364810000.000 6.06 3.31 3.05 6.31 5.82

35488000.000 3.18208 364880000.000 6.06 3.31 3.05 6.31 5.82

35495000.000 3.18211 364950000.000 6.06 3.31 3.05 6.31 5.82

35502000.000 3.18214 365020000.000 6.06 3.31 3.05 6.31 5.82

35509000.000 3.18217 365090000.000 6.06 3.31 3.05 6.31 5.82

35516000.000 3.18221 365160000.000 6.06 3.31 3.05 6.31 5.82

35523000.000 3.18224 365230000.000 6.06 3.31 3.05 6.31 5.82

35530000.000 3.18227 365300000.000 6.06 3.31 3.05 6.31 5.82

35537000.000 3.18230 365370000.000 6.06 3.31 3.06 6.31 5.82

35544000.000 3.18233 365440000.000 6.06 3.31 3.06 6.31 5.82

35551000.000 3.18236 365510000.000 6.06 3.31 3.06 6.31 5.82

35558000.000 3.18239 365580000.000 6.06 3.31 3.06 6.31 5.82

35565000.000 3.18243 365650000.000 6.06 3.31 3.06 6.31 5.82

35572000.000 3.18246 365720000.000 6.06 3.31 3.06 6.31 5.82

35579000.000 3.18249 365790000.000 6.06 3.31 3.06 6.31 5.82

35586000.000 3.18252 365860000.000 6.06 3.31 3.06 6.31 5.82

35593000.000 3.18255 365930000.000 6.06 3.31 3.06 6.31 5.82

35600000.000 3.18258 366000000.000 6.06 3.31 3.06 6.31 5.82

35607000.000 3.18261 366070000.000 6.06 3.31 3.06 6.31 5.82

35614000.000 3.18265 366140000.000 6.06 3.31 3.06 6.31 5.82

35621000.000 3.18268 366210000.000 6.06 3.31 3.06 6.31 5.82

35628000.000 3.18271 366280000.000 6.06 3.31 3.06 6.31 5.82

35635000.000 3.18274 366350000.000 6.06 3.31 3.06 6.31 5.82

35642000.000 3.18277 366420000.000 6.06 3.31 3.06 6.31 5.82

35649000.000 3.18280 366490000.000 6.06 3.31 3.06 6.31 5.82

35656000.000 3.18283 366560000.000 6.06 3.31 3.06 6.31 5.82

35663000.000 3.18287 366630000.000 6.06 3.31 3.06 6.31 5.82

35670000.000 3.18290 366700000.000 6.07 3.31 3.06 6.31 5.82

35677000.000 3.18293 366770000.000 6.07 3.31 3.06 6.31 5.82

35684000.000 3.18296 366840000.000 6.07 3.31 3.06 6.31 5.82

35691000.000 3.18299 366910000.000 6.07 3.31 3.06 6.31 5.82

35698000.000 3.18302 366980000.000 6.07 3.31 3.06 6.31 5.82

35705000.000 3.18305 367050000.000 6.07 3.31 3.06 6.31 5.82

35712000.000 3.18308 367120000.000 6.07 3.31 3.06 6.31 5.82

35719000.000 3.18312 367190000.000 6.07 3.31 3.06 6.31 5.82

35726000.000 3.18315 367260000.000 6.07 3.31 3.06 6.31 5.82

35733000.000 3.18318 367330000.000 6.07 3.31 3.06 6.31 5.82

35740000.000 3.18321 367400000.000 6.07 3.31 3.06 6.31 5.82

35747000.000 3.18324 367470000.000 6.07 3.31 3.06 6.31 5.82

35754000.000 3.18327 367540000.000 6.07 3.31 3.06 6.31 5.82

35761000.000 3.18330 367610000.000 6.07 3.31 3.06 6.31 5.82

35768000.000 3.18333 367680000.000 6.07 3.31 3.06 6.31 5.82

35775000.000 3.18337 367750000.000 6.07 3.31 3.06 6.31 5.82

35782000.000 3.18340 367820000.000 6.07 3.31 3.06 6.31 5.82

35789000.000 3.18343 367890000.000 6.07 3.31 3.06 6.31 5.82

35796000.000 3.18346 367960000.000 6.07 3.31 3.06 6.31 5.82

35803000.000 3.18349 368030000.000 6.07 3.31 3.06 6.31 5.82

35810000.000 3.18352 368100000.000 6.07 3.31 3.06 6.31 5.82

35817000.000 3.18355 368170000.000 6.07 3.31 3.06 6.31 5.82

35824000.000 3.18358 368240000.000 6.07 3.31 3.06 6.31 5.82

35831000.000 3.18362 368310000.000 6.07 3.31 3.06 6.31 5.82

35838000.000 3.18365 368380000.000 6.07 3.31 3.06 6.31 5.82

35845000.000 3.18368 368450000.000 6.07 3.31 3.06 6.31 5.82

35852000.000 3.18371 368520000.000 6.07 3.31 3.06 6.31 5.82

35859000.000 3.18374 368590000.000 6.07 3.31 3.06 6.31 5.82

35866000.000 3.18377 368660000.000 6.07 3.31 3.06 6.31 5.82

35873000.000 3.18380 368730000.000 6.07 3.31 3.06 6.31 5.82

35880000.000 3.18383 368800000.000 6.07 3.31 3.06 6.31 5.82

35887000.000 3.18387 368870000.000 6.07 3.31 3.06 6.31 5.82

35894000.000 3.18390 368940000.000 6.07 3.31 3.06 6.31 5.82

35901000.000 3.18393 369010000.000 6.07 3.31 3.06 6.31 5.82

35908000.000 3.18396 369080000.000 6.07 3.31 3.06 6.31 5.82

35915000.000 3.18399 369150000.000 6.07 3.31 3.06 6.31 5.82

35922000.000 3.18402 369220000.000 6.07 3.31 3.06 6.31 5.82

35929000.000 3.18405 369290000.000 6.07 3.31 3.06 6.31 5.82

35936000.000 3.18408 369360000.000 6.07 3.31 3.06 6.31 5.82

35943000.000 3.18411 369430000.000 6.07 3.31 3.06 6.31 5.82

35950000.000 3.18415 369500000.000 6.07 3.31 3.06 6.31 5.82

35957000.000 3.18418 369570000.000 6.07 3.31 3.06 6.31 5.82

35964000.000 3.18421 369640000.000 6.07 3.31 3.06 6.31 5.82

35971000.000 3.18424 369710000.000 6.07 3.31 3.06 6.31 5.82

35978000.000 3.18427 369780000.000 6.07 3.31 3.06 6.31 5.82

35985000.000 3.18430 369850000.000 6.07 3.31 3.06 6.31 5.82

35992000.000 3.18433 369920000.000 6.07 3.31 3.06 6.31 5.82

35999000.000 3.18436 369990000.000 6.07 3.31 3.06 6.31 5.83

36006000.000 3.18439 370060000.000 6.07 3.31 3.06 6.31 5.83

36013000.000 3.18443 370130000.000 6.07 3.31 3.06 6.31 5.83

36020000.000 3.18446 370200000.000 6.07 3.31 3.06 6.31 5.83

36027000.000 3.18449 370270000.000 6.07 3.31 3.06 6.31 5.83

36034000.000 3.18452 370340000.000 6.07 3.31 3.06 6.31 5.83

36041000.000 3.18455 370410000.000 6.07 3.31 3.06 6.31 5.83

36048000.000 3.18458 370480000.000 6.07 3.31 3.06 6.31 5.83

36055000.000 3.18461 370550000.000 6.07 3.31 3.06 6.31 5.83

36062000.000 3.18464 370620000.000 6.07 3.31 3.06 6.31 5.83

36069000.000 3.18467 370690000.000 6.07 3.31 3.06 6.31 5.83

36076000.000 3.18470 370760000.000 6.07 3.31 3.06 6.31 5.83

36083000.000 3.18474 370830000.000 6.07 3.31 3.06 6.31 5.83

36090000.000 3.18477 370900000.000 6.07 3.31 3.06 6.31 5.83

36097000.000 3.18480 370970000.000 6.07 3.31 3.06 6.31 5.83

36104000.000 3.18483 371040000.000 6.07 3.31 3.06 6.31 5.83

36111000.000 3.18486 371110000.000 6.07 3.31 3.06 6.31 5.83

36118000.000 3.18489 371180000.000 6.07 3.31 3.06 6.31 5.83

36125000.000 3.18492 371250000.000 6.07 3.31 3.06 6.31 5.83

36132000.000 3.18495 371320000.000 6.07 3.31 3.06 6.31 5.83

36139000.000 3.18498 371390000.000 6.07 3.31 3.06 6.31 5.83

36146000.000 3.18501 371460000.000 6.07 3.31 3.06 6.31 5.83

36153000.000 3.18504 371530000.000 6.07 3.31 3.06 6.31 5.83

36160000.000 3.18508 371600000.000 6.07 3.31 3.06 6.31 5.83

36167000.000 3.18511 371670000.000 6.07 3.31 3.06 6.31 5.83

36174000.000 3.18514 371740000.000 6.07 3.31 3.06 6.31 5.83

36181000.000 3.18517 371810000.000 6.07 3.31 3.06 6.31 5.83

36188000.000 3.18520 371880000.000 6.07 3.31 3.06 6.31 5.83

36195000.000 3.18523 371950000.000 6.07 3.31 3.06 6.31 5.83

36202000.000 3.18526 372020000.000 6.07 3.31 3.06 6.31 5.83

36209000.000 3.18529 372090000.000 6.07 3.31 3.06 6.31 5.83

36216000.000 3.18532 372160000.000 6.07 3.31 3.06 6.31 5.83

36223000.000 3.18535 372230000.000 6.07 3.31 3.06 6.31 5.83

36230000.000 3.18538 372300000.000 6.07 3.31 3.06 6.31 5.83

36237000.000 3.18542 372370000.000 6.07 3.31 3.06 6.31 5.83

36244000.000 3.18545 372440000.000 6.07 3.31 3.06 6.31 5.83

36251000.000 3.18548 372510000.000 6.07 3.31 3.06 6.31 5.83

36258000.000 3.18551 372580000.000 6.07 3.31 3.06 6.31 5.83

36265000.000 3.18554 372650000.000 6.07 3.31 3.06 6.31 5.83

36272000.000 3.18557 372720000.000 6.07 3.31 3.06 6.31 5.83

36279000.000 3.18560 372790000.000 6.07 3.31 3.06 6.31 5.83

36286000.000 3.18563 372860000.000 6.07 3.31 3.06 6.31 5.83

36293000.000 3.18566 372930000.000 6.07 3.31 3.06 6.31 5.83

36300000.000 3.18569 373000000.000 6.07 3.31 3.06 6.31 5.83

36307000.000 3.18572 373070000.000 6.07 3.31 3.06 6.31 5.83

36314000.000 3.18575 373140000.000 6.07 3.31 3.06 6.31 5.83

36321000.000 3.18579 373210000.000 6.07 3.31 3.06 6.31 5.83

36328000.000 3.18582 373280000.000 6.07 3.31 3.06 6.31 5.83

36335000.000 3.18585 373350000.000 6.07 3.31 3.06 6.31 5.83

36342000.000 3.18588 373420000.000 6.07 3.31 3.06 6.31 5.83

36349000.000 3.18591 373490000.000 6.07 3.31 3.06 6.31 5.83

36356000.000 3.18594 373560000.000 6.07 3.31 3.06 6.31 5.83

36363000.000 3.18597 373630000.000 6.07 3.31 3.06 6.31 5.83

36370000.000 3.18600 373700000.000 6.07 3.31 3.06 6.31 5.83

36377000.000 3.18603 373770000.000 6.07 3.31 3.06 6.31 5.83

36384000.000 3.18606 373840000.000 6.07 3.31 3.06 6.31 5.83

36391000.000 3.18609 373910000.000 6.07 3.31 3.06 6.31 5.83

36398000.000 3.18612 373980000.000 6.07 3.31 3.06 6.31 5.83

36405000.000 3.18615 374050000.000 6.07 3.31 3.06 6.31 5.83

36412000.000 3.18619 374120000.000 6.07 3.31 3.06 6.31 5.83

36419000.000 3.18622 374190000.000 6.07 3.31 3.06 6.31 5.83

36426000.000 3.18625 374260000.000 6.07 3.31 3.06 6.31 5.83

36433000.000 3.18628 374330000.000 6.07 3.31 3.06 6.31 5.83

36440000.000 3.18631 374400000.000 6.07 3.31 3.06 6.31 5.83

36447000.000 3.18634 374470000.000 6.07 3.31 3.06 6.31 5.83

36454000.000 3.18637 374540000.000 6.07 3.31 3.06 6.31 5.83

36461000.000 3.18640 374610000.000 6.07 3.31 3.06 6.31 5.83

36468000.000 3.18643 374680000.000 6.07 3.31 3.06 6.31 5.83

36475000.000 3.18646 374750000.000 6.07 3.31 3.06 6.31 5.83

36482000.000 3.18649 374820000.000 6.07 3.31 3.06 6.31 5.83

36489000.000 3.18652 374890000.000 6.07 3.31 3.06 6.31 5.83

36496000.000 3.18655 374960000.000 6.07 3.31 3.06 6.31 5.83

36503000.000 3.18658 375030000.000 6.07 3.31 3.06 6.31 5.83

36510000.000 3.18662 375100000.000 6.07 3.31 3.06 6.31 5.83

36517000.000 3.18665 375170000.000 6.07 3.31 3.06 6.31 5.83

36524000.000 3.18668 375240000.000 6.07 3.31 3.06 6.31 5.83

36531000.000 3.18671 375310000.000 6.07 3.31 3.06 6.31 5.83

36538000.000 3.18674 375380000.000 6.07 3.31 3.06 6.32 5.83

36545000.000 3.18677 375450000.000 6.07 3.31 3.06 6.32 5.83

36552000.000 3.18680 375520000.000 6.07 3.31 3.06 6.32 5.83

36559000.000 3.18683 375590000.000 6.07 3.31 3.06 6.32 5.83

36566000.000 3.18686 375660000.000 6.07 3.31 3.06 6.32 5.83

36573000.000 3.18689 375730000.000 6.07 3.31 3.06 6.32 5.83

36580000.000 3.18692 375800000.000 6.07 3.31 3.06 6.32 5.83

36587000.000 3.18695 375870000.000 6.07 3.31 3.06 6.32 5.83

36594000.000 3.18698 375940000.000 6.07 3.31 3.06 6.32 5.83

36601000.000 3.18701 376010000.000 6.07 3.31 3.06 6.32 5.83

36608000.000 3.18704 376080000.000 6.07 3.31 3.06 6.32 5.83

36615000.000 3.18707 376150000.000 6.07 3.31 3.06 6.32 5.83

36622000.000 3.18710 376220000.000 6.07 3.31 3.06 6.32 5.83

36629000.000 3.18714 376290000.000 6.07 3.31 3.06 6.32 5.83

36636000.000 3.18717 376360000.000 6.07 3.31 3.06 6.32 5.83

36643000.000 3.18720 376430000.000 6.07 3.31 3.06 6.32 5.83

36650000.000 3.18723 376500000.000 6.07 3.31 3.06 6.32 5.83

36657000.000 3.18726 376570000.000 6.07 3.31 3.06 6.32 5.83

36664000.000 3.18729 376640000.000 6.07 3.31 3.06 6.32 5.83

36671000.000 3.18732 376710000.000 6.07 3.31 3.06 6.32 5.83

36678000.000 3.18735 376780000.000 6.07 3.31 3.06 6.32 5.83

36685000.000 3.18738 376850000.000 6.07 3.31 3.06 6.32 5.83

36692000.000 3.18741 376920000.000 6.07 3.31 3.06 6.32 5.83

36699000.000 3.18744 376990000.000 6.07 3.31 3.06 6.32 5.83

36706000.000 3.18747 377060000.000 6.07 3.31 3.06 6.32 5.83

36713000.000 3.18750 377130000.000 6.07 3.32 3.06 6.32 5.83

36720000.000 3.18753 377200000.000 6.07 3.32 3.06 6.32 5.83

36727000.000 3.18756 377270000.000 6.07 3.32 3.06 6.32 5.83

36734000.000 3.18759 377340000.000 6.07 3.32 3.06 6.32 5.83

36741000.000 3.18762 377410000.000 6.07 3.32 3.06 6.32 5.83

36748000.000 3.18765 377480000.000 6.07 3.32 3.06 6.32 5.83

36755000.000 3.18768 377550000.000 6.07 3.32 3.06 6.32 5.83

36762000.000 3.18771 377620000.000 6.07 3.32 3.06 6.32 5.83

36769000.000 3.18775 377690000.000 6.07 3.32 3.06 6.32 5.83

36776000.000 3.18778 377760000.000 6.07 3.32 3.06 6.32 5.83

36783000.000 3.18781 377830000.000 6.07 3.32 3.06 6.32 5.83

36790000.000 3.18784 377900000.000 6.07 3.32 3.06 6.32 5.83

36797000.000 3.18787 377970000.000 6.07 3.32 3.06 6.32 5.83

36804000.000 3.18790 378040000.000 6.07 3.32 3.06 6.32 5.83

36811000.000 3.18793 378110000.000 6.07 3.32 3.06 6.32 5.83

36818000.000 3.18796 378180000.000 6.07 3.32 3.06 6.32 5.83

36825000.000 3.18799 378250000.000 6.07 3.32 3.06 6.32 5.83

36832000.000 3.18802 378320000.000 6.07 3.32 3.06 6.32 5.83

36839000.000 3.18805 378390000.000 6.07 3.32 3.06 6.32 5.83

36846000.000 3.18808 378460000.000 6.07 3.32 3.06 6.32 5.83

36853000.000 3.18811 378530000.000 6.07 3.32 3.06 6.32 5.83

36860000.000 3.18814 378600000.000 6.07 3.32 3.06 6.32 5.83

36867000.000 3.18817 378670000.000 6.07 3.32 3.06 6.32 5.83

36874000.000 3.18820 378740000.000 6.07 3.32 3.06 6.32 5.83

36881000.000 3.18823 378810000.000 6.07 3.32 3.06 6.32 5.83

36888000.000 3.18826 378880000.000 6.07 3.32 3.06 6.32 5.83

36895000.000 3.18829 378950000.000 6.08 3.32 3.06 6.32 5.83

36902000.000 3.18832 379020000.000 6.08 3.32 3.06 6.32 5.83

36909000.000 3.18835 379090000.000 6.08 3.32 3.06 6.32 5.83

36916000.000 3.18838 379160000.000 6.08 3.32 3.06 6.32 5.83

36923000.000 3.18841 379230000.000 6.08 3.32 3.06 6.32 5.83

36930000.000 3.18844 379300000.000 6.08 3.32 3.06 6.32 5.83

36937000.000 3.18847 379370000.000 6.08 3.32 3.06 6.32 5.83

36944000.000 3.18850 379440000.000 6.08 3.32 3.06 6.32 5.83

36951000.000 3.18853 379510000.000 6.08 3.32 3.06 6.32 5.83

36958000.000 3.18857 379580000.000 6.08 3.32 3.06 6.32 5.83

36965000.000 3.18860 379650000.000 6.08 3.32 3.06 6.32 5.83

36972000.000 3.18863 379720000.000 6.08 3.32 3.06 6.32 5.83

36979000.000 3.18866 379790000.000 6.08 3.32 3.06 6.32 5.83

36986000.000 3.18869 379860000.000 6.08 3.32 3.06 6.32 5.83

36993000.000 3.18872 379930000.000 6.08 3.32 3.06 6.32 5.83

37000000.000 3.18875 380000000.000 6.08 3.32 3.06 6.32 5.83

37007000.000 3.18878 380070000.000 6.08 3.32 3.06 6.32 5.83

37014000.000 3.18881 380140000.000 6.08 3.32 3.06 6.32 5.83

37021000.000 3.18884 380210000.000 6.08 3.32 3.06 6.32 5.83

37028000.000 3.18887 380280000.000 6.08 3.32 3.06 6.32 5.83

37035000.000 3.18890 380350000.000 6.08 3.32 3.06 6.32 5.83

37042000.000 3.18893 380420000.000 6.08 3.32 3.06 6.32 5.83

37049000.000 3.18896 380490000.000 6.08 3.32 3.06 6.32 5.83

37056000.000 3.18899 380560000.000 6.08 3.32 3.06 6.32 5.83

37063000.000 3.18902 380630000.000 6.08 3.32 3.06 6.32 5.83

37070000.000 3.18905 380700000.000 6.08 3.32 3.06 6.32 5.83

37077000.000 3.18908 380770000.000 6.08 3.32 3.06 6.32 5.83

37084000.000 3.18911 380840000.000 6.08 3.32 3.06 6.32 5.83

37091000.000 3.18914 380910000.000 6.08 3.32 3.06 6.32 5.83

37098000.000 3.18917 380980000.000 6.08 3.32 3.06 6.32 5.83

37105000.000 3.18920 381050000.000 6.08 3.32 3.06 6.32 5.83

37112000.000 3.18923 381120000.000 6.08 3.32 3.06 6.32 5.83

37119000.000 3.18926 381190000.000 6.08 3.32 3.06 6.32 5.83

37126000.000 3.18929 381260000.000 6.08 3.32 3.06 6.32 5.83

37133000.000 3.18932 381330000.000 6.08 3.32 3.06 6.32 5.83

37140000.000 3.18935 381400000.000 6.08 3.32 3.06 6.32 5.83

37147000.000 3.18938 381470000.000 6.08 3.32 3.06 6.32 5.83

37154000.000 3.18941 381540000.000 6.08 3.32 3.06 6.32 5.83

37161000.000 3.18944 381610000.000 6.08 3.32 3.06 6.32 5.83

37168000.000 3.18947 381680000.000 6.08 3.32 3.06 6.32 5.83

37175000.000 3.18950 381750000.000 6.08 3.32 3.06 6.32 5.83

37182000.000 3.18953 381820000.000 6.08 3.32 3.06 6.32 5.83

37189000.000 3.18956 381890000.000 6.08 3.32 3.06 6.32 5.83

37196000.000 3.18959 381960000.000 6.08 3.32 3.06 6.32 5.83

37203000.000 3.18962 382030000.000 6.08 3.32 3.06 6.32 5.83

37210000.000 3.18965 382100000.000 6.08 3.32 3.06 6.32 5.83

37217000.000 3.18968 382170000.000 6.08 3.32 3.06 6.32 5.83

37224000.000 3.18971 382240000.000 6.08 3.32 3.06 6.32 5.83

37231000.000 3.18974 382310000.000 6.08 3.32 3.06 6.32 5.83

37238000.000 3.18977 382380000.000 6.08 3.32 3.06 6.32 5.83

37245000.000 3.18980 382450000.000 6.08 3.32 3.06 6.32 5.83

37252000.000 3.18983 382520000.000 6.08 3.32 3.06 6.32 5.83

37259000.000 3.18986 382590000.000 6.08 3.32 3.06 6.32 5.83

37266000.000 3.18989 382660000.000 6.08 3.32 3.06 6.32 5.83

37273000.000 3.18992 382730000.000 6.08 3.32 3.06 6.32 5.83

37280000.000 3.18995 382800000.000 6.08 3.32 3.06 6.32 5.84

37287000.000 3.18998 382870000.000 6.08 3.32 3.06 6.32 5.84

37294000.000 3.19001 382940000.000 6.08 3.32 3.06 6.32 5.84

37301000.000 3.19004 383010000.000 6.08 3.32 3.06 6.32 5.84

37308000.000 3.19007 383080000.000 6.08 3.32 3.06 6.32 5.84

37315000.000 3.19010 383150000.000 6.08 3.32 3.06 6.32 5.84

37322000.000 3.19013 383220000.000 6.08 3.32 3.06 6.32 5.84

37329000.000 3.19016 383290000.000 6.08 3.32 3.06 6.32 5.84

37336000.000 3.19019 383360000.000 6.08 3.32 3.06 6.32 5.84

37343000.000 3.19022 383430000.000 6.08 3.32 3.06 6.32 5.84

37350000.000 3.19025 383500000.000 6.08 3.32 3.06 6.32 5.84

37357000.000 3.19028 383570000.000 6.08 3.32 3.06 6.32 5.84

37364000.000 3.19031 383640000.000 6.08 3.32 3.06 6.32 5.84

37371000.000 3.19034 383710000.000 6.08 3.32 3.06 6.32 5.84

37378000.000 3.19037 383780000.000 6.08 3.32 3.06 6.32 5.84

37385000.000 3.19040 383850000.000 6.08 3.32 3.06 6.32 5.84

37392000.000 3.19043 383920000.000 6.08 3.32 3.06 6.32 5.84

37399000.000 3.19046 383990000.000 6.08 3.32 3.06 6.32 5.84

37406000.000 3.19049 384060000.000 6.08 3.32 3.06 6.32 5.84

37413000.000 3.19052 384130000.000 6.08 3.32 3.06 6.32 5.84

37420000.000 3.19055 384200000.000 6.08 3.32 3.06 6.32 5.84

37427000.000 3.19058 384270000.000 6.08 3.32 3.06 6.32 5.84

37434000.000 3.19061 384340000.000 6.08 3.32 3.06 6.32 5.84

37441000.000 3.19064 384410000.000 6.08 3.32 3.06 6.32 5.84

37448000.000 3.19067 384480000.000 6.08 3.32 3.06 6.32 5.84

37455000.000 3.19070 384550000.000 6.08 3.32 3.06 6.32 5.84

37462000.000 3.19073 384620000.000 6.08 3.32 3.06 6.32 5.84

37469000.000 3.19076 384690000.000 6.08 3.32 3.06 6.32 5.84

37476000.000 3.19079 384760000.000 6.08 3.32 3.06 6.32 5.84

37483000.000 3.19082 384830000.000 6.08 3.32 3.06 6.32 5.84

37490000.000 3.19085 384900000.000 6.08 3.32 3.06 6.32 5.84

37497000.000 3.19088 384970000.000 6.08 3.32 3.06 6.32 5.84

37504000.000 3.19091 385040000.000 6.08 3.32 3.06 6.32 5.84

37511000.000 3.19094 385110000.000 6.08 3.32 3.06 6.32 5.84

37518000.000 3.19097 385180000.000 6.08 3.32 3.06 6.32 5.84

37525000.000 3.19100 385250000.000 6.08 3.32 3.06 6.32 5.84

37532000.000 3.19103 385320000.000 6.08 3.32 3.06 6.32 5.84

37539000.000 3.19106 385390000.000 6.08 3.32 3.06 6.32 5.84

37546000.000 3.19109 385460000.000 6.08 3.32 3.06 6.32 5.84

37553000.000 3.19112 385530000.000 6.08 3.32 3.06 6.32 5.84

37560000.000 3.19115 385600000.000 6.08 3.32 3.06 6.32 5.84

37567000.000 3.19118 385670000.000 6.08 3.32 3.06 6.32 5.84

37574000.000 3.19121 385740000.000 6.08 3.32 3.06 6.32 5.84

37581000.000 3.19124 385810000.000 6.08 3.32 3.06 6.32 5.84

37588000.000 3.19127 385880000.000 6.08 3.32 3.06 6.32 5.84

37595000.000 3.19130 385950000.000 6.08 3.32 3.06 6.32 5.84

37602000.000 3.19133 386020000.000 6.08 3.32 3.06 6.32 5.84

37609000.000 3.19136 386090000.000 6.08 3.32 3.06 6.32 5.84

37616000.000 3.19139 386160000.000 6.08 3.32 3.06 6.32 5.84

37623000.000 3.19142 386230000.000 6.08 3.32 3.06 6.32 5.84

37630000.000 3.19145 386300000.000 6.08 3.32 3.06 6.32 5.84

37637000.000 3.19148 386370000.000 6.08 3.32 3.06 6.32 5.84

37644000.000 3.19151 386440000.000 6.08 3.32 3.06 6.32 5.84

37651000.000 3.19154 386510000.000 6.08 3.32 3.06 6.32 5.84

37658000.000 3.19157 386580000.000 6.08 3.32 3.06 6.32 5.84

37665000.000 3.19160 386650000.000 6.08 3.32 3.06 6.32 5.84

37672000.000 3.19163 386720000.000 6.08 3.32 3.06 6.32 5.84

37679000.000 3.19166 386790000.000 6.08 3.32 3.06 6.32 5.84

37686000.000 3.19169 386860000.000 6.08 3.32 3.06 6.32 5.84

37693000.000 3.19172 386930000.000 6.08 3.32 3.06 6.32 5.84

37700000.000 3.19175 387000000.000 6.08 3.32 3.06 6.32 5.84

37707000.000 3.19178 387070000.000 6.08 3.32 3.06 6.32 5.84

37714000.000 3.19181 387140000.000 6.08 3.32 3.06 6.32 5.84

37721000.000 3.19184 387210000.000 6.08 3.32 3.06 6.32 5.84

37728000.000 3.19186 387280000.000 6.08 3.32 3.06 6.32 5.84

37735000.000 3.19189 387350000.000 6.08 3.32 3.06 6.32 5.84

37742000.000 3.19192 387420000.000 6.08 3.32 3.06 6.33 5.84

37749000.000 3.19195 387490000.000 6.08 3.32 3.06 6.33 5.84

37756000.000 3.19198 387560000.000 6.08 3.32 3.06 6.33 5.84

37763000.000 3.19201 387630000.000 6.08 3.32 3.06 6.33 5.84

37770000.000 3.19204 387700000.000 6.08 3.32 3.06 6.33 5.84

37777000.000 3.19207 387770000.000 6.08 3.32 3.06 6.33 5.84

37784000.000 3.19210 387840000.000 6.08 3.32 3.06 6.33 5.84

37791000.000 3.19213 387910000.000 6.08 3.32 3.06 6.33 5.84

37798000.000 3.19216 387980000.000 6.08 3.32 3.06 6.33 5.84

37805000.000 3.19219 388050000.000 6.08 3.32 3.06 6.33 5.84

37812000.000 3.19222 388120000.000 6.08 3.32 3.06 6.33 5.84

37819000.000 3.19225 388190000.000 6.08 3.32 3.06 6.33 5.84

37826000.000 3.19228 388260000.000 6.08 3.32 3.06 6.33 5.84

37833000.000 3.19231 388330000.000 6.08 3.32 3.06 6.33 5.84

37840000.000 3.19234 388400000.000 6.08 3.32 3.06 6.33 5.84

37847000.000 3.19237 388470000.000 6.08 3.32 3.06 6.33 5.84

37854000.000 3.19240 388540000.000 6.08 3.32 3.06 6.33 5.84

37861000.000 3.19243 388610000.000 6.08 3.32 3.06 6.33 5.84

37868000.000 3.19246 388680000.000 6.08 3.32 3.06 6.33 5.84

37875000.000 3.19249 388750000.000 6.08 3.32 3.06 6.33 5.84

37882000.000 3.19252 388820000.000 6.08 3.32 3.06 6.33 5.84

37889000.000 3.19255 388890000.000 6.08 3.32 3.06 6.33 5.84

37896000.000 3.19258 388960000.000 6.08 3.32 3.06 6.33 5.84

37903000.000 3.19261 389030000.000 6.08 3.32 3.06 6.33 5.84

37910000.000 3.19264 389100000.000 6.08 3.32 3.06 6.33 5.84

37917000.000 3.19267 389170000.000 6.08 3.32 3.06 6.33 5.84

37924000.000 3.19269 389240000.000 6.08 3.32 3.06 6.33 5.84

37931000.000 3.19272 389310000.000 6.08 3.32 3.07 6.33 5.84

37938000.000 3.19275 389380000.000 6.08 3.32 3.07 6.33 5.84

37945000.000 3.19278 389450000.000 6.08 3.32 3.07 6.33 5.84

37952000.000 3.19281 389520000.000 6.08 3.32 3.07 6.33 5.84

37959000.000 3.19284 389590000.000 6.08 3.32 3.07 6.33 5.84

37966000.000 3.19287 389660000.000 6.08 3.32 3.07 6.33 5.84

37973000.000 3.19290 389730000.000 6.08 3.32 3.07 6.33 5.84

37980000.000 3.19293 389800000.000 6.08 3.32 3.07 6.33 5.84

37987000.000 3.19296 389870000.000 6.08 3.32 3.07 6.33 5.84

37994000.000 3.19299 389940000.000 6.08 3.32 3.07 6.33 5.84

38001000.000 3.19302 390010000.000 6.08 3.32 3.07 6.33 5.84

38008000.000 3.19305 390080000.000 6.08 3.32 3.07 6.33 5.84

38015000.000 3.19308 390150000.000 6.08 3.32 3.07 6.33 5.84

38022000.000 3.19311 390220000.000 6.08 3.32 3.07 6.33 5.84

38029000.000 3.19314 390290000.000 6.08 3.32 3.07 6.33 5.84

38036000.000 3.19317 390360000.000 6.08 3.32 3.07 6.33 5.84

38043000.000 3.19320 390430000.000 6.08 3.32 3.07 6.33 5.84

38050000.000 3.19323 390500000.000 6.08 3.32 3.07 6.33 5.84

38057000.000 3.19326 390570000.000 6.08 3.32 3.07 6.33 5.84

38064000.000 3.19328 390640000.000 6.08 3.32 3.07 6.33 5.84

38071000.000 3.19331 390710000.000 6.08 3.32 3.07 6.33 5.84

38078000.000 3.19334 390780000.000 6.08 3.32 3.07 6.33 5.84

38085000.000 3.19337 390850000.000 6.08 3.32 3.07 6.33 5.84

38092000.000 3.19340 390920000.000 6.08 3.32 3.07 6.33 5.84

38099000.000 3.19343 390990000.000 6.08 3.32 3.07 6.33 5.84

38106000.000 3.19346 391060000.000 6.08 3.32 3.07 6.33 5.84

38113000.000 3.19349 391130000.000 6.08 3.32 3.07 6.33 5.84

38120000.000 3.19352 391200000.000 6.08 3.32 3.07 6.33 5.84

38127000.000 3.19355 391270000.000 6.08 3.32 3.07 6.33 5.84

38134000.000 3.19358 391340000.000 6.08 3.32 3.07 6.33 5.84

38141000.000 3.19361 391410000.000 6.08 3.32 3.07 6.33 5.84

38148000.000 3.19364 391480000.000 6.08 3.32 3.07 6.33 5.84

38155000.000 3.19367 391550000.000 6.09 3.32 3.07 6.33 5.84

38162000.000 3.19370 391620000.000 6.09 3.32 3.07 6.33 5.84

38169000.000 3.19373 391690000.000 6.09 3.32 3.07 6.33 5.84

38176000.000 3.19376 391760000.000 6.09 3.32 3.07 6.33 5.84

38183000.000 3.19378 391830000.000 6.09 3.32 3.07 6.33 5.84

38190000.000 3.19381 391900000.000 6.09 3.32 3.07 6.33 5.84

38197000.000 3.19384 391970000.000 6.09 3.32 3.07 6.33 5.84

38204000.000 3.19387 392040000.000 6.09 3.32 3.07 6.33 5.84

38211000.000 3.19390 392110000.000 6.09 3.32 3.07 6.33 5.84

38218000.000 3.19393 392180000.000 6.09 3.32 3.07 6.33 5.84

38225000.000 3.19396 392250000.000 6.09 3.32 3.07 6.33 5.84

38232000.000 3.19399 392320000.000 6.09 3.32 3.07 6.33 5.84

38239000.000 3.19402 392390000.000 6.09 3.32 3.07 6.33 5.84

38246000.000 3.19405 392460000.000 6.09 3.32 3.07 6.33 5.84

38253000.000 3.19408 392530000.000 6.09 3.32 3.07 6.33 5.84

38260000.000 3.19411 392600000.000 6.09 3.32 3.07 6.33 5.84

38267000.000 3.19414 392670000.000 6.09 3.32 3.07 6.33 5.84

38274000.000 3.19417 392740000.000 6.09 3.32 3.07 6.33 5.84

38281000.000 3.19420 392810000.000 6.09 3.32 3.07 6.33 5.84

38288000.000 3.19422 392880000.000 6.09 3.32 3.07 6.33 5.84

38295000.000 3.19425 392950000.000 6.09 3.32 3.07 6.33 5.84

38302000.000 3.19428 393020000.000 6.09 3.32 3.07 6.33 5.84

38309000.000 3.19431 393090000.000 6.09 3.32 3.07 6.33 5.84

38316000.000 3.19434 393160000.000 6.09 3.32 3.07 6.33 5.84

38323000.000 3.19437 393230000.000 6.09 3.32 3.07 6.33 5.84

38330000.000 3.19440 393300000.000 6.09 3.32 3.07 6.33 5.84

38337000.000 3.19443 393370000.000 6.09 3.32 3.07 6.33 5.84

38344000.000 3.19446 393440000.000 6.09 3.32 3.07 6.33 5.84

38351000.000 3.19449 393510000.000 6.09 3.32 3.07 6.33 5.84

38358000.000 3.19452 393580000.000 6.09 3.32 3.07 6.33 5.84

38365000.000 3.19455 393650000.000 6.09 3.32 3.07 6.33 5.84

38372000.000 3.19458 393720000.000 6.09 3.32 3.07 6.33 5.84

38379000.000 3.19461 393790000.000 6.09 3.32 3.07 6.33 5.84

38386000.000 3.19463 393860000.000 6.09 3.32 3.07 6.33 5.84

38393000.000 3.19466 393930000.000 6.09 3.32 3.07 6.33 5.84

38400000.000 3.19469 394000000.000 6.09 3.32 3.07 6.33 5.84

38407000.000 3.19472 394070000.000 6.09 3.32 3.07 6.33 5.84

38414000.000 3.19475 394140000.000 6.09 3.32 3.07 6.33 5.84

38421000.000 3.19478 394210000.000 6.09 3.32 3.07 6.33 5.84

38428000.000 3.19481 394280000.000 6.09 3.32 3.07 6.33 5.84

38435000.000 3.19484 394350000.000 6.09 3.32 3.07 6.33 5.84

38442000.000 3.19487 394420000.000 6.09 3.32 3.07 6.33 5.84

38449000.000 3.19490 394490000.000 6.09 3.32 3.07 6.33 5.84

38456000.000 3.19493 394560000.000 6.09 3.32 3.07 6.33 5.84

38463000.000 3.19496 394630000.000 6.09 3.32 3.07 6.33 5.84

38470000.000 3.19498 394700000.000 6.09 3.32 3.07 6.33 5.84

38477000.000 3.19501 394770000.000 6.09 3.32 3.07 6.33 5.84

38484000.000 3.19504 394840000.000 6.09 3.32 3.07 6.33 5.84

38491000.000 3.19507 394910000.000 6.09 3.32 3.07 6.33 5.84

38498000.000 3.19510 394980000.000 6.09 3.32 3.07 6.33 5.84

38505000.000 3.19513 395050000.000 6.09 3.32 3.07 6.33 5.84

38512000.000 3.19516 395120000.000 6.09 3.32 3.07 6.33 5.84

38519000.000 3.19519 395190000.000 6.09 3.32 3.07 6.33 5.84

38526000.000 3.19522 395260000.000 6.09 3.32 3.07 6.33 5.84

38533000.000 3.19525 395330000.000 6.09 3.32 3.07 6.33 5.84

38540000.000 3.19528 395400000.000 6.09 3.32 3.07 6.33 5.84

38547000.000 3.19531 395470000.000 6.09 3.32 3.07 6.33 5.84

38554000.000 3.19533 395540000.000 6.09 3.32 3.07 6.33 5.84

38561000.000 3.19536 395610000.000 6.09 3.32 3.07 6.33 5.84

38568000.000 3.19539 395680000.000 6.09 3.32 3.07 6.33 5.84

38575000.000 3.19542 395750000.000 6.09 3.32 3.07 6.33 5.84

38582000.000 3.19545 395820000.000 6.09 3.32 3.07 6.33 5.84

38589000.000 3.19548 395890000.000 6.09 3.32 3.07 6.33 5.84

38596000.000 3.19551 395960000.000 6.09 3.32 3.07 6.33 5.84

38603000.000 3.19554 396030000.000 6.09 3.32 3.07 6.33 5.84

38610000.000 3.19557 396100000.000 6.09 3.32 3.07 6.33 5.85

38617000.000 3.19560 396170000.000 6.09 3.32 3.07 6.33 5.85

38624000.000 3.19562 396240000.000 6.09 3.32 3.07 6.33 5.85

38631000.000 3.19565 396310000.000 6.09 3.32 3.07 6.33 5.85

38638000.000 3.19568 396380000.000 6.09 3.32 3.07 6.33 5.85

38645000.000 3.19571 396450000.000 6.09 3.32 3.07 6.33 5.85

38652000.000 3.19574 396520000.000 6.09 3.32 3.07 6.33 5.85

38659000.000 3.19577 396590000.000 6.09 3.32 3.07 6.33 5.85

38666000.000 3.19580 396660000.000 6.09 3.32 3.07 6.33 5.85

38673000.000 3.19583 396730000.000 6.09 3.32 3.07 6.33 5.85

38680000.000 3.19586 396800000.000 6.09 3.32 3.07 6.33 5.85

38687000.000 3.19589 396870000.000 6.09 3.32 3.07 6.33 5.85

38694000.000 3.19592 396940000.000 6.09 3.32 3.07 6.33 5.85

38701000.000 3.19594 397010000.000 6.09 3.32 3.07 6.33 5.85

38708000.000 3.19597 397080000.000 6.09 3.32 3.07 6.33 5.85

38715000.000 3.19600 397150000.000 6.09 3.32 3.07 6.33 5.85

38722000.000 3.19603 397220000.000 6.09 3.32 3.07 6.33 5.85

38729000.000 3.19606 397290000.000 6.09 3.32 3.07 6.33 5.85

38736000.000 3.19609 397360000.000 6.09 3.32 3.07 6.33 5.85

38743000.000 3.19612 397430000.000 6.09 3.32 3.07 6.33 5.85

38750000.000 3.19615 397500000.000 6.09 3.32 3.07 6.33 5.85

38757000.000 3.19618 397570000.000 6.09 3.32 3.07 6.33 5.85

38764000.000 3.19620 397640000.000 6.09 3.32 3.07 6.33 5.85

38771000.000 3.19623 397710000.000 6.09 3.32 3.07 6.33 5.85

38778000.000 3.19626 397780000.000 6.09 3.32 3.07 6.33 5.85

38785000.000 3.19629 397850000.000 6.09 3.32 3.07 6.33 5.85

38792000.000 3.19632 397920000.000 6.09 3.32 3.07 6.33 5.85

38799000.000 3.19635 397990000.000 6.09 3.32 3.07 6.33 5.85

38806000.000 3.19638 398060000.000 6.09 3.32 3.07 6.33 5.85

38813000.000 3.19641 398130000.000 6.09 3.32 3.07 6.33 5.85

38820000.000 3.19644 398200000.000 6.09 3.32 3.07 6.33 5.85

38827000.000 3.19647 398270000.000 6.09 3.32 3.07 6.33 5.85

38834000.000 3.19649 398340000.000 6.09 3.32 3.07 6.33 5.85

38841000.000 3.19652 398410000.000 6.09 3.32 3.07 6.33 5.85

38848000.000 3.19655 398480000.000 6.09 3.32 3.07 6.33 5.85

38855000.000 3.19658 398550000.000 6.09 3.32 3.07 6.33 5.85

38862000.000 3.19661 398620000.000 6.09 3.32 3.07 6.33 5.85

38869000.000 3.19664 398690000.000 6.09 3.32 3.07 6.33 5.85

38876000.000 3.19667 398760000.000 6.09 3.32 3.07 6.33 5.85

38883000.000 3.19670 398830000.000 6.09 3.32 3.07 6.33 5.85

38890000.000 3.19673 398900000.000 6.09 3.32 3.07 6.33 5.85

38897000.000 3.19675 398970000.000 6.09 3.32 3.07 6.33 5.85

38904000.000 3.19678 399040000.000 6.09 3.32 3.07 6.33 5.85

38911000.000 3.19681 399110000.000 6.09 3.32 3.07 6.33 5.85

38918000.000 3.19684 399180000.000 6.09 3.32 3.07 6.33 5.85

38925000.000 3.19687 399250000.000 6.09 3.32 3.07 6.33 5.85

38932000.000 3.19690 399320000.000 6.09 3.32 3.07 6.33 5.85

38939000.000 3.19693 399390000.000 6.09 3.32 3.07 6.33 5.85

38946000.000 3.19696 399460000.000 6.09 3.32 3.07 6.33 5.85

38953000.000 3.19698 399530000.000 6.09 3.32 3.07 6.33 5.85

38960000.000 3.19701 399600000.000 6.09 3.32 3.07 6.33 5.85

38967000.000 3.19704 399670000.000 6.09 3.32 3.07 6.33 5.85

38974000.000 3.19707 399740000.000 6.09 3.32 3.07 6.33 5.85

38981000.000 3.19710 399810000.000 6.09 3.32 3.07 6.34 5.85

38988000.000 3.19713 399880000.000 6.09 3.33 3.07 6.34 5.85

38995000.000 3.19716 399950000.000 6.09 3.33 3.07 6.34 5.85

39002000.000 3.19719 400020000.000 6.09 3.33 3.07 6.34 5.85

39009000.000 3.19722 400090000.000 6.09 3.33 3.07 6.34 5.85

39016000.000 3.19724 400160000.000 6.09 3.33 3.07 6.34 5.85

39023000.000 3.19727 400230000.000 6.09 3.33 3.07 6.34 5.85

39030000.000 3.19730 400300000.000 6.09 3.33 3.07 6.34 5.85

39037000.000 3.19733 400370000.000 6.09 3.33 3.07 6.34 5.85

39044000.000 3.19736 400440000.000 6.09 3.33 3.07 6.34 5.85

39051000.000 3.19739 400510000.000 6.09 3.33 3.07 6.34 5.85

39058000.000 3.19742 400580000.000 6.09 3.33 3.07 6.34 5.85

39065000.000 3.19745 400650000.000 6.09 3.33 3.07 6.34 5.85

39072000.000 3.19747 400720000.000 6.09 3.33 3.07 6.34 5.85

39079000.000 3.19750 400790000.000 6.09 3.33 3.07 6.34 5.85

39086000.000 3.19753 400860000.000 6.09 3.33 3.07 6.34 5.85

39093000.000 3.19756 400930000.000 6.09 3.33 3.07 6.34 5.85

39100000.000 3.19759 401000000.000 6.09 3.33 3.07 6.34 5.85

39107000.000 3.19762 401070000.000 6.09 3.33 3.07 6.34 5.85

39114000.000 3.19765 401140000.000 6.09 3.33 3.07 6.34 5.85

39121000.000 3.19768 401210000.000 6.09 3.33 3.07 6.34 5.85

39128000.000 3.19770 401280000.000 6.09 3.33 3.07 6.34 5.85

39135000.000 3.19773 401350000.000 6.09 3.33 3.07 6.34 5.85

39142000.000 3.19776 401420000.000 6.09 3.33 3.07 6.34 5.85

39149000.000 3.19779 401490000.000 6.09 3.33 3.07 6.34 5.85

39156000.000 3.19782 401560000.000 6.09 3.33 3.07 6.34 5.85

39163000.000 3.19785 401630000.000 6.09 3.33 3.07 6.34 5.85

39170000.000 3.19788 401700000.000 6.09 3.33 3.07 6.34 5.85

39177000.000 3.19790 401770000.000 6.09 3.33 3.07 6.34 5.85

39184000.000 3.19793 401840000.000 6.09 3.33 3.07 6.34 5.85

39191000.000 3.19796 401910000.000 6.09 3.33 3.07 6.34 5.85

39198000.000 3.19799 401980000.000 6.09 3.33 3.07 6.34 5.85

39205000.000 3.19802 402050000.000 6.09 3.33 3.07 6.34 5.85

39212000.000 3.19805 402120000.000 6.09 3.33 3.07 6.34 5.85

39219000.000 3.19808 402190000.000 6.09 3.33 3.07 6.34 5.85

39226000.000 3.19811 402260000.000 6.09 3.33 3.07 6.34 5.85

39233000.000 3.19813 402330000.000 6.09 3.33 3.07 6.34 5.85

39240000.000 3.19816 402400000.000 6.09 3.33 3.07 6.34 5.85

39247000.000 3.19819 402470000.000 6.09 3.33 3.07 6.34 5.85

39254000.000 3.19822 402540000.000 6.09 3.33 3.07 6.34 5.85

39261000.000 3.19825 402610000.000 6.09 3.33 3.07 6.34 5.85

39268000.000 3.19828 402680000.000 6.09 3.33 3.07 6.34 5.85

39275000.000 3.19831 402750000.000 6.09 3.33 3.07 6.34 5.85

39282000.000 3.19833 402820000.000 6.09 3.33 3.07 6.34 5.85

39289000.000 3.19836 402890000.000 6.09 3.33 3.07 6.34 5.85

39296000.000 3.19839 402960000.000 6.09 3.33 3.07 6.34 5.85

39303000.000 3.19842 403030000.000 6.09 3.33 3.07 6.34 5.85

39310000.000 3.19845 403100000.000 6.09 3.33 3.07 6.34 5.85

39317000.000 3.19848 403170000.000 6.09 3.33 3.07 6.34 5.85

39324000.000 3.19851 403240000.000 6.09 3.33 3.07 6.34 5.85

39331000.000 3.19853 403310000.000 6.09 3.33 3.07 6.34 5.85

39338000.000 3.19856 403380000.000 6.09 3.33 3.07 6.34 5.85

39345000.000 3.19859 403450000.000 6.09 3.33 3.07 6.34 5.85

39352000.000 3.19862 403520000.000 6.09 3.33 3.07 6.34 5.85

39359000.000 3.19865 403590000.000 6.09 3.33 3.07 6.34 5.85

39366000.000 3.19868 403660000.000 6.09 3.33 3.07 6.34 5.85

39373000.000 3.19871 403730000.000 6.09 3.33 3.07 6.34 5.85

39380000.000 3.19873 403800000.000 6.09 3.33 3.07 6.34 5.85

39387000.000 3.19876 403870000.000 6.09 3.33 3.07 6.34 5.85

39394000.000 3.19879 403940000.000 6.09 3.33 3.07 6.34 5.85

39401000.000 3.19882 404010000.000 6.09 3.33 3.07 6.34 5.85

39408000.000 3.19885 404080000.000 6.09 3.33 3.07 6.34 5.85

39415000.000 3.19888 404150000.000 6.09 3.33 3.07 6.34 5.85

39422000.000 3.19890 404220000.000 6.09 3.33 3.07 6.34 5.85

39429000.000 3.19893 404290000.000 6.09 3.33 3.07 6.34 5.85

39436000.000 3.19896 404360000.000 6.09 3.33 3.07 6.34 5.85

39443000.000 3.19899 404430000.000 6.09 3.33 3.07 6.34 5.85

39450000.000 3.19902 404500000.000 6.09 3.33 3.07 6.34 5.85

39457000.000 3.19905 404570000.000 6.10 3.33 3.07 6.34 5.85

39464000.000 3.19908 404640000.000 6.10 3.33 3.07 6.34 5.85

39471000.000 3.19910 404710000.000 6.10 3.33 3.07 6.34 5.85

39478000.000 3.19913 404780000.000 6.10 3.33 3.07 6.34 5.85

39485000.000 3.19916 404850000.000 6.10 3.33 3.07 6.34 5.85

39492000.000 3.19919 404920000.000 6.10 3.33 3.07 6.34 5.85

39499000.000 3.19922 404990000.000 6.10 3.33 3.07 6.34 5.85

39506000.000 3.19925 405060000.000 6.10 3.33 3.07 6.34 5.85

39513000.000 3.19927 405130000.000 6.10 3.33 3.07 6.34 5.85

39520000.000 3.19930 405200000.000 6.10 3.33 3.07 6.34 5.85

39527000.000 3.19933 405270000.000 6.10 3.33 3.07 6.34 5.85

39534000.000 3.19936 405340000.000 6.10 3.33 3.07 6.34 5.85

39541000.000 3.19939 405410000.000 6.10 3.33 3.07 6.34 5.85

39548000.000 3.19942 405480000.000 6.10 3.33 3.07 6.34 5.85

39555000.000 3.19945 405550000.000 6.10 3.33 3.07 6.34 5.85

39562000.000 3.19947 405620000.000 6.10 3.33 3.07 6.34 5.85

39569000.000 3.19950 405690000.000 6.10 3.33 3.07 6.34 5.85

39576000.000 3.19953 405760000.000 6.10 3.33 3.07 6.34 5.85

39583000.000 3.19956 405830000.000 6.10 3.33 3.07 6.34 5.85

39590000.000 3.19959 405900000.000 6.10 3.33 3.07 6.34 5.85

39597000.000 3.19962 405970000.000 6.10 3.33 3.07 6.34 5.85

39604000.000 3.19964 406040000.000 6.10 3.33 3.07 6.34 5.85

39611000.000 3.19967 406110000.000 6.10 3.33 3.07 6.34 5.85

39618000.000 3.19970 406180000.000 6.10 3.33 3.07 6.34 5.85

39625000.000 3.19973 406250000.000 6.10 3.33 3.07 6.34 5.85

39632000.000 3.19976 406320000.000 6.10 3.33 3.07 6.34 5.85

39639000.000 3.19979 406390000.000 6.10 3.33 3.07 6.34 5.85

39646000.000 3.19981 406460000.000 6.10 3.33 3.07 6.34 5.85

39653000.000 3.19984 406530000.000 6.10 3.33 3.07 6.34 5.85

39660000.000 3.19987 406600000.000 6.10 3.33 3.07 6.34 5.85

39667000.000 3.19990 406670000.000 6.10 3.33 3.07 6.34 5.85

39674000.000 3.19993 406740000.000 6.10 3.33 3.07 6.34 5.85

39681000.000 3.19996 406810000.000 6.10 3.33 3.07 6.34 5.85

39688000.000 3.19998 406880000.000 6.10 3.33 3.07 6.34 5.85

39695000.000 3.20001 406950000.000 6.10 3.33 3.07 6.34 5.85

39702000.000 3.20004 407020000.000 6.10 3.33 3.07 6.34 5.85

39709000.000 3.20007 407090000.000 6.10 3.33 3.07 6.34 5.85

39716000.000 3.20010 407160000.000 6.10 3.33 3.07 6.34 5.85

39723000.000 3.20013 407230000.000 6.10 3.33 3.07 6.34 5.85

39730000.000 3.20015 407300000.000 6.10 3.33 3.07 6.34 5.85

39737000.000 3.20018 407370000.000 6.10 3.33 3.07 6.34 5.85

39744000.000 3.20021 407440000.000 6.10 3.33 3.07 6.34 5.85

39751000.000 3.20024 407510000.000 6.10 3.33 3.07 6.34 5.85

39758000.000 3.20027 407580000.000 6.10 3.33 3.07 6.34 5.85

39765000.000 3.20030 407650000.000 6.10 3.33 3.07 6.34 5.85

39772000.000 3.20032 407720000.000 6.10 3.33 3.07 6.34 5.85

39779000.000 3.20035 407790000.000 6.10 3.33 3.07 6.34 5.85

39786000.000 3.20038 407860000.000 6.10 3.33 3.07 6.34 5.85

39793000.000 3.20041 407930000.000 6.10 3.33 3.07 6.34 5.85

39800000.000 3.20044 408000000.000 6.10 3.33 3.07 6.34 5.85

39807000.000 3.20046 408070000.000 6.10 3.33 3.07 6.34 5.85

39814000.000 3.20049 408140000.000 6.10 3.33 3.07 6.34 5.85

39821000.000 3.20052 408210000.000 6.10 3.33 3.07 6.34 5.85

39828000.000 3.20055 408280000.000 6.10 3.33 3.07 6.34 5.85

39835000.000 3.20058 408350000.000 6.10 3.33 3.07 6.34 5.85

39842000.000 3.20061 408420000.000 6.10 3.33 3.07 6.34 5.85

39849000.000 3.20063 408490000.000 6.10 3.33 3.07 6.34 5.85

39856000.000 3.20066 408560000.000 6.10 3.33 3.07 6.34 5.85

39863000.000 3.20069 408630000.000 6.10 3.33 3.07 6.34 5.85

39870000.000 3.20072 408700000.000 6.10 3.33 3.07 6.34 5.85

39877000.000 3.20075 408770000.000 6.10 3.33 3.07 6.34 5.85

39884000.000 3.20077 408840000.000 6.10 3.33 3.07 6.34 5.85

39891000.000 3.20080 408910000.000 6.10 3.33 3.07 6.34 5.85

39898000.000 3.20083 408980000.000 6.10 3.33 3.07 6.34 5.85

39905000.000 3.20086 409050000.000 6.10 3.33 3.07 6.34 5.85

39912000.000 3.20089 409120000.000 6.10 3.33 3.07 6.34 5.85

39919000.000 3.20092 409190000.000 6.10 3.33 3.07 6.34 5.85

39926000.000 3.20094 409260000.000 6.10 3.33 3.07 6.34 5.85

39933000.000 3.20097 409330000.000 6.10 3.33 3.07 6.34 5.85

39940000.000 3.20100 409400000.000 6.10 3.33 3.07 6.34 5.85

39947000.000 3.20103 409470000.000 6.10 3.33 3.07 6.34 5.85

39954000.000 3.20106 409540000.000 6.10 3.33 3.07 6.34 5.85

39961000.000 3.20108 409610000.000 6.10 3.33 3.07 6.34 5.85

39968000.000 3.20111 409680000.000 6.10 3.33 3.07 6.34 5.85

39975000.000 3.20114 409750000.000 6.10 3.33 3.07 6.34 5.85

39982000.000 3.20117 409820000.000 6.10 3.33 3.07 6.34 5.85

39989000.000 3.20120 409890000.000 6.10 3.33 3.07 6.34 5.86

39996000.000 3.20122 409960000.000 6.10 3.33 3.07 6.34 5.86

40003000.000 3.20125 410030000.000 6.10 3.33 3.07 6.34 5.86

40010000.000 3.20128 410100000.000 6.10 3.33 3.07 6.34 5.86

40017000.000 3.20131 410170000.000 6.10 3.33 3.07 6.34 5.86

40024000.000 3.20134 410240000.000 6.10 3.33 3.07 6.34 5.86

40031000.000 3.20137 410310000.000 6.10 3.33 3.07 6.34 5.86

40038000.000 3.20139 410380000.000 6.10 3.33 3.07 6.34 5.86

40045000.000 3.20142 410450000.000 6.10 3.33 3.07 6.34 5.86

40052000.000 3.20145 410520000.000 6.10 3.33 3.07 6.34 5.86

40059000.000 3.20148 410590000.000 6.10 3.33 3.07 6.34 5.86

40066000.000 3.20151 410660000.000 6.10 3.33 3.07 6.34 5.86

40073000.000 3.20153 410730000.000 6.10 3.33 3.07 6.34 5.86

40080000.000 3.20156 410800000.000 6.10 3.33 3.07 6.34 5.86

40087000.000 3.20159 410870000.000 6.10 3.33 3.07 6.34 5.86

40094000.000 3.20162 410940000.000 6.10 3.33 3.07 6.34 5.86

40101000.000 3.20165 411010000.000 6.10 3.33 3.07 6.34 5.86

40108000.000 3.20167 411080000.000 6.10 3.33 3.07 6.34 5.86

40115000.000 3.20170 411150000.000 6.10 3.33 3.07 6.34 5.86

40122000.000 3.20173 411220000.000 6.10 3.33 3.07 6.34 5.86

40129000.000 3.20176 411290000.000 6.10 3.33 3.07 6.34 5.86

40136000.000 3.20179 411360000.000 6.10 3.33 3.07 6.34 5.86

40143000.000 3.20181 411430000.000 6.10 3.33 3.07 6.34 5.86

40150000.000 3.20184 411500000.000 6.10 3.33 3.07 6.34 5.86

40157000.000 3.20187 411570000.000 6.10 3.33 3.07 6.34 5.86

40164000.000 3.20190 411640000.000 6.10 3.33 3.07 6.34 5.86

40171000.000 3.20193 411710000.000 6.10 3.33 3.07 6.34 5.86

40178000.000 3.20195 411780000.000 6.10 3.33 3.07 6.34 5.86

40185000.000 3.20198 411850000.000 6.10 3.33 3.07 6.34 5.86

40192000.000 3.20201 411920000.000 6.10 3.33 3.07 6.34 5.86

40199000.000 3.20204 411990000.000 6.10 3.33 3.07 6.34 5.86

40206000.000 3.20207 412060000.000 6.10 3.33 3.07 6.34 5.86

40213000.000 3.20209 412130000.000 6.10 3.33 3.07 6.34 5.86

40220000.000 3.20212 412200000.000 6.10 3.33 3.07 6.34 5.86

40227000.000 3.20215 412270000.000 6.10 3.33 3.07 6.34 5.86

40234000.000 3.20218 412340000.000 6.10 3.33 3.07 6.34 5.86

40241000.000 3.20221 412410000.000 6.10 3.33 3.07 6.34 5.86

40248000.000 3.20223 412480000.000 6.10 3.33 3.07 6.34 5.86

40255000.000 3.20226 412550000.000 6.10 3.33 3.07 6.35 5.86

40262000.000 3.20229 412620000.000 6.10 3.33 3.07 6.35 5.86

40269000.000 3.20232 412690000.000 6.10 3.33 3.07 6.35 5.86

40276000.000 3.20235 412760000.000 6.10 3.33 3.07 6.35 5.86

40283000.000 3.20237 412830000.000 6.10 3.33 3.07 6.35 5.86

40290000.000 3.20240 412900000.000 6.10 3.33 3.07 6.35 5.86

40297000.000 3.20243 412970000.000 6.10 3.33 3.07 6.35 5.86

40304000.000 3.20246 413040000.000 6.10 3.33 3.07 6.35 5.86

40311000.000 3.20248 413110000.000 6.10 3.33 3.07 6.35 5.86

40318000.000 3.20251 413180000.000 6.10 3.33 3.07 6.35 5.86

40325000.000 3.20254 413250000.000 6.10 3.33 3.07 6.35 5.86

40332000.000 3.20257 413320000.000 6.10 3.33 3.07 6.35 5.86

40339000.000 3.20260 413390000.000 6.10 3.33 3.07 6.35 5.86

40346000.000 3.20262 413460000.000 6.10 3.33 3.07 6.35 5.86

40353000.000 3.20265 413530000.000 6.10 3.33 3.07 6.35 5.86

40360000.000 3.20268 413600000.000 6.10 3.33 3.07 6.35 5.86

40367000.000 3.20271 413670000.000 6.10 3.33 3.07 6.35 5.86

40374000.000 3.20274 413740000.000 6.10 3.33 3.07 6.35 5.86

40381000.000 3.20276 413810000.000 6.10 3.33 3.07 6.35 5.86

40388000.000 3.20279 413880000.000 6.10 3.33 3.07 6.35 5.86

40395000.000 3.20282 413950000.000 6.10 3.33 3.07 6.35 5.86

40402000.000 3.20285 414020000.000 6.10 3.33 3.07 6.35 5.86

40409000.000 3.20287 414090000.000 6.10 3.33 3.07 6.35 5.86

40416000.000 3.20290 414160000.000 6.10 3.33 3.07 6.35 5.86

40423000.000 3.20293 414230000.000 6.10 3.33 3.07 6.35 5.86

40430000.000 3.20296 414300000.000 6.10 3.33 3.07 6.35 5.86

40437000.000 3.20299 414370000.000 6.10 3.33 3.07 6.35 5.86

40444000.000 3.20301 414440000.000 6.10 3.33 3.07 6.35 5.86

40451000.000 3.20304 414510000.000 6.10 3.33 3.07 6.35 5.86

40458000.000 3.20307 414580000.000 6.10 3.33 3.07 6.35 5.86

40465000.000 3.20310 414650000.000 6.10 3.33 3.07 6.35 5.86

40472000.000 3.20313 414720000.000 6.10 3.33 3.08 6.35 5.86

40479000.000 3.20315 414790000.000 6.10 3.33 3.08 6.35 5.86

40486000.000 3.20318 414860000.000 6.10 3.33 3.08 6.35 5.86

40493000.000 3.20321 414930000.000 6.10 3.33 3.08 6.35 5.86

40500000.000 3.20324 415000000.000 6.10 3.33 3.08 6.35 5.86

40507000.000 3.20326 415070000.000 6.10 3.33 3.08 6.35 5.86

40514000.000 3.20329 415140000.000 6.10 3.33 3.08 6.35 5.86

40521000.000 3.20332 415210000.000 6.10 3.33 3.08 6.35 5.86

40528000.000 3.20335 415280000.000 6.10 3.33 3.08 6.35 5.86

40535000.000 3.20338 415350000.000 6.10 3.33 3.08 6.35 5.86

40542000.000 3.20340 415420000.000 6.10 3.33 3.08 6.35 5.86

40549000.000 3.20343 415490000.000 6.10 3.33 3.08 6.35 5.86

40556000.000 3.20346 415560000.000 6.10 3.33 3.08 6.35 5.86

40563000.000 3.20349 415630000.000 6.10 3.33 3.08 6.35 5.86

40570000.000 3.20351 415700000.000 6.10 3.33 3.08 6.35 5.86

40577000.000 3.20354 415770000.000 6.10 3.33 3.08 6.35 5.86

40584000.000 3.20357 415840000.000 6.10 3.33 3.08 6.35 5.86

40591000.000 3.20360 415910000.000 6.10 3.33 3.08 6.35 5.86

40598000.000 3.20362 415980000.000 6.10 3.33 3.08 6.35 5.86

40605000.000 3.20365 416050000.000 6.10 3.33 3.08 6.35 5.86

40612000.000 3.20368 416120000.000 6.10 3.33 3.08 6.35 5.86

40619000.000 3.20371 416190000.000 6.10 3.33 3.08 6.35 5.86

40626000.000 3.20374 416260000.000 6.10 3.33 3.08 6.35 5.86

40633000.000 3.20376 416330000.000 6.10 3.33 3.08 6.35 5.86

40640000.000 3.20379 416400000.000 6.10 3.33 3.08 6.35 5.86

40647000.000 3.20382 416470000.000 6.10 3.33 3.08 6.35 5.86

40654000.000 3.20385 416540000.000 6.10 3.33 3.08 6.35 5.86

40661000.000 3.20387 416610000.000 6.10 3.33 3.08 6.35 5.86

40668000.000 3.20390 416680000.000 6.10 3.33 3.08 6.35 5.86

40675000.000 3.20393 416750000.000 6.10 3.33 3.08 6.35 5.86

40682000.000 3.20396 416820000.000 6.10 3.33 3.08 6.35 5.86

40689000.000 3.20398 416890000.000 6.10 3.33 3.08 6.35 5.86

40696000.000 3.20401 416960000.000 6.10 3.33 3.08 6.35 5.86

40703000.000 3.20404 417030000.000 6.10 3.33 3.08 6.35 5.86

40710000.000 3.20407 417100000.000 6.10 3.33 3.08 6.35 5.86

40717000.000 3.20410 417170000.000 6.10 3.33 3.08 6.35 5.86

40724000.000 3.20412 417240000.000 6.10 3.33 3.08 6.35 5.86

40731000.000 3.20415 417310000.000 6.10 3.33 3.08 6.35 5.86

40738000.000 3.20418 417380000.000 6.10 3.33 3.08 6.35 5.86

40745000.000 3.20421 417450000.000 6.10 3.33 3.08 6.35 5.86

40752000.000 3.20423 417520000.000 6.10 3.33 3.08 6.35 5.86

40759000.000 3.20426 417590000.000 6.10 3.33 3.08 6.35 5.86

40766000.000 3.20429 417660000.000 6.10 3.33 3.08 6.35 5.86

40773000.000 3.20432 417730000.000 6.10 3.33 3.08 6.35 5.86

40780000.000 3.20434 417800000.000 6.10 3.33 3.08 6.35 5.86

40787000.000 3.20437 417870000.000 6.10 3.33 3.08 6.35 5.86

40794000.000 3.20440 417940000.000 6.10 3.33 3.08 6.35 5.86

40801000.000 3.20443 418010000.000 6.11 3.33 3.08 6.35 5.86

40808000.000 3.20445 418080000.000 6.11 3.33 3.08 6.35 5.86

40815000.000 3.20448 418150000.000 6.11 3.33 3.08 6.35 5.86

40822000.000 3.20451 418220000.000 6.11 3.33 3.08 6.35 5.86

40829000.000 3.20454 418290000.000 6.11 3.33 3.08 6.35 5.86

40836000.000 3.20456 418360000.000 6.11 3.33 3.08 6.35 5.86

40843000.000 3.20459 418430000.000 6.11 3.33 3.08 6.35 5.86

40850000.000 3.20462 418500000.000 6.11 3.33 3.08 6.35 5.86

40857000.000 3.20465 418570000.000 6.11 3.33 3.08 6.35 5.86

40864000.000 3.20467 418640000.000 6.11 3.33 3.08 6.35 5.86

40871000.000 3.20470 418710000.000 6.11 3.33 3.08 6.35 5.86

40878000.000 3.20473 418780000.000 6.11 3.33 3.08 6.35 5.86

40885000.000 3.20476 418850000.000 6.11 3.33 3.08 6.35 5.86

40892000.000 3.20478 418920000.000 6.11 3.33 3.08 6.35 5.86

40899000.000 3.20481 418990000.000 6.11 3.33 3.08 6.35 5.86

40906000.000 3.20484 419060000.000 6.11 3.33 3.08 6.35 5.86

40913000.000 3.20487 419130000.000 6.11 3.33 3.08 6.35 5.86

40920000.000 3.20489 419200000.000 6.11 3.33 3.08 6.35 5.86

40927000.000 3.20492 419270000.000 6.11 3.33 3.08 6.35 5.86

40934000.000 3.20495 419340000.000 6.11 3.33 3.08 6.35 5.86

40941000.000 3.20498 419410000.000 6.11 3.33 3.08 6.35 5.86

40948000.000 3.20500 419480000.000 6.11 3.33 3.08 6.35 5.86

40955000.000 3.20503 419550000.000 6.11 3.33 3.08 6.35 5.86

40962000.000 3.20506 419620000.000 6.11 3.33 3.08 6.35 5.86

40969000.000 3.20509 419690000.000 6.11 3.33 3.08 6.35 5.86

40976000.000 3.20511 419760000.000 6.11 3.33 3.08 6.35 5.86

40983000.000 3.20514 419830000.000 6.11 3.33 3.08 6.35 5.86

40990000.000 3.20517 419900000.000 6.11 3.33 3.08 6.35 5.86

40997000.000 3.20520 419970000.000 6.11 3.33 3.08 6.35 5.86

41004000.000 3.20522 420040000.000 6.11 3.33 3.08 6.35 5.86

41011000.000 3.20525 420110000.000 6.11 3.33 3.08 6.35 5.86

41018000.000 3.20528 420180000.000 6.11 3.33 3.08 6.35 5.86

41025000.000 3.20531 420250000.000 6.11 3.33 3.08 6.35 5.86

41032000.000 3.20533 420320000.000 6.11 3.33 3.08 6.35 5.86

41039000.000 3.20536 420390000.000 6.11 3.33 3.08 6.35 5.86

41046000.000 3.20539 420460000.000 6.11 3.33 3.08 6.35 5.86

41053000.000 3.20542 420530000.000 6.11 3.33 3.08 6.35 5.86

41060000.000 3.20544 420600000.000 6.11 3.33 3.08 6.35 5.86

41067000.000 3.20547 420670000.000 6.11 3.33 3.08 6.35 5.86

41074000.000 3.20550 420740000.000 6.11 3.33 3.08 6.35 5.86

41081000.000 3.20553 420810000.000 6.11 3.33 3.08 6.35 5.86

41088000.000 3.20555 420880000.000 6.11 3.33 3.08 6.35 5.86

41095000.000 3.20558 420950000.000 6.11 3.33 3.08 6.35 5.86

41102000.000 3.20561 421020000.000 6.11 3.33 3.08 6.35 5.86

41109000.000 3.20564 421090000.000 6.11 3.33 3.08 6.35 5.86

41116000.000 3.20566 421160000.000 6.11 3.33 3.08 6.35 5.86

41123000.000 3.20569 421230000.000 6.11 3.33 3.08 6.35 5.86

41130000.000 3.20572 421300000.000 6.11 3.33 3.08 6.35 5.86

41137000.000 3.20574 421370000.000 6.11 3.33 3.08 6.35 5.86

41144000.000 3.20577 421440000.000 6.11 3.33 3.08 6.35 5.86

41151000.000 3.20580 421510000.000 6.11 3.33 3.08 6.35 5.86

41158000.000 3.20583 421580000.000 6.11 3.33 3.08 6.35 5.86

41165000.000 3.20585 421650000.000 6.11 3.33 3.08 6.35 5.86

41172000.000 3.20588 421720000.000 6.11 3.33 3.08 6.35 5.86

41179000.000 3.20591 421790000.000 6.11 3.33 3.08 6.35 5.86

41186000.000 3.20594 421860000.000 6.11 3.33 3.08 6.35 5.86

41193000.000 3.20596 421930000.000 6.11 3.33 3.08 6.35 5.86

41200000.000 3.20599 422000000.000 6.11 3.33 3.08 6.35 5.86

41207000.000 3.20602 422070000.000 6.11 3.33 3.08 6.35 5.86

41214000.000 3.20605 422140000.000 6.11 3.33 3.08 6.35 5.86

41221000.000 3.20607 422210000.000 6.11 3.33 3.08 6.35 5.86

41228000.000 3.20610 422280000.000 6.11 3.33 3.08 6.35 5.86

41235000.000 3.20613 422350000.000 6.11 3.33 3.08 6.35 5.86

41242000.000 3.20615 422420000.000 6.11 3.33 3.08 6.35 5.86

41249000.000 3.20618 422490000.000 6.11 3.33 3.08 6.35 5.86

41256000.000 3.20621 422560000.000 6.11 3.33 3.08 6.35 5.86

41263000.000 3.20624 422630000.000 6.11 3.33 3.08 6.35 5.86

41270000.000 3.20626 422700000.000 6.11 3.33 3.08 6.35 5.86

41277000.000 3.20629 422770000.000 6.11 3.33 3.08 6.35 5.86

41284000.000 3.20632 422840000.000 6.11 3.33 3.08 6.35 5.86

41291000.000 3.20635 422910000.000 6.11 3.33 3.08 6.35 5.86

41298000.000 3.20637 422980000.000 6.11 3.33 3.08 6.35 5.86

41305000.000 3.20640 423050000.000 6.11 3.33 3.08 6.35 5.86

41312000.000 3.20643 423120000.000 6.11 3.33 3.08 6.35 5.86

41319000.000 3.20645 423190000.000 6.11 3.33 3.08 6.35 5.86

41326000.000 3.20648 423260000.000 6.11 3.33 3.08 6.35 5.86

41333000.000 3.20651 423330000.000 6.11 3.33 3.08 6.35 5.86

41340000.000 3.20654 423400000.000 6.11 3.33 3.08 6.35 5.86

41347000.000 3.20656 423470000.000 6.11 3.33 3.08 6.35 5.86

41354000.000 3.20659 423540000.000 6.11 3.33 3.08 6.35 5.86

41361000.000 3.20662 423610000.000 6.11 3.33 3.08 6.35 5.86

41368000.000 3.20665 423680000.000 6.11 3.33 3.08 6.35 5.86

41375000.000 3.20667 423750000.000 6.11 3.33 3.08 6.35 5.86

41382000.000 3.20670 423820000.000 6.11 3.33 3.08 6.35 5.86

41389000.000 3.20673 423890000.000 6.11 3.33 3.08 6.35 5.86

41396000.000 3.20675 423960000.000 6.11 3.34 3.08 6.35 5.86

41403000.000 3.20678 424030000.000 6.11 3.34 3.08 6.35 5.87

41410000.000 3.20681 424100000.000 6.11 3.34 3.08 6.35 5.87

41417000.000 3.20684 424170000.000 6.11 3.34 3.08 6.35 5.87

41424000.000 3.20686 424240000.000 6.11 3.34 3.08 6.35 5.87

41431000.000 3.20689 424310000.000 6.11 3.34 3.08 6.35 5.87

41438000.000 3.20692 424380000.000 6.11 3.34 3.08 6.35 5.87

41445000.000 3.20694 424450000.000 6.11 3.34 3.08 6.35 5.87

41452000.000 3.20697 424520000.000 6.11 3.34 3.08 6.35 5.87

41459000.000 3.20700 424590000.000 6.11 3.34 3.08 6.35 5.87

41466000.000 3.20703 424660000.000 6.11 3.34 3.08 6.35 5.87

41473000.000 3.20705 424730000.000 6.11 3.34 3.08 6.35 5.87

41480000.000 3.20708 424800000.000 6.11 3.34 3.08 6.35 5.87

41487000.000 3.20711 424870000.000 6.11 3.34 3.08 6.35 5.87

41494000.000 3.20713 424940000.000 6.11 3.34 3.08 6.35 5.87

41501000.000 3.20716 425010000.000 6.11 3.34 3.08 6.35 5.87

41508000.000 3.20719 425080000.000 6.11 3.34 3.08 6.35 5.87

41515000.000 3.20722 425150000.000 6.11 3.34 3.08 6.35 5.87

41522000.000 3.20724 425220000.000 6.11 3.34 3.08 6.35 5.87

41529000.000 3.20727 425290000.000 6.11 3.34 3.08 6.35 5.87

41536000.000 3.20730 425360000.000 6.11 3.34 3.08 6.35 5.87

41543000.000 3.20732 425430000.000 6.11 3.34 3.08 6.35 5.87

41550000.000 3.20735 425500000.000 6.11 3.34 3.08 6.35 5.87

41557000.000 3.20738 425570000.000 6.11 3.34 3.08 6.35 5.87

41564000.000 3.20741 425640000.000 6.11 3.34 3.08 6.35 5.87

41571000.000 3.20743 425710000.000 6.11 3.34 3.08 6.36 5.87

41578000.000 3.20746 425780000.000 6.11 3.34 3.08 6.36 5.87

41585000.000 3.20749 425850000.000 6.11 3.34 3.08 6.36 5.87

41592000.000 3.20751 425920000.000 6.11 3.34 3.08 6.36 5.87

41599000.000 3.20754 425990000.000 6.11 3.34 3.08 6.36 5.87

41606000.000 3.20757 426060000.000 6.11 3.34 3.08 6.36 5.87

41613000.000 3.20760 426130000.000 6.11 3.34 3.08 6.36 5.87

41620000.000 3.20762 426200000.000 6.11 3.34 3.08 6.36 5.87

41627000.000 3.20765 426270000.000 6.11 3.34 3.08 6.36 5.87

41634000.000 3.20768 426340000.000 6.11 3.34 3.08 6.36 5.87

41641000.000 3.20770 426410000.000 6.11 3.34 3.08 6.36 5.87

41648000.000 3.20773 426480000.000 6.11 3.34 3.08 6.36 5.87

41655000.000 3.20776 426550000.000 6.11 3.34 3.08 6.36 5.87

41662000.000 3.20778 426620000.000 6.11 3.34 3.08 6.36 5.87

41669000.000 3.20781 426690000.000 6.11 3.34 3.08 6.36 5.87

41676000.000 3.20784 426760000.000 6.11 3.34 3.08 6.36 5.87

41683000.000 3.20787 426830000.000 6.11 3.34 3.08 6.36 5.87

41690000.000 3.20789 426900000.000 6.11 3.34 3.08 6.36 5.87

41697000.000 3.20792 426970000.000 6.11 3.34 3.08 6.36 5.87

41704000.000 3.20795 427040000.000 6.11 3.34 3.08 6.36 5.87

41711000.000 3.20797 427110000.000 6.11 3.34 3.08 6.36 5.87

41718000.000 3.20800 427180000.000 6.11 3.34 3.08 6.36 5.87

41725000.000 3.20803 427250000.000 6.11 3.34 3.08 6.36 5.87

41732000.000 3.20805 427320000.000 6.11 3.34 3.08 6.36 5.87

41739000.000 3.20808 427390000.000 6.11 3.34 3.08 6.36 5.87

41746000.000 3.20811 427460000.000 6.11 3.34 3.08 6.36 5.87

41753000.000 3.20814 427530000.000 6.11 3.34 3.08 6.36 5.87

41760000.000 3.20816 427600000.000 6.11 3.34 3.08 6.36 5.87

41767000.000 3.20819 427670000.000 6.11 3.34 3.08 6.36 5.87

41774000.000 3.20822 427740000.000 6.11 3.34 3.08 6.36 5.87

41781000.000 3.20824 427810000.000 6.11 3.34 3.08 6.36 5.87

41788000.000 3.20827 427880000.000 6.11 3.34 3.08 6.36 5.87

41795000.000 3.20830 427950000.000 6.11 3.34 3.08 6.36 5.87

41802000.000 3.20832 428020000.000 6.11 3.34 3.08 6.36 5.87

41809000.000 3.20835 428090000.000 6.11 3.34 3.08 6.36 5.87

41816000.000 3.20838 428160000.000 6.11 3.34 3.08 6.36 5.87

41823000.000 3.20841 428230000.000 6.11 3.34 3.08 6.36 5.87

41830000.000 3.20843 428300000.000 6.11 3.34 3.08 6.36 5.87

41837000.000 3.20846 428370000.000 6.11 3.34 3.08 6.36 5.87

41844000.000 3.20849 428440000.000 6.11 3.34 3.08 6.36 5.87

41851000.000 3.20851 428510000.000 6.11 3.34 3.08 6.36 5.87

41858000.000 3.20854 428580000.000 6.11 3.34 3.08 6.36 5.87

41865000.000 3.20857 428650000.000 6.11 3.34 3.08 6.36 5.87

41872000.000 3.20859 428720000.000 6.11 3.34 3.08 6.36 5.87

41879000.000 3.20862 428790000.000 6.11 3.34 3.08 6.36 5.87

41886000.000 3.20865 428860000.000 6.11 3.34 3.08 6.36 5.87

41893000.000 3.20867 428930000.000 6.11 3.34 3.08 6.36 5.87

41900000.000 3.20870 429000000.000 6.11 3.34 3.08 6.36 5.87

41907000.000 3.20873 429070000.000 6.11 3.34 3.08 6.36 5.87

41914000.000 3.20876 429140000.000 6.11 3.34 3.08 6.36 5.87

41921000.000 3.20878 429210000.000 6.11 3.34 3.08 6.36 5.87

41928000.000 3.20881 429280000.000 6.11 3.34 3.08 6.36 5.87

41935000.000 3.20884 429350000.000 6.11 3.34 3.08 6.36 5.87

41942000.000 3.20886 429420000.000 6.11 3.34 3.08 6.36 5.87

41949000.000 3.20889 429490000.000 6.11 3.34 3.08 6.36 5.87

41956000.000 3.20892 429560000.000 6.11 3.34 3.08 6.36 5.87

41963000.000 3.20894 429630000.000 6.11 3.34 3.08 6.36 5.87

41970000.000 3.20897 429700000.000 6.11 3.34 3.08 6.36 5.87

41977000.000 3.20900 429770000.000 6.11 3.34 3.08 6.36 5.87

41984000.000 3.20902 429840000.000 6.11 3.34 3.08 6.36 5.87

41991000.000 3.20905 429910000.000 6.11 3.34 3.08 6.36 5.87

41998000.000 3.20908 429980000.000 6.11 3.34 3.08 6.36 5.87

42005000.000 3.20910 430050000.000 6.11 3.34 3.08 6.36 5.87

42012000.000 3.20913 430120000.000 6.11 3.34 3.08 6.36 5.87

42019000.000 3.20916 430190000.000 6.11 3.34 3.08 6.36 5.87

42026000.000 3.20918 430260000.000 6.11 3.34 3.08 6.36 5.87

42033000.000 3.20921 430330000.000 6.11 3.34 3.08 6.36 5.87

42040000.000 3.20924 430400000.000 6.11 3.34 3.08 6.36 5.87

42047000.000 3.20927 430470000.000 6.11 3.34 3.08 6.36 5.87

42054000.000 3.20929 430540000.000 6.11 3.34 3.08 6.36 5.87

42061000.000 3.20932 430610000.000 6.11 3.34 3.08 6.36 5.87

42068000.000 3.20935 430680000.000 6.11 3.34 3.08 6.36 5.87

42075000.000 3.20937 430750000.000 6.11 3.34 3.08 6.36 5.87

42082000.000 3.20940 430820000.000 6.11 3.34 3.08 6.36 5.87

42089000.000 3.20943 430890000.000 6.11 3.34 3.08 6.36 5.87

42096000.000 3.20945 430960000.000 6.11 3.34 3.08 6.36 5.87

42103000.000 3.20948 431030000.000 6.11 3.34 3.08 6.36 5.87

42110000.000 3.20951 431100000.000 6.11 3.34 3.08 6.36 5.87

42117000.000 3.20953 431170000.000 6.11 3.34 3.08 6.36 5.87

42124000.000 3.20956 431240000.000 6.11 3.34 3.08 6.36 5.87

42131000.000 3.20959 431310000.000 6.11 3.34 3.08 6.36 5.87

42138000.000 3.20961 431380000.000 6.11 3.34 3.08 6.36 5.87

42145000.000 3.20964 431450000.000 6.11 3.34 3.08 6.36 5.87

42152000.000 3.20967 431520000.000 6.11 3.34 3.08 6.36 5.87

42159000.000 3.20969 431590000.000 6.11 3.34 3.08 6.36 5.87

42166000.000 3.20972 431660000.000 6.11 3.34 3.08 6.36 5.87

42173000.000 3.20975 431730000.000 6.11 3.34 3.08 6.36 5.87

42180000.000 3.20977 431800000.000 6.11 3.34 3.08 6.36 5.87

42187000.000 3.20980 431870000.000 6.12 3.34 3.08 6.36 5.87

42194000.000 3.20983 431940000.000 6.12 3.34 3.08 6.36 5.87

42201000.000 3.20985 432010000.000 6.12 3.34 3.08 6.36 5.87

42208000.000 3.20988 432080000.000 6.12 3.34 3.08 6.36 5.87

42215000.000 3.20991 432150000.000 6.12 3.34 3.08 6.36 5.87

42222000.000 3.20993 432220000.000 6.12 3.34 3.08 6.36 5.87

42229000.000 3.20996 432290000.000 6.12 3.34 3.08 6.36 5.87

42236000.000 3.20999 432360000.000 6.12 3.34 3.08 6.36 5.87

42243000.000 3.21001 432430000.000 6.12 3.34 3.08 6.36 5.87

42250000.000 3.21004 432500000.000 6.12 3.34 3.08 6.36 5.87

42257000.000 3.21007 432570000.000 6.12 3.34 3.08 6.36 5.87

42264000.000 3.21009 432640000.000 6.12 3.34 3.08 6.36 5.87

42271000.000 3.21012 432710000.000 6.12 3.34 3.08 6.36 5.87

42278000.000 3.21015 432780000.000 6.12 3.34 3.08 6.36 5.87

42285000.000 3.21017 432850000.000 6.12 3.34 3.08 6.36 5.87

42292000.000 3.21020 432920000.000 6.12 3.34 3.08 6.36 5.87

42299000.000 3.21023 432990000.000 6.12 3.34 3.08 6.36 5.87

42306000.000 3.21025 433060000.000 6.12 3.34 3.08 6.36 5.87

42313000.000 3.21028 433130000.000 6.12 3.34 3.08 6.36 5.87

42320000.000 3.21031 433200000.000 6.12 3.34 3.08 6.36 5.87

42327000.000 3.21033 433270000.000 6.12 3.34 3.08 6.36 5.87

42334000.000 3.21036 433340000.000 6.12 3.34 3.08 6.36 5.87

42341000.000 3.21039 433410000.000 6.12 3.34 3.08 6.36 5.87

42348000.000 3.21041 433480000.000 6.12 3.34 3.08 6.36 5.87

42355000.000 3.21044 433550000.000 6.12 3.34 3.08 6.36 5.87

42362000.000 3.21047 433620000.000 6.12 3.34 3.08 6.36 5.87

42369000.000 3.21049 433690000.000 6.12 3.34 3.08 6.36 5.87

42376000.000 3.21052 433760000.000 6.12 3.34 3.08 6.36 5.87

42383000.000 3.21055 433830000.000 6.12 3.34 3.08 6.36 5.87

42390000.000 3.21057 433900000.000 6.12 3.34 3.08 6.36 5.87

42397000.000 3.21060 433970000.000 6.12 3.34 3.08 6.36 5.87

42404000.000 3.21063 434040000.000 6.12 3.34 3.08 6.36 5.87

42411000.000 3.21065 434110000.000 6.12 3.34 3.08 6.36 5.87

42418000.000 3.21068 434180000.000 6.12 3.34 3.08 6.36 5.87

42425000.000 3.21071 434250000.000 6.12 3.34 3.08 6.36 5.87

42432000.000 3.21073 434320000.000 6.12 3.34 3.08 6.36 5.87

42439000.000 3.21076 434390000.000 6.12 3.34 3.08 6.36 5.87

42446000.000 3.21079 434460000.000 6.12 3.34 3.08 6.36 5.87

42453000.000 3.21081 434530000.000 6.12 3.34 3.08 6.36 5.87

42460000.000 3.21084 434600000.000 6.12 3.34 3.08 6.36 5.87

42467000.000 3.21087 434670000.000 6.12 3.34 3.08 6.36 5.87

42474000.000 3.21089 434740000.000 6.12 3.34 3.08 6.36 5.87

42481000.000 3.21092 434810000.000 6.12 3.34 3.08 6.36 5.87

42488000.000 3.21095 434880000.000 6.12 3.34 3.08 6.36 5.87

42495000.000 3.21097 434950000.000 6.12 3.34 3.08 6.36 5.87

42502000.000 3.21100 435020000.000 6.12 3.34 3.08 6.36 5.87

42509000.000 3.21102 435090000.000 6.12 3.34 3.08 6.36 5.87

42516000.000 3.21105 435160000.000 6.12 3.34 3.08 6.36 5.87

42523000.000 3.21108 435230000.000 6.12 3.34 3.08 6.36 5.87

42530000.000 3.21110 435300000.000 6.12 3.34 3.08 6.36 5.87

42537000.000 3.21113 435370000.000 6.12 3.34 3.08 6.36 5.87

42544000.000 3.21116 435440000.000 6.12 3.34 3.08 6.36 5.87

42551000.000 3.21118 435510000.000 6.12 3.34 3.08 6.36 5.87

42558000.000 3.21121 435580000.000 6.12 3.34 3.08 6.36 5.87

42565000.000 3.21124 435650000.000 6.12 3.34 3.08 6.36 5.87

42572000.000 3.21126 435720000.000 6.12 3.34 3.08 6.36 5.87

42579000.000 3.21129 435790000.000 6.12 3.34 3.08 6.36 5.87

42586000.000 3.21132 435860000.000 6.12 3.34 3.08 6.36 5.87

42593000.000 3.21134 435930000.000 6.12 3.34 3.08 6.36 5.87

42600000.000 3.21137 436000000.000 6.12 3.34 3.08 6.36 5.87

42607000.000 3.21140 436070000.000 6.12 3.34 3.08 6.36 5.87

42614000.000 3.21142 436140000.000 6.12 3.34 3.08 6.36 5.87

42621000.000 3.21145 436210000.000 6.12 3.34 3.08 6.36 5.87

42628000.000 3.21148 436280000.000 6.12 3.34 3.08 6.36 5.87

42635000.000 3.21150 436350000.000 6.12 3.34 3.08 6.36 5.87

42642000.000 3.21153 436420000.000 6.12 3.34 3.08 6.36 5.87

42649000.000 3.21155 436490000.000 6.12 3.34 3.08 6.36 5.87

42656000.000 3.21158 436560000.000 6.12 3.34 3.08 6.36 5.87

42663000.000 3.21161 436630000.000 6.12 3.34 3.08 6.36 5.87

42670000.000 3.21163 436700000.000 6.12 3.34 3.08 6.36 5.87

42677000.000 3.21166 436770000.000 6.12 3.34 3.08 6.36 5.87

42684000.000 3.21169 436840000.000 6.12 3.34 3.08 6.36 5.87

42691000.000 3.21171 436910000.000 6.12 3.34 3.08 6.36 5.87

42698000.000 3.21174 436980000.000 6.12 3.34 3.08 6.36 5.87

42705000.000 3.21177 437050000.000 6.12 3.34 3.08 6.36 5.87

42712000.000 3.21179 437120000.000 6.12 3.34 3.08 6.36 5.87

42719000.000 3.21182 437190000.000 6.12 3.34 3.08 6.36 5.87

42726000.000 3.21184 437260000.000 6.12 3.34 3.08 6.36 5.87

42733000.000 3.21187 437330000.000 6.12 3.34 3.08 6.36 5.87

42740000.000 3.21190 437400000.000 6.12 3.34 3.08 6.36 5.87

42747000.000 3.21192 437470000.000 6.12 3.34 3.08 6.36 5.87

42754000.000 3.21195 437540000.000 6.12 3.34 3.08 6.36 5.87

42761000.000 3.21198 437610000.000 6.12 3.34 3.08 6.36 5.87

42768000.000 3.21200 437680000.000 6.12 3.34 3.08 6.36 5.87

42775000.000 3.21203 437750000.000 6.12 3.34 3.08 6.36 5.87

42782000.000 3.21206 437820000.000 6.12 3.34 3.08 6.36 5.87

42789000.000 3.21208 437890000.000 6.12 3.34 3.08 6.36 5.87

42796000.000 3.21211 437960000.000 6.12 3.34 3.08 6.36 5.87

42803000.000 3.21214 438030000.000 6.12 3.34 3.08 6.36 5.87

42810000.000 3.21216 438100000.000 6.12 3.34 3.08 6.36 5.87

42817000.000 3.21219 438170000.000 6.12 3.34 3.08 6.36 5.87

42824000.000 3.21221 438240000.000 6.12 3.34 3.08 6.36 5.87

42831000.000 3.21224 438310000.000 6.12 3.34 3.08 6.36 5.87

42838000.000 3.21227 438380000.000 6.12 3.34 3.08 6.36 5.87

42845000.000 3.21229 438450000.000 6.12 3.34 3.08 6.36 5.87

42852000.000 3.21232 438520000.000 6.12 3.34 3.08 6.36 5.87

42859000.000 3.21235 438590000.000 6.12 3.34 3.08 6.36 5.87

42866000.000 3.21237 438660000.000 6.12 3.34 3.08 6.36 5.88

42873000.000 3.21240 438730000.000 6.12 3.34 3.08 6.36 5.88

42880000.000 3.21242 438800000.000 6.12 3.34 3.08 6.36 5.88

42887000.000 3.21245 438870000.000 6.12 3.34 3.08 6.36 5.88

42894000.000 3.21248 438940000.000 6.12 3.34 3.08 6.36 5.88

42901000.000 3.21250 439010000.000 6.12 3.34 3.08 6.36 5.88

42908000.000 3.21253 439080000.000 6.12 3.34 3.08 6.36 5.88

42915000.000 3.21256 439150000.000 6.12 3.34 3.08 6.36 5.88

42922000.000 3.21258 439220000.000 6.12 3.34 3.08 6.36 5.88

42929000.000 3.21261 439290000.000 6.12 3.34 3.08 6.37 5.88

42936000.000 3.21263 439360000.000 6.12 3.34 3.08 6.37 5.88

42943000.000 3.21266 439430000.000 6.12 3.34 3.08 6.37 5.88

42950000.000 3.21269 439500000.000 6.12 3.34 3.08 6.37 5.88

42957000.000 3.21271 439570000.000 6.12 3.34 3.08 6.37 5.88

42964000.000 3.21274 439640000.000 6.12 3.34 3.08 6.37 5.88

42971000.000 3.21277 439710000.000 6.12 3.34 3.08 6.37 5.88

42978000.000 3.21279 439780000.000 6.12 3.34 3.08 6.37 5.88

42985000.000 3.21282 439850000.000 6.12 3.34 3.08 6.37 5.88

42992000.000 3.21284 439920000.000 6.12 3.34 3.08 6.37 5.88

42999000.000 3.21287 439990000.000 6.12 3.34 3.08 6.37 5.88

43006000.000 3.21290 440060000.000 6.12 3.34 3.08 6.37 5.88

43013000.000 3.21292 440130000.000 6.12 3.34 3.08 6.37 5.88

43020000.000 3.21295 440200000.000 6.12 3.34 3.08 6.37 5.88

43027000.000 3.21298 440270000.000 6.12 3.34 3.08 6.37 5.88

43034000.000 3.21300 440340000.000 6.12 3.34 3.08 6.37 5.88

43041000.000 3.21303 440410000.000 6.12 3.34 3.08 6.37 5.88

43048000.000 3.21305 440480000.000 6.12 3.34 3.08 6.37 5.88

43055000.000 3.21308 440550000.000 6.12 3.34 3.08 6.37 5.88

43062000.000 3.21311 440620000.000 6.12 3.34 3.08 6.37 5.88

43069000.000 3.21313 440690000.000 6.12 3.34 3.08 6.37 5.88

43076000.000 3.21316 440760000.000 6.12 3.34 3.08 6.37 5.88

43083000.000 3.21319 440830000.000 6.12 3.34 3.08 6.37 5.88

43090000.000 3.21321 440900000.000 6.12 3.34 3.08 6.37 5.88

43097000.000 3.21324 440970000.000 6.12 3.34 3.08 6.37 5.88

43104000.000 3.21326 441040000.000 6.12 3.34 3.08 6.37 5.88

43111000.000 3.21329 441110000.000 6.12 3.34 3.08 6.37 5.88

43118000.000 3.21332 441180000.000 6.12 3.34 3.08 6.37 5.88

43125000.000 3.21334 441250000.000 6.12 3.34 3.08 6.37 5.88

43132000.000 3.21337 441320000.000 6.12 3.34 3.08 6.37 5.88

43139000.000 3.21340 441390000.000 6.12 3.34 3.08 6.37 5.88

43146000.000 3.21342 441460000.000 6.12 3.34 3.08 6.37 5.88

43153000.000 3.21345 441530000.000 6.12 3.34 3.08 6.37 5.88

43160000.000 3.21347 441600000.000 6.12 3.34 3.08 6.37 5.88

43167000.000 3.21350 441670000.000 6.12 3.34 3.08 6.37 5.88

43174000.000 3.21353 441740000.000 6.12 3.34 3.08 6.37 5.88

43181000.000 3.21355 441810000.000 6.12 3.34 3.09 6.37 5.88

43188000.000 3.21358 441880000.000 6.12 3.34 3.09 6.37 5.88

43195000.000 3.21360 441950000.000 6.12 3.34 3.09 6.37 5.88

43202000.000 3.21363 442020000.000 6.12 3.34 3.09 6.37 5.88

43209000.000 3.21366 442090000.000 6.12 3.34 3.09 6.37 5.88

43216000.000 3.21368 442160000.000 6.12 3.34 3.09 6.37 5.88

43223000.000 3.21371 442230000.000 6.12 3.34 3.09 6.37 5.88

43230000.000 3.21373 442300000.000 6.12 3.34 3.09 6.37 5.88

43237000.000 3.21376 442370000.000 6.12 3.34 3.09 6.37 5.88

43244000.000 3.21379 442440000.000 6.12 3.34 3.09 6.37 5.88

43251000.000 3.21381 442510000.000 6.12 3.34 3.09 6.37 5.88

43258000.000 3.21384 442580000.000 6.12 3.34 3.09 6.37 5.88

43265000.000 3.21387 442650000.000 6.12 3.34 3.09 6.37 5.88

43272000.000 3.21389 442720000.000 6.12 3.34 3.09 6.37 5.88

43279000.000 3.21392 442790000.000 6.12 3.34 3.09 6.37 5.88

43286000.000 3.21394 442860000.000 6.12 3.34 3.09 6.37 5.88

43293000.000 3.21397 442930000.000 6.12 3.34 3.09 6.37 5.88

43300000.000 3.21400 443000000.000 6.12 3.34 3.09 6.37 5.88

43307000.000 3.21402 443070000.000 6.12 3.34 3.09 6.37 5.88

43314000.000 3.21405 443140000.000 6.12 3.34 3.09 6.37 5.88

43321000.000 3.21407 443210000.000 6.12 3.34 3.09 6.37 5.88

43328000.000 3.21410 443280000.000 6.12 3.34 3.09 6.37 5.88

43335000.000 3.21413 443350000.000 6.12 3.34 3.09 6.37 5.88

43342000.000 3.21415 443420000.000 6.12 3.34 3.09 6.37 5.88

43349000.000 3.21418 443490000.000 6.12 3.34 3.09 6.37 5.88

43356000.000 3.21420 443560000.000 6.12 3.34 3.09 6.37 5.88

43363000.000 3.21423 443630000.000 6.12 3.34 3.09 6.37 5.88

43370000.000 3.21426 443700000.000 6.12 3.34 3.09 6.37 5.88

43377000.000 3.21428 443770000.000 6.12 3.34 3.09 6.37 5.88

43384000.000 3.21431 443840000.000 6.12 3.34 3.09 6.37 5.88

43391000.000 3.21433 443910000.000 6.12 3.34 3.09 6.37 5.88

43398000.000 3.21436 443980000.000 6.12 3.34 3.09 6.37 5.88

43405000.000 3.21439 444050000.000 6.12 3.34 3.09 6.37 5.88

43412000.000 3.21441 444120000.000 6.12 3.34 3.09 6.37 5.88

43419000.000 3.21444 444190000.000 6.12 3.34 3.09 6.37 5.88

43426000.000 3.21446 444260000.000 6.12 3.34 3.09 6.37 5.88

43433000.000 3.21449 444330000.000 6.12 3.34 3.09 6.37 5.88

43440000.000 3.21452 444400000.000 6.12 3.34 3.09 6.37 5.88

43447000.000 3.21454 444470000.000 6.12 3.34 3.09 6.37 5.88

43454000.000 3.21457 444540000.000 6.12 3.34 3.09 6.37 5.88

43461000.000 3.21459 444610000.000 6.12 3.34 3.09 6.37 5.88

43468000.000 3.21462 444680000.000 6.12 3.34 3.09 6.37 5.88

43475000.000 3.21465 444750000.000 6.12 3.34 3.09 6.37 5.88

43482000.000 3.21467 444820000.000 6.12 3.34 3.09 6.37 5.88

43489000.000 3.21470 444890000.000 6.12 3.34 3.09 6.37 5.88

43496000.000 3.21472 444960000.000 6.12 3.34 3.09 6.37 5.88

43503000.000 3.21475 445030000.000 6.12 3.34 3.09 6.37 5.88

43510000.000 3.21478 445100000.000 6.12 3.34 3.09 6.37 5.88

43517000.000 3.21480 445170000.000 6.12 3.34 3.09 6.37 5.88

43524000.000 3.21483 445240000.000 6.12 3.34 3.09 6.37 5.88

43531000.000 3.21485 445310000.000 6.12 3.34 3.09 6.37 5.88

43538000.000 3.21488 445380000.000 6.12 3.34 3.09 6.37 5.88

43545000.000 3.21491 445450000.000 6.12 3.34 3.09 6.37 5.88

43552000.000 3.21493 445520000.000 6.12 3.34 3.09 6.37 5.88

43559000.000 3.21496 445590000.000 6.12 3.34 3.09 6.37 5.88

43566000.000 3.21498 445660000.000 6.12 3.34 3.09 6.37 5.88

43573000.000 3.21501 445730000.000 6.12 3.34 3.09 6.37 5.88

43580000.000 3.21503 445800000.000 6.12 3.34 3.09 6.37 5.88

43587000.000 3.21506 445870000.000 6.12 3.34 3.09 6.37 5.88

43594000.000 3.21509 445940000.000 6.12 3.34 3.09 6.37 5.88

43601000.000 3.21511 446010000.000 6.12 3.34 3.09 6.37 5.88

43608000.000 3.21514 446080000.000 6.12 3.34 3.09 6.37 5.88

43615000.000 3.21516 446150000.000 6.12 3.34 3.09 6.37 5.88

43622000.000 3.21519 446220000.000 6.13 3.34 3.09 6.37 5.88

43629000.000 3.21522 446290000.000 6.13 3.34 3.09 6.37 5.88

43636000.000 3.21524 446360000.000 6.13 3.34 3.09 6.37 5.88

43643000.000 3.21527 446430000.000 6.13 3.34 3.09 6.37 5.88

43650000.000 3.21529 446500000.000 6.13 3.34 3.09 6.37 5.88

43657000.000 3.21532 446570000.000 6.13 3.34 3.09 6.37 5.88

43664000.000 3.21535 446640000.000 6.13 3.34 3.09 6.37 5.88

43671000.000 3.21537 446710000.000 6.13 3.34 3.09 6.37 5.88

43678000.000 3.21540 446780000.000 6.13 3.34 3.09 6.37 5.88

43685000.000 3.21542 446850000.000 6.13 3.34 3.09 6.37 5.88

43692000.000 3.21545 446920000.000 6.13 3.34 3.09 6.37 5.88

43699000.000 3.21547 446990000.000 6.13 3.34 3.09 6.37 5.88

43706000.000 3.21550 447060000.000 6.13 3.34 3.09 6.37 5.88

43713000.000 3.21553 447130000.000 6.13 3.34 3.09 6.37 5.88

43720000.000 3.21555 447200000.000 6.13 3.34 3.09 6.37 5.88

43727000.000 3.21558 447270000.000 6.13 3.34 3.09 6.37 5.88

43734000.000 3.21560 447340000.000 6.13 3.34 3.09 6.37 5.88

43741000.000 3.21563 447410000.000 6.13 3.34 3.09 6.37 5.88

43748000.000 3.21566 447480000.000 6.13 3.34 3.09 6.37 5.88

43755000.000 3.21568 447550000.000 6.13 3.34 3.09 6.37 5.88

43762000.000 3.21571 447620000.000 6.13 3.34 3.09 6.37 5.88

43769000.000 3.21573 447690000.000 6.13 3.34 3.09 6.37 5.88

43776000.000 3.21576 447760000.000 6.13 3.34 3.09 6.37 5.88

43783000.000 3.21578 447830000.000 6.13 3.34 3.09 6.37 5.88

43790000.000 3.21581 447900000.000 6.13 3.34 3.09 6.37 5.88

43797000.000 3.21584 447970000.000 6.13 3.34 3.09 6.37 5.88

43804000.000 3.21586 448040000.000 6.13 3.34 3.09 6.37 5.88

43811000.000 3.21589 448110000.000 6.13 3.34 3.09 6.37 5.88

43818000.000 3.21591 448180000.000 6.13 3.34 3.09 6.37 5.88

43825000.000 3.21594 448250000.000 6.13 3.34 3.09 6.37 5.88

43832000.000 3.21596 448320000.000 6.13 3.34 3.09 6.37 5.88

43839000.000 3.21599 448390000.000 6.13 3.34 3.09 6.37 5.88

43846000.000 3.21602 448460000.000 6.13 3.34 3.09 6.37 5.88

43853000.000 3.21604 448530000.000 6.13 3.34 3.09 6.37 5.88

43860000.000 3.21607 448600000.000 6.13 3.34 3.09 6.37 5.88

43867000.000 3.21609 448670000.000 6.13 3.34 3.09 6.37 5.88

43874000.000 3.21612 448740000.000 6.13 3.34 3.09 6.37 5.88

43881000.000 3.21615 448810000.000 6.13 3.34 3.09 6.37 5.88

43888000.000 3.21617 448880000.000 6.13 3.34 3.09 6.37 5.88

43895000.000 3.21620 448950000.000 6.13 3.34 3.09 6.37 5.88

43902000.000 3.21622 449020000.000 6.13 3.34 3.09 6.37 5.88

43909000.000 3.21625 449090000.000 6.13 3.34 3.09 6.37 5.88

43916000.000 3.21627 449160000.000 6.13 3.34 3.09 6.37 5.88

43923000.000 3.21630 449230000.000 6.13 3.34 3.09 6.37 5.88

43930000.000 3.21633 449300000.000 6.13 3.34 3.09 6.37 5.88

43937000.000 3.21635 449370000.000 6.13 3.35 3.09 6.37 5.88

43944000.000 3.21638 449440000.000 6.13 3.35 3.09 6.37 5.88

43951000.000 3.21640 449510000.000 6.13 3.35 3.09 6.37 5.88

43958000.000 3.21643 449580000.000 6.13 3.35 3.09 6.37 5.88

43965000.000 3.21645 449650000.000 6.13 3.35 3.09 6.37 5.88

43972000.000 3.21648 449720000.000 6.13 3.35 3.09 6.37 5.88

43979000.000 3.21651 449790000.000 6.13 3.35 3.09 6.37 5.88

43986000.000 3.21653 449860000.000 6.13 3.35 3.09 6.37 5.88

43993000.000 3.21656 449930000.000 6.13 3.35 3.09 6.37 5.88

44000000.000 3.21658 450000000.000 6.13 3.35 3.09 6.37 5.88

44007000.000 3.21661 450070000.000 6.13 3.35 3.09 6.37 5.88

44014000.000 3.21663 450140000.000 6.13 3.35 3.09 6.37 5.88

44021000.000 3.21666 450210000.000 6.13 3.35 3.09 6.37 5.88

44028000.000 3.21668 450280000.000 6.13 3.35 3.09 6.37 5.88

44035000.000 3.21671 450350000.000 6.13 3.35 3.09 6.37 5.88

44042000.000 3.21674 450420000.000 6.13 3.35 3.09 6.37 5.88

44049000.000 3.21676 450490000.000 6.13 3.35 3.09 6.37 5.88

44056000.000 3.21679 450560000.000 6.13 3.35 3.09 6.37 5.88

44063000.000 3.21681 450630000.000 6.13 3.35 3.09 6.37 5.88

44070000.000 3.21684 450700000.000 6.13 3.35 3.09 6.37 5.88

44077000.000 3.21686 450770000.000 6.13 3.35 3.09 6.37 5.88

44084000.000 3.21689 450840000.000 6.13 3.35 3.09 6.37 5.88

44091000.000 3.21692 450910000.000 6.13 3.35 3.09 6.37 5.88

44098000.000 3.21694 450980000.000 6.13 3.35 3.09 6.37 5.88

44105000.000 3.21697 451050000.000 6.13 3.35 3.09 6.37 5.88

44112000.000 3.21699 451120000.000 6.13 3.35 3.09 6.37 5.88

44119000.000 3.21702 451190000.000 6.13 3.35 3.09 6.37 5.88

44126000.000 3.21704 451260000.000 6.13 3.35 3.09 6.37 5.88

44133000.000 3.21707 451330000.000 6.13 3.35 3.09 6.37 5.88

44140000.000 3.21709 451400000.000 6.13 3.35 3.09 6.37 5.88

44147000.000 3.21712 451470000.000 6.13 3.35 3.09 6.37 5.88

44154000.000 3.21715 451540000.000 6.13 3.35 3.09 6.37 5.88

44161000.000 3.21717 451610000.000 6.13 3.35 3.09 6.37 5.88

44168000.000 3.21720 451680000.000 6.13 3.35 3.09 6.37 5.88

44175000.000 3.21722 451750000.000 6.13 3.35 3.09 6.37 5.88

44182000.000 3.21725 451820000.000 6.13 3.35 3.09 6.37 5.88

44189000.000 3.21727 451890000.000 6.13 3.35 3.09 6.37 5.88

44196000.000 3.21730 451960000.000 6.13 3.35 3.09 6.37 5.88

44203000.000 3.21732 452030000.000 6.13 3.35 3.09 6.37 5.88

44210000.000 3.21735 452100000.000 6.13 3.35 3.09 6.37 5.88

44217000.000 3.21738 452170000.000 6.13 3.35 3.09 6.37 5.88

44224000.000 3.21740 452240000.000 6.13 3.35 3.09 6.37 5.88

44231000.000 3.21743 452310000.000 6.13 3.35 3.09 6.37 5.88

44238000.000 3.21745 452380000.000 6.13 3.35 3.09 6.37 5.88

44245000.000 3.21748 452450000.000 6.13 3.35 3.09 6.37 5.88

44252000.000 3.21750 452520000.000 6.13 3.35 3.09 6.37 5.88

44259000.000 3.21753 452590000.000 6.13 3.35 3.09 6.37 5.88

44266000.000 3.21755 452660000.000 6.13 3.35 3.09 6.37 5.88

44273000.000 3.21758 452730000.000 6.13 3.35 3.09 6.37 5.88

44280000.000 3.21761 452800000.000 6.13 3.35 3.09 6.37 5.88

44287000.000 3.21763 452870000.000 6.13 3.35 3.09 6.37 5.88

44294000.000 3.21766 452940000.000 6.13 3.35 3.09 6.37 5.88

44301000.000 3.21768 453010000.000 6.13 3.35 3.09 6.37 5.88

44308000.000 3.21771 453080000.000 6.13 3.35 3.09 6.37 5.88

44315000.000 3.21773 453150000.000 6.13 3.35 3.09 6.37 5.88

44322000.000 3.21776 453220000.000 6.13 3.35 3.09 6.38 5.88

44329000.000 3.21778 453290000.000 6.13 3.35 3.09 6.38 5.88

44336000.000 3.21781 453360000.000 6.13 3.35 3.09 6.38 5.88

44343000.000 3.21784 453430000.000 6.13 3.35 3.09 6.38 5.88

44350000.000 3.21786 453500000.000 6.13 3.35 3.09 6.38 5.88

44357000.000 3.21789 453570000.000 6.13 3.35 3.09 6.38 5.88

44364000.000 3.21791 453640000.000 6.13 3.35 3.09 6.38 5.88

44371000.000 3.21794 453710000.000 6.13 3.35 3.09 6.38 5.88

44378000.000 3.21796 453780000.000 6.13 3.35 3.09 6.38 5.88

44385000.000 3.21799 453850000.000 6.13 3.35 3.09 6.38 5.89

44392000.000 3.21801 453920000.000 6.13 3.35 3.09 6.38 5.89

44399000.000 3.21804 453990000.000 6.13 3.35 3.09 6.38 5.89

44406000.000 3.21806 454060000.000 6.13 3.35 3.09 6.38 5.89

44413000.000 3.21809 454130000.000 6.13 3.35 3.09 6.38 5.89

44420000.000 3.21812 454200000.000 6.13 3.35 3.09 6.38 5.89

44427000.000 3.21814 454270000.000 6.13 3.35 3.09 6.38 5.89

44434000.000 3.21817 454340000.000 6.13 3.35 3.09 6.38 5.89

44441000.000 3.21819 454410000.000 6.13 3.35 3.09 6.38 5.89

44448000.000 3.21822 454480000.000 6.13 3.35 3.09 6.38 5.89

44455000.000 3.21824 454550000.000 6.13 3.35 3.09 6.38 5.89

44462000.000 3.21827 454620000.000 6.13 3.35 3.09 6.38 5.89

44469000.000 3.21829 454690000.000 6.13 3.35 3.09 6.38 5.89

44476000.000 3.21832 454760000.000 6.13 3.35 3.09 6.38 5.89

44483000.000 3.21834 454830000.000 6.13 3.35 3.09 6.38 5.89

44490000.000 3.21837 454900000.000 6.13 3.35 3.09 6.38 5.89

44497000.000 3.21839 454970000.000 6.13 3.35 3.09 6.38 5.89

44504000.000 3.21842 455040000.000 6.13 3.35 3.09 6.38 5.89

44511000.000 3.21845 455110000.000 6.13 3.35 3.09 6.38 5.89

44518000.000 3.21847 455180000.000 6.13 3.35 3.09 6.38 5.89

44525000.000 3.21850 455250000.000 6.13 3.35 3.09 6.38 5.89

44532000.000 3.21852 455320000.000 6.13 3.35 3.09 6.38 5.89

44539000.000 3.21855 455390000.000 6.13 3.35 3.09 6.38 5.89

44546000.000 3.21857 455460000.000 6.13 3.35 3.09 6.38 5.89

44553000.000 3.21860 455530000.000 6.13 3.35 3.09 6.38 5.89

44560000.000 3.21862 455600000.000 6.13 3.35 3.09 6.38 5.89

44567000.000 3.21865 455670000.000 6.13 3.35 3.09 6.38 5.89

44574000.000 3.21867 455740000.000 6.13 3.35 3.09 6.38 5.89

44581000.000 3.21870 455810000.000 6.13 3.35 3.09 6.38 5.89

44588000.000 3.21872 455880000.000 6.13 3.35 3.09 6.38 5.89

44595000.000 3.21875 455950000.000 6.13 3.35 3.09 6.38 5.89

44602000.000 3.21878 456020000.000 6.13 3.35 3.09 6.38 5.89

44609000.000 3.21880 456090000.000 6.13 3.35 3.09 6.38 5.89

44616000.000 3.21883 456160000.000 6.13 3.35 3.09 6.38 5.89

44623000.000 3.21885 456230000.000 6.13 3.35 3.09 6.38 5.89

44630000.000 3.21888 456300000.000 6.13 3.35 3.09 6.38 5.89

44637000.000 3.21890 456370000.000 6.13 3.35 3.09 6.38 5.89

44644000.000 3.21893 456440000.000 6.13 3.35 3.09 6.38 5.89

44651000.000 3.21895 456510000.000 6.13 3.35 3.09 6.38 5.89

44658000.000 3.21898 456580000.000 6.13 3.35 3.09 6.38 5.89

44665000.000 3.21900 456650000.000 6.13 3.35 3.09 6.38 5.89

44672000.000 3.21903 456720000.000 6.13 3.35 3.09 6.38 5.89

44679000.000 3.21905 456790000.000 6.13 3.35 3.09 6.38 5.89

44686000.000 3.21908 456860000.000 6.13 3.35 3.09 6.38 5.89

44693000.000 3.21910 456930000.000 6.13 3.35 3.09 6.38 5.89

44700000.000 3.21913 457000000.000 6.13 3.35 3.09 6.38 5.89

44707000.000 3.21915 457070000.000 6.13 3.35 3.09 6.38 5.89

44714000.000 3.21918 457140000.000 6.13 3.35 3.09 6.38 5.89

44721000.000 3.21921 457210000.000 6.13 3.35 3.09 6.38 5.89

44728000.000 3.21923 457280000.000 6.13 3.35 3.09 6.38 5.89

44735000.000 3.21926 457350000.000 6.13 3.35 3.09 6.38 5.89

44742000.000 3.21928 457420000.000 6.13 3.35 3.09 6.38 5.89

44749000.000 3.21931 457490000.000 6.13 3.35 3.09 6.38 5.89

44756000.000 3.21933 457560000.000 6.13 3.35 3.09 6.38 5.89

44763000.000 3.21936 457630000.000 6.13 3.35 3.09 6.38 5.89

44770000.000 3.21938 457700000.000 6.13 3.35 3.09 6.38 5.89

44777000.000 3.21941 457770000.000 6.13 3.35 3.09 6.38 5.89

44784000.000 3.21943 457840000.000 6.13 3.35 3.09 6.38 5.89

44791000.000 3.21946 457910000.000 6.13 3.35 3.09 6.38 5.89

44798000.000 3.21948 457980000.000 6.13 3.35 3.09 6.38 5.89

44805000.000 3.21951 458050000.000 6.13 3.35 3.09 6.38 5.89

44812000.000 3.21953 458120000.000 6.13 3.35 3.09 6.38 5.89

44819000.000 3.21956 458190000.000 6.13 3.35 3.09 6.38 5.89

44826000.000 3.21958 458260000.000 6.13 3.35 3.09 6.38 5.89

44833000.000 3.21961 458330000.000 6.13 3.35 3.09 6.38 5.89

44840000.000 3.21963 458400000.000 6.13 3.35 3.09 6.38 5.89

44847000.000 3.21966 458470000.000 6.13 3.35 3.09 6.38 5.89

44854000.000 3.21969 458540000.000 6.13 3.35 3.09 6.38 5.89

44861000.000 3.21971 458610000.000 6.13 3.35 3.09 6.38 5.89

44868000.000 3.21974 458680000.000 6.13 3.35 3.09 6.38 5.89

44875000.000 3.21976 458750000.000 6.13 3.35 3.09 6.38 5.89

44882000.000 3.21979 458820000.000 6.13 3.35 3.09 6.38 5.89

44889000.000 3.21981 458890000.000 6.13 3.35 3.09 6.38 5.89

44896000.000 3.21984 458960000.000 6.13 3.35 3.09 6.38 5.89

44903000.000 3.21986 459030000.000 6.13 3.35 3.09 6.38 5.89

44910000.000 3.21989 459100000.000 6.13 3.35 3.09 6.38 5.89

44917000.000 3.21991 459170000.000 6.13 3.35 3.09 6.38 5.89

44924000.000 3.21994 459240000.000 6.13 3.35 3.09 6.38 5.89

44931000.000 3.21996 459310000.000 6.13 3.35 3.09 6.38 5.89

44938000.000 3.21999 459380000.000 6.13 3.35 3.09 6.38 5.89

44945000.000 3.22001 459450000.000 6.13 3.35 3.09 6.38 5.89

44952000.000 3.22004 459520000.000 6.13 3.35 3.09 6.38 5.89

44959000.000 3.22006 459590000.000 6.13 3.35 3.09 6.38 5.89

44966000.000 3.22009 459660000.000 6.13 3.35 3.09 6.38 5.89

44973000.000 3.22011 459730000.000 6.13 3.35 3.09 6.38 5.89

44980000.000 3.22014 459800000.000 6.13 3.35 3.09 6.38 5.89

44987000.000 3.22016 459870000.000 6.13 3.35 3.09 6.38 5.89

44994000.000 3.22019 459940000.000 6.13 3.35 3.09 6.38 5.89

45001000.000 3.22021 460010000.000 6.13 3.35 3.09 6.38 5.89

45008000.000 3.22024 460080000.000 6.13 3.35 3.09 6.38 5.89

45015000.000 3.22026 460150000.000 6.13 3.35 3.09 6.38 5.89

45022000.000 3.22029 460220000.000 6.13 3.35 3.09 6.38 5.89

45029000.000 3.22031 460290000.000 6.13 3.35 3.09 6.38 5.89

45036000.000 3.22034 460360000.000 6.13 3.35 3.09 6.38 5.89

45043000.000 3.22036 460430000.000 6.13 3.35 3.09 6.38 5.89

45050000.000 3.22039 460500000.000 6.13 3.35 3.09 6.38 5.89

45057000.000 3.22041 460570000.000 6.13 3.35 3.09 6.38 5.89

45064000.000 3.22044 460640000.000 6.13 3.35 3.09 6.38 5.89

45071000.000 3.22046 460710000.000 6.13 3.35 3.09 6.38 5.89

45078000.000 3.22049 460780000.000 6.13 3.35 3.09 6.38 5.89

45085000.000 3.22051 460850000.000 6.13 3.35 3.09 6.38 5.89

45092000.000 3.22054 460920000.000 6.14 3.35 3.09 6.38 5.89

45099000.000 3.22056 460990000.000 6.14 3.35 3.09 6.38 5.89

45106000.000 3.22059 461060000.000 6.14 3.35 3.09 6.38 5.89

45113000.000 3.22062 461130000.000 6.14 3.35 3.09 6.38 5.89

45120000.000 3.22064 461200000.000 6.14 3.35 3.09 6.38 5.89

45127000.000 3.22067 461270000.000 6.14 3.35 3.09 6.38 5.89

45134000.000 3.22069 461340000.000 6.14 3.35 3.09 6.38 5.89

45141000.000 3.22072 461410000.000 6.14 3.35 3.09 6.38 5.89

45148000.000 3.22074 461480000.000 6.14 3.35 3.09 6.38 5.89

45155000.000 3.22077 461550000.000 6.14 3.35 3.09 6.38 5.89

45162000.000 3.22079 461620000.000 6.14 3.35 3.09 6.38 5.89

45169000.000 3.22082 461690000.000 6.14 3.35 3.09 6.38 5.89

45176000.000 3.22084 461760000.000 6.14 3.35 3.09 6.38 5.89

45183000.000 3.22087 461830000.000 6.14 3.35 3.09 6.38 5.89

45190000.000 3.22089 461900000.000 6.14 3.35 3.09 6.38 5.89

45197000.000 3.22092 461970000.000 6.14 3.35 3.09 6.38 5.89

45204000.000 3.22094 462040000.000 6.14 3.35 3.09 6.38 5.89

45211000.000 3.22097 462110000.000 6.14 3.35 3.09 6.38 5.89

45218000.000 3.22099 462180000.000 6.14 3.35 3.09 6.38 5.89

45225000.000 3.22102 462250000.000 6.14 3.35 3.09 6.38 5.89

45232000.000 3.22104 462320000.000 6.14 3.35 3.09 6.38 5.89

45239000.000 3.22107 462390000.000 6.14 3.35 3.09 6.38 5.89

45246000.000 3.22109 462460000.000 6.14 3.35 3.09 6.38 5.89

45253000.000 3.22112 462530000.000 6.14 3.35 3.09 6.38 5.89

45260000.000 3.22114 462600000.000 6.14 3.35 3.09 6.38 5.89

45267000.000 3.22117 462670000.000 6.14 3.35 3.09 6.38 5.89

45274000.000 3.22119 462740000.000 6.14 3.35 3.09 6.38 5.89

45281000.000 3.22122 462810000.000 6.14 3.35 3.09 6.38 5.89

45288000.000 3.22124 462880000.000 6.14 3.35 3.09 6.38 5.89

45295000.000 3.22127 462950000.000 6.14 3.35 3.09 6.38 5.89

45302000.000 3.22129 463020000.000 6.14 3.35 3.09 6.38 5.89

45309000.000 3.22132 463090000.000 6.14 3.35 3.09 6.38 5.89

45316000.000 3.22134 463160000.000 6.14 3.35 3.09 6.38 5.89

45323000.000 3.22137 463230000.000 6.14 3.35 3.09 6.38 5.89

45330000.000 3.22139 463300000.000 6.14 3.35 3.09 6.38 5.89

45337000.000 3.22142 463370000.000 6.14 3.35 3.09 6.38 5.89

45344000.000 3.22144 463440000.000 6.14 3.35 3.09 6.38 5.89

45351000.000 3.22147 463510000.000 6.14 3.35 3.09 6.38 5.89

45358000.000 3.22149 463580000.000 6.14 3.35 3.09 6.38 5.89

45365000.000 3.22152 463650000.000 6.14 3.35 3.09 6.38 5.89

45372000.000 3.22154 463720000.000 6.14 3.35 3.09 6.38 5.89

45379000.000 3.22156 463790000.000 6.14 3.35 3.09 6.38 5.89

45386000.000 3.22159 463860000.000 6.14 3.35 3.09 6.38 5.89

45393000.000 3.22161 463930000.000 6.14 3.35 3.09 6.38 5.89

45400000.000 3.22164 464000000.000 6.14 3.35 3.09 6.38 5.89

45407000.000 3.22166 464070000.000 6.14 3.35 3.09 6.38 5.89

45414000.000 3.22169 464140000.000 6.14 3.35 3.09 6.38 5.89

45421000.000 3.22171 464210000.000 6.14 3.35 3.09 6.38 5.89

45428000.000 3.22174 464280000.000 6.14 3.35 3.09 6.38 5.89

45435000.000 3.22176 464350000.000 6.14 3.35 3.09 6.38 5.89

45442000.000 3.22179 464420000.000 6.14 3.35 3.09 6.38 5.89

45449000.000 3.22181 464490000.000 6.14 3.35 3.09 6.38 5.89

45456000.000 3.22184 464560000.000 6.14 3.35 3.09 6.38 5.89

45463000.000 3.22186 464630000.000 6.14 3.35 3.09 6.38 5.89

45470000.000 3.22189 464700000.000 6.14 3.35 3.09 6.38 5.89

45477000.000 3.22191 464770000.000 6.14 3.35 3.09 6.38 5.89

45484000.000 3.22194 464840000.000 6.14 3.35 3.09 6.38 5.89

45491000.000 3.22196 464910000.000 6.14 3.35 3.09 6.38 5.89

45498000.000 3.22199 464980000.000 6.14 3.35 3.09 6.38 5.89

45505000.000 3.22201 465050000.000 6.14 3.35 3.09 6.38 5.89

45512000.000 3.22204 465120000.000 6.14 3.35 3.09 6.38 5.89

45519000.000 3.22206 465190000.000 6.14 3.35 3.09 6.38 5.89

45526000.000 3.22209 465260000.000 6.14 3.35 3.09 6.38 5.89

45533000.000 3.22211 465330000.000 6.14 3.35 3.09 6.38 5.89

45540000.000 3.22214 465400000.000 6.14 3.35 3.09 6.38 5.89

45547000.000 3.22216 465470000.000 6.14 3.35 3.09 6.38 5.89

45554000.000 3.22219 465540000.000 6.14 3.35 3.09 6.38 5.89

45561000.000 3.22221 465610000.000 6.14 3.35 3.09 6.38 5.89

45568000.000 3.22224 465680000.000 6.14 3.35 3.09 6.38 5.89

45575000.000 3.22226 465750000.000 6.14 3.35 3.09 6.38 5.89

45582000.000 3.22229 465820000.000 6.14 3.35 3.09 6.38 5.89

45589000.000 3.22231 465890000.000 6.14 3.35 3.09 6.38 5.89

45596000.000 3.22234 465960000.000 6.14 3.35 3.09 6.38 5.89

45603000.000 3.22236 466030000.000 6.14 3.35 3.09 6.38 5.89

45610000.000 3.22239 466100000.000 6.14 3.35 3.09 6.38 5.89

45617000.000 3.22241 466170000.000 6.14 3.35 3.09 6.38 5.89

45624000.000 3.22244 466240000.000 6.14 3.35 3.09 6.38 5.89

45631000.000 3.22246 466310000.000 6.14 3.35 3.09 6.38 5.89

45638000.000 3.22248 466380000.000 6.14 3.35 3.09 6.38 5.89

45645000.000 3.22251 466450000.000 6.14 3.35 3.09 6.38 5.89

45652000.000 3.22253 466520000.000 6.14 3.35 3.09 6.38 5.89

45659000.000 3.22256 466590000.000 6.14 3.35 3.09 6.38 5.89

45666000.000 3.22258 466660000.000 6.14 3.35 3.09 6.38 5.89

45673000.000 3.22261 466730000.000 6.14 3.35 3.09 6.38 5.89

45680000.000 3.22263 466800000.000 6.14 3.35 3.09 6.38 5.89

45687000.000 3.22266 466870000.000 6.14 3.35 3.09 6.38 5.89

45694000.000 3.22268 466940000.000 6.14 3.35 3.09 6.38 5.89

45701000.000 3.22271 467010000.000 6.14 3.35 3.09 6.38 5.89

45708000.000 3.22273 467080000.000 6.14 3.35 3.09 6.38 5.89

45715000.000 3.22276 467150000.000 6.14 3.35 3.09 6.38 5.89

45722000.000 3.22278 467220000.000 6.14 3.35 3.09 6.38 5.89

45729000.000 3.22281 467290000.000 6.14 3.35 3.09 6.38 5.89

45736000.000 3.22283 467360000.000 6.14 3.35 3.09 6.38 5.89

45743000.000 3.22286 467430000.000 6.14 3.35 3.09 6.38 5.89

45750000.000 3.22288 467500000.000 6.14 3.35 3.09 6.38 5.89

45757000.000 3.22291 467570000.000 6.14 3.35 3.09 6.38 5.89

45764000.000 3.22293 467640000.000 6.14 3.35 3.09 6.39 5.89

45771000.000 3.22296 467710000.000 6.14 3.35 3.09 6.39 5.89

45778000.000 3.22298 467780000.000 6.14 3.35 3.09 6.39 5.89

45785000.000 3.22300 467850000.000 6.14 3.35 3.09 6.39 5.89

45792000.000 3.22303 467920000.000 6.14 3.35 3.09 6.39 5.89

45799000.000 3.22305 467990000.000 6.14 3.35 3.09 6.39 5.89

45806000.000 3.22308 468060000.000 6.14 3.35 3.09 6.39 5.89

45813000.000 3.22310 468130000.000 6.14 3.35 3.09 6.39 5.89

45820000.000 3.22313 468200000.000 6.14 3.35 3.09 6.39 5.89

45827000.000 3.22315 468270000.000 6.14 3.35 3.09 6.39 5.89

45834000.000 3.22318 468340000.000 6.14 3.35 3.09 6.39 5.89

45841000.000 3.22320 468410000.000 6.14 3.35 3.09 6.39 5.89

45848000.000 3.22323 468480000.000 6.14 3.35 3.09 6.39 5.89

45855000.000 3.22325 468550000.000 6.14 3.35 3.09 6.39 5.89

45862000.000 3.22328 468620000.000 6.14 3.35 3.09 6.39 5.89

45869000.000 3.22330 468690000.000 6.14 3.35 3.09 6.39 5.89

45876000.000 3.22333 468760000.000 6.14 3.35 3.09 6.39 5.89

45883000.000 3.22335 468830000.000 6.14 3.35 3.09 6.39 5.89

45890000.000 3.22337 468900000.000 6.14 3.35 3.09 6.39 5.89

45897000.000 3.22340 468970000.000 6.14 3.35 3.09 6.39 5.89

45904000.000 3.22342 469040000.000 6.14 3.35 3.09 6.39 5.89

45911000.000 3.22345 469110000.000 6.14 3.35 3.09 6.39 5.89

45918000.000 3.22347 469180000.000 6.14 3.35 3.09 6.39 5.89

45925000.000 3.22350 469250000.000 6.14 3.35 3.09 6.39 5.89

45932000.000 3.22352 469320000.000 6.14 3.35 3.09 6.39 5.89

45939000.000 3.22355 469390000.000 6.14 3.35 3.09 6.39 5.89

45946000.000 3.22357 469460000.000 6.14 3.35 3.09 6.39 5.90

45953000.000 3.22360 469530000.000 6.14 3.35 3.09 6.39 5.90

45960000.000 3.22362 469600000.000 6.14 3.35 3.09 6.39 5.90

45967000.000 3.22365 469670000.000 6.14 3.35 3.09 6.39 5.90

45974000.000 3.22367 469740000.000 6.14 3.35 3.09 6.39 5.90

45981000.000 3.22370 469810000.000 6.14 3.35 3.09 6.39 5.90

45988000.000 3.22372 469880000.000 6.14 3.35 3.09 6.39 5.90

45995000.000 3.22374 469950000.000 6.14 3.35 3.09 6.39 5.90

46002000.000 3.22377 470020000.000 6.14 3.35 3.09 6.39 5.90

46009000.000 3.22379 470090000.000 6.14 3.35 3.09 6.39 5.90

46016000.000 3.22382 470160000.000 6.14 3.35 3.09 6.39 5.90

46023000.000 3.22384 470230000.000 6.14 3.35 3.09 6.39 5.90

46030000.000 3.22387 470300000.000 6.14 3.35 3.09 6.39 5.90

46037000.000 3.22389 470370000.000 6.14 3.35 3.09 6.39 5.90

46044000.000 3.22392 470440000.000 6.14 3.35 3.09 6.39 5.90

46051000.000 3.22394 470510000.000 6.14 3.35 3.09 6.39 5.90

46058000.000 3.22397 470580000.000 6.14 3.35 3.10 6.39 5.90

46065000.000 3.22399 470650000.000 6.14 3.35 3.10 6.39 5.90

46072000.000 3.22401 470720000.000 6.14 3.35 3.10 6.39 5.90

46079000.000 3.22404 470790000.000 6.14 3.35 3.10 6.39 5.90

46086000.000 3.22406 470860000.000 6.14 3.35 3.10 6.39 5.90

46093000.000 3.22409 470930000.000 6.14 3.35 3.10 6.39 5.90

46100000.000 3.22411 471000000.000 6.14 3.35 3.10 6.39 5.90

46107000.000 3.22414 471070000.000 6.14 3.35 3.10 6.39 5.90

46114000.000 3.22416 471140000.000 6.14 3.35 3.10 6.39 5.90

46121000.000 3.22419 471210000.000 6.14 3.35 3.10 6.39 5.90

46128000.000 3.22421 471280000.000 6.14 3.35 3.10 6.39 5.90

46135000.000 3.22424 471350000.000 6.14 3.35 3.10 6.39 5.90

46142000.000 3.22426 471420000.000 6.14 3.35 3.10 6.39 5.90

46149000.000 3.22429 471490000.000 6.14 3.35 3.10 6.39 5.90

46156000.000 3.22431 471560000.000 6.14 3.35 3.10 6.39 5.90

46163000.000 3.22433 471630000.000 6.14 3.35 3.10 6.39 5.90

46170000.000 3.22436 471700000.000 6.14 3.35 3.10 6.39 5.90

46177000.000 3.22438 471770000.000 6.14 3.35 3.10 6.39 5.90

46184000.000 3.22441 471840000.000 6.14 3.35 3.10 6.39 5.90

46191000.000 3.22443 471910000.000 6.14 3.35 3.10 6.39 5.90

46198000.000 3.22446 471980000.000 6.14 3.35 3.10 6.39 5.90

46205000.000 3.22448 472050000.000 6.14 3.35 3.10 6.39 5.90

46212000.000 3.22451 472120000.000 6.14 3.35 3.10 6.39 5.90

46219000.000 3.22453 472190000.000 6.14 3.35 3.10 6.39 5.90

46226000.000 3.22455 472260000.000 6.14 3.35 3.10 6.39 5.90

46233000.000 3.22458 472330000.000 6.14 3.35 3.10 6.39 5.90

46240000.000 3.22460 472400000.000 6.14 3.35 3.10 6.39 5.90

46247000.000 3.22463 472470000.000 6.14 3.35 3.10 6.39 5.90

46254000.000 3.22465 472540000.000 6.14 3.35 3.10 6.39 5.90

46261000.000 3.22468 472610000.000 6.14 3.35 3.10 6.39 5.90

46268000.000 3.22470 472680000.000 6.14 3.35 3.10 6.39 5.90

46275000.000 3.22473 472750000.000 6.14 3.35 3.10 6.39 5.90

46282000.000 3.22475 472820000.000 6.14 3.35 3.10 6.39 5.90

46289000.000 3.22477 472890000.000 6.14 3.35 3.10 6.39 5.90

46296000.000 3.22480 472960000.000 6.14 3.35 3.10 6.39 5.90

46303000.000 3.22482 473030000.000 6.14 3.35 3.10 6.39 5.90

46310000.000 3.22485 473100000.000 6.14 3.35 3.10 6.39 5.90

46317000.000 3.22487 473170000.000 6.14 3.35 3.10 6.39 5.90

46324000.000 3.22490 473240000.000 6.14 3.35 3.10 6.39 5.90

46331000.000 3.22492 473310000.000 6.14 3.35 3.10 6.39 5.90

46338000.000 3.22495 473380000.000 6.14 3.35 3.10 6.39 5.90

46345000.000 3.22497 473450000.000 6.14 3.35 3.10 6.39 5.90

46352000.000 3.22499 473520000.000 6.14 3.35 3.10 6.39 5.90

46359000.000 3.22502 473590000.000 6.14 3.35 3.10 6.39 5.90

46366000.000 3.22504 473660000.000 6.14 3.35 3.10 6.39 5.90

46373000.000 3.22507 473730000.000 6.14 3.35 3.10 6.39 5.90

46380000.000 3.22509 473800000.000 6.14 3.35 3.10 6.39 5.90

46387000.000 3.22512 473870000.000 6.14 3.35 3.10 6.39 5.90

46394000.000 3.22514 473940000.000 6.14 3.35 3.10 6.39 5.90

46401000.000 3.22517 474010000.000 6.14 3.35 3.10 6.39 5.90

46408000.000 3.22519 474080000.000 6.14 3.35 3.10 6.39 5.90

46415000.000 3.22521 474150000.000 6.14 3.35 3.10 6.39 5.90

46422000.000 3.22524 474220000.000 6.14 3.35 3.10 6.39 5.90

46429000.000 3.22526 474290000.000 6.14 3.35 3.10 6.39 5.90

46436000.000 3.22529 474360000.000 6.14 3.35 3.10 6.39 5.90

46443000.000 3.22531 474430000.000 6.14 3.35 3.10 6.39 5.90

46450000.000 3.22534 474500000.000 6.14 3.35 3.10 6.39 5.90

46457000.000 3.22536 474570000.000 6.14 3.35 3.10 6.39 5.90

46464000.000 3.22539 474640000.000 6.14 3.35 3.10 6.39 5.90

46471000.000 3.22541 474710000.000 6.14 3.35 3.10 6.39 5.90

46478000.000 3.22543 474780000.000 6.14 3.35 3.10 6.39 5.90

46485000.000 3.22546 474850000.000 6.14 3.35 3.10 6.39 5.90

46492000.000 3.22548 474920000.000 6.14 3.35 3.10 6.39 5.90

46499000.000 3.22551 474990000.000 6.14 3.35 3.10 6.39 5.90

46506000.000 3.22553 475060000.000 6.14 3.35 3.10 6.39 5.90

46513000.000 3.22556 475130000.000 6.14 3.35 3.10 6.39 5.90

46520000.000 3.22558 475200000.000 6.14 3.35 3.10 6.39 5.90

46527000.000 3.22560 475270000.000 6.14 3.35 3.10 6.39 5.90

46534000.000 3.22563 475340000.000 6.14 3.35 3.10 6.39 5.90

46541000.000 3.22565 475410000.000 6.14 3.35 3.10 6.39 5.90

46548000.000 3.22568 475480000.000 6.14 3.35 3.10 6.39 5.90

46555000.000 3.22570 475550000.000 6.14 3.35 3.10 6.39 5.90

46562000.000 3.22573 475620000.000 6.14 3.35 3.10 6.39 5.90

46569000.000 3.22575 475690000.000 6.14 3.35 3.10 6.39 5.90

46576000.000 3.22577 475760000.000 6.14 3.35 3.10 6.39 5.90

46583000.000 3.22580 475830000.000 6.14 3.35 3.10 6.39 5.90

46590000.000 3.22582 475900000.000 6.14 3.35 3.10 6.39 5.90

46597000.000 3.22585 475970000.000 6.14 3.35 3.10 6.39 5.90

46604000.000 3.22587 476040000.000 6.14 3.35 3.10 6.39 5.90

46611000.000 3.22590 476110000.000 6.14 3.35 3.10 6.39 5.90

46618000.000 3.22592 476180000.000 6.15 3.35 3.10 6.39 5.90

46625000.000 3.22595 476250000.000 6.15 3.35 3.10 6.39 5.90

46632000.000 3.22597 476320000.000 6.15 3.36 3.10 6.39 5.90

46639000.000 3.22599 476390000.000 6.15 3.36 3.10 6.39 5.90

46646000.000 3.22602 476460000.000 6.15 3.36 3.10 6.39 5.90

46653000.000 3.22604 476530000.000 6.15 3.36 3.10 6.39 5.90

46660000.000 3.22607 476600000.000 6.15 3.36 3.10 6.39 5.90

46667000.000 3.22609 476670000.000 6.15 3.36 3.10 6.39 5.90

46674000.000 3.22612 476740000.000 6.15 3.36 3.10 6.39 5.90

46681000.000 3.22614 476810000.000 6.15 3.36 3.10 6.39 5.90

46688000.000 3.22616 476880000.000 6.15 3.36 3.10 6.39 5.90

46695000.000 3.22619 476950000.000 6.15 3.36 3.10 6.39 5.90

46702000.000 3.22621 477020000.000 6.15 3.36 3.10 6.39 5.90

46709000.000 3.22624 477090000.000 6.15 3.36 3.10 6.39 5.90

46716000.000 3.22626 477160000.000 6.15 3.36 3.10 6.39 5.90

46723000.000 3.22628 477230000.000 6.15 3.36 3.10 6.39 5.90

46730000.000 3.22631 477300000.000 6.15 3.36 3.10 6.39 5.90

46737000.000 3.22633 477370000.000 6.15 3.36 3.10 6.39 5.90

46744000.000 3.22636 477440000.000 6.15 3.36 3.10 6.39 5.90

46751000.000 3.22638 477510000.000 6.15 3.36 3.10 6.39 5.90

46758000.000 3.22641 477580000.000 6.15 3.36 3.10 6.39 5.90

46765000.000 3.22643 477650000.000 6.15 3.36 3.10 6.39 5.90

46772000.000 3.22645 477720000.000 6.15 3.36 3.10 6.39 5.90

46779000.000 3.22648 477790000.000 6.15 3.36 3.10 6.39 5.90

46786000.000 3.22650 477860000.000 6.15 3.36 3.10 6.39 5.90

46793000.000 3.22653 477930000.000 6.15 3.36 3.10 6.39 5.90

46800000.000 3.22655 478000000.000 6.15 3.36 3.10 6.39 5.90

46807000.000 3.22658 478070000.000 6.15 3.36 3.10 6.39 5.90

46814000.000 3.22660 478140000.000 6.15 3.36 3.10 6.39 5.90

46821000.000 3.22662 478210000.000 6.15 3.36 3.10 6.39 5.90

46828000.000 3.22665 478280000.000 6.15 3.36 3.10 6.39 5.90

46835000.000 3.22667 478350000.000 6.15 3.36 3.10 6.39 5.90

46842000.000 3.22670 478420000.000 6.15 3.36 3.10 6.39 5.90

46849000.000 3.22672 478490000.000 6.15 3.36 3.10 6.39 5.90

46856000.000 3.22674 478560000.000 6.15 3.36 3.10 6.39 5.90

46863000.000 3.22677 478630000.000 6.15 3.36 3.10 6.39 5.90

46870000.000 3.22679 478700000.000 6.15 3.36 3.10 6.39 5.90

46877000.000 3.22682 478770000.000 6.15 3.36 3.10 6.39 5.90

46884000.000 3.22684 478840000.000 6.15 3.36 3.10 6.39 5.90

46891000.000 3.22687 478910000.000 6.15 3.36 3.10 6.39 5.90

46898000.000 3.22689 478980000.000 6.15 3.36 3.10 6.39 5.90

46905000.000 3.22691 479050000.000 6.15 3.36 3.10 6.39 5.90

46912000.000 3.22694 479120000.000 6.15 3.36 3.10 6.39 5.90

46919000.000 3.22696 479190000.000 6.15 3.36 3.10 6.39 5.90

46926000.000 3.22699 479260000.000 6.15 3.36 3.10 6.39 5.90

46933000.000 3.22701 479330000.000 6.15 3.36 3.10 6.39 5.90

46940000.000 3.22703 479400000.000 6.15 3.36 3.10 6.39 5.90

46947000.000 3.22706 479470000.000 6.15 3.36 3.10 6.39 5.90

46954000.000 3.22708 479540000.000 6.15 3.36 3.10 6.39 5.90

46961000.000 3.22711 479610000.000 6.15 3.36 3.10 6.39 5.90

46968000.000 3.22713 479680000.000 6.15 3.36 3.10 6.39 5.90

46975000.000 3.22716 479750000.000 6.15 3.36 3.10 6.39 5.90

46982000.000 3.22718 479820000.000 6.15 3.36 3.10 6.39 5.90

46989000.000 3.22720 479890000.000 6.15 3.36 3.10 6.39 5.90

46996000.000 3.22723 479960000.000 6.15 3.36 3.10 6.39 5.90

47003000.000 3.22725 480030000.000 6.15 3.36 3.10 6.39 5.90

47010000.000 3.22728 480100000.000 6.15 3.36 3.10 6.39 5.90

47017000.000 3.22730 480170000.000 6.15 3.36 3.10 6.39 5.90

47024000.000 3.22732 480240000.000 6.15 3.36 3.10 6.39 5.90

47031000.000 3.22735 480310000.000 6.15 3.36 3.10 6.39 5.90

47038000.000 3.22737 480380000.000 6.15 3.36 3.10 6.39 5.90

47045000.000 3.22740 480450000.000 6.15 3.36 3.10 6.39 5.90

47052000.000 3.22742 480520000.000 6.15 3.36 3.10 6.39 5.90

47059000.000 3.22744 480590000.000 6.15 3.36 3.10 6.39 5.90

47066000.000 3.22747 480660000.000 6.15 3.36 3.10 6.39 5.90

47073000.000 3.22749 480730000.000 6.15 3.36 3.10 6.39 5.90

47080000.000 3.22752 480800000.000 6.15 3.36 3.10 6.39 5.90

47087000.000 3.22754 480870000.000 6.15 3.36 3.10 6.39 5.90

47094000.000 3.22757 480940000.000 6.15 3.36 3.10 6.39 5.90

47101000.000 3.22759 481010000.000 6.15 3.36 3.10 6.39 5.90

47108000.000 3.22761 481080000.000 6.15 3.36 3.10 6.39 5.90

47115000.000 3.22764 481150000.000 6.15 3.36 3.10 6.39 5.90

47122000.000 3.22766 481220000.000 6.15 3.36 3.10 6.39 5.90

47129000.000 3.22769 481290000.000 6.15 3.36 3.10 6.39 5.90

47136000.000 3.22771 481360000.000 6.15 3.36 3.10 6.39 5.90

47143000.000 3.22773 481430000.000 6.15 3.36 3.10 6.39 5.90

47150000.000 3.22776 481500000.000 6.15 3.36 3.10 6.39 5.90

47157000.000 3.22778 481570000.000 6.15 3.36 3.10 6.39 5.90

47164000.000 3.22781 481640000.000 6.15 3.36 3.10 6.39 5.90

47171000.000 3.22783 481710000.000 6.15 3.36 3.10 6.39 5.90

47178000.000 3.22785 481780000.000 6.15 3.36 3.10 6.39 5.90

47185000.000 3.22788 481850000.000 6.15 3.36 3.10 6.39 5.90

47192000.000 3.22790 481920000.000 6.15 3.36 3.10 6.39 5.90

47199000.000 3.22793 481990000.000 6.15 3.36 3.10 6.39 5.90

47206000.000 3.22795 482060000.000 6.15 3.36 3.10 6.39 5.90

47213000.000 3.22797 482130000.000 6.15 3.36 3.10 6.39 5.90

47220000.000 3.22800 482200000.000 6.15 3.36 3.10 6.39 5.90

47227000.000 3.22802 482270000.000 6.15 3.36 3.10 6.39 5.90

47234000.000 3.22805 482340000.000 6.15 3.36 3.10 6.39 5.90

47241000.000 3.22807 482410000.000 6.15 3.36 3.10 6.40 5.90

47248000.000 3.22809 482480000.000 6.15 3.36 3.10 6.40 5.90

47255000.000 3.22812 482550000.000 6.15 3.36 3.10 6.40 5.90

47262000.000 3.22814 482620000.000 6.15 3.36 3.10 6.40 5.90

47269000.000 3.22817 482690000.000 6.15 3.36 3.10 6.40 5.90

47276000.000 3.22819 482760000.000 6.15 3.36 3.10 6.40 5.90

47283000.000 3.22821 482830000.000 6.15 3.36 3.10 6.40 5.90

47290000.000 3.22824 482900000.000 6.15 3.36 3.10 6.40 5.90

47297000.000 3.22826 482970000.000 6.15 3.36 3.10 6.40 5.90

47304000.000 3.22829 483040000.000 6.15 3.36 3.10 6.40 5.90

47311000.000 3.22831 483110000.000 6.15 3.36 3.10 6.40 5.90

47318000.000 3.22833 483180000.000 6.15 3.36 3.10 6.40 5.90

47325000.000 3.22836 483250000.000 6.15 3.36 3.10 6.40 5.90

47332000.000 3.22838 483320000.000 6.15 3.36 3.10 6.40 5.90

47339000.000 3.22841 483390000.000 6.15 3.36 3.10 6.40 5.90

47346000.000 3.22843 483460000.000 6.15 3.36 3.10 6.40 5.90

47353000.000 3.22845 483530000.000 6.15 3.36 3.10 6.40 5.90

47360000.000 3.22848 483600000.000 6.15 3.36 3.10 6.40 5.90

47367000.000 3.22850 483670000.000 6.15 3.36 3.10 6.40 5.90

47374000.000 3.22852 483740000.000 6.15 3.36 3.10 6.40 5.90

47381000.000 3.22855 483810000.000 6.15 3.36 3.10 6.40 5.90

47388000.000 3.22857 483880000.000 6.15 3.36 3.10 6.40 5.90

47395000.000 3.22860 483950000.000 6.15 3.36 3.10 6.40 5.90

47402000.000 3.22862 484020000.000 6.15 3.36 3.10 6.40 5.90

47409000.000 3.22864 484090000.000 6.15 3.36 3.10 6.40 5.90

47416000.000 3.22867 484160000.000 6.15 3.36 3.10 6.40 5.90

47423000.000 3.22869 484230000.000 6.15 3.36 3.10 6.40 5.90

47430000.000 3.22872 484300000.000 6.15 3.36 3.10 6.40 5.90

47437000.000 3.22874 484370000.000 6.15 3.36 3.10 6.40 5.90

47444000.000 3.22876 484440000.000 6.15 3.36 3.10 6.40 5.90

47451000.000 3.22879 484510000.000 6.15 3.36 3.10 6.40 5.90

47458000.000 3.22881 484580000.000 6.15 3.36 3.10 6.40 5.90

47465000.000 3.22884 484650000.000 6.15 3.36 3.10 6.40 5.90

47472000.000 3.22886 484720000.000 6.15 3.36 3.10 6.40 5.90

47479000.000 3.22888 484790000.000 6.15 3.36 3.10 6.40 5.90

47486000.000 3.22891 484860000.000 6.15 3.36 3.10 6.40 5.90

47493000.000 3.22893 484930000.000 6.15 3.36 3.10 6.40 5.90

47500000.000 3.22896 485000000.000 6.15 3.36 3.10 6.40 5.90

47507000.000 3.22898 485070000.000 6.15 3.36 3.10 6.40 5.90

47514000.000 3.22900 485140000.000 6.15 3.36 3.10 6.40 5.90

47521000.000 3.22903 485210000.000 6.15 3.36 3.10 6.40 5.90

47528000.000 3.22905 485280000.000 6.15 3.36 3.10 6.40 5.90

47535000.000 3.22907 485350000.000 6.15 3.36 3.10 6.40 5.90

47542000.000 3.22910 485420000.000 6.15 3.36 3.10 6.40 5.90

47549000.000 3.22912 485490000.000 6.15 3.36 3.10 6.40 5.90

47556000.000 3.22915 485560000.000 6.15 3.36 3.10 6.40 5.91

47563000.000 3.22917 485630000.000 6.15 3.36 3.10 6.40 5.91

47570000.000 3.22919 485700000.000 6.15 3.36 3.10 6.40 5.91

47577000.000 3.22922 485770000.000 6.15 3.36 3.10 6.40 5.91

47584000.000 3.22924 485840000.000 6.15 3.36 3.10 6.40 5.91

47591000.000 3.22927 485910000.000 6.15 3.36 3.10 6.40 5.91

47598000.000 3.22929 485980000.000 6.15 3.36 3.10 6.40 5.91

47605000.000 3.22931 486050000.000 6.15 3.36 3.10 6.40 5.91

47612000.000 3.22934 486120000.000 6.15 3.36 3.10 6.40 5.91

47619000.000 3.22936 486190000.000 6.15 3.36 3.10 6.40 5.91

47626000.000 3.22938 486260000.000 6.15 3.36 3.10 6.40 5.91

47633000.000 3.22941 486330000.000 6.15 3.36 3.10 6.40 5.91

47640000.000 3.22943 486400000.000 6.15 3.36 3.10 6.40 5.91

47647000.000 3.22946 486470000.000 6.15 3.36 3.10 6.40 5.91

47654000.000 3.22948 486540000.000 6.15 3.36 3.10 6.40 5.91

47661000.000 3.22950 486610000.000 6.15 3.36 3.10 6.40 5.91

47668000.000 3.22953 486680000.000 6.15 3.36 3.10 6.40 5.91

47675000.000 3.22955 486750000.000 6.15 3.36 3.10 6.40 5.91

47682000.000 3.22957 486820000.000 6.15 3.36 3.10 6.40 5.91

47689000.000 3.22960 486890000.000 6.15 3.36 3.10 6.40 5.91

47696000.000 3.22962 486960000.000 6.15 3.36 3.10 6.40 5.91

47703000.000 3.22965 487030000.000 6.15 3.36 3.10 6.40 5.91

47710000.000 3.22967 487100000.000 6.15 3.36 3.10 6.40 5.91

47717000.000 3.22969 487170000.000 6.15 3.36 3.10 6.40 5.91

47724000.000 3.22972 487240000.000 6.15 3.36 3.10 6.40 5.91

47731000.000 3.22974 487310000.000 6.15 3.36 3.10 6.40 5.91

47738000.000 3.22976 487380000.000 6.15 3.36 3.10 6.40 5.91

47745000.000 3.22979 487450000.000 6.15 3.36 3.10 6.40 5.91

47752000.000 3.22981 487520000.000 6.15 3.36 3.10 6.40 5.91

47759000.000 3.22984 487590000.000 6.15 3.36 3.10 6.40 5.91

47766000.000 3.22986 487660000.000 6.15 3.36 3.10 6.40 5.91

47773000.000 3.22988 487730000.000 6.15 3.36 3.10 6.40 5.91

47780000.000 3.22991 487800000.000 6.15 3.36 3.10 6.40 5.91

47787000.000 3.22993 487870000.000 6.15 3.36 3.10 6.40 5.91

47794000.000 3.22995 487940000.000 6.15 3.36 3.10 6.40 5.91

47801000.000 3.22998 488010000.000 6.15 3.36 3.10 6.40 5.91

47808000.000 3.23000 488080000.000 6.15 3.36 3.10 6.40 5.91

47815000.000 3.23003 488150000.000 6.15 3.36 3.10 6.40 5.91

47822000.000 3.23005 488220000.000 6.15 3.36 3.10 6.40 5.91

47829000.000 3.23007 488290000.000 6.15 3.36 3.10 6.40 5.91

47836000.000 3.23010 488360000.000 6.15 3.36 3.10 6.40 5.91

47843000.000 3.23012 488430000.000 6.15 3.36 3.10 6.40 5.91

47850000.000 3.23014 488500000.000 6.15 3.36 3.10 6.40 5.91

47857000.000 3.23017 488570000.000 6.15 3.36 3.10 6.40 5.91

47864000.000 3.23019 488640000.000 6.15 3.36 3.10 6.40 5.91

47871000.000 3.23022 488710000.000 6.15 3.36 3.10 6.40 5.91

47878000.000 3.23024 488780000.000 6.15 3.36 3.10 6.40 5.91

47885000.000 3.23026 488850000.000 6.15 3.36 3.10 6.40 5.91

47892000.000 3.23029 488920000.000 6.15 3.36 3.10 6.40 5.91

47899000.000 3.23031 488990000.000 6.15 3.36 3.10 6.40 5.91

47906000.000 3.23033 489060000.000 6.15 3.36 3.10 6.40 5.91

47913000.000 3.23036 489130000.000 6.15 3.36 3.10 6.40 5.91

47920000.000 3.23038 489200000.000 6.15 3.36 3.10 6.40 5.91

47927000.000 3.23040 489270000.000 6.15 3.36 3.10 6.40 5.91

47934000.000 3.23043 489340000.000 6.15 3.36 3.10 6.40 5.91

47941000.000 3.23045 489410000.000 6.15 3.36 3.10 6.40 5.91

47948000.000 3.23048 489480000.000 6.15 3.36 3.10 6.40 5.91

47955000.000 3.23050 489550000.000 6.15 3.36 3.10 6.40 5.91

47962000.000 3.23052 489620000.000 6.15 3.36 3.10 6.40 5.91

47969000.000 3.23055 489690000.000 6.15 3.36 3.10 6.40 5.91

47976000.000 3.23057 489760000.000 6.15 3.36 3.10 6.40 5.91

47983000.000 3.23059 489830000.000 6.15 3.36 3.10 6.40 5.91

47990000.000 3.23062 489900000.000 6.15 3.36 3.10 6.40 5.91

47997000.000 3.23064 489970000.000 6.15 3.36 3.10 6.40 5.91

48004000.000 3.23066 490040000.000 6.15 3.36 3.10 6.40 5.91

48011000.000 3.23069 490110000.000 6.15 3.36 3.10 6.40 5.91

48018000.000 3.23071 490180000.000 6.15 3.36 3.10 6.40 5.91

48025000.000 3.23074 490250000.000 6.15 3.36 3.10 6.40 5.91

48032000.000 3.23076 490320000.000 6.15 3.36 3.10 6.40 5.91

48039000.000 3.23078 490390000.000 6.15 3.36 3.10 6.40 5.91

48046000.000 3.23081 490460000.000 6.15 3.36 3.10 6.40 5.91

48053000.000 3.23083 490530000.000 6.15 3.36 3.10 6.40 5.91

48060000.000 3.23085 490600000.000 6.15 3.36 3.10 6.40 5.91

48067000.000 3.23088 490670000.000 6.15 3.36 3.10 6.40 5.91

48074000.000 3.23090 490740000.000 6.15 3.36 3.10 6.40 5.91

48081000.000 3.23092 490810000.000 6.15 3.36 3.10 6.40 5.91

48088000.000 3.23095 490880000.000 6.15 3.36 3.10 6.40 5.91

48095000.000 3.23097 490950000.000 6.15 3.36 3.10 6.40 5.91

48102000.000 3.23100 491020000.000 6.15 3.36 3.10 6.40 5.91
[truncated: 208,192 more chars]
