# Supplementary material for: Free convection heat transfer inside square water-filled shallow enclosures
Source: PLoS One. 2018 Oct 31;13(10):e0204251. doi: 10.1371/journal.pone.0204251 (PMC6209140; doi:10.1371/journal.pone.0204251)
Supplement: S4 Table — (DOCX) [file pone.0204251.s005.docx]

Data for the symbols in Fig.6.

| Square symbols | | Circular symbols | |
| --- | --- | --- | --- |
| Ra_H_ | Nu_L_ | Ra_H_ | Nu_L_ |
| 6892576.405 | 37.815 | 1379309.170 | 34.968 |
| 12738496.479 | 40.500 | 2746063.214 | 34.975 |
| 23152545.962 | 41.490 | 5183424.277 | 35.683 |
| 34817570.257 | 42.257 | 8116509.759 | 37.217 |
| 59814156.035 | 42.661 | 11628308.872 | 39.131 |
|  |  | 19298435.360 | 39.140 |

Data for the solid and dashed lines in Fig. 6

Ra_H_, solid Nu_L_, solid Ra_H_, dashed Nu_L_, dashed

2000000.000 26.05100 7000000.000 30.17

2007000.000 26.06992 7070000.000 30.23

2014000.000 26.08808 7140000.000 30.29

2021000.000 26.10619 7210000.000 30.35

2028000.000 26.12425 7280000.000 30.41

2035000.000 26.14226 7350000.000 30.47

2042000.000 26.16022 7420000.000 30.52

2049000.000 26.17813 7490000.000 30.58

2056000.000 26.19600 7560000.000 30.64

2063000.000 26.21381 7630000.000 30.69

2070000.000 26.23157 7700000.000 30.75

2077000.000 26.24929 7770000.000 30.81

2084000.000 26.26696 7840000.000 30.86

2091000.000 26.28458 7910000.000 30.92

2098000.000 26.30216 7980000.000 30.97

2105000.000 26.31969 8050000.000 31.03

2112000.000 26.33717 8120000.000 31.08

2119000.000 26.35460 8190000.000 31.13

2126000.000 26.37199 8260000.000 31.19

2133000.000 26.38934 8330000.000 31.24

2140000.000 26.40663 8400000.000 31.29

2147000.000 26.42389 8470000.000 31.34

2154000.000 26.44109 8540000.000 31.39

2161000.000 26.45826 8610000.000 31.45

2168000.000 26.47538 8680000.000 31.50

2175000.000 26.49245 8750000.000 31.55

2182000.000 26.50948 8820000.000 31.60

2189000.000 26.52647 8890000.000 31.65

2196000.000 26.54341 8960000.000 31.70

2203000.000 26.56031 9030000.000 31.75

2210000.000 26.57717 9100000.000 31.80

2217000.000 26.59398 9170000.000 31.84

2224000.000 26.61076 9240000.000 31.89

2231000.000 26.62749 9310000.000 31.94

2238000.000 26.64418 9380000.000 31.99

2245000.000 26.66082 9450000.000 32.04

2252000.000 26.67743 9520000.000 32.08

2259000.000 26.69399 9590000.000 32.13

2266000.000 26.71051 9660000.000 32.18

2273000.000 26.72700 9730000.000 32.22

2280000.000 26.74344 9800000.000 32.27

2287000.000 26.75984 9870000.000 32.32

2294000.000 26.77620 9940000.000 32.36

2301000.000 26.79252 10010000.000 32.41

2308000.000 26.80880 10080000.000 32.45

2315000.000 26.82505 10150000.000 32.50

2322000.000 26.84125 10220000.000 32.54

2329000.000 26.85741 10290000.000 32.59

2336000.000 26.87354 10360000.000 32.63

2343000.000 26.88962 10430000.000 32.67

2350000.000 26.90567 10500000.000 32.72

2357000.000 26.92168 10570000.000 32.76

2364000.000 26.93765 10640000.000 32.81

2371000.000 26.95359 10710000.000 32.85

2378000.000 26.96948 10780000.000 32.89

2385000.000 26.98534 10850000.000 32.93

2392000.000 27.00117 10920000.000 32.98

2399000.000 27.01695 10990000.000 33.02

2406000.000 27.03270 11060000.000 33.06

2413000.000 27.04841 11130000.000 33.10

2420000.000 27.06409 11200000.000 33.14

2427000.000 27.07972 11270000.000 33.19

2434000.000 27.09533 11340000.000 33.23

2441000.000 27.11089 11410000.000 33.27

2448000.000 27.12643 11480000.000 33.31

2455000.000 27.14192 11550000.000 33.35

2462000.000 27.15738 11620000.000 33.39

2469000.000 27.17281 11690000.000 33.43

2476000.000 27.18820 11760000.000 33.47

2483000.000 27.20355 11830000.000 33.51

2490000.000 27.21887 11900000.000 33.55

2497000.000 27.23416 11970000.000 33.59

2504000.000 27.24941 12040000.000 33.63

2511000.000 27.26463 12110000.000 33.67

2518000.000 27.27982 12180000.000 33.70

2525000.000 27.29497 12250000.000 33.74

2532000.000 27.31008 12320000.000 33.78

2539000.000 27.32517 12390000.000 33.82

2546000.000 27.34022 12460000.000 33.86

2553000.000 27.35523 12530000.000 33.90

2560000.000 27.37022 12600000.000 33.93

2567000.000 27.38517 12670000.000 33.97

2574000.000 27.40009 12740000.000 34.01

2581000.000 27.41498 12810000.000 34.05

2588000.000 27.42983 12880000.000 34.08

2595000.000 27.44465 12950000.000 34.12

2602000.000 27.45944 13020000.000 34.16

2609000.000 27.47420 13090000.000 34.19

2616000.000 27.48893 13160000.000 34.23

2623000.000 27.50363 13230000.000 34.27

2630000.000 27.51829 13300000.000 34.30

2637000.000 27.53292 13370000.000 34.34

2644000.000 27.54752 13440000.000 34.37

2651000.000 27.56210 13510000.000 34.41

2658000.000 27.57664 13580000.000 34.45

2665000.000 27.59114 13650000.000 34.48

2672000.000 27.60562 13720000.000 34.52

2679000.000 27.62007 13790000.000 34.55

2686000.000 27.63449 13860000.000 34.59

2693000.000 27.64888 13930000.000 34.62

2700000.000 27.66324 14000000.000 34.66

2707000.000 27.67757 14070000.000 34.69

2714000.000 27.69187 14140000.000 34.73

2721000.000 27.70614 14210000.000 34.76

2728000.000 27.72038 14280000.000 34.79

2735000.000 27.73459 14350000.000 34.83

2742000.000 27.74877 14420000.000 34.86

2749000.000 27.76293 14490000.000 34.90

2756000.000 27.77705 14560000.000 34.93

2763000.000 27.79115 14630000.000 34.96

2770000.000 27.80521 14700000.000 35.00

2777000.000 27.81925 14770000.000 35.03

2784000.000 27.83326 14840000.000 35.06

2791000.000 27.84725 14910000.000 35.10

2798000.000 27.86120 14980000.000 35.13

2805000.000 27.87513 15050000.000 35.16

2812000.000 27.88903 15120000.000 35.19

2819000.000 27.90290 15190000.000 35.23

2826000.000 27.91674 15260000.000 35.26

2833000.000 27.93056 15330000.000 35.29

2840000.000 27.94435 15400000.000 35.32

2847000.000 27.95811 15470000.000 35.36

2854000.000 27.97184 15540000.000 35.39

2861000.000 27.98555 15610000.000 35.42

2868000.000 27.99923 15680000.000 35.45

2875000.000 28.01289 15750000.000 35.48

2882000.000 28.02651 15820000.000 35.51

2889000.000 28.04011 15890000.000 35.55

2896000.000 28.05369 15960000.000 35.58

2903000.000 28.06724 16030000.000 35.61

2910000.000 28.08076 16100000.000 35.64

2917000.000 28.09426 16170000.000 35.67

2924000.000 28.10773 16240000.000 35.70

2931000.000 28.12117 16310000.000 35.73

2938000.000 28.13459 16380000.000 35.76

2945000.000 28.14799 16450000.000 35.79

2952000.000 28.16136 16520000.000 35.82

2959000.000 28.17470 16590000.000 35.85

2966000.000 28.18802 16660000.000 35.88

2973000.000 28.20131 16730000.000 35.91

2980000.000 28.21458 16800000.000 35.94

2987000.000 28.22782 16870000.000 35.97

2994000.000 28.24104 16940000.000 36.00

3001000.000 28.25423 17010000.000 36.03

3008000.000 28.26740 17080000.000 36.06

3015000.000 28.28054 17150000.000 36.09

3022000.000 28.29366 17220000.000 36.12

3029000.000 28.30676 17290000.000 36.15

3036000.000 28.31983 17360000.000 36.18

3043000.000 28.33288 17430000.000 36.21

3050000.000 28.34590 17500000.000 36.24

3057000.000 28.35890 17570000.000 36.27

3064000.000 28.37187 17640000.000 36.30

3071000.000 28.38483 17710000.000 36.32

3078000.000 28.39775 17780000.000 36.35

3085000.000 28.41066 17850000.000 36.38

3092000.000 28.42354 17920000.000 36.41

3099000.000 28.43640 17990000.000 36.44

3106000.000 28.44923 18060000.000 36.47

3113000.000 28.46205 18130000.000 36.50

3120000.000 28.47483 18200000.000 36.52

3127000.000 28.48760 18270000.000 36.55

3134000.000 28.50034 18340000.000 36.58

3141000.000 28.51306 18410000.000 36.61

3148000.000 28.52576 18480000.000 36.64

3155000.000 28.53844 18550000.000 36.66

3162000.000 28.55109 18620000.000 36.69

3169000.000 28.56372 18690000.000 36.72

3176000.000 28.57633 18760000.000 36.75

3183000.000 28.58891 18830000.000 36.77

3190000.000 28.60147 18900000.000 36.80

3197000.000 28.61402 18970000.000 36.83

3204000.000 28.62653 19040000.000 36.85

3211000.000 28.63903 19110000.000 36.88

3218000.000 28.65151 19180000.000 36.91

3225000.000 28.66396 19250000.000 36.94

3232000.000 28.67639 19320000.000 36.96

3239000.000 28.68881 19390000.000 36.99

3246000.000 28.70120 19460000.000 37.02

3253000.000 28.71356 19530000.000 37.04

3260000.000 28.72591 19600000.000 37.07

3267000.000 28.73824 19670000.000 37.10

3274000.000 28.75054 19740000.000 37.12

3281000.000 28.76282 19810000.000 37.15

3288000.000 28.77509 19880000.000 37.17

3295000.000 28.78733 19950000.000 37.20

3302000.000 28.79955 20020000.000 37.23

3309000.000 28.81175 20090000.000 37.25

3316000.000 28.82393 20160000.000 37.28

3323000.000 28.83609 20230000.000 37.30

3330000.000 28.84823 20300000.000 37.33

3337000.000 28.86035 20370000.000 37.36

3344000.000 28.87244 20440000.000 37.38

3351000.000 28.88452 20510000.000 37.41

3358000.000 28.89658 20580000.000 37.43

3365000.000 28.90862 20650000.000 37.46

3372000.000 28.92063 20720000.000 37.48

3379000.000 28.93263 20790000.000 37.51

3386000.000 28.94461 20860000.000 37.53

3393000.000 28.95657 20930000.000 37.56

3400000.000 28.96850 21000000.000 37.58

3407000.000 28.98042 21070000.000 37.61

3414000.000 28.99232 21140000.000 37.63

3421000.000 29.00420 21210000.000 37.66

3428000.000 29.01606 21280000.000 37.68

3435000.000 29.02790 21350000.000 37.71

3442000.000 29.03972 21420000.000 37.73

3449000.000 29.05152 21490000.000 37.76

3456000.000 29.06331 21560000.000 37.78

3463000.000 29.07507 21630000.000 37.81

3470000.000 29.08682 21700000.000 37.83

3477000.000 29.09854 21770000.000 37.86

3484000.000 29.11025 21840000.000 37.88

3491000.000 29.12194 21910000.000 37.90

3498000.000 29.13361 21980000.000 37.93

3505000.000 29.14526 22050000.000 37.95

3512000.000 29.15689 22120000.000 37.98

3519000.000 29.16850 22190000.000 38.00

3526000.000 29.18010 22260000.000 38.02

3533000.000 29.19167 22330000.000 38.05

3540000.000 29.20323 22400000.000 38.07

3547000.000 29.21477 22470000.000 38.10

3554000.000 29.22630 22540000.000 38.12

3561000.000 29.23780 22610000.000 38.14

3568000.000 29.24928 22680000.000 38.17

3575000.000 29.26075 22750000.000 38.19

3582000.000 29.27220 22820000.000 38.21

3589000.000 29.28363 22890000.000 38.24

3596000.000 29.29505 22960000.000 38.26

3603000.000 29.30644 23030000.000 38.28

3610000.000 29.31782 23100000.000 38.31

3617000.000 29.32918 23170000.000 38.33

3624000.000 29.34053 23240000.000 38.35

3631000.000 29.35185 23310000.000 38.38

3638000.000 29.36316 23380000.000 38.40

3645000.000 29.37445 23450000.000 38.42

3652000.000 29.38573 23520000.000 38.45

3659000.000 29.39698 23590000.000 38.47

3666000.000 29.40822 23660000.000 38.49

3673000.000 29.41944 23730000.000 38.51

3680000.000 29.43065 23800000.000 38.54

3687000.000 29.44184 23870000.000 38.56

3694000.000 29.45301 23940000.000 38.58

3701000.000 29.46416 24010000.000 38.60

3708000.000 29.47530 24080000.000 38.63

3715000.000 29.48642 24150000.000 38.65

3722000.000 29.49752 24220000.000 38.67

3729000.000 29.50861 24290000.000 38.69

3736000.000 29.51968 24360000.000 38.72

3743000.000 29.53073 24430000.000 38.74

3750000.000 29.54177 24500000.000 38.76

3757000.000 29.55279 24570000.000 38.78

3764000.000 29.56380 24640000.000 38.80

3771000.000 29.57478 24710000.000 38.83

3778000.000 29.58576 24780000.000 38.85

3785000.000 29.59671 24850000.000 38.87

3792000.000 29.60765 24920000.000 38.89

3799000.000 29.61857 24990000.000 38.91

3806000.000 29.62948 25060000.000 38.94

3813000.000 29.64037 25130000.000 38.96

3820000.000 29.65125 25200000.000 38.98

3827000.000 29.66211 25270000.000 39.00

3834000.000 29.67295 25340000.000 39.02

3841000.000 29.68378 25410000.000 39.04

3848000.000 29.69459 25480000.000 39.07

3855000.000 29.70538 25550000.000 39.09

3862000.000 29.71616 25620000.000 39.11

3869000.000 29.72693 25690000.000 39.13

3876000.000 29.73768 25760000.000 39.15

3883000.000 29.74841 25830000.000 39.17

3890000.000 29.75913 25900000.000 39.19

3897000.000 29.76983 25970000.000 39.22

3904000.000 29.78052 26040000.000 39.24

3911000.000 29.79119 26110000.000 39.26

3918000.000 29.80185 26180000.000 39.28

3925000.000 29.81249 26250000.000 39.30

3932000.000 29.82311 26320000.000 39.32

3939000.000 29.83372 26390000.000 39.34

3946000.000 29.84432 26460000.000 39.36

3953000.000 29.85490 26530000.000 39.38

3960000.000 29.86547 26600000.000 39.40

3967000.000 29.87602 26670000.000 39.42

3974000.000 29.88655 26740000.000 39.44

3981000.000 29.89708 26810000.000 39.47

3988000.000 29.90758 26880000.000 39.49

3995000.000 29.91807 26950000.000 39.51

4002000.000 29.92855 27020000.000 39.53

4009000.000 29.93901 27090000.000 39.55

4016000.000 29.94946 27160000.000 39.57

4023000.000 29.95990 27230000.000 39.59

4030000.000 29.97031 27300000.000 39.61

4037000.000 29.98072 27370000.000 39.63

4044000.000 29.99111 27440000.000 39.65

4051000.000 30.00148 27510000.000 39.67

4058000.000 30.01184 27580000.000 39.69

4065000.000 30.02219 27650000.000 39.71

4072000.000 30.03252 27720000.000 39.73

4079000.000 30.04284 27790000.000 39.75

4086000.000 30.05315 27860000.000 39.77

4093000.000 30.06344 27930000.000 39.79

4100000.000 30.07371 28000000.000 39.81

4107000.000 30.08398 28070000.000 39.83

4114000.000 30.09422 28140000.000 39.85

4121000.000 30.10446 28210000.000 39.87

4128000.000 30.11468 28280000.000 39.89

4135000.000 30.12488 28350000.000 39.91

4142000.000 30.13508 28420000.000 39.93

4149000.000 30.14526 28490000.000 39.95

4156000.000 30.15542 28560000.000 39.97

4163000.000 30.16557 28630000.000 39.99

4170000.000 30.17571 28700000.000 40.01

4177000.000 30.18583 28770000.000 40.03

4184000.000 30.19594 28840000.000 40.05

4191000.000 30.20604 28910000.000 40.07

4198000.000 30.21612 28980000.000 40.08

4205000.000 30.22620 29050000.000 40.10

4212000.000 30.23625 29120000.000 40.12

4219000.000 30.24630 29190000.000 40.14

4226000.000 30.25633 29260000.000 40.16

4233000.000 30.26634 29330000.000 40.18

4240000.000 30.27635 29400000.000 40.20

4247000.000 30.28634 29470000.000 40.22

4254000.000 30.29631 29540000.000 40.24

4261000.000 30.30628 29610000.000 40.26

4268000.000 30.31623 29680000.000 40.28

4275000.000 30.32617 29750000.000 40.30

4282000.000 30.33609 29820000.000 40.31

4289000.000 30.34600 29890000.000 40.33

4296000.000 30.35590 29960000.000 40.35

4303000.000 30.36579 30030000.000 40.37

4310000.000 30.37566 30100000.000 40.39

4317000.000 30.38552 30170000.000 40.41

4324000.000 30.39537 30240000.000 40.43

4331000.000 30.40520 30310000.000 40.45

4338000.000 30.41503 30380000.000 40.46

4345000.000 30.42484 30450000.000 40.48

4352000.000 30.43463 30520000.000 40.50

4359000.000 30.44442 30590000.000 40.52

4366000.000 30.45419 30660000.000 40.54

4373000.000 30.46395 30730000.000 40.56

4380000.000 30.47369 30800000.000 40.58

4387000.000 30.48343 30870000.000 40.59

4394000.000 30.49315 30940000.000 40.61

4401000.000 30.50286 31010000.000 40.63

4408000.000 30.51256 31080000.000 40.65

4415000.000 30.52224 31150000.000 40.67

4422000.000 30.53191 31220000.000 40.69

4429000.000 30.54157 31290000.000 40.70

4436000.000 30.55122 31360000.000 40.72

4443000.000 30.56086 31430000.000 40.74

4450000.000 30.57048 31500000.000 40.76

4457000.000 30.58009 31570000.000 40.78

4464000.000 30.58969 31640000.000 40.79

4471000.000 30.59928 31710000.000 40.81

4478000.000 30.60886 31780000.000 40.83

4485000.000 30.61842 31850000.000 40.85

4492000.000 30.62797 31920000.000 40.87

4499000.000 30.63751 31990000.000 40.88

4506000.000 30.64704 32060000.000 40.90

4513000.000 30.65655 32130000.000 40.92

4520000.000 30.66606 32200000.000 40.94

4527000.000 30.67555 32270000.000 40.96

4534000.000 30.68503 32340000.000 40.97

4541000.000 30.69450 32410000.000 40.99

4548000.000 30.70396 32480000.000 41.01

4555000.000 30.71340 32550000.000 41.03

4562000.000 30.72284 32620000.000 41.04

4569000.000 30.73226 32690000.000 41.06

4576000.000 30.74167 32760000.000 41.08

4583000.000 30.75107 32830000.000 41.10

4590000.000 30.76046 32900000.000 41.11

4597000.000 30.76984 32970000.000 41.13

4604000.000 30.77920 33040000.000 41.15

4611000.000 30.78855 33110000.000 41.17

4618000.000 30.79790 33180000.000 41.18

4625000.000 30.80723 33250000.000 41.20

4632000.000 30.81655 33320000.000 41.22

4639000.000 30.82586 33390000.000 41.24

4646000.000 30.83515 33460000.000 41.25

4653000.000 30.84444 33530000.000 41.27

4660000.000 30.85372 33600000.000 41.29

4667000.000 30.86298 33670000.000 41.31

4674000.000 30.87223 33740000.000 41.32

4681000.000 30.88147 33810000.000 41.34

4688000.000 30.89070 33880000.000 41.36

4695000.000 30.89992 33950000.000 41.37

4702000.000 30.90913 34020000.000 41.39

4709000.000 30.91833 34090000.000 41.41

4716000.000 30.92752 34160000.000 41.42

4723000.000 30.93669 34230000.000 41.44

4730000.000 30.94586 34300000.000 41.46

4737000.000 30.95501 34370000.000 41.48

4744000.000 30.96415 34440000.000 41.49

4751000.000 30.97329 34510000.000 41.51

4758000.000 30.98241 34580000.000 41.53

4765000.000 30.99152 34650000.000 41.54

4772000.000 31.00062 34720000.000 41.56

4779000.000 31.00971 34790000.000 41.58

4786000.000 31.01879 34860000.000 41.59

4793000.000 31.02786 34930000.000 41.61

4800000.000 31.03691 35000000.000 41.63

4807000.000 31.04596 35070000.000 41.64

4814000.000 31.05500 35140000.000 41.66

4821000.000 31.06402 35210000.000 41.68

4828000.000 31.07304 35280000.000 41.69

4835000.000 31.08204 35350000.000 41.71

4842000.000 31.09104 35420000.000 41.73

4849000.000 31.10002 35490000.000 41.74

4856000.000 31.10900 35560000.000 41.76

4863000.000 31.11796 35630000.000 41.78

4870000.000 31.12691 35700000.000 41.79

4877000.000 31.13586 35770000.000 41.81

4884000.000 31.14479 35840000.000 41.82

4891000.000 31.15371 35910000.000 41.84

4898000.000 31.16263 35980000.000 41.86

4905000.000 31.17153 36050000.000 41.87

4912000.000 31.18042 36120000.000 41.89

4919000.000 31.18930 36190000.000 41.91

4926000.000 31.19817 36260000.000 41.92

4933000.000 31.20703 36330000.000 41.94

4940000.000 31.21589 36400000.000 41.95

4947000.000 31.22473 36470000.000 41.97

4954000.000 31.23356 36540000.000 41.99

4961000.000 31.24238 36610000.000 42.00

4968000.000 31.25119 36680000.000 42.02

4975000.000 31.25999 36750000.000 42.03

4982000.000 31.26879 36820000.000 42.05

4989000.000 31.27757 36890000.000 42.07

4996000.000 31.28634 36960000.000 42.08

5003000.000 31.29510 37030000.000 42.10

5010000.000 31.30386 37100000.000 42.11

5017000.000 31.31260 37170000.000 42.13

5024000.000 31.32133 37240000.000 42.15

5031000.000 31.33005 37310000.000 42.16

5038000.000 31.33877 37380000.000 42.18

5045000.000 31.34747 37450000.000 42.19

5052000.000 31.35617 37520000.000 42.21

5059000.000 31.36485 37590000.000 42.23

5066000.000 31.37353 37660000.000 42.24

5073000.000 31.38219 37730000.000 42.26

5080000.000 31.39085 37800000.000 42.27

5087000.000 31.39949 37870000.000 42.29

5094000.000 31.40813 37940000.000 42.30

5101000.000 31.41676 38010000.000 42.32

5108000.000 31.42537 38080000.000 42.33

5115000.000 31.43398 38150000.000 42.35

5122000.000 31.44258 38220000.000 42.37

5129000.000 31.45117 38290000.000 42.38

5136000.000 31.45975 38360000.000 42.40

5143000.000 31.46832 38430000.000 42.41

5150000.000 31.47688 38500000.000 42.43

5157000.000 31.48544 38570000.000 42.44

5164000.000 31.49398 38640000.000 42.46

5171000.000 31.50251 38710000.000 42.47

5178000.000 31.51104 38780000.000 42.49

5185000.000 31.51955 38850000.000 42.50

5192000.000 31.52806 38920000.000 42.52

5199000.000 31.53655 38990000.000 42.54

5206000.000 31.54504 39060000.000 42.55

5213000.000 31.55352 39130000.000 42.57

5220000.000 31.56199 39200000.000 42.58

5227000.000 31.57045 39270000.000 42.60

5234000.000 31.57890 39340000.000 42.61

5241000.000 31.58734 39410000.000 42.63

5248000.000 31.59578 39480000.000 42.64

5255000.000 31.60420 39550000.000 42.66

5262000.000 31.61262 39620000.000 42.67

5269000.000 31.62102 39690000.000 42.69

5276000.000 31.62942 39760000.000 42.70

5283000.000 31.63781 39830000.000 42.72

5290000.000 31.64619 39900000.000 42.73

5297000.000 31.65456 39970000.000 42.75

5304000.000 31.66292 40040000.000 42.76

5311000.000 31.67127 40110000.000 42.78

5318000.000 31.67962 40180000.000 42.79

5325000.000 31.68795 40250000.000 42.81

5332000.000 31.69628 40320000.000 42.82

5339000.000 31.70460 40390000.000 42.84

5346000.000 31.71291 40460000.000 42.85

5353000.000 31.72121 40530000.000 42.87

5360000.000 31.72950 40600000.000 42.88

5367000.000 31.73778 40670000.000 42.90

5374000.000 31.74606 40740000.000 42.91

5381000.000 31.75432 40810000.000 42.93

5388000.000 31.76258 40880000.000 42.94

5395000.000 31.77083 40950000.000 42.95

5402000.000 31.77907 41020000.000 42.97

5409000.000 31.78730 41090000.000 42.98

5416000.000 31.79553 41160000.000 43.00

5423000.000 31.80374 41230000.000 43.01

5430000.000 31.81195 41300000.000 43.03

5437000.000 31.82014 41370000.000 43.04

5444000.000 31.82833 41440000.000 43.06

5451000.000 31.83651 41510000.000 43.07

5458000.000 31.84469 41580000.000 43.09

5465000.000 31.85285 41650000.000 43.10

5472000.000 31.86101 41720000.000 43.11

5479000.000 31.86915 41790000.000 43.13

5486000.000 31.87729 41860000.000 43.14

5493000.000 31.88542 41930000.000 43.16

5500000.000 31.89355 42000000.000 43.17

5507000.000 31.90166 42070000.000 43.19

5514000.000 31.90977 42140000.000 43.20

5521000.000 31.91786 42210000.000 43.22

5528000.000 31.92595 42280000.000 43.23

5535000.000 31.93404 42350000.000 43.24

5542000.000 31.94211 42420000.000 43.26

5549000.000 31.95017 42490000.000 43.27

5556000.000 31.95823 42560000.000 43.29

5563000.000 31.96628 42630000.000 43.30

5570000.000 31.97432 42700000.000 43.32

5577000.000 31.98235 42770000.000 43.33

5584000.000 31.99038 42840000.000 43.34

5591000.000 31.99839 42910000.000 43.36

5598000.000 32.00640 42980000.000 43.37

5605000.000 32.01440 43050000.000 43.39

5612000.000 32.02240 43120000.000 43.40

5619000.000 32.03038 43190000.000 43.41

5626000.000 32.03836 43260000.000 43.43

5633000.000 32.04632 43330000.000 43.44

5640000.000 32.05429 43400000.000 43.46

5647000.000 32.06224 43470000.000 43.47

5654000.000 32.07018 43540000.000 43.48

5661000.000 32.07812 43610000.000 43.50

5668000.000 32.08605 43680000.000 43.51

5675000.000 32.09397 43750000.000 43.53

5682000.000 32.10188 43820000.000 43.54

5689000.000 32.10979 43890000.000 43.55

5696000.000 32.11769 43960000.000 43.57

5703000.000 32.12558 44030000.000 43.58

5710000.000 32.13346 44100000.000 43.60

5717000.000 32.14134 44170000.000 43.61

5724000.000 32.14920 44240000.000 43.62

5731000.000 32.15706 44310000.000 43.64

5738000.000 32.16491 44380000.000 43.65

5745000.000 32.17276 44450000.000 43.66

5752000.000 32.18059 44520000.000 43.68

5759000.000 32.18842 44590000.000 43.69

5766000.000 32.19624 44660000.000 43.71

5773000.000 32.20406 44730000.000 43.72

5780000.000 32.21186 44800000.000 43.73

5787000.000 32.21966 44870000.000 43.75

5794000.000 32.22745 44940000.000 43.76

5801000.000 32.23524 45010000.000 43.77

5808000.000 32.24301 45080000.000 43.79

5815000.000 32.25078 45150000.000 43.80

5822000.000 32.25854 45220000.000 43.82

5829000.000 32.26629 45290000.000 43.83

5836000.000 32.27404 45360000.000 43.84

5843000.000 32.28178 45430000.000 43.86

5850000.000 32.28951 45500000.000 43.87

5857000.000 32.29723 45570000.000 43.88

5864000.000 32.30495 45640000.000 43.90

5871000.000 32.31266 45710000.000 43.91

5878000.000 32.32036 45780000.000 43.92

5885000.000 32.32805 45850000.000 43.94

5892000.000 32.33574 45920000.000 43.95

5899000.000 32.34342 45990000.000 43.96

5906000.000 32.35109 46060000.000 43.98

5913000.000 32.35876 46130000.000 43.99

5920000.000 32.36642 46200000.000 44.00

5927000.000 32.37407 46270000.000 44.02

5934000.000 32.38171 46340000.000 44.03

5941000.000 32.38935 46410000.000 44.04

5948000.000 32.39698 46480000.000 44.06

5955000.000 32.40460 46550000.000 44.07

5962000.000 32.41221 46620000.000 44.08

5969000.000 32.41982 46690000.000 44.10

5976000.000 32.42742 46760000.000 44.11

5983000.000 32.43501 46830000.000 44.12

5990000.000 32.44260 46900000.000 44.14

5997000.000 32.45018 46970000.000 44.15

6004000.000 32.45775 47040000.000 44.16

6011000.000 32.46532 47110000.000 44.18

6018000.000 32.47287 47180000.000 44.19

6025000.000 32.48042 47250000.000 44.20

6032000.000 32.48797 47320000.000 44.21

6039000.000 32.49550 47390000.000 44.23

6046000.000 32.50303 47460000.000 44.24

6053000.000 32.51056 47530000.000 44.25

6060000.000 32.51807 47600000.000 44.27

6067000.000 32.52558 47670000.000 44.28

6074000.000 32.53308 47740000.000 44.29

6081000.000 32.54058 47810000.000 44.31

6088000.000 32.54807 47880000.000 44.32

6095000.000 32.55555 47950000.000 44.33

6102000.000 32.56302 48020000.000 44.34

6109000.000 32.57049 48090000.000 44.36

6116000.000 32.57795 48160000.000 44.37

6123000.000 32.58541 48230000.000 44.38

6130000.000 32.59285 48300000.000 44.40

6137000.000 32.60029 48370000.000 44.41

6144000.000 32.60773 48440000.000 44.42

6151000.000 32.61515 48510000.000 44.43

6158000.000 32.62257 48580000.000 44.45

6165000.000 32.62999 48650000.000 44.46

6172000.000 32.63739 48720000.000 44.47

6179000.000 32.64479 48790000.000 44.49

6186000.000 32.65219 48860000.000 44.50

6193000.000 32.65957 48930000.000 44.51

6200000.000 32.66695 49000000.000 44.52

6207000.000 32.67433 49070000.000 44.54

6214000.000 32.68169 49140000.000 44.55

6221000.000 32.68905 49210000.000 44.56

6228000.000 32.69640 49280000.000 44.58

6235000.000 32.70375 49350000.000 44.59

6242000.000 32.71109 49420000.000 44.60

6249000.000 32.71842 49490000.000 44.61

6256000.000 32.72575 49560000.000 44.63

6263000.000 32.73307 49630000.000 44.64

6270000.000 32.74039 49700000.000 44.65

6277000.000 32.74769 49770000.000 44.66

6284000.000 32.75499 49840000.000 44.68

6291000.000 32.76229 49910000.000 44.69

6298000.000 32.76958 49980000.000 44.70

6305000.000 32.77686 50050000.000 44.71

6312000.000 32.78413 50120000.000 44.73

6319000.000 32.79140 50190000.000 44.74

6326000.000 32.79866 50260000.000 44.75

6333000.000 32.80592 50330000.000 44.76

6340000.000 32.81317 50400000.000 44.78

6347000.000 32.82041 50470000.000 44.79

6354000.000 32.82764 50540000.000 44.80

6361000.000 32.83487 50610000.000 44.81

6368000.000 32.84210 50680000.000 44.83

6375000.000 32.84932 50750000.000 44.84

6382000.000 32.85653 50820000.000 44.85

6389000.000 32.86373 50890000.000 44.86

6396000.000 32.87093 50960000.000 44.87

6403000.000 32.87812 51030000.000 44.89

6410000.000 32.88531 51100000.000 44.90

6417000.000 32.89249 51170000.000 44.91

6424000.000 32.89966 51240000.000 44.92

6431000.000 32.90683 51310000.000 44.94

6438000.000 32.91399 51380000.000 44.95

6445000.000 32.92114 51450000.000 44.96

6452000.000 32.92829 51520000.000 44.97

6459000.000 32.93543 51590000.000 44.99

6466000.000 32.94257 51660000.000 45.00

6473000.000 32.94970 51730000.000 45.01

6480000.000 32.95682 51800000.000 45.02

6487000.000 32.96394 51870000.000 45.03

6494000.000 32.97105 51940000.000 45.05

6501000.000 32.97815 52010000.000 45.06

6508000.000 32.98525 52080000.000 45.07

6515000.000 32.99234 52150000.000 45.08

6522000.000 32.99943 52220000.000 45.09

6529000.000 33.00651 52290000.000 45.11

6536000.000 33.01359 52360000.000 45.12

6543000.000 33.02065 52430000.000 45.13

6550000.000 33.02772 52500000.000 45.14

6557000.000 33.03477 52570000.000 45.15

6564000.000 33.04182 52640000.000 45.17

6571000.000 33.04887 52710000.000 45.18

6578000.000 33.05591 52780000.000 45.19

6585000.000 33.06294 52850000.000 45.20

6592000.000 33.06996 52920000.000 45.21

6599000.000 33.07698 52990000.000 45.23

6606000.000 33.08400 53060000.000 45.24

6613000.000 33.09101 53130000.000 45.25

6620000.000 33.09801 53200000.000 45.26

6627000.000 33.10501 53270000.000 45.27

6634000.000 33.11200 53340000.000 45.29

6641000.000 33.11898 53410000.000 45.30

6648000.000 33.12596 53480000.000 45.31

6655000.000 33.13293 53550000.000 45.32

6662000.000 33.13990 53620000.000 45.33

6669000.000 33.14686 53690000.000 45.35

6676000.000 33.15382 53760000.000 45.36

6683000.000 33.16077 53830000.000 45.37

6690000.000 33.16771 53900000.000 45.38

6697000.000 33.17465 53970000.000 45.39

6704000.000 33.18158 54040000.000 45.40

6711000.000 33.18851 54110000.000 45.42

6718000.000 33.19543 54180000.000 45.43

6725000.000 33.20234 54250000.000 45.44

6732000.000 33.20925 54320000.000 45.45

6739000.000 33.21616 54390000.000 45.46

6746000.000 33.22305 54460000.000 45.48

6753000.000 33.22995 54530000.000 45.49

6760000.000 33.23683 54600000.000 45.50

6767000.000 33.24371 54670000.000 45.51

6774000.000 33.25059 54740000.000 45.52

6781000.000 33.25746 54810000.000 45.53

6788000.000 33.26432 54880000.000 45.55

6795000.000 33.27118 54950000.000 45.56

6802000.000 33.27803 55020000.000 45.57

6809000.000 33.28488 55090000.000 45.58

6816000.000 33.29172 55160000.000 45.59

6823000.000 33.29855 55230000.000 45.60

6830000.000 33.30538 55300000.000 45.61

6837000.000 33.31221 55370000.000 45.63

6844000.000 33.31902 55440000.000 45.64

6851000.000 33.32584 55510000.000 45.65

6858000.000 33.33265 55580000.000 45.66

6865000.000 33.33945 55650000.000 45.67

6872000.000 33.34624 55720000.000 45.68

6879000.000 33.35303 55790000.000 45.70

6886000.000 33.35982 55860000.000 45.71

6893000.000 33.36660 55930000.000 45.72

6900000.000 33.37337 56000000.000 45.73

6907000.000 33.38014 56070000.000 45.74

6914000.000 33.38690 56140000.000 45.75

6921000.000 33.39366 56210000.000 45.76

6928000.000 33.40041 56280000.000 45.78

6935000.000 33.40716 56350000.000 45.79

6942000.000 33.41390 56420000.000 45.80

6949000.000 33.42064 56490000.000 45.81

6956000.000 33.42737 56560000.000 45.82

6963000.000 33.43409 56630000.000 45.83

6970000.000 33.44081 56700000.000 45.84

6977000.000 33.44753 56770000.000 45.85

6984000.000 33.45424 56840000.000 45.87

6991000.000 33.46094 56910000.000 45.88

6998000.000 33.46764 56980000.000 45.89

7005000.000 33.47433 57050000.000 45.90

7012000.000 33.48102 57120000.000 45.91

7019000.000 33.48770 57190000.000 45.92

7026000.000 33.49438 57260000.000 45.93

7033000.000 33.50105 57330000.000 45.94

7040000.000 33.50772 57400000.000 45.96

7047000.000 33.51438 57470000.000 45.97

7054000.000 33.52103 57540000.000 45.98

7061000.000 33.52768 57610000.000 45.99

7068000.000 33.53433 57680000.000 46.00

7075000.000 33.54097 57750000.000 46.01

7082000.000 33.54760 57820000.000 46.02

7089000.000 33.55423 57890000.000 46.03

7096000.000 33.56085 57960000.000 46.05

7103000.000 33.56747 58030000.000 46.06

7110000.000 33.57409 58100000.000 46.07

7117000.000 33.58069 58170000.000 46.08

7124000.000 33.58730 58240000.000 46.09

7131000.000 33.59390 58310000.000 46.10

7138000.000 33.60049 58380000.000 46.11

7145000.000 33.60708 58450000.000 46.12

7152000.000 33.61366 58520000.000 46.13

7159000.000 33.62024 58590000.000 46.14

7166000.000 33.62681 58660000.000 46.16

7173000.000 33.63337 58730000.000 46.17

7180000.000 33.63994 58800000.000 46.18

7187000.000 33.64649 58870000.000 46.19

7194000.000 33.65305 58940000.000 46.20

7201000.000 33.65959 59010000.000 46.21

7208000.000 33.66613 59080000.000 46.22

7215000.000 33.67267 59150000.000 46.23

7222000.000 33.67920 59220000.000 46.24

7229000.000 33.68573 59290000.000 46.25

7236000.000 33.69225 59360000.000 46.27

7243000.000 33.69876 59430000.000 46.28

7250000.000 33.70528 59500000.000 46.29

7257000.000 33.71178 59570000.000 46.30

7264000.000 33.71828 59640000.000 46.31

7271000.000 33.72478 59710000.000 46.32

7278000.000 33.73127 59780000.000 46.33

7285000.000 33.73776 59850000.000 46.34

7292000.000 33.74424 59920000.000 46.35

7299000.000 33.75071 59990000.000 46.36

7306000.000 33.75718 60060000.000 46.37

7313000.000 33.76365 60130000.000 46.38

7320000.000 33.77011 60200000.000 46.40

7327000.000 33.77657 60270000.000 46.41

7334000.000 33.78302 60340000.000 46.42

7341000.000 33.78947 60410000.000 46.43

7348000.000 33.79591 60480000.000 46.44

7355000.000 33.80234 60550000.000 46.45

7362000.000 33.80878 60620000.000 46.46

7369000.000 33.81520 60690000.000 46.47

7376000.000 33.82162 60760000.000 46.48

7383000.000 33.82804 60830000.000 46.49

7390000.000 33.83445 60900000.000 46.50

7397000.000 33.84086 60970000.000 46.51

7404000.000 33.84726 61040000.000 46.52

7411000.000 33.85366 61110000.000 46.54

7418000.000 33.86005 61180000.000 46.55

7425000.000 33.86644 61250000.000 46.56

7432000.000 33.87283 61320000.000 46.57

7439000.000 33.87920 61390000.000 46.58

7446000.000 33.88558 61460000.000 46.59

7453000.000 33.89195 61530000.000 46.60

7460000.000 33.89831 61600000.000 46.61

7467000.000 33.90467 61670000.000 46.62

7474000.000 33.91102 61740000.000 46.63

7481000.000 33.91737 61810000.000 46.64

7488000.000 33.92372 61880000.000 46.65

7495000.000 33.93006 61950000.000 46.66

7502000.000 33.93639 62020000.000 46.67

7509000.000 33.94272 62090000.000 46.68

7516000.000 33.94905 62160000.000 46.69

7523000.000 33.95537 62230000.000 46.70

7530000.000 33.96169 62300000.000 46.71

7537000.000 33.96800 62370000.000 46.73

7544000.000 33.97431 62440000.000 46.74

7551000.000 33.98061 62510000.000 46.75

7558000.000 33.98691 62580000.000 46.76

7565000.000 33.99320 62650000.000 46.77

7572000.000 33.99949 62720000.000 46.78

7579000.000 34.00577 62790000.000 46.79

7586000.000 34.01205 62860000.000 46.80

7593000.000 34.01833 62930000.000 46.81

7600000.000 34.02460 63000000.000 46.82

7607000.000 34.03086 63070000.000 46.83

7614000.000 34.03712 63140000.000 46.84

7621000.000 34.04338 63210000.000 46.85

7628000.000 34.04963 63280000.000 46.86

7635000.000 34.05588 63350000.000 46.87

7642000.000 34.06212 63420000.000 46.88

7649000.000 34.06836 63490000.000 46.89

7656000.000 34.07459 63560000.000 46.90

7663000.000 34.08082 63630000.000 46.91

7670000.000 34.08705 63700000.000 46.92

7677000.000 34.09326 63770000.000 46.93

7684000.000 34.09948 63840000.000 46.94

7691000.000 34.10569 63910000.000 46.95

7698000.000 34.11190 63980000.000 46.96

7705000.000 34.11810 64050000.000 46.97

7712000.000 34.12429 64120000.000 46.98

7719000.000 34.13049 64190000.000 46.99

7726000.000 34.13668 64260000.000 47.01

7733000.000 34.14286 64330000.000 47.02

7740000.000 34.14904 64400000.000 47.03

7747000.000 34.15521 64470000.000 47.04

7754000.000 34.16138 64540000.000 47.05

7761000.000 34.16755 64610000.000 47.06

7768000.000 34.17371 64680000.000 47.07

7775000.000 34.17987 64750000.000 47.08

7782000.000 34.18602 64820000.000 47.09

7789000.000 34.19217 64890000.000 47.10

7796000.000 34.19831 64960000.000 47.11

7803000.000 34.20445 65030000.000 47.12

7810000.000 34.21058 65100000.000 47.13

7817000.000 34.21671 65170000.000 47.14

7824000.000 34.22284 65240000.000 47.15

7831000.000 34.22896 65310000.000 47.16

7838000.000 34.23508 65380000.000 47.17

7845000.000 34.24119 65450000.000 47.18

7852000.000 34.24730 65520000.000 47.19

7859000.000 34.25340 65590000.000 47.20

7866000.000 34.25950 65660000.000 47.21

7873000.000 34.26560 65730000.000 47.22

7880000.000 34.27169 65800000.000 47.23

7887000.000 34.27778 65870000.000 47.24

7894000.000 34.28386 65940000.000 47.25

7901000.000 34.28994 66010000.000 47.26

7908000.000 34.29601 66080000.000 47.27

7915000.000 34.30208 66150000.000 47.28

7922000.000 34.30815 66220000.000 47.29

7929000.000 34.31421 66290000.000 47.30

7936000.000 34.32026 66360000.000 47.31

7943000.000 34.32632 66430000.000 47.32

7950000.000 34.33236 66500000.000 47.33

7957000.000 34.33841 66570000.000 47.34

7964000.000 34.34445 66640000.000 47.35

7971000.000 34.35048 66710000.000 47.36

7978000.000 34.35651 66780000.000 47.37

7985000.000 34.36254 66850000.000 47.38

7992000.000 34.36856 66920000.000 47.39

7999000.000 34.37458 66990000.000 47.40

8006000.000 34.38060 67060000.000 47.41

8013000.000 34.38661 67130000.000 47.42

8020000.000 34.39261 67200000.000 47.43

8027000.000 34.39861 67270000.000 47.44

8034000.000 34.40461 67340000.000 47.45

8041000.000 34.41060 67410000.000 47.46

8048000.000 34.41659 67480000.000 47.47

8055000.000 34.42258 67550000.000 47.48

8062000.000 34.42856 67620000.000 47.49

8069000.000 34.43454 67690000.000 47.50

8076000.000 34.44051 67760000.000 47.51

8083000.000 34.44648 67830000.000 47.52

8090000.000 34.45244 67900000.000 47.53

8097000.000 34.45840 67970000.000 47.54

8104000.000 34.46436 68040000.000 47.55

8111000.000 34.47031 68110000.000 47.56

8118000.000 34.47626 68180000.000 47.57

8125000.000 34.48220 68250000.000 47.57

8132000.000 34.48814 68320000.000 47.58

8139000.000 34.49407 68390000.000 47.59

8146000.000 34.50001 68460000.000 47.60

8153000.000 34.50593 68530000.000 47.61

8160000.000 34.51186 68600000.000 47.62

8167000.000 34.51777 68670000.000 47.63

8174000.000 34.52369 68740000.000 47.64

8181000.000 34.52960 68810000.000 47.65

8188000.000 34.53551 68880000.000 47.66

8195000.000 34.54141 68950000.000 47.67

8202000.000 34.54731 69020000.000 47.68

8209000.000 34.55320 69090000.000 47.69

8216000.000 34.55910 69160000.000 47.70

8223000.000 34.56498 69230000.000 47.71

8230000.000 34.57087 69300000.000 47.72

8237000.000 34.57674 69370000.000 47.73

8244000.000 34.58262 69440000.000 47.74

8251000.000 34.58849 69510000.000 47.75

8258000.000 34.59436 69580000.000 47.76

8265000.000 34.60022 69650000.000 47.77

8272000.000 34.60608 69720000.000 47.78

8279000.000 34.61193 69790000.000 47.79

8286000.000 34.61778 69860000.000 47.80

8293000.000 34.62363 69930000.000 47.81

8300000.000 34.62947 70000000.000 47.82

8307000.000 34.63531 70070000.000 47.83

8314000.000 34.64115 70140000.000 47.84

8321000.000 34.64698 70210000.000 47.85

8328000.000 34.65281 70280000.000 47.85

8335000.000 34.65863 70350000.000 47.86

8342000.000 34.66445 70420000.000 47.87

8349000.000 34.67027 70490000.000 47.88

8356000.000 34.67608 70560000.000 47.89

8363000.000 34.68189 70630000.000 47.90

8370000.000 34.68769 70700000.000 47.91

8377000.000 34.69349 70770000.000 47.92

8384000.000 34.69929 70840000.000 47.93

8391000.000 34.70508 70910000.000 47.94

8398000.000 34.71087 70980000.000 47.95

8405000.000 34.71665 71050000.000 47.96

8412000.000 34.72243 71120000.000 47.97

8419000.000 34.72821 71190000.000 47.98

8426000.000 34.73398 71260000.000 47.99

8433000.000 34.73975 71330000.000 48.00

8440000.000 34.74552 71400000.000 48.01

8447000.000 34.75128 71470000.000 48.02

8454000.000 34.75704 71540000.000 48.03

8461000.000 34.76279 71610000.000 48.03

8468000.000 34.76854 71680000.000 48.04

8475000.000 34.77429 71750000.000 48.05

8482000.000 34.78003 71820000.000 48.06

8489000.000 34.78577 71890000.000 48.07

8496000.000 34.79150 71960000.000 48.08

8503000.000 34.79723 72030000.000 48.09

8510000.000 34.80296 72100000.000 48.10

8517000.000 34.80868 72170000.000 48.11

8524000.000 34.81440 72240000.000 48.12

8531000.000 34.82012 72310000.000 48.13

8538000.000 34.82583 72380000.000 48.14

8545000.000 34.83154 72450000.000 48.15

8552000.000 34.83725 72520000.000 48.16

8559000.000 34.84295 72590000.000 48.17

8566000.000 34.84864 72660000.000 48.17

8573000.000 34.85434 72730000.000 48.18

8580000.000 34.86003 72800000.000 48.19

8587000.000 34.86571 72870000.000 48.20

8594000.000 34.87140 72940000.000 48.21

8601000.000 34.87708 73010000.000 48.22

8608000.000 34.88275 73080000.000 48.23

8615000.000 34.88842 73150000.000 48.24

8622000.000 34.89409 73220000.000 48.25

8629000.000 34.89975 73290000.000 48.26

8636000.000 34.90542 73360000.000 48.27

8643000.000 34.91107 73430000.000 48.28

8650000.000 34.91673 73500000.000 48.29

8657000.000 34.92237 73570000.000 48.29

8664000.000 34.92802 73640000.000 48.30

8671000.000 34.93366 73710000.000 48.31

8678000.000 34.93930 73780000.000 48.32

8685000.000 34.94494 73850000.000 48.33

8692000.000 34.95057 73920000.000 48.34

8699000.000 34.95619 73990000.000 48.35

8706000.000 34.96182 74060000.000 48.36

8713000.000 34.96744 74130000.000 48.37

8720000.000 34.97306 74200000.000 48.38

8727000.000 34.97867 74270000.000 48.39

8734000.000 34.98428 74340000.000 48.40

8741000.000 34.98988 74410000.000 48.40

8748000.000 34.99549 74480000.000 48.41

8755000.000 35.00109 74550000.000 48.42

8762000.000 35.00668 74620000.000 48.43

8769000.000 35.01227 74690000.000 48.44

8776000.000 35.01786 74760000.000 48.45

8783000.000 35.02344 74830000.000 48.46

8790000.000 35.02903 74900000.000 48.47

8797000.000 35.03460 74970000.000 48.48

8804000.000 35.04018 75040000.000 48.49

8811000.000 35.04575 75110000.000 48.50

8818000.000 35.05131 75180000.000 48.50

8825000.000 35.05688 75250000.000 48.51

8832000.000 35.06244 75320000.000 48.52

8839000.000 35.06799 75390000.000 48.53

8846000.000 35.07355 75460000.000 48.54

8853000.000 35.07909 75530000.000 48.55

8860000.000 35.08464 75600000.000 48.56

8867000.000 35.09018 75670000.000 48.57

8874000.000 35.09572 75740000.000 48.58

8881000.000 35.10126 75810000.000 48.59

8888000.000 35.10679 75880000.000 48.59

8895000.000 35.11232 75950000.000 48.60

8902000.000 35.11784 76020000.000 48.61

8909000.000 35.12336 76090000.000 48.62

8916000.000 35.12888 76160000.000 48.63

8923000.000 35.13439 76230000.000 48.64

8930000.000 35.13990 76300000.000 48.65

8937000.000 35.14541 76370000.000 48.66

8944000.000 35.15092 76440000.000 48.67

8951000.000 35.15642 76510000.000 48.67

8958000.000 35.16191 76580000.000 48.68

8965000.000 35.16741 76650000.000 48.69

8972000.000 35.17290 76720000.000 48.70

8979000.000 35.17838 76790000.000 48.71

8986000.000 35.18387 76860000.000 48.72

8993000.000 35.18935 76930000.000 48.73

9000000.000 35.19482 77000000.000 48.74

9007000.000 35.20030 77070000.000 48.75

9014000.000 35.20577 77140000.000 48.75

9021000.000 35.21123 77210000.000 48.76

9028000.000 35.21669 77280000.000 48.77

9035000.000 35.22215 77350000.000 48.78

9042000.000 35.22761 77420000.000 48.79

9049000.000 35.23306 77490000.000 48.80

9056000.000 35.23851 77560000.000 48.81

9063000.000 35.24396 77630000.000 48.82

9070000.000 35.24940 77700000.000 48.82

9077000.000 35.25484 77770000.000 48.83

9084000.000 35.26028 77840000.000 48.84

9091000.000 35.26571 77910000.000 48.85

9098000.000 35.27114 77980000.000 48.86

9105000.000 35.27656 78050000.000 48.87

9112000.000 35.28199 78120000.000 48.88

9119000.000 35.28741 78190000.000 48.89

9126000.000 35.29282 78260000.000 48.90

9133000.000 35.29823 78330000.000 48.90

9140000.000 35.30364 78400000.000 48.91

9147000.000 35.30905 78470000.000 48.92

9154000.000 35.31445 78540000.000 48.93

9161000.000 35.31985 78610000.000 48.94

9168000.000 35.32525 78680000.000 48.95

9175000.000 35.33064 78750000.000 48.96

9182000.000 35.33603 78820000.000 48.96

9189000.000 35.34141 78890000.000 48.97

9196000.000 35.34680 78960000.000 48.98

9203000.000 35.35218 79030000.000 48.99

9210000.000 35.35755 79100000.000 49.00

9217000.000 35.36293 79170000.000 49.01

9224000.000 35.36830 79240000.000 49.02

9231000.000 35.37366 79310000.000 49.03

9238000.000 35.37903 79380000.000 49.03

9245000.000 35.38439 79450000.000 49.04

9252000.000 35.38974 79520000.000 49.05

9259000.000 35.39510 79590000.000 49.06

9266000.000 35.40045 79660000.000 49.07

9273000.000 35.40579 79730000.000 49.08

9280000.000 35.41114 79800000.000 49.09

9287000.000 35.41648 79870000.000 49.09

9294000.000 35.42182 79940000.000 49.10

9301000.000 35.42715 80010000.000 49.11

9308000.000 35.43248 80080000.000 49.12

9315000.000 35.43781 80150000.000 49.13

9322000.000 35.44313 80220000.000 49.14

9329000.000 35.44845 80290000.000 49.15

9336000.000 35.45377 80360000.000 49.15

9343000.000 35.45909 80430000.000 49.16

9350000.000 35.46440 80500000.000 49.17

9357000.000 35.46971 80570000.000 49.18

9364000.000 35.47501 80640000.000 49.19

9371000.000 35.48032 80710000.000 49.20

9378000.000 35.48561 80780000.000 49.21

9385000.000 35.49091 80850000.000 49.21

9392000.000 35.49620 80920000.000 49.22

9399000.000 35.50149 80990000.000 49.23

9406000.000 35.50678 81060000.000 49.24

9413000.000 35.51206 81130000.000 49.25

9420000.000 35.51734 81200000.000 49.26

9427000.000 35.52262 81270000.000 49.27

9434000.000 35.52789 81340000.000 49.27

9441000.000 35.53316 81410000.000 49.28

9448000.000 35.53843 81480000.000 49.29

9455000.000 35.54370 81550000.000 49.30

9462000.000 35.54896 81620000.000 49.31

9469000.000 35.55422 81690000.000 49.32

9476000.000 35.55947 81760000.000 49.32

9483000.000 35.56472 81830000.000 49.33

9490000.000 35.56997 81900000.000 49.34

9497000.000 35.57522 81970000.000 49.35

9504000.000 35.58046 82040000.000 49.36

9511000.000 35.58570 82110000.000 49.37

9518000.000 35.59094 82180000.000 49.38

9525000.000 35.59617 82250000.000 49.38

9532000.000 35.60140 82320000.000 49.39

9539000.000 35.60663 82390000.000 49.40

9546000.000 35.61185 82460000.000 49.41

9553000.000 35.61707 82530000.000 49.42

9560000.000 35.62229 82600000.000 49.43

9567000.000 35.62751 82670000.000 49.43

9574000.000 35.63272 82740000.000 49.44

9581000.000 35.63793 82810000.000 49.45

9588000.000 35.64313 82880000.000 49.46

9595000.000 35.64834 82950000.000 49.47

9602000.000 35.65354 83020000.000 49.48

9609000.000 35.65873 83090000.000 49.48

9616000.000 35.66393 83160000.000 49.49

9623000.000 35.66912 83230000.000 49.50

9630000.000 35.67431 83300000.000 49.51

9637000.000 35.67949 83370000.000 49.52

9644000.000 35.68467 83440000.000 49.53

9651000.000 35.68985 83510000.000 49.53

9658000.000 35.69503 83580000.000 49.54

9665000.000 35.70020 83650000.000 49.55

9672000.000 35.70537 83720000.000 49.56

9679000.000 35.71054 83790000.000 49.57

9686000.000 35.71570 83860000.000 49.58

9693000.000 35.72086 83930000.000 49.58

9700000.000 35.72602 84000000.000 49.59

9707000.000 35.73117 84070000.000 49.60

9714000.000 35.73633 84140000.000 49.61

9721000.000 35.74147 84210000.000 49.62

9728000.000 35.74662 84280000.000 49.63

9735000.000 35.75176 84350000.000 49.63

9742000.000 35.75690 84420000.000 49.64

9749000.000 35.76204 84490000.000 49.65

9756000.000 35.76718 84560000.000 49.66

9763000.000 35.77231 84630000.000 49.67

9770000.000 35.77743 84700000.000 49.67

9777000.000 35.78256 84770000.000 49.68

9784000.000 35.78768 84840000.000 49.69

9791000.000 35.79280 84910000.000 49.70

9798000.000 35.79792 84980000.000 49.71

9805000.000 35.80303 85050000.000 49.72

9812000.000 35.80814 85120000.000 49.72

9819000.000 35.81325 85190000.000 49.73

9826000.000 35.81835 85260000.000 49.74

9833000.000 35.82346 85330000.000 49.75

9840000.000 35.82856 85400000.000 49.76

9847000.000 35.83365 85470000.000 49.76

9854000.000 35.83875 85540000.000 49.77

9861000.000 35.84384 85610000.000 49.78

9868000.000 35.84892 85680000.000 49.79

9875000.000 35.85401 85750000.000 49.80

9882000.000 35.85909 85820000.000 49.81

9889000.000 35.86417 85890000.000 49.81

9896000.000 35.86924 85960000.000 49.82

9903000.000 35.87432 86030000.000 49.83

9910000.000 35.87939 86100000.000 49.84

9917000.000 35.88445 86170000.000 49.85

9924000.000 35.88952 86240000.000 49.85

9931000.000 35.89458 86310000.000 49.86

9938000.000 35.89964 86380000.000 49.87

9945000.000 35.90469 86450000.000 49.88

9952000.000 35.90975 86520000.000 49.89

9959000.000 35.91480 86590000.000 49.89

9966000.000 35.91985 86660000.000 49.90

9973000.000 35.92489 86730000.000 49.91

9980000.000 35.92993 86800000.000 49.92

9987000.000 35.93497 86870000.000 49.93

9994000.000 35.94001 86940000.000 49.93

10001000.000 35.94504 87010000.000 49.94

10008000.000 35.95007 87080000.000 49.95

10015000.000 35.95510 87150000.000 49.96

10022000.000 35.96012 87220000.000 49.97

10029000.000 35.96514 87290000.000 49.97

10036000.000 35.97016 87360000.000 49.98

10043000.000 35.97518 87430000.000 49.99

10050000.000 35.98019 87500000.000 50.00

10057000.000 35.98520 87570000.000 50.01

10064000.000 35.99021 87640000.000 50.01

10071000.000 35.99522 87710000.000 50.02

10078000.000 36.00022 87780000.000 50.03

10085000.000 36.00522 87850000.000 50.04

10092000.000 36.01022 87920000.000 50.05

10099000.000 36.01521 87990000.000 50.05

10106000.000 36.02020 88060000.000 50.06

10113000.000 36.02519 88130000.000 50.07

10120000.000 36.03018 88200000.000 50.08

10127000.000 36.03516 88270000.000 50.09

10134000.000 36.04014 88340000.000 50.09

10141000.000 36.04512 88410000.000 50.10

10148000.000 36.05009 88480000.000 50.11

10155000.000 36.05506 88550000.000 50.12

10162000.000 36.06003 88620000.000 50.13

10169000.000 36.06500 88690000.000 50.13

10176000.000 36.06996 88760000.000 50.14

10183000.000 36.07492 88830000.000 50.15

10190000.000 36.07988 88900000.000 50.16

10197000.000 36.08484 88970000.000 50.17

10204000.000 36.08979 89040000.000 50.17

10211000.000 36.09474 89110000.000 50.18

10218000.000 36.09969 89180000.000 50.19

10225000.000 36.10463 89250000.000 50.20

10232000.000 36.10958 89320000.000 50.21

10239000.000 36.11452 89390000.000 50.21

10246000.000 36.11945 89460000.000 50.22

10253000.000 36.12439 89530000.000 50.23

10260000.000 36.12932 89600000.000 50.24

10267000.000 36.13425 89670000.000 50.24

10274000.000 36.13917 89740000.000 50.25

10281000.000 36.14409 89810000.000 50.26

10288000.000 36.14902 89880000.000 50.27

10295000.000 36.15393 89950000.000 50.28

10302000.000 36.15885 90020000.000 50.28

10309000.000 36.16376 90090000.000 50.29

10316000.000 36.16867 90160000.000 50.30

10323000.000 36.17358 90230000.000 50.31

10330000.000 36.17848 90300000.000 50.31

10337000.000 36.18338 90370000.000 50.32

10344000.000 36.18828 90440000.000 50.33

10351000.000 36.19318 90510000.000 50.34

10358000.000 36.19807 90580000.000 50.35

10365000.000 36.20297 90650000.000 50.35

10372000.000 36.20785 90720000.000 50.36

10379000.000 36.21274 90790000.000 50.37

10386000.000 36.21762 90860000.000 50.38

10393000.000 36.22250 90930000.000 50.38

10400000.000 36.22738 91000000.000 50.39

10407000.000 36.23226 91070000.000 50.40

10414000.000 36.23713 91140000.000 50.41

10421000.000 36.24200 91210000.000 50.42

10428000.000 36.24687 91280000.000 50.42

10435000.000 36.25173 91350000.000 50.43

10442000.000 36.25660 91420000.000 50.44

10449000.000 36.26146 91490000.000 50.45

10456000.000 36.26631 91560000.000 50.45

10463000.000 36.27117 91630000.000 50.46

10470000.000 36.27602 91700000.000 50.47

10477000.000 36.28087 91770000.000 50.48

10484000.000 36.28571 91840000.000 50.49

10491000.000 36.29056 91910000.000 50.49

10498000.000 36.29540 91980000.000 50.50

10505000.000 36.30024 92050000.000 50.51

10512000.000 36.30508 92120000.000 50.52

10519000.000 36.30991 92190000.000 50.52

10526000.000 36.31474 92260000.000 50.53

10533000.000 36.31957 92330000.000 50.54

10540000.000 36.32440 92400000.000 50.55

10547000.000 36.32922 92470000.000 50.55

10554000.000 36.33404 92540000.000 50.56

10561000.000 36.33886 92610000.000 50.57

10568000.000 36.34368 92680000.000 50.58

10575000.000 36.34849 92750000.000 50.58

10582000.000 36.35330 92820000.000 50.59

10589000.000 36.35811 92890000.000 50.60

10596000.000 36.36291 92960000.000 50.61

10603000.000 36.36772 93030000.000 50.62

10610000.000 36.37252 93100000.000 50.62

10617000.000 36.37732 93170000.000 50.63

10624000.000 36.38211 93240000.000 50.64

10631000.000 36.38690 93310000.000 50.65

10638000.000 36.39169 93380000.000 50.65

10645000.000 36.39648 93450000.000 50.66

10652000.000 36.40127 93520000.000 50.67

10659000.000 36.40605 93590000.000 50.68

10666000.000 36.41083 93660000.000 50.68

10673000.000 36.41561 93730000.000 50.69

10680000.000 36.42039 93800000.000 50.70

10687000.000 36.42516 93870000.000 50.71

10694000.000 36.42993 93940000.000 50.71

10701000.000 36.43470 94010000.000 50.72

10708000.000 36.43946 94080000.000 50.73

10715000.000 36.44422 94150000.000 50.74

10722000.000 36.44899 94220000.000 50.74

10729000.000 36.45374 94290000.000 50.75

10736000.000 36.45850 94360000.000 50.76

10743000.000 36.46325 94430000.000 50.77

10750000.000 36.46800 94500000.000 50.77

10757000.000 36.47275 94570000.000 50.78

10764000.000 36.47750 94640000.000 50.79

10771000.000 36.48224 94710000.000 50.80

10778000.000 36.48698 94780000.000 50.80

10785000.000 36.49172 94850000.000 50.81

10792000.000 36.49645 94920000.000 50.82

10799000.000 36.50119 94990000.000 50.83

10806000.000 36.50592 95060000.000 50.83

10813000.000 36.51065 95130000.000 50.84

10820000.000 36.51537 95200000.000 50.85

10827000.000 36.52010 95270000.000 50.86

10834000.000 36.52482 95340000.000 50.86

10841000.000 36.52954 95410000.000 50.87

10848000.000 36.53425 95480000.000 50.88

10855000.000 36.53897 95550000.000 50.89

10862000.000 36.54368 95620000.000 50.89

10869000.000 36.54839 95690000.000 50.90

10876000.000 36.55309 95760000.000 50.91

10883000.000 36.55780 95830000.000 50.92

10890000.000 36.56250 95900000.000 50.92

10897000.000 36.56720 95970000.000 50.93

10904000.000 36.57189 96040000.000 50.94

10911000.000 36.57659 96110000.000 50.95

10918000.000 36.58128 96180000.000 50.95

10925000.000 36.58597 96250000.000 50.96

10932000.000 36.59066 96320000.000 50.97

10939000.000 36.59534 96390000.000 50.98

10946000.000 36.60002 96460000.000 50.98

10953000.000 36.60470 96530000.000 50.99

10960000.000 36.60938 96600000.000 51.00

10967000.000 36.61406 96670000.000 51.01

10974000.000 36.61873 96740000.000 51.01

10981000.000 36.62340 96810000.000 51.02

10988000.000 36.62807 96880000.000 51.03

10995000.000 36.63273 96950000.000 51.03

11002000.000 36.63740 97020000.000 51.04

11009000.000 36.64206 97090000.000 51.05

11016000.000 36.64672 97160000.000 51.06

11023000.000 36.65137 97230000.000 51.06

11030000.000 36.65603 97300000.000 51.07

11037000.000 36.66068 97370000.000 51.08

11044000.000 36.66533 97440000.000 51.09

11051000.000 36.66997 97510000.000 51.09

11058000.000 36.67462 97580000.000 51.10

11065000.000 36.67926 97650000.000 51.11

11072000.000 36.68390 97720000.000 51.12

11079000.000 36.68854 97790000.000 51.12

11086000.000 36.69317 97860000.000 51.13

11093000.000 36.69780 97930000.000 51.14

11100000.000 36.70244 98000000.000 51.15

11107000.000 36.70706 98070000.000 51.15

11114000.000 36.71169 98140000.000 51.16

11121000.000 36.71631 98210000.000 51.17

11128000.000 36.72093 98280000.000 51.17

11135000.000 36.72555 98350000.000 51.18

11142000.000 36.73017 98420000.000 51.19

11149000.000 36.73478 98490000.000 51.20

11156000.000 36.73939 98560000.000 51.20

11163000.000 36.74400 98630000.000 51.21

11170000.000 36.74861 98700000.000 51.22

11177000.000 36.75321 98770000.000 51.23

11184000.000 36.75782 98840000.000 51.23

11191000.000 36.76242 98910000.000 51.24

11198000.000 36.76702 98980000.000 51.25

11205000.000 36.77161 99050000.000 51.25

11212000.000 36.77620 99120000.000 51.26

11219000.000 36.78080 99190000.000 51.27

11226000.000 36.78538 99260000.000 51.28

11233000.000 36.78997 99330000.000 51.28

11240000.000 36.79455 99400000.000 51.29

11247000.000 36.79914 99470000.000 51.30

11254000.000 36.80372 99540000.000 51.30

11261000.000 36.80829 99610000.000 51.31

11268000.000 36.81287 99680000.000 51.32

11275000.000 36.81744 99750000.000 51.33

11282000.000 36.82201 99820000.000 51.33

11289000.000 36.82658 99890000.000 51.34

11296000.000 36.83114 99960000.000 51.35

11303000.000 36.83571 100030000.000 51.36

11310000.000 36.84027 100100000.000 51.36

11317000.000 36.84483 100170000.000 51.37

11324000.000 36.84939 100240000.000 51.38

11331000.000 36.85394 100310000.000 51.38

11338000.000 36.85849 100380000.000 51.39

11345000.000 36.86304 100450000.000 51.40

11352000.000 36.86759 100520000.000 51.41

11359000.000 36.87214 100590000.000 51.41

11366000.000 36.87668 100660000.000 51.42

11373000.000 36.88122 100730000.000 51.43

11380000.000 36.88576 100800000.000 51.43

11387000.000 36.89030 100870000.000 51.44

11394000.000 36.89483 100940000.000 51.45

11401000.000 36.89936 101010000.000 51.46

11408000.000 36.90389 101080000.000 51.46

11415000.000 36.90842 101150000.000 51.47

11422000.000 36.91295 101220000.000 51.48

11429000.000 36.91747 101290000.000 51.48

11436000.000 36.92199 101360000.000 51.49

11443000.000 36.92651 101430000.000 51.50

11450000.000 36.93103 101500000.000 51.51

11457000.000 36.93554 101570000.000 51.51

11464000.000 36.94005 101640000.000 51.52

11471000.000 36.94456 101710000.000 51.53

11478000.000 36.94907 101780000.000 51.53

11485000.000 36.95358 101850000.000 51.54

11492000.000 36.95808 101920000.000 51.55

11499000.000 36.96258 101990000.000 51.55

11506000.000 36.96708 102060000.000 51.56

11513000.000 36.97158 102130000.000 51.57

11520000.000 36.97607 102200000.000 51.58

11527000.000 36.98056 102270000.000 51.58

11534000.000 36.98506 102340000.000 51.59

11541000.000 36.98954 102410000.000 51.60

11548000.000 36.99403 102480000.000 51.60

11555000.000 36.99851 102550000.000 51.61

11562000.000 37.00299 102620000.000 51.62

11569000.000 37.00747 102690000.000 51.63

11576000.000 37.01195 102760000.000 51.63

11583000.000 37.01643 102830000.000 51.64

11590000.000 37.02090 102900000.000 51.65

11597000.000 37.02537 102970000.000 51.65

11604000.000 37.02984 103040000.000 51.66

11611000.000 37.03431 103110000.000 51.67

11618000.000 37.03877 103180000.000 51.67

11625000.000 37.04323 103250000.000 51.68

11632000.000 37.04769 103320000.000 51.69

11639000.000 37.05215 103390000.000 51.70

11646000.000 37.05661 103460000.000 51.70

11653000.000 37.06106 103530000.000 51.71

11660000.000 37.06551 103600000.000 51.72

11667000.000 37.06996 103670000.000 51.72

11674000.000 37.07441 103740000.000 51.73

11681000.000 37.07885 103810000.000 51.74

11688000.000 37.08330 103880000.000 51.74

11695000.000 37.08774 103950000.000 51.75

11702000.000 37.09218 104020000.000 51.76

11709000.000 37.09661 104090000.000 51.77

11716000.000 37.10105 104160000.000 51.77

11723000.000 37.10548 104230000.000 51.78

11730000.000 37.10991 104300000.000 51.79

11737000.000 37.11434 104370000.000 51.79

11744000.000 37.11876 104440000.000 51.80

11751000.000 37.12319 104510000.000 51.81

11758000.000 37.12761 104580000.000 51.81

11765000.000 37.13203 104650000.000 51.82

11772000.000 37.13645 104720000.000 51.83

11779000.000 37.14086 104790000.000 51.83

11786000.000 37.14527 104860000.000 51.84

11793000.000 37.14969 104930000.000 51.85

11800000.000 37.15409 105000000.000 51.86

11807000.000 37.15850 105070000.000 51.86

11814000.000 37.16291 105140000.000 51.87

11821000.000 37.16731 105210000.000 51.88

11828000.000 37.17171 105280000.000 51.88

11835000.000 37.17611 105350000.000 51.89

11842000.000 37.18051 105420000.000 51.90

11849000.000 37.18490 105490000.000 51.90

11856000.000 37.18929 105560000.000 51.91

11863000.000 37.19368 105630000.000 51.92

11870000.000 37.19807 105700000.000 51.92

11877000.000 37.20246 105770000.000 51.93

11884000.000 37.20684 105840000.000 51.94

11891000.000 37.21122 105910000.000 51.95

11898000.000 37.21560 105980000.000 51.95

11905000.000 37.21998 106050000.000 51.96

11912000.000 37.22436 106120000.000 51.97

11919000.000 37.22873 106190000.000 51.97

11926000.000 37.23310 106260000.000 51.98

11933000.000 37.23747 106330000.000 51.99

11940000.000 37.24184 106400000.000 51.99

11947000.000 37.24621 106470000.000 52.00

11954000.000 37.25057 106540000.000 52.01

11961000.000 37.25493 106610000.000 52.01

11968000.000 37.25929 106680000.000 52.02

11975000.000 37.26365 106750000.000 52.03

11982000.000 37.26801 106820000.000 52.03

11989000.000 37.27236 106890000.000 52.04

11996000.000 37.27671 106960000.000 52.05

12003000.000 37.28106 107030000.000 52.05

12010000.000 37.28541 107100000.000 52.06

12017000.000 37.28975 107170000.000 52.07

12024000.000 37.29410 107240000.000 52.08

12031000.000 37.29844 107310000.000 52.08

12038000.000 37.30278 107380000.000 52.09

12045000.000 37.30711 107450000.000 52.10

12052000.000 37.31145 107520000.000 52.10

12059000.000 37.31578 107590000.000 52.11

12066000.000 37.32011 107660000.000 52.12

12073000.000 37.32444 107730000.000 52.12

12080000.000 37.32877 107800000.000 52.13

12087000.000 37.33309 107870000.000 52.14

12094000.000 37.33742 107940000.000 52.14

12101000.000 37.34174 108010000.000 52.15

12108000.000 37.34606 108080000.000 52.16

12115000.000 37.35037 108150000.000 52.16

12122000.000 37.35469 108220000.000 52.17

12129000.000 37.35900 108290000.000 52.18

12136000.000 37.36331 108360000.000 52.18

12143000.000 37.36762 108430000.000 52.19

12150000.000 37.37193 108500000.000 52.20

12157000.000 37.37624 108570000.000 52.20

12164000.000 37.38054 108640000.000 52.21

12171000.000 37.38484 108710000.000 52.22

12178000.000 37.38914 108780000.000 52.22

12185000.000 37.39344 108850000.000 52.23

12192000.000 37.39773 108920000.000 52.24

12199000.000 37.40203 108990000.000 52.24

12206000.000 37.40632 109060000.000 52.25

12213000.000 37.41061 109130000.000 52.26

12220000.000 37.41489 109200000.000 52.26

12227000.000 37.41918 109270000.000 52.27

12234000.000 37.42346 109340000.000 52.28

12241000.000 37.42775 109410000.000 52.28

12248000.000 37.43202 109480000.000 52.29

12255000.000 37.43630 109550000.000 52.30

12262000.000 37.44058 109620000.000 52.30

12269000.000 37.44485 109690000.000 52.31

12276000.000 37.44912 109760000.000 52.32

12283000.000 37.45339 109830000.000 52.32

12290000.000 37.45766 109900000.000 52.33

12297000.000 37.46193 109970000.000 52.34

12304000.000 37.46619 110040000.000 52.34

12311000.000 37.47045 110110000.000 52.35

12318000.000 37.47471 110180000.000 52.36

12325000.000 37.47897 110250000.000 52.36

12332000.000 37.48323 110320000.000 52.37

12339000.000 37.48748 110390000.000 52.38

12346000.000 37.49173 110460000.000 52.38

12353000.000 37.49599 110530000.000 52.39

12360000.000 37.50023 110600000.000 52.40

12367000.000 37.50448 110670000.000 52.40

12374000.000 37.50873 110740000.000 52.41

12381000.000 37.51297 110810000.000 52.42

12388000.000 37.51721 110880000.000 52.42

12395000.000 37.52145 110950000.000 52.43

12402000.000 37.52568 111020000.000 52.44

12409000.000 37.52992 111090000.000 52.44

12416000.000 37.53415 111160000.000 52.45

12423000.000 37.53838 111230000.000 52.46

12430000.000 37.54261 111300000.000 52.46

12437000.000 37.54684 111370000.000 52.47

12444000.000 37.55107 111440000.000 52.48

12451000.000 37.55529 111510000.000 52.48

12458000.000 37.55951 111580000.000 52.49

12465000.000 37.56373 111650000.000 52.50

12472000.000 37.56795 111720000.000 52.50

12479000.000 37.57217 111790000.000 52.51

12486000.000 37.57638 111860000.000 52.52

12493000.000 37.58059 111930000.000 52.52

12500000.000 37.58480 112000000.000 52.53

12507000.000 37.58901 112070000.000 52.54

12514000.000 37.59322 112140000.000 52.54

12521000.000 37.59742 112210000.000 52.55

12528000.000 37.60163 112280000.000 52.56

12535000.000 37.60583 112350000.000 52.56

12542000.000 37.61003 112420000.000 52.57

12549000.000 37.61422 112490000.000 52.58

12556000.000 37.61842 112560000.000 52.58

12563000.000 37.62261 112630000.000 52.59

12570000.000 37.62680 112700000.000 52.59

12577000.000 37.63099 112770000.000 52.60

12584000.000 37.63518 112840000.000 52.61

12591000.000 37.63937 112910000.000 52.61

12598000.000 37.64355 112980000.000 52.62

12605000.000 37.64773 113050000.000 52.63

12612000.000 37.65192 113120000.000 52.63

12619000.000 37.65609 113190000.000 52.64

12626000.000 37.66027 113260000.000 52.65

12633000.000 37.66445 113330000.000 52.65

12640000.000 37.66862 113400000.000 52.66

12647000.000 37.67279 113470000.000 52.67

12654000.000 37.67696 113540000.000 52.67

12661000.000 37.68113 113610000.000 52.68

12668000.000 37.68529 113680000.000 52.69

12675000.000 37.68946 113750000.000 52.69

12682000.000 37.69362 113820000.000 52.70

12689000.000 37.69778 113890000.000 52.71

12696000.000 37.70194 113960000.000 52.71

12703000.000 37.70609 114030000.000 52.72

12710000.000 37.71025 114100000.000 52.72

12717000.000 37.71440 114170000.000 52.73

12724000.000 37.71855 114240000.000 52.74

12731000.000 37.72270 114310000.000 52.74

12738000.000 37.72685 114380000.000 52.75

12745000.000 37.73099 114450000.000 52.76

12752000.000 37.73514 114520000.000 52.76

12759000.000 37.73928 114590000.000 52.77

12766000.000 37.74342 114660000.000 52.78

12773000.000 37.74756 114730000.000 52.78

12780000.000 37.75169 114800000.000 52.79

12787000.000 37.75583 114870000.000 52.80

12794000.000 37.75996 114940000.000 52.80

12801000.000 37.76409 115010000.000 52.81

12808000.000 37.76822 115080000.000 52.82

12815000.000 37.77235 115150000.000 52.82

12822000.000 37.77648 115220000.000 52.83

12829000.000 37.78060 115290000.000 52.83

12836000.000 37.78472 115360000.000 52.84

12843000.000 37.78884 115430000.000 52.85

12850000.000 37.79296 115500000.000 52.85

12857000.000 37.79708 115570000.000 52.86

12864000.000 37.80119 115640000.000 52.87

12871000.000 37.80530 115710000.000 52.87

12878000.000 37.80942 115780000.000 52.88

12885000.000 37.81353 115850000.000 52.89

12892000.000 37.81763 115920000.000 52.89

12899000.000 37.82174 115990000.000 52.90

12906000.000 37.82584 116060000.000 52.90

12913000.000 37.82995 116130000.000 52.91

12920000.000 37.83405 116200000.000 52.92

12927000.000 37.83814 116270000.000 52.92

12934000.000 37.84224 116340000.000 52.93

12941000.000 37.84634 116410000.000 52.94

12948000.000 37.85043 116480000.000 52.94

12955000.000 37.85452 116550000.000 52.95

12962000.000 37.85861 116620000.000 52.96

12969000.000 37.86270 116690000.000 52.96

12976000.000 37.86679 116760000.000 52.97

12983000.000 37.87087 116830000.000 52.97

12990000.000 37.87495 116900000.000 52.98

12997000.000 37.87904 116970000.000 52.99

13004000.000 37.88311 117040000.000 52.99

13011000.000 37.88719 117110000.000 53.00

13018000.000 37.89127 117180000.000 53.01

13025000.000 37.89534 117250000.000 53.01

13032000.000 37.89941 117320000.000 53.02

13039000.000 37.90348 117390000.000 53.03

13046000.000 37.90755 117460000.000 53.03

13053000.000 37.91162 117530000.000 53.04

13060000.000 37.91569 117600000.000 53.04

13067000.000 37.91975 117670000.000 53.05

13074000.000 37.92381 117740000.000 53.06

13081000.000 37.92787 117810000.000 53.06

13088000.000 37.93193 117880000.000 53.07

13095000.000 37.93599 117950000.000 53.08

13102000.000 37.94004 118020000.000 53.08

13109000.000 37.94409 118090000.000 53.09

13116000.000 37.94815 118160000.000 53.09

13123000.000 37.95220 118230000.000 53.10

13130000.000 37.95624 118300000.000 53.11

13137000.000 37.96029 118370000.000 53.11

13144000.000 37.96433 118440000.000 53.12

13151000.000 37.96838 118510000.000 53.13

13158000.000 37.97242 118580000.000 53.13

13165000.000 37.97646 118650000.000 53.14

13172000.000 37.98050 118720000.000 53.15

13179000.000 37.98453 118790000.000 53.15

13186000.000 37.98857 118860000.000 53.16

13193000.000 37.99260 118930000.000 53.16

13200000.000 37.99663 119000000.000 53.17

13207000.000 38.00066 119070000.000 53.18

13214000.000 38.00469 119140000.000 53.18

13221000.000 38.00871 119210000.000 53.19

13228000.000 38.01274 119280000.000 53.20

13235000.000 38.01676 119350000.000 53.20

13242000.000 38.02078 119420000.000 53.21

13249000.000 38.02480 119490000.000 53.21

13256000.000 38.02881 119560000.000 53.22

13263000.000 38.03283 119630000.000 53.23

13270000.000 38.03684 119700000.000 53.23

13277000.000 38.04086 119770000.000 53.24

13284000.000 38.04487 119840000.000 53.24

13291000.000 38.04887 119910000.000 53.25

13298000.000 38.05288 119980000.000 53.26

13305000.000 38.05689 120050000.000 53.26

13312000.000 38.06089 120120000.000 53.27

13319000.000 38.06489 120190000.000 53.28

13326000.000 38.06889 120260000.000 53.28

13333000.000 38.07289 120330000.000 53.29

13340000.000 38.07689 120400000.000 53.29

13347000.000 38.08088 120470000.000 53.30

13354000.000 38.08488 120540000.000 53.31

13361000.000 38.08887 120610000.000 53.31

13368000.000 38.09286 120680000.000 53.32

13375000.000 38.09685 120750000.000 53.33

13382000.000 38.10083 120820000.000 53.33

13389000.000 38.10482 120890000.000 53.34

13396000.000 38.10880 120960000.000 53.34

13403000.000 38.11279 121030000.000 53.35

13410000.000 38.11677 121100000.000 53.36

13417000.000 38.12074 121170000.000 53.36

13424000.000 38.12472 121240000.000 53.37

13431000.000 38.12870 121310000.000 53.37

13438000.000 38.13267 121380000.000 53.38

13445000.000 38.13664 121450000.000 53.39

13452000.000 38.14061 121520000.000 53.39

13459000.000 38.14458 121590000.000 53.40

13466000.000 38.14855 121660000.000 53.41

13473000.000 38.15251 121730000.000 53.41

13480000.000 38.15648 121800000.000 53.42

13487000.000 38.16044 121870000.000 53.42

13494000.000 38.16440 121940000.000 53.43

13501000.000 38.16836 122010000.000 53.44

13508000.000 38.17231 122080000.000 53.44

13515000.000 38.17627 122150000.000 53.45

13522000.000 38.18022 122220000.000 53.45

13529000.000 38.18418 122290000.000 53.46

13536000.000 38.18813 122360000.000 53.47

13543000.000 38.19208 122430000.000 53.47

13550000.000 38.19602 122500000.000 53.48

13557000.000 38.19997 122570000.000 53.49

13564000.000 38.20391 122640000.000 53.49

13571000.000 38.20785 122710000.000 53.50

13578000.000 38.21180 122780000.000 53.50

13585000.000 38.21573 122850000.000 53.51

13592000.000 38.21967 122920000.000 53.52

13599000.000 38.22361 122990000.000 53.52

13606000.000 38.22754 123060000.000 53.53

13613000.000 38.23147 123130000.000 53.53

13620000.000 38.23541 123200000.000 53.54

13627000.000 38.23934 123270000.000 53.55

13634000.000 38.24326 123340000.000 53.55

13641000.000 38.24719 123410000.000 53.56

13648000.000 38.25111 123480000.000 53.56

13655000.000 38.25504 123550000.000 53.57

13662000.000 38.25896 123620000.000 53.58

13669000.000 38.26288 123690000.000 53.58

13676000.000 38.26680 123760000.000 53.59

13683000.000 38.27071 123830000.000 53.59

13690000.000 38.27463 123900000.000 53.60

13697000.000 38.27854 123970000.000 53.61

13704000.000 38.28245 124040000.000 53.61

13711000.000 38.28636 124110000.000 53.62

13718000.000 38.29027 124180000.000 53.63

13725000.000 38.29418 124250000.000 53.63

13732000.000 38.29808 124320000.000 53.64

13739000.000 38.30199 124390000.000 53.64

13746000.000 38.30589 124460000.000 53.65

13753000.000 38.30979 124530000.000 53.66

13760000.000 38.31369 124600000.000 53.66

13767000.000 38.31759 124670000.000 53.67

13774000.000 38.32148 124740000.000 53.67

13781000.000 38.32538 124810000.000 53.68

13788000.000 38.32927 124880000.000 53.69

13795000.000 38.33316 124950000.000 53.69

13802000.000 38.33705 125020000.000 53.70

13809000.000 38.34094 125090000.000 53.70

13816000.000 38.34482 125160000.000 53.71

13823000.000 38.34871 125230000.000 53.72

13830000.000 38.35259 125300000.000 53.72

13837000.000 38.35647 125370000.000 53.73

13844000.000 38.36035 125440000.000 53.73

13851000.000 38.36423 125510000.000 53.74

13858000.000 38.36811 125580000.000 53.75

13865000.000 38.37198 125650000.000 53.75

13872000.000 38.37586 125720000.000 53.76

13879000.000 38.37973 125790000.000 53.76

13886000.000 38.38360 125860000.000 53.77

13893000.000 38.38747 125930000.000 53.78

13900000.000 38.39134 126000000.000 53.78

13907000.000 38.39520 126070000.000 53.79

13914000.000 38.39907 126140000.000 53.79

13921000.000 38.40293 126210000.000 53.80

13928000.000 38.40679 126280000.000 53.81

13935000.000 38.41065 126350000.000 53.81

13942000.000 38.41451 126420000.000 53.82

13949000.000 38.41837 126490000.000 53.82

13956000.000 38.42222 126560000.000 53.83

13963000.000 38.42608 126630000.000 53.84

13970000.000 38.42993 126700000.000 53.84

13977000.000 38.43378 126770000.000 53.85

13984000.000 38.43763 126840000.000 53.85

13991000.000 38.44147 126910000.000 53.86

13998000.000 38.44532 126980000.000 53.86

14005000.000 38.44916 127050000.000 53.87

14012000.000 38.45301 127120000.000 53.88

14019000.000 38.45685 127190000.000 53.88

14026000.000 38.46069 127260000.000 53.89

14033000.000 38.46453 127330000.000 53.89

14040000.000 38.46836 127400000.000 53.90

14047000.000 38.47220 127470000.000 53.91

14054000.000 38.47603 127540000.000 53.91

14061000.000 38.47986 127610000.000 53.92

14068000.000 38.48369 127680000.000 53.92

14075000.000 38.48752 127750000.000 53.93

14082000.000 38.49135 127820000.000 53.94

14089000.000 38.49518 127890000.000 53.94

14096000.000 38.49900 127960000.000 53.95

14103000.000 38.50282 128030000.000 53.95

14110000.000 38.50665 128100000.000 53.96

14117000.000 38.51047 128170000.000 53.97

14124000.000 38.51428 128240000.000 53.97

14131000.000 38.51810 128310000.000 53.98

14138000.000 38.52192 128380000.000 53.98

14145000.000 38.52573 128450000.000 53.99

14152000.000 38.52954 128520000.000 53.99

14159000.000 38.53335 128590000.000 54.00

14166000.000 38.53716 128660000.000 54.01

14173000.000 38.54097 128730000.000 54.01

14180000.000 38.54478 128800000.000 54.02

14187000.000 38.54858 128870000.000 54.02

14194000.000 38.55238 128940000.000 54.03

14201000.000 38.55619 129010000.000 54.04

14208000.000 38.55999 129080000.000 54.04

14215000.000 38.56379 129150000.000 54.05

14222000.000 38.56758 129220000.000 54.05

14229000.000 38.57138 129290000.000 54.06

14236000.000 38.57517 129360000.000 54.07

14243000.000 38.57897 129430000.000 54.07

14250000.000 38.58276 129500000.000 54.08

14257000.000 38.58655 129570000.000 54.08

14264000.000 38.59034 129640000.000 54.09

14271000.000 38.59412 129710000.000 54.09

14278000.000 38.59791 129780000.000 54.10

14285000.000 38.60169 129850000.000 54.11

14292000.000 38.60547 129920000.000 54.11

14299000.000 38.60925 129990000.000 54.12

14306000.000 38.61303 130060000.000 54.12

14313000.000 38.61681 130130000.000 54.13

14320000.000 38.62059 130200000.000 54.14

14327000.000 38.62436 130270000.000 54.14

14334000.000 38.62814 130340000.000 54.15

14341000.000 38.63191 130410000.000 54.15

14348000.000 38.63568 130480000.000 54.16

14355000.000 38.63945 130550000.000 54.16

14362000.000 38.64322 130620000.000 54.17

14369000.000 38.64698 130690000.000 54.18

14376000.000 38.65075 130760000.000 54.18

14383000.000 38.65451 130830000.000 54.19

14390000.000 38.65827 130900000.000 54.19

14397000.000 38.66203 130970000.000 54.20

14404000.000 38.66579 131040000.000 54.20

14411000.000 38.66955 131110000.000 54.21

14418000.000 38.67330 131180000.000 54.22

14425000.000 38.67706 131250000.000 54.22

14432000.000 38.68081 131320000.000 54.23

14439000.000 38.68456 131390000.000 54.23

14446000.000 38.68831 131460000.000 54.24

14453000.000 38.69206 131530000.000 54.25

14460000.000 38.69581 131600000.000 54.25

14467000.000 38.69956 131670000.000 54.26

14474000.000 38.70330 131740000.000 54.26

14481000.000 38.70704 131810000.000 54.27

14488000.000 38.71078 131880000.000 54.27

14495000.000 38.71452 131950000.000 54.28

14502000.000 38.71826 132020000.000 54.29

14509000.000 38.72200 132090000.000 54.29

14516000.000 38.72574 132160000.000 54.30

14523000.000 38.72947 132230000.000 54.30

14530000.000 38.73320 132300000.000 54.31

14537000.000 38.73693 132370000.000 54.31

14544000.000 38.74066 132440000.000 54.32

14551000.000 38.74439 132510000.000 54.33

14558000.000 38.74812 132580000.000 54.33

14565000.000 38.75184 132650000.000 54.34

14572000.000 38.75557 132720000.000 54.34

14579000.000 38.75929 132790000.000 54.35

14586000.000 38.76301 132860000.000 54.35

14593000.000 38.76673 132930000.000 54.36

14600000.000 38.77045 133000000.000 54.37

14607000.000 38.77417 133070000.000 54.37

14614000.000 38.77788 133140000.000 54.38

14621000.000 38.78160 133210000.000 54.38

14628000.000 38.78531 133280000.000 54.39

14635000.000 38.78902 133350000.000 54.39

14642000.000 38.79273 133420000.000 54.40

14649000.000 38.79644 133490000.000 54.41

14656000.000 38.80015 133560000.000 54.41

14663000.000 38.80385 133630000.000 54.42

14670000.000 38.80756 133700000.000 54.42

14677000.000 38.81126 133770000.000 54.43

14684000.000 38.81496 133840000.000 54.43

14691000.000 38.81866 133910000.000 54.44

14698000.000 38.82236 133980000.000 54.45

14705000.000 38.82606 134050000.000 54.45

14712000.000 38.82975 134120000.000 54.46

14719000.000 38.83345 134190000.000 54.46

14726000.000 38.83714 134260000.000 54.47

14733000.000 38.84083 134330000.000 54.47

14740000.000 38.84452 134400000.000 54.48

14747000.000 38.84821 134470000.000 54.49

14754000.000 38.85190 134540000.000 54.49

14761000.000 38.85558 134610000.000 54.50

14768000.000 38.85927 134680000.000 54.50

14775000.000 38.86295 134750000.000 54.51

14782000.000 38.86663 134820000.000 54.51

14789000.000 38.87031 134890000.000 54.52

14796000.000 38.87399 134960000.000 54.53

14803000.000 38.87767 135030000.000 54.53

14810000.000 38.88135 135100000.000 54.54

14817000.000 38.88502 135170000.000 54.54

14824000.000 38.88869 135240000.000 54.55

14831000.000 38.89237 135310000.000 54.55

14838000.000 38.89604 135380000.000 54.56

14845000.000 38.89971 135450000.000 54.56

14852000.000 38.90337 135520000.000 54.57

14859000.000 38.90704 135590000.000 54.58

14866000.000 38.91071 135660000.000 54.58

14873000.000 38.91437 135730000.000 54.59

14880000.000 38.91803 135800000.000 54.59

14887000.000 38.92169 135870000.000 54.60

14894000.000 38.92535 135940000.000 54.60

14901000.000 38.92901 136010000.000 54.61

14908000.000 38.93267 136080000.000 54.62

14915000.000 38.93632 136150000.000 54.62

14922000.000 38.93998 136220000.000 54.63

14929000.000 38.94363 136290000.000 54.63

14936000.000 38.94728 136360000.000 54.64

14943000.000 38.95093 136430000.000 54.64

14950000.000 38.95458 136500000.000 54.65

14957000.000 38.95823 136570000.000 54.65

14964000.000 38.96187 136640000.000 54.66

14971000.000 38.96552 136710000.000 54.67

14978000.000 38.96916 136780000.000 54.67

14985000.000 38.97280 136850000.000 54.68

14992000.000 38.97644 136920000.000 54.68

14999000.000 38.98008 136990000.000 54.69

15006000.000 38.98372 137060000.000 54.69

15013000.000 38.98736 137130000.000 54.70

15020000.000 38.99099 137200000.000 54.71

15027000.000 38.99462 137270000.000 54.71

15034000.000 38.99826 137340000.000 54.72

15041000.000 39.00189 137410000.000 54.72

15048000.000 39.00552 137480000.000 54.73

15055000.000 39.00915 137550000.000 54.73

15062000.000 39.01277 137620000.000 54.74

15069000.000 39.01640 137690000.000 54.74

15076000.000 39.02002 137760000.000 54.75

15083000.000 39.02364 137830000.000 54.76

15090000.000 39.02727 137900000.000 54.76

15097000.000 39.03089 137970000.000 54.77

15104000.000 39.03451 138040000.000 54.77

15111000.000 39.03812 138110000.000 54.78

15118000.000 39.04174 138180000.000 54.78

15125000.000 39.04535 138250000.000 54.79

15132000.000 39.04897 138320000.000 54.79

15139000.000 39.05258 138390000.000 54.80

15146000.000 39.05619 138460000.000 54.81

15153000.000 39.05980 138530000.000 54.81

15160000.000 39.06341 138600000.000 54.82

15167000.000 39.06701 138670000.000 54.82

15174000.000 39.07062 138740000.000 54.83

15181000.000 39.07422 138810000.000 54.83

15188000.000 39.07783 138880000.000 54.84

15195000.000 39.08143 138950000.000 54.84

15202000.000 39.08503 139020000.000 54.85

15209000.000 39.08863 139090000.000 54.86

15216000.000 39.09222 139160000.000 54.86

15223000.000 39.09582 139230000.000 54.87

15230000.000 39.09942 139300000.000 54.87

15237000.000 39.10301 139370000.000 54.88

15244000.000 39.10660 139440000.000 54.88

15251000.000 39.11019 139510000.000 54.89

15258000.000 39.11378 139580000.000 54.89

15265000.000 39.11737 139650000.000 54.90

15272000.000 39.12096 139720000.000 54.90

15279000.000 39.12454 139790000.000 54.91

15286000.000 39.12813 139860000.000 54.92

15293000.000 39.13171 139930000.000 54.92

15300000.000 39.13529 140000000.000 54.93

15307000.000 39.13887 140070000.000 54.93

15314000.000 39.14245 140140000.000 54.94

15321000.000 39.14603 140210000.000 54.94

15328000.000 39.14960 140280000.000 54.95

15335000.000 39.15318 140350000.000 54.95

15342000.000 39.15675 140420000.000 54.96

15349000.000 39.16033 140490000.000 54.97

15356000.000 39.16390 140560000.000 54.97

15363000.000 39.16747 140630000.000 54.98

15370000.000 39.17104 140700000.000 54.98

15377000.000 39.17460 140770000.000 54.99

15384000.000 39.17817 140840000.000 54.99

15391000.000 39.18173 140910000.000 55.00

15398000.000 39.18530 140980000.000 55.00

15405000.000 39.18886 141050000.000 55.01

15412000.000 39.19242 141120000.000 55.01

15419000.000 39.19598 141190000.000 55.02

15426000.000 39.19954 141260000.000 55.03

15433000.000 39.20309 141330000.000 55.03

15440000.000 39.20665 141400000.000 55.04

15447000.000 39.21020 141470000.000 55.04

15454000.000 39.21376 141540000.000 55.05

15461000.000 39.21731 141610000.000 55.05

15468000.000 39.22086 141680000.000 55.06

15475000.000 39.22441 141750000.000 55.06

15482000.000 39.22796 141820000.000 55.07

15489000.000 39.23150 141890000.000 55.07

15496000.000 39.23505 141960000.000 55.08

15503000.000 39.23859 142030000.000 55.09

15510000.000 39.24214 142100000.000 55.09

15517000.000 39.24568 142170000.000 55.10

15524000.000 39.24922 142240000.000 55.10

15531000.000 39.25276 142310000.000 55.11

15538000.000 39.25629 142380000.000 55.11

15545000.000 39.25983 142450000.000 55.12

15552000.000 39.26337 142520000.000 55.12

15559000.000 39.26690 142590000.000 55.13

15566000.000 39.27043 142660000.000 55.13

15573000.000 39.27396 142730000.000 55.14

15580000.000 39.27749 142800000.000 55.14

15587000.000 39.28102 142870000.000 55.15

15594000.000 39.28455 142940000.000 55.16

15601000.000 39.28808 143010000.000 55.16

15608000.000 39.29160 143080000.000 55.17

15615000.000 39.29513 143150000.000 55.17

15622000.000 39.29865 143220000.000 55.18

15629000.000 39.30217 143290000.000 55.18

15636000.000 39.30569 143360000.000 55.19

15643000.000 39.30921 143430000.000 55.19

15650000.000 39.31273 143500000.000 55.20

15657000.000 39.31624 143570000.000 55.20

15664000.000 39.31976 143640000.000 55.21

15671000.000 39.32327 143710000.000 55.21

15678000.000 39.32678 143780000.000 55.22

15685000.000 39.33029 143850000.000 55.23

15692000.000 39.33380 143920000.000 55.23

15699000.000 39.33731 143990000.000 55.24

15706000.000 39.34082 144060000.000 55.24

15713000.000 39.34433 144130000.000 55.25

15720000.000 39.34783 144200000.000 55.25

15727000.000 39.35133 144270000.000 55.26

15734000.000 39.35484 144340000.000 55.26

15741000.000 39.35834 144410000.000 55.27

15748000.000 39.36184 144480000.000 55.27

15755000.000 39.36534 144550000.000 55.28

15762000.000 39.36883 144620000.000 55.28

15769000.000 39.37233 144690000.000 55.29

15776000.000 39.37582 144760000.000 55.30

15783000.000 39.37932 144830000.000 55.30

15790000.000 39.38281 144900000.000 55.31

15797000.000 39.38630 144970000.000 55.31

15804000.000 39.38979 145040000.000 55.32

15811000.000 39.39328 145110000.000 55.32

15818000.000 39.39677 145180000.000 55.33

15825000.000 39.40025 145250000.000 55.33

15832000.000 39.40374 145320000.000 55.34

15839000.000 39.40722 145390000.000 55.34

15846000.000 39.41071 145460000.000 55.35

15853000.000 39.41419 145530000.000 55.35

15860000.000 39.41767 145600000.000 55.36

15867000.000 39.42115 145670000.000 55.36

15874000.000 39.42462 145740000.000 55.37

15881000.000 39.42810 145810000.000 55.38

15888000.000 39.43158 145880000.000 55.38

15895000.000 39.43505 145950000.000 55.39

15902000.000 39.43852 146020000.000 55.39

15909000.000 39.44199 146090000.000 55.40

15916000.000 39.44546 146160000.000 55.40

15923000.000 39.44893 146230000.000 55.41

15930000.000 39.45240 146300000.000 55.41

15937000.000 39.45587 146370000.000 55.42

15944000.000 39.45933 146440000.000 55.42

15951000.000 39.46280 146510000.000 55.43

15958000.000 39.46626 146580000.000 55.43

15965000.000 39.46972 146650000.000 55.44

15972000.000 39.47318 146720000.000 55.44

15979000.000 39.47664 146790000.000 55.45

15986000.000 39.48010 146860000.000 55.45

15993000.000 39.48356 146930000.000 55.46

16000000.000 39.48701 147000000.000 55.47

16007000.000 39.49047 147070000.000 55.47

16014000.000 39.49392 147140000.000 55.48

16021000.000 39.49737 147210000.000 55.48

16028000.000 39.50082 147280000.000 55.49

16035000.000 39.50427 147350000.000 55.49

16042000.000 39.50772 147420000.000 55.50

16049000.000 39.51117 147490000.000 55.50

16056000.000 39.51462 147560000.000 55.51

16063000.000 39.51806 147630000.000 55.51

16070000.000 39.52150 147700000.000 55.52

16077000.000 39.52495 147770000.000 55.52

16084000.000 39.52839 147840000.000 55.53

16091000.000 39.53183 147910000.000 55.53

16098000.000 39.53527 147980000.000 55.54

16105000.000 39.53870 148050000.000 55.54

16112000.000 39.54214 148120000.000 55.55

16119000.000 39.54558 148190000.000 55.55

16126000.000 39.54901 148260000.000 55.56

16133000.000 39.55244 148330000.000 55.57

16140000.000 39.55587 148400000.000 55.57

16147000.000 39.55930 148470000.000 55.58

16154000.000 39.56273 148540000.000 55.58

16161000.000 39.56616 148610000.000 55.59

16168000.000 39.56959 148680000.000 55.59

16175000.000 39.57302 148750000.000 55.60

16182000.000 39.57644 148820000.000 55.60

16189000.000 39.57986 148890000.000 55.61

16196000.000 39.58329 148960000.000 55.61

16203000.000 39.58671 149030000.000 55.62

16210000.000 39.59013 149100000.000 55.62

16217000.000 39.59354 149170000.000 55.63

16224000.000 39.59696 149240000.000 55.63

16231000.000 39.60038 149310000.000 55.64

16238000.000 39.60379 149380000.000 55.64

16245000.000 39.60721 149450000.000 55.65

16252000.000 39.61062 149520000.000 55.65

16259000.000 39.61403 149590000.000 55.66

16266000.000 39.61744 149660000.000 55.66

16273000.000 39.62085 149730000.000 55.67

16280000.000 39.62426 149800000.000 55.68

16287000.000 39.62767 149870000.000 55.68

16294000.000 39.63107 149940000.000 55.69

16301000.000 39.63448 150010000.000 55.69

16308000.000 39.63788 150080000.000 55.70

16315000.000 39.64128 150150000.000 55.70

16322000.000 39.64468 150220000.000 55.71

16329000.000 39.64808 150290000.000 55.71

16336000.000 39.65148 150360000.000 55.72

16343000.000 39.65488 150430000.000 55.72

16350000.000 39.65828 150500000.000 55.73

16357000.000 39.66167 150570000.000 55.73

16364000.000 39.66507 150640000.000 55.74

16371000.000 39.66846 150710000.000 55.74

16378000.000 39.67185 150780000.000 55.75

16385000.000 39.67524 150850000.000 55.75

16392000.000 39.67863 150920000.000 55.76

16399000.000 39.68202 150990000.000 55.76

16406000.000 39.68541 151060000.000 55.77

16413000.000 39.68879 151130000.000 55.77

16420000.000 39.69218 151200000.000 55.78

16427000.000 39.69556 151270000.000 55.78

16434000.000 39.69894 151340000.000 55.79

16441000.000 39.70232 151410000.000 55.79

16448000.000 39.70570 151480000.000 55.80

16455000.000 39.70908 151550000.000 55.80

16462000.000 39.71246 151620000.000 55.81

16469000.000 39.71584 151690000.000 55.81

16476000.000 39.71921 151760000.000 55.82

16483000.000 39.72259 151830000.000 55.83

16490000.000 39.72596 151900000.000 55.83

16497000.000 39.72933 151970000.000 55.84

16504000.000 39.73270 152040000.000 55.84

16511000.000 39.73607 152110000.000 55.85

16518000.000 39.73944 152180000.000 55.85

16525000.000 39.74281 152250000.000 55.86

16532000.000 39.74618 152320000.000 55.86

16539000.000 39.74954 152390000.000 55.87

16546000.000 39.75291 152460000.000 55.87

16553000.000 39.75627 152530000.000 55.88

16560000.000 39.75963 152600000.000 55.88

16567000.000 39.76299 152670000.000 55.89

16574000.000 39.76635 152740000.000 55.89

16581000.000 39.76971 152810000.000 55.90

16588000.000 39.77307 152880000.000 55.90

16595000.000 39.77642 152950000.000 55.91

16602000.000 39.77978 153020000.000 55.91

16609000.000 39.78313 153090000.000 55.92

16616000.000 39.78649 153160000.000 55.92

16623000.000 39.78984 153230000.000 55.93

16630000.000 39.79319 153300000.000 55.93

16637000.000 39.79654 153370000.000 55.94

16644000.000 39.79989 153440000.000 55.94

16651000.000 39.80323 153510000.000 55.95

16658000.000 39.80658 153580000.000 55.95

16665000.000 39.80992 153650000.000 55.96

16672000.000 39.81327 153720000.000 55.96

16679000.000 39.81661 153790000.000 55.97

16686000.000 39.81995 153860000.000 55.97

16693000.000 39.82329 153930000.000 55.98

16700000.000 39.82663 154000000.000 55.98

16707000.000 39.82997 154070000.000 55.99

16714000.000 39.83331 154140000.000 55.99

16721000.000 39.83664 154210000.000 56.00

16728000.000 39.83998 154280000.000 56.00

16735000.000 39.84331 154350000.000 56.01

16742000.000 39.84664 154420000.000 56.01

16749000.000 39.84998 154490000.000 56.02

16756000.000 39.85331 154560000.000 56.02

16763000.000 39.85664 154630000.000 56.03

16770000.000 39.85996 154700000.000 56.03

16777000.000 39.86329 154770000.000 56.04

16784000.000 39.86662 154840000.000 56.04

16791000.000 39.86994 154910000.000 56.05

16798000.000 39.87326 154980000.000 56.05

16805000.000 39.87659 155050000.000 56.06

16812000.000 39.87991 155120000.000 56.06

16819000.000 39.88323 155190000.000 56.07

16826000.000 39.88655 155260000.000 56.08

16833000.000 39.88987 155330000.000 56.08

16840000.000 39.89318 155400000.000 56.09

16847000.000 39.89650 155470000.000 56.09

16854000.000 39.89981 155540000.000 56.10

16861000.000 39.90313 155610000.000 56.10

16868000.000 39.90644 155680000.000 56.11

16875000.000 39.90975 155750000.000 56.11

16882000.000 39.91306 155820000.000 56.12

16889000.000 39.91637 155890000.000 56.12

16896000.000 39.91968 155960000.000 56.13

16903000.000 39.92299 156030000.000 56.13

16910000.000 39.92629 156100000.000 56.14

16917000.000 39.92960 156170000.000 56.14

16924000.000 39.93290 156240000.000 56.15

16931000.000 39.93621 156310000.000 56.15

16938000.000 39.93951 156380000.000 56.16

16945000.000 39.94281 156450000.000 56.16

16952000.000 39.94611 156520000.000 56.17

16959000.000 39.94941 156590000.000 56.17

16966000.000 39.95270 156660000.000 56.18

16973000.000 39.95600 156730000.000 56.18

16980000.000 39.95930 156800000.000 56.19

16987000.000 39.96259 156870000.000 56.19

16994000.000 39.96588 156940000.000 56.20

17001000.000 39.96917 157010000.000 56.20

17008000.000 39.97247 157080000.000 56.21

17015000.000 39.97575 157150000.000 56.21

17022000.000 39.97904 157220000.000 56.22

17029000.000 39.98233 157290000.000 56.22

17036000.000 39.98562 157360000.000 56.23

17043000.000 39.98890 157430000.000 56.23

17050000.000 39.99219 157500000.000 56.24

17057000.000 39.99547 157570000.000 56.24

17064000.000 39.99875 157640000.000 56.25

17071000.000 40.00203 157710000.000 56.25

17078000.000 40.00531 157780000.000 56.26

17085000.000 40.00859 157850000.000 56.26

17092000.000 40.01187 157920000.000 56.27

17099000.000 40.01515 157990000.000 56.27

17106000.000 40.01842 158060000.000 56.28

17113000.000 40.02170 158130000.000 56.28

17120000.000 40.02497 158200000.000 56.29

17127000.000 40.02824 158270000.000 56.29

17134000.000 40.03152 158340000.000 56.30

17141000.000 40.03479 158410000.000 56.30

17148000.000 40.03806 158480000.000 56.31

17155000.000 40.04132 158550000.000 56.31

17162000.000 40.04459 158620000.000 56.32

17169000.000 40.04786 158690000.000 56.32

17176000.000 40.05112 158760000.000 56.33

17183000.000 40.05439 158830000.000 56.33

17190000.000 40.05765 158900000.000 56.34

17197000.000 40.06091 158970000.000 56.34

17204000.000 40.06417 159040000.000 56.35

17211000.000 40.06743 159110000.000 56.35

17218000.000 40.07069 159180000.000 56.36

17225000.000 40.07395 159250000.000 56.36

17232000.000 40.07720 159320000.000 56.37

17239000.000 40.08046 159390000.000 56.37

17246000.000 40.08371 159460000.000 56.38

17253000.000 40.08697 159530000.000 56.38

17260000.000 40.09022 159600000.000 56.39

17267000.000 40.09347 159670000.000 56.39

17274000.000 40.09672 159740000.000 56.40

17281000.000 40.09997 159810000.000 56.40

17288000.000 40.10322 159880000.000 56.40

17295000.000 40.10647 159950000.000 56.41

17302000.000 40.10971 160020000.000 56.41

17309000.000 40.11296 160090000.000 56.42

17316000.000 40.11620 160160000.000 56.42

17323000.000 40.11944 160230000.000 56.43

17330000.000 40.12269 160300000.000 56.43

17337000.000 40.12593 160370000.000 56.44

17344000.000 40.12917 160440000.000 56.44

17351000.000 40.13240 160510000.000 56.45

17358000.000 40.13564 160580000.000 56.45

17365000.000 40.13888 160650000.000 56.46

17372000.000 40.14211 160720000.000 56.46

17379000.000 40.14535 160790000.000 56.47

17386000.000 40.14858 160860000.000 56.47

17393000.000 40.15182 160930000.000 56.48

17400000.000 40.15505 161000000.000 56.48

17407000.000 40.15828 161070000.000 56.49

17414000.000 40.16151 161140000.000 56.49

17421000.000 40.16473 161210000.000 56.50

17428000.000 40.16796 161280000.000 56.50

17435000.000 40.17119 161350000.000 56.51

17442000.000 40.17441 161420000.000 56.51

17449000.000 40.17764 161490000.000 56.52

17456000.000 40.18086 161560000.000 56.52

17463000.000 40.18408 161630000.000 56.53

17470000.000 40.18730 161700000.000 56.53

17477000.000 40.19052 161770000.000 56.54

17484000.000 40.19374 161840000.000 56.54

17491000.000 40.19696 161910000.000 56.55

17498000.000 40.20018 161980000.000 56.55

17505000.000 40.20339 162050000.000 56.56

17512000.000 40.20661 162120000.000 56.56

17519000.000 40.20982 162190000.000 56.57

17526000.000 40.21303 162260000.000 56.57

17533000.000 40.21625 162330000.000 56.58

17540000.000 40.21946 162400000.000 56.58

17547000.000 40.22267 162470000.000 56.59

17554000.000 40.22588 162540000.000 56.59

17561000.000 40.22908 162610000.000 56.60

17568000.000 40.23229 162680000.000 56.60

17575000.000 40.23550 162750000.000 56.61

17582000.000 40.23870 162820000.000 56.61

17589000.000 40.24190 162890000.000 56.62

17596000.000 40.24511 162960000.000 56.62

17603000.000 40.24831 163030000.000 56.63

17610000.000 40.25151 163100000.000 56.63

17617000.000 40.25471 163170000.000 56.64

17624000.000 40.25791 163240000.000 56.64

17631000.000 40.26110 163310000.000 56.64

17638000.000 40.26430 163380000.000 56.65

17645000.000 40.26750 163450000.000 56.65

17652000.000 40.27069 163520000.000 56.66

17659000.000 40.27388 163590000.000 56.66

17666000.000 40.27708 163660000.000 56.67

17673000.000 40.28027 163730000.000 56.67

17680000.000 40.28346 163800000.000 56.68

17687000.000 40.28665 163870000.000 56.68

17694000.000 40.28983 163940000.000 56.69

17701000.000 40.29302 164010000.000 56.69

17708000.000 40.29621 164080000.000 56.70

17715000.000 40.29939 164150000.000 56.70

17722000.000 40.30258 164220000.000 56.71

17729000.000 40.30576 164290000.000 56.71

17736000.000 40.30894 164360000.000 56.72

17743000.000 40.31213 164430000.000 56.72

17750000.000 40.31531 164500000.000 56.73

17757000.000 40.31848 164570000.000 56.73

17764000.000 40.32166 164640000.000 56.74

17771000.000 40.32484 164710000.000 56.74

17778000.000 40.32802 164780000.000 56.75

17785000.000 40.33119 164850000.000 56.75

17792000.000 40.33437 164920000.000 56.76

17799000.000 40.33754 164990000.000 56.76

17806000.000 40.34071 165060000.000 56.77

17813000.000 40.34388 165130000.000 56.77

17820000.000 40.34705 165200000.000 56.78

17827000.000 40.35022 165270000.000 56.78

17834000.000 40.35339 165340000.000 56.78

17841000.000 40.35656 165410000.000 56.79

17848000.000 40.35972 165480000.000 56.79

17855000.000 40.36289 165550000.000 56.80

17862000.000 40.36605 165620000.000 56.80

17869000.000 40.36922 165690000.000 56.81

17876000.000 40.37238 165760000.000 56.81

17883000.000 40.37554 165830000.000 56.82

17890000.000 40.37870 165900000.000 56.82

17897000.000 40.38186 165970000.000 56.83

17904000.000 40.38502 166040000.000 56.83

17911000.000 40.38818 166110000.000 56.84

17918000.000 40.39133 166180000.000 56.84

17925000.000 40.39449 166250000.000 56.85

17932000.000 40.39764 166320000.000 56.85

17939000.000 40.40080 166390000.000 56.86

17946000.000 40.40395 166460000.000 56.86

17953000.000 40.40710 166530000.000 56.87

17960000.000 40.41025 166600000.000 56.87

17967000.000 40.41340 166670000.000 56.88

17974000.000 40.41655 166740000.000 56.88

17981000.000 40.41970 166810000.000 56.89

17988000.000 40.42284 166880000.000 56.89

17995000.000 40.42599 166950000.000 56.90

18002000.000 40.42913 167020000.000 56.90

18009000.000 40.43228 167090000.000 56.90

18016000.000 40.43542 167160000.000 56.91

18023000.000 40.43856 167230000.000 56.91

18030000.000 40.44170 167300000.000 56.92

18037000.000 40.44484 167370000.000 56.92

18044000.000 40.44798 167440000.000 56.93

18051000.000 40.45112 167510000.000 56.93

18058000.000 40.45426 167580000.000 56.94

18065000.000 40.45739 167650000.000 56.94

18072000.000 40.46053 167720000.000 56.95

18079000.000 40.46366 167790000.000 56.95

18086000.000 40.46679 167860000.000 56.96

18093000.000 40.46993 167930000.000 56.96

18100000.000 40.47306 168000000.000 56.97

18107000.000 40.47619 168070000.000 56.97

18114000.000 40.47932 168140000.000 56.98

18121000.000 40.48244 168210000.000 56.98

18128000.000 40.48557 168280000.000 56.99

18135000.000 40.48870 168350000.000 56.99

18142000.000 40.49182 168420000.000 56.99

18149000.000 40.49495 168490000.000 57.00

18156000.000 40.49807 168560000.000 57.00

18163000.000 40.50119 168630000.000 57.01

18170000.000 40.50431 168700000.000 57.01

18177000.000 40.50743 168770000.000 57.02

18184000.000 40.51055 168840000.000 57.02

18191000.000 40.51367 168910000.000 57.03

18198000.000 40.51679 168980000.000 57.03

18205000.000 40.51991 169050000.000 57.04

18212000.000 40.52302 169120000.000 57.04

18219000.000 40.52614 169190000.000 57.05

18226000.000 40.52925 169260000.000 57.05

18233000.000 40.53236 169330000.000 57.06

18240000.000 40.53547 169400000.000 57.06

18247000.000 40.53858 169470000.000 57.07

18254000.000 40.54169 169540000.000 57.07

18261000.000 40.54480 169610000.000 57.08

18268000.000 40.54791 169680000.000 57.08

18275000.000 40.55102 169750000.000 57.08

18282000.000 40.55412 169820000.000 57.09

18289000.000 40.55723 169890000.000 57.09

18296000.000 40.56033 169960000.000 57.10

18303000.000 40.56344 170030000.000 57.10

18310000.000 40.56654 170100000.000 57.11

18317000.000 40.56964 170170000.000 57.11

18324000.000 40.57274 170240000.000 57.12

18331000.000 40.57584 170310000.000 57.12

18338000.000 40.57894 170380000.000 57.13

18345000.000 40.58204 170450000.000 57.13

18352000.000 40.58513 170520000.000 57.14

18359000.000 40.58823 170590000.000 57.14

18366000.000 40.59132 170660000.000 57.15

18373000.000 40.59442 170730000.000 57.15

18380000.000 40.59751 170800000.000 57.16

18387000.000 40.60060 170870000.000 57.16

18394000.000 40.60369 170940000.000 57.16

18401000.000 40.60678 171010000.000 57.17

18408000.000 40.60987 171080000.000 57.17

18415000.000 40.61296 171150000.000 57.18

18422000.000 40.61605 171220000.000 57.18

18429000.000 40.61913 171290000.000 57.19

18436000.000 40.62222 171360000.000 57.19

18443000.000 40.62530 171430000.000 57.20

18450000.000 40.62838 171500000.000 57.20

18457000.000 40.63147 171570000.000 57.21

18464000.000 40.63455 171640000.000 57.21

18471000.000 40.63763 171710000.000 57.22

18478000.000 40.64071 171780000.000 57.22

18485000.000 40.64379 171850000.000 57.23

18492000.000 40.64687 171920000.000 57.23

18499000.000 40.64994 171990000.000 57.23

18506000.000 40.65302 172060000.000 57.24

18513000.000 40.65609 172130000.000 57.24

18520000.000 40.65917 172200000.000 57.25

18527000.000 40.66224 172270000.000 57.25

18534000.000 40.66531 172340000.000 57.26

18541000.000 40.66838 172410000.000 57.26

18548000.000 40.67145 172480000.000 57.27

18555000.000 40.67452 172550000.000 57.27

18562000.000 40.67759 172620000.000 57.28

18569000.000 40.68066 172690000.000 57.28

18576000.000 40.68373 172760000.000 57.29

18583000.000 40.68679 172830000.000 57.29

18590000.000 40.68986 172900000.000 57.30

18597000.000 40.69292 172970000.000 57.30

18604000.000 40.69598 173040000.000 57.30

18611000.000 40.69905 173110000.000 57.31

18618000.000 40.70211 173180000.000 57.31

18625000.000 40.70517 173250000.000 57.32

18632000.000 40.70823 173320000.000 57.32

18639000.000 40.71128 173390000.000 57.33

18646000.000 40.71434 173460000.000 57.33

18653000.000 40.71740 173530000.000 57.34

18660000.000 40.72045 173600000.000 57.34

18667000.000 40.72351 173670000.000 57.35

18674000.000 40.72656 173740000.000 57.35

18681000.000 40.72962 173810000.000 57.36

18688000.000 40.73267 173880000.000 57.36

18695000.000 40.73572 173950000.000 57.36

18702000.000 40.73877 174020000.000 57.37

18709000.000 40.74182 174090000.000 57.37

18716000.000 40.74487 174160000.000 57.38

18723000.000 40.74791 174230000.000 57.38

18730000.000 40.75096 174300000.000 57.39

18737000.000 40.75401 174370000.000 57.39

18744000.000 40.75705 174440000.000 57.40

18751000.000 40.76009 174510000.000 57.40

18758000.000 40.76314 174580000.000 57.41

18765000.000 40.76618 174650000.000 57.41

18772000.000 40.76922 174720000.000 57.42

18779000.000 40.77226 174790000.000 57.42

18786000.000 40.77530 174860000.000 57.42

18793000.000 40.77834 174930000.000 57.43

18800000.000 40.78137 175000000.000 57.43

18807000.000 40.78441 175070000.000 57.44

18814000.000 40.78745 175140000.000 57.44

18821000.000 40.79048 175210000.000 57.45

18828000.000 40.79351 175280000.000 57.45

18835000.000 40.79655 175350000.000 57.46

18842000.000 40.79958 175420000.000 57.46

18849000.000 40.80261 175490000.000 57.47

18856000.000 40.80564 175560000.000 57.47

18863000.000 40.80867 175630000.000 57.47

18870000.000 40.81170 175700000.000 57.48

18877000.000 40.81473 175770000.000 57.48

18884000.000 40.81775 175840000.000 57.49

18891000.000 40.82078 175910000.000 57.49

18898000.000 40.82380 175980000.000 57.50

18905000.000 40.82683 176050000.000 57.50

18912000.000 40.82985 176120000.000 57.51

18919000.000 40.83287 176190000.000 57.51

18926000.000 40.83589 176260000.000 57.52

18933000.000 40.83891 176330000.000 57.52

18940000.000 40.84193 176400000.000 57.53

18947000.000 40.84495 176470000.000 57.53

18954000.000 40.84797 176540000.000 57.53

18961000.000 40.85098 176610000.000 57.54

18968000.000 40.85400 176680000.000 57.54

18975000.000 40.85702 176750000.000 57.55

18982000.000 40.86003 176820000.000 57.55

18989000.000 40.86304 176890000.000 57.56

18996000.000 40.86606 176960000.000 57.56

19003000.000 40.86907 177030000.000 57.57

19010000.000 40.87208 177100000.000 57.57

19017000.000 40.87509 177170000.000 57.58

19024000.000 40.87810 177240000.000 57.58

19031000.000 40.88110 177310000.000 57.58

19038000.000 40.88411 177380000.000 57.59

19045000.000 40.88712 177450000.000 57.59

19052000.000 40.89012 177520000.000 57.60

19059000.000 40.89313 177590000.000 57.60

19066000.000 40.89613 177660000.000 57.61

19073000.000 40.89913 177730000.000 57.61

19080000.000 40.90213 177800000.000 57.62

19087000.000 40.90513 177870000.000 57.62

19094000.000 40.90813 177940000.000 57.63

19101000.000 40.91113 178010000.000 57.63

19108000.000 40.91413 178080000.000 57.63

19115000.000 40.91713 178150000.000 57.64

19122000.000 40.92012 178220000.000 57.64

19129000.000 40.92312 178290000.000 57.65

19136000.000 40.92611 178360000.000 57.65

19143000.000 40.92911 178430000.000 57.66

19150000.000 40.93210 178500000.000 57.66

19157000.000 40.93509 178570000.000 57.67

19164000.000 40.93808 178640000.000 57.67

19171000.000 40.94107 178710000.000 57.68

19178000.000 40.94406 178780000.000 57.68

19185000.000 40.94705 178850000.000 57.68

19192000.000 40.95004 178920000.000 57.69

19199000.000 40.95303 178990000.000 57.69

19206000.000 40.95601 179060000.000 57.70

19213000.000 40.95900 179130000.000 57.70

19220000.000 40.96198 179200000.000 57.71

19227000.000 40.96497 179270000.000 57.71

19234000.000 40.96795 179340000.000 57.72

19241000.000 40.97093 179410000.000 57.72

19248000.000 40.97391 179480000.000 57.72

19255000.000 40.97689 179550000.000 57.73

19262000.000 40.97987 179620000.000 57.73

19269000.000 40.98285 179690000.000 57.74

19276000.000 40.98582 179760000.000 57.74

19283000.000 40.98880 179830000.000 57.75

19290000.000 40.99178 179900000.000 57.75

19297000.000 40.99475 179970000.000 57.76

19304000.000 40.99772 180040000.000 57.76

19311000.000 41.00070 180110000.000 57.77

19318000.000 41.00367 180180000.000 57.77

19325000.000 41.00664 180250000.000 57.77

19332000.000 41.00961 180320000.000 57.78

19339000.000 41.01258 180390000.000 57.78

19346000.000 41.01555 180460000.000 57.79

19353000.000 41.01852 180530000.000 57.79

19360000.000 41.02148 180600000.000 57.80

19367000.000 41.02445 180670000.000 57.80

19374000.000 41.02741 180740000.000 57.81

19381000.000 41.03038 180810000.000 57.81

19388000.000 41.03334 180880000.000 57.81

19395000.000 41.03630 180950000.000 57.82

19402000.000 41.03927 181020000.000 57.82

19409000.000 41.04223 181090000.000 57.83

19416000.000 41.04519 181160000.000 57.83

19423000.000 41.04815 181230000.000 57.84

19430000.000 41.05110 181300000.000 57.84

19437000.000 41.05406 181370000.000 57.85

19444000.000 41.05702 181440000.000 57.85

19451000.000 41.05997 181510000.000 57.85

19458000.000 41.06293 181580000.000 57.86

19465000.000 41.06588 181650000.000 57.86

19472000.000 41.06884 181720000.000 57.87

19479000.000 41.07179 181790000.000 57.87

19486000.000 41.07474 181860000.000 57.88

19493000.000 41.07769 181930000.000 57.88

19500000.000 41.08064 182000000.000 57.89

19507000.000 41.08359 182070000.000 57.89

19514000.000 41.08654 182140000.000 57.89

19521000.000 41.08948 182210000.000 57.90

19528000.000 41.09243 182280000.000 57.90

19535000.000 41.09538 182350000.000 57.91

19542000.000 41.09832 182420000.000 57.91

19549000.000 41.10127 182490000.000 57.92

19556000.000 41.10421 182560000.000 57.92

19563000.000 41.10715 182630000.000 57.93

19570000.000 41.11009 182700000.000 57.93

19577000.000 41.11303 182770000.000 57.93

19584000.000 41.11597 182840000.000 57.94

19591000.000 41.11891 182910000.000 57.94

19598000.000 41.12185 182980000.000 57.95

19605000.000 41.12479 183050000.000 57.95

19612000.000 41.12772 183120000.000 57.96

19619000.000 41.13066 183190000.000 57.96

19626000.000 41.13359 183260000.000 57.97

19633000.000 41.13653 183330000.000 57.97

19640000.000 41.13946 183400000.000 57.97

19647000.000 41.14239 183470000.000 57.98

19654000.000 41.14532 183540000.000 57.98

19661000.000 41.14825 183610000.000 57.99

19668000.000 41.15118 183680000.000 57.99

19675000.000 41.15411 183750000.000 58.00

19682000.000 41.15704 183820000.000 58.00

19689000.000 41.15997 183890000.000 58.01

19696000.000 41.16289 183960000.000 58.01

19703000.000 41.16582 184030000.000 58.01

19710000.000 41.16874 184100000.000 58.02

19717000.000 41.17167 184170000.000 58.02

19724000.000 41.17459 184240000.000 58.03

19731000.000 41.17751 184310000.000 58.03

19738000.000 41.18043 184380000.000 58.04

19745000.000 41.18335 184450000.000 58.04

19752000.000 41.18627 184520000.000 58.05

19759000.000 41.18919 184590000.000 58.05

19766000.000 41.19211 184660000.000 58.05

19773000.000 41.19503 184730000.000 58.06

19780000.000 41.19794 184800000.000 58.06

19787000.000 41.20086 184870000.000 58.07

19794000.000 41.20377 184940000.000 58.07

19801000.000 41.20669 185010000.000 58.08

19808000.000 41.20960 185080000.000 58.08

19815000.000 41.21251 185150000.000 58.08

19822000.000 41.21542 185220000.000 58.09

19829000.000 41.21834 185290000.000 58.09

19836000.000 41.22124 185360000.000 58.10

19843000.000 41.22415 185430000.000 58.10

19850000.000 41.22706 185500000.000 58.11

19857000.000 41.22997 185570000.000 58.11

19864000.000 41.23288 185640000.000 58.12

19871000.000 41.23578 185710000.000 58.12

19878000.000 41.23869 185780000.000 58.12

19885000.000 41.24159 185850000.000 58.13

19892000.000 41.24449 185920000.000 58.13

19899000.000 41.24740 185990000.000 58.14

19906000.000 41.25030 186060000.000 58.14

19913000.000 41.25320 186130000.000 58.15

19920000.000 41.25610 186200000.000 58.15

19927000.000 41.25900 186270000.000 58.15

19934000.000 41.26190 186340000.000 58.16

19941000.000 41.26479 186410000.000 58.16

19948000.000 41.26769 186480000.000 58.17

19955000.000 41.27059 186550000.000 58.17

19962000.000 41.27348 186620000.000 58.18

19969000.000 41.27637 186690000.000 58.18

19976000.000 41.27927 186760000.000 58.19

19983000.000 41.28216 186830000.000 58.19

19990000.000 41.28505 186900000.000 58.19

19997000.000 41.28794 186970000.000 58.20

20004000.000 41.29083 187040000.000 58.20

20011000.000 41.29372 187110000.000 58.21

20018000.000 41.29661 187180000.000 58.21

20025000.000 41.29950 187250000.000 58.22

20032000.000 41.30239 187320000.000 58.22

20039000.000 41.30527 187390000.000 58.22

20046000.000 41.30816 187460000.000 58.23

20053000.000 41.31104 187530000.000 58.23

20060000.000 41.31393 187600000.000 58.24

20067000.000 41.31681 187670000.000 58.24

20074000.000 41.31969 187740000.000 58.25

20081000.000 41.32257 187810000.000 58.25

20088000.000 41.32545 187880000.000 58.26

20095000.000 41.32833 187950000.000 58.26

20102000.000 41.33121 188020000.000 58.26

20109000.000 41.33409 188090000.000 58.27

20116000.000 41.33697 188160000.000 58.27

20123000.000 41.33984 188230000.000 58.28

20130000.000 41.34272 188300000.000 58.28

20137000.000 41.34559 188370000.000 58.29

20144000.000 41.34847 188440000.000 58.29

20151000.000 41.35134 188510000.000 58.29

20158000.000 41.35421 188580000.000 58.30

20165000.000 41.35709 188650000.000 58.30

20172000.000 41.35996 188720000.000 58.31

20179000.000 41.36283 188790000.000 58.31

20186000.000 41.36570 188860000.000 58.32

20193000.000 41.36856 188930000.000 58.32

20200000.000 41.37143 189000000.000 58.32

20207000.000 41.37430 189070000.000 58.33

20214000.000 41.37717 189140000.000 58.33

20221000.000 41.38003 189210000.000 58.34

20228000.000 41.38290 189280000.000 58.34

20235000.000 41.38576 189350000.000 58.35

20242000.000 41.38862 189420000.000 58.35

20249000.000 41.39148 189490000.000 58.35

20256000.000 41.39435 189560000.000 58.36

20263000.000 41.39721 189630000.000 58.36

20270000.000 41.40007 189700000.000 58.37

20277000.000 41.40292 189770000.000 58.37

20284000.000 41.40578 189840000.000 58.38

20291000.000 41.40864 189910000.000 58.38

20298000.000 41.41150 189980000.000 58.38

20305000.000 41.41435 190050000.000 58.39

20312000.000 41.41721 190120000.000 58.39

20319000.000 41.42006 190190000.000 58.40

20326000.000 41.42292 190260000.000 58.40

20333000.000 41.42577 190330000.000 58.41

20340000.000 41.42862 190400000.000 58.41

20347000.000 41.43147 190470000.000 58.41

20354000.000 41.43432 190540000.000 58.42

20361000.000 41.43717 190610000.000 58.42

20368000.000 41.44002 190680000.000 58.43

20375000.000 41.44287 190750000.000 58.43

20382000.000 41.44572 190820000.000 58.44

20389000.000 41.44856 190890000.000 58.44

20396000.000 41.45141 190960000.000 58.44

20403000.000 41.45425 191030000.000 58.45

20410000.000 41.45710 191100000.000 58.45

20417000.000 41.45994 191170000.000 58.46

20424000.000 41.46278 191240000.000 58.46

20431000.000 41.46562 191310000.000 58.47

20438000.000 41.46847 191380000.000 58.47

20445000.000 41.47131 191450000.000 58.47

20452000.000 41.47414 191520000.000 58.48

20459000.000 41.47698 191590000.000 58.48

20466000.000 41.47982 191660000.000 58.49

20473000.000 41.48266 191730000.000 58.49

20480000.000 41.48549 191800000.000 58.50

20487000.000 41.48833 191870000.000 58.50

20494000.000 41.49117 191940000.000 58.50

20501000.000 41.49400 192010000.000 58.51

20508000.000 41.49683 192080000.000 58.51

20515000.000 41.49966 192150000.000 58.52

20522000.000 41.50250 192220000.000 58.52

20529000.000 41.50533 192290000.000 58.53

20536000.000 41.50816 192360000.000 58.53

20543000.000 41.51099 192430000.000 58.53

20550000.000 41.51382 192500000.000 58.54

20557000.000 41.51664 192570000.000 58.54

20564000.000 41.51947 192640000.000 58.55

20571000.000 41.52230 192710000.000 58.55

20578000.000 41.52512 192780000.000 58.56

20585000.000 41.52795 192850000.000 58.56

20592000.000 41.53077 192920000.000 58.56

20599000.000 41.53359 192990000.000 58.57

20606000.000 41.53642 193060000.000 58.57

20613000.000 41.53924 193130000.000 58.58

20620000.000 41.54206 193200000.000 58.58

20627000.000 41.54488 193270000.000 58.59

20634000.000 41.54770 193340000.000 58.59

20641000.000 41.55052 193410000.000 58.59

20648000.000 41.55333 193480000.000 58.60

20655000.000 41.55615 193550000.000 58.60

20662000.000 41.55897 193620000.000 58.61

20669000.000 41.56178 193690000.000 58.61

20676000.000 41.56460 193760000.000 58.62

20683000.000 41.56741 193830000.000 58.62

20690000.000 41.57023 193900000.000 58.62

20697000.000 41.57304 193970000.000 58.63

20704000.000 41.57585 194040000.000 58.63

20711000.000 41.57866 194110000.000 58.64

20718000.000 41.58147 194180000.000 58.64

20725000.000 41.58428 194250000.000 58.64

20732000.000 41.58709 194320000.000 58.65

20739000.000 41.58990 194390000.000 58.65

20746000.000 41.59270 194460000.000 58.66

20753000.000 41.59551 194530000.000 58.66

20760000.000 41.59832 194600000.000 58.67

20767000.000 41.60112 194670000.000 58.67

20774000.000 41.60393 194740000.000 58.67

20781000.000 41.60673 194810000.000 58.68

20788000.000 41.60953 194880000.000 58.68

20795000.000 41.61233 194950000.000 58.69

20802000.000 41.61513 195020000.000 58.69

20809000.000 41.61793 195090000.000 58.70

20816000.000 41.62073 195160000.000 58.70

20823000.000 41.62353 195230000.000 58.70

20830000.000 41.62633 195300000.000 58.71

20837000.000 41.62913 195370000.000 58.71

20844000.000 41.63193 195440000.000 58.72

20851000.000 41.63472 195510000.000 58.72

20858000.000 41.63752 195580000.000 58.72

20865000.000 41.64031 195650000.000 58.73

20872000.000 41.64310 195720000.000 58.73

20879000.000 41.64590 195790000.000 58.74

20886000.000 41.64869 195860000.000 58.74

20893000.000 41.65148 195930000.000 58.75

20900000.000 41.65427 196000000.000 58.75

20907000.000 41.65706 196070000.000 58.75

20914000.000 41.65985 196140000.000 58.76

20921000.000 41.66264 196210000.000 58.76

20928000.000 41.66543 196280000.000 58.77

20935000.000 41.66821 196350000.000 58.77

20942000.000 41.67100 196420000.000 58.78

20949000.000 41.67378 196490000.000 58.78

20956000.000 41.67657 196560000.000 58.78

20963000.000 41.67935 196630000.000 58.79

20970000.000 41.68214 196700000.000 58.79

20977000.000 41.68492 196770000.000 58.80

20984000.000 41.68770 196840000.000 58.80

20991000.000 41.69048 196910000.000 58.80

20998000.000 41.69326 196980000.000 58.81

21005000.000 41.69604 197050000.000 58.81

21012000.000 41.69882 197120000.000 58.82

21019000.000 41.70160 197190000.000 58.82

21026000.000 41.70437 197260000.000 58.83

21033000.000 41.70715 197330000.000 58.83

21040000.000 41.70993 197400000.000 58.83

21047000.000 41.71270 197470000.000 58.84

21054000.000 41.71548 197540000.000 58.84

21061000.000 41.71825 197610000.000 58.85

21068000.000 41.72102 197680000.000 58.85

21075000.000 41.72379 197750000.000 58.85

21082000.000 41.72657 197820000.000 58.86

21089000.000 41.72934 197890000.000 58.86

21096000.000 41.73211 197960000.000 58.87

21103000.000 41.73488 198030000.000 58.87

21110000.000 41.73764 198100000.000 58.88

21117000.000 41.74041 198170000.000 58.88

21124000.000 41.74318 198240000.000 58.88

21131000.000 41.74594 198310000.000 58.89

21138000.000 41.74871 198380000.000 58.89

21145000.000 41.75147 198450000.000 58.90

21152000.000 41.75424 198520000.000 58.90

21159000.000 41.75700 198590000.000 58.90

21166000.000 41.75976 198660000.000 58.91

21173000.000 41.76253 198730000.000 58.91

21180000.000 41.76529 198800000.000 58.92

21187000.000 41.76805 198870000.000 58.92

21194000.000 41.77081 198940000.000 58.93

21201000.000 41.77357 199010000.000 58.93

21208000.000 41.77632 199080000.000 58.93

21215000.000 41.77908 199150000.000 58.94

21222000.000 41.78184 199220000.000 58.94

21229000.000 41.78459 199290000.000 58.95

21236000.000 41.78735 199360000.000 58.95

21243000.000 41.79010 199430000.000 58.95

21250000.000 41.79286 199500000.000 58.96

21257000.000 41.79561 199570000.000 58.96

21264000.000 41.79836 199640000.000 58.97

21271000.000 41.80111 199710000.000 58.97

21278000.000 41.80387 199780000.000 58.98

21285000.000 41.80662 199850000.000 58.98

21292000.000 41.80937 199920000.000 58.98

21299000.000 41.81211 199990000.000 58.99

21306000.000 41.81486 200060000.000 58.99

21313000.000 41.81761 200130000.000 59.00

21320000.000 41.82036 200200000.000 59.00

21327000.000 41.82310 200270000.000 59.00

21334000.000 41.82585 200340000.000 59.01

21341000.000 41.82859 200410000.000 59.01

21348000.000 41.83133 200480000.000 59.02

21355000.000 41.83408 200550000.000 59.02

21362000.000 41.83682 200620000.000 59.02

21369000.000 41.83956 200690000.000 59.03

21376000.000 41.84230 200760000.000 59.03

21383000.000 41.84504 200830000.000 59.04

21390000.000 41.84778 200900000.000 59.04

21397000.000 41.85052 200970000.000 59.05

21404000.000 41.85326 201040000.000 59.05

21411000.000 41.85600 201110000.000 59.05

21418000.000 41.85873 201180000.000 59.06

21425000.000 41.86147 201250000.000 59.06

21432000.000 41.86420 201320000.000 59.07

21439000.000 41.86694 201390000.000 59.07

21446000.000 41.86967 201460000.000 59.07

21453000.000 41.87240 201530000.000 59.08

21460000.000 41.87514 201600000.000 59.08

21467000.000 41.87787 201670000.000 59.09

21474000.000 41.88060 201740000.000 59.09

21481000.000 41.88333 201810000.000 59.09

21488000.000 41.88606 201880000.000 59.10

21495000.000 41.88879 201950000.000 59.10

21502000.000 41.89151 202020000.000 59.11

21509000.000 41.89424 202090000.000 59.11

21516000.000 41.89697 202160000.000 59.11

21523000.000 41.89969 202230000.000 59.12

21530000.000 41.90242 202300000.000 59.12

21537000.000 41.90514 202370000.000 59.13

21544000.000 41.90787 202440000.000 59.13

21551000.000 41.91059 202510000.000 59.14

21558000.000 41.91331 202580000.000 59.14

21565000.000 41.91603 202650000.000 59.14

21572000.000 41.91875 202720000.000 59.15

21579000.000 41.92147 202790000.000 59.15

21586000.000 41.92419 202860000.000 59.16

21593000.000 41.92691 202930000.000 59.16

21600000.000 41.92963 203000000.000 59.16

21607000.000 41.93235 203070000.000 59.17

21614000.000 41.93506 203140000.000 59.17

21621000.000 41.93778 203210000.000 59.18

21628000.000 41.94050 203280000.000 59.18

21635000.000 41.94321 203350000.000 59.18

21642000.000 41.94592 203420000.000 59.19

21649000.000 41.94864 203490000.000 59.19

21656000.000 41.95135 203560000.000 59.20

21663000.000 41.95406 203630000.000 59.20

21670000.000 41.95677 203700000.000 59.20

21677000.000 41.95948 203770000.000 59.21

21684000.000 41.96219 203840000.000 59.21

21691000.000 41.96490 203910000.000 59.22

21698000.000 41.96761 203980000.000 59.22

21705000.000 41.97032 204050000.000 59.23

21712000.000 41.97302 204120000.000 59.23

21719000.000 41.97573 204190000.000 59.23

21726000.000 41.97843 204260000.000 59.24

21733000.000 41.98114 204330000.000 59.24

21740000.000 41.98384 204400000.000 59.25

21747000.000 41.98655 204470000.000 59.25

21754000.000 41.98925 204540000.000 59.25

21761000.000 41.99195 204610000.000 59.26

21768000.000 41.99465 204680000.000 59.26

21775000.000 41.99735 204750000.000 59.27

21782000.000 42.00005 204820000.000 59.27

21789000.000 42.00275 204890000.000 59.27

21796000.000 42.00545 204960000.000 59.28

21803000.000 42.00815 205030000.000 59.28

21810000.000 42.01084 205100000.000 59.29

21817000.000 42.01354 205170000.000 59.29

21824000.000 42.01624 205240000.000 59.29

21831000.000 42.01893 205310000.000 59.30

21838000.000 42.02163 205380000.000 59.30

21845000.000 42.02432 205450000.000 59.31

21852000.000 42.02701 205520000.000 59.31

21859000.000 42.02970 205590000.000 59.31

21866000.000 42.03240 205660000.000 59.32

21873000.000 42.03509 205730000.000 59.32

21880000.000 42.03778 205800000.000 59.33

21887000.000 42.04047 205870000.000 59.33

21894000.000 42.04316 205940000.000 59.33

21901000.000 42.04584 206010000.000 59.34

21908000.000 42.04853 206080000.000 59.34

21915000.000 42.05122 206150000.000 59.35

21922000.000 42.05390 206220000.000 59.35

21929000.000 42.05659 206290000.000 59.35

21936000.000 42.05927 206360000.000 59.36

21943000.000 42.06196 206430000.000 59.36

21950000.000 42.06464 206500000.000 59.37

21957000.000 42.06732 206570000.000 59.37

21964000.000 42.07001 206640000.000 59.37

21971000.000 42.07269 206710000.000 59.38

21978000.000 42.07537 206780000.000 59.38

21985000.000 42.07805 206850000.000 59.39

21992000.000 42.08073 206920000.000 59.39

21999000.000 42.08340 206990000.000 59.39

22006000.000 42.08608 207060000.000 59.40

22013000.000 42.08876 207130000.000 59.40

22020000.000 42.09144 207200000.000 59.41

22027000.000 42.09411 207270000.000 59.41

22034000.000 42.09679 207340000.000 59.41

22041000.000 42.09946 207410000.000 59.42

22048000.000 42.10214 207480000.000 59.42

22055000.000 42.10481 207550000.000 59.43

22062000.000 42.10748 207620000.000 59.43

22069000.000 42.11015 207690000.000 59.43

22076000.000 42.11282 207760000.000 59.44

22083000.000 42.11549 207830000.000 59.44

22090000.000 42.11816 207900000.000 59.45

22097000.000 42.12083 207970000.000 59.45

22104000.000 42.12350 208040000.000 59.45

22111000.000 42.12617 208110000.000 59.46

22118000.000 42.12884 208180000.000 59.46

22125000.000 42.13150 208250000.000 59.47

22132000.000 42.13417 208320000.000 59.47

22139000.000 42.13683 208390000.000 59.47

22146000.000 42.13950 208460000.000 59.48

22153000.000 42.14216 208530000.000 59.48

22160000.000 42.14482 208600000.000 59.49

22167000.000 42.14749 208670000.000 59.49

22174000.000 42.15015 208740000.000 59.49

22181000.000 42.15281 208810000.000 59.50

22188000.000 42.15547 208880000.000 59.50

22195000.000 42.15813 208950000.000 59.51

22202000.000 42.16079 209020000.000 59.51

22209000.000 42.16344 209090000.000 59.51

22216000.000 42.16610 209160000.000 59.52

22223000.000 42.16876 209230000.000 59.52

22230000.000 42.17142 209300000.000 59.53

22237000.000 42.17407 209370000.000 59.53

22244000.000 42.17673 209440000.000 59.53

22251000.000 42.17938 209510000.000 59.54

22258000.000 42.18203 209580000.000 59.54

22265000.000 42.18469 209650000.000 59.55

22272000.000 42.18734 209720000.000 59.55

22279000.000 42.18999 209790000.000 59.55

22286000.000 42.19264 209860000.000 59.56

22293000.000 42.19529 209930000.000 59.56

22300000.000 42.19794 210000000.000 59.57

22307000.000 42.20059 210070000.000 59.57

22314000.000 42.20324 210140000.000 59.57

22321000.000 42.20589 210210000.000 59.58

22328000.000 42.20853 210280000.000 59.58

22335000.000 42.21118 210350000.000 59.59

22342000.000 42.21382 210420000.000 59.59

22349000.000 42.21647 210490000.000 59.59

22356000.000 42.21911 210560000.000 59.60

22363000.000 42.22176 210630000.000 59.60

22370000.000 42.22440 210700000.000 59.61

22377000.000 42.22704 210770000.000 59.61

22384000.000 42.22968 210840000.000 59.61

22391000.000 42.23232 210910000.000 59.62

22398000.000 42.23496 210980000.000 59.62

22405000.000 42.23760 211050000.000 59.63

22412000.000 42.24024 211120000.000 59.63

22419000.000 42.24288 211190000.000 59.63

22426000.000 42.24552 211260000.000 59.64

22433000.000 42.24816 211330000.000 59.64

22440000.000 42.25079 211400000.000 59.65

22447000.000 42.25343 211470000.000 59.65

22454000.000 42.25606 211540000.000 59.65

22461000.000 42.25870 211610000.000 59.66

22468000.000 42.26133 211680000.000 59.66

22475000.000 42.26396 211750000.000 59.67

22482000.000 42.26660 211820000.000 59.67

22489000.000 42.26923 211890000.000 59.67

22496000.000 42.27186 211960000.000 59.68

22503000.000 42.27449 212030000.000 59.68

22510000.000 42.27712 212100000.000 59.69

22517000.000 42.27975 212170000.000 59.69

22524000.000 42.28238 212240000.000 59.69

22531000.000 42.28500 212310000.000 59.70

22538000.000 42.28763 212380000.000 59.70

22545000.000 42.29026 212450000.000 59.70

22552000.000 42.29288 212520000.000 59.71

22559000.000 42.29551 212590000.000 59.71

22566000.000 42.29813 212660000.000 59.72

22573000.000 42.30076 212730000.000 59.72

22580000.000 42.30338 212800000.000 59.72

22587000.000 42.30600 212870000.000 59.73

22594000.000 42.30862 212940000.000 59.73

22601000.000 42.31125 213010000.000 59.74

22608000.000 42.31387 213080000.000 59.74

22615000.000 42.31649 213150000.000 59.74

22622000.000 42.31911 213220000.000 59.75

22629000.000 42.32172 213290000.000 59.75

22636000.000 42.32434 213360000.000 59.76

22643000.000 42.32696 213430000.000 59.76

22650000.000 42.32958 213500000.000 59.76

22657000.000 42.33219 213570000.000 59.77

22664000.000 42.33481 213640000.000 59.77

22671000.000 42.33742 213710000.000 59.78

22678000.000 42.34004 213780000.000 59.78

22685000.000 42.34265 213850000.000 59.78

22692000.000 42.34526 213920000.000 59.79

22699000.000 42.34788 213990000.000 59.79

22706000.000 42.35049 214060000.000 59.80

22713000.000 42.35310 214130000.000 59.80

22720000.000 42.35571 214200000.000 59.80

22727000.000 42.35832 214270000.000 59.81

22734000.000 42.36093 214340000.000 59.81

22741000.000 42.36354 214410000.000 59.81

22748000.000 42.36614 214480000.000 59.82

22755000.000 42.36875 214550000.000 59.82

22762000.000 42.37136 214620000.000 59.83

22769000.000 42.37396 214690000.000 59.83

22776000.000 42.37657 214760000.000 59.83

22783000.000 42.37917 214830000.000 59.84

22790000.000 42.38178 214900000.000 59.84

22797000.000 42.38438 214970000.000 59.85

22804000.000 42.38698 215040000.000 59.85

22811000.000 42.38958 215110000.000 59.85

22818000.000 42.39218 215180000.000 59.86

22825000.000 42.39479 215250000.000 59.86

22832000.000 42.39739 215320000.000 59.87

22839000.000 42.39998 215390000.000 59.87

22846000.000 42.40258 215460000.000 59.87

22853000.000 42.40518 215530000.000 59.88

22860000.000 42.40778 215600000.000 59.88

22867000.000 42.41038 215670000.000 59.88

22874000.000 42.41297 215740000.000 59.89

22881000.000 42.41557 215810000.000 59.89

22888000.000 42.41816 215880000.000 59.90

22895000.000 42.42076 215950000.000 59.90

22902000.000 42.42335 216020000.000 59.90

22909000.000 42.42594 216090000.000 59.91

22916000.000 42.42854 216160000.000 59.91

22923000.000 42.43113 216230000.000 59.92

22930000.000 42.43372 216300000.000 59.92

22937000.000 42.43631 216370000.000 59.92

22944000.000 42.43890 216440000.000 59.93

22951000.000 42.44149 216510000.000 59.93

22958000.000 42.44408 216580000.000 59.94

22965000.000 42.44666 216650000.000 59.94

22972000.000 42.44925 216720000.000 59.94

22979000.000 42.45184 216790000.000 59.95

22986000.000 42.45443 216860000.000 59.95

22993000.000 42.45701 216930000.000 59.95

23000000.000 42.45960 217000000.000 59.96

23007000.000 42.46218 217070000.000 59.96

23014000.000 42.46476 217140000.000 59.97

23021000.000 42.46735 217210000.000 59.97

23028000.000 42.46993 217280000.000 59.97

23035000.000 42.47251 217350000.000 59.98

23042000.000 42.47509 217420000.000 59.98

23049000.000 42.47767 217490000.000 59.99

23056000.000 42.48025 217560000.000 59.99

23063000.000 42.48283 217630000.000 59.99

23070000.000 42.48541 217700000.000 60.00

23077000.000 42.48799 217770000.000 60.00

23084000.000 42.49056 217840000.000 60.00

23091000.000 42.49314 217910000.000 60.01

23098000.000 42.49572 217980000.000 60.01

23105000.000 42.49829 218050000.000 60.02

23112000.000 42.50087 218120000.000 60.02

23119000.000 42.50344 218190000.000 60.02

23126000.000 42.50601 218260000.000 60.03

23133000.000 42.50859 218330000.000 60.03

23140000.000 42.51116 218400000.000 60.04

23147000.000 42.51373 218470000.000 60.04

23154000.000 42.51630 218540000.000 60.04

23161000.000 42.51887 218610000.000 60.05

23168000.000 42.52144 218680000.000 60.05

23175000.000 42.52401 218750000.000 60.05

23182000.000 42.52658 218820000.000 60.06

23189000.000 42.52915 218890000.000 60.06

23196000.000 42.53172 218960000.000 60.07

23203000.000 42.53428 219030000.000 60.07

23210000.000 42.53685 219100000.000 60.07

23217000.000 42.53941 219170000.000 60.08

23224000.000 42.54198 219240000.000 60.08

23231000.000 42.54454 219310000.000 60.09

23238000.000 42.54711 219380000.000 60.09

23245000.000 42.54967 219450000.000 60.09

23252000.000 42.55223 219520000.000 60.10

23259000.000 42.55479 219590000.000 60.10

23266000.000 42.55736 219660000.000 60.10

23273000.000 42.55992 219730000.000 60.11

23280000.000 42.56248 219800000.000 60.11

23287000.000 42.56503 219870000.000 60.12

23294000.000 42.56759 219940000.000 60.12

23301000.000 42.57015 220010000.000 60.12

23308000.000 42.57271 220080000.000 60.13

23315000.000 42.57527 220150000.000 60.13

23322000.000 42.57782 220220000.000 60.14

23329000.000 42.58038 220290000.000 60.14

23336000.000 42.58293 220360000.000 60.14

23343000.000 42.58549 220430000.000 60.15

23350000.000 42.58804 220500000.000 60.15

23357000.000 42.59059 220570000.000 60.15

23364000.000 42.59315 220640000.000 60.16

23371000.000 42.59570 220710000.000 60.16

23378000.000 42.59825 220780000.000 60.17

23385000.000 42.60080 220850000.000 60.17

23392000.000 42.60335 220920000.000 60.17

23399000.000 42.60590 220990000.000 60.18

23406000.000 42.60845 221060000.000 60.18

23413000.000 42.61100 221130000.000 60.18

23420000.000 42.61354 221200000.000 60.19

23427000.000 42.61609 221270000.000 60.19

23434000.000 42.61864 221340000.000 60.20

23441000.000 42.62118 221410000.000 60.20

23448000.000 42.62373 221480000.000 60.20

23455000.000 42.62627 221550000.000 60.21

23462000.000 42.62882 221620000.000 60.21

23469000.000 42.63136 221690000.000 60.22

23476000.000 42.63390 221760000.000 60.22

23483000.000 42.63645 221830000.000 60.22

23490000.000 42.63899 221900000.000 60.23

23497000.000 42.64153 221970000.000 60.23

23504000.000 42.64407 222040000.000 60.23

23511000.000 42.64661 222110000.000 60.24

23518000.000 42.64915 222180000.000 60.24

23525000.000 42.65169 222250000.000 60.25

23532000.000 42.65422 222320000.000 60.25

23539000.000 42.65676 222390000.000 60.25

23546000.000 42.65930 222460000.000 60.26

23553000.000 42.66183 222530000.000 60.26

23560000.000 42.66437 222600000.000 60.26

23567000.000 42.66691 222670000.000 60.27

23574000.000 42.66944 222740000.000 60.27

23581000.000 42.67197 222810000.000 60.28

23588000.000 42.67451 222880000.000 60.28

23595000.000 42.67704 222950000.000 60.28

23602000.000 42.67957 223020000.000 60.29

23609000.000 42.68210 223090000.000 60.29

23616000.000 42.68463 223160000.000 60.29

23623000.000 42.68716 223230000.000 60.30

23630000.000 42.68969 223300000.000 60.30

23637000.000 42.69222 223370000.000 60.31

23644000.000 42.69475 223440000.000 60.31

23651000.000 42.69728 223510000.000 60.31

23658000.000 42.69980 223580000.000 60.32

23665000.000 42.70233 223650000.000 60.32

23672000.000 42.70486 223720000.000 60.33

23679000.000 42.70738 223790000.000 60.33

23686000.000 42.70991 223860000.000 60.33

23693000.000 42.71243 223930000.000 60.34

23700000.000 42.71495 224000000.000 60.34

23707000.000 42.71748 224070000.000 60.34

23714000.000 42.72000 224140000.000 60.35

23721000.000 42.72252 224210000.000 60.35

23728000.000 42.72504 224280000.000 60.36

23735000.000 42.72756 224350000.000 60.36

23742000.000 42.73008 224420000.000 60.36

23749000.000 42.73260 224490000.000 60.37

23756000.000 42.73512 224560000.000 60.37

23763000.000 42.73764 224630000.000 60.37

23770000.000 42.74016 224700000.000 60.38

23777000.000 42.74267 224770000.000 60.38

23784000.000 42.74519 224840000.000 60.39

23791000.000 42.74771 224910000.000 60.39

23798000.000 42.75022 224980000.000 60.39

23805000.000 42.75274 225050000.000 60.40

23812000.000 42.75525 225120000.000 60.40

23819000.000 42.75776 225190000.000 60.40

23826000.000 42.76028 225260000.000 60.41

23833000.000 42.76279 225330000.000 60.41

23840000.000 42.76530 225400000.000 60.42

23847000.000 42.76781 225470000.000 60.42

23854000.000 42.77032 225540000.000 60.42

23861000.000 42.77283 225610000.000 60.43

23868000.000 42.77534 225680000.000 60.43

23875000.000 42.77785 225750000.000 60.43

23882000.000 42.78036 225820000.000 60.44

23889000.000 42.78287 225890000.000 60.44

23896000.000 42.78537 225960000.000 60.45

23903000.000 42.78788 226030000.000 60.45

23910000.000 42.79039 226100000.000 60.45

23917000.000 42.79289 226170000.000 60.46

23924000.000 42.79540 226240000.000 60.46

23931000.000 42.79790 226310000.000 60.46

23938000.000 42.80040 226380000.000 60.47

23945000.000 42.80291 226450000.000 60.47

23952000.000 42.80541 226520000.000 60.48

23959000.000 42.80791 226590000.000 60.48

23966000.000 42.81041 226660000.000 60.48

23973000.000 42.81291 226730000.000 60.49

23980000.000 42.81541 226800000.000 60.49

23987000.000 42.81791 226870000.000 60.49

23994000.000 42.82041 226940000.000 60.50

24001000.000 42.82291 227010000.000 60.50

24008000.000 42.82541 227080000.000 60.51

24015000.000 42.82790 227150000.000 60.51

24022000.000 42.83040 227220000.000 60.51

24029000.000 42.83289 227290000.000 60.52

24036000.000 42.83539 227360000.000 60.52

24043000.000 42.83788 227430000.000 60.52

24050000.000 42.84038 227500000.000 60.53

24057000.000 42.84287 227570000.000 60.53

24064000.000 42.84537 227640000.000 60.54

24071000.000 42.84786 227710000.000 60.54

24078000.000 42.85035 227780000.000 60.54

24085000.000 42.85284 227850000.000 60.55

24092000.000 42.85533 227920000.000 60.55

24099000.000 42.85782 227990000.000 60.55

24106000.000 42.86031 228060000.000 60.56

24113000.000 42.86280 228130000.000 60.56

24120000.000 42.86529 228200000.000 60.56

24127000.000 42.86778 228270000.000 60.57

24134000.000 42.87026 228340000.000 60.57

24141000.000 42.87275 228410000.000 60.58

24148000.000 42.87524 228480000.000 60.58

24155000.000 42.87772 228550000.000 60.58

24162000.000 42.88021 228620000.000 60.59

24169000.000 42.88269 228690000.000 60.59

24176000.000 42.88517 228760000.000 60.59

24183000.000 42.88766 228830000.000 60.60

24190000.000 42.89014 228900000.000 60.60

24197000.000 42.89262 228970000.000 60.61

24204000.000 42.89510 229040000.000 60.61

24211000.000 42.89758 229110000.000 60.61

24218000.000 42.90006 229180000.000 60.62

24225000.000 42.90254 229250000.000 60.62

24232000.000 42.90502 229320000.000 60.62

24239000.000 42.90750 229390000.000 60.63

24246000.000 42.90998 229460000.000 60.63

24253000.000 42.91246 229530000.000 60.64

24260000.000 42.91493 229600000.000 60.64

24267000.000 42.91741 229670000.000 60.64

24274000.000 42.91989 229740000.000 60.65

24281000.000 42.92236 229810000.000 60.65

24288000.000 42.92484 229880000.000 60.65

24295000.000 42.92731 229950000.000 60.66

24302000.000 42.92978 230020000.000 60.66

24309000.000 42.93226 230090000.000 60.66

24316000.000 42.93473 230160000.000 60.67

24323000.000 42.93720 230230000.000 60.67

24330000.000 42.93967 230300000.000 60.68

24337000.000 42.94214 230370000.000 60.68

24344000.000 42.94461 230440000.000 60.68

24351000.000 42.94708 230510000.000 60.69

24358000.000 42.94955 230580000.000 60.69

24365000.000 42.95202 230650000.000 60.69

24372000.000 42.95449 230720000.000 60.70

24379000.000 42.95695 230790000.000 60.70

24386000.000 42.95942 230860000.000 60.71

24393000.000 42.96188 230930000.000 60.71

24400000.000 42.96435 231000000.000 60.71

24407000.000 42.96682 231070000.000 60.72

24414000.000 42.96928 231140000.000 60.72

24421000.000 42.97174 231210000.000 60.72

24428000.000 42.97421 231280000.000 60.73

24435000.000 42.97667 231350000.000 60.73

24442000.000 42.97913 231420000.000 60.73

24449000.000 42.98159 231490000.000 60.74

24456000.000 42.98405 231560000.000 60.74

24463000.000 42.98651 231630000.000 60.75

24470000.000 42.98897 231700000.000 60.75

24477000.000 42.99143 231770000.000 60.75

24484000.000 42.99389 231840000.000 60.76

24491000.000 42.99635 231910000.000 60.76

24498000.000 42.99881 231980000.000 60.76

24505000.000 43.00126 232050000.000 60.77

24512000.000 43.00372 232120000.000 60.77

24519000.000 43.00618 232190000.000 60.78

24526000.000 43.00863 232260000.000 60.78

24533000.000 43.01109 232330000.000 60.78

24540000.000 43.01354 232400000.000 60.79

24547000.000 43.01599 232470000.000 60.79

24554000.000 43.01845 232540000.000 60.79

24561000.000 43.02090 232610000.000 60.80

24568000.000 43.02335 232680000.000 60.80

24575000.000 43.02580 232750000.000 60.80

24582000.000 43.02825 232820000.000 60.81

24589000.000 43.03070 232890000.000 60.81

24596000.000 43.03315 232960000.000 60.82

24603000.000 43.03560 233030000.000 60.82

24610000.000 43.03805 233100000.000 60.82

24617000.000 43.04050 233170000.000 60.83

24624000.000 43.04295 233240000.000 60.83

24631000.000 43.04539 233310000.000 60.83

24638000.000 43.04784 233380000.000 60.84

24645000.000 43.05029 233450000.000 60.84

24652000.000 43.05273 233520000.000 60.84

24659000.000 43.05518 233590000.000 60.85

24666000.000 43.05762 233660000.000 60.85

24673000.000 43.06006 233730000.000 60.86

24680000.000 43.06251 233800000.000 60.86

24687000.000 43.06495 233870000.000 60.86

24694000.000 43.06739 233940000.000 60.87

24701000.000 43.06983 234010000.000 60.87

24708000.000 43.07227 234080000.000 60.87

24715000.000 43.07471 234150000.000 60.88

24722000.000 43.07715 234220000.000 60.88

24729000.000 43.07959 234290000.000 60.88

24736000.000 43.08203 234360000.000 60.89

24743000.000 43.08447 234430000.000 60.89

24750000.000 43.08691 234500000.000 60.90

24757000.000 43.08934 234570000.000 60.90

24764000.000 43.09178 234640000.000 60.90

24771000.000 43.09422 234710000.000 60.91

24778000.000 43.09665 234780000.000 60.91

24785000.000 43.09909 234850000.000 60.91

24792000.000 43.10152 234920000.000 60.92

24799000.000 43.10395 234990000.000 60.92

24806000.000 43.10639 235060000.000 60.92

24813000.000 43.10882 235130000.000 60.93

24820000.000 43.11125 235200000.000 60.93

24827000.000 43.11368 235270000.000 60.94

24834000.000 43.11611 235340000.000 60.94

24841000.000 43.11855 235410000.000 60.94

24848000.000 43.12097 235480000.000 60.95

24855000.000 43.12340 235550000.000 60.95

24862000.000 43.12583 235620000.000 60.95

24869000.000 43.12826 235690000.000 60.96

24876000.000 43.13069 235760000.000 60.96

24883000.000 43.13312 235830000.000 60.96

24890000.000 43.13554 235900000.000 60.97

24897000.000 43.13797 235970000.000 60.97

24904000.000 43.14039 236040000.000 60.98

24911000.000 43.14282 236110000.000 60.98

24918000.000 43.14524 236180000.000 60.98

24925000.000 43.14767 236250000.000 60.99

24932000.000 43.15009 236320000.000 60.99

24939000.000 43.15251 236390000.000 60.99

24946000.000 43.15494 236460000.000 61.00

24953000.000 43.15736 236530000.000 61.00

24960000.000 43.15978 236600000.000 61.00

24967000.000 43.16220 236670000.000 61.01

24974000.000 43.16462 236740000.000 61.01

24981000.000 43.16704 236810000.000 61.02

24988000.000 43.16946 236880000.000 61.02

24995000.000 43.17188 236950000.000 61.02

25002000.000 43.17429 237020000.000 61.03

25009000.000 43.17671 237090000.000 61.03

25016000.000 43.17913 237160000.000 61.03

25023000.000 43.18154 237230000.000 61.04

25030000.000 43.18396 237300000.000 61.04

25037000.000 43.18637 237370000.000 61.04

25044000.000 43.18879 237440000.000 61.05

25051000.000 43.19120 237510000.000 61.05

25058000.000 43.19362 237580000.000 61.05

25065000.000 43.19603 237650000.000 61.06

25072000.000 43.19844 237720000.000 61.06

25079000.000 43.20085 237790000.000 61.07

25086000.000 43.20326 237860000.000 61.07

25093000.000 43.20568 237930000.000 61.07

25100000.000 43.20809 238000000.000 61.08

25107000.000 43.21050 238070000.000 61.08

25114000.000 43.21290 238140000.000 61.08

25121000.000 43.21531 238210000.000 61.09

25128000.000 43.21772 238280000.000 61.09

25135000.000 43.22013 238350000.000 61.09

25142000.000 43.22254 238420000.000 61.10

25149000.000 43.22494 238490000.000 61.10

25156000.000 43.22735 238560000.000 61.11

25163000.000 43.22975 238630000.000 61.11

25170000.000 43.23216 238700000.000 61.11

25177000.000 43.23456 238770000.000 61.12

25184000.000 43.23697 238840000.000 61.12

25191000.000 43.23937 238910000.000 61.12

25198000.000 43.24177 238980000.000 61.13

25205000.000 43.24418 239050000.000 61.13

25212000.000 43.24658 239120000.000 61.13

25219000.000 43.24898 239190000.000 61.14

25226000.000 43.25138 239260000.000 61.14

25233000.000 43.25378 239330000.000 61.14

25240000.000 43.25618 239400000.000 61.15

25247000.000 43.25858 239470000.000 61.15

25254000.000 43.26098 239540000.000 61.16

25261000.000 43.26337 239610000.000 61.16

25268000.000 43.26577 239680000.000 61.16

25275000.000 43.26817 239750000.000 61.17

25282000.000 43.27057 239820000.000 61.17

25289000.000 43.27296 239890000.000 61.17

25296000.000 43.27536 239960000.000 61.18

25303000.000 43.27775 240030000.000 61.18

25310000.000 43.28015 240100000.000 61.18

25317000.000 43.28254 240170000.000 61.19

25324000.000 43.28493 240240000.000 61.19

25331000.000 43.28733 240310000.000 61.19

25338000.000 43.28972 240380000.000 61.20

25345000.000 43.29211 240450000.000 61.20

25352000.000 43.29450 240520000.000 61.21

25359000.000 43.29689 240590000.000 61.21

25366000.000 43.29928 240660000.000 61.21

25373000.000 43.30167 240730000.000 61.22

25380000.000 43.30406 240800000.000 61.22

25387000.000 43.30645 240870000.000 61.22

25394000.000 43.30884 240940000.000 61.23

25401000.000 43.31122 241010000.000 61.23

25408000.000 43.31361 241080000.000 61.23

25415000.000 43.31600 241150000.000 61.24

25422000.000 43.31838 241220000.000 61.24

25429000.000 43.32077 241290000.000 61.24

25436000.000 43.32315 241360000.000 61.25

25443000.000 43.32554 241430000.000 61.25

25450000.000 43.32792 241500000.000 61.25

25457000.000 43.33030 241570000.000 61.26

25464000.000 43.33269 241640000.000 61.26

25471000.000 43.33507 241710000.000 61.27

25478000.000 43.33745 241780000.000 61.27

25485000.000 43.33983 241850000.000 61.27

25492000.000 43.34221 241920000.000 61.28

25499000.000 43.34459 241990000.000 61.28

25506000.000 43.34697 242060000.000 61.28

25513000.000 43.34935 242130000.000 61.29

25520000.000 43.35173 242200000.000 61.29

25527000.000 43.35411 242270000.000 61.29

25534000.000 43.35648 242340000.000 61.30

25541000.000 43.35886 242410000.000 61.30

25548000.000 43.36124 242480000.000 61.30

25555000.000 43.36361 242550000.000 61.31

25562000.000 43.36599 242620000.000 61.31

25569000.000 43.36836 242690000.000 61.32

25576000.000 43.37074 242760000.000 61.32

25583000.000 43.37311 242830000.000 61.32

25590000.000 43.37548 242900000.000 61.33

25597000.000 43.37786 242970000.000 61.33

25604000.000 43.38023 243040000.000 61.33

25611000.000 43.38260 243110000.000 61.34

25618000.000 43.38497 243180000.000 61.34

25625000.000 43.38734 243250000.000 61.34

25632000.000 43.38971 243320000.000 61.35

25639000.000 43.39208 243390000.000 61.35

25646000.000 43.39445 243460000.000 61.35

25653000.000 43.39682 243530000.000 61.36

25660000.000 43.39919 243600000.000 61.36

25667000.000 43.40156 243670000.000 61.36

25674000.000 43.40392 243740000.000 61.37

25681000.000 43.40629 243810000.000 61.37

25688000.000 43.40866 243880000.000 61.38

25695000.000 43.41102 243950000.000 61.38

25702000.000 43.41339 244020000.000 61.38

25709000.000 43.41575 244090000.000 61.39

25716000.000 43.41812 244160000.000 61.39

25723000.000 43.42048 244230000.000 61.39

25730000.000 43.42284 244300000.000 61.40

25737000.000 43.42520 244370000.000 61.40

25744000.000 43.42757 244440000.000 61.40

25751000.000 43.42993 244510000.000 61.41

25758000.000 43.43229 244580000.000 61.41

25765000.000 43.43465 244650000.000 61.41

25772000.000 43.43701 244720000.000 61.42

25779000.000 43.43937 244790000.000 61.42

25786000.000 43.44173 244860000.000 61.42

25793000.000 43.44409 244930000.000 61.43

25800000.000 43.44644 245000000.000 61.43

25807000.000 43.44880 245070000.000 61.44

25814000.000 43.45116 245140000.000 61.44

25821000.000 43.45351 245210000.000 61.44

25828000.000 43.45587 245280000.000 61.45

25835000.000 43.45822 245350000.000 61.45

25842000.000 43.46058 245420000.000 61.45

25849000.000 43.46293 245490000.000 61.46

25856000.000 43.46529 245560000.000 61.46

25863000.000 43.46764 245630000.000 61.46

25870000.000 43.46999 245700000.000 61.47

25877000.000 43.47235 245770000.000 61.47

25884000.000 43.47470 245840000.000 61.47

25891000.000 43.47705 245910000.000 61.48

25898000.000 43.47940 245980000.000 61.48

25905000.000 43.48175 246050000.000 61.48

25912000.000 43.48410 246120000.000 61.49

25919000.000 43.48645 246190000.000 61.49

25926000.000 43.48880 246260000.000 61.49

25933000.000 43.49114 246330000.000 61.50

25940000.000 43.49349 246400000.000 61.50

25947000.000 43.49584 246470000.000 61.51

25954000.000 43.49819 246540000.000 61.51

25961000.000 43.50053 246610000.000 61.51

25968000.000 43.50288 246680000.000 61.52

25975000.000 43.50522 246750000.000 61.52

25982000.000 43.50757 246820000.000 61.52

25989000.000 43.50991 246890000.000 61.53

25996000.000 43.51225 246960000.000 61.53

26003000.000 43.51460 247030000.000 61.53

26010000.000 43.51694 247100000.000 61.54

26017000.000 43.51928 247170000.000 61.54

26024000.000 43.52162 247240000.000 61.54

26031000.000 43.52397 247310000.000 61.55

26038000.000 43.52631 247380000.000 61.55

26045000.000 43.52865 247450000.000 61.55

26052000.000 43.53099 247520000.000 61.56

26059000.000 43.53332 247590000.000 61.56

26066000.000 43.53566 247660000.000 61.56

26073000.000 43.53800 247730000.000 61.57

26080000.000 43.54034 247800000.000 61.57

26087000.000 43.54268 247870000.000 61.57

26094000.000 43.54501 247940000.000 61.58

26101000.000 43.54735 248010000.000 61.58

26108000.000 43.54968 248080000.000 61.59

26115000.000 43.55202 248150000.000 61.59

26122000.000 43.55435 248220000.000 61.59

26129000.000 43.55669 248290000.000 61.60

26136000.000 43.55902 248360000.000 61.60

26143000.000 43.56135 248430000.000 61.60

26150000.000 43.56369 248500000.000 61.61

26157000.000 43.56602 248570000.000 61.61

26164000.000 43.56835 248640000.000 61.61

26171000.000 43.57068 248710000.000 61.62

26178000.000 43.57301 248780000.000 61.62

26185000.000 43.57534 248850000.000 61.62

26192000.000 43.57767 248920000.000 61.63

26199000.000 43.58000 248990000.000 61.63

26206000.000 43.58233 249060000.000 61.63

26213000.000 43.58466 249130000.000 61.64

26220000.000 43.58698 249200000.000 61.64

26227000.000 43.58931 249270000.000 61.64

26234000.000 43.59164 249340000.000 61.65

26241000.000 43.59396 249410000.000 61.65

26248000.000 43.59629 249480000.000 61.65

26255000.000 43.59861 249550000.000 61.66

26262000.000 43.60094 249620000.000 61.66

26269000.000 43.60326 249690000.000 61.66

26276000.000 43.60559 249760000.000 61.67

26283000.000 43.60791 249830000.000 61.67

26290000.000 43.61023 249900000.000 61.68

26297000.000 43.61255 249970000.000 61.68

26304000.000 43.61488 250040000.000 61.68

26311000.000 43.61720 250110000.000 61.69

26318000.000 43.61952 250180000.000 61.69

26325000.000 43.62184 250250000.000 61.69

26332000.000 43.62416 250320000.000 61.70

26339000.000 43.62648 250390000.000 61.70

26346000.000 43.62880 250460000.000 61.70

26353000.000 43.63111 250530000.000 61.71

26360000.000 43.63343 250600000.000 61.71

26367000.000 43.63575 250670000.000 61.71

26374000.000 43.63806 250740000.000 61.72

26381000.000 43.64038 250810000.000 61.72

26388000.000 43.64270 250880000.000 61.72

26395000.000 43.64501 250950000.000 61.73

26402000.000 43.64733 251020000.000 61.73

26409000.000 43.64964 251090000.000 61.73

26416000.000 43.65195 251160000.000 61.74

26423000.000 43.65427 251230000.000 61.74

26430000.000 43.65658 251300000.000 61.74

26437000.000 43.65889 251370000.000 61.75

26444000.000 43.66120 251440000.000 61.75

26451000.000 43.66352 251510000.000 61.75

26458000.000 43.66583 251580000.000 61.76

26465000.000 43.66814 251650000.000 61.76

26472000.000 43.67045 251720000.000 61.76

26479000.000 43.67276 251790000.000 61.77

26486000.000 43.67506 251860000.000 61.77

26493000.000 43.67737 251930000.000 61.78

26500000.000 43.67968 252000000.000 61.78

26507000.000 43.68199 252070000.000 61.78

26514000.000 43.68430 252140000.000 61.79

26521000.000 43.68660 252210000.000 61.79

26528000.000 43.68891 252280000.000 61.79

26535000.000 43.69121 252350000.000 61.80

26542000.000 43.69352 252420000.000 61.80

26549000.000 43.69582 252490000.000 61.80

26556000.000 43.69813 252560000.000 61.81

26563000.000 43.70043 252630000.000 61.81

26570000.000 43.70273 252700000.000 61.81

26577000.000 43.70504 252770000.000 61.82

26584000.000 43.70734 252840000.000 61.82

26591000.000 43.70964 252910000.000 61.82

26598000.000 43.71194 252980000.000 61.83

26605000.000 43.71424 253050000.000 61.83

26612000.000 43.71654 253120000.000 61.83

26619000.000 43.71884 253190000.000 61.84

26626000.000 43.72114 253260000.000 61.84

26633000.000 43.72344 253330000.000 61.84

26640000.000 43.72574 253400000.000 61.85

26647000.000 43.72803 253470000.000 61.85

26654000.000 43.73033 253540000.000 61.85

26661000.000 43.73263 253610000.000 61.86

26668000.000 43.73492 253680000.000 61.86

26675000.000 43.73722 253750000.000 61.86

26682000.000 43.73951 253820000.000 61.87

26689000.000 43.74181 253890000.000 61.87

26696000.000 43.74410 253960000.000 61.87

26703000.000 43.74640 254030000.000 61.88

26710000.000 43.74869 254100000.000 61.88

26717000.000 43.75098 254170000.000 61.88

26724000.000 43.75328 254240000.000 61.89

26731000.000 43.75557 254310000.000 61.89

26738000.000 43.75786 254380000.000 61.89

26745000.000 43.76015 254450000.000 61.90

26752000.000 43.76244 254520000.000 61.90

26759000.000 43.76473 254590000.000 61.91

26766000.000 43.76702 254660000.000 61.91

26773000.000 43.76931 254730000.000 61.91

26780000.000 43.77160 254800000.000 61.92

26787000.000 43.77389 254870000.000 61.92

26794000.000 43.77617 254940000.000 61.92

26801000.000 43.77846 255010000.000 61.93

26808000.000 43.78075 255080000.000 61.93

26815000.000 43.78303 255150000.000 61.93

26822000.000 43.78532 255220000.000 61.94

26829000.000 43.78760 255290000.000 61.94

26836000.000 43.78989 255360000.000 61.94

26843000.000 43.79217 255430000.000 61.95

26850000.000 43.79446 255500000.000 61.95

26857000.000 43.79674 255570000.000 61.95

26864000.000 43.79902 255640000.000 61.96

26871000.000 43.80130 255710000.000 61.96

26878000.000 43.80359 255780000.000 61.96

26885000.000 43.80587 255850000.000 61.97

26892000.000 43.80815 255920000.000 61.97

26899000.000 43.81043 255990000.000 61.97

26906000.000 43.81271 256060000.000 61.98

26913000.000 43.81499 256130000.000 61.98

26920000.000 43.81727 256200000.000 61.98

26927000.000 43.81955 256270000.000 61.99

26934000.000 43.82182 256340000.000 61.99

26941000.000 43.82410 256410000.000 61.99

26948000.000 43.82638 256480000.000 62.00

26955000.000 43.82866 256550000.000 62.00

26962000.000 43.83093 256620000.000 62.00

26969000.000 43.83321 256690000.000 62.01

26976000.000 43.83548 256760000.000 62.01

26983000.000 43.83776 256830000.000 62.01

26990000.000 43.84003 256900000.000 62.02

26997000.000 43.84231 256970000.000 62.02

27004000.000 43.84458 257040000.000 62.02

27011000.000 43.84685 257110000.000 62.03

27018000.000 43.84912 257180000.000 62.03

27025000.000 43.85140 257250000.000 62.03

27032000.000 43.85367 257320000.000 62.04

27039000.000 43.85594 257390000.000 62.04

27046000.000 43.85821 257460000.000 62.04

27053000.000 43.86048 257530000.000 62.05

27060000.000 43.86275 257600000.000 62.05

27067000.000 43.86502 257670000.000 62.05

27074000.000 43.86729 257740000.000 62.06

27081000.000 43.86955 257810000.000 62.06

27088000.000 43.87182 257880000.000 62.06

27095000.000 43.87409 257950000.000 62.07

27102000.000 43.87636 258020000.000 62.07

27109000.000 43.87862 258090000.000 62.07

27116000.000 43.88089 258160000.000 62.08

27123000.000 43.88315 258230000.000 62.08

27130000.000 43.88542 258300000.000 62.08

27137000.000 43.88768 258370000.000 62.09

27144000.000 43.88995 258440000.000 62.09

27151000.000 43.89221 258510000.000 62.09

27158000.000 43.89447 258580000.000 62.10

27165000.000 43.89674 258650000.000 62.10

27172000.000 43.89900 258720000.000 62.10

27179000.000 43.90126 258790000.000 62.11

27186000.000 43.90352 258860000.000 62.11

27193000.000 43.90578 258930000.000 62.11

27200000.000 43.90804 259000000.000 62.12

27207000.000 43.91030 259070000.000 62.12

27214000.000 43.91256 259140000.000 62.12

27221000.000 43.91482 259210000.000 62.13

27228000.000 43.91708 259280000.000 62.13

27235000.000 43.91934 259350000.000 62.13

27242000.000 43.92159 259420000.000 62.14

27249000.000 43.92385 259490000.000 62.14

27256000.000 43.92611 259560000.000 62.14

27263000.000 43.92836 259630000.000 62.15

27270000.000 43.93062 259700000.000 62.15

27277000.000 43.93287 259770000.000 62.15

27284000.000 43.93513 259840000.000 62.16

27291000.000 43.93738 259910000.000 62.16

27298000.000 43.93964 259980000.000 62.17

27305000.000 43.94189 260050000.000 62.17

27312000.000 43.94414 260120000.000 62.17

27319000.000 43.94639 260190000.000 62.18

27326000.000 43.94865 260260000.000 62.18

27333000.000 43.95090 260330000.000 62.18

27340000.000 43.95315 260400000.000 62.19

27347000.000 43.95540 260470000.000 62.19

27354000.000 43.95765 260540000.000 62.19

27361000.000 43.95990 260610000.000 62.20

27368000.000 43.96215 260680000.000 62.20

27375000.000 43.96440 260750000.000 62.20

27382000.000 43.96664 260820000.000 62.21

27389000.000 43.96889 260890000.000 62.21

27396000.000 43.97114 260960000.000 62.21

27403000.000 43.97339 261030000.000 62.22

27410000.000 43.97563 261100000.000 62.22

27417000.000 43.97788 261170000.000 62.22

27424000.000 43.98012 261240000.000 62.23

27431000.000 43.98237 261310000.000 62.23

27438000.000 43.98461 261380000.000 62.23

27445000.000 43.98686 261450000.000 62.24

27452000.000 43.98910 261520000.000 62.24

27459000.000 43.99134 261590000.000 62.24

27466000.000 43.99359 261660000.000 62.25

27473000.000 43.99583 261730000.000 62.25

27480000.000 43.99807 261800000.000 62.25

27487000.000 44.00031 261870000.000 62.26

27494000.000 44.00255 261940000.000 62.26

27501000.000 44.00479 262010000.000 62.26

27508000.000 44.00703 262080000.000 62.27

27515000.000 44.00927 262150000.000 62.27

27522000.000 44.01151 262220000.000 62.27

27529000.000 44.01375 262290000.000 62.28

27536000.000 44.01599 262360000.000 62.28

27543000.000 44.01823 262430000.000 62.28

27550000.000 44.02046 262500000.000 62.29

27557000.000 44.02270 262570000.000 62.29

27564000.000 44.02494 262640000.000 62.29

27571000.000 44.02717 262710000.000 62.30

27578000.000 44.02941 262780000.000 62.30

27585000.000 44.03164 262850000.000 62.30

27592000.000 44.03388 262920000.000 62.30

27599000.000 44.03611 262990000.000 62.31

27606000.000 44.03834 263060000.000 62.31

27613000.000 44.04058 263130000.000 62.31

27620000.000 44.04281 263200000.000 62.32

27627000.000 44.04504 263270000.000 62.32

27634000.000 44.04727 263340000.000 62.32

27641000.000 44.04951 263410000.000 62.33

27648000.000 44.05174 263480000.000 62.33

27655000.000 44.05397 263550000.000 62.33

27662000.000 44.05620 263620000.000 62.34

27669000.000 44.05843 263690000.000 62.34

27676000.000 44.06065 263760000.000 62.34

27683000.000 44.06288 263830000.000 62.35

27690000.000 44.06511 263900000.000 62.35

27697000.000 44.06734 263970000.000 62.35

27704000.000 44.06957 264040000.000 62.36

27711000.000 44.07179 264110000.000 62.36

27718000.000 44.07402 264180000.000 62.36

27725000.000 44.07625 264250000.000 62.37

27732000.000 44.07847 264320000.000 62.37

27739000.000 44.08070 264390000.000 62.37

27746000.000 44.08292 264460000.000 62.38

27753000.000 44.08514 264530000.000 62.38

27760000.000 44.08737 264600000.000 62.38

27767000.000 44.08959 264670000.000 62.39

27774000.000 44.09181 264740000.000 62.39

27781000.000 44.09404 264810000.000 62.39

27788000.000 44.09626 264880000.000 62.40

27795000.000 44.09848 264950000.000 62.40

27802000.000 44.10070 265020000.000 62.40

27809000.000 44.10292 265090000.000 62.41

27816000.000 44.10514 265160000.000 62.41

27823000.000 44.10736 265230000.000 62.41

27830000.000 44.10958 265300000.000 62.42

27837000.000 44.11180 265370000.000 62.42

27844000.000 44.11402 265440000.000 62.42

27851000.000 44.11624 265510000.000 62.43

27858000.000 44.11845 265580000.000 62.43

27865000.000 44.12067 265650000.000 62.43

27872000.000 44.12289 265720000.000 62.44

27879000.000 44.12510 265790000.000 62.44

27886000.000 44.12732 265860000.000 62.44

27893000.000 44.12953 265930000.000 62.45

27900000.000 44.13175 266000000.000 62.45

27907000.000 44.13396 266070000.000 62.45

27914000.000 44.13618 266140000.000 62.46

27921000.000 44.13839 266210000.000 62.46

27928000.000 44.14060 266280000.000 62.46

27935000.000 44.14281 266350000.000 62.47

27942000.000 44.14503 266420000.000 62.47

27949000.000 44.14724 266490000.000 62.47

27956000.000 44.14945 266560000.000 62.48

27963000.000 44.15166 266630000.000 62.48

27970000.000 44.15387 266700000.000 62.48

27977000.000 44.15608 266770000.000 62.49

27984000.000 44.15829 266840000.000 62.49

27991000.000 44.16050 266910000.000 62.49

27998000.000 44.16271 266980000.000 62.50

28005000.000 44.16492 267050000.000 62.50

28012000.000 44.16712 267120000.000 62.50

28019000.000 44.16933 267190000.000 62.51

28026000.000 44.17154 267260000.000 62.51

28033000.000 44.17374 267330000.000 62.51

28040000.000 44.17595 267400000.000 62.52

28047000.000 44.17815 267470000.000 62.52

28054000.000 44.18036 267540000.000 62.52

28061000.000 44.18256 267610000.000 62.53

28068000.000 44.18477 267680000.000 62.53

28075000.000 44.18697 267750000.000 62.53

28082000.000 44.18917 267820000.000 62.54

28089000.000 44.19138 267890000.000 62.54

28096000.000 44.19358 267960000.000 62.54

28103000.000 44.19578 268030000.000 62.55

28110000.000 44.19798 268100000.000 62.55

28117000.000 44.20018 268170000.000 62.55

28124000.000 44.20238 268240000.000 62.56

28131000.000 44.20459 268310000.000 62.56

28138000.000 44.20678 268380000.000 62.56

28145000.000 44.20898 268450000.000 62.56

28152000.000 44.21118 268520000.000 62.57

28159000.000 44.21338 268590000.000 62.57

28166000.000 44.21558 268660000.000 62.57

28173000.000 44.21778 268730000.000 62.58

28180000.000 44.21997 268800000.000 62.58

28187000.000 44.22217 268870000.000 62.58

28194000.000 44.22437 268940000.000 62.59

28201000.000 44.22656 269010000.000 62.59

28208000.000 44.22876 269080000.000 62.59

28215000.000 44.23095 269150000.000 62.60

28222000.000 44.23315 269220000.000 62.60

28229000.000 44.23534 269290000.000 62.60

28236000.000 44.23753 269360000.000 62.61

28243000.000 44.23973 269430000.000 62.61

28250000.000 44.24192 269500000.000 62.61

28257000.000 44.24411 269570000.000 62.62

28264000.000 44.24630 269640000.000 62.62

28271000.000 44.24850 269710000.000 62.62

28278000.000 44.25069 269780000.000 62.63

28285000.000 44.25288 269850000.000 62.63

28292000.000 44.25507 269920000.000 62.63

28299000.000 44.25726 269990000.000 62.64

28306000.000 44.25945 270060000.000 62.64

28313000.000 44.26164 270130000.000 62.64

28320000.000 44.26382 270200000.000 62.65

28327000.000 44.26601 270270000.000 62.65

28334000.000 44.26820 270340000.000 62.65

28341000.000 44.27039 270410000.000 62.66

28348000.000 44.27257 270480000.000 62.66

28355000.000 44.27476 270550000.000 62.66

28362000.000 44.27695 270620000.000 62.67

28369000.000 44.27913 270690000.000 62.67

28376000.000 44.28132 270760000.000 62.67

28383000.000 44.28350 270830000.000 62.68

28390000.000 44.28568 270900000.000 62.68

28397000.000 44.28787 270970000.000 62.68

28404000.000 44.29005 271040000.000 62.69

28411000.000 44.29223 271110000.000 62.69

28418000.000 44.29442 271180000.000 62.69

28425000.000 44.29660 271250000.000 62.69

28432000.000 44.29878 271320000.000 62.70

28439000.000 44.30096 271390000.000 62.70

28446000.000 44.30314 271460000.000 62.70

28453000.000 44.30532 271530000.000 62.71

28460000.000 44.30750 271600000.000 62.71

28467000.000 44.30968 271670000.000 62.71

28474000.000 44.31186 271740000.000 62.72

28481000.000 44.31404 271810000.000 62.72

28488000.000 44.31622 271880000.000 62.72

28495000.000 44.31839 271950000.000 62.73

28502000.000 44.32057 272020000.000 62.73

28509000.000 44.32275 272090000.000 62.73

28516000.000 44.32492 272160000.000 62.74

28523000.000 44.32710 272230000.000 62.74

28530000.000 44.32928 272300000.000 62.74

28537000.000 44.33145 272370000.000 62.75

28544000.000 44.33363 272440000.000 62.75

28551000.000 44.33580 272510000.000 62.75

28558000.000 44.33797 272580000.000 62.76

28565000.000 44.34015 272650000.000 62.76

28572000.000 44.34232 272720000.000 62.76

28579000.000 44.34449 272790000.000 62.77

28586000.000 44.34666 272860000.000 62.77

28593000.000 44.34884 272930000.000 62.77

28600000.000 44.35101 273000000.000 62.78

28607000.000 44.35318 273070000.000 62.78

28614000.000 44.35535 273140000.000 62.78

28621000.000 44.35752 273210000.000 62.79

28628000.000 44.35969 273280000.000 62.79

28635000.000 44.36186 273350000.000 62.79

28642000.000 44.36403 273420000.000 62.79

28649000.000 44.36619 273490000.000 62.80

28656000.000 44.36836 273560000.000 62.80

28663000.000 44.37053 273630000.000 62.80

28670000.000 44.37270 273700000.000 62.81

28677000.000 44.37486 273770000.000 62.81

28684000.000 44.37703 273840000.000 62.81

28691000.000 44.37920 273910000.000 62.82

28698000.000 44.38136 273980000.000 62.82

28705000.000 44.38353 274050000.000 62.82

28712000.000 44.38569 274120000.000 62.83

28719000.000 44.38785 274190000.000 62.83

28726000.000 44.39002 274260000.000 62.83

28733000.000 44.39218 274330000.000 62.84

28740000.000 44.39434 274400000.000 62.84

28747000.000 44.39651 274470000.000 62.84

28754000.000 44.39867 274540000.000 62.85

28761000.000 44.40083 274610000.000 62.85

28768000.000 44.40299 274680000.000 62.85

28775000.000 44.40515 274750000.000 62.86

28782000.000 44.40731 274820000.000 62.86

28789000.000 44.40947 274890000.000 62.86

28796000.000 44.41163 274960000.000 62.87

28803000.000 44.41379 275030000.000 62.87

28810000.000 44.41595 275100000.000 62.87

28817000.000 44.41811 275170000.000 62.88

28824000.000 44.42026 275240000.000 62.88

28831000.000 44.42242 275310000.000 62.88

28838000.000 44.42458 275380000.000 62.88

28845000.000 44.42673 275450000.000 62.89

28852000.000 44.42889 275520000.000 62.89

28859000.000 44.43105 275590000.000 62.89

28866000.000 44.43320 275660000.000 62.90

28873000.000 44.43536 275730000.000 62.90

28880000.000 44.43751 275800000.000 62.90

28887000.000 44.43966 275870000.000 62.91

28894000.000 44.44182 275940000.000 62.91

28901000.000 44.44397 276010000.000 62.91

28908000.000 44.44612 276080000.000 62.92

28915000.000 44.44828 276150000.000 62.92

28922000.000 44.45043 276220000.000 62.92

28929000.000 44.45258 276290000.000 62.93

28936000.000 44.45473 276360000.000 62.93

28943000.000 44.45688 276430000.000 62.93

28950000.000 44.45903 276500000.000 62.94

28957000.000 44.46118 276570000.000 62.94

28964000.000 44.46333 276640000.000 62.94

28971000.000 44.46548 276710000.000 62.95

28978000.000 44.46763 276780000.000 62.95

28985000.000 44.46978 276850000.000 62.95

28992000.000 44.47192 276920000.000 62.95

28999000.000 44.47407 276990000.000 62.96

29006000.000 44.47622 277060000.000 62.96

29013000.000 44.47836 277130000.000 62.96

29020000.000 44.48051 277200000.000 62.97

29027000.000 44.48266 277270000.000 62.97

29034000.000 44.48480 277340000.000 62.97

29041000.000 44.48695 277410000.000 62.98

29048000.000 44.48909 277480000.000 62.98

29055000.000 44.49123 277550000.000 62.98

29062000.000 44.49338 277620000.000 62.99

29069000.000 44.49552 277690000.000 62.99

29076000.000 44.49766 277760000.000 62.99

29083000.000 44.49981 277830000.000 63.00

29090000.000 44.50195 277900000.000 63.00

29097000.000 44.50409 277970000.000 63.00

29104000.000 44.50623 278040000.000 63.01

29111000.000 44.50837 278110000.000 63.01

29118000.000 44.51051 278180000.000 63.01

29125000.000 44.51265 278250000.000 63.02

29132000.000 44.51479 278320000.000 63.02

29139000.000 44.51693 278390000.000 63.02

29146000.000 44.51907 278460000.000 63.02

29153000.000 44.52121 278530000.000 63.03

29160000.000 44.52335 278600000.000 63.03

29167000.000 44.52548 278670000.000 63.03

29174000.000 44.52762 278740000.000 63.04

29181000.000 44.52976 278810000.000 63.04

29188000.000 44.53189 278880000.000 63.04

29195000.000 44.53403 278950000.000 63.05

29202000.000 44.53616 279020000.000 63.05

29209000.000 44.53830 279090000.000 63.05

29216000.000 44.54043 279160000.000 63.06

29223000.000 44.54257 279230000.000 63.06

29230000.000 44.54470 279300000.000 63.06

29237000.000 44.54683 279370000.000 63.07

29244000.000 44.54897 279440000.000 63.07

29251000.000 44.55110 279510000.000 63.07

29258000.000 44.55323 279580000.000 63.08

29265000.000 44.55536 279650000.000 63.08

29272000.000 44.55749 279720000.000 63.08

29279000.000 44.55963 279790000.000 63.08

29286000.000 44.56176 279860000.000 63.09

29293000.000 44.56389 279930000.000 63.09

29300000.000 44.56602 280000000.000 63.09

29307000.000 44.56814 280070000.000 63.10

29314000.000 44.57027 280140000.000 63.10

29321000.000 44.57240 280210000.000 63.10

29328000.000 44.57453 280280000.000 63.11

29335000.000 44.57666 280350000.000 63.11

29342000.000 44.57878 280420000.000 63.11

29349000.000 44.58091 280490000.000 63.12

29356000.000 44.58304 280560000.000 63.12

29363000.000 44.58516 280630000.000 63.12

29370000.000 44.58729 280700000.000 63.13

29377000.000 44.58941 280770000.000 63.13

29384000.000 44.59154 280840000.000 63.13

29391000.000 44.59366 280910000.000 63.14

29398000.000 44.59579 280980000.000 63.14

29405000.000 44.59791 281050000.000 63.14

29412000.000 44.60003 281120000.000 63.14

29419000.000 44.60216 281190000.000 63.15

29426000.000 44.60428 281260000.000 63.15

29433000.000 44.60640 281330000.000 63.15

29440000.000 44.60852 281400000.000 63.16

29447000.000 44.61064 281470000.000 63.16

29454000.000 44.61276 281540000.000 63.16

29461000.000 44.61489 281610000.000 63.17

29468000.000 44.61701 281680000.000 63.17

29475000.000 44.61912 281750000.000 63.17

29482000.000 44.62124 281820000.000 63.18

29489000.000 44.62336 281890000.000 63.18

29496000.000 44.62548 281960000.000 63.18

29503000.000 44.62760 282030000.000 63.19

29510000.000 44.62972 282100000.000 63.19

29517000.000 44.63183 282170000.000 63.19

29524000.000 44.63395 282240000.000 63.19

29531000.000 44.63607 282310000.000 63.20

29538000.000 44.63818 282380000.000 63.20

29545000.000 44.64030 282450000.000 63.20

29552000.000 44.64241 282520000.000 63.21

29559000.000 44.64453 282590000.000 63.21

29566000.000 44.64664 282660000.000 63.21

29573000.000 44.64876 282730000.000 63.22

29580000.000 44.65087 282800000.000 63.22

29587000.000 44.65298 282870000.000 63.22

29594000.000 44.65510 282940000.000 63.23

29601000.000 44.65721 283010000.000 63.23

29608000.000 44.65932 283080000.000 63.23

29615000.000 44.66143 283150000.000 63.24

29622000.000 44.66354 283220000.000 63.24

29629000.000 44.66565 283290000.000 63.24

29636000.000 44.66776 283360000.000 63.24

29643000.000 44.66987 283430000.000 63.25

29650000.000 44.67198 283500000.000 63.25

29657000.000 44.67409 283570000.000 63.25

29664000.000 44.67620 283640000.000 63.26

29671000.000 44.67831 283710000.000 63.26

29678000.000 44.68042 283780000.000 63.26

29685000.000 44.68252 283850000.000 63.27

29692000.000 44.68463 283920000.000 63.27

29699000.000 44.68674 283990000.000 63.27

29706000.000 44.68884 284060000.000 63.28

29713000.000 44.69095 284130000.000 63.28

29720000.000 44.69306 284200000.000 63.28

29727000.000 44.69516 284270000.000 63.29

29734000.000 44.69727 284340000.000 63.29

29741000.000 44.69937 284410000.000 63.29

29748000.000 44.70147 284480000.000 63.29

29755000.000 44.70358 284550000.000 63.30

29762000.000 44.70568 284620000.000 63.30

29769000.000 44.70778 284690000.000 63.30

29776000.000 44.70989 284760000.000 63.31

29783000.000 44.71199 284830000.000 63.31

29790000.000 44.71409 284900000.000 63.31

29797000.000 44.71619 284970000.000 63.32

29804000.000 44.71829 285040000.000 63.32

29811000.000 44.72039 285110000.000 63.32

29818000.000 44.72249 285180000.000 63.33

29825000.000 44.72459 285250000.000 63.33

29832000.000 44.72669 285320000.000 63.33

29839000.000 44.72879 285390000.000 63.34

29846000.000 44.73089 285460000.000 63.34

29853000.000 44.73299 285530000.000 63.34

29860000.000 44.73508 285600000.000 63.34

29867000.000 44.73718 285670000.000 63.35

29874000.000 44.73928 285740000.000 63.35

29881000.000 44.74137 285810000.000 63.35

29888000.000 44.74347 285880000.000 63.36

29895000.000 44.74556 285950000.000 63.36

29902000.000 44.74766 286020000.000 63.36

29909000.000 44.74976 286090000.000 63.37

29916000.000 44.75185 286160000.000 63.37

29923000.000 44.75394 286230000.000 63.37

29930000.000 44.75604 286300000.000 63.38

29937000.000 44.75813 286370000.000 63.38

29944000.000 44.76022 286440000.000 63.38

29951000.000 44.76232 286510000.000 63.38

29958000.000 44.76441 286580000.000 63.39

29965000.000 44.76650 286650000.000 63.39

29972000.000 44.76859 286720000.000 63.39

29979000.000 44.77068 286790000.000 63.40

29986000.000 44.77277 286860000.000 63.40

29993000.000 44.77486 286930000.000 63.40

30000000.000 44.77695 287000000.000 63.41

30007000.000 44.77904 287070000.000 63.41

30014000.000 44.78113 287140000.000 63.41

30021000.000 44.78322 287210000.000 63.42

30028000.000 44.78531 287280000.000 63.42

30035000.000 44.78740 287350000.000 63.42

30042000.000 44.78948 287420000.000 63.43

30049000.000 44.79157 287490000.000 63.43

30056000.000 44.79366 287560000.000 63.43

30063000.000 44.79574 287630000.000 63.43

30070000.000 44.79783 287700000.000 63.44

30077000.000 44.79991 287770000.000 63.44

30084000.000 44.80200 287840000.000 63.44

30091000.000 44.80408 287910000.000 63.45

30098000.000 44.80617 287980000.000 63.45

30105000.000 44.80825 288050000.000 63.45

30112000.000 44.81034 288120000.000 63.46

30119000.000 44.81242 288190000.000 63.46

30126000.000 44.81450 288260000.000 63.46

30133000.000 44.81658 288330000.000 63.47

30140000.000 44.81867 288400000.000 63.47

30147000.000 44.82075 288470000.000 63.47

30154000.000 44.82283 288540000.000 63.47

30161000.000 44.82491 288610000.000 63.48

30168000.000 44.82699 288680000.000 63.48

30175000.000 44.82907 288750000.000 63.48

30182000.000 44.83115 288820000.000 63.49

30189000.000 44.83323 288890000.000 63.49

30196000.000 44.83531 288960000.000 63.49

30203000.000 44.83739 289030000.000 63.50

30210000.000 44.83947 289100000.000 63.50

30217000.000 44.84154 289170000.000 63.50

30224000.000 44.84362 289240000.000 63.51

30231000.000 44.84570 289310000.000 63.51

30238000.000 44.84777 289380000.000 63.51

30245000.000 44.84985 289450000.000 63.51

30252000.000 44.85193 289520000.000 63.52

30259000.000 44.85400 289590000.000 63.52

30266000.000 44.85608 289660000.000 63.52

30273000.000 44.85815 289730000.000 63.53

30280000.000 44.86023 289800000.000 63.53

30287000.000 44.86230 289870000.000 63.53

30294000.000 44.86437 289940000.000 63.54

30301000.000 44.86645 290010000.000 63.54

30308000.000 44.86852 290080000.000 63.54

30315000.000 44.87059 290150000.000 63.55

30322000.000 44.87266 290220000.000 63.55

30329000.000 44.87474 290290000.000 63.55

30336000.000 44.87681 290360000.000 63.55

30343000.000 44.87888 290430000.000 63.56

30350000.000 44.88095 290500000.000 63.56

30357000.000 44.88302 290570000.000 63.56

30364000.000 44.88509 290640000.000 63.57

30371000.000 44.88716 290710000.000 63.57

30378000.000 44.88923 290780000.000 63.57

30385000.000 44.89129 290850000.000 63.58

30392000.000 44.89336 290920000.000 63.58

30399000.000 44.89543 290990000.000 63.58

30406000.000 44.89750 291060000.000 63.58

30413000.000 44.89957 291130000.000 63.59

30420000.000 44.90163 291200000.000 63.59

30427000.000 44.90370 291270000.000 63.59

30434000.000 44.90576 291340000.000 63.60

30441000.000 44.90783 291410000.000 63.60

30448000.000 44.90989 291480000.000 63.60

30455000.000 44.91196 291550000.000 63.61

30462000.000 44.91402 291620000.000 63.61

30469000.000 44.91609 291690000.000 63.61

30476000.000 44.91815 291760000.000 63.62

30483000.000 44.92021 291830000.000 63.62

30490000.000 44.92228 291900000.000 63.62

30497000.000 44.92434 291970000.000 63.62

30504000.000 44.92640 292040000.000 63.63

30511000.000 44.92846 292110000.000 63.63

30518000.000 44.93053 292180000.000 63.63

30525000.000 44.93259 292250000.000 63.64

30532000.000 44.93465 292320000.000 63.64

30539000.000 44.93671 292390000.000 63.64

30546000.000 44.93877 292460000.000 63.65

30553000.000 44.94083 292530000.000 63.65

30560000.000 44.94289 292600000.000 63.65

30567000.000 44.94494 292670000.000 63.66

30574000.000 44.94700 292740000.000 63.66

30581000.000 44.94906 292810000.000 63.66

30588000.000 44.95112 292880000.000 63.66

30595000.000 44.95318 292950000.000 63.67

30602000.000 44.95523 293020000.000 63.67

30609000.000 44.95729 293090000.000 63.67

30616000.000 44.95934 293160000.000 63.68

30623000.000 44.96140 293230000.000 63.68

30630000.000 44.96346 293300000.000 63.68

30637000.000 44.96551 293370000.000 63.69

30644000.000 44.96757 293440000.000 63.69

30651000.000 44.96962 293510000.000 63.69

30658000.000 44.97167 293580000.000 63.69

30665000.000 44.97373 293650000.000 63.70

30672000.000 44.97578 293720000.000 63.70

30679000.000 44.97783 293790000.000 63.70

30686000.000 44.97988 293860000.000 63.71

30693000.000 44.98194 293930000.000 63.71

30700000.000 44.98399 294000000.000 63.71

30707000.000 44.98604 294070000.000 63.72

30714000.000 44.98809 294140000.000 63.72

30721000.000 44.99014 294210000.000 63.72

30728000.000 44.99219 294280000.000 63.73

30735000.000 44.99424 294350000.000 63.73

30742000.000 44.99629 294420000.000 63.73

30749000.000 44.99834 294490000.000 63.73

30756000.000 45.00039 294560000.000 63.74

30763000.000 45.00244 294630000.000 63.74

30770000.000 45.00448 294700000.000 63.74

30777000.000 45.00653 294770000.000 63.75

30784000.000 45.00858 294840000.000 63.75

30791000.000 45.01062 294910000.000 63.75

30798000.000 45.01267 294980000.000 63.76

30805000.000 45.01472 295050000.000 63.76

30812000.000 45.01676 295120000.000 63.76

30819000.000 45.01881 295190000.000 63.76

30826000.000 45.02085 295260000.000 63.77

30833000.000 45.02290 295330000.000 63.77

30840000.000 45.02494 295400000.000 63.77

30847000.000 45.02699 295470000.000 63.78

30854000.000 45.02903 295540000.000 63.78

30861000.000 45.03107 295610000.000 63.78

30868000.000 45.03311 295680000.000 63.79

30875000.000 45.03516 295750000.000 63.79

30882000.000 45.03720 295820000.000 63.79

30889000.000 45.03924 295890000.000 63.79

30896000.000 45.04128 295960000.000 63.80

30903000.000 45.04332 296030000.000 63.80

30910000.000 45.04536 296100000.000 63.80

30917000.000 45.04740 296170000.000 63.81

30924000.000 45.04944 296240000.000 63.81

30931000.000 45.05148 296310000.000 63.81

30938000.000 45.05352 296380000.000 63.82

30945000.000 45.05556 296450000.000 63.82

30952000.000 45.05760 296520000.000 63.82

30959000.000 45.05963 296590000.000 63.82

30966000.000 45.06167 296660000.000 63.83

30973000.000 45.06371 296730000.000 63.83

30980000.000 45.06575 296800000.000 63.83

30987000.000 45.06778 296870000.000 63.84

30994000.000 45.06982 296940000.000 63.84

31001000.000 45.07185 297010000.000 63.84

31008000.000 45.07389 297080000.000 63.85

31015000.000 45.07592 297150000.000 63.85

31022000.000 45.07796 297220000.000 63.85

31029000.000 45.07999 297290000.000 63.85

31036000.000 45.08203 297360000.000 63.86

31043000.000 45.08406 297430000.000 63.86

31050000.000 45.08609 297500000.000 63.86

31057000.000 45.08813 297570000.000 63.87

31064000.000 45.09016 297640000.000 63.87

31071000.000 45.09219 297710000.000 63.87

31078000.000 45.09422 297780000.000 63.88

31085000.000 45.09625 297850000.000 63.88

31092000.000 45.09828 297920000.000 63.88

31099000.000 45.10031 297990000.000 63.88

31106000.000 45.10234 298060000.000 63.89

31113000.000 45.10437 298130000.000 63.89

31120000.000 45.10640 298200000.000 63.89

31127000.000 45.10843 298270000.000 63.90

31134000.000 45.11046 298340000.000 63.90

31141000.000 45.11249 298410000.000 63.90

31148000.000 45.11452 298480000.000 63.91

31155000.000 45.11655 298550000.000 63.91

31162000.000 45.11857 298620000.000 63.91

31169000.000 45.12060 298690000.000 63.91

31176000.000 45.12263 298760000.000 63.92

31183000.000 45.12465 298830000.000 63.92

31190000.000 45.12668 298900000.000 63.92

31197000.000 45.12870 298970000.000 63.93

31204000.000 45.13073 299040000.000 63.93

31211000.000 45.13275 299110000.000 63.93

31218000.000 45.13478 299180000.000 63.94

31225000.000 45.13680 299250000.000 63.94

31232000.000 45.13882 299320000.000 63.94

31239000.000 45.14085 299390000.000 63.94

31246000.000 45.14287 299460000.000 63.95

31253000.000 45.14489 299530000.000 63.95

31260000.000 45.14691 299600000.000 63.95

31267000.000 45.14894 299670000.000 63.96

31274000.000 45.15096 299740000.000 63.96

31281000.000 45.15298 299810000.000 63.96

31288000.000 45.15500 299880000.000 63.97

31295000.000 45.15702 299950000.000 63.97

31302000.000 45.15904 300020000.000 63.97

31309000.000 45.16106 300090000.000 63.97

31316000.000 45.16308 300160000.000 63.98

31323000.000 45.16510 300230000.000 63.98

31330000.000 45.16712 300300000.000 63.98

31337000.000 45.16913 300370000.000 63.99

31344000.000 45.17115 300440000.000 63.99

31351000.000 45.17317 300510000.000 63.99

31358000.000 45.17519 300580000.000 64.00

31365000.000 45.17720 300650000.000 64.00

31372000.000 45.17922 300720000.000 64.00

31379000.000 45.18124 300790000.000 64.00

31386000.000 45.18325 300860000.000 64.01

31393000.000 45.18527 300930000.000 64.01

31400000.000 45.18728 301000000.000 64.01

31407000.000 45.18930 301070000.000 64.02

31414000.000 45.19131 301140000.000 64.02

31421000.000 45.19332 301210000.000 64.02

31428000.000 45.19534 301280000.000 64.03

31435000.000 45.19735 301350000.000 64.03

31442000.000 45.19936 301420000.000 64.03

31449000.000 45.20138 301490000.000 64.03

31456000.000 45.20339 301560000.000 64.04

31463000.000 45.20540 301630000.000 64.04

31470000.000 45.20741 301700000.000 64.04

31477000.000 45.20942 301770000.000 64.05

31484000.000 45.21143 301840000.000 64.05

31491000.000 45.21344 301910000.000 64.05

31498000.000 45.21545 301980000.000 64.06

31505000.000 45.21746 302050000.000 64.06

31512000.000 45.21947 302120000.000 64.06

31519000.000 45.22148 302190000.000 64.06

31526000.000 45.22349 302260000.000 64.07

31533000.000 45.22550 302330000.000 64.07

31540000.000 45.22750 302400000.000 64.07

31547000.000 45.22951 302470000.000 64.08

31554000.000 45.23152 302540000.000 64.08

31561000.000 45.23353 302610000.000 64.08

31568000.000 45.23553 302680000.000 64.08

31575000.000 45.23754 302750000.000 64.09

31582000.000 45.23954 302820000.000 64.09

31589000.000 45.24155 302890000.000 64.09

31596000.000 45.24355 302960000.000 64.10

31603000.000 45.24556 303030000.000 64.10

31610000.000 45.24756 303100000.000 64.10

31617000.000 45.24957 303170000.000 64.11

31624000.000 45.25157 303240000.000 64.11

31631000.000 45.25357 303310000.000 64.11

31638000.000 45.25558 303380000.000 64.11

31645000.000 45.25758 303450000.000 64.12

31652000.000 45.25958 303520000.000 64.12

31659000.000 45.26158 303590000.000 64.12

31666000.000 45.26358 303660000.000 64.13

31673000.000 45.26558 303730000.000 64.13

31680000.000 45.26758 303800000.000 64.13

31687000.000 45.26958 303870000.000 64.14

31694000.000 45.27158 303940000.000 64.14

31701000.000 45.27358 304010000.000 64.14

31708000.000 45.27558 304080000.000 64.14

31715000.000 45.27758 304150000.000 64.15

31722000.000 45.27958 304220000.000 64.15

31729000.000 45.28158 304290000.000 64.15

31736000.000 45.28358 304360000.000 64.16

31743000.000 45.28557 304430000.000 64.16

31750000.000 45.28757 304500000.000 64.16

31757000.000 45.28957 304570000.000 64.16

31764000.000 45.29156 304640000.000 64.17

31771000.000 45.29356 304710000.000 64.17

31778000.000 45.29556 304780000.000 64.17

31785000.000 45.29755 304850000.000 64.18

31792000.000 45.29955 304920000.000 64.18

31799000.000 45.30154 304990000.000 64.18

31806000.000 45.30354 305060000.000 64.19

31813000.000 45.30553 305130000.000 64.19

31820000.000 45.30752 305200000.000 64.19

31827000.000 45.30952 305270000.000 64.19

31834000.000 45.31151 305340000.000 64.20

31841000.000 45.31350 305410000.000 64.20

31848000.000 45.31549 305480000.000 64.20

31855000.000 45.31749 305550000.000 64.21

31862000.000 45.31948 305620000.000 64.21

31869000.000 45.32147 305690000.000 64.21

31876000.000 45.32346 305760000.000 64.21

31883000.000 45.32545 305830000.000 64.22

31890000.000 45.32744 305900000.000 64.22

31897000.000 45.32943 305970000.000 64.22

31904000.000 45.33142 306040000.000 64.23

31911000.000 45.33341 306110000.000 64.23

31918000.000 45.33540 306180000.000 64.23

31925000.000 45.33738 306250000.000 64.24

31932000.000 45.33937 306320000.000 64.24

31939000.000 45.34136 306390000.000 64.24

31946000.000 45.34335 306460000.000 64.24

31953000.000 45.34533 306530000.000 64.25

31960000.000 45.34732 306600000.000 64.25

31967000.000 45.34931 306670000.000 64.25

31974000.000 45.35129 306740000.000 64.26

31981000.000 45.35328 306810000.000 64.26

31988000.000 45.35526 306880000.000 64.26

31995000.000 45.35725 306950000.000 64.26

32002000.000 45.35923 307020000.000 64.27

32009000.000 45.36122 307090000.000 64.27

32016000.000 45.36320 307160000.000 64.27

32023000.000 45.36519 307230000.000 64.28

32030000.000 45.36717 307300000.000 64.28

32037000.000 45.36915 307370000.000 64.28

32044000.000 45.37113 307440000.000 64.29

32051000.000 45.37312 307510000.000 64.29

32058000.000 45.37510 307580000.000 64.29

32065000.000 45.37708 307650000.000 64.29

32072000.000 45.37906 307720000.000 64.30

32079000.000 45.38104 307790000.000 64.30

32086000.000 45.38302 307860000.000 64.30

32093000.000 45.38500 307930000.000 64.31

32100000.000 45.38698 308000000.000 64.31

32107000.000 45.38896 308070000.000 64.31

32114000.000 45.39094 308140000.000 64.31

32121000.000 45.39292 308210000.000 64.32

32128000.000 45.39490 308280000.000 64.32

32135000.000 45.39687 308350000.000 64.32

32142000.000 45.39885 308420000.000 64.33

32149000.000 45.40083 308490000.000 64.33

32156000.000 45.40281 308560000.000 64.33

32163000.000 45.40478 308630000.000 64.33

32170000.000 45.40676 308700000.000 64.34

32177000.000 45.40873 308770000.000 64.34

32184000.000 45.41071 308840000.000 64.34

32191000.000 45.41268 308910000.000 64.35

32198000.000 45.41466 308980000.000 64.35

32205000.000 45.41663 309050000.000 64.35

32212000.000 45.41861 309120000.000 64.36

32219000.000 45.42058 309190000.000 64.36

32226000.000 45.42256 309260000.000 64.36

32233000.000 45.42453 309330000.000 64.36

32240000.000 45.42650 309400000.000 64.37

32247000.000 45.42847 309470000.000 64.37

32254000.000 45.43045 309540000.000 64.37

32261000.000 45.43242 309610000.000 64.38

32268000.000 45.43439 309680000.000 64.38

32275000.000 45.43636 309750000.000 64.38

32282000.000 45.43833 309820000.000 64.38

32289000.000 45.44030 309890000.000 64.39

32296000.000 45.44227 309960000.000 64.39

32303000.000 45.44424 310030000.000 64.39

32310000.000 45.44621 310100000.000 64.40

32317000.000 45.44818 310170000.000 64.40

32324000.000 45.45015 310240000.000 64.40

32331000.000 45.45212 310310000.000 64.40

32338000.000 45.45408 310380000.000 64.41

32345000.000 45.45605 310450000.000 64.41

32352000.000 45.45802 310520000.000 64.41

32359000.000 45.45999 310590000.000 64.42

32366000.000 45.46195 310660000.000 64.42

32373000.000 45.46392 310730000.000 64.42

32380000.000 45.46589 310800000.000 64.42

32387000.000 45.46785 310870000.000 64.43

32394000.000 45.46982 310940000.000 64.43

32401000.000 45.47178 311010000.000 64.43

32408000.000 45.47375 311080000.000 64.44

32415000.000 45.47571 311150000.000 64.44

32422000.000 45.47767 311220000.000 64.44

32429000.000 45.47964 311290000.000 64.45

32436000.000 45.48160 311360000.000 64.45

32443000.000 45.48356 311430000.000 64.45

32450000.000 45.48553 311500000.000 64.45

32457000.000 45.48749 311570000.000 64.46

32464000.000 45.48945 311640000.000 64.46

32471000.000 45.49141 311710000.000 64.46

32478000.000 45.49337 311780000.000 64.47

32485000.000 45.49533 311850000.000 64.47

32492000.000 45.49729 311920000.000 64.47

32499000.000 45.49925 311990000.000 64.47

32506000.000 45.50121 312060000.000 64.48

32513000.000 45.50317 312130000.000 64.48

32520000.000 45.50513 312200000.000 64.48

32527000.000 45.50709 312270000.000 64.49

32534000.000 45.50905 312340000.000 64.49

32541000.000 45.51101 312410000.000 64.49

32548000.000 45.51297 312480000.000 64.49

32555000.000 45.51492 312550000.000 64.50

32562000.000 45.51688 312620000.000 64.50

32569000.000 45.51884 312690000.000 64.50

32576000.000 45.52079 312760000.000 64.51

32583000.000 45.52275 312830000.000 64.51

32590000.000 45.52471 312900000.000 64.51

32597000.000 45.52666 312970000.000 64.51

32604000.000 45.52862 313040000.000 64.52

32611000.000 45.53057 313110000.000 64.52

32618000.000 45.53253 313180000.000 64.52

32625000.000 45.53448 313250000.000 64.53

32632000.000 45.53643 313320000.000 64.53

32639000.000 45.53839 313390000.000 64.53

32646000.000 45.54034 313460000.000 64.53

32653000.000 45.54229 313530000.000 64.54

32660000.000 45.54425 313600000.000 64.54

32667000.000 45.54620 313670000.000 64.54

32674000.000 45.54815 313740000.000 64.55

32681000.000 45.55010 313810000.000 64.55

32688000.000 45.55205 313880000.000 64.55

32695000.000 45.55400 313950000.000 64.56

32702000.000 45.55595 314020000.000 64.56

32709000.000 45.55790 314090000.000 64.56

32716000.000 45.55985 314160000.000 64.56

32723000.000 45.56180 314230000.000 64.57

32730000.000 45.56375 314300000.000 64.57

32737000.000 45.56570 314370000.000 64.57

32744000.000 45.56765 314440000.000 64.58

32751000.000 45.56960 314510000.000 64.58

32758000.000 45.57155 314580000.000 64.58

32765000.000 45.57349 314650000.000 64.58

32772000.000 45.57544 314720000.000 64.59

32779000.000 45.57739 314790000.000 64.59

32786000.000 45.57933 314860000.000 64.59

32793000.000 45.58128 314930000.000 64.60

32800000.000 45.58323 315000000.000 64.60

32807000.000 45.58517 315070000.000 64.60

32814000.000 45.58712 315140000.000 64.60

32821000.000 45.58906 315210000.000 64.61

32828000.000 45.59101 315280000.000 64.61

32835000.000 45.59295 315350000.000 64.61

32842000.000 45.59489 315420000.000 64.62

32849000.000 45.59684 315490000.000 64.62

32856000.000 45.59878 315560000.000 64.62

32863000.000 45.60072 315630000.000 64.62

32870000.000 45.60267 315700000.000 64.63

32877000.000 45.60461 315770000.000 64.63

32884000.000 45.60655 315840000.000 64.63

32891000.000 45.60849 315910000.000 64.64

32898000.000 45.61043 315980000.000 64.64

32905000.000 45.61237 316050000.000 64.64

32912000.000 45.61431 316120000.000 64.64

32919000.000 45.61625 316190000.000 64.65

32926000.000 45.61819 316260000.000 64.65

32933000.000 45.62013 316330000.000 64.65

32940000.000 45.62207 316400000.000 64.66

32947000.000 45.62401 316470000.000 64.66

32954000.000 45.62595 316540000.000 64.66

32961000.000 45.62789 316610000.000 64.66

32968000.000 45.62983 316680000.000 64.67

32975000.000 45.63176 316750000.000 64.67

32982000.000 45.63370 316820000.000 64.67

32989000.000 45.63564 316890000.000 64.68

32996000.000 45.63757 316960000.000 64.68

33003000.000 45.63951 317030000.000 64.68

33010000.000 45.64145 317100000.000 64.68

33017000.000 45.64338 317170000.000 64.69

33024000.000 45.64532 317240000.000 64.69

33031000.000 45.64725 317310000.000 64.69

33038000.000 45.64919 317380000.000 64.70

33045000.000 45.65112 317450000.000 64.70

33052000.000 45.65305 317520000.000 64.70

33059000.000 45.65499 317590000.000 64.70

33066000.000 45.65692 317660000.000 64.71

33073000.000 45.65885 317730000.000 64.71

33080000.000 45.66079 317800000.000 64.71

33087000.000 45.66272 317870000.000 64.72

33094000.000 45.66465 317940000.000 64.72

33101000.000 45.66658 318010000.000 64.72

33108000.000 45.66851 318080000.000 64.72

33115000.000 45.67044 318150000.000 64.73

33122000.000 45.67237 318220000.000 64.73

33129000.000 45.67431 318290000.000 64.73

33136000.000 45.67624 318360000.000 64.74

33143000.000 45.67816 318430000.000 64.74

33150000.000 45.68009 318500000.000 64.74

33157000.000 45.68202 318570000.000 64.74

33164000.000 45.68395 318640000.000 64.75

33171000.000 45.68588 318710000.000 64.75

33178000.000 45.68781 318780000.000 64.75

33185000.000 45.68974 318850000.000 64.76

33192000.000 45.69166 318920000.000 64.76

33199000.000 45.69359 318990000.000 64.76

33206000.000 45.69552 319060000.000 64.76

33213000.000 45.69744 319130000.000 64.77

33220000.000 45.69937 319200000.000 64.77

33227000.000 45.70130 319270000.000 64.77

33234000.000 45.70322 319340000.000 64.78

33241000.000 45.70515 319410000.000 64.78

33248000.000 45.70707 319480000.000 64.78

33255000.000 45.70900 319550000.000 64.78

33262000.000 45.71092 319620000.000 64.79

33269000.000 45.71284 319690000.000 64.79

33276000.000 45.71477 319760000.000 64.79

33283000.000 45.71669 319830000.000 64.80

33290000.000 45.71861 319900000.000 64.80

33297000.000 45.72054 319970000.000 64.80

33304000.000 45.72246 320040000.000 64.80

33311000.000 45.72438 320110000.000 64.81

33318000.000 45.72630 320180000.000 64.81

33325000.000 45.72822 320250000.000 64.81

33332000.000 45.73014 320320000.000 64.81

33339000.000 45.73206 320390000.000 64.82

33346000.000 45.73398 320460000.000 64.82

33353000.000 45.73590 320530000.000 64.82

33360000.000 45.73782 320600000.000 64.83

33367000.000 45.73974 320670000.000 64.83

33374000.000 45.74166 320740000.000 64.83

33381000.000 45.74358 320810000.000 64.83

33388000.000 45.74550 320880000.000 64.84

33395000.000 45.74742 320950000.000 64.84

33402000.000 45.74933 321020000.000 64.84

33409000.000 45.75125 321090000.000 64.85

33416000.000 45.75317 321160000.000 64.85

33423000.000 45.75509 321230000.000 64.85

33430000.000 45.75700 321300000.000 64.85

33437000.000 45.75892 321370000.000 64.86

33444000.000 45.76083 321440000.000 64.86

33451000.000 45.76275 321510000.000 64.86

33458000.000 45.76466 321580000.000 64.87

33465000.000 45.76658 321650000.000 64.87

33472000.000 45.76849 321720000.000 64.87

33479000.000 45.77041 321790000.000 64.87

33486000.000 45.77232 321860000.000 64.88

33493000.000 45.77423 321930000.000 64.88

33500000.000 45.77615 322000000.000 64.88

33507000.000 45.77806 322070000.000 64.89

33514000.000 45.77997 322140000.000 64.89

33521000.000 45.78189 322210000.000 64.89

33528000.000 45.78380 322280000.000 64.89

33535000.000 45.78571 322350000.000 64.90

33542000.000 45.78762 322420000.000 64.90

33549000.000 45.78953 322490000.000 64.90

33556000.000 45.79144 322560000.000 64.91

33563000.000 45.79335 322630000.000 64.91

33570000.000 45.79526 322700000.000 64.91

33577000.000 45.79717 322770000.000 64.91

33584000.000 45.79908 322840000.000 64.92

33591000.000 45.80099 322910000.000 64.92

33598000.000 45.80290 322980000.000 64.92

33605000.000 45.80481 323050000.000 64.93

33612000.000 45.80672 323120000.000 64.93

33619000.000 45.80862 323190000.000 64.93

33626000.000 45.81053 323260000.000 64.93

33633000.000 45.81244 323330000.000 64.94

33640000.000 45.81434 323400000.000 64.94

33647000.000 45.81625 323470000.000 64.94

33654000.000 45.81816 323540000.000 64.94

33661000.000 45.82006 323610000.000 64.95

33668000.000 45.82197 323680000.000 64.95

33675000.000 45.82387 323750000.000 64.95

33682000.000 45.82578 323820000.000 64.96

33689000.000 45.82768 323890000.000 64.96

33696000.000 45.82959 323960000.000 64.96

33703000.000 45.83149 324030000.000 64.96

33710000.000 45.83340 324100000.000 64.97

33717000.000 45.83530 324170000.000 64.97

33724000.000 45.83720 324240000.000 64.97

33731000.000 45.83910 324310000.000 64.98

33738000.000 45.84101 324380000.000 64.98

33745000.000 45.84291 324450000.000 64.98

33752000.000 45.84481 324520000.000 64.98

33759000.000 45.84671 324590000.000 64.99

33766000.000 45.84861 324660000.000 64.99

33773000.000 45.85051 324730000.000 64.99

33780000.000 45.85241 324800000.000 65.00

33787000.000 45.85432 324870000.000 65.00

33794000.000 45.85621 324940000.000 65.00

33801000.000 45.85811 325010000.000 65.00

33808000.000 45.86001 325080000.000 65.01

33815000.000 45.86191 325150000.000 65.01

33822000.000 45.86381 325220000.000 65.01

33829000.000 45.86571 325290000.000 65.01

33836000.000 45.86761 325360000.000 65.02

33843000.000 45.86951 325430000.000 65.02

33850000.000 45.87140 325500000.000 65.02

33857000.000 45.87330 325570000.000 65.03

33864000.000 45.87520 325640000.000 65.03

33871000.000 45.87709 325710000.000 65.03

33878000.000 45.87899 325780000.000 65.03

33885000.000 45.88088 325850000.000 65.04

33892000.000 45.88278 325920000.000 65.04

33899000.000 45.88468 325990000.000 65.04

33906000.000 45.88657 326060000.000 65.05

33913000.000 45.88846 326130000.000 65.05

33920000.000 45.89036 326200000.000 65.05

33927000.000 45.89225 326270000.000 65.05

33934000.000 45.89415 326340000.000 65.06

33941000.000 45.89604 326410000.000 65.06

33948000.000 45.89793 326480000.000 65.06

33955000.000 45.89983 326550000.000 65.07

33962000.000 45.90172 326620000.000 65.07

33969000.000 45.90361 326690000.000 65.07

33976000.000 45.90550 326760000.000 65.07

33983000.000 45.90739 326830000.000 65.08

33990000.000 45.90928 326900000.000 65.08

33997000.000 45.91117 326970000.000 65.08

34004000.000 45.91306 327040000.000 65.08

34011000.000 45.91496 327110000.000 65.09

34018000.000 45.91684 327180000.000 65.09

34025000.000 45.91873 327250000.000 65.09

34032000.000 45.92062 327320000.000 65.10

34039000.000 45.92251 327390000.000 65.10

34046000.000 45.92440 327460000.000 65.10

34053000.000 45.92629 327530000.000 65.10

34060000.000 45.92818 327600000.000 65.11

34067000.000 45.93007 327670000.000 65.11

34074000.000 45.93195 327740000.000 65.11

34081000.000 45.93384 327810000.000 65.12

34088000.000 45.93573 327880000.000 65.12

34095000.000 45.93761 327950000.000 65.12

34102000.000 45.93950 328020000.000 65.12

34109000.000 45.94138 328090000.000 65.13

34116000.000 45.94327 328160000.000 65.13

34123000.000 45.94516 328230000.000 65.13

34130000.000 45.94704 328300000.000 65.13

34137000.000 45.94892 328370000.000 65.14

34144000.000 45.95081 328440000.000 65.14

34151000.000 45.95269 328510000.000 65.14

34158000.000 45.95458 328580000.000 65.15

34165000.000 45.95646 328650000.000 65.15

34172000.000 45.95834 328720000.000 65.15

34179000.000 45.96023 328790000.000 65.15

34186000.000 45.96211 328860000.000 65.16

34193000.000 45.96399 328930000.000 65.16

34200000.000 45.96587 329000000.000 65.16

34207000.000 45.96775 329070000.000 65.17

34214000.000 45.96963 329140000.000 65.17

34221000.000 45.97152 329210000.000 65.17

34228000.000 45.97340 329280000.000 65.17

34235000.000 45.97528 329350000.000 65.18

34242000.000 45.97716 329420000.000 65.18

34249000.000 45.97904 329490000.000 65.18

34256000.000 45.98092 329560000.000 65.18

34263000.000 45.98279 329630000.000 65.19

34270000.000 45.98467 329700000.000 65.19

34277000.000 45.98655 329770000.000 65.19

34284000.000 45.98843 329840000.000 65.20

34291000.000 45.99031 329910000.000 65.20

34298000.000 45.99219 329980000.000 65.20

34305000.000 45.99406 330050000.000 65.20

34312000.000 45.99594 330120000.000 65.21

34319000.000 45.99782 330190000.000 65.21

34326000.000 45.99969 330260000.000 65.21

34333000.000 46.00157 330330000.000 65.22

34340000.000 46.00344 330400000.000 65.22

34347000.000 46.00532 330470000.000 65.22

34354000.000 46.00719 330540000.000 65.22

34361000.000 46.00907 330610000.000 65.23

34368000.000 46.01094 330680000.000 65.23

34375000.000 46.01282 330750000.000 65.23

34382000.000 46.01469 330820000.000 65.23

34389000.000 46.01656 330890000.000 65.24

34396000.000 46.01844 330960000.000 65.24

34403000.000 46.02031 331030000.000 65.24

34410000.000 46.02218 331100000.000 65.25

34417000.000 46.02406 331170000.000 65.25

34424000.000 46.02593 331240000.000 65.25

34431000.000 46.02780 331310000.000 65.25

34438000.000 46.02967 331380000.000 65.26

34445000.000 46.03154 331450000.000 65.26

34452000.000 46.03341 331520000.000 65.26

34459000.000 46.03528 331590000.000 65.26

34466000.000 46.03715 331660000.000 65.27

34473000.000 46.03902 331730000.000 65.27

34480000.000 46.04089 331800000.000 65.27

34487000.000 46.04276 331870000.000 65.28

34494000.000 46.04463 331940000.000 65.28

34501000.000 46.04650 332010000.000 65.28

34508000.000 46.04837 332080000.000 65.28

34515000.000 46.05024 332150000.000 65.29

34522000.000 46.05210 332220000.000 65.29

34529000.000 46.05397 332290000.000 65.29

34536000.000 46.05584 332360000.000 65.29

34543000.000 46.05771 332430000.000 65.30

34550000.000 46.05957 332500000.000 65.30

34557000.000 46.06144 332570000.000 65.30

34564000.000 46.06330 332640000.000 65.31

34571000.000 46.06517 332710000.000 65.31

34578000.000 46.06703 332780000.000 65.31

34585000.000 46.06890 332850000.000 65.31

34592000.000 46.07076 332920000.000 65.32

34599000.000 46.07263 332990000.000 65.32

34606000.000 46.07449 333060000.000 65.32

34613000.000 46.07636 333130000.000 65.33

34620000.000 46.07822 333200000.000 65.33

34627000.000 46.08008 333270000.000 65.33

34634000.000 46.08195 333340000.000 65.33

34641000.000 46.08381 333410000.000 65.34

34648000.000 46.08567 333480000.000 65.34

34655000.000 46.08753 333550000.000 65.34

34662000.000 46.08940 333620000.000 65.34

34669000.000 46.09126 333690000.000 65.35

34676000.000 46.09312 333760000.000 65.35

34683000.000 46.09498 333830000.000 65.35

34690000.000 46.09684 333900000.000 65.36

34697000.000 46.09870 333970000.000 65.36

34704000.000 46.10056 334040000.000 65.36

34711000.000 46.10242 334110000.000 65.36

34718000.000 46.10428 334180000.000 65.37

34725000.000 46.10614 334250000.000 65.37

34732000.000 46.10800 334320000.000 65.37

34739000.000 46.10985 334390000.000 65.37

34746000.000 46.11171 334460000.000 65.38

34753000.000 46.11357 334530000.000 65.38

34760000.000 46.11543 334600000.000 65.38

34767000.000 46.11728 334670000.000 65.39

34774000.000 46.11914 334740000.000 65.39

34781000.000 46.12100 334810000.000 65.39

34788000.000 46.12285 334880000.000 65.39

34795000.000 46.12471 334950000.000 65.40

34802000.000 46.12657 335020000.000 65.40

34809000.000 46.12842 335090000.000 65.40

34816000.000 46.13028 335160000.000 65.40

34823000.000 46.13213 335230000.000 65.41

34830000.000 46.13399 335300000.000 65.41

34837000.000 46.13584 335370000.000 65.41

34844000.000 46.13769 335440000.000 65.42

34851000.000 46.13955 335510000.000 65.42

34858000.000 46.14140 335580000.000 65.42

34865000.000 46.14325 335650000.000 65.42

34872000.000 46.14511 335720000.000 65.43

34879000.000 46.14696 335790000.000 65.43

34886000.000 46.14881 335860000.000 65.43

34893000.000 46.15066 335930000.000 65.43

34900000.000 46.15251 336000000.000 65.44

34907000.000 46.15437 336070000.000 65.44

34914000.000 46.15622 336140000.000 65.44

34921000.000 46.15807 336210000.000 65.45

34928000.000 46.15992 336280000.000 65.45

34935000.000 46.16177 336350000.000 65.45

34942000.000 46.16362 336420000.000 65.45

34949000.000 46.16547 336490000.000 65.46

34956000.000 46.16732 336560000.000 65.46

34963000.000 46.16917 336630000.000 65.46

34970000.000 46.17101 336700000.000 65.46

34977000.000 46.17286 336770000.000 65.47

34984000.000 46.17471 336840000.000 65.47

34991000.000 46.17656 336910000.000 65.47

34998000.000 46.17841 336980000.000 65.48

35005000.000 46.18025 337050000.000 65.48

35012000.000 46.18210 337120000.000 65.48

35019000.000 46.18395 337190000.000 65.48

35026000.000 46.18579 337260000.000 65.49

35033000.000 46.18764 337330000.000 65.49

35040000.000 46.18948 337400000.000 65.49

35047000.000 46.19133 337470000.000 65.49

35054000.000 46.19317 337540000.000 65.50

35061000.000 46.19502 337610000.000 65.50

35068000.000 46.19686 337680000.000 65.50

35075000.000 46.19871 337750000.000 65.51

35082000.000 46.20055 337820000.000 65.51

35089000.000 46.20239 337890000.000 65.51

35096000.000 46.20424 337960000.000 65.51

35103000.000 46.20608 338030000.000 65.52

35110000.000 46.20792 338100000.000 65.52

35117000.000 46.20977 338170000.000 65.52

35124000.000 46.21161 338240000.000 65.52

35131000.000 46.21345 338310000.000 65.53

35138000.000 46.21529 338380000.000 65.53

35145000.000 46.21713 338450000.000 65.53

35152000.000 46.21897 338520000.000 65.54

35159000.000 46.22081 338590000.000 65.54

35166000.000 46.22265 338660000.000 65.54

35173000.000 46.22449 338730000.000 65.54

35180000.000 46.22633 338800000.000 65.55

35187000.000 46.22817 338870000.000 65.55

35194000.000 46.23001 338940000.000 65.55

35201000.000 46.23185 339010000.000 65.55

35208000.000 46.23369 339080000.000 65.56

35215000.000 46.23553 339150000.000 65.56

35222000.000 46.23737 339220000.000 65.56

35229000.000 46.23920 339290000.000 65.57

35236000.000 46.24104 339360000.000 65.57

35243000.000 46.24288 339430000.000 65.57

35250000.000 46.24472 339500000.000 65.57

35257000.000 46.24655 339570000.000 65.58

35264000.000 46.24839 339640000.000 65.58

35271000.000 46.25022 339710000.000 65.58

35278000.000 46.25206 339780000.000 65.58

35285000.000 46.25389 339850000.000 65.59

35292000.000 46.25573 339920000.000 65.59

35299000.000 46.25756 339990000.000 65.59

35306000.000 46.25940 340060000.000 65.59

35313000.000 46.26123 340130000.000 65.60

35320000.000 46.26307 340200000.000 65.60

35327000.000 46.26490 340270000.000 65.60

35334000.000 46.26673 340340000.000 65.61

35341000.000 46.26857 340410000.000 65.61

35348000.000 46.27040 340480000.000 65.61

35355000.000 46.27223 340550000.000 65.61

35362000.000 46.27406 340620000.000 65.62

35369000.000 46.27590 340690000.000 65.62

35376000.000 46.27773 340760000.000 65.62

35383000.000 46.27956 340830000.000 65.62

35390000.000 46.28139 340900000.000 65.63

35397000.000 46.28322 340970000.000 65.63

35404000.000 46.28505 341040000.000 65.63

35411000.000 46.28688 341110000.000 65.64

35418000.000 46.28871 341180000.000 65.64

35425000.000 46.29054 341250000.000 65.64

35432000.000 46.29237 341320000.000 65.64

35439000.000 46.29420 341390000.000 65.65

35446000.000 46.29603 341460000.000 65.65

35453000.000 46.29786 341530000.000 65.65

35460000.000 46.29968 341600000.000 65.65

35467000.000 46.30151 341670000.000 65.66

35474000.000 46.30334 341740000.000 65.66

35481000.000 46.30517 341810000.000 65.66

35488000.000 46.30699 341880000.000 65.66

35495000.000 46.30882 341950000.000 65.67

35502000.000 46.31065 342020000.000 65.67

35509000.000 46.31247 342090000.000 65.67

35516000.000 46.31430 342160000.000 65.68

35523000.000 46.31612 342230000.000 65.68

35530000.000 46.31795 342300000.000 65.68

35537000.000 46.31977 342370000.000 65.68

35544000.000 46.32160 342440000.000 65.69

35551000.000 46.32342 342510000.000 65.69

35558000.000 46.32525 342580000.000 65.69

35565000.000 46.32707 342650000.000 65.69

35572000.000 46.32889 342720000.000 65.70

35579000.000 46.33072 342790000.000 65.70

35586000.000 46.33254 342860000.000 65.70

35593000.000 46.33436 342930000.000 65.71

35600000.000 46.33619 343000000.000 65.71

35607000.000 46.33801 343070000.000 65.71

35614000.000 46.33983 343140000.000 65.71

35621000.000 46.34165 343210000.000 65.72

35628000.000 46.34347 343280000.000 65.72

35635000.000 46.34529 343350000.000 65.72

35642000.000 46.34711 343420000.000 65.72

35649000.000 46.34893 343490000.000 65.73

35656000.000 46.35075 343560000.000 65.73

35663000.000 46.35257 343630000.000 65.73

35670000.000 46.35439 343700000.000 65.73

35677000.000 46.35621 343770000.000 65.74

35684000.000 46.35803 343840000.000 65.74

35691000.000 46.35985 343910000.000 65.74

35698000.000 46.36167 343980000.000 65.75

35705000.000 46.36349 344050000.000 65.75

35712000.000 46.36530 344120000.000 65.75

35719000.000 46.36712 344190000.000 65.75

35726000.000 46.36894 344260000.000 65.76

35733000.000 46.37076 344330000.000 65.76

35740000.000 46.37257 344400000.000 65.76

35747000.000 46.37439 344470000.000 65.76

35754000.000 46.37621 344540000.000 65.77

35761000.000 46.37802 344610000.000 65.77

35768000.000 46.37984 344680000.000 65.77

35775000.000 46.38165 344750000.000 65.77

35782000.000 46.38347 344820000.000 65.78

35789000.000 46.38528 344890000.000 65.78

35796000.000 46.38710 344960000.000 65.78

35803000.000 46.38891 345030000.000 65.79

35810000.000 46.39072 345100000.000 65.79

35817000.000 46.39254 345170000.000 65.79

35824000.000 46.39435 345240000.000 65.79

35831000.000 46.39616 345310000.000 65.80

35838000.000 46.39798 345380000.000 65.80

35845000.000 46.39979 345450000.000 65.80

35852000.000 46.40160 345520000.000 65.80

35859000.000 46.40341 345590000.000 65.81

35866000.000 46.40522 345660000.000 65.81

35873000.000 46.40704 345730000.000 65.81

35880000.000 46.40885 345800000.000 65.81

35887000.000 46.41066 345870000.000 65.82

35894000.000 46.41247 345940000.000 65.82

35901000.000 46.41428 346010000.000 65.82

35908000.000 46.41609 346080000.000 65.83

35915000.000 46.41790 346150000.000 65.83

35922000.000 46.41971 346220000.000 65.83

35929000.000 46.42152 346290000.000 65.83

35936000.000 46.42332 346360000.000 65.84

35943000.000 46.42513 346430000.000 65.84

35950000.000 46.42694 346500000.000 65.84

35957000.000 46.42875 346570000.000 65.84

35964000.000 46.43056 346640000.000 65.85

35971000.000 46.43236 346710000.000 65.85

35978000.000 46.43417 346780000.000 65.85

35985000.000 46.43598 346850000.000 65.85

35992000.000 46.43778 346920000.000 65.86

35999000.000 46.43959 346990000.000 65.86

36006000.000 46.44140 347060000.000 65.86

36013000.000 46.44320 347130000.000 65.87

36020000.000 46.44501 347200000.000 65.87

36027000.000 46.44681 347270000.000 65.87

36034000.000 46.44862 347340000.000 65.87

36041000.000 46.45042 347410000.000 65.88

36048000.000 46.45222 347480000.000 65.88

36055000.000 46.45403 347550000.000 65.88

36062000.000 46.45583 347620000.000 65.88

36069000.000 46.45764 347690000.000 65.89

36076000.000 46.45944 347760000.000 65.89

36083000.000 46.46124 347830000.000 65.89

36090000.000 46.46304 347900000.000 65.89

36097000.000 46.46485 347970000.000 65.90

36104000.000 46.46665 348040000.000 65.90

36111000.000 46.46845 348110000.000 65.90

36118000.000 46.47025 348180000.000 65.91

36125000.000 46.47205 348250000.000 65.91

36132000.000 46.47385 348320000.000 65.91

36139000.000 46.47565 348390000.000 65.91

36146000.000 46.47745 348460000.000 65.92

36153000.000 46.47925 348530000.000 65.92

36160000.000 46.48105 348600000.000 65.92

36167000.000 46.48285 348670000.000 65.92

36174000.000 46.48465 348740000.000 65.93

36181000.000 46.48645 348810000.000 65.93

36188000.000 46.48825 348880000.000 65.93

36195000.000 46.49005 348950000.000 65.93

36202000.000 46.49185 349020000.000 65.94

36209000.000 46.49364 349090000.000 65.94

36216000.000 46.49544 349160000.000 65.94

36223000.000 46.49724 349230000.000 65.94

36230000.000 46.49904 349300000.000 65.95

36237000.000 46.50083 349370000.000 65.95

36244000.000 46.50263 349440000.000 65.95

36251000.000 46.50443 349510000.000 65.96

36258000.000 46.50622 349580000.000 65.96

36265000.000 46.50802 349650000.000 65.96

36272000.000 46.50981 349720000.000 65.96

36279000.000 46.51161 349790000.000 65.97

36286000.000 46.51340 349860000.000 65.97

36293000.000 46.51520 349930000.000 65.97

36300000.000 46.51699 350000000.000 65.97

36307000.000 46.51878 350070000.000 65.98

36314000.000 46.52058 350140000.000 65.98

36321000.000 46.52237 350210000.000 65.98

36328000.000 46.52416 350280000.000 65.98

36335000.000 46.52596 350350000.000 65.99

36342000.000 46.52775 350420000.000 65.99

36349000.000 46.52954 350490000.000 65.99

36356000.000 46.53133 350560000.000 65.99

36363000.000 46.53313 350630000.000 66.00

36370000.000 46.53492 350700000.000 66.00

36377000.000 46.53671 350770000.000 66.00

36384000.000 46.53850 350840000.000 66.01

36391000.000 46.54029 350910000.000 66.01

36398000.000 46.54208 350980000.000 66.01

36405000.000 46.54387 351050000.000 66.01

36412000.000 46.54566 351120000.000 66.02

36419000.000 46.54745 351190000.000 66.02

36426000.000 46.54924 351260000.000 66.02

36433000.000 46.55103 351330000.000 66.02

36440000.000 46.55282 351400000.000 66.03

36447000.000 46.55460 351470000.000 66.03

36454000.000 46.55639 351540000.000 66.03

36461000.000 46.55818 351610000.000 66.03

36468000.000 46.55997 351680000.000 66.04

36475000.000 46.56176 351750000.000 66.04

36482000.000 46.56354 351820000.000 66.04

36489000.000 46.56533 351890000.000 66.04

36496000.000 46.56712 351960000.000 66.05

36503000.000 46.56890 352030000.000 66.05

36510000.000 46.57069 352100000.000 66.05

36517000.000 46.57247 352170000.000 66.06

36524000.000 46.57426 352240000.000 66.06

36531000.000 46.57604 352310000.000 66.06

36538000.000 46.57783 352380000.000 66.06

36545000.000 46.57961 352450000.000 66.07

36552000.000 46.58140 352520000.000 66.07

36559000.000 46.58318 352590000.000 66.07

36566000.000 46.58497 352660000.000 66.07

36573000.000 46.58675 352730000.000 66.08

36580000.000 46.58853 352800000.000 66.08

36587000.000 46.59031 352870000.000 66.08

36594000.000 46.59210 352940000.000 66.08

36601000.000 46.59388 353010000.000 66.09

36608000.000 46.59566 353080000.000 66.09

36615000.000 46.59744 353150000.000 66.09

36622000.000 46.59923 353220000.000 66.09

36629000.000 46.60101 353290000.000 66.10

36636000.000 46.60279 353360000.000 66.10

36643000.000 46.60457 353430000.000 66.10

36650000.000 46.60635 353500000.000 66.11

36657000.000 46.60813 353570000.000 66.11

36664000.000 46.60991 353640000.000 66.11

36671000.000 46.61169 353710000.000 66.11

36678000.000 46.61347 353780000.000 66.12

36685000.000 46.61525 353850000.000 66.12

36692000.000 46.61703 353920000.000 66.12

36699000.000 46.61880 353990000.000 66.12

36706000.000 46.62058 354060000.000 66.13

36713000.000 46.62236 354130000.000 66.13

36720000.000 46.62414 354200000.000 66.13

36727000.000 46.62592 354270000.000 66.13

36734000.000 46.62769 354340000.000 66.14

36741000.000 46.62947 354410000.000 66.14

36748000.000 46.63125 354480000.000 66.14

36755000.000 46.63302 354550000.000 66.14

36762000.000 46.63480 354620000.000 66.15

36769000.000 46.63657 354690000.000 66.15

36776000.000 46.63835 354760000.000 66.15

36783000.000 46.64013 354830000.000 66.15

36790000.000 46.64190 354900000.000 66.16

36797000.000 46.64368 354970000.000 66.16

36804000.000 46.64545 355040000.000 66.16

36811000.000 46.64722 355110000.000 66.17

36818000.000 46.64900 355180000.000 66.17

36825000.000 46.65077 355250000.000 66.17

36832000.000 46.65255 355320000.000 66.17

36839000.000 46.65432 355390000.000 66.18

36846000.000 46.65609 355460000.000 66.18

36853000.000 46.65786 355530000.000 66.18

36860000.000 46.65964 355600000.000 66.18

36867000.000 46.66141 355670000.000 66.19

36874000.000 46.66318 355740000.000 66.19

36881000.000 46.66495 355810000.000 66.19

36888000.000 46.66672 355880000.000 66.19

36895000.000 46.66849 355950000.000 66.20

36902000.000 46.67026 356020000.000 66.20

36909000.000 46.67204 356090000.000 66.20

36916000.000 46.67381 356160000.000 66.20

36923000.000 46.67558 356230000.000 66.21

36930000.000 46.67735 356300000.000 66.21

36937000.000 46.67911 356370000.000 66.21

36944000.000 46.68088 356440000.000 66.21

36951000.000 46.68265 356510000.000 66.22

36958000.000 46.68442 356580000.000 66.22

36965000.000 46.68619 356650000.000 66.22

36972000.000 46.68796 356720000.000 66.23

36979000.000 46.68973 356790000.000 66.23

36986000.000 46.69149 356860000.000 66.23

36993000.000 46.69326 356930000.000 66.23

37000000.000 46.69503 357000000.000 66.24

37007000.000 46.69679 357070000.000 66.24

37014000.000 46.69856 357140000.000 66.24

37021000.000 46.70033 357210000.000 66.24

37028000.000 46.70209 357280000.000 66.25

37035000.000 46.70386 357350000.000 66.25

37042000.000 46.70562 357420000.000 66.25

37049000.000 46.70739 357490000.000 66.25

37056000.000 46.70915 357560000.000 66.26

37063000.000 46.71092 357630000.000 66.26

37070000.000 46.71268 357700000.000 66.26

37077000.000 46.71445 357770000.000 66.26

37084000.000 46.71621 357840000.000 66.27

37091000.000 46.71797 357910000.000 66.27

37098000.000 46.71974 357980000.000 66.27

37105000.000 46.72150 358050000.000 66.27

37112000.000 46.72326 358120000.000 66.28

37119000.000 46.72502 358190000.000 66.28

37126000.000 46.72679 358260000.000 66.28

37133000.000 46.72855 358330000.000 66.28

37140000.000 46.73031 358400000.000 66.29

37147000.000 46.73207 358470000.000 66.29

37154000.000 46.73383 358540000.000 66.29

37161000.000 46.73559 358610000.000 66.30

37168000.000 46.73735 358680000.000 66.30

37175000.000 46.73911 358750000.000 66.30

37182000.000 46.74087 358820000.000 66.30

37189000.000 46.74263 358890000.000 66.31

37196000.000 46.74439 358960000.000 66.31

37203000.000 46.74615 359030000.000 66.31

37210000.000 46.74791 359100000.000 66.31

37217000.000 46.74967 359170000.000 66.32

37224000.000 46.75143 359240000.000 66.32

37231000.000 46.75319 359310000.000 66.32

37238000.000 46.75495 359380000.000 66.32

37245000.000 46.75670 359450000.000 66.33

37252000.000 46.75846 359520000.000 66.33

37259000.000 46.76022 359590000.000 66.33

37266000.000 46.76197 359660000.000 66.33

37273000.000 46.76373 359730000.000 66.34

37280000.000 46.76549 359800000.000 66.34

37287000.000 46.76724 359870000.000 66.34

37294000.000 46.76900 359940000.000 66.34

37301000.000 46.77075 360010000.000 66.35

37308000.000 46.77251 360080000.000 66.35

37315000.000 46.77427 360150000.000 66.35

37322000.000 46.77602 360220000.000 66.35

37329000.000 46.77777 360290000.000 66.36

37336000.000 46.77953 360360000.000 66.36

37343000.000 46.78128 360430000.000 66.36

37350000.000 46.78304 360500000.000 66.36

37357000.000 46.78479 360570000.000 66.37

37364000.000 46.78654 360640000.000 66.37

37371000.000 46.78830 360710000.000 66.37

37378000.000 46.79005 360780000.000 66.38

37385000.000 46.79180 360850000.000 66.38

37392000.000 46.79355 360920000.000 66.38

37399000.000 46.79530 360990000.000 66.38

37406000.000 46.79706 361060000.000 66.39

37413000.000 46.79881 361130000.000 66.39

37420000.000 46.80056 361200000.000 66.39

37427000.000 46.80231 361270000.000 66.39

37434000.000 46.80406 361340000.000 66.40

37441000.000 46.80581 361410000.000 66.40

37448000.000 46.80756 361480000.000 66.40

37455000.000 46.80931 361550000.000 66.40

37462000.000 46.81106 361620000.000 66.41

37469000.000 46.81281 361690000.000 66.41

37476000.000 46.81456 361760000.000 66.41

37483000.000 46.81631 361830000.000 66.41

37490000.000 46.81806 361900000.000 66.42

37497000.000 46.81980 361970000.000 66.42

37504000.000 46.82155 362040000.000 66.42

37511000.000 46.82330 362110000.000 66.42

37518000.000 46.82505 362180000.000 66.43

37525000.000 46.82679 362250000.000 66.43

37532000.000 46.82854 362320000.000 66.43

37539000.000 46.83029 362390000.000 66.43

37546000.000 46.83203 362460000.000 66.44

37553000.000 46.83378 362530000.000 66.44

37560000.000 46.83553 362600000.000 66.44

37567000.000 46.83727 362670000.000 66.44

37574000.000 46.83902 362740000.000 66.45

37581000.000 46.84076 362810000.000 66.45

37588000.000 46.84251 362880000.000 66.45

37595000.000 46.84425 362950000.000 66.45

37602000.000 46.84600 363020000.000 66.46

37609000.000 46.84774 363090000.000 66.46

37616000.000 46.84948 363160000.000 66.46

37623000.000 46.85123 363230000.000 66.47

37630000.000 46.85297 363300000.000 66.47

37637000.000 46.85471 363370000.000 66.47

37644000.000 46.85646 363440000.000 66.47

37651000.000 46.85820 363510000.000 66.48

37658000.000 46.85994 363580000.000 66.48

37665000.000 46.86168 363650000.000 66.48

37672000.000 46.86342 363720000.000 66.48

37679000.000 46.86517 363790000.000 66.49

37686000.000 46.86691 363860000.000 66.49

37693000.000 46.86865 363930000.000 66.49

37700000.000 46.87039 364000000.000 66.49

37707000.000 46.87213 364070000.000 66.50

37714000.000 46.87387 364140000.000 66.50

37721000.000 46.87561 364210000.000 66.50

37728000.000 46.87735 364280000.000 66.50

37735000.000 46.87909 364350000.000 66.51

37742000.000 46.88083 364420000.000 66.51

37749000.000 46.88257 364490000.000 66.51

37756000.000 46.88430 364560000.000 66.51

37763000.000 46.88604 364630000.000 66.52

37770000.000 46.88778 364700000.000 66.52

37777000.000 46.88952 364770000.000 66.52

37784000.000 46.89126 364840000.000 66.52

37791000.000 46.89299 364910000.000 66.53

37798000.000 46.89473 364980000.000 66.53

37805000.000 46.89647 365050000.000 66.53

37812000.000 46.89820 365120000.000 66.53

37819000.000 46.89994 365190000.000 66.54

37826000.000 46.90168 365260000.000 66.54

37833000.000 46.90341 365330000.000 66.54

37840000.000 46.90515 365400000.000 66.54

37847000.000 46.90688 365470000.000 66.55

37854000.000 46.90862 365540000.000 66.55

37861000.000 46.91035 365610000.000 66.55

37868000.000 46.91209 365680000.000 66.55

37875000.000 46.91382 365750000.000 66.56

37882000.000 46.91556 365820000.000 66.56

37889000.000 46.91729 365890000.000 66.56

37896000.000 46.91902 365960000.000 66.56

37903000.000 46.92076 366030000.000 66.57

37910000.000 46.92249 366100000.000 66.57

37917000.000 46.92422 366170000.000 66.57

37924000.000 46.92595 366240000.000 66.57

37931000.000 46.92769 366310000.000 66.58

37938000.000 46.92942 366380000.000 66.58

37945000.000 46.93115 366450000.000 66.58

37952000.000 46.93288 366520000.000 66.59

37959000.000 46.93461 366590000.000 66.59

37966000.000 46.93634 366660000.000 66.59

37973000.000 46.93807 366730000.000 66.59

37980000.000 46.93980 366800000.000 66.60

37987000.000 46.94153 366870000.000 66.60

37994000.000 46.94326 366940000.000 66.60

38001000.000 46.94499 367010000.000 66.60

38008000.000 46.94672 367080000.000 66.61

38015000.000 46.94845 367150000.000 66.61

38022000.000 46.95018 367220000.000 66.61

38029000.000 46.95191 367290000.000 66.61

38036000.000 46.95364 367360000.000 66.62

38043000.000 46.95537 367430000.000 66.62

38050000.000 46.95709 367500000.000 66.62

38057000.000 46.95882 367570000.000 66.62

38064000.000 46.96055 367640000.000 66.63

38071000.000 46.96228 367710000.000 66.63

38078000.000 46.96400 367780000.000 66.63

38085000.000 46.96573 367850000.000 66.63

38092000.000 46.96746 367920000.000 66.64

38099000.000 46.96918 367990000.000 66.64

38106000.000 46.97091 368060000.000 66.64

38113000.000 46.97263 368130000.000 66.64

38120000.000 46.97436 368200000.000 66.65

38127000.000 46.97608 368270000.000 66.65

38134000.000 46.97781 368340000.000 66.65

38141000.000 46.97953 368410000.000 66.65

38148000.000 46.98126 368480000.000 66.66

38155000.000 46.98298 368550000.000 66.66

38162000.000 46.98471 368620000.000 66.66

38169000.000 46.98643 368690000.000 66.66

38176000.000 46.98815 368760000.000 66.67

38183000.000 46.98988 368830000.000 66.67

38190000.000 46.99160 368900000.000 66.67

38197000.000 46.99332 368970000.000 66.67

38204000.000 46.99504 369040000.000 66.68

38211000.000 46.99677 369110000.000 66.68

38218000.000 46.99849 369180000.000 66.68

38225000.000 47.00021 369250000.000 66.68

38232000.000 47.00193 369320000.000 66.69

38239000.000 47.00365 369390000.000 66.69

38246000.000 47.00537 369460000.000 66.69

38253000.000 47.00709 369530000.000 66.69

38260000.000 47.00881 369600000.000 66.70

38267000.000 47.01053 369670000.000 66.70

38274000.000 47.01225 369740000.000 66.70

38281000.000 47.01397 369810000.000 66.70

38288000.000 47.01569 369880000.000 66.71

38295000.000 47.01741 369950000.000 66.71

38302000.000 47.01913 370020000.000 66.71

38309000.000 47.02085 370090000.000 66.71

38316000.000 47.02257 370160000.000 66.72

38323000.000 47.02428 370230000.000 66.72

38330000.000 47.02600 370300000.000 66.72

38337000.000 47.02772 370370000.000 66.72

38344000.000 47.02944 370440000.000 66.73

38351000.000 47.03115 370510000.000 66.73

38358000.000 47.03287 370580000.000 66.73

38365000.000 47.03459 370650000.000 66.73

38372000.000 47.03630 370720000.000 66.74

38379000.000 47.03802 370790000.000 66.74

38386000.000 47.03973 370860000.000 66.74

38393000.000 47.04145 370930000.000 66.74

38400000.000 47.04316 371000000.000 66.75

38407000.000 47.04488 371070000.000 66.75

38414000.000 47.04659 371140000.000 66.75

38421000.000 47.04831 371210000.000 66.75

38428000.000 47.05002 371280000.000 66.76

38435000.000 47.05174 371350000.000 66.76

38442000.000 47.05345 371420000.000 66.76

38449000.000 47.05516 371490000.000 66.76

38456000.000 47.05688 371560000.000 66.77

38463000.000 47.05859 371630000.000 66.77

38470000.000 47.06030 371700000.000 66.77

38477000.000 47.06202 371770000.000 66.77

38484000.000 47.06373 371840000.000 66.78

38491000.000 47.06544 371910000.000 66.78

38498000.000 47.06715 371980000.000 66.78

38505000.000 47.06886 372050000.000 66.78

38512000.000 47.07057 372120000.000 66.79

38519000.000 47.07229 372190000.000 66.79

38526000.000 47.07400 372260000.000 66.79

38533000.000 47.07571 372330000.000 66.79

38540000.000 47.07742 372400000.000 66.80

38547000.000 47.07913 372470000.000 66.80

38554000.000 47.08084 372540000.000 66.80

38561000.000 47.08255 372610000.000 66.80

38568000.000 47.08426 372680000.000 66.81

38575000.000 47.08596 372750000.000 66.81

38582000.000 47.08767 372820000.000 66.81

38589000.000 47.08938 372890000.000 66.82

38596000.000 47.09109 372960000.000 66.82

38603000.000 47.09280 373030000.000 66.82

38610000.000 47.09451 373100000.000 66.82

38617000.000 47.09621 373170000.000 66.83

38624000.000 47.09792 373240000.000 66.83

38631000.000 47.09963 373310000.000 66.83

38638000.000 47.10133 373380000.000 66.83

38645000.000 47.10304 373450000.000 66.84

38652000.000 47.10475 373520000.000 66.84

38659000.000 47.10645 373590000.000 66.84

38666000.000 47.10816 373660000.000 66.84

38673000.000 47.10986 373730000.000 66.85

38680000.000 47.11157 373800000.000 66.85

38687000.000 47.11328 373870000.000 66.85

38694000.000 47.11498 373940000.000 66.85

38701000.000 47.11668 374010000.000 66.86

38708000.000 47.11839 374080000.000 66.86

38715000.000 47.12009 374150000.000 66.86

38722000.000 47.12180 374220000.000 66.86

38729000.000 47.12350 374290000.000 66.87

38736000.000 47.12520 374360000.000 66.87

38743000.000 47.12691 374430000.000 66.87

38750000.000 47.12861 374500000.000 66.87

38757000.000 47.13031 374570000.000 66.88

38764000.000 47.13201 374640000.000 66.88

38771000.000 47.13372 374710000.000 66.88

38778000.000 47.13542 374780000.000 66.88

38785000.000 47.13712 374850000.000 66.89

38792000.000 47.13882 374920000.000 66.89

38799000.000 47.14052 374990000.000 66.89

38806000.000 47.14222 375060000.000 66.89

38813000.000 47.14392 375130000.000 66.90

38820000.000 47.14562 375200000.000 66.90

38827000.000 47.14732 375270000.000 66.90

38834000.000 47.14902 375340000.000 66.90

38841000.000 47.15072 375410000.000 66.91

38848000.000 47.15242 375480000.000 66.91

38855000.000 47.15412 375550000.000 66.91

38862000.000 47.15582 375620000.000 66.91

38869000.000 47.15752 375690000.000 66.92

38876000.000 47.15922 375760000.000 66.92

38883000.000 47.16092 375830000.000 66.92

38890000.000 47.16261 375900000.000 66.92

38897000.000 47.16431 375970000.000 66.93

38904000.000 47.16601 376040000.000 66.93

38911000.000 47.16771 376110000.000 66.93

38918000.000 47.16940 376180000.000 66.93

38925000.000 47.17110 376250000.000 66.94

38932000.000 47.17280 376320000.000 66.94

38939000.000 47.17449 376390000.000 66.94

38946000.000 47.17619 376460000.000 66.94

38953000.000 47.17788 376530000.000 66.94

38960000.000 47.17958 376600000.000 66.95

38967000.000 47.18128 376670000.000 66.95

38974000.000 47.18297 376740000.000 66.95

38981000.000 47.18467 376810000.000 66.95

38988000.000 47.18636 376880000.000 66.96

38995000.000 47.18805 376950000.000 66.96

39002000.000 47.18975 377020000.000 66.96

39009000.000 47.19144 377090000.000 66.96

39016000.000 47.19314 377160000.000 66.97

39023000.000 47.19483 377230000.000 66.97

39030000.000 47.19652 377300000.000 66.97

39037000.000 47.19821 377370000.000 66.97

39044000.000 47.19991 377440000.000 66.98

39051000.000 47.20160 377510000.000 66.98

39058000.000 47.20329 377580000.000 66.98

39065000.000 47.20498 377650000.000 66.98

39072000.000 47.20668 377720000.000 66.99

39079000.000 47.20837 377790000.000 66.99

39086000.000 47.21006 377860000.000 66.99

39093000.000 47.21175 377930000.000 66.99

39100000.000 47.21344 378000000.000 67.00

39107000.000 47.21513 378070000.000 67.00

39114000.000 47.21682 378140000.000 67.00

39121000.000 47.21851 378210000.000 67.00

39128000.000 47.22020 378280000.000 67.01

39135000.000 47.22189 378350000.000 67.01

39142000.000 47.22358 378420000.000 67.01

39149000.000 47.22527 378490000.000 67.01

39156000.000 47.22696 378560000.000 67.02

39163000.000 47.22864 378630000.000 67.02

39170000.000 47.23033 378700000.000 67.02

39177000.000 47.23202 378770000.000 67.02

39184000.000 47.23371 378840000.000 67.03

39191000.000 47.23540 378910000.000 67.03

39198000.000 47.23708 378980000.000 67.03

39205000.000 47.23877 379050000.000 67.03

39212000.000 47.24046 379120000.000 67.04

39219000.000 47.24214 379190000.000 67.04

39226000.000 47.24383 379260000.000 67.04

39233000.000 47.24552 379330000.000 67.04

39240000.000 47.24720 379400000.000 67.05

39247000.000 47.24889 379470000.000 67.05

39254000.000 47.25057 379540000.000 67.05

39261000.000 47.25226 379610000.000 67.05

39268000.000 47.25394 379680000.000 67.06

39275000.000 47.25563 379750000.000 67.06

39282000.000 47.25731 379820000.000 67.06

39289000.000 47.25899 379890000.000 67.06

39296000.000 47.26068 379960000.000 67.07

39303000.000 47.26236 380030000.000 67.07

39310000.000 47.26405 380100000.000 67.07

39317000.000 47.26573 380170000.000 67.07

39324000.000 47.26741 380240000.000 67.08

39331000.000 47.26909 380310000.000 67.08

39338000.000 47.27078 380380000.000 67.08

39345000.000 47.27246 380450000.000 67.08

39352000.000 47.27414 380520000.000 67.09

39359000.000 47.27582 380590000.000 67.09

39366000.000 47.27750 380660000.000 67.09

39373000.000 47.27919 380730000.000 67.09

39380000.000 47.28087 380800000.000 67.10

39387000.000 47.28255 380870000.000 67.10

39394000.000 47.28423 380940000.000 67.10

39401000.000 47.28591 381010000.000 67.10

39408000.000 47.28759 381080000.000 67.11

39415000.000 47.28927 381150000.000 67.11

39422000.000 47.29095 381220000.000 67.11

39429000.000 47.29263 381290000.000 67.11

39436000.000 47.29431 381360000.000 67.12

39443000.000 47.29598 381430000.000 67.12

39450000.000 47.29766 381500000.000 67.12

39457000.000 47.29934 381570000.000 67.12

39464000.000 47.30102 381640000.000 67.13

39471000.000 47.30270 381710000.000 67.13

39478000.000 47.30438 381780000.000 67.13

39485000.000 47.30605 381850000.000 67.13

39492000.000 47.30773 381920000.000 67.14

39499000.000 47.30941 381990000.000 67.14

39506000.000 47.31108 382060000.000 67.14

39513000.000 47.31276 382130000.000 67.14

39520000.000 47.31444 382200000.000 67.15

39527000.000 47.31611 382270000.000 67.15

39534000.000 47.31779 382340000.000 67.15

39541000.000 47.31946 382410000.000 67.15

39548000.000 47.32114 382480000.000 67.16

39555000.000 47.32281 382550000.000 67.16

39562000.000 47.32449 382620000.000 67.16

39569000.000 47.32616 382690000.000 67.16

39576000.000 47.32784 382760000.000 67.17

39583000.000 47.32951 382830000.000 67.17

39590000.000 47.33119 382900000.000 67.17

39597000.000 47.33286 382970000.000 67.17

39604000.000 47.33453 383040000.000 67.17

39611000.000 47.33621 383110000.000 67.18

39618000.000 47.33788 383180000.000 67.18

39625000.000 47.33955 383250000.000 67.18

39632000.000 47.34122 383320000.000 67.18

39639000.000 47.34290 383390000.000 67.19

39646000.000 47.34457 383460000.000 67.19

39653000.000 47.34624 383530000.000 67.19

39660000.000 47.34791 383600000.000 67.19

39667000.000 47.34958 383670000.000 67.20

39674000.000 47.35125 383740000.000 67.20

39681000.000 47.35292 383810000.000 67.20

39688000.000 47.35460 383880000.000 67.20

39695000.000 47.35627 383950000.000 67.21

39702000.000 47.35794 384020000.000 67.21

39709000.000 47.35961 384090000.000 67.21

39716000.000 47.36127 384160000.000 67.21

39723000.000 47.36294 384230000.000 67.22

39730000.000 47.36461 384300000.000 67.22

39737000.000 47.36628 384370000.000 67.22

39744000.000 47.36795 384440000.000 67.22

39751000.000 47.36962 384510000.000 67.23

39758000.000 47.37129 384580000.000 67.23

39765000.000 47.37296 384650000.000 67.23

39772000.000 47.37462 384720000.000 67.23

39779000.000 47.37629 384790000.000 67.24

39786000.000 47.37796 384860000.000 67.24

39793000.000 47.37963 384930000.000 67.24

39800000.000 47.38129 385000000.000 67.24

39807000.000 47.38296 385070000.000 67.25

39814000.000 47.38462 385140000.000 67.25

39821000.000 47.38629 385210000.000 67.25

39828000.000 47.38796 385280000.000 67.25

39835000.000 47.38962 385350000.000 67.26

39842000.000 47.39129 385420000.000 67.26

39849000.000 47.39295 385490000.000 67.26

39856000.000 47.39462 385560000.000 67.26

39863000.000 47.39628 385630000.000 67.27

39870000.000 47.39795 385700000.000 67.27

39877000.000 47.39961 385770000.000 67.27

39884000.000 47.40128 385840000.000 67.27

39891000.000 47.40294 385910000.000 67.28

39898000.000 47.40460 385980000.000 67.28

39905000.000 47.40627 386050000.000 67.28

39912000.000 47.40793 386120000.000 67.28

39919000.000 47.40959 386190000.000 67.28

39926000.000 47.41125 386260000.000 67.29

39933000.000 47.41292 386330000.000 67.29

39940000.000 47.41458 386400000.000 67.29

39947000.000 47.41624 386470000.000 67.29

39954000.000 47.41790 386540000.000 67.30

39961000.000 47.41956 386610000.000 67.30

39968000.000 47.42122 386680000.000 67.30

39975000.000 47.42289 386750000.000 67.30

39982000.000 47.42455 386820000.000 67.31

39989000.000 47.42621 386890000.000 67.31

39996000.000 47.42787 386960000.000 67.31

40003000.000 47.42953 387030000.000 67.31

40010000.000 47.43119 387100000.000 67.32

40017000.000 47.43285 387170000.000 67.32

40024000.000 47.43451 387240000.000 67.32

40031000.000 47.43617 387310000.000 67.32

40038000.000 47.43782 387380000.000 67.33

40045000.000 47.43948 387450000.000 67.33

40052000.000 47.44114 387520000.000 67.33

40059000.000 47.44280 387590000.000 67.33

40066000.000 47.44446 387660000.000 67.34

40073000.000 47.44611 387730000.000 67.34

40080000.000 47.44777 387800000.000 67.34

40087000.000 47.44943 387870000.000 67.34

40094000.000 47.45109 387940000.000 67.35

40101000.000 47.45274 388010000.000 67.35

40108000.000 47.45440 388080000.000 67.35

40115000.000 47.45606 388150000.000 67.35

40122000.000 47.45771 388220000.000 67.36

40129000.000 47.45937 388290000.000 67.36

40136000.000 47.46102 388360000.000 67.36

40143000.000 47.46268 388430000.000 67.36

40150000.000 47.46433 388500000.000 67.37

40157000.000 47.46599 388570000.000 67.37

40164000.000 47.46764 388640000.000 67.37

40171000.000 47.46930 388710000.000 67.37

40178000.000 47.47095 388780000.000 67.38

40185000.000 47.47261 388850000.000 67.38

40192000.000 47.47426 388920000.000 67.38

40199000.000 47.47591 388990000.000 67.38

40206000.000 47.47757 389060000.000 67.38

40213000.000 47.47922 389130000.000 67.39

40220000.000 47.48087 389200000.000 67.39

40227000.000 47.48253 389270000.000 67.39

40234000.000 47.48418 389340000.000 67.39

40241000.000 47.48583 389410000.000 67.40

40248000.000 47.48748 389480000.000 67.40

40255000.000 47.48913 389550000.000 67.40

40262000.000 47.49079 389620000.000 67.40

40269000.000 47.49244 389690000.000 67.41

40276000.000 47.49409 389760000.000 67.41

40283000.000 47.49574 389830000.000 67.41

40290000.000 47.49739 389900000.000 67.41

40297000.000 47.49904 389970000.000 67.42

40304000.000 47.50069 390040000.000 67.42

40311000.000 47.50234 390110000.000 67.42

40318000.000 47.50399 390180000.000 67.42

40325000.000 47.50564 390250000.000 67.43

40332000.000 47.50729 390320000.000 67.43

40339000.000 47.50894 390390000.000 67.43

40346000.000 47.51059 390460000.000 67.43

40353000.000 47.51223 390530000.000 67.44

40360000.000 47.51388 390600000.000 67.44

40367000.000 47.51553 390670000.000 67.44

40374000.000 47.51718 390740000.000 67.44

40381000.000 47.51883 390810000.000 67.45

40388000.000 47.52047 390880000.000 67.45

40395000.000 47.52212 390950000.000 67.45

40402000.000 47.52377 391020000.000 67.45

40409000.000 47.52541 391090000.000 67.45

40416000.000 47.52706 391160000.000 67.46

40423000.000 47.52871 391230000.000 67.46

40430000.000 47.53035 391300000.000 67.46

40437000.000 47.53200 391370000.000 67.46

40444000.000 47.53364 391440000.000 67.47

40451000.000 47.53529 391510000.000 67.47

40458000.000 47.53693 391580000.000 67.47

40465000.000 47.53858 391650000.000 67.47

40472000.000 47.54022 391720000.000 67.48

40479000.000 47.54187 391790000.000 67.48

40486000.000 47.54351 391860000.000 67.48

40493000.000 47.54516 391930000.000 67.48

40500000.000 47.54680 392000000.000 67.49

40507000.000 47.54844 392070000.000 67.49

40514000.000 47.55009 392140000.000 67.49

40521000.000 47.55173 392210000.000 67.49

40528000.000 47.55337 392280000.000 67.50

40535000.000 47.55501 392350000.000 67.50

40542000.000 47.55666 392420000.000 67.50

40549000.000 47.55830 392490000.000 67.50

40556000.000 47.55994 392560000.000 67.51

40563000.000 47.56158 392630000.000 67.51

40570000.000 47.56322 392700000.000 67.51

40577000.000 47.56487 392770000.000 67.51

40584000.000 47.56651 392840000.000 67.52

40591000.000 47.56815 392910000.000 67.52

40598000.000 47.56979 392980000.000 67.52

40605000.000 47.57143 393050000.000 67.52

40612000.000 47.57307 393120000.000 67.52

40619000.000 47.57471 393190000.000 67.53

40626000.000 47.57635 393260000.000 67.53

40633000.000 47.57799 393330000.000 67.53

40640000.000 47.57963 393400000.000 67.53

40647000.000 47.58126 393470000.000 67.54

40654000.000 47.58290 393540000.000 67.54

40661000.000 47.58454 393610000.000 67.54

40668000.000 47.58618 393680000.000 67.54

40675000.000 47.58782 393750000.000 67.55

40682000.000 47.58946 393820000.000 67.55

40689000.000 47.59109 393890000.000 67.55

40696000.000 47.59273 393960000.000 67.55

40703000.000 47.59437 394030000.000 67.56

40710000.000 47.59601 394100000.000 67.56

40717000.000 47.59764 394170000.000 67.56

40724000.000 47.59928 394240000.000 67.56

40731000.000 47.60091 394310000.000 67.57

40738000.000 47.60255 394380000.000 67.57

40745000.000 47.60419 394450000.000 67.57

40752000.000 47.60582 394520000.000 67.57

40759000.000 47.60746 394590000.000 67.58

40766000.000 47.60909 394660000.000 67.58

40773000.000 47.61073 394730000.000 67.58

40780000.000 47.61236 394800000.000 67.58

40787000.000 47.61400 394870000.000 67.58

40794000.000 47.61563 394940000.000 67.59

40801000.000 47.61726 395010000.000 67.59

40808000.000 47.61890 395080000.000 67.59

40815000.000 47.62053 395150000.000 67.59

40822000.000 47.62217 395220000.000 67.60

40829000.000 47.62380 395290000.000 67.60

40836000.000 47.62543 395360000.000 67.60

40843000.000 47.62706 395430000.000 67.60

40850000.000 47.62870 395500000.000 67.61

40857000.000 47.63033 395570000.000 67.61

40864000.000 47.63196 395640000.000 67.61

40871000.000 47.63359 395710000.000 67.61

40878000.000 47.63522 395780000.000 67.62

40885000.000 47.63686 395850000.000 67.62

40892000.000 47.63849 395920000.000 67.62

40899000.000 47.64012 395990000.000 67.62

40906000.000 47.64175 396060000.000 67.63

40913000.000 47.64338 396130000.000 67.63

40920000.000 47.64501 396200000.000 67.63

40927000.000 47.64664 396270000.000 67.63

40934000.000 47.64827 396340000.000 67.64

40941000.000 47.64990 396410000.000 67.64

40948000.000 47.65153 396480000.000 67.64

40955000.000 47.65316 396550000.000 67.64

40962000.000 47.65479 396620000.000 67.64

40969000.000 47.65641 396690000.000 67.65

40976000.000 47.65804 396760000.000 67.65

40983000.000 47.65967 396830000.000 67.65

40990000.000 47.66130 396900000.000 67.65

40997000.000 47.66293 396970000.000 67.66

41004000.000 47.66455 397040000.000 67.66

41011000.000 47.66618 397110000.000 67.66

41018000.000 47.66781 397180000.000 67.66

41025000.000 47.66943 397250000.000 67.67

41032000.000 47.67106 397320000.000 67.67

41039000.000 47.67269 397390000.000 67.67

41046000.000 47.67431 397460000.000 67.67

41053000.000 47.67594 397530000.000 67.68

41060000.000 47.67757 397600000.000 67.68

41067000.000 47.67919 397670000.000 67.68

41074000.000 47.68082 397740000.000 67.68

41081000.000 47.68244 397810000.000 67.69

41088000.000 47.68407 397880000.000 67.69

41095000.000 47.68569 397950000.000 67.69

41102000.000 47.68732 398020000.000 67.69

41109000.000 47.68894 398090000.000 67.69

41116000.000 47.69056 398160000.000 67.70

41123000.000 47.69219 398230000.000 67.70

41130000.000 47.69381 398300000.000 67.70

41137000.000 47.69543 398370000.000 67.70

41144000.000 47.69706 398440000.000 67.71

41151000.000 47.69868 398510000.000 67.71

41158000.000 47.70030 398580000.000 67.71

41165000.000 47.70193 398650000.000 67.71

41172000.000 47.70355 398720000.000 67.72

41179000.000 47.70517 398790000.000 67.72

41186000.000 47.70679 398860000.000 67.72

41193000.000 47.70841 398930000.000 67.72

41200000.000 47.71003 399000000.000 67.73

41207000.000 47.71166 399070000.000 67.73

41214000.000 47.71328 399140000.000 67.73

41221000.000 47.71490 399210000.000 67.73

41228000.000 47.71652 399280000.000 67.74

41235000.000 47.71814 399350000.000 67.74

41242000.000 47.71976 399420000.000 67.74

41249000.000 47.72138 399490000.000 67.74

41256000.000 47.72300 399560000.000 67.74

41263000.000 47.72462 399630000.000 67.75

41270000.000 47.72624 399700000.000 67.75

41277000.000 47.72785 399770000.000 67.75

41284000.000 47.72947 399840000.000 67.75

41291000.000 47.73109 399910000.000 67.76

41298000.000 47.73271 399980000.000 67.76

41305000.000 47.73433 400050000.000 67.76

41312000.000 47.73595 400120000.000 67.76

41319000.000 47.73756 400190000.000 67.77

41326000.000 47.73918 400260000.000 67.77

41333000.000 47.74080 400330000.000 67.77

41340000.000 47.74241 400400000.000 67.77

41347000.000 47.74403 400470000.000 67.78

41354000.000 47.74565 400540000.000 67.78

41361000.000 47.74726 400610000.000 67.78

41368000.000 47.74888 400680000.000 67.78

41375000.000 47.75050 400750000.000 67.78

41382000.000 47.75211 400820000.000 67.79

41389000.000 47.75373 400890000.000 67.79

41396000.000 47.75534 400960000.000 67.79

41403000.000 47.75696 401030000.000 67.79

41410000.000 47.75857 401100000.000 67.80

41417000.000 47.76019 401170000.000 67.80

41424000.000 47.76180 401240000.000 67.80

41431000.000 47.76341 401310000.000 67.80

41438000.000 47.76503 401380000.000 67.81

41445000.000 47.76664 401450000.000 67.81

41452000.000 47.76826 401520000.000 67.81

41459000.000 47.76987 401590000.000 67.81

41466000.000 47.77148 401660000.000 67.82

41473000.000 47.77309 401730000.000 67.82

41480000.000 47.77471 401800000.000 67.82

41487000.000 47.77632 401870000.000 67.82

41494000.000 47.77793 401940000.000 67.83

41501000.000 47.77954 402010000.000 67.83

41508000.000 47.78116 402080000.000 67.83

41515000.000 47.78277 402150000.000 67.83

41522000.000 47.78438 402220000.000 67.83

41529000.000 47.78599 402290000.000 67.84

41536000.000 47.78760 402360000.000 67.84

41543000.000 47.78921 402430000.000 67.84

41550000.000 47.79082 402500000.000 67.84

41557000.000 47.79243 402570000.000 67.85

41564000.000 47.79404 402640000.000 67.85

41571000.000 47.79565 402710000.000 67.85

41578000.000 47.79726 402780000.000 67.85

41585000.000 47.79887 402850000.000 67.86

41592000.000 47.80048 402920000.000 67.86

41599000.000 47.80209 402990000.000 67.86

41606000.000 47.80370 403060000.000 67.86

41613000.000 47.80530 403130000.000 67.87

41620000.000 47.80691 403200000.000 67.87

41627000.000 47.80852 403270000.000 67.87

41634000.000 47.81013 403340000.000 67.87

41641000.000 47.81174 403410000.000 67.87

41648000.000 47.81334 403480000.000 67.88

41655000.000 47.81495 403550000.000 67.88

41662000.000 47.81656 403620000.000 67.88

41669000.000 47.81816 403690000.000 67.88

41676000.000 47.81977 403760000.000 67.89

41683000.000 47.82138 403830000.000 67.89

41690000.000 47.82298 403900000.000 67.89

41697000.000 47.82459 403970000.000 67.89

41704000.000 47.82619 404040000.000 67.90

41711000.000 47.82780 404110000.000 67.90

41718000.000 47.82941 404180000.000 67.90

41725000.000 47.83101 404250000.000 67.90

41732000.000 47.83261 404320000.000 67.91

41739000.000 47.83422 404390000.000 67.91

41746000.000 47.83582 404460000.000 67.91

41753000.000 47.83743 404530000.000 67.91

41760000.000 47.83903 404600000.000 67.91

41767000.000 47.84064 404670000.000 67.92

41774000.000 47.84224 404740000.000 67.92

41781000.000 47.84384 404810000.000 67.92

41788000.000 47.84545 404880000.000 67.92

41795000.000 47.84705 404950000.000 67.93

41802000.000 47.84865 405020000.000 67.93

41809000.000 47.85025 405090000.000 67.93

41816000.000 47.85186 405160000.000 67.93

41823000.000 47.85346 405230000.000 67.94

41830000.000 47.85506 405300000.000 67.94

41837000.000 47.85666 405370000.000 67.94

41844000.000 47.85826 405440000.000 67.94

41851000.000 47.85986 405510000.000 67.95

41858000.000 47.86146 405580000.000 67.95

41865000.000 47.86306 405650000.000 67.95

41872000.000 47.86467 405720000.000 67.95

41879000.000 47.86627 405790000.000 67.95

41886000.000 47.86787 405860000.000 67.96

41893000.000 47.86947 405930000.000 67.96

41900000.000 47.87106 406000000.000 67.96

41907000.000 47.87266 406070000.000 67.96

41914000.000 47.87426 406140000.000 67.97

41921000.000 47.87586 406210000.000 67.97

41928000.000 47.87746 406280000.000 67.97

41935000.000 47.87906 406350000.000 67.97

41942000.000 47.88066 406420000.000 67.98

41949000.000 47.88226 406490000.000 67.98

41956000.000 47.88385 406560000.000 67.98

41963000.000 47.88545 406630000.000 67.98

41970000.000 47.88705 406700000.000 67.98

41977000.000 47.88865 406770000.000 67.99

41984000.000 47.89024 406840000.000 67.99

41991000.000 47.89184 406910000.000 67.99

41998000.000 47.89344 406980000.000 67.99

42005000.000 47.89503 407050000.000 68.00

42012000.000 47.89663 407120000.000 68.00

42019000.000 47.89823 407190000.000 68.00

42026000.000 47.89982 407260000.000 68.00

42033000.000 47.90142 407330000.000 68.01

42040000.000 47.90301 407400000.000 68.01

42047000.000 47.90461 407470000.000 68.01

42054000.000 47.90620 407540000.000 68.01

42061000.000 47.90780 407610000.000 68.02

42068000.000 47.90939 407680000.000 68.02

42075000.000 47.91099 407750000.000 68.02

42082000.000 47.91258 407820000.000 68.02

42089000.000 47.91417 407890000.000 68.02

42096000.000 47.91577 407960000.000 68.03

42103000.000 47.91736 408030000.000 68.03

42110000.000 47.91895 408100000.000 68.03

42117000.000 47.92055 408170000.000 68.03

42124000.000 47.92214 408240000.000 68.04

42131000.000 47.92373 408310000.000 68.04

42138000.000 47.92533 408380000.000 68.04

42145000.000 47.92692 408450000.000 68.04

42152000.000 47.92851 408520000.000 68.05

42159000.000 47.93010 408590000.000 68.05

42166000.000 47.93169 408660000.000 68.05

42173000.000 47.93328 408730000.000 68.05

42180000.000 47.93488 408800000.000 68.06

42187000.000 47.93647 408870000.000 68.06

42194000.000 47.93806 408940000.000 68.06

42201000.000 47.93965 409010000.000 68.06

42208000.000 47.94124 409080000.000 68.06

42215000.000 47.94283 409150000.000 68.07

42222000.000 47.94442 409220000.000 68.07

42229000.000 47.94601 409290000.000 68.07

42236000.000 47.94760 409360000.000 68.07

42243000.000 47.94919 409430000.000 68.08

42250000.000 47.95077 409500000.000 68.08

42257000.000 47.95236 409570000.000 68.08

42264000.000 47.95395 409640000.000 68.08

42271000.000 47.95554 409710000.000 68.09

42278000.000 47.95713 409780000.000 68.09

42285000.000 47.95872 409850000.000 68.09

42292000.000 47.96030 409920000.000 68.09

42299000.000 47.96189 409990000.000 68.09

42306000.000 47.96348 410060000.000 68.10

42313000.000 47.96507 410130000.000 68.10

42320000.000 47.96665 410200000.000 68.10

42327000.000 47.96824 410270000.000 68.10

42334000.000 47.96983 410340000.000 68.11

42341000.000 47.97141 410410000.000 68.11

42348000.000 47.97300 410480000.000 68.11

42355000.000 47.97458 410550000.000 68.11

42362000.000 47.97617 410620000.000 68.12

42369000.000 47.97776 410690000.000 68.12

42376000.000 47.97934 410760000.000 68.12

42383000.000 47.98093 410830000.000 68.12

42390000.000 47.98251 410900000.000 68.12

42397000.000 47.98410 410970000.000 68.13

42404000.000 47.98568 411040000.000 68.13

42411000.000 47.98726 411110000.000 68.13

42418000.000 47.98885 411180000.000 68.13

42425000.000 47.99043 411250000.000 68.14

42432000.000 47.99202 411320000.000 68.14

42439000.000 47.99360 411390000.000 68.14

42446000.000 47.99518 411460000.000 68.14

42453000.000 47.99676 411530000.000 68.15

42460000.000 47.99835 411600000.000 68.15

42467000.000 47.99993 411670000.000 68.15

42474000.000 48.00151 411740000.000 68.15

42481000.000 48.00309 411810000.000 68.15

42488000.000 48.00468 411880000.000 68.16

42495000.000 48.00626 411950000.000 68.16

42502000.000 48.00784 412020000.000 68.16

42509000.000 48.00942 412090000.000 68.16

42516000.000 48.01100 412160000.000 68.17

42523000.000 48.01258 412230000.000 68.17

42530000.000 48.01416 412300000.000 68.17

42537000.000 48.01574 412370000.000 68.17

42544000.000 48.01732 412440000.000 68.18

42551000.000 48.01890 412510000.000 68.18

42558000.000 48.02048 412580000.000 68.18

42565000.000 48.02206 412650000.000 68.18

42572000.000 48.02364 412720000.000 68.19

42579000.000 48.02522 412790000.000 68.19

42586000.000 48.02680 412860000.000 68.19

42593000.000 48.02838 412930000.000 68.19

42600000.000 48.02996 413000000.000 68.19

42607000.000 48.03154 413070000.000 68.20

42614000.000 48.03311 413140000.000 68.20

42621000.000 48.03469 413210000.000 68.20

42628000.000 48.03627 413280000.000 68.20

42635000.000 48.03785 413350000.000 68.21

42642000.000 48.03942 413420000.000 68.21

42649000.000 48.04100 413490000.000 68.21

42656000.000 48.04258 413560000.000 68.21

42663000.000 48.04416 413630000.000 68.22

42670000.000 48.04573 413700000.000 68.22

42677000.000 48.04731 413770000.000 68.22

42684000.000 48.04888 413840000.000 68.22

42691000.000 48.05046 413910000.000 68.22

42698000.000 48.05204 413980000.000 68.23

42705000.000 48.05361 414050000.000 68.23

42712000.000 48.05519 414120000.000 68.23

42719000.000 48.05676 414190000.000 68.23

42726000.000 48.05834 414260000.000 68.24

42733000.000 48.05991 414330000.000 68.24

42740000.000 48.06149 414400000.000 68.24

42747000.000 48.06306 414470000.000 68.24

42754000.000 48.06463 414540000.000 68.25

42761000.000 48.06621 414610000.000 68.25

42768000.000 48.06778 414680000.000 68.25

42775000.000 48.06935 414750000.000 68.25

42782000.000 48.07093 414820000.000 68.25

42789000.000 48.07250 414890000.000 68.26

42796000.000 48.07407 414960000.000 68.26

42803000.000 48.07565 415030000.000 68.26

42810000.000 48.07722 415100000.000 68.26

42817000.000 48.07879 415170000.000 68.27

42824000.000 48.08036 415240000.000 68.27

42831000.000 48.08193 415310000.000 68.27

42838000.000 48.08351 415380000.000 68.27

42845000.000 48.08508 415450000.000 68.28

42852000.000 48.08665 415520000.000 68.28

42859000.000 48.08822 415590000.000 68.28

42866000.000 48.08979 415660000.000 68.28

42873000.000 48.09136 415730000.000 68.28

42880000.000 48.09293 415800000.000 68.29

42887000.000 48.09450 415870000.000 68.29

42894000.000 48.09607 415940000.000 68.29

42901000.000 48.09764 416010000.000 68.29

42908000.000 48.09921 416080000.000 68.30

42915000.000 48.10078 416150000.000 68.30

42922000.000 48.10235 416220000.000 68.30

42929000.000 48.10392 416290000.000 68.30

42936000.000 48.10549 416360000.000 68.30

42943000.000 48.10705 416430000.000 68.31

42950000.000 48.10862 416500000.000 68.31

42957000.000 48.11019 416570000.000 68.31

42964000.000 48.11176 416640000.000 68.31

42971000.000 48.11333 416710000.000 68.32

42978000.000 48.11489 416780000.000 68.32

42985000.000 48.11646 416850000.000 68.32

42992000.000 48.11803 416920000.000 68.32

42999000.000 48.11959 416990000.000 68.33

43006000.000 48.12116 417060000.000 68.33

43013000.000 48.12273 417130000.000 68.33

43020000.000 48.12429 417200000.000 68.33

43027000.000 48.12586 417270000.000 68.33

43034000.000 48.12743 417340000.000 68.34

43041000.000 48.12899 417410000.000 68.34

43048000.000 48.13056 417480000.000 68.34

43055000.000 48.13212 417550000.000 68.34

43062000.000 48.13369 417620000.000 68.35

43069000.000 48.13525 417690000.000 68.35

43076000.000 48.13682 417760000.000 68.35

43083000.000 48.13838 417830000.000 68.35

43090000.000 48.13994 417900000.000 68.36

43097000.000 48.14151 417970000.000 68.36

43104000.000 48.14307 418040000.000 68.36

43111000.000 48.14464 418110000.000 68.36

43118000.000 48.14620 418180000.000 68.36

43125000.000 48.14776 418250000.000 68.37

43132000.000 48.14933 418320000.000 68.37

43139000.000 48.15089 418390000.000 68.37

43146000.000 48.15245 418460000.000 68.37

43153000.000 48.15401 418530000.000 68.38

43160000.000 48.15557 418600000.000 68.38

43167000.000 48.15714 418670000.000 68.38

43174000.000 48.15870 418740000.000 68.38

43181000.000 48.16026 418810000.000 68.39

43188000.000 48.16182 418880000.000 68.39

43195000.000 48.16338 418950000.000 68.39

43202000.000 48.16494 419020000.000 68.39

43209000.000 48.16650 419090000.000 68.39

43216000.000 48.16806 419160000.000 68.40

43223000.000 48.16963 419230000.000 68.40

43230000.000 48.17119 419300000.000 68.40

43237000.000 48.17275 419370000.000 68.40

43244000.000 48.17430 419440000.000 68.41

43251000.000 48.17586 419510000.000 68.41

43258000.000 48.17742 419580000.000 68.41

43265000.000 48.17898 419650000.000 68.41

43272000.000 48.18054 419720000.000 68.41

43279000.000 48.18210 419790000.000 68.42

43286000.000 48.18366 419860000.000 68.42

43293000.000 48.18522 419930000.000 68.42

43300000.000 48.18678 420000000.000 68.42

43307000.000 48.18833 420070000.000 68.43

43314000.000 48.18989 420140000.000 68.43

43321000.000 48.19145 420210000.000 68.43

43328000.000 48.19301 420280000.000 68.43

43335000.000 48.19456 420350000.000 68.44

43342000.000 48.19612 420420000.000 68.44

43349000.000 48.19768 420490000.000 68.44

43356000.000 48.19923 420560000.000 68.44

43363000.000 48.20079 420630000.000 68.44

43370000.000 48.20235 420700000.000 68.45

43377000.000 48.20390 420770000.000 68.45

43384000.000 48.20546 420840000.000 68.45

43391000.000 48.20701 420910000.000 68.45

43398000.000 48.20857 420980000.000 68.46

43405000.000 48.21012 421050000.000 68.46

43412000.000 48.21168 421120000.000 68.46

43419000.000 48.21323 421190000.000 68.46

43426000.000 48.21479 421260000.000 68.46

43433000.000 48.21634 421330000.000 68.47

43440000.000 48.21790 421400000.000 68.47

43447000.000 48.21945 421470000.000 68.47

43454000.000 48.22100 421540000.000 68.47

43461000.000 48.22256 421610000.000 68.48

43468000.000 48.22411 421680000.000 68.48

43475000.000 48.22566 421750000.000 68.48

43482000.000 48.22722 421820000.000 68.48

43489000.000 48.22877 421890000.000 68.49

43496000.000 48.23032 421960000.000 68.49

43503000.000 48.23187 422030000.000 68.49

43510000.000 48.23342 422100000.000 68.49

43517000.000 48.23498 422170000.000 68.49

43524000.000 48.23653 422240000.000 68.50

43531000.000 48.23808 422310000.000 68.50

43538000.000 48.23963 422380000.000 68.50

43545000.000 48.24118 422450000.000 68.50

43552000.000 48.24273 422520000.000 68.51

43559000.000 48.24428 422590000.000 68.51

43566000.000 48.24583 422660000.000 68.51

43573000.000 48.24738 422730000.000 68.51

43580000.000 48.24893 422800000.000 68.51

43587000.000 48.25048 422870000.000 68.52

43594000.000 48.25203 422940000.000 68.52

43601000.000 48.25358 423010000.000 68.52

43608000.000 48.25513 423080000.000 68.52

43615000.000 48.25668 423150000.000 68.53

43622000.000 48.25823 423220000.000 68.53

43629000.000 48.25978 423290000.000 68.53

43636000.000 48.26133 423360000.000 68.53

43643000.000 48.26288 423430000.000 68.54

43650000.000 48.26442 423500000.000 68.54

43657000.000 48.26597 423570000.000 68.54

43664000.000 48.26752 423640000.000 68.54

43671000.000 48.26907 423710000.000 68.54

43678000.000 48.27062 423780000.000 68.55

43685000.000 48.27216 423850000.000 68.55

43692000.000 48.27371 423920000.000 68.55

43699000.000 48.27526 423990000.000 68.55

43706000.000 48.27680 424060000.000 68.56

43713000.000 48.27835 424130000.000 68.56

43720000.000 48.27989 424200000.000 68.56

43727000.000 48.28144 424270000.000 68.56

43734000.000 48.28299 424340000.000 68.56

43741000.000 48.28453 424410000.000 68.57

43748000.000 48.28608 424480000.000 68.57

43755000.000 48.28762 424550000.000 68.57

43762000.000 48.28917 424620000.000 68.57

43769000.000 48.29071 424690000.000 68.58

43776000.000 48.29226 424760000.000 68.58

43783000.000 48.29380 424830000.000 68.58

43790000.000 48.29535 424900000.000 68.58

43797000.000 48.29689 424970000.000 68.59

43804000.000 48.29843 425040000.000 68.59

43811000.000 48.29998 425110000.000 68.59

43818000.000 48.30152 425180000.000 68.59

43825000.000 48.30306 425250000.000 68.59

43832000.000 48.30461 425320000.000 68.60

43839000.000 48.30615 425390000.000 68.60

43846000.000 48.30769 425460000.000 68.60

43853000.000 48.30923 425530000.000 68.60

43860000.000 48.31078 425600000.000 68.61

43867000.000 48.31232 425670000.000 68.61

43874000.000 48.31386 425740000.000 68.61

43881000.000 48.31540 425810000.000 68.61

43888000.000 48.31694 425880000.000 68.61

43895000.000 48.31848 425950000.000 68.62

43902000.000 48.32002 426020000.000 68.62

43909000.000 48.32157 426090000.000 68.62

43916000.000 48.32311 426160000.000 68.62

43923000.000 48.32465 426230000.000 68.63

43930000.000 48.32619 426300000.000 68.63

43937000.000 48.32773 426370000.000 68.63

43944000.000 48.32927 426440000.000 68.63

43951000.000 48.33081 426510000.000 68.63

43958000.000 48.33235 426580000.000 68.64

43965000.000 48.33388 426650000.000 68.64

43972000.000 48.33542 426720000.000 68.64

43979000.000 48.33696 426790000.000 68.64

43986000.000 48.33850 426860000.000 68.65

43993000.000 48.34004 426930000.000 68.65

44000000.000 48.34158 427000000.000 68.65

44007000.000 48.34312 427070000.000 68.65

44014000.000 48.34465 427140000.000 68.65

44021000.000 48.34619 427210000.000 68.66

44028000.000 48.34773 427280000.000 68.66

44035000.000 48.34927 427350000.000 68.66

44042000.000 48.35080 427420000.000 68.66

44049000.000 48.35234 427490000.000 68.67

44056000.000 48.35388 427560000.000 68.67

44063000.000 48.35541 427630000.000 68.67

44070000.000 48.35695 427700000.000 68.67

44077000.000 48.35849 427770000.000 68.68

44084000.000 48.36002 427840000.000 68.68

44091000.000 48.36156 427910000.000 68.68

44098000.000 48.36309 427980000.000 68.68

44105000.000 48.36463 428050000.000 68.68

44112000.000 48.36616 428120000.000 68.69

44119000.000 48.36770 428190000.000 68.69

44126000.000 48.36923 428260000.000 68.69

44133000.000 48.37077 428330000.000 68.69

44140000.000 48.37230 428400000.000 68.70

44147000.000 48.37384 428470000.000 68.70

44154000.000 48.37537 428540000.000 68.70

44161000.000 48.37690 428610000.000 68.70

44168000.000 48.37844 428680000.000 68.70

44175000.000 48.37997 428750000.000 68.71

44182000.000 48.38150 428820000.000 68.71

44189000.000 48.38304 428890000.000 68.71

44196000.000 48.38457 428960000.000 68.71

44203000.000 48.38610 429030000.000 68.72

44210000.000 48.38763 429100000.000 68.72

44217000.000 48.38917 429170000.000 68.72

44224000.000 48.39070 429240000.000 68.72

44231000.000 48.39223 429310000.000 68.72

44238000.000 48.39376 429380000.000 68.73

44245000.000 48.39529 429450000.000 68.73

44252000.000 48.39682 429520000.000 68.73

44259000.000 48.39836 429590000.000 68.73

44266000.000 48.39989 429660000.000 68.74

44273000.000 48.40142 429730000.000 68.74

44280000.000 48.40295 429800000.000 68.74

44287000.000 48.40448 429870000.000 68.74

44294000.000 48.40601 429940000.000 68.74

44301000.000 48.40754 430010000.000 68.75

44308000.000 48.40907 430080000.000 68.75

44315000.000 48.41060 430150000.000 68.75

44322000.000 48.41213 430220000.000 68.75

44329000.000 48.41366 430290000.000 68.76

44336000.000 48.41518 430360000.000 68.76

44343000.000 48.41671 430430000.000 68.76

44350000.000 48.41824 430500000.000 68.76

44357000.000 48.41977 430570000.000 68.76

44364000.000 48.42130 430640000.000 68.77

44371000.000 48.42283 430710000.000 68.77

44378000.000 48.42435 430780000.000 68.77

44385000.000 48.42588 430850000.000 68.77

44392000.000 48.42741 430920000.000 68.78

44399000.000 48.42894 430990000.000 68.78

44406000.000 48.43046 431060000.000 68.78

44413000.000 48.43199 431130000.000 68.78

44420000.000 48.43352 431200000.000 68.79

44427000.000 48.43504 431270000.000 68.79

44434000.000 48.43657 431340000.000 68.79

44441000.000 48.43809 431410000.000 68.79

44448000.000 48.43962 431480000.000 68.79

44455000.000 48.44115 431550000.000 68.80

44462000.000 48.44267 431620000.000 68.80

44469000.000 48.44420 431690000.000 68.80

44476000.000 48.44572 431760000.000 68.80

44483000.000 48.44725 431830000.000 68.81

44490000.000 48.44877 431900000.000 68.81

44497000.000 48.45030 431970000.000 68.81

44504000.000 48.45182 432040000.000 68.81

44511000.000 48.45334 432110000.000 68.81

44518000.000 48.45487 432180000.000 68.82

44525000.000 48.45639 432250000.000 68.82

44532000.000 48.45792 432320000.000 68.82

44539000.000 48.45944 432390000.000 68.82

44546000.000 48.46096 432460000.000 68.83

44553000.000 48.46248 432530000.000 68.83

44560000.000 48.46401 432600000.000 68.83

44567000.000 48.46553 432670000.000 68.83

44574000.000 48.46705 432740000.000 68.83

44581000.000 48.46857 432810000.000 68.84

44588000.000 48.47010 432880000.000 68.84

44595000.000 48.47162 432950000.000 68.84

44602000.000 48.47314 433020000.000 68.84

44609000.000 48.47466 433090000.000 68.85

44616000.000 48.47618 433160000.000 68.85

44623000.000 48.47770 433230000.000 68.85

44630000.000 48.47922 433300000.000 68.85

44637000.000 48.48074 433370000.000 68.85

44644000.000 48.48227 433440000.000 68.86

44651000.000 48.48379 433510000.000 68.86

44658000.000 48.48531 433580000.000 68.86

44665000.000 48.48683 433650000.000 68.86

44672000.000 48.48835 433720000.000 68.87

44679000.000 48.48986 433790000.000 68.87

44686000.000 48.49138 433860000.000 68.87

44693000.000 48.49290 433930000.000 68.87

44700000.000 48.49442 434000000.000 68.87

44707000.000 48.49594 434070000.000 68.88

44714000.000 48.49746 434140000.000 68.88

44721000.000 48.49898 434210000.000 68.88

44728000.000 48.50050 434280000.000 68.88

44735000.000 48.50201 434350000.000 68.89

44742000.000 48.50353 434420000.000 68.89

44749000.000 48.50505 434490000.000 68.89

44756000.000 48.50657 434560000.000 68.89

44763000.000 48.50808 434630000.000 68.89

44770000.000 48.50960 434700000.000 68.90

44777000.000 48.51112 434770000.000 68.90

44784000.000 48.51263 434840000.000 68.90

44791000.000 48.51415 434910000.000 68.90

44798000.000 48.51567 434980000.000 68.91

44805000.000 48.51718 435050000.000 68.91

44812000.000 48.51870 435120000.000 68.91

44819000.000 48.52022 435190000.000 68.91

44826000.000 48.52173 435260000.000 68.91

44833000.000 48.52325 435330000.000 68.92

44840000.000 48.52476 435400000.000 68.92

44847000.000 48.52628 435470000.000 68.92

44854000.000 48.52779 435540000.000 68.92

44861000.000 48.52931 435610000.000 68.93

44868000.000 48.53082 435680000.000 68.93

44875000.000 48.53233 435750000.000 68.93

44882000.000 48.53385 435820000.000 68.93

44889000.000 48.53536 435890000.000 68.93

44896000.000 48.53688 435960000.000 68.94

44903000.000 48.53839 436030000.000 68.94

44910000.000 48.53990 436100000.000 68.94

44917000.000 48.54142 436170000.000 68.94

44924000.000 48.54293 436240000.000 68.95

44931000.000 48.54444 436310000.000 68.95

44938000.000 48.54595 436380000.000 68.95

44945000.000 48.54747 436450000.000 68.95

44952000.000 48.54898 436520000.000 68.95

44959000.000 48.55049 436590000.000 68.96

44966000.000 48.55200 436660000.000 68.96

44973000.000 48.55351 436730000.000 68.96

44980000.000 48.55502 436800000.000 68.96

44987000.000 48.55654 436870000.000 68.96

44994000.000 48.55805 436940000.000 68.97

45001000.000 48.55956 437010000.000 68.97

45008000.000 48.56107 437080000.000 68.97

45015000.000 48.56258 437150000.000 68.97

45022000.000 48.56409 437220000.000 68.98

45029000.000 48.56560 437290000.000 68.98

45036000.000 48.56711 437360000.000 68.98

45043000.000 48.56862 437430000.000 68.98

45050000.000 48.57013 437500000.000 68.98

45057000.000 48.57164 437570000.000 68.99

45064000.000 48.57315 437640000.000 68.99

45071000.000 48.57465 437710000.000 68.99

45078000.000 48.57616 437780000.000 68.99

45085000.000 48.57767 437850000.000 69.00

45092000.000 48.57918 437920000.000 69.00

45099000.000 48.58069 437990000.000 69.00

45106000.000 48.58220 438060000.000 69.00

45113000.000 48.58370 438130000.000 69.00

45120000.000 48.58521 438200000.000 69.01

45127000.000 48.58672 438270000.000 69.01

45134000.000 48.58823 438340000.000 69.01

45141000.000 48.58973 438410000.000 69.01

45148000.000 48.59124 438480000.000 69.02

45155000.000 48.59275 438550000.000 69.02

45162000.000 48.59425 438620000.000 69.02

45169000.000 48.59576 438690000.000 69.02

45176000.000 48.59727 438760000.000 69.02

45183000.000 48.59877 438830000.000 69.03

45190000.000 48.60028 438900000.000 69.03

45197000.000 48.60178 438970000.000 69.03

45204000.000 48.60329 439040000.000 69.03

45211000.000 48.60479 439110000.000 69.04

45218000.000 48.60630 439180000.000 69.04

45225000.000 48.60780 439250000.000 69.04

45232000.000 48.60931 439320000.000 69.04

45239000.000 48.61081 439390000.000 69.04

45246000.000 48.61232 439460000.000 69.05

45253000.000 48.61382 439530000.000 69.05

45260000.000 48.61533 439600000.000 69.05

45267000.000 48.61683 439670000.000 69.05

45274000.000 48.61833 439740000.000 69.06

45281000.000 48.61984 439810000.000 69.06

45288000.000 48.62134 439880000.000 69.06

45295000.000 48.62284 439950000.000 69.06

45302000.000 48.62434 440020000.000 69.06

45309000.000 48.62585 440090000.000 69.07

45316000.000 48.62735 440160000.000 69.07

45323000.000 48.62885 440230000.000 69.07

45330000.000 48.63035 440300000.000 69.07

45337000.000 48.63186 440370000.000 69.08

45344000.000 48.63336 440440000.000 69.08

45351000.000 48.63486 440510000.000 69.08

45358000.000 48.63636 440580000.000 69.08

45365000.000 48.63786 440650000.000 69.08

45372000.000 48.63936 440720000.000 69.09

45379000.000 48.64086 440790000.000 69.09

45386000.000 48.64236 440860000.000 69.09

45393000.000 48.64386 440930000.000 69.09

45400000.000 48.64536 441000000.000 69.09

45407000.000 48.64686 441070000.000 69.10

45414000.000 48.64836 441140000.000 69.10

45421000.000 48.64986 441210000.000 69.10

45428000.000 48.65136 441280000.000 69.10

45435000.000 48.65286 441350000.000 69.11

45442000.000 48.65436 441420000.000 69.11

45449000.000 48.65586 441490000.000 69.11

45456000.000 48.65736 441560000.000 69.11

45463000.000 48.65886 441630000.000 69.11

45470000.000 48.66036 441700000.000 69.12

45477000.000 48.66185 441770000.000 69.12

45484000.000 48.66335 441840000.000 69.12

45491000.000 48.66485 441910000.000 69.12

45498000.000 48.66635 441980000.000 69.13

45505000.000 48.66784 442050000.000 69.13

45512000.000 48.66934 442120000.000 69.13

45519000.000 48.67084 442190000.000 69.13

45526000.000 48.67234 442260000.000 69.13

45533000.000 48.67383 442330000.000 69.14

45540000.000 48.67533 442400000.000 69.14

45547000.000 48.67682 442470000.000 69.14

45554000.000 48.67832 442540000.000 69.14

45561000.000 48.67982 442610000.000 69.15

45568000.000 48.68131 442680000.000 69.15

45575000.000 48.68281 442750000.000 69.15

45582000.000 48.68430 442820000.000 69.15

45589000.000 48.68580 442890000.000 69.15

45596000.000 48.68729 442960000.000 69.16

45603000.000 48.68879 443030000.000 69.16

45610000.000 48.69028 443100000.000 69.16

45617000.000 48.69178 443170000.000 69.16

45624000.000 48.69327 443240000.000 69.16

45631000.000 48.69477 443310000.000 69.17

45638000.000 48.69626 443380000.000 69.17

45645000.000 48.69775 443450000.000 69.17

45652000.000 48.69925 443520000.000 69.17

45659000.000 48.70074 443590000.000 69.18

45666000.000 48.70223 443660000.000 69.18

45673000.000 48.70373 443730000.000 69.18

45680000.000 48.70522 443800000.000 69.18

45687000.000 48.70671 443870000.000 69.18

45694000.000 48.70820 443940000.000 69.19

45701000.000 48.70970 444010000.000 69.19

45708000.000 48.71119 444080000.000 69.19

45715000.000 48.71268 444150000.000 69.19

45722000.000 48.71417 444220000.000 69.20

45729000.000 48.71566 444290000.000 69.20

45736000.000 48.71716 444360000.000 69.20

45743000.000 48.71865 444430000.000 69.20

45750000.000 48.72014 444500000.000 69.20

45757000.000 48.72163 444570000.000 69.21

45764000.000 48.72312 444640000.000 69.21

45771000.000 48.72461 444710000.000 69.21

45778000.000 48.72610 444780000.000 69.21

45785000.000 48.72759 444850000.000 69.22

45792000.000 48.72908 444920000.000 69.22

45799000.000 48.73057 444990000.000 69.22

45806000.000 48.73206 445060000.000 69.22

45813000.000 48.73355 445130000.000 69.22

45820000.000 48.73504 445200000.000 69.23

45827000.000 48.73653 445270000.000 69.23

45834000.000 48.73801 445340000.000 69.23

45841000.000 48.73950 445410000.000 69.23

45848000.000 48.74099 445480000.000 69.23

45855000.000 48.74248 445550000.000 69.24

45862000.000 48.74397 445620000.000 69.24

45869000.000 48.74546 445690000.000 69.24

45876000.000 48.74694 445760000.000 69.24

45883000.000 48.74843 445830000.000 69.25

45890000.000 48.74992 445900000.000 69.25

45897000.000 48.75141 445970000.000 69.25

45904000.000 48.75289 446040000.000 69.25

45911000.000 48.75438 446110000.000 69.25

45918000.000 48.75587 446180000.000 69.26

45925000.000 48.75735 446250000.000 69.26

45932000.000 48.75884 446320000.000 69.26

45939000.000 48.76033 446390000.000 69.26

45946000.000 48.76181 446460000.000 69.27

45953000.000 48.76330 446530000.000 69.27

45960000.000 48.76478 446600000.000 69.27

45967000.000 48.76627 446670000.000 69.27

45974000.000 48.76775 446740000.000 69.27

45981000.000 48.76924 446810000.000 69.28

45988000.000 48.77072 446880000.000 69.28

45995000.000 48.77221 446950000.000 69.28

46002000.000 48.77369 447020000.000 69.28

46009000.000 48.77518 447090000.000 69.28

46016000.000 48.77666 447160000.000 69.29

46023000.000 48.77814 447230000.000 69.29

46030000.000 48.77963 447300000.000 69.29

46037000.000 48.78111 447370000.000 69.29

46044000.000 48.78259 447440000.000 69.30

46051000.000 48.78408 447510000.000 69.30

46058000.000 48.78556 447580000.000 69.30

46065000.000 48.78704 447650000.000 69.30

46072000.000 48.78853 447720000.000 69.30

46079000.000 48.79001 447790000.000 69.31

46086000.000 48.79149 447860000.000 69.31

46093000.000 48.79297 447930000.000 69.31

46100000.000 48.79445 448000000.000 69.31

46107000.000 48.79594 448070000.000 69.31

46114000.000 48.79742 448140000.000 69.32

46121000.000 48.79890 448210000.000 69.32

46128000.000 48.80038 448280000.000 69.32

46135000.000 48.80186 448350000.000 69.32

46142000.000 48.80334 448420000.000 69.33

46149000.000 48.80482 448490000.000 69.33

46156000.000 48.80630 448560000.000 69.33

46163000.000 48.80778 448630000.000 69.33

46170000.000 48.80926 448700000.000 69.33

46177000.000 48.81074 448770000.000 69.34

46184000.000 48.81222 448840000.000 69.34

46191000.000 48.81370 448910000.000 69.34

46198000.000 48.81518 448980000.000 69.34

46205000.000 48.81666 449050000.000 69.35

46212000.000 48.81814 449120000.000 69.35

46219000.000 48.81962 449190000.000 69.35

46226000.000 48.82110 449260000.000 69.35

46233000.000 48.82258 449330000.000 69.35

46240000.000 48.82406 449400000.000 69.36

46247000.000 48.82553 449470000.000 69.36

46254000.000 48.82701 449540000.000 69.36

46261000.000 48.82849 449610000.000 69.36

46268000.000 48.82997 449680000.000 69.36

46275000.000 48.83144 449750000.000 69.37

46282000.000 48.83292 449820000.000 69.37

46289000.000 48.83440 449890000.000 69.37

46296000.000 48.83588 449960000.000 69.37

46303000.000 48.83735 450030000.000 69.38

46310000.000 48.83883 450100000.000 69.38

46317000.000 48.84031 450170000.000 69.38

46324000.000 48.84178 450240000.000 69.38

46331000.000 48.84326 450310000.000 69.38

46338000.000 48.84473 450380000.000 69.39

46345000.000 48.84621 450450000.000 69.39

46352000.000 48.84768 450520000.000 69.39

46359000.000 48.84916 450590000.000 69.39

46366000.000 48.85063 450660000.000 69.39

46373000.000 48.85211 450730000.000 69.40

46380000.000 48.85358 450800000.000 69.40

46387000.000 48.85506 450870000.000 69.40

46394000.000 48.85653 450940000.000 69.40

46401000.000 48.85801 451010000.000 69.41

46408000.000 48.85948 451080000.000 69.41

46415000.000 48.86096 451150000.000 69.41

46422000.000 48.86243 451220000.000 69.41

46429000.000 48.86390 451290000.000 69.41

46436000.000 48.86538 451360000.000 69.42

46443000.000 48.86685 451430000.000 69.42

46450000.000 48.86832 451500000.000 69.42

46457000.000 48.86979 451570000.000 69.42

46464000.000 48.87127 451640000.000 69.43

46471000.000 48.87274 451710000.000 69.43

46478000.000 48.87421 451780000.000 69.43

46485000.000 48.87568 451850000.000 69.43

46492000.000 48.87716 451920000.000 69.43

46499000.000 48.87863 451990000.000 69.44

46506000.000 48.88010 452060000.000 69.44

46513000.000 48.88157 452130000.000 69.44

46520000.000 48.88304 452200000.000 69.44

46527000.000 48.88451 452270000.000 69.44

46534000.000 48.88598 452340000.000 69.45

46541000.000 48.88745 452410000.000 69.45

46548000.000 48.88893 452480000.000 69.45

46555000.000 48.89040 452550000.000 69.45

46562000.000 48.89187 452620000.000 69.46

46569000.000 48.89334 452690000.000 69.46

46576000.000 48.89481 452760000.000 69.46

46583000.000 48.89628 452830000.000 69.46

46590000.000 48.89774 452900000.000 69.46

46597000.000 48.89921 452970000.000 69.47

46604000.000 48.90068 453040000.000 69.47

46611000.000 48.90215 453110000.000 69.47

46618000.000 48.90362 453180000.000 69.47

46625000.000 48.90509 453250000.000 69.47

46632000.000 48.90656 453320000.000 69.48

46639000.000 48.90803 453390000.000 69.48

46646000.000 48.90949 453460000.000 69.48

46653000.000 48.91096 453530000.000 69.48

46660000.000 48.91243 453600000.000 69.49

46667000.000 48.91390 453670000.000 69.49

46674000.000 48.91536 453740000.000 69.49

46681000.000 48.91683 453810000.000 69.49

46688000.000 48.91830 453880000.000 69.49

46695000.000 48.91976 453950000.000 69.50

46702000.000 48.92123 454020000.000 69.50

46709000.000 48.92270 454090000.000 69.50

46716000.000 48.92416 454160000.000 69.50

46723000.000 48.92563 454230000.000 69.50

46730000.000 48.92710 454300000.000 69.51

46737000.000 48.92856 454370000.000 69.51

46744000.000 48.93003 454440000.000 69.51

46751000.000 48.93149 454510000.000 69.51

46758000.000 48.93296 454580000.000 69.52

46765000.000 48.93442 454650000.000 69.52

46772000.000 48.93589 454720000.000 69.52

46779000.000 48.93735 454790000.000 69.52

46786000.000 48.93882 454860000.000 69.52

46793000.000 48.94028 454930000.000 69.53

46800000.000 48.94175 455000000.000 69.53

46807000.000 48.94321 455070000.000 69.53

46814000.000 48.94467 455140000.000 69.53

46821000.000 48.94614 455210000.000 69.53

46828000.000 48.94760 455280000.000 69.54

46835000.000 48.94906 455350000.000 69.54

46842000.000 48.95053 455420000.000 69.54

46849000.000 48.95199 455490000.000 69.54

46856000.000 48.95345 455560000.000 69.55

46863000.000 48.95492 455630000.000 69.55

46870000.000 48.95638 455700000.000 69.55

46877000.000 48.95784 455770000.000 69.55

46884000.000 48.95930 455840000.000 69.55

46891000.000 48.96076 455910000.000 69.56

46898000.000 48.96223 455980000.000 69.56

46905000.000 48.96369 456050000.000 69.56

46912000.000 48.96515 456120000.000 69.56

46919000.000 48.96661 456190000.000 69.56

46926000.000 48.96807 456260000.000 69.57

46933000.000 48.96953 456330000.000 69.57

46940000.000 48.97099 456400000.000 69.57

46947000.000 48.97245 456470000.000 69.57

46954000.000 48.97391 456540000.000 69.58

46961000.000 48.97537 456610000.000 69.58

46968000.000 48.97683 456680000.000 69.58

46975000.000 48.97829 456750000.000 69.58

46982000.000 48.97975 456820000.000 69.58

46989000.000 48.98121 456890000.000 69.59

46996000.000 48.98267 456960000.000 69.59

47003000.000 48.98413 457030000.000 69.59

47010000.000 48.98559 457100000.000 69.59

47017000.000 48.98705 457170000.000 69.59

47024000.000 48.98851 457240000.000 69.60

47031000.000 48.98996 457310000.000 69.60

47038000.000 48.99142 457380000.000 69.60

47045000.000 48.99288 457450000.000 69.60

47052000.000 48.99434 457520000.000 69.60

47059000.000 48.99580 457590000.000 69.61

47066000.000 48.99725 457660000.000 69.61

47073000.000 48.99871 457730000.000 69.61

47080000.000 49.00017 457800000.000 69.61

47087000.000 49.00163 457870000.000 69.62

47094000.000 49.00308 457940000.000 69.62

47101000.000 49.00454 458010000.000 69.62

47108000.000 49.00600 458080000.000 69.62

47115000.000 49.00745 458150000.000 69.62

47122000.000 49.00891 458220000.000 69.63

47129000.000 49.01036 458290000.000 69.63

47136000.000 49.01182 458360000.000 69.63

47143000.000 49.01328 458430000.000 69.63

47150000.000 49.01473 458500000.000 69.63

47157000.000 49.01619 458570000.000 69.64

47164000.000 49.01764 458640000.000 69.64

47171000.000 49.01910 458710000.000 69.64

47178000.000 49.02055 458780000.000 69.64

47185000.000 49.02201 458850000.000 69.65

47192000.000 49.02346 458920000.000 69.65

47199000.000 49.02491 458990000.000 69.65

47206000.000 49.02637 459060000.000 69.65

47213000.000 49.02782 459130000.000 69.65

47220000.000 49.02928 459200000.000 69.66

47227000.000 49.03073 459270000.000 69.66

47234000.000 49.03218 459340000.000 69.66

47241000.000 49.03364 459410000.000 69.66

47248000.000 49.03509 459480000.000 69.66

47255000.000 49.03654 459550000.000 69.67

47262000.000 49.03799 459620000.000 69.67

47269000.000 49.03945 459690000.000 69.67

47276000.000 49.04090 459760000.000 69.67

47283000.000 49.04235 459830000.000 69.68

47290000.000 49.04380 459900000.000 69.68

47297000.000 49.04526 459970000.000 69.68

47304000.000 49.04671 460040000.000 69.68

47311000.000 49.04816 460110000.000 69.68

47318000.000 49.04961 460180000.000 69.69

47325000.000 49.05106 460250000.000 69.69

47332000.000 49.05251 460320000.000 69.69

47339000.000 49.05396 460390000.000 69.69

47346000.000 49.05541 460460000.000 69.69

47353000.000 49.05686 460530000.000 69.70

47360000.000 49.05831 460600000.000 69.70

47367000.000 49.05976 460670000.000 69.70

47374000.000 49.06121 460740000.000 69.70

47381000.000 49.06266 460810000.000 69.70

47388000.000 49.06411 460880000.000 69.71

47395000.000 49.06556 460950000.000 69.71

47402000.000 49.06701 461020000.000 69.71

47409000.000 49.06846 461090000.000 69.71

47416000.000 49.06991 461160000.000 69.72

47423000.000 49.07136 461230000.000 69.72

47430000.000 49.07281 461300000.000 69.72

47437000.000 49.07426 461370000.000 69.72

47444000.000 49.07570 461440000.000 69.72

47451000.000 49.07715 461510000.000 69.73

47458000.000 49.07860 461580000.000 69.73

47465000.000 49.08005 461650000.000 69.73

47472000.000 49.08150 461720000.000 69.73

47479000.000 49.08294 461790000.000 69.73

47486000.000 49.08439 461860000.000 69.74

47493000.000 49.08584 461930000.000 69.74

47500000.000 49.08728 462000000.000 69.74

47507000.000 49.08873 462070000.000 69.74

47514000.000 49.09018 462140000.000 69.74

47521000.000 49.09162 462210000.000 69.75

47528000.000 49.09307 462280000.000 69.75

47535000.000 49.09452 462350000.000 69.75

47542000.000 49.09596 462420000.000 69.75

47549000.000 49.09741 462490000.000 69.76

47556000.000 49.09885 462560000.000 69.76

47563000.000 49.10030 462630000.000 69.76

47570000.000 49.10174 462700000.000 69.76

47577000.000 49.10319 462770000.000 69.76

47584000.000 49.10463 462840000.000 69.77

47591000.000 49.10608 462910000.000 69.77

47598000.000 49.10752 462980000.000 69.77

47605000.000 49.10897 463050000.000 69.77

47612000.000 49.11041 463120000.000 69.77

47619000.000 49.11186 463190000.000 69.78

47626000.000 49.11330 463260000.000 69.78

47633000.000 49.11474 463330000.000 69.78

47640000.000 49.11619 463400000.000 69.78

47647000.000 49.11763 463470000.000 69.79

47654000.000 49.11907 463540000.000 69.79

47661000.000 49.12052 463610000.000 69.79

47668000.000 49.12196 463680000.000 69.79

47675000.000 49.12340 463750000.000 69.79

47682000.000 49.12484 463820000.000 69.80

47689000.000 49.12629 463890000.000 69.80

47696000.000 49.12773 463960000.000 69.80

47703000.000 49.12917 464030000.000 69.80

47710000.000 49.13061 464100000.000 69.80

47717000.000 49.13205 464170000.000 69.81

47724000.000 49.13349 464240000.000 69.81

47731000.000 49.13494 464310000.000 69.81

47738000.000 49.13638 464380000.000 69.81

47745000.000 49.13782 464450000.000 69.81

47752000.000 49.13926 464520000.000 69.82

47759000.000 49.14070 464590000.000 69.82

47766000.000 49.14214 464660000.000 69.82

47773000.000 49.14358 464730000.000 69.82

47780000.000 49.14502 464800000.000 69.83

47787000.000 49.14646 464870000.000 69.83

47794000.000 49.14790 464940000.000 69.83

47801000.000 49.14934 465010000.000 69.83

47808000.000 49.15078 465080000.000 69.83

47815000.000 49.15222 465150000.000 69.84

47822000.000 49.15366 465220000.000 69.84

47829000.000 49.15510 465290000.000 69.84

47836000.000 49.15653 465360000.000 69.84

47843000.000 49.15797 465430000.000 69.84

47850000.000 49.15941 465500000.000 69.85

47857000.000 49.16085 465570000.000 69.85

47864000.000 49.16229 465640000.000 69.85

47871000.000 49.16373 465710000.000 69.85

47878000.000 49.16516 465780000.000 69.85

47885000.000 49.16660 465850000.000 69.86

47892000.000 49.16804 465920000.000 69.86

47899000.000 49.16948 465990000.000 69.86

47906000.000 49.17091 466060000.000 69.86

47913000.000 49.17235 466130000.000 69.86

47920000.000 49.17379 466200000.000 69.87

47927000.000 49.17522 466270000.000 69.87

47934000.000 49.17666 466340000.000 69.87

47941000.000 49.17810 466410000.000 69.87

47948000.000 49.17953 466480000.000 69.88

47955000.000 49.18097 466550000.000 69.88

47962000.000 49.18240 466620000.000 69.88

47969000.000 49.18384 466690000.000 69.88

47976000.000 49.18527 466760000.000 69.88

47983000.000 49.18671 466830000.000 69.89

47990000.000 49.18814 466900000.000 69.89

47997000.000 49.18958 466970000.000 69.89

48004000.000 49.19101 467040000.000 69.89

48011000.000 49.19245 467110000.000 69.89

48018000.000 49.19388 467180000.000 69.90

48025000.000 49.19532 467250000.000 69.90

48032000.000 49.19675 467320000.000 69.90

48039000.000 49.19818 467390000.000 69.90

48046000.000 49.19962 467460000.000 69.90

48053000.000 49.20105 467530000.000 69.91

48060000.000 49.20249 467600000.000 69.91

48067000.000 49.20392 467670000.000 69.91

48074000.000 49.20535 467740000.000 69.91

48081000.000 49.20678 467810000.000 69.92

48088000.000 49.20822 467880000.000 69.92

48095000.000 49.20965 467950000.000 69.92

48102000.000 49.21108 468020000.000 69.92

48109000.000 49.21251 468090000.000 69.92

48116000.000 49.21395 468160000.000 69.93

48123000.000 49.21538 468230000.000 69.93

48130000.000 49.21681 468300000.000 69.93

48137000.000 49.21824 468370000.000 69.93

48144000.000 49.21967 468440000.000 69.93

48151000.000 49.22110 468510000.000 69.94

48158000.000 49.22253 468580000.000 69.94

48165000.000 49.22397 468650000.000 69.94

48172000.000 49.22540 468720000.000 69.94

48179000.000 49.22683 468790000.000 69.94

48186000.000 49.22826 468860000.000 69.95

48193000.000 49.22969 468930000.000 69.95

48200000.000 49.23112 469000000.000 69.95

48207000.000 49.23255 469070000.000 69.95

48214000.000 49.23398 469140000.000 69.95

48221000.000 49.23541 469210000.000 69.96

48228000.000 49.23684 469280000.000 69.96

48235000.000 49.23826 469350000.000 69.96

48242000.000 49.23969 469420000.000 69.96

48249000.000 49.24112 469490000.000 69.97

48256000.000 49.24255 469560000.000 69.97

48263000.000 49.24398 469630000.000 69.97

48270000.000 49.24541 469700000.000 69.97

48277000.000 49.24684 469770000.000 69.97

48284000.000 49.24826 469840000.000 69.98

48291000.000 49.24969 469910000.000 69.98

48298000.000 49.25112 469980000.000 69.98

48305000.000 49.25255 470050000.000 69.98

48312000.000 49.25398 470120000.000 69.98

48319000.000 49.25540 470190000.000 69.99

48326000.000 49.25683 470260000.000 69.99

48333000.000 49.25826 470330000.000 69.99

48340000.000 49.25968 470400000.000 69.99

48347000.000 49.26111 470470000.000 69.99

48354000.000 49.26254 470540000.000 70.00

48361000.000 49.26396 470610000.000 70.00

48368000.000 49.26539 470680000.000 70.00

48375000.000 49.26681 470750000.000 70.00

48382000.000 49.26824 470820000.000 70.00

48389000.000 49.26967 470890000.000 70.01

48396000.000 49.27109 470960000.000 70.01

48403000.000 49.27252 471030000.000 70.01

48410000.000 49.27394 471100000.000 70.01

48417000.000 49.27537 471170000.000 70.02

48424000.000 49.27679 471240000.000 70.02

48431000.000 49.27822 471310000.000 70.02

48438000.000 49.27964 471380000.000 70.02

48445000.000 49.28106 471450000.000 70.02

48452000.000 49.28249 471520000.000 70.03

48459000.000 49.28391 471590000.000 70.03

48466000.000 49.28534 471660000.000 70.03

48473000.000 49.28676 471730000.000 70.03

48480000.000 49.28818 471800000.000 70.03

48487000.000 49.28961 471870000.000 70.04

48494000.000 49.29103 471940000.000 70.04

48501000.000 49.29245 472010000.000 70.04

48508000.000 49.29387 472080000.000 70.04

48515000.000 49.29530 472150000.000 70.04

48522000.000 49.29672 472220000.000 70.05

48529000.000 49.29814 472290000.000 70.05

48536000.000 49.29956 472360000.000 70.05

48543000.000 49.30099 472430000.000 70.05

48550000.000 49.30241 472500000.000 70.05

48557000.000 49.30383 472570000.000 70.06

48564000.000 49.30525 472640000.000 70.06

48571000.000 49.30667 472710000.000 70.06

48578000.000 49.30809 472780000.000 70.06

48585000.000 49.30951 472850000.000 70.07

48592000.000 49.31094 472920000.000 70.07

48599000.000 49.31236 472990000.000 70.07

48606000.000 49.31378 473060000.000 70.07

48613000.000 49.31520 473130000.000 70.07

48620000.000 49.31662 473200000.000 70.08

48627000.000 49.31804 473270000.000 70.08

48634000.000 49.31946 473340000.000 70.08

48641000.000 49.32088 473410000.000 70.08

48648000.000 49.32230 473480000.000 70.08

48655000.000 49.32372 473550000.000 70.09

48662000.000 49.32513 473620000.000 70.09

48669000.000 49.32655 473690000.000 70.09

48676000.000 49.32797 473760000.000 70.09

48683000.000 49.32939 473830000.000 70.09

48690000.000 49.33081 473900000.000 70.10

48697000.000 49.33223 473970000.000 70.10

48704000.000 49.33365 474040000.000 70.10

48711000.000 49.33506 474110000.000 70.10

48718000.000 49.33648 474180000.000 70.10

48725000.000 49.33790 474250000.000 70.11

48732000.000 49.33932 474320000.000 70.11

48739000.000 49.34073 474390000.000 70.11

48746000.000 49.34215 474460000.000 70.11

48753000.000 49.34357 474530000.000 70.11

48760000.000 49.34499 474600000.000 70.12

48767000.000 49.34640 474670000.000 70.12

48774000.000 49.34782 474740000.000 70.12

48781000.000 49.34923 474810000.000 70.12

48788000.000 49.35065 474880000.000 70.13

48795000.000 49.35207 474950000.000 70.13

48802000.000 49.35348 475020000.000 70.13

48809000.000 49.35490 475090000.000 70.13

48816000.000 49.35631 475160000.000 70.13

48823000.000 49.35773 475230000.000 70.14

48830000.000 49.35915 475300000.000 70.14

48837000.000 49.36056 475370000.000 70.14

48844000.000 49.36198 475440000.000 70.14

48851000.000 49.36339 475510000.000 70.14

48858000.000 49.36480 475580000.000 70.15

48865000.000 49.36622 475650000.000 70.15

48872000.000 49.36763 475720000.000 70.15

48879000.000 49.36905 475790000.000 70.15

48886000.000 49.37046 475860000.000 70.15

48893000.000 49.37188 475930000.000 70.16

48900000.000 49.37329 476000000.000 70.16

48907000.000 49.37470 476070000.000 70.16

48914000.000 49.37612 476140000.000 70.16

48921000.000 49.37753 476210000.000 70.16

48928000.000 49.37894 476280000.000 70.17

48935000.000 49.38035 476350000.000 70.17

48942000.000 49.38177 476420000.000 70.17

48949000.000 49.38318 476490000.000 70.17

48956000.000 49.38459 476560000.000 70.17

48963000.000 49.38600 476630000.000 70.18

48970000.000 49.38742 476700000.000 70.18

48977000.000 49.38883 476770000.000 70.18

48984000.000 49.39024 476840000.000 70.18

48991000.000 49.39165 476910000.000 70.19

48998000.000 49.39306 476980000.000 70.19

49005000.000 49.39447 477050000.000 70.19

49012000.000 49.39588 477120000.000 70.19

49019000.000 49.39730 477190000.000 70.19

49026000.000 49.39871 477260000.000 70.20

49033000.000 49.40012 477330000.000 70.20

49040000.000 49.40153 477400000.000 70.20

49047000.000 49.40294 477470000.000 70.20

49054000.000 49.40435 477540000.000 70.20

49061000.000 49.40576 477610000.000 70.21

49068000.000 49.40717 477680000.000 70.21

49075000.000 49.40858 477750000.000 70.21

49082000.000 49.40999 477820000.000 70.21

49089000.000 49.41140 477890000.000 70.21

49096000.000 49.41280 477960000.000 70.22

49103000.000 49.41421 478030000.000 70.22

49110000.000 49.41562 478100000.000 70.22

49117000.000 49.41703 478170000.000 70.22

49124000.000 49.41844 478240000.000 70.22

49131000.000 49.41985 478310000.000 70.23

49138000.000 49.42126 478380000.000 70.23

49145000.000 49.42266 478450000.000 70.23

49152000.000 49.42407 478520000.000 70.23

49159000.000 49.42548 478590000.000 70.23

49166000.000 49.42689 478660000.000 70.24

49173000.000 49.42829 478730000.000 70.24

49180000.000 49.42970 478800000.000 70.24

49187000.000 49.43111 478870000.000 70.24

49194000.000 49.43252 478940000.000 70.24

49201000.000 49.43392 479010000.000 70.25

49208000.000 49.43533 479080000.000 70.25

49215000.000 49.43674 479150000.000 70.25

49222000.000 49.43814 479220000.000 70.25

49229000.000 49.43955 479290000.000 70.26

49236000.000 49.44095 479360000.000 70.26

49243000.000 49.44236 479430000.000 70.26

49250000.000 49.44376 479500000.000 70.26

49257000.000 49.44517 479570000.000 70.26

49264000.000 49.44658 479640000.000 70.27

49271000.000 49.44798 479710000.000 70.27

49278000.000 49.44939 479780000.000 70.27

49285000.000 49.45079 479850000.000 70.27

49292000.000 49.45219 479920000.000 70.27

49299000.000 49.45360 479990000.000 70.28

49306000.000 49.45500 480060000.000 70.28

49313000.000 49.45641 480130000.000 70.28

49320000.000 49.45781 480200000.000 70.28

49327000.000 49.45922 480270000.000 70.28

49334000.000 49.46062 480340000.000 70.29

49341000.000 49.46202 480410000.000 70.29

49348000.000 49.46343 480480000.000 70.29

49355000.000 49.46483 480550000.000 70.29

49362000.000 49.46623 480620000.000 70.29

49369000.000 49.46764 480690000.000 70.30

49376000.000 49.46904 480760000.000 70.30

49383000.000 49.47044 480830000.000 70.30

49390000.000 49.47184 480900000.000 70.30

49397000.000 49.47325 480970000.000 70.30

49404000.000 49.47465 481040000.000 70.31

49411000.000 49.47605 481110000.000 70.31

49418000.000 49.47745 481180000.000 70.31

49425000.000 49.47885 481250000.000 70.31

49432000.000 49.48025 481320000.000 70.31

49439000.000 49.48166 481390000.000 70.32

49446000.000 49.48306 481460000.000 70.32

49453000.000 49.48446 481530000.000 70.32

49460000.000 49.48586 481600000.000 70.32

49467000.000 49.48726 481670000.000 70.32

49474000.000 49.48866 481740000.000 70.33

49481000.000 49.49006 481810000.000 70.33

49488000.000 49.49146 481880000.000 70.33

49495000.000 49.49286 481950000.000 70.33

49502000.000 49.49426 482020000.000 70.33

49509000.000 49.49566 482090000.000 70.34

49516000.000 49.49706 482160000.000 70.34

49523000.000 49.49846 482230000.000 70.34

49530000.000 49.49986 482300000.000 70.34

49537000.000 49.50126 482370000.000 70.35

49544000.000 49.50266 482440000.000 70.35

49551000.000 49.50405 482510000.000 70.35

49558000.000 49.50545 482580000.000 70.35

49565000.000 49.50685 482650000.000 70.35

49572000.000 49.50825 482720000.000 70.36

49579000.000 49.50965 482790000.000 70.36

49586000.000 49.51105 482860000.000 70.36

49593000.000 49.51244 482930000.000 70.36

49600000.000 49.51384 483000000.000 70.36

49607000.000 49.51524 483070000.000 70.37

49614000.000 49.51664 483140000.000 70.37

49621000.000 49.51803 483210000.000 70.37

49628000.000 49.51943 483280000.000 70.37

49635000.000 49.52083 483350000.000 70.37

49642000.000 49.52222 483420000.000 70.38

49649000.000 49.52362 483490000.000 70.38

49656000.000 49.52502 483560000.000 70.38

49663000.000 49.52641 483630000.000 70.38

49670000.000 49.52781 483700000.000 70.38

49677000.000 49.52920 483770000.000 70.39

49684000.000 49.53060 483840000.000 70.39

49691000.000 49.53200 483910000.000 70.39

49698000.000 49.53339 483980000.000 70.39

49705000.000 49.53479 484050000.000 70.39

49712000.000 49.53618 484120000.000 70.40

49719000.000 49.53758 484190000.000 70.40

49726000.000 49.53897 484260000.000 70.40

49733000.000 49.54037 484330000.000 70.40

49740000.000 49.54176 484400000.000 70.40

49747000.000 49.54316 484470000.000 70.41

49754000.000 49.54455 484540000.000 70.41

49761000.000 49.54594 484610000.000 70.41

49768000.000 49.54734 484680000.000 70.41

49775000.000 49.54873 484750000.000 70.41

49782000.000 49.55012 484820000.000 70.42

49789000.000 49.55152 484890000.000 70.42

49796000.000 49.55291 484960000.000 70.42

49803000.000 49.55430 485030000.000 70.42

49810000.000 49.55570 485100000.000 70.42

49817000.000 49.55709 485170000.000 70.43

49824000.000 49.55848 485240000.000 70.43

49831000.000 49.55988 485310000.000 70.43

49838000.000 49.56127 485380000.000 70.43

49845000.000 49.56266 485450000.000 70.43

49852000.000 49.56405 485520000.000 70.44

49859000.000 49.56544 485590000.000 70.44

49866000.000 49.56684 485660000.000 70.44

49873000.000 49.56823 485730000.000 70.44

49880000.000 49.56962 485800000.000 70.44

49887000.000 49.57101 485870000.000 70.45

49894000.000 49.57240 485940000.000 70.45

49901000.000 49.57379 486010000.000 70.45

49908000.000 49.57518 486080000.000 70.45

49915000.000 49.57657 486150000.000 70.46

49922000.000 49.57796 486220000.000 70.46

49929000.000 49.57935 486290000.000 70.46

49936000.000 49.58074 486360000.000 70.46

49943000.000 49.58213 486430000.000 70.46

49950000.000 49.58352 486500000.000 70.47

49957000.000 49.58491 486570000.000 70.47

49964000.000 49.58630 486640000.000 70.47

49971000.000 49.58769 486710000.000 70.47

49978000.000 49.58908 486780000.000 70.47

49985000.000 49.59047 486850000.000 70.48

49992000.000 49.59186 486920000.000 70.48

49999000.000 49.59325 486990000.000 70.48

50006000.000 49.59464 487060000.000 70.48

50013000.000 49.59602 487130000.000 70.48

50020000.000 49.59741 487200000.000 70.49

50027000.000 49.59880 487270000.000 70.49

50034000.000 49.60019 487340000.000 70.49

50041000.000 49.60158 487410000.000 70.49

50048000.000 49.60296 487480000.000 70.49

50055000.000 49.60435 487550000.000 70.50

50062000.000 49.60574 487620000.000 70.50

50069000.000 49.60713 487690000.000 70.50

50076000.000 49.60851 487760000.000 70.50

50083000.000 49.60990 487830000.000 70.50

50090000.000 49.61129 487900000.000 70.51

50097000.000 49.61267 487970000.000 70.51

50104000.000 49.61406 488040000.000 70.51

50111000.000 49.61545 488110000.000 70.51

50118000.000 49.61683 488180000.000 70.51

50125000.000 49.61822 488250000.000 70.52

50132000.000 49.61960 488320000.000 70.52

50139000.000 49.62099 488390000.000 70.52

50146000.000 49.62237 488460000.000 70.52

50153000.000 49.62376 488530000.000 70.52

50160000.000 49.62515 488600000.000 70.53

50167000.000 49.62653 488670000.000 70.53

50174000.000 49.62791 488740000.000 70.53

50181000.000 49.62930 488810000.000 70.53

50188000.000 49.63068 488880000.000 70.53

50195000.000 49.63207 488950000.000 70.54

50202000.000 49.63345 489020000.000 70.54

50209000.000 49.63484 489090000.000 70.54

50216000.000 49.63622 489160000.000 70.54

50223000.000 49.63760 489230000.000 70.54

50230000.000 49.63899 489300000.000 70.55

50237000.000 49.64037 489370000.000 70.55

50244000.000 49.64175 489440000.000 70.55

50251000.000 49.64314 489510000.000 70.55

50258000.000 49.64452 489580000.000 70.55

50265000.000 49.64590 489650000.000 70.56

50272000.000 49.64729 489720000.000 70.56

50279000.000 49.64867 489790000.000 70.56

50286000.000 49.65005 489860000.000 70.56

50293000.000 49.65143 489930000.000 70.56

50300000.000 49.65282 490000000.000 70.57

50307000.000 49.65420 490070000.000 70.57

50314000.000 49.65558 490140000.000 70.57

50321000.000 49.65696 490210000.000 70.57

50328000.000 49.65834 490280000.000 70.57

50335000.000 49.65972 490350000.000 70.58

50342000.000 49.66110 490420000.000 70.58

50349000.000 49.66249 490490000.000 70.58

50356000.000 49.66387 490560000.000 70.58

50363000.000 49.66525 490630000.000 70.58

50370000.000 49.66663 490700000.000 70.59

50377000.000 49.66801 490770000.000 70.59

50384000.000 49.66939 490840000.000 70.59

50391000.000 49.67077 490910000.000 70.59

50398000.000 49.67215 490980000.000 70.59

50405000.000 49.67353 491050000.000 70.60

50412000.000 49.67491 491120000.000 70.60

50419000.000 49.67629 491190000.000 70.60

50426000.000 49.67767 491260000.000 70.60

50433000.000 49.67905 491330000.000 70.60

50440000.000 49.68042 491400000.000 70.61

50447000.000 49.68180 491470000.000 70.61

50454000.000 49.68318 491540000.000 70.61

50461000.000 49.68456 491610000.000 70.61

50468000.000 49.68594 491680000.000 70.61

50475000.000 49.68732 491750000.000 70.62

50482000.000 49.68870 491820000.000 70.62

50489000.000 49.69007 491890000.000 70.62

50496000.000 49.69145 491960000.000 70.62

50503000.000 49.69283 492030000.000 70.62

50510000.000 49.69421 492100000.000 70.63

50517000.000 49.69558 492170000.000 70.63

50524000.000 49.69696 492240000.000 70.63

50531000.000 49.69834 492310000.000 70.63

50538000.000 49.69971 492380000.000 70.63

50545000.000 49.70109 492450000.000 70.64

50552000.000 49.70247 492520000.000 70.64

50559000.000 49.70384 492590000.000 70.64

50566000.000 49.70522 492660000.000 70.64

50573000.000 49.70660 492730000.000 70.64

50580000.000 49.70797 492800000.000 70.65

50587000.000 49.70935 492870000.000 70.65

50594000.000 49.71072 492940000.000 70.65

50601000.000 49.71210 493010000.000 70.65

50608000.000 49.71347 493080000.000 70.65

50615000.000 49.71485 493150000.000 70.66

50622000.000 49.71622 493220000.000 70.66

50629000.000 49.71760 493290000.000 70.66

50636000.000 49.71897 493360000.000 70.66

50643000.000 49.72035 493430000.000 70.66

50650000.000 49.72172 493500000.000 70.67

50657000.000 49.72310 493570000.000 70.67

50664000.000 49.72447 493640000.000 70.67

50671000.000 49.72585 493710000.000 70.67

50678000.000 49.72722 493780000.000 70.67

50685000.000 49.72859 493850000.000 70.68

50692000.000 49.72997 493920000.000 70.68

50699000.000 49.73134 493990000.000 70.68

50706000.000 49.73271 494060000.000 70.68

50713000.000 49.73409 494130000.000 70.68

50720000.000 49.73546 494200000.000 70.69

50727000.000 49.73683 494270000.000 70.69

50734000.000 49.73820 494340000.000 70.69

50741000.000 49.73958 494410000.000 70.69

50748000.000 49.74095 494480000.000 70.69

50755000.000 49.74232 494550000.000 70.70

50762000.000 49.74369 494620000.000 70.70

50769000.000 49.74507 494690000.000 70.70

50776000.000 49.74644 494760000.000 70.70

50783000.000 49.74781 494830000.000 70.70

50790000.000 49.74918 494900000.000 70.71

50797000.000 49.75055 494970000.000 70.71

50804000.000 49.75192 495040000.000 70.71

50811000.000 49.75329 495110000.000 70.71

50818000.000 49.75466 495180000.000 70.71

50825000.000 49.75603 495250000.000 70.72

50832000.000 49.75741 495320000.000 70.72

50839000.000 49.75878 495390000.000 70.72

50846000.000 49.76015 495460000.000 70.72

50853000.000 49.76152 495530000.000 70.72

50860000.000 49.76289 495600000.000 70.73

50867000.000 49.76426 495670000.000 70.73

50874000.000 49.76562 495740000.000 70.73

50881000.000 49.76699 495810000.000 70.73

50888000.000 49.76836 495880000.000 70.73

50895000.000 49.76973 495950000.000 70.74

50902000.000 49.77110 496020000.000 70.74

50909000.000 49.77247 496090000.000 70.74

50916000.000 49.77384 496160000.000 70.74

50923000.000 49.77521 496230000.000 70.74

50930000.000 49.77658 496300000.000 70.75

50937000.000 49.77794 496370000.000 70.75

50944000.000 49.77931 496440000.000 70.75

50951000.000 49.78068 496510000.000 70.75

50958000.000 49.78205 496580000.000 70.75

50965000.000 49.78342 496650000.000 70.76

50972000.000 49.78478 496720000.000 70.76

50979000.000 49.78615 496790000.000 70.76

50986000.000 49.78752 496860000.000 70.76

50993000.000 49.78888 496930000.000 70.76

51000000.000 49.79025 497000000.000 70.77

51007000.000 49.79162 497070000.000 70.77

51014000.000 49.79298 497140000.000 70.77

51021000.000 49.79435 497210000.000 70.77

51028000.000 49.79572 497280000.000 70.77

51035000.000 49.79708 497350000.000 70.78

51042000.000 49.79845 497420000.000 70.78

51049000.000 49.79982 497490000.000 70.78

51056000.000 49.80118 497560000.000 70.78

51063000.000 49.80255 497630000.000 70.78

51070000.000 49.80391 497700000.000 70.79

51077000.000 49.80528 497770000.000 70.79

51084000.000 49.80664 497840000.000 70.79

51091000.000 49.80801 497910000.000 70.79

51098000.000 49.80937 497980000.000 70.79

51105000.000 49.81074 498050000.000 70.80

51112000.000 49.81210 498120000.000 70.80

51119000.000 49.81347 498190000.000 70.80

51126000.000 49.81483 498260000.000 70.80

51133000.000 49.81619 498330000.000 70.80

51140000.000 49.81756 498400000.000 70.81

51147000.000 49.81892 498470000.000 70.81

51154000.000 49.82028 498540000.000 70.81

51161000.000 49.82165 498610000.000 70.81

51168000.000 49.82301 498680000.000 70.81

51175000.000 49.82437 498750000.000 70.82

51182000.000 49.82574 498820000.000 70.82

51189000.000 49.82710 498890000.000 70.82

51196000.000 49.82846 498960000.000 70.82

51203000.000 49.82983 499030000.000 70.82

51210000.000 49.83119 499100000.000 70.83

51217000.000 49.83255 499170000.000 70.83

51224000.000 49.83391 499240000.000 70.83

51231000.000 49.83527 499310000.000 70.83

51238000.000 49.83664 499380000.000 70.83

51245000.000 49.83800 499450000.000 70.84

51252000.000 49.83936 499520000.000 70.84

51259000.000 49.84072 499590000.000 70.84

51266000.000 49.84208 499660000.000 70.84

51273000.000 49.84344 499730000.000 70.84

51280000.000 49.84480 499800000.000 70.85

51287000.000 49.84616 499870000.000 70.85

51294000.000 49.84752 499940000.000 70.85

51301000.000 49.84889 500010000.000 70.85

51308000.000 49.85025 500080000.000 70.85

51315000.000 49.85161 500150000.000 70.86

51322000.000 49.85297 500220000.000 70.86

51329000.000 49.85433 500290000.000 70.86

51336000.000 49.85569 500360000.000 70.86

51343000.000 49.85704 500430000.000 70.86

51350000.000 49.85840 500500000.000 70.87

51357000.000 49.85976 500570000.000 70.87

51364000.000 49.86112 500640000.000 70.87

51371000.000 49.86248 500710000.000 70.87

51378000.000 49.86384 500780000.000 70.87

51385000.000 49.86520 500850000.000 70.88

51392000.000 49.86656 500920000.000 70.88

51399000.000 49.86792 500990000.000 70.88

51406000.000 49.86927 501060000.000 70.88

51413000.000 49.87063 501130000.000 70.88

51420000.000 49.87199 501200000.000 70.89

51427000.000 49.87335 501270000.000 70.89

51434000.000 49.87471 501340000.000 70.89

51441000.000 49.87606 501410000.000 70.89

51448000.000 49.87742 501480000.000 70.89

51455000.000 49.87878 501550000.000 70.90

51462000.000 49.88013 501620000.000 70.90

51469000.000 49.88149 501690000.000 70.90

51476000.000 49.88285 501760000.000 70.90

51483000.000 49.88420 501830000.000 70.90

51490000.000 49.88556 501900000.000 70.91

51497000.000 49.88692 501970000.000 70.91

51504000.000 49.88827 502040000.000 70.91

51511000.000 49.88963 502110000.000 70.91

51518000.000 49.89099 502180000.000 70.91

51525000.000 49.89234 502250000.000 70.92

51532000.000 49.89370 502320000.000 70.92

51539000.000 49.89505 502390000.000 70.92

51546000.000 49.89641 502460000.000 70.92

51553000.000 49.89776 502530000.000 70.92

51560000.000 49.89912 502600000.000 70.93

51567000.000 49.90047 502670000.000 70.93

51574000.000 49.90183 502740000.000 70.93

51581000.000 49.90318 502810000.000 70.93

51588000.000 49.90454 502880000.000 70.93

51595000.000 49.90589 502950000.000 70.94

51602000.000 49.90724 503020000.000 70.94

51609000.000 49.90860 503090000.000 70.94

51616000.000 49.90995 503160000.000 70.94

51623000.000 49.91131 503230000.000 70.94

51630000.000 49.91266 503300000.000 70.95

51637000.000 49.91401 503370000.000 70.95

51644000.000 49.91537 503440000.000 70.95

51651000.000 49.91672 503510000.000 70.95

51658000.000 49.91807 503580000.000 70.95

51665000.000 49.91942 503650000.000 70.96

51672000.000 49.92078 503720000.000 70.96

51679000.000 49.92213 503790000.000 70.96

51686000.000 49.92348 503860000.000 70.96

51693000.000 49.92483 503930000.000 70.96

51700000.000 49.92619 504000000.000 70.96

51707000.000 49.92754 504070000.000 70.97

51714000.000 49.92889 504140000.000 70.97

51721000.000 49.93024 504210000.000 70.97

51728000.000 49.93159 504280000.000 70.97

51735000.000 49.93294 504350000.000 70.97

51742000.000 49.93430 504420000.000 70.98

51749000.000 49.93565 504490000.000 70.98

51756000.000 49.93700 504560000.000 70.98

51763000.000 49.93835 504630000.000 70.98

51770000.000 49.93970 504700000.000 70.98

51777000.000 49.94105 504770000.000 70.99

51784000.000 49.94240 504840000.000 70.99

51791000.000 49.94375 504910000.000 70.99

51798000.000 49.94510 504980000.000 70.99

51805000.000 49.94645 505050000.000 70.99

51812000.000 49.94780 505120000.000 71.00

51819000.000 49.94915 505190000.000 71.00

51826000.000 49.95050 505260000.000 71.00

51833000.000 49.95185 505330000.000 71.00

51840000.000 49.95320 505400000.000 71.00

51847000.000 49.95455 505470000.000 71.01

51854000.000 49.95589 505540000.000 71.01

51861000.000 49.95724 505610000.000 71.01

51868000.000 49.95859 505680000.000 71.01

51875000.000 49.95994 505750000.000 71.01

51882000.000 49.96129 505820000.000 71.02

51889000.000 49.96264 505890000.000 71.02

51896000.000 49.96398 505960000.000 71.02

51903000.000 49.96533 506030000.000 71.02

51910000.000 49.96668 506100000.000 71.02

51917000.000 49.96803 506170000.000 71.03

51924000.000 49.96937 506240000.000 71.03

51931000.000 49.97072 506310000.000 71.03

51938000.000 49.97207 506380000.000 71.03

51945000.000 49.97342 506450000.000 71.03

51952000.000 49.97476 506520000.000 71.04

51959000.000 49.97611 506590000.000 71.04

51966000.000 49.97746 506660000.000 71.04

51973000.000 49.97880 506730000.000 71.04

51980000.000 49.98015 506800000.000 71.04

51987000.000 49.98149 506870000.000 71.05

51994000.000 49.98284 506940000.000 71.05

52001000.000 49.98419 507010000.000 71.05

52008000.000 49.98553 507080000.000 71.05

52015000.000 49.98688 507150000.000 71.05

52022000.000 49.98822 507220000.000 71.06

52029000.000 49.98957 507290000.000 71.06

52036000.000 49.99091 507360000.000 71.06

52043000.000 49.99226 507430000.000 71.06

52050000.000 49.99360 507500000.000 71.06

52057000.000 49.99495 507570000.000 71.07

52064000.000 49.99629 507640000.000 71.07

52071000.000 49.99764 507710000.000 71.07

52078000.000 49.99898 507780000.000 71.07

52085000.000 50.00032 507850000.000 71.07

52092000.000 50.00167 507920000.000 71.08

52099000.000 50.00301 507990000.000 71.08

52106000.000 50.00436 508060000.000 71.08

52113000.000 50.00570 508130000.000 71.08

52120000.000 50.00704 508200000.000 71.08

52127000.000 50.00839 508270000.000 71.08

52134000.000 50.00973 508340000.000 71.09

52141000.000 50.01107 508410000.000 71.09

52148000.000 50.01241 508480000.000 71.09

52155000.000 50.01376 508550000.000 71.09

52162000.000 50.01510 508620000.000 71.09

52169000.000 50.01644 508690000.000 71.10

52176000.000 50.01778 508760000.000 71.10

52183000.000 50.01913 508830000.000 71.10

52190000.000 50.02047 508900000.000 71.10

52197000.000 50.02181 508970000.000 71.10

52204000.000 50.02315 509040000.000 71.11

52211000.000 50.02449 509110000.000 71.11

52218000.000 50.02583 509180000.000 71.11

52225000.000 50.02717 509250000.000 71.11

52232000.000 50.02852 509320000.000 71.11

52239000.000 50.02986 509390000.000 71.12

52246000.000 50.03120 509460000.000 71.12

52253000.000 50.03254 509530000.000 71.12

52260000.000 50.03388 509600000.000 71.12

52267000.000 50.03522 509670000.000 71.12

52274000.000 50.03656 509740000.000 71.13

52281000.000 50.03790 509810000.000 71.13

52288000.000 50.03924 509880000.000 71.13

52295000.000 50.04058 509950000.000 71.13

52302000.000 50.04192 510020000.000 71.13

52309000.000 50.04326 510090000.000 71.14

52316000.000 50.04460 510160000.000 71.14

52323000.000 50.04594 510230000.000 71.14

52330000.000 50.04727 510300000.000 71.14

52337000.000 50.04861 510370000.000 71.14

52344000.000 50.04995 510440000.000 71.15

52351000.000 50.05129 510510000.000 71.15

52358000.000 50.05263 510580000.000 71.15

52365000.000 50.05397 510650000.000 71.15

52372000.000 50.05531 510720000.000 71.15

52379000.000 50.05664 510790000.000 71.16

52386000.000 50.05798 510860000.000 71.16

52393000.000 50.05932 510930000.000 71.16

52400000.000 50.06066 511000000.000 71.16

52407000.000 50.06199 511070000.000 71.16

52414000.000 50.06333 511140000.000 71.16

52421000.000 50.06467 511210000.000 71.17

52428000.000 50.06601 511280000.000 71.17

52435000.000 50.06734 511350000.000 71.17

52442000.000 50.06868 511420000.000 71.17

52449000.000 50.07002 511490000.000 71.17

52456000.000 50.07135 511560000.000 71.18

52463000.000 50.07269 511630000.000 71.18

52470000.000 50.07402 511700000.000 71.18

52477000.000 50.07536 511770000.000 71.18

52484000.000 50.07670 511840000.000 71.18

52491000.000 50.07803 511910000.000 71.19

52498000.000 50.07937 511980000.000 71.19

52505000.000 50.08070 512050000.000 71.19

52512000.000 50.08204 512120000.000 71.19

52519000.000 50.08337 512190000.000 71.19

52526000.000 50.08471 512260000.000 71.20

52533000.000 50.08604 512330000.000 71.20

52540000.000 50.08738 512400000.000 71.20

52547000.000 50.08871 512470000.000 71.20

52554000.000 50.09005 512540000.000 71.20

52561000.000 50.09138 512610000.000 71.21

52568000.000 50.09272 512680000.000 71.21

52575000.000 50.09405 512750000.000 71.21

52582000.000 50.09538 512820000.000 71.21

52589000.000 50.09672 512890000.000 71.21

52596000.000 50.09805 512960000.000 71.22

52603000.000 50.09938 513030000.000 71.22

52610000.000 50.10072 513100000.000 71.22

52617000.000 50.10205 513170000.000 71.22

52624000.000 50.10338 513240000.000 71.22

52631000.000 50.10472 513310000.000 71.23

52638000.000 50.10605 513380000.000 71.23

52645000.000 50.10738 513450000.000 71.23

52652000.000 50.10871 513520000.000 71.23

52659000.000 50.11005 513590000.000 71.23

52666000.000 50.11138 513660000.000 71.23

52673000.000 50.11271 513730000.000 71.24

52680000.000 50.11404 513800000.000 71.24

52687000.000 50.11537 513870000.000 71.24

52694000.000 50.11671 513940000.000 71.24

52701000.000 50.11804 514010000.000 71.24

52708000.000 50.11937 514080000.000 71.25

52715000.000 50.12070 514150000.000 71.25

52722000.000 50.12203 514220000.000 71.25

52729000.000 50.12336 514290000.000 71.25

52736000.000 50.12469 514360000.000 71.25

52743000.000 50.12602 514430000.000 71.26

52750000.000 50.12735 514500000.000 71.26

52757000.000 50.12868 514570000.000 71.26

52764000.000 50.13001 514640000.000 71.26

52771000.000 50.13134 514710000.000 71.26

52778000.000 50.13267 514780000.000 71.27

52785000.000 50.13400 514850000.000 71.27

52792000.000 50.13533 514920000.000 71.27

52799000.000 50.13666 514990000.000 71.27

52806000.000 50.13799 515060000.000 71.27

52813000.000 50.13932 515130000.000 71.28

52820000.000 50.14065 515200000.000 71.28

52827000.000 50.14198 515270000.000 71.28

52834000.000 50.14331 515340000.000 71.28

52841000.000 50.14464 515410000.000 71.28

52848000.000 50.14597 515480000.000 71.29

52855000.000 50.14729 515550000.000 71.29

52862000.000 50.14862 515620000.000 71.29

52869000.000 50.14995 515690000.000 71.29

52876000.000 50.15128 515760000.000 71.29

52883000.000 50.15261 515830000.000 71.30

52890000.000 50.15393 515900000.000 71.30

52897000.000 50.15526 515970000.000 71.30

52904000.000 50.15659 516040000.000 71.30

52911000.000 50.15792 516110000.000 71.30

52918000.000 50.15924 516180000.000 71.30

52925000.000 50.16057 516250000.000 71.31

52932000.000 50.16190 516320000.000 71.31

52939000.000 50.16322 516390000.000 71.31

52946000.000 50.16455 516460000.000 71.31

52953000.000 50.16588 516530000.000 71.31

52960000.000 50.16720 516600000.000 71.32

52967000.000 50.16853 516670000.000 71.32

52974000.000 50.16985 516740000.000 71.32

52981000.000 50.17118 516810000.000 71.32

52988000.000 50.17251 516880000.000 71.32

52995000.000 50.17383 516950000.000 71.33

53002000.000 50.17516 517020000.000 71.33

53009000.000 50.17648 517090000.000 71.33

53016000.000 50.17781 517160000.000 71.33

53023000.000 50.17913 517230000.000 71.33

53030000.000 50.18046 517300000.000 71.34

53037000.000 50.18178 517370000.000 71.34

53044000.000 50.18311 517440000.000 71.34

53051000.000 50.18443 517510000.000 71.34

53058000.000 50.18575 517580000.000 71.34

53065000.000 50.18708 517650000.000 71.35

53072000.000 50.18840 517720000.000 71.35

53079000.000 50.18973 517790000.000 71.35

53086000.000 50.19105 517860000.000 71.35

53093000.000 50.19237 517930000.000 71.35

53100000.000 50.19370 518000000.000 71.35

53107000.000 50.19502 518070000.000 71.36

53114000.000 50.19634 518140000.000 71.36

53121000.000 50.19767 518210000.000 71.36

53128000.000 50.19899 518280000.000 71.36

53135000.000 50.20031 518350000.000 71.36

53142000.000 50.20164 518420000.000 71.37

53149000.000 50.20296 518490000.000 71.37

53156000.000 50.20428 518560000.000 71.37

53163000.000 50.20560 518630000.000 71.37

53170000.000 50.20692 518700000.000 71.37

53177000.000 50.20825 518770000.000 71.38

53184000.000 50.20957 518840000.000 71.38

53191000.000 50.21089 518910000.000 71.38

53198000.000 50.21221 518980000.000 71.38

53205000.000 50.21353 519050000.000 71.38

53212000.000 50.21485 519120000.000 71.39

53219000.000 50.21617 519190000.000 71.39

53226000.000 50.21750 519260000.000 71.39

53233000.000 50.21882 519330000.000 71.39

53240000.000 50.22014 519400000.000 71.39

53247000.000 50.22146 519470000.000 71.40

53254000.000 50.22278 519540000.000 71.40

53261000.000 50.22410 519610000.000 71.40

53268000.000 50.22542 519680000.000 71.40

53275000.000 50.22674 519750000.000 71.40

53282000.000 50.22806 519820000.000 71.40

53289000.000 50.22938 519890000.000 71.41

53296000.000 50.23070 519960000.000 71.41

53303000.000 50.23202 520030000.000 71.41

53310000.000 50.23334 520100000.000 71.41

53317000.000 50.23466 520170000.000 71.41

53324000.000 50.23597 520240000.000 71.42

53331000.000 50.23729 520310000.000 71.42

53338000.000 50.23861 520380000.000 71.42

53345000.000 50.23993 520450000.000 71.42

53352000.000 50.24125 520520000.000 71.42

53359000.000 50.24257 520590000.000 71.43

53366000.000 50.24389 520660000.000 71.43

53373000.000 50.24520 520730000.000 71.43

53380000.000 50.24652 520800000.000 71.43

53387000.000 50.24784 520870000.000 71.43

53394000.000 50.24916 520940000.000 71.44

53401000.000 50.25047 521010000.000 71.44

53408000.000 50.25179 521080000.000 71.44

53415000.000 50.25311 521150000.000 71.44

53422000.000 50.25443 521220000.000 71.44

53429000.000 50.25574 521290000.000 71.45

53436000.000 50.25706 521360000.000 71.45

53443000.000 50.25838 521430000.000 71.45

53450000.000 50.25969 521500000.000 71.45

53457000.000 50.26101 521570000.000 71.45

53464000.000 50.26233 521640000.000 71.45

53471000.000 50.26364 521710000.000 71.46

53478000.000 50.26496 521780000.000 71.46

53485000.000 50.26627 521850000.000 71.46

53492000.000 50.26759 521920000.000 71.46

53499000.000 50.26890 521990000.000 71.46

53506000.000 50.27022 522060000.000 71.47

53513000.000 50.27153 522130000.000 71.47

53520000.000 50.27285 522200000.000 71.47

53527000.000 50.27416 522270000.000 71.47

53534000.000 50.27548 522340000.000 71.47

53541000.000 50.27679 522410000.000 71.48

53548000.000 50.27811 522480000.000 71.48

53555000.000 50.27942 522550000.000 71.48

53562000.000 50.28074 522620000.000 71.48

53569000.000 50.28205 522690000.000 71.48

53576000.000 50.28337 522760000.000 71.49

53583000.000 50.28468 522830000.000 71.49

53590000.000 50.28599 522900000.000 71.49

53597000.000 50.28731 522970000.000 71.49

53604000.000 50.28862 523040000.000 71.49

53611000.000 50.28993 523110000.000 71.50

53618000.000 50.29125 523180000.000 71.50

53625000.000 50.29256 523250000.000 71.50

53632000.000 50.29387 523320000.000 71.50

53639000.000 50.29519 523390000.000 71.50

53646000.000 50.29650 523460000.000 71.50

53653000.000 50.29781 523530000.000 71.51

53660000.000 50.29912 523600000.000 71.51

53667000.000 50.30044 523670000.000 71.51

53674000.000 50.30175 523740000.000 71.51

53681000.000 50.30306 523810000.000 71.51

53688000.000 50.30437 523880000.000 71.52

53695000.000 50.30568 523950000.000 71.52

53702000.000 50.30700 524020000.000 71.52

53709000.000 50.30831 524090000.000 71.52

53716000.000 50.30962 524160000.000 71.52

53723000.000 50.31093 524230000.000 71.53

53730000.000 50.31224 524300000.000 71.53

53737000.000 50.31355 524370000.000 71.53

53744000.000 50.31486 524440000.000 71.53

53751000.000 50.31617 524510000.000 71.53

53758000.000 50.31748 524580000.000 71.54

53765000.000 50.31879 524650000.000 71.54

53772000.000 50.32010 524720000.000 71.54

53779000.000 50.32141 524790000.000 71.54

53786000.000 50.32272 524860000.000 71.54

53793000.000 50.32403 524930000.000 71.54

53800000.000 50.32534 525000000.000 71.55

53807000.000 50.32665 525070000.000 71.55

53814000.000 50.32796 525140000.000 71.55

53821000.000 50.32927 525210000.000 71.55

53828000.000 50.33058 525280000.000 71.55

53835000.000 50.33189 525350000.000 71.56

53842000.000 50.33320 525420000.000 71.56

53849000.000 50.33451 525490000.000 71.56

53856000.000 50.33581 525560000.000 71.56

53863000.000 50.33712 525630000.000 71.56

53870000.000 50.33843 525700000.000 71.57

53877000.000 50.33974 525770000.000 71.57

53884000.000 50.34105 525840000.000 71.57

53891000.000 50.34236 525910000.000 71.57

53898000.000 50.34366 525980000.000 71.57

53905000.000 50.34497 526050000.000 71.58

53912000.000 50.34628 526120000.000 71.58

53919000.000 50.34759 526190000.000 71.58

53926000.000 50.34889 526260000.000 71.58

53933000.000 50.35020 526330000.000 71.58

53940000.000 50.35151 526400000.000 71.58

53947000.000 50.35281 526470000.000 71.59

53954000.000 50.35412 526540000.000 71.59

53961000.000 50.35543 526610000.000 71.59

53968000.000 50.35673 526680000.000 71.59

53975000.000 50.35804 526750000.000 71.59

53982000.000 50.35935 526820000.000 71.60

53989000.000 50.36065 526890000.000 71.60

53996000.000 50.36196 526960000.000 71.60

54003000.000 50.36326 527030000.000 71.60

54010000.000 50.36457 527100000.000 71.60

54017000.000 50.36587 527170000.000 71.61

54024000.000 50.36718 527240000.000 71.61

54031000.000 50.36848 527310000.000 71.61

54038000.000 50.36979 527380000.000 71.61

54045000.000 50.37109 527450000.000 71.61

54052000.000 50.37240 527520000.000 71.62

54059000.000 50.37370 527590000.000 71.62

54066000.000 50.37501 527660000.000 71.62

54073000.000 50.37631 527730000.000 71.62

54080000.000 50.37762 527800000.000 71.62

54087000.000 50.37892 527870000.000 71.62

54094000.000 50.38023 527940000.000 71.63

54101000.000 50.38153 528010000.000 71.63

54108000.000 50.38283 528080000.000 71.63

54115000.000 50.38414 528150000.000 71.63

54122000.000 50.38544 528220000.000 71.63

54129000.000 50.38674 528290000.000 71.64

54136000.000 50.38805 528360000.000 71.64

54143000.000 50.38935 528430000.000 71.64

54150000.000 50.39065 528500000.000 71.64

54157000.000 50.39195 528570000.000 71.64

54164000.000 50.39326 528640000.000 71.65

54171000.000 50.39456 528710000.000 71.65

54178000.000 50.39586 528780000.000 71.65

54185000.000 50.39716 528850000.000 71.65

54192000.000 50.39847 528920000.000 71.65

54199000.000 50.39977 528990000.000 71.66

54206000.000 50.40107 529060000.000 71.66

54213000.000 50.40237 529130000.000 71.66

54220000.000 50.40367 529200000.000 71.66

54227000.000 50.40497 529270000.000 71.66

54234000.000 50.40628 529340000.000 71.66

54241000.000 50.40758 529410000.000 71.67

54248000.000 50.40888 529480000.000 71.67

54255000.000 50.41018 529550000.000 71.67

54262000.000 50.41148 529620000.000 71.67

54269000.000 50.41278 529690000.000 71.67

54276000.000 50.41408 529760000.000 71.68

54283000.000 50.41538 529830000.000 71.68

54290000.000 50.41668 529900000.000 71.68

54297000.000 50.41798 529970000.000 71.68

54304000.000 50.41928 530040000.000 71.68

54311000.000 50.42058 530110000.000 71.69

54318000.000 50.42188 530180000.000 71.69

54325000.000 50.42318 530250000.000 71.69

54332000.000 50.42448 530320000.000 71.69

54339000.000 50.42578 530390000.000 71.69

54346000.000 50.42708 530460000.000 71.69

54353000.000 50.42838 530530000.000 71.70

54360000.000 50.42968 530600000.000 71.70

54367000.000 50.43097 530670000.000 71.70

54374000.000 50.43227 530740000.000 71.70

54381000.000 50.43357 530810000.000 71.70

54388000.000 50.43487 530880000.000 71.71

54395000.000 50.43617 530950000.000 71.71

54402000.000 50.43747 531020000.000 71.71

54409000.000 50.43876 531090000.000 71.71

54416000.000 50.44006 531160000.000 71.71

54423000.000 50.44136 531230000.000 71.72

54430000.000 50.44266 531300000.000 71.72

54437000.000 50.44395 531370000.000 71.72

54444000.000 50.44525 531440000.000 71.72

54451000.000 50.44655 531510000.000 71.72

54458000.000 50.44785 531580000.000 71.73

54465000.000 50.44914 531650000.000 71.73

54472000.000 50.45044 531720000.000 71.73

54479000.000 50.45174 531790000.000 71.73

54486000.000 50.45303 531860000.000 71.73

54493000.000 50.45433 531930000.000 71.73

54500000.000 50.45562 532000000.000 71.74

54507000.000 50.45692 532070000.000 71.74

54514000.000 50.45822 532140000.000 71.74

54521000.000 50.45951 532210000.000 71.74

54528000.000 50.46081 532280000.000 71.74

54535000.000 50.46210 532350000.000 71.75

54542000.000 50.46340 532420000.000 71.75

54549000.000 50.46469 532490000.000 71.75

54556000.000 50.46599 532560000.000 71.75

54563000.000 50.46728 532630000.000 71.75

54570000.000 50.46858 532700000.000 71.76

54577000.000 50.46987 532770000.000 71.76

54584000.000 50.47117 532840000.000 71.76

54591000.000 50.47246 532910000.000 71.76

54598000.000 50.47376 532980000.000 71.76

54605000.000 50.47505 533050000.000 71.76

54612000.000 50.47635 533120000.000 71.77

54619000.000 50.47764 533190000.000 71.77

54626000.000 50.47893 533260000.000 71.77

54633000.000 50.48023 533330000.000 71.77

54640000.000 50.48152 533400000.000 71.77

54647000.000 50.48281 533470000.000 71.78

54654000.000 50.48411 533540000.000 71.78

54661000.000 50.48540 533610000.000 71.78

54668000.000 50.48669 533680000.000 71.78

54675000.000 50.48799 533750000.000 71.78

54682000.000 50.48928 533820000.000 71.79

54689000.000 50.49057 533890000.000 71.79

54696000.000 50.49186 533960000.000 71.79

54703000.000 50.49316 534030000.000 71.79

54710000.000 50.49445 534100000.000 71.79

54717000.000 50.49574 534170000.000 71.79

54724000.000 50.49703 534240000.000 71.80

54731000.000 50.49832 534310000.000 71.80

54738000.000 50.49962 534380000.000 71.80

54745000.000 50.50091 534450000.000 71.80

54752000.000 50.50220 534520000.000 71.80

54759000.000 50.50349 534590000.000 71.81

54766000.000 50.50478 534660000.000 71.81

54773000.000 50.50607 534730000.000 71.81

54780000.000 50.50736 534800000.000 71.81

54787000.000 50.50865 534870000.000 71.81

54794000.000 50.50994 534940000.000 71.82

54801000.000 50.51123 535010000.000 71.82

54808000.000 50.51252 535080000.000 71.82

54815000.000 50.51381 535150000.000 71.82

54822000.000 50.51510 535220000.000 71.82

54829000.000 50.51639 535290000.000 71.83

54836000.000 50.51768 535360000.000 71.83

54843000.000 50.51897 535430000.000 71.83

54850000.000 50.52026 535500000.000 71.83

54857000.000 50.52155 535570000.000 71.83

54864000.000 50.52284 535640000.000 71.83

54871000.000 50.52413 535710000.000 71.84

54878000.000 50.52542 535780000.000 71.84

54885000.000 50.52671 535850000.000 71.84

54892000.000 50.52800 535920000.000 71.84

54899000.000 50.52929 535990000.000 71.84

54906000.000 50.53058 536060000.000 71.85

54913000.000 50.53186 536130000.000 71.85

54920000.000 50.53315 536200000.000 71.85

54927000.000 50.53444 536270000.000 71.85

54934000.000 50.53573 536340000.000 71.85

54941000.000 50.53702 536410000.000 71.86

54948000.000 50.53830 536480000.000 71.86

54955000.000 50.53959 536550000.000 71.86

54962000.000 50.54088 536620000.000 71.86

54969000.000 50.54217 536690000.000 71.86

54976000.000 50.54345 536760000.000 71.86

54983000.000 50.54474 536830000.000 71.87

54990000.000 50.54603 536900000.000 71.87

54997000.000 50.54731 536970000.000 71.87

55004000.000 50.54860 537040000.000 71.87

55011000.000 50.54989 537110000.000 71.87

55018000.000 50.55117 537180000.000 71.88

55025000.000 50.55246 537250000.000 71.88

55032000.000 50.55375 537320000.000 71.88

55039000.000 50.55503 537390000.000 71.88

55046000.000 50.55632 537460000.000 71.88

55053000.000 50.55760 537530000.000 71.89

55060000.000 50.55889 537600000.000 71.89

55067000.000 50.56017 537670000.000 71.89

55074000.000 50.56146 537740000.000 71.89

55081000.000 50.56275 537810000.000 71.89

55088000.000 50.56403 537880000.000 71.89

55095000.000 50.56532 537950000.000 71.90

55102000.000 50.56660 538020000.000 71.90

55109000.000 50.56789 538090000.000 71.90

55116000.000 50.56917 538160000.000 71.90

55123000.000 50.57045 538230000.000 71.90

55130000.000 50.57174 538300000.000 71.91

55137000.000 50.57302 538370000.000 71.91

55144000.000 50.57431 538440000.000 71.91

55151000.000 50.57559 538510000.000 71.91

55158000.000 50.57687 538580000.000 71.91

55165000.000 50.57816 538650000.000 71.91

55172000.000 50.57944 538720000.000 71.92

55179000.000 50.58072 538790000.000 71.92

55186000.000 50.58201 538860000.000 71.92

55193000.000 50.58329 538930000.000 71.92

55200000.000 50.58457 539000000.000 71.92

55207000.000 50.58586 539070000.000 71.93

55214000.000 50.58714 539140000.000 71.93

55221000.000 50.58842 539210000.000 71.93

55228000.000 50.58971 539280000.000 71.93

55235000.000 50.59099 539350000.000 71.93

55242000.000 50.59227 539420000.000 71.94

55249000.000 50.59355 539490000.000 71.94

55256000.000 50.59483 539560000.000 71.94

55263000.000 50.59612 539630000.000 71.94

55270000.000 50.59740 539700000.000 71.94

55277000.000 50.59868 539770000.000 71.94

55284000.000 50.59996 539840000.000 71.95

55291000.000 50.60124 539910000.000 71.95

55298000.000 50.60252 539980000.000 71.95

55305000.000 50.60380 540050000.000 71.95

55312000.000 50.60508 540120000.000 71.95

55319000.000 50.60637 540190000.000 71.96

55326000.000 50.60765 540260000.000 71.96

55333000.000 50.60893 540330000.000 71.96

55340000.000 50.61021 540400000.000 71.96

55347000.000 50.61149 540470000.000 71.96

55354000.000 50.61277 540540000.000 71.97

55361000.000 50.61405 540610000.000 71.97

55368000.000 50.61533 540680000.000 71.97

55375000.000 50.61661 540750000.000 71.97

55382000.000 50.61789 540820000.000 71.97

55389000.000 50.61917 540890000.000 71.97

55396000.000 50.62045 540960000.000 71.98

55403000.000 50.62173 541030000.000 71.98

55410000.000 50.62300 541100000.000 71.98

55417000.000 50.62428 541170000.000 71.98

55424000.000 50.62556 541240000.000 71.98

55431000.000 50.62684 541310000.000 71.99

55438000.000 50.62812 541380000.000 71.99

55445000.000 50.62940 541450000.000 71.99

55452000.000 50.63068 541520000.000 71.99

55459000.000 50.63195 541590000.000 71.99

55466000.000 50.63323 541660000.000 72.00

55473000.000 50.63451 541730000.000 72.00

55480000.000 50.63579 541800000.000 72.00

55487000.000 50.63707 541870000.000 72.00

55494000.000 50.63834 541940000.000 72.00

55501000.000 50.63962 542010000.000 72.00

55508000.000 50.64090 542080000.000 72.01

55515000.000 50.64218 542150000.000 72.01

55522000.000 50.64345 542220000.000 72.01

55529000.000 50.64473 542290000.000 72.01

55536000.000 50.64601 542360000.000 72.01

55543000.000 50.64728 542430000.000 72.02

55550000.000 50.64856 542500000.000 72.02

55557000.000 50.64984 542570000.000 72.02

55564000.000 50.65111 542640000.000 72.02

55571000.000 50.65239 542710000.000 72.02

55578000.000 50.65366 542780000.000 72.02

55585000.000 50.65494 542850000.000 72.03

55592000.000 50.65622 542920000.000 72.03

55599000.000 50.65749 542990000.000 72.03

55606000.000 50.65877 543060000.000 72.03

55613000.000 50.66004 543130000.000 72.03

55620000.000 50.66132 543200000.000 72.04

55627000.000 50.66259 543270000.000 72.04

55634000.000 50.66387 543340000.000 72.04

55641000.000 50.66514 543410000.000 72.04

55648000.000 50.66642 543480000.000 72.04

55655000.000 50.66769 543550000.000 72.05

55662000.000 50.66897 543620000.000 72.05

55669000.000 50.67024 543690000.000 72.05

55676000.000 50.67152 543760000.000 72.05

55683000.000 50.67279 543830000.000 72.05

55690000.000 50.67406 543900000.000 72.05

55697000.000 50.67534 543970000.000 72.06

55704000.000 50.67661 544040000.000 72.06

55711000.000 50.67788 544110000.000 72.06

55718000.000 50.67916 544180000.000 72.06

55725000.000 50.68043 544250000.000 72.06

55732000.000 50.68170 544320000.000 72.07

55739000.000 50.68298 544390000.000 72.07

55746000.000 50.68425 544460000.000 72.07

55753000.000 50.68552 544530000.000 72.07

55760000.000 50.68680 544600000.000 72.07

55767000.000 50.68807 544670000.000 72.08

55774000.000 50.68934 544740000.000 72.08

55781000.000 50.69061 544810000.000 72.08

55788000.000 50.69189 544880000.000 72.08

55795000.000 50.69316 544950000.000 72.08

55802000.000 50.69443 545020000.000 72.08

55809000.000 50.69570 545090000.000 72.09

55816000.000 50.69697 545160000.000 72.09

55823000.000 50.69824 545230000.000 72.09

55830000.000 50.69952 545300000.000 72.09

55837000.000 50.70079 545370000.000 72.09

55844000.000 50.70206 545440000.000 72.10

55851000.000 50.70333 545510000.000 72.10

55858000.000 50.70460 545580000.000 72.10

55865000.000 50.70587 545650000.000 72.10

55872000.000 50.70714 545720000.000 72.10

55879000.000 50.70841 545790000.000 72.10

55886000.000 50.70968 545860000.000 72.11

55893000.000 50.71095 545930000.000 72.11

55900000.000 50.71222 546000000.000 72.11

55907000.000 50.71349 546070000.000 72.11

55914000.000 50.71476 546140000.000 72.11

55921000.000 50.71603 546210000.000 72.12

55928000.000 50.71730 546280000.000 72.12

55935000.000 50.71857 546350000.000 72.12

55942000.000 50.71984 546420000.000 72.12

55949000.000 50.72111 546490000.000 72.12

55956000.000 50.72238 546560000.000 72.12

55963000.000 50.72365 546630000.000 72.13

55970000.000 50.72492 546700000.000 72.13

55977000.000 50.72619 546770000.000 72.13

55984000.000 50.72745 546840000.000 72.13

55991000.000 50.72872 546910000.000 72.13

55998000.000 50.72999 546980000.000 72.14

56005000.000 50.73126 547050000.000 72.14

56012000.000 50.73253 547120000.000 72.14

56019000.000 50.73380 547190000.000 72.14

56026000.000 50.73506 547260000.000 72.14

56033000.000 50.73633 547330000.000 72.15

56040000.000 50.73760 547400000.000 72.15

56047000.000 50.73887 547470000.000 72.15

56054000.000 50.74013 547540000.000 72.15

56061000.000 50.74140 547610000.000 72.15

56068000.000 50.74267 547680000.000 72.15

56075000.000 50.74393 547750000.000 72.16

56082000.000 50.74520 547820000.000 72.16

56089000.000 50.74647 547890000.000 72.16

56096000.000 50.74774 547960000.000 72.16

56103000.000 50.74900 548030000.000 72.16

56110000.000 50.75027 548100000.000 72.17

56117000.000 50.75153 548170000.000 72.17

56124000.000 50.75280 548240000.000 72.17

56131000.000 50.75407 548310000.000 72.17

56138000.000 50.75533 548380000.000 72.17

56145000.000 50.75660 548450000.000 72.17

56152000.000 50.75786 548520000.000 72.18

56159000.000 50.75913 548590000.000 72.18

56166000.000 50.76039 548660000.000 72.18

56173000.000 50.76166 548730000.000 72.18

56180000.000 50.76292 548800000.000 72.18

56187000.000 50.76419 548870000.000 72.19

56194000.000 50.76545 548940000.000 72.19

56201000.000 50.76672 549010000.000 72.19

56208000.000 50.76798 549080000.000 72.19

56215000.000 50.76925 549150000.000 72.19

56222000.000 50.77051 549220000.000 72.20

56229000.000 50.77178 549290000.000 72.20

56236000.000 50.77304 549360000.000 72.20

56243000.000 50.77430 549430000.000 72.20

56250000.000 50.77557 549500000.000 72.20

56257000.000 50.77683 549570000.000 72.20

56264000.000 50.77810 549640000.000 72.21

56271000.000 50.77936 549710000.000 72.21

56278000.000 50.78062 549780000.000 72.21

56285000.000 50.78189 549850000.000 72.21

56292000.000 50.78315 549920000.000 72.21

56299000.000 50.78441 549990000.000 72.22

56306000.000 50.78567 550060000.000 72.22

56313000.000 50.78694 550130000.000 72.22

56320000.000 50.78820 550200000.000 72.22

56327000.000 50.78946 550270000.000 72.22

56334000.000 50.79072 550340000.000 72.22

56341000.000 50.79199 550410000.000 72.23

56348000.000 50.79325 550480000.000 72.23

56355000.000 50.79451 550550000.000 72.23

56362000.000 50.79577 550620000.000 72.23

56369000.000 50.79703 550690000.000 72.23

56376000.000 50.79830 550760000.000 72.24

56383000.000 50.79956 550830000.000 72.24

56390000.000 50.80082 550900000.000 72.24

56397000.000 50.80208 550970000.000 72.24

56404000.000 50.80334 551040000.000 72.24

56411000.000 50.80460 551110000.000 72.24

56418000.000 50.80586 551180000.000 72.25

56425000.000 50.80712 551250000.000 72.25

56432000.000 50.80838 551320000.000 72.25

56439000.000 50.80964 551390000.000 72.25

56446000.000 50.81090 551460000.000 72.25

56453000.000 50.81216 551530000.000 72.26

56460000.000 50.81342 551600000.000 72.26

56467000.000 50.81468 551670000.000 72.26

56474000.000 50.81594 551740000.000 72.26

56481000.000 50.81720 551810000.000 72.26

56488000.000 50.81846 551880000.000 72.26

56495000.000 50.81972 551950000.000 72.27

56502000.000 50.82098 552020000.000 72.27

56509000.000 50.82224 552090000.000 72.27

56516000.000 50.82350 552160000.000 72.27

56523000.000 50.82476 552230000.000 72.27

56530000.000 50.82602 552300000.000 72.28

56537000.000 50.82728 552370000.000 72.28

56544000.000 50.82853 552440000.000 72.28

56551000.000 50.82979 552510000.000 72.28

56558000.000 50.83105 552580000.000 72.28

56565000.000 50.83231 552650000.000 72.28

56572000.000 50.83357 552720000.000 72.29

56579000.000 50.83483 552790000.000 72.29

56586000.000 50.83608 552860000.000 72.29

56593000.000 50.83734 552930000.000 72.29

56600000.000 50.83860 553000000.000 72.29

56607000.000 50.83986 553070000.000 72.30

56614000.000 50.84111 553140000.000 72.30

56621000.000 50.84237 553210000.000 72.30

56628000.000 50.84363 553280000.000 72.30

56635000.000 50.84488 553350000.000 72.30

56642000.000 50.84614 553420000.000 72.31

56649000.000 50.84740 553490000.000 72.31

56656000.000 50.84865 553560000.000 72.31

56663000.000 50.84991 553630000.000 72.31

56670000.000 50.85117 553700000.000 72.31

56677000.000 50.85242 553770000.000 72.31

56684000.000 50.85368 553840000.000 72.32

56691000.000 50.85494 553910000.000 72.32

56698000.000 50.85619 553980000.000 72.32

56705000.000 50.85745 554050000.000 72.32

56712000.000 50.85870 554120000.000 72.32

56719000.000 50.85996 554190000.000 72.33

56726000.000 50.86121 554260000.000 72.33

56733000.000 50.86247 554330000.000 72.33

56740000.000 50.86372 554400000.000 72.33

56747000.000 50.86498 554470000.000 72.33

56754000.000 50.86623 554540000.000 72.33

56761000.000 50.86749 554610000.000 72.34

56768000.000 50.86874 554680000.000 72.34

56775000.000 50.87000 554750000.000 72.34

56782000.000 50.87125 554820000.000 72.34

56789000.000 50.87251 554890000.000 72.34

56796000.000 50.87376 554960000.000 72.35

56803000.000 50.87501 555030000.000 72.35

56810000.000 50.87627 555100000.000 72.35

56817000.000 50.87752 555170000.000 72.35

56824000.000 50.87877 555240000.000 72.35

56831000.000 50.88003 555310000.000 72.35

56838000.000 50.88128 555380000.000 72.36

56845000.000 50.88253 555450000.000 72.36

56852000.000 50.88379 555520000.000 72.36

56859000.000 50.88504 555590000.000 72.36

56866000.000 50.88629 555660000.000 72.36

56873000.000 50.88755 555730000.000 72.37

56880000.000 50.88880 555800000.000 72.37

56887000.000 50.89005 555870000.000 72.37

56894000.000 50.89130 555940000.000 72.37

56901000.000 50.89256 556010000.000 72.37

56908000.000 50.89381 556080000.000 72.37

56915000.000 50.89506 556150000.000 72.38

56922000.000 50.89631 556220000.000 72.38

56929000.000 50.89756 556290000.000 72.38

56936000.000 50.89882 556360000.000 72.38

56943000.000 50.90007 556430000.000 72.38

56950000.000 50.90132 556500000.000 72.39

56957000.000 50.90257 556570000.000 72.39

56964000.000 50.90382 556640000.000 72.39

56971000.000 50.90507 556710000.000 72.39

56978000.000 50.90632 556780000.000 72.39

56985000.000 50.90757 556850000.000 72.39

56992000.000 50.90882 556920000.000 72.40

56999000.000 50.91007 556990000.000 72.40

57006000.000 50.91132 557060000.000 72.40

57013000.000 50.91258 557130000.000 72.40

57020000.000 50.91383 557200000.000 72.40

57027000.000 50.91508 557270000.000 72.41

57034000.000 50.91633 557340000.000 72.41

57041000.000 50.91757 557410000.000 72.41

57048000.000 50.91882 557480000.000 72.41

57055000.000 50.92007 557550000.000 72.41

57062000.000 50.92132 557620000.000 72.41

57069000.000 50.92257 557690000.000 72.42

57076000.000 50.92382 557760000.000 72.42

57083000.000 50.92507 557830000.000 72.42

57090000.000 50.92632 557900000.000 72.42

57097000.000 50.92757 557970000.000 72.42

57104000.000 50.92882 558040000.000 72.43

57111000.000 50.93007 558110000.000 72.43

57118000.000 50.93131 558180000.000 72.43

57125000.000 50.93256 558250000.000 72.43

57132000.000 50.93381 558320000.000 72.43

57139000.000 50.93506 558390000.000 72.43

57146000.000 50.93631 558460000.000 72.44

57153000.000 50.93755 558530000.000 72.44

57160000.000 50.93880 558600000.000 72.44

57167000.000 50.94005 558670000.000 72.44

57174000.000 50.94130 558740000.000 72.44

57181000.000 50.94254 558810000.000 72.45

57188000.000 50.94379 558880000.000 72.45

57195000.000 50.94504 558950000.000 72.45

57202000.000 50.94629 559020000.000 72.45

57209000.000 50.94753 559090000.000 72.45

57216000.000 50.94878 559160000.000 72.45

57223000.000 50.95003 559230000.000 72.46

57230000.000 50.95127 559300000.000 72.46

57237000.000 50.95252 559370000.000 72.46

57244000.000 50.95376 559440000.000 72.46

57251000.000 50.95501 559510000.000 72.46

57258000.000 50.95626 559580000.000 72.47

57265000.000 50.95750 559650000.000 72.47

57272000.000 50.95875 559720000.000 72.47

57279000.000 50.95999 559790000.000 72.47

57286000.000 50.96124 559860000.000 72.47

57293000.000 50.96249 559930000.000 72.47

57300000.000 50.96373 560000000.000 72.48

57307000.000 50.96498 560070000.000 72.48

57314000.000 50.96622 560140000.000 72.48

57321000.000 50.96747 560210000.000 72.48

57328000.000 50.96871 560280000.000 72.48

57335000.000 50.96995 560350000.000 72.49

57342000.000 50.97120 560420000.000 72.49

57349000.000 50.97244 560490000.000 72.49

57356000.000 50.97369 560560000.000 72.49

57363000.000 50.97493 560630000.000 72.49

57370000.000 50.97618 560700000.000 72.49

57377000.000 50.97742 560770000.000 72.50

57384000.000 50.97866 560840000.000 72.50

57391000.000 50.97991 560910000.000 72.50

57398000.000 50.98115 560980000.000 72.50

57405000.000 50.98239 561050000.000 72.50

57412000.000 50.98364 561120000.000 72.51

57419000.000 50.98488 561190000.000 72.51

57426000.000 50.98612 561260000.000 72.51

57433000.000 50.98737 561330000.000 72.51

57440000.000 50.98861 561400000.000 72.51

57447000.000 50.98985 561470000.000 72.51

57454000.000 50.99110 561540000.000 72.52

57461000.000 50.99234 561610000.000 72.52

57468000.000 50.99358 561680000.000 72.52

57475000.000 50.99482 561750000.000 72.52

57482000.000 50.99606 561820000.000 72.52

57489000.000 50.99731 561890000.000 72.53

57496000.000 50.99855 561960000.000 72.53

57503000.000 50.99979 562030000.000 72.53

57510000.000 51.00103 562100000.000 72.53

57517000.000 51.00227 562170000.000 72.53

57524000.000 51.00351 562240000.000 72.53

57531000.000 51.00476 562310000.000 72.54

57538000.000 51.00600 562380000.000 72.54

57545000.000 51.00724 562450000.000 72.54

57552000.000 51.00848 562520000.000 72.54

57559000.000 51.00972 562590000.000 72.54

57566000.000 51.01096 562660000.000 72.54

57573000.000 51.01220 562730000.000 72.55

57580000.000 51.01344 562800000.000 72.55

57587000.000 51.01468 562870000.000 72.55

57594000.000 51.01592 562940000.000 72.55

57601000.000 51.01716 563010000.000 72.55

57608000.000 51.01840 563080000.000 72.56

57615000.000 51.01964 563150000.000 72.56

57622000.000 51.02088 563220000.000 72.56

57629000.000 51.02212 563290000.000 72.56

57636000.000 51.02336 563360000.000 72.56

57643000.000 51.02460 563430000.000 72.56

57650000.000 51.02584 563500000.000 72.57

57657000.000 51.02708 563570000.000 72.57

57664000.000 51.02832 563640000.000 72.57

57671000.000 51.02956 563710000.000 72.57

57678000.000 51.03079 563780000.000 72.57

57685000.000 51.03203 563850000.000 72.58

57692000.000 51.03327 563920000.000 72.58

57699000.000 51.03451 563990000.000 72.58

57706000.000 51.03575 564060000.000 72.58

57713000.000 51.03699 564130000.000 72.58

57720000.000 51.03822 564200000.000 72.58

57727000.000 51.03946 564270000.000 72.59

57734000.000 51.04070 564340000.000 72.59

57741000.000 51.04194 564410000.000 72.59

57748000.000 51.04317 564480000.000 72.59

57755000.000 51.04441 564550000.000 72.59

57762000.000 51.04565 564620000.000 72.60

57769000.000 51.04689 564690000.000 72.60

57776000.000 51.04812 564760000.000 72.60

57783000.000 51.04936 564830000.000 72.60

57790000.000 51.05060 564900000.000 72.60

57797000.000 51.05183 564970000.000 72.60

57804000.000 51.05307 565040000.000 72.61

57811000.000 51.05431 565110000.000 72.61

57818000.000 51.05554 565180000.000 72.61

57825000.000 51.05678 565250000.000 72.61

57832000.000 51.05802 565320000.000 72.61

57839000.000 51.05925 565390000.000 72.62

57846000.000 51.06049 565460000.000 72.62

57853000.000 51.06172 565530000.000 72.62

57860000.000 51.06296 565600000.000 72.62

57867000.000 51.06419 565670000.000 72.62

57874000.000 51.06543 565740000.000 72.62

57881000.000 51.06666 565810000.000 72.63

57888000.000 51.06790 565880000.000 72.63

57895000.000 51.06913 565950000.000 72.63

57902000.000 51.07037 566020000.000 72.63

57909000.000 51.07160 566090000.000 72.63

57916000.000 51.07284 566160000.000 72.63

57923000.000 51.07407 566230000.000 72.64

57930000.000 51.07531 566300000.000 72.64

57937000.000 51.07654 566370000.000 72.64

57944000.000 51.07778 566440000.000 72.64

57951000.000 51.07901 566510000.000 72.64

57958000.000 51.08024 566580000.000 72.65

57965000.000 51.08148 566650000.000 72.65

57972000.000 51.08271 566720000.000 72.65

57979000.000 51.08395 566790000.000 72.65

57986000.000 51.08518 566860000.000 72.65

57993000.000 51.08641 566930000.000 72.65

58000000.000 51.08765 567000000.000 72.66

58007000.000 51.08888 567070000.000 72.66

58014000.000 51.09011 567140000.000 72.66

58021000.000 51.09134 567210000.000 72.66

58028000.000 51.09258 567280000.000 72.66

58035000.000 51.09381 567350000.000 72.67

58042000.000 51.09504 567420000.000 72.67

58049000.000 51.09627 567490000.000 72.67

58056000.000 51.09751 567560000.000 72.67

58063000.000 51.09874 567630000.000 72.67

58070000.000 51.09997 567700000.000 72.67

58077000.000 51.10120 567770000.000 72.68

58084000.000 51.10243 567840000.000 72.68

58091000.000 51.10367 567910000.000 72.68

58098000.000 51.10490 567980000.000 72.68

58105000.000 51.10613 568050000.000 72.68

58112000.000 51.10736 568120000.000 72.69

58119000.000 51.10859 568190000.000 72.69

58126000.000 51.10982 568260000.000 72.69

58133000.000 51.11105 568330000.000 72.69

58140000.000 51.11228 568400000.000 72.69

58147000.000 51.11351 568470000.000 72.69

58154000.000 51.11475 568540000.000 72.70

58161000.000 51.11598 568610000.000 72.70

58168000.000 51.11721 568680000.000 72.70

58175000.000 51.11844 568750000.000 72.70

58182000.000 51.11967 568820000.000 72.70

58189000.000 51.12090 568890000.000 72.70

58196000.000 51.12213 568960000.000 72.71

58203000.000 51.12336 569030000.000 72.71

58210000.000 51.12459 569100000.000 72.71

58217000.000 51.12582 569170000.000 72.71

58224000.000 51.12704 569240000.000 72.71

58231000.000 51.12827 569310000.000 72.72

58238000.000 51.12950 569380000.000 72.72

58245000.000 51.13073 569450000.000 72.72

58252000.000 51.13196 569520000.000 72.72

58259000.000 51.13319 569590000.000 72.72

58266000.000 51.13442 569660000.000 72.72

58273000.000 51.13565 569730000.000 72.73

58280000.000 51.13688 569800000.000 72.73

58287000.000 51.13810 569870000.000 72.73

58294000.000 51.13933 569940000.000 72.73

58301000.000 51.14056 570010000.000 72.73

58308000.000 51.14179 570080000.000 72.74

58315000.000 51.14302 570150000.000 72.74

58322000.000 51.14424 570220000.000 72.74

58329000.000 51.14547 570290000.000 72.74

58336000.000 51.14670 570360000.000 72.74

58343000.000 51.14793 570430000.000 72.74

58350000.000 51.14915 570500000.000 72.75

58357000.000 51.15038 570570000.000 72.75

58364000.000 51.15161 570640000.000 72.75

58371000.000 51.15284 570710000.000 72.75

58378000.000 51.15406 570780000.000 72.75

58385000.000 51.15529 570850000.000 72.75

58392000.000 51.15652 570920000.000 72.76

58399000.000 51.15774 570990000.000 72.76

58406000.000 51.15897 571060000.000 72.76

58413000.000 51.16019 571130000.000 72.76

58420000.000 51.16142 571200000.000 72.76

58427000.000 51.16265 571270000.000 72.77

58434000.000 51.16387 571340000.000 72.77

58441000.000 51.16510 571410000.000 72.77

58448000.000 51.16632 571480000.000 72.77

58455000.000 51.16755 571550000.000 72.77

58462000.000 51.16877 571620000.000 72.77

58469000.000 51.17000 571690000.000 72.78

58476000.000 51.17123 571760000.000 72.78

58483000.000 51.17245 571830000.000 72.78

58490000.000 51.17368 571900000.000 72.78

58497000.000 51.17490 571970000.000 72.78

58504000.000 51.17612 572040000.000 72.79

58511000.000 51.17735 572110000.000 72.79

58518000.000 51.17857 572180000.000 72.79

58525000.000 51.17980 572250000.000 72.79

58532000.000 51.18102 572320000.000 72.79

58539000.000 51.18225 572390000.000 72.79

58546000.000 51.18347 572460000.000 72.80

58553000.000 51.18469 572530000.000 72.80

58560000.000 51.18592 572600000.000 72.80

58567000.000 51.18714 572670000.000 72.80

58574000.000 51.18837 572740000.000 72.80

58581000.000 51.18959 572810000.000 72.80

58588000.000 51.19081 572880000.000 72.81

58595000.000 51.19204 572950000.000 72.81

58602000.000 51.19326 573020000.000 72.81

58609000.000 51.19448 573090000.000 72.81

58616000.000 51.19570 573160000.000 72.81

58623000.000 51.19693 573230000.000 72.82

58630000.000 51.19815 573300000.000 72.82

58637000.000 51.19937 573370000.000 72.82

58644000.000 51.20059 573440000.000 72.82

58651000.000 51.20182 573510000.000 72.82

58658000.000 51.20304 573580000.000 72.82

58665000.000 51.20426 573650000.000 72.83

58672000.000 51.20548 573720000.000 72.83

58679000.000 51.20670 573790000.000 72.83

58686000.000 51.20793 573860000.000 72.83

58693000.000 51.20915 573930000.000 72.83

58700000.000 51.21037 574000000.000 72.84

58707000.000 51.21159 574070000.000 72.84

58714000.000 51.21281 574140000.000 72.84

58721000.000 51.21403 574210000.000 72.84

58728000.000 51.21525 574280000.000 72.84

58735000.000 51.21647 574350000.000 72.84

58742000.000 51.21770 574420000.000 72.85

58749000.000 51.21892 574490000.000 72.85

58756000.000 51.22014 574560000.000 72.85

58763000.000 51.22136 574630000.000 72.85

58770000.000 51.22258 574700000.000 72.85

58777000.000 51.22380 574770000.000 72.85

58784000.000 51.22502 574840000.000 72.86

58791000.000 51.22624 574910000.000 72.86

58798000.000 51.22746 574980000.000 72.86

58805000.000 51.22868 575050000.000 72.86

58812000.000 51.22990 575120000.000 72.86

58819000.000 51.23112 575190000.000 72.87

58826000.000 51.23233 575260000.000 72.87

58833000.000 51.23355 575330000.000 72.87

58840000.000 51.23477 575400000.000 72.87

58847000.000 51.23599 575470000.000 72.87

58854000.000 51.23721 575540000.000 72.87

58861000.000 51.23843 575610000.000 72.88

58868000.000 51.23965 575680000.000 72.88

58875000.000 51.24087 575750000.000 72.88

58882000.000 51.24209 575820000.000 72.88

58889000.000 51.24330 575890000.000 72.88

58896000.000 51.24452 575960000.000 72.88

58903000.000 51.24574 576030000.000 72.89

58910000.000 51.24696 576100000.000 72.89

58917000.000 51.24818 576170000.000 72.89

58924000.000 51.24939 576240000.000 72.89

58931000.000 51.25061 576310000.000 72.89

58938000.000 51.25183 576380000.000 72.90

58945000.000 51.25305 576450000.000 72.90

58952000.000 51.25426 576520000.000 72.90

58959000.000 51.25548 576590000.000 72.90

58966000.000 51.25670 576660000.000 72.90

58973000.000 51.25791 576730000.000 72.90

58980000.000 51.25913 576800000.000 72.91

58987000.000 51.26035 576870000.000 72.91

58994000.000 51.26156 576940000.000 72.91

59001000.000 51.26278 577010000.000 72.91

59008000.000 51.26400 577080000.000 72.91

59015000.000 51.26521 577150000.000 72.91

59022000.000 51.26643 577220000.000 72.92

59029000.000 51.26765 577290000.000 72.92

59036000.000 51.26886 577360000.000 72.92

59043000.000 51.27008 577430000.000 72.92

59050000.000 51.27129 577500000.000 72.92

59057000.000 51.27251 577570000.000 72.93

59064000.000 51.27372 577640000.000 72.93

59071000.000 51.27494 577710000.000 72.93

59078000.000 51.27615 577780000.000 72.93

59085000.000 51.27737 577850000.000 72.93

59092000.000 51.27858 577920000.000 72.93

59099000.000 51.27980 577990000.000 72.94

59106000.000 51.28101 578060000.000 72.94

59113000.000 51.28223 578130000.000 72.94

59120000.000 51.28344 578200000.000 72.94

59127000.000 51.28466 578270000.000 72.94

59134000.000 51.28587 578340000.000 72.94

59141000.000 51.28709 578410000.000 72.95

59148000.000 51.28830 578480000.000 72.95

59155000.000 51.28951 578550000.000 72.95

59162000.000 51.29073 578620000.000 72.95

59169000.000 51.29194 578690000.000 72.95

59176000.000 51.29315 578760000.000 72.96

59183000.000 51.29437 578830000.000 72.96

59190000.000 51.29558 578900000.000 72.96

59197000.000 51.29679 578970000.000 72.96

59204000.000 51.29801 579040000.000 72.96

59211000.000 51.29922 579110000.000 72.96

59218000.000 51.30043 579180000.000 72.97

59225000.000 51.30165 579250000.000 72.97

59232000.000 51.30286 579320000.000 72.97

59239000.000 51.30407 579390000.000 72.97

59246000.000 51.30528 579460000.000 72.97

59253000.000 51.30650 579530000.000 72.97

59260000.000 51.30771 579600000.000 72.98

59267000.000 51.30892 579670000.000 72.98

59274000.000 51.31013 579740000.000 72.98

59281000.000 51.31134 579810000.000 72.98

59288000.000 51.31256 579880000.000 72.98

59295000.000 51.31377 579950000.000 72.99

59302000.000 51.31498 580020000.000 72.99

59309000.000 51.31619 580090000.000 72.99

59316000.000 51.31740 580160000.000 72.99

59323000.000 51.31861 580230000.000 72.99

59330000.000 51.31982 580300000.000 72.99

59337000.000 51.32103 580370000.000 73.00

59344000.000 51.32225 580440000.000 73.00

59351000.000 51.32346 580510000.000 73.00

59358000.000 51.32467 580580000.000 73.00

59365000.000 51.32588 580650000.000 73.00

59372000.000 51.32709 580720000.000 73.00

59379000.000 51.32830 580790000.000 73.01

59386000.000 51.32951 580860000.000 73.01

59393000.000 51.33072 580930000.000 73.01

59400000.000 51.33193 581000000.000 73.01

59407000.000 51.33314 581070000.000 73.01

59414000.000 51.33435 581140000.000 73.02

59421000.000 51.33556 581210000.000 73.02

59428000.000 51.33677 581280000.000 73.02

59435000.000 51.33798 581350000.000 73.02

59442000.000 51.33918 581420000.000 73.02

59449000.000 51.34039 581490000.000 73.02

59456000.000 51.34160 581560000.000 73.03

59463000.000 51.34281 581630000.000 73.03

59470000.000 51.34402 581700000.000 73.03

59477000.000 51.34523 581770000.000 73.03

59484000.000 51.34644 581840000.000 73.03

59491000.000 51.34765 581910000.000 73.03

59498000.000 51.34885 581980000.000 73.04

59505000.000 51.35006 582050000.000 73.04

59512000.000 51.35127 582120000.000 73.04

59519000.000 51.35248 582190000.000 73.04

59526000.000 51.35369 582260000.000 73.04

59533000.000 51.35489 582330000.000 73.05

59540000.000 51.35610 582400000.000 73.05

59547000.000 51.35731 582470000.000 73.05

59554000.000 51.35852 582540000.000 73.05

59561000.000 51.35972 582610000.000 73.05

59568000.000 51.36093 582680000.000 73.05

59575000.000 51.36214 582750000.000 73.06

59582000.000 51.36335 582820000.000 73.06

59589000.000 51.36455 582890000.000 73.06

59596000.000 51.36576 582960000.000 73.06

59603000.000 51.36697 583030000.000 73.06

59610000.000 51.36817 583100000.000 73.06

59617000.000 51.36938 583170000.000 73.07

59624000.000 51.37058 583240000.000 73.07

59631000.000 51.37179 583310000.000 73.07

59638000.000 51.37300 583380000.000 73.07

59645000.000 51.37420 583450000.000 73.07

59652000.000 51.37541 583520000.000 73.08

59659000.000 51.37661 583590000.000 73.08

59666000.000 51.37782 583660000.000 73.08

59673000.000 51.37903 583730000.000 73.08

59680000.000 51.38023 583800000.000 73.08

59687000.000 51.38144 583870000.000 73.08

59694000.000 51.38264 583940000.000 73.09

59701000.000 51.38385 584010000.000 73.09

59708000.000 51.38505 584080000.000 73.09

59715000.000 51.38626 584150000.000 73.09

59722000.000 51.38746 584220000.000 73.09

59729000.000 51.38866 584290000.000 73.09

59736000.000 51.38987 584360000.000 73.10

59743000.000 51.39107 584430000.000 73.10

59750000.000 51.39228 584500000.000 73.10

59757000.000 51.39348 584570000.000 73.10

59764000.000 51.39469 584640000.000 73.10

59771000.000 51.39589 584710000.000 73.10

59778000.000 51.39709 584780000.000 73.11

59785000.000 51.39830 584850000.000 73.11

59792000.000 51.39950 584920000.000 73.11

59799000.000 51.40070 584990000.000 73.11

59806000.000 51.40191 585060000.000 73.11

59813000.000 51.40311 585130000.000 73.12

59820000.000 51.40431 585200000.000 73.12

59827000.000 51.40552 585270000.000 73.12

59834000.000 51.40672 585340000.000 73.12

59841000.000 51.40792 585410000.000 73.12

59848000.000 51.40913 585480000.000 73.12

59855000.000 51.41033 585550000.000 73.13

59862000.000 51.41153 585620000.000 73.13

59869000.000 51.41273 585690000.000 73.13

59876000.000 51.41393 585760000.000 73.13

59883000.000 51.41514 585830000.000 73.13

59890000.000 51.41634 585900000.000 73.13

59897000.000 51.41754 585970000.000 73.14

59904000.000 51.41874 586040000.000 73.14

59911000.000 51.41994 586110000.000 73.14

59918000.000 51.42115 586180000.000 73.14

59925000.000 51.42235 586250000.000 73.14

59932000.000 51.42355 586320000.000 73.15

59939000.000 51.42475 586390000.000 73.15

59946000.000 51.42595 586460000.000 73.15

59953000.000 51.42715 586530000.000 73.15

59960000.000 51.42835 586600000.000 73.15

59967000.000 51.42955 586670000.000 73.15

59974000.000 51.43075 586740000.000 73.16

59981000.000 51.43195 586810000.000 73.16

59988000.000 51.43315 586880000.000 73.16

59995000.000 51.43435 586950000.000 73.16

60002000.000 51.43555 587020000.000 73.16

60009000.000 51.43676 587090000.000 73.16

60016000.000 51.43795 587160000.000 73.17

60023000.000 51.43915 587230000.000 73.17

60030000.000 51.44035 587300000.000 73.17

60037000.000 51.44155 587370000.000 73.17

60044000.000 51.44275 587440000.000 73.17

60051000.000 51.44395 587510000.000 73.17

60058000.000 51.44515 587580000.000 73.18

60065000.000 51.44635 587650000.000 73.18

60072000.000 51.44755 587720000.000 73.18

60079000.000 51.44875 587790000.000 73.18

60086000.000 51.44995 587860000.000 73.18

60093000.000 51.45115 587930000.000 73.19

60100000.000 51.45235 588000000.000 73.19

60107000.000 51.45354 588070000.000 73.19

60114000.000 51.45474 588140000.000 73.19

60121000.000 51.45594 588210000.000 73.19

60128000.000 51.45714 588280000.000 73.19

60135000.000 51.45834 588350000.000 73.20

60142000.000 51.45954 588420000.000 73.20

60149000.000 51.46073 588490000.000 73.20

60156000.000 51.46193 588560000.000 73.20

60163000.000 51.46313 588630000.000 73.20

60170000.000 51.46433 588700000.000 73.20

60177000.000 51.46552 588770000.000 73.21

60184000.000 51.46672 588840000.000 73.21

60191000.000 51.46792 588910000.000 73.21

60198000.000 51.46911 588980000.000 73.21

60205000.000 51.47031 589050000.000 73.21

60212000.000 51.47151 589120000.000 73.21

60219000.000 51.47271 589190000.000 73.22

60226000.000 51.47390 589260000.000 73.22

60233000.000 51.47510 589330000.000 73.22

60240000.000 51.47629 589400000.000 73.22

60247000.000 51.47749 589470000.000 73.22

60254000.000 51.47869 589540000.000 73.23

60261000.000 51.47988 589610000.000 73.23

60268000.000 51.48108 589680000.000 73.23

60275000.000 51.48227 589750000.000 73.23

60282000.000 51.48347 589820000.000 73.23

60289000.000 51.48467 589890000.000 73.23

60296000.000 51.48586 589960000.000 73.24

60303000.000 51.48706 590030000.000 73.24

60310000.000 51.48825 590100000.000 73.24

60317000.000 51.48945 590170000.000 73.24

60324000.000 51.49064 590240000.000 73.24

60331000.000 51.49184 590310000.000 73.24

60338000.000 51.49303 590380000.000 73.25

60345000.000 51.49423 590450000.000 73.25

60352000.000 51.49542 590520000.000 73.25

60359000.000 51.49662 590590000.000 73.25

60366000.000 51.49781 590660000.000 73.25

60373000.000 51.49900 590730000.000 73.25

60380000.000 51.50020 590800000.000 73.26

60387000.000 51.50139 590870000.000 73.26

60394000.000 51.50259 590940000.000 73.26

60401000.000 51.50378 591010000.000 73.26

60408000.000 51.50497 591080000.000 73.26

60415000.000 51.50617 591150000.000 73.27

60422000.000 51.50736 591220000.000 73.27

60429000.000 51.50855 591290000.000 73.27

60436000.000 51.50975 591360000.000 73.27

60443000.000 51.51094 591430000.000 73.27

60450000.000 51.51213 591500000.000 73.27

60457000.000 51.51333 591570000.000 73.28

60464000.000 51.51452 591640000.000 73.28

60471000.000 51.51571 591710000.000 73.28

60478000.000 51.51691 591780000.000 73.28

60485000.000 51.51810 591850000.000 73.28

60492000.000 51.51929 591920000.000 73.28

60499000.000 51.52048 591990000.000 73.29

60506000.000 51.52167 592060000.000 73.29

60513000.000 51.52287 592130000.000 73.29

60520000.000 51.52406 592200000.000 73.29

60527000.000 51.52525 592270000.000 73.29

60534000.000 51.52644 592340000.000 73.29

60541000.000 51.52763 592410000.000 73.30

60548000.000 51.52883 592480000.000 73.30

60555000.000 51.53002 592550000.000 73.30

60562000.000 51.53121 592620000.000 73.30

60569000.000 51.53240 592690000.000 73.30

60576000.000 51.53359 592760000.000 73.31

60583000.000 51.53478 592830000.000 73.31

60590000.000 51.53597 592900000.000 73.31

60597000.000 51.53716 592970000.000 73.31

60604000.000 51.53835 593040000.000 73.31

60611000.000 51.53954 593110000.000 73.31

60618000.000 51.54073 593180000.000 73.32

60625000.000 51.54193 593250000.000 73.32

60632000.000 51.54312 593320000.000 73.32

60639000.000 51.54431 593390000.000 73.32

60646000.000 51.54550 593460000.000 73.32

60653000.000 51.54669 593530000.000 73.32

60660000.000 51.54787 593600000.000 73.33

60667000.000 51.54906 593670000.000 73.33

60674000.000 51.55025 593740000.000 73.33

60681000.000 51.55144 593810000.000 73.33

60688000.000 51.55263 593880000.000 73.33

60695000.000 51.55382 593950000.000 73.33

60702000.000 51.55501 594020000.000 73.34

60709000.000 51.55620 594090000.000 73.34

60716000.000 51.55739 594160000.000 73.34

60723000.000 51.55858 594230000.000 73.34

60730000.000 51.55977 594300000.000 73.34

60737000.000 51.56095 594370000.000 73.34

60744000.000 51.56214 594440000.000 73.35

60751000.000 51.56333 594510000.000 73.35

60758000.000 51.56452 594580000.000 73.35

60765000.000 51.56571 594650000.000 73.35

60772000.000 51.56690 594720000.000 73.35

60779000.000 51.56808 594790000.000 73.36

60786000.000 51.56927 594860000.000 73.36

60793000.000 51.57046 594930000.000 73.36

60800000.000 51.57165 595000000.000 73.36

60807000.000 51.57283 595070000.000 73.36

60814000.000 51.57402 595140000.000 73.36

60821000.000 51.57521 595210000.000 73.37

60828000.000 51.57640 595280000.000 73.37

60835000.000 51.57758 595350000.000 73.37

60842000.000 51.57877 595420000.000 73.37

60849000.000 51.57996 595490000.000 73.37

60856000.000 51.58114 595560000.000 73.37

60863000.000 51.58233 595630000.000 73.38

60870000.000 51.58352 595700000.000 73.38

60877000.000 51.58470 595770000.000 73.38

60884000.000 51.58589 595840000.000 73.38

60891000.000 51.58708 595910000.000 73.38

60898000.000 51.58826 595980000.000 73.38

60905000.000 51.58945 596050000.000 73.39

60912000.000 51.59063 596120000.000 73.39

60919000.000 51.59182 596190000.000 73.39

60926000.000 51.59300 596260000.000 73.39

60933000.000 51.59419 596330000.000 73.39

60940000.000 51.59538 596400000.000 73.39

60947000.000 51.59656 596470000.000 73.40

60954000.000 51.59775 596540000.000 73.40

60961000.000 51.59893 596610000.000 73.40

60968000.000 51.60012 596680000.000 73.40

60975000.000 51.60130 596750000.000 73.40

60982000.000 51.60249 596820000.000 73.41

60989000.000 51.60367 596890000.000 73.41

60996000.000 51.60485 596960000.000 73.41

61003000.000 51.60604 597030000.000 73.41

61010000.000 51.60722 597100000.000 73.41

61017000.000 51.60841 597170000.000 73.41

61024000.000 51.60959 597240000.000 73.42

61031000.000 51.61078 597310000.000 73.42

61038000.000 51.61196 597380000.000 73.42

61045000.000 51.61314 597450000.000 73.42

61052000.000 51.61433 597520000.000 73.42

61059000.000 51.61551 597590000.000 73.42

61066000.000 51.61669 597660000.000 73.43

61073000.000 51.61788 597730000.000 73.43

61080000.000 51.61906 597800000.000 73.43

61087000.000 51.62024 597870000.000 73.43

61094000.000 51.62143 597940000.000 73.43

61101000.000 51.62261 598010000.000 73.43

61108000.000 51.62379 598080000.000 73.44

61115000.000 51.62497 598150000.000 73.44

61122000.000 51.62616 598220000.000 73.44

61129000.000 51.62734 598290000.000 73.44

61136000.000 51.62852 598360000.000 73.44

61143000.000 51.62970 598430000.000 73.44

61150000.000 51.63089 598500000.000 73.45

61157000.000 51.63207 598570000.000 73.45

61164000.000 51.63325 598640000.000 73.45

61171000.000 51.63443 598710000.000 73.45

61178000.000 51.63561 598780000.000 73.45

61185000.000 51.63679 598850000.000 73.46

61192000.000 51.63798 598920000.000 73.46

61199000.000 51.63916 598990000.000 73.46

61206000.000 51.64034 599060000.000 73.46

61213000.000 51.64152 599130000.000 73.46

61220000.000 51.64270 599200000.000 73.46

61227000.000 51.64388 599270000.000 73.47

61234000.000 51.64506 599340000.000 73.47

61241000.000 51.64624 599410000.000 73.47

61248000.000 51.64742 599480000.000 73.47

61255000.000 51.64860 599550000.000 73.47

61262000.000 51.64979 599620000.000 73.47

61269000.000 51.65097 599690000.000 73.48

61276000.000 51.65215 599760000.000 73.48

61283000.000 51.65333 599830000.000 73.48

61290000.000 51.65451 599900000.000 73.48

61297000.000 51.65569 599970000.000 73.48

61304000.000 51.65687 600040000.000 73.48

61311000.000 51.65804 600110000.000 73.49

61318000.000 51.65922 600180000.000 73.49

61325000.000 51.66040 600250000.000 73.49

61332000.000 51.66158 600320000.000 73.49

61339000.000 51.66276 600390000.000 73.49

61346000.000 51.66394 600460000.000 73.49

61353000.000 51.66512 600530000.000 73.50

61360000.000 51.66630 600600000.000 73.50

61367000.000 51.66748 600670000.000 73.50

61374000.000 51.66866 600740000.000 73.50

61381000.000 51.66984 600810000.000 73.50

61388000.000 51.67101 600880000.000 73.50

61395000.000 51.67219 600950000.000 73.51

61402000.000 51.67337 601020000.000 73.51

61409000.000 51.67455 601090000.000 73.51

61416000.000 51.67573 601160000.000 73.51

61423000.000 51.67690 601230000.000 73.51

61430000.000 51.67808 601300000.000 73.52

61437000.000 51.67926 601370000.000 73.52

61444000.000 51.68044 601440000.000 73.52

61451000.000 51.68161 601510000.000 73.52

61458000.000 51.68279 601580000.000 73.52

61465000.000 51.68397 601650000.000 73.52

61472000.000 51.68515 601720000.000 73.53

61479000.000 51.68632 601790000.000 73.53

61486000.000 51.68750 601860000.000 73.53

61493000.000 51.68868 601930000.000 73.53

61500000.000 51.68985 602000000.000 73.53

61507000.000 51.69103 602070000.000 73.53

61514000.000 51.69221 602140000.000 73.54

61521000.000 51.69338 602210000.000 73.54

61528000.000 51.69456 602280000.000 73.54

61535000.000 51.69574 602350000.000 73.54

61542000.000 51.69691 602420000.000 73.54

61549000.000 51.69809 602490000.000 73.54

61556000.000 51.69926 602560000.000 73.55

61563000.000 51.70044 602630000.000 73.55

61570000.000 51.70162 602700000.000 73.55

61577000.000 51.70279 602770000.000 73.55

61584000.000 51.70397 602840000.000 73.55

61591000.000 51.70514 602910000.000 73.55

61598000.000 51.70632 602980000.000 73.56

61605000.000 51.70749 603050000.000 73.56

61612000.000 51.70867 603120000.000 73.56

61619000.000 51.70984 603190000.000 73.56

61626000.000 51.71102 603260000.000 73.56

61633000.000 51.71219 603330000.000 73.56

61640000.000 51.71337 603400000.000 73.57

61647000.000 51.71454 603470000.000 73.57

61654000.000 51.71572 603540000.000 73.57

61661000.000 51.71689 603610000.000 73.57

61668000.000 51.71806 603680000.000 73.57

61675000.000 51.71924 603750000.000 73.57

61682000.000 51.72041 603820000.000 73.58

61689000.000 51.72159 603890000.000 73.58

61696000.000 51.72276 603960000.000 73.58

61703000.000 51.72393 604030000.000 73.58

61710000.000 51.72511 604100000.000 73.58

61717000.000 51.72628 604170000.000 73.59

61724000.000 51.72745 604240000.000 73.59

61731000.000 51.72863 604310000.000 73.59

61738000.000 51.72980 604380000.000 73.59

61745000.000 51.73097 604450000.000 73.59

61752000.000 51.73215 604520000.000 73.59

61759000.000 51.73332 604590000.000 73.60

61766000.000 51.73449 604660000.000 73.60

61773000.000 51.73566 604730000.000 73.60

61780000.000 51.73684 604800000.000 73.60

61787000.000 51.73801 604870000.000 73.60

61794000.000 51.73918 604940000.000 73.60

61801000.000 51.74035 605010000.000 73.61

61808000.000 51.74152 605080000.000 73.61

61815000.000 51.74270 605150000.000 73.61

61822000.000 51.74387 605220000.000 73.61

61829000.000 51.74504 605290000.000 73.61

61836000.000 51.74621 605360000.000 73.61

61843000.000 51.74738 605430000.000 73.62

61850000.000 51.74855 605500000.000 73.62

61857000.000 51.74973 605570000.000 73.62

61864000.000 51.75090 605640000.000 73.62

61871000.000 51.75207 605710000.000 73.62

61878000.000 51.75324 605780000.000 73.62

61885000.000 51.75441 605850000.000 73.63

61892000.000 51.75558 605920000.000 73.63

61899000.000 51.75675 605990000.000 73.63

61906000.000 51.75792 606060000.000 73.63

61913000.000 51.75909 606130000.000 73.63

61920000.000 51.76026 606200000.000 73.63

61927000.000 51.76143 606270000.000 73.64

61934000.000 51.76260 606340000.000 73.64

61941000.000 51.76377 606410000.000 73.64

61948000.000 51.76494 606480000.000 73.64

61955000.000 51.76611 606550000.000 73.64

61962000.000 51.76728 606620000.000 73.64

61969000.000 51.76845 606690000.000 73.65

61976000.000 51.76962 606760000.000 73.65

61983000.000 51.77079 606830000.000 73.65

61990000.000 51.77196 606900000.000 73.65

61997000.000 51.77313 606970000.000 73.65

62004000.000 51.77430 607040000.000 73.65

62011000.000 51.77547 607110000.000 73.66

62018000.000 51.77664 607180000.000 73.66

62025000.000 51.77781 607250000.000 73.66

62032000.000 51.77897 607320000.000 73.66

62039000.000 51.78014 607390000.000 73.66

62046000.000 51.78131 607460000.000 73.67

62053000.000 51.78248 607530000.000 73.67

62060000.000 51.78365 607600000.000 73.67

62067000.000 51.78482 607670000.000 73.67

62074000.000 51.78598 607740000.000 73.67

62081000.000 51.78715 607810000.000 73.67

62088000.000 51.78832 607880000.000 73.68

62095000.000 51.78949 607950000.000 73.68

62102000.000 51.79065 608020000.000 73.68

62109000.000 51.79182 608090000.000 73.68

62116000.000 51.79299 608160000.000 73.68

62123000.000 51.79416 608230000.000 73.68

62130000.000 51.79532 608300000.000 73.69

62137000.000 51.79649 608370000.000 73.69

62144000.000 51.79766 608440000.000 73.69

62151000.000 51.79883 608510000.000 73.69

62158000.000 51.79999 608580000.000 73.69

62165000.000 51.80116 608650000.000 73.69

62172000.000 51.80233 608720000.000 73.70

62179000.000 51.80349 608790000.000 73.70

62186000.000 51.80466 608860000.000 73.70

62193000.000 51.80582 608930000.000 73.70

62200000.000 51.80699 609000000.000 73.70

62207000.000 51.80816 609070000.000 73.70

62214000.000 51.80932 609140000.000 73.71

62221000.000 51.81049 609210000.000 73.71

62228000.000 51.81165 609280000.000 73.71

62235000.000 51.81282 609350000.000 73.71

62242000.000 51.81398 609420000.000 73.71

62249000.000 51.81515 609490000.000 73.71

62256000.000 51.81632 609560000.000 73.72

62263000.000 51.81748 609630000.000 73.72

62270000.000 51.81865 609700000.000 73.72

62277000.000 51.81981 609770000.000 73.72

62284000.000 51.82098 609840000.000 73.72

62291000.000 51.82214 609910000.000 73.72

62298000.000 51.82330 609980000.000 73.73

62305000.000 51.82447 610050000.000 73.73

62312000.000 51.82563 610120000.000 73.73

62319000.000 51.82680 610190000.000 73.73

62326000.000 51.82796 610260000.000 73.73

62333000.000 51.82913 610330000.000 73.73

62340000.000 51.83029 610400000.000 73.74

62347000.000 51.83145 610470000.000 73.74

62354000.000 51.83262 610540000.000 73.74

62361000.000 51.83378 610610000.000 73.74

62368000.000 51.83495 610680000.000 73.74

62375000.000 51.83611 610750000.000 73.74

62382000.000 51.83727 610820000.000 73.75

62389000.000 51.83844 610890000.000 73.75

62396000.000 51.83960 610960000.000 73.75

62403000.000 51.84076 611030000.000 73.75

62410000.000 51.84193 611100000.000 73.75

62417000.000 51.84309 611170000.000 73.75

62424000.000 51.84425 611240000.000 73.76

62431000.000 51.84541 611310000.000 73.76

62438000.000 51.84658 611380000.000 73.76

62445000.000 51.84774 611450000.000 73.76

62452000.000 51.84890 611520000.000 73.76

62459000.000 51.85006 611590000.000 73.76

62466000.000 51.85123 611660000.000 73.77

62473000.000 51.85239 611730000.000 73.77

62480000.000 51.85355 611800000.000 73.77

62487000.000 51.85471 611870000.000 73.77

62494000.000 51.85587 611940000.000 73.77

62501000.000 51.85703 612010000.000 73.78

62508000.000 51.85820 612080000.000 73.78

62515000.000 51.85936 612150000.000 73.78

62522000.000 51.86052 612220000.000 73.78

62529000.000 51.86168 612290000.000 73.78

62536000.000 51.86284 612360000.000 73.78

62543000.000 51.86400 612430000.000 73.79

62550000.000 51.86516 612500000.000 73.79

62557000.000 51.86632 612570000.000 73.79

62564000.000 51.86748 612640000.000 73.79

62571000.000 51.86865 612710000.000 73.79

62578000.000 51.86981 612780000.000 73.79

62585000.000 51.87097 612850000.000 73.80

62592000.000 51.87213 612920000.000 73.80

62599000.000 51.87329 612990000.000 73.80

62606000.000 51.87445 613060000.000 73.80

62613000.000 51.87561 613130000.000 73.80

62620000.000 51.87677 613200000.000 73.80

62627000.000 51.87793 613270000.000 73.81

62634000.000 51.87909 613340000.000 73.81

62641000.000 51.88025 613410000.000 73.81

62648000.000 51.88140 613480000.000 73.81

62655000.000 51.88256 613550000.000 73.81

62662000.000 51.88372 613620000.000 73.81

62669000.000 51.88488 613690000.000 73.82

62676000.000 51.88604 613760000.000 73.82

62683000.000 51.88720 613830000.000 73.82

62690000.000 51.88836 613900000.000 73.82

62697000.000 51.88952 613970000.000 73.82

62704000.000 51.89068 614040000.000 73.82

62711000.000 51.89184 614110000.000 73.83

62718000.000 51.89299 614180000.000 73.83

62725000.000 51.89415 614250000.000 73.83

62732000.000 51.89531 614320000.000 73.83

62739000.000 51.89647 614390000.000 73.83

62746000.000 51.89763 614460000.000 73.83

62753000.000 51.89878 614530000.000 73.84

62760000.000 51.89994 614600000.000 73.84

62767000.000 51.90110 614670000.000 73.84

62774000.000 51.90226 614740000.000 73.84

62781000.000 51.90341 614810000.000 73.84

62788000.000 51.90457 614880000.000 73.84

62795000.000 51.90573 614950000.000 73.85

62802000.000 51.90689 615020000.000 73.85

62809000.000 51.90804 615090000.000 73.85

62816000.000 51.90920 615160000.000 73.85

62823000.000 51.91036 615230000.000 73.85

62830000.000 51.91151 615300000.000 73.85

62837000.000 51.91267 615370000.000 73.86

62844000.000 51.91383 615440000.000 73.86

62851000.000 51.91498 615510000.000 73.86

62858000.000 51.91614 615580000.000 73.86

62865000.000 51.91730 615650000.000 73.86

62872000.000 51.91845 615720000.000 73.86

62879000.000 51.91961 615790000.000 73.87

62886000.000 51.92076 615860000.000 73.87

62893000.000 51.92192 615930000.000 73.87

62900000.000 51.92308 616000000.000 73.87

62907000.000 51.92423 616070000.000 73.87

62914000.000 51.92539 616140000.000 73.87

62921000.000 51.92654 616210000.000 73.88

62928000.000 51.92770 616280000.000 73.88

62935000.000 51.92885 616350000.000 73.88

62942000.000 51.93001 616420000.000 73.88

62949000.000 51.93116 616490000.000 73.88

62956000.000 51.93232 616560000.000 73.88

62963000.000 51.93347 616630000.000 73.89

62970000.000 51.93463 616700000.000 73.89

62977000.000 51.93578 616770000.000 73.89

62984000.000 51.93694 616840000.000 73.89

62991000.000 51.93809 616910000.000 73.89

62998000.000 51.93925 616980000.000 73.89

63005000.000 51.94040 617050000.000 73.90

63012000.000 51.94155 617120000.000 73.90

63019000.000 51.94271 617190000.000 73.90

63026000.000 51.94386 617260000.000 73.90

63033000.000 51.94502 617330000.000 73.90

63040000.000 51.94617 617400000.000 73.90

63047000.000 51.94732 617470000.000 73.91

63054000.000 51.94848 617540000.000 73.91

63061000.000 51.94963 617610000.000 73.91

63068000.000 51.95078 617680000.000 73.91

63075000.000 51.95194 617750000.000 73.91

63082000.000 51.95309 617820000.000 73.91

63089000.000 51.95424 617890000.000 73.92

63096000.000 51.95539 617960000.000 73.92

63103000.000 51.95655 618030000.000 73.92

63110000.000 51.95770 618100000.000 73.92

63117000.000 51.95885 618170000.000 73.92

63124000.000 51.96001 618240000.000 73.92

63131000.000 51.96116 618310000.000 73.93

63138000.000 51.96231 618380000.000 73.93

63145000.000 51.96346 618450000.000 73.93

63152000.000 51.96461 618520000.000 73.93

63159000.000 51.96577 618590000.000 73.93

63166000.000 51.96692 618660000.000 73.93

63173000.000 51.96807 618730000.000 73.94

63180000.000 51.96922 618800000.000 73.94

63187000.000 51.97037 618870000.000 73.94

63194000.000 51.97152 618940000.000 73.94

63201000.000 51.97268 619010000.000 73.94

63208000.000 51.97383 619080000.000 73.94

63215000.000 51.97498 619150000.000 73.95

63222000.000 51.97613 619220000.000 73.95

63229000.000 51.97728 619290000.000 73.95

63236000.000 51.97843 619360000.000 73.95

63243000.000 51.97958 619430000.000 73.95

63250000.000 51.98073 619500000.000 73.95

63257000.000 51.98188 619570000.000 73.96

63264000.000 51.98303 619640000.000 73.96

63271000.000 51.98418 619710000.000 73.96

63278000.000 51.98533 619780000.000 73.96

63285000.000 51.98648 619850000.000 73.96

63292000.000 51.98763 619920000.000 73.96

63299000.000 51.98878 619990000.000 73.97

63306000.000 51.98993 620060000.000 73.97

63313000.000 51.99108 620130000.000 73.97

63320000.000 51.99223 620200000.000 73.97

63327000.000 51.99338 620270000.000 73.97

63334000.000 51.99453 620340000.000 73.97

63341000.000 51.99568 620410000.000 73.98

63348000.000 51.99683 620480000.000 73.98

63355000.000 51.99798 620550000.000 73.98

63362000.000 51.99913 620620000.000 73.98

63369000.000 52.00028 620690000.000 73.98

63376000.000 52.00143 620760000.000 73.98

63383000.000 52.00257 620830000.000 73.99

63390000.000 52.00372 620900000.000 73.99

63397000.000 52.00487 620970000.000 73.99

63404000.000 52.00602 621040000.000 73.99

63411000.000 52.00717 621110000.000 73.99

63418000.000 52.00832 621180000.000 73.99

63425000.000 52.00946 621250000.000 74.00

63432000.000 52.01061 621320000.000 74.00

63439000.000 52.01176 621390000.000 74.00

63446000.000 52.01291 621460000.000 74.00

63453000.000 52.01406 621530000.000 74.00

63460000.000 52.01520 621600000.000 74.00

63467000.000 52.01635 621670000.000 74.01

63474000.000 52.01750 621740000.000 74.01

63481000.000 52.01865 621810000.000 74.01

63488000.000 52.01979 621880000.000 74.01

63495000.000 52.02094 621950000.000 74.01

63502000.000 52.02209 622020000.000 74.01

63509000.000 52.02323 622090000.000 74.02

63516000.000 52.02438 622160000.000 74.02

63523000.000 52.02553 622230000.000 74.02

63530000.000 52.02667 622300000.000 74.02

63537000.000 52.02782 622370000.000 74.02

63544000.000 52.02897 622440000.000 74.02

63551000.000 52.03011 622510000.000 74.03

63558000.000 52.03126 622580000.000 74.03

63565000.000 52.03240 622650000.000 74.03

63572000.000 52.03355 622720000.000 74.03

63579000.000 52.03470 622790000.000 74.03

63586000.000 52.03584 622860000.000 74.03

63593000.000 52.03699 622930000.000 74.04

63600000.000 52.03813 623000000.000 74.04

63607000.000 52.03928 623070000.000 74.04

63614000.000 52.04042 623140000.000 74.04

63621000.000 52.04157 623210000.000 74.04

63628000.000 52.04271 623280000.000 74.04

63635000.000 52.04386 623350000.000 74.05

63642000.000 52.04500 623420000.000 74.05

63649000.000 52.04615 623490000.000 74.05

63656000.000 52.04729 623560000.000 74.05

63663000.000 52.04844 623630000.000 74.05

63670000.000 52.04958 623700000.000 74.05

63677000.000 52.05073 623770000.000 74.06

63684000.000 52.05187 623840000.000 74.06

63691000.000 52.05302 623910000.000 74.06

63698000.000 52.05416 623980000.000 74.06

63705000.000 52.05530 624050000.000 74.06

63712000.000 52.05645 624120000.000 74.06

63719000.000 52.05759 624190000.000 74.07

63726000.000 52.05874 624260000.000 74.07

63733000.000 52.05988 624330000.000 74.07

63740000.000 52.06102 624400000.000 74.07

63747000.000 52.06217 624470000.000 74.07

63754000.000 52.06331 624540000.000 74.07

63761000.000 52.06445 624610000.000 74.08

63768000.000 52.06560 624680000.000 74.08

63775000.000 52.06674 624750000.000 74.08

63782000.000 52.06788 624820000.000 74.08

63789000.000 52.06902 624890000.000 74.08

63796000.000 52.07017 624960000.000 74.08

63803000.000 52.07131 625030000.000 74.09

63810000.000 52.07245 625100000.000 74.09

63817000.000 52.07360 625170000.000 74.09

63824000.000 52.07474 625240000.000 74.09

63831000.000 52.07588 625310000.000 74.09

63838000.000 52.07702 625380000.000 74.09

63845000.000 52.07816 625450000.000 74.10

63852000.000 52.07931 625520000.000 74.10

63859000.000 52.08045 625590000.000 74.10

63866000.000 52.08159 625660000.000 74.10

63873000.000 52.08273 625730000.000 74.10

63880000.000 52.08387 625800000.000 74.10

63887000.000 52.08501 625870000.000 74.11

63894000.000 52.08616 625940000.000 74.11

63901000.000 52.08730 626010000.000 74.11

63908000.000 52.08844 626080000.000 74.11

63915000.000 52.08958 626150000.000 74.11

63922000.000 52.09072 626220000.000 74.11

63929000.000 52.09186 626290000.000 74.12

63936000.000 52.09300 626360000.000 74.12

63943000.000 52.09414 626430000.000 74.12

63950000.000 52.09528 626500000.000 74.12

63957000.000 52.09642 626570000.000 74.12

63964000.000 52.09756 626640000.000 74.12

63971000.000 52.09870 626710000.000 74.13

63978000.000 52.09984 626780000.000 74.13

63985000.000 52.10098 626850000.000 74.13

63992000.000 52.10212 626920000.000 74.13

63999000.000 52.10326 626990000.000 74.13

64006000.000 52.10440 627060000.000 74.13

64013000.000 52.10554 627130000.000 74.14

64020000.000 52.10668 627200000.000 74.14

64027000.000 52.10782 627270000.000 74.14

64034000.000 52.10896 627340000.000 74.14

64041000.000 52.11010 627410000.000 74.14

64048000.000 52.11124 627480000.000 74.14

64055000.000 52.11238 627550000.000 74.15

64062000.000 52.11352 627620000.000 74.15

64069000.000 52.11466 627690000.000 74.15

64076000.000 52.11579 627760000.000 74.15

64083000.000 52.11693 627830000.000 74.15

64090000.000 52.11807 627900000.000 74.15

64097000.000 52.11921 627970000.000 74.16

64104000.000 52.12035 628040000.000 74.16

64111000.000 52.12149 628110000.000 74.16

64118000.000 52.12262 628180000.000 74.16

64125000.000 52.12376 628250000.000 74.16

64132000.000 52.12490 628320000.000 74.16

64139000.000 52.12604 628390000.000 74.17

64146000.000 52.12718 628460000.000 74.17

64153000.000 52.12831 628530000.000 74.17

64160000.000 52.12945 628600000.000 74.17

64167000.000 52.13059 628670000.000 74.17

64174000.000 52.13173 628740000.000 74.17

64181000.000 52.13286 628810000.000 74.18

64188000.000 52.13400 628880000.000 74.18

64195000.000 52.13514 628950000.000 74.18

64202000.000 52.13627 629020000.000 74.18

64209000.000 52.13741 629090000.000 74.18

64216000.000 52.13855 629160000.000 74.18

64223000.000 52.13968 629230000.000 74.19

64230000.000 52.14082 629300000.000 74.19

64237000.000 52.14196 629370000.000 74.19

64244000.000 52.14309 629440000.000 74.19

64251000.000 52.14423 629510000.000 74.19

64258000.000 52.14537 629580000.000 74.19

64265000.000 52.14650 629650000.000 74.20

64272000.000 52.14764 629720000.000 74.20

64279000.000 52.14877 629790000.000 74.20

64286000.000 52.14991 629860000.000 74.20

64293000.000 52.15105 629930000.000 74.20

64300000.000 52.15218 630000000.000 74.20

64307000.000 52.15332 630070000.000 74.21

64314000.000 52.15445 630140000.000 74.21

64321000.000 52.15559 630210000.000 74.21

64328000.000 52.15672 630280000.000 74.21

64335000.000 52.15786 630350000.000 74.21

64342000.000 52.15899 630420000.000 74.21

64349000.000 52.16013 630490000.000 74.22

64356000.000 52.16126 630560000.000 74.22

64363000.000 52.16240 630630000.000 74.22

64370000.000 52.16353 630700000.000 74.22

64377000.000 52.16467 630770000.000 74.22

64384000.000 52.16580 630840000.000 74.22

64391000.000 52.16693 630910000.000 74.23

64398000.000 52.16807 630980000.000 74.23

64405000.000 52.16920 631050000.000 74.23

64412000.000 52.17034 631120000.000 74.23

64419000.000 52.17147 631190000.000 74.23

64426000.000 52.17260 631260000.000 74.23

64433000.000 52.17374 631330000.000 74.24

64440000.000 52.17487 631400000.000 74.24

64447000.000 52.17601 631470000.000 74.24

64454000.000 52.17714 631540000.000 74.24

64461000.000 52.17827 631610000.000 74.24

64468000.000 52.17941 631680000.000 74.24

64475000.000 52.18054 631750000.000 74.24

64482000.000 52.18167 631820000.000 74.25

64489000.000 52.18280 631890000.000 74.25

64496000.000 52.18394 631960000.000 74.25

64503000.000 52.18507 632030000.000 74.25

64510000.000 52.18620 632100000.000 74.25

64517000.000 52.18733 632170000.000 74.25

64524000.000 52.18847 632240000.000 74.26

64531000.000 52.18960 632310000.000 74.26

64538000.000 52.19073 632380000.000 74.26

64545000.000 52.19186 632450000.000 74.26

64552000.000 52.19300 632520000.000 74.26

64559000.000 52.19413 632590000.000 74.26

64566000.000 52.19526 632660000.000 74.27

64573000.000 52.19639 632730000.000 74.27

64580000.000 52.19752 632800000.000 74.27

64587000.000 52.19865 632870000.000 74.27

64594000.000 52.19979 632940000.000 74.27

64601000.000 52.20092 633010000.000 74.27

64608000.000 52.20205 633080000.000 74.28

64615000.000 52.20318 633150000.000 74.28

64622000.000 52.20431 633220000.000 74.28

64629000.000 52.20544 633290000.000 74.28

64636000.000 52.20657 633360000.000 74.28

64643000.000 52.20770 633430000.000 74.28

64650000.000 52.20883 633500000.000 74.29

64657000.000 52.20996 633570000.000 74.29

64664000.000 52.21109 633640000.000 74.29

64671000.000 52.21222 633710000.000 74.29

64678000.000 52.21336 633780000.000 74.29

64685000.000 52.21449 633850000.000 74.29

64692000.000 52.21562 633920000.000 74.30

64699000.000 52.21675 633990000.000 74.30

64706000.000 52.21788 634060000.000 74.30

64713000.000 52.21900 634130000.000 74.30

64720000.000 52.22013 634200000.000 74.30

64727000.000 52.22126 634270000.000 74.30

64734000.000 52.22239 634340000.000 74.31

64741000.000 52.22352 634410000.000 74.31

64748000.000 52.22465 634480000.000 74.31

64755000.000 52.22578 634550000.000 74.31

64762000.000 52.22691 634620000.000 74.31

64769000.000 52.22804 634690000.000 74.31

64776000.000 52.22917 634760000.000 74.32

64783000.000 52.23030 634830000.000 74.32

64790000.000 52.23143 634900000.000 74.32

64797000.000 52.23255 634970000.000 74.32

64804000.000 52.23368 635040000.000 74.32

64811000.000 52.23481 635110000.000 74.32

64818000.000 52.23594 635180000.000 74.33

64825000.000 52.23707 635250000.000 74.33

64832000.000 52.23820 635320000.000 74.33

64839000.000 52.23932 635390000.000 74.33

64846000.000 52.24045 635460000.000 74.33

64853000.000 52.24158 635530000.000 74.33

64860000.000 52.24271 635600000.000 74.34

64867000.000 52.24383 635670000.000 74.34

64874000.000 52.24496 635740000.000 74.34

64881000.000 52.24609 635810000.000 74.34

64888000.000 52.24722 635880000.000 74.34

64895000.000 52.24834 635950000.000 74.34

64902000.000 52.24947 636020000.000 74.35

64909000.000 52.25060 636090000.000 74.35

64916000.000 52.25173 636160000.000 74.35

64923000.000 52.25285 636230000.000 74.35

64930000.000 52.25398 636300000.000 74.35

64937000.000 52.25511 636370000.000 74.35

64944000.000 52.25623 636440000.000 74.35

64951000.000 52.25736 636510000.000 74.36

64958000.000 52.25848 636580000.000 74.36

64965000.000 52.25961 636650000.000 74.36

64972000.000 52.26074 636720000.000 74.36

64979000.000 52.26186 636790000.000 74.36

64986000.000 52.26299 636860000.000 74.36

64993000.000 52.26412 636930000.000 74.37

65000000.000 52.26524 637000000.000 74.37

65007000.000 52.26637 637070000.000 74.37

65014000.000 52.26749 637140000.000 74.37

65021000.000 52.26862 637210000.000 74.37

65028000.000 52.26974 637280000.000 74.37

65035000.000 52.27087 637350000.000 74.38

65042000.000 52.27199 637420000.000 74.38

65049000.000 52.27312 637490000.000 74.38

65056000.000 52.27424 637560000.000 74.38

65063000.000 52.27537 637630000.000 74.38

65070000.000 52.27649 637700000.000 74.38

65077000.000 52.27762 637770000.000 74.39

65084000.000 52.27874 637840000.000 74.39

65091000.000 52.27987 637910000.000 74.39

65098000.000 52.28099 637980000.000 74.39

65105000.000 52.28212 638050000.000 74.39

65112000.000 52.28324 638120000.000 74.39

65119000.000 52.28436 638190000.000 74.40

65126000.000 52.28549 638260000.000 74.40

65133000.000 52.28661 638330000.000 74.40

65140000.000 52.28774 638400000.000 74.40

65147000.000 52.28886 638470000.000 74.40

65154000.000 52.28998 638540000.000 74.40

65161000.000 52.29111 638610000.000 74.41

65168000.000 52.29223 638680000.000 74.41

65175000.000 52.29335 638750000.000 74.41

65182000.000 52.29448 638820000.000 74.41

65189000.000 52.29560 638890000.000 74.41

65196000.000 52.29672 638960000.000 74.41

65203000.000 52.29785 639030000.000 74.42

65210000.000 52.29897 639100000.000 74.42

65217000.000 52.30009 639170000.000 74.42

65224000.000 52.30121 639240000.000 74.42

65231000.000 52.30234 639310000.000 74.42

65238000.000 52.30346 639380000.000 74.42

65245000.000 52.30458 639450000.000 74.43

65252000.000 52.30570 639520000.000 74.43

65259000.000 52.30683 639590000.000 74.43

65266000.000 52.30795 639660000.000 74.43

65273000.000 52.30907 639730000.000 74.43

65280000.000 52.31019 639800000.000 74.43

65287000.000 52.31131 639870000.000 74.43

65294000.000 52.31244 639940000.000 74.44

65301000.000 52.31356 640010000.000 74.44

65308000.000 52.31468 640080000.000 74.44

65315000.000 52.31580 640150000.000 74.44

65322000.000 52.31692 640220000.000 74.44

65329000.000 52.31804 640290000.000 74.44

65336000.000 52.31916 640360000.000 74.45

65343000.000 52.32028 640430000.000 74.45

65350000.000 52.32141 640500000.000 74.45

65357000.000 52.32253 640570000.000 74.45

65364000.000 52.32365 640640000.000 74.45

65371000.000 52.32477 640710000.000 74.45

65378000.000 52.32589 640780000.000 74.46

65385000.000 52.32701 640850000.000 74.46

65392000.000 52.32813 640920000.000 74.46

65399000.000 52.32925 640990000.000 74.46

65406000.000 52.33037 641060000.000 74.46

65413000.000 52.33149 641130000.000 74.46

65420000.000 52.33261 641200000.000 74.47

65427000.000 52.33373 641270000.000 74.47

65434000.000 52.33485 641340000.000 74.47

65441000.000 52.33597 641410000.000 74.47

65448000.000 52.33709 641480000.000 74.47

65455000.000 52.33821 641550000.000 74.47

65462000.000 52.33933 641620000.000 74.48

65469000.000 52.34045 641690000.000 74.48

65476000.000 52.34157 641760000.000 74.48

65483000.000 52.34269 641830000.000 74.48

65490000.000 52.34380 641900000.000 74.48

65497000.000 52.34492 641970000.000 74.48

65504000.000 52.34604 642040000.000 74.49

65511000.000 52.34716 642110000.000 74.49

65518000.000 52.34828 642180000.000 74.49

65525000.000 52.34940 642250000.000 74.49

65532000.000 52.35052 642320000.000 74.49

65539000.000 52.35163 642390000.000 74.49

65546000.000 52.35275 642460000.000 74.50

65553000.000 52.35387 642530000.000 74.50

65560000.000 52.35499 642600000.000 74.50

65567000.000 52.35611 642670000.000 74.50

65574000.000 52.35722 642740000.000 74.50

65581000.000 52.35834 642810000.000 74.50

65588000.000 52.35946 642880000.000 74.50

65595000.000 52.36058 642950000.000 74.51

65602000.000 52.36170 643020000.000 74.51

65609000.000 52.36281 643090000.000 74.51

65616000.000 52.36393 643160000.000 74.51

65623000.000 52.36505 643230000.000 74.51

65630000.000 52.36616 643300000.000 74.51

65637000.000 52.36728 643370000.000 74.52

65644000.000 52.36840 643440000.000 74.52

65651000.000 52.36952 643510000.000 74.52

65658000.000 52.37063 643580000.000 74.52

65665000.000 52.37175 643650000.000 74.52

65672000.000 52.37287 643720000.000 74.52

65679000.000 52.37398 643790000.000 74.53

65686000.000 52.37510 643860000.000 74.53

65693000.000 52.37621 643930000.000 74.53

65700000.000 52.37733 644000000.000 74.53

65707000.000 52.37845 644070000.000 74.53

65714000.000 52.37956 644140000.000 74.53

65721000.000 52.38068 644210000.000 74.54

65728000.000 52.38179 644280000.000 74.54

65735000.000 52.38291 644350000.000 74.54

65742000.000 52.38403 644420000.000 74.54

65749000.000 52.38514 644490000.000 74.54

65756000.000 52.38626 644560000.000 74.54

65763000.000 52.38737 644630000.000 74.55

65770000.000 52.38849 644700000.000 74.55

65777000.000 52.38960 644770000.000 74.55

65784000.000 52.39072 644840000.000 74.55

65791000.000 52.39183 644910000.000 74.55

65798000.000 52.39295 644980000.000 74.55

65805000.000 52.39406 645050000.000 74.55

65812000.000 52.39518 645120000.000 74.56

65819000.000 52.39629 645190000.000 74.56

65826000.000 52.39740 645260000.000 74.56

65833000.000 52.39852 645330000.000 74.56

65840000.000 52.39963 645400000.000 74.56

65847000.000 52.40075 645470000.000 74.56

65854000.000 52.40186 645540000.000 74.57

65861000.000 52.40298 645610000.000 74.57

65868000.000 52.40409 645680000.000 74.57

65875000.000 52.40520 645750000.000 74.57

65882000.000 52.40632 645820000.000 74.57

65889000.000 52.40743 645890000.000 74.57

65896000.000 52.40854 645960000.000 74.58

65903000.000 52.40966 646030000.000 74.58

65910000.000 52.41077 646100000.000 74.58

65917000.000 52.41188 646170000.000 74.58

65924000.000 52.41300 646240000.000 74.58

65931000.000 52.41411 646310000.000 74.58

65938000.000 52.41522 646380000.000 74.59

65945000.000 52.41634 646450000.000 74.59

65952000.000 52.41745 646520000.000 74.59

65959000.000 52.41856 646590000.000 74.59

65966000.000 52.41967 646660000.000 74.59

65973000.000 52.42079 646730000.000 74.59

65980000.000 52.42190 646800000.000 74.60

65987000.000 52.42301 646870000.000 74.60

65994000.000 52.42412 646940000.000 74.60

66001000.000 52.42524 647010000.000 74.60

66008000.000 52.42635 647080000.000 74.60

66015000.000 52.42746 647150000.000 74.60

66022000.000 52.42857 647220000.000 74.61

66029000.000 52.42968 647290000.000 74.61

66036000.000 52.43079 647360000.000 74.61

66043000.000 52.43191 647430000.000 74.61

66050000.000 52.43302 647500000.000 74.61

66057000.000 52.43413 647570000.000 74.61

66064000.000 52.43524 647640000.000 74.61

66071000.000 52.43635 647710000.000 74.62

66078000.000 52.43746 647780000.000 74.62

66085000.000 52.43857 647850000.000 74.62

66092000.000 52.43968 647920000.000 74.62

66099000.000 52.44079 647990000.000 74.62

66106000.000 52.44191 648060000.000 74.62

66113000.000 52.44302 648130000.000 74.63

66120000.000 52.44413 648200000.000 74.63

66127000.000 52.44524 648270000.000 74.63

66134000.000 52.44635 648340000.000 74.63

66141000.000 52.44746 648410000.000 74.63

66148000.000 52.44857 648480000.000 74.63

66155000.000 52.44968 648550000.000 74.64

66162000.000 52.45079 648620000.000 74.64

66169000.000 52.45190 648690000.000 74.64

66176000.000 52.45301 648760000.000 74.64

66183000.000 52.45412 648830000.000 74.64

66190000.000 52.45523 648900000.000 74.64

66197000.000 52.45634 648970000.000 74.65

66204000.000 52.45744 649040000.000 74.65

66211000.000 52.45855 649110000.000 74.65

66218000.000 52.45966 649180000.000 74.65

66225000.000 52.46077 649250000.000 74.65

66232000.000 52.46188 649320000.000 74.65

66239000.000 52.46299 649390000.000 74.66

66246000.000 52.46410 649460000.000 74.66

66253000.000 52.46521 649530000.000 74.66

66260000.000 52.46632 649600000.000 74.66

66267000.000 52.46742 649670000.000 74.66

66274000.000 52.46853 649740000.000 74.66

66281000.000 52.46964 649810000.000 74.66

66288000.000 52.47075 649880000.000 74.67

66295000.000 52.47186 649950000.000 74.67

66302000.000 52.47297 650020000.000 74.67

66309000.000 52.47407 650090000.000 74.67

66316000.000 52.47518 650160000.000 74.67

66323000.000 52.47629 650230000.000 74.67

66330000.000 52.47740 650300000.000 74.68

66337000.000 52.47850 650370000.000 74.68

66344000.000 52.47961 650440000.000 74.68

66351000.000 52.48072 650510000.000 74.68

66358000.000 52.48183 650580000.000 74.68

66365000.000 52.48293 650650000.000 74.68

66372000.000 52.48404 650720000.000 74.69

66379000.000 52.48515 650790000.000 74.69

66386000.000 52.48625 650860000.000 74.69

66393000.000 52.48736 650930000.000 74.69

66400000.000 52.48847 651000000.000 74.69

66407000.000 52.48958 651070000.000 74.69

66414000.000 52.49068 651140000.000 74.70

66421000.000 52.49179 651210000.000 74.70

66428000.000 52.49289 651280000.000 74.70

66435000.000 52.49400 651350000.000 74.70

66442000.000 52.49511 651420000.000 74.70

66449000.000 52.49621 651490000.000 74.70

66456000.000 52.49732 651560000.000 74.70

66463000.000 52.49842 651630000.000 74.71

66470000.000 52.49953 651700000.000 74.71

66477000.000 52.50064 651770000.000 74.71

66484000.000 52.50174 651840000.000 74.71

66491000.000 52.50285 651910000.000 74.71

66498000.000 52.50395 651980000.000 74.71

66505000.000 52.50506 652050000.000 74.72

66512000.000 52.50616 652120000.000 74.72

66519000.000 52.50727 652190000.000 74.72

66526000.000 52.50837 652260000.000 74.72

66533000.000 52.50948 652330000.000 74.72

66540000.000 52.51058 652400000.000 74.72

66547000.000 52.51169 652470000.000 74.73

66554000.000 52.51279 652540000.000 74.73

66561000.000 52.51390 652610000.000 74.73

66568000.000 52.51500 652680000.000 74.73

66575000.000 52.51611 652750000.000 74.73

66582000.000 52.51721 652820000.000 74.73

66589000.000 52.51831 652890000.000 74.74

66596000.000 52.51942 652960000.000 74.74

66603000.000 52.52052 653030000.000 74.74

66610000.000 52.52163 653100000.000 74.74

66617000.000 52.52273 653170000.000 74.74

66624000.000 52.52383 653240000.000 74.74

66631000.000 52.52494 653310000.000 74.74

66638000.000 52.52604 653380000.000 74.75

66645000.000 52.52715 653450000.000 74.75

66652000.000 52.52825 653520000.000 74.75

66659000.000 52.52935 653590000.000 74.75

66666000.000 52.53046 653660000.000 74.75

66673000.000 52.53156 653730000.000 74.75

66680000.000 52.53266 653800000.000 74.76

66687000.000 52.53376 653870000.000 74.76

66694000.000 52.53487 653940000.000 74.76

66701000.000 52.53597 654010000.000 74.76

66708000.000 52.53707 654080000.000 74.76

66715000.000 52.53818 654150000.000 74.76

66722000.000 52.53928 654220000.000 74.77

66729000.000 52.54038 654290000.000 74.77

66736000.000 52.54148 654360000.000 74.77

66743000.000 52.54258 654430000.000 74.77

66750000.000 52.54369 654500000.000 74.77

66757000.000 52.54479 654570000.000 74.77

66764000.000 52.54589 654640000.000 74.78

66771000.000 52.54699 654710000.000 74.78

66778000.000 52.54809 654780000.000 74.78

66785000.000 52.54920 654850000.000 74.78

66792000.000 52.55030 654920000.000 74.78

66799000.000 52.55140 654990000.000 74.78

66806000.000 52.55250 655060000.000 74.78

66813000.000 52.55360 655130000.000 74.79

66820000.000 52.55470 655200000.000 74.79

66827000.000 52.55580 655270000.000 74.79

66834000.000 52.55690 655340000.000 74.79

66841000.000 52.55801 655410000.000 74.79

66848000.000 52.55911 655480000.000 74.79

66855000.000 52.56021 655550000.000 74.80

66862000.000 52.56131 655620000.000 74.80

66869000.000 52.56241 655690000.000 74.80

66876000.000 52.56351 655760000.000 74.80

66883000.000 52.56461 655830000.000 74.80

66890000.000 52.56571 655900000.000 74.80

66897000.000 52.56681 655970000.000 74.81

66904000.000 52.56791 656040000.000 74.81

66911000.000 52.56901 656110000.000 74.81

66918000.000 52.57011 656180000.000 74.81

66925000.000 52.57121 656250000.000 74.81

66932000.000 52.57231 656320000.000 74.81

66939000.000 52.57341 656390000.000 74.82

66946000.000 52.57451 656460000.000 74.82

66953000.000 52.57561 656530000.000 74.82

66960000.000 52.57671 656600000.000 74.82

66967000.000 52.57781 656670000.000 74.82

66974000.000 52.57890 656740000.000 74.82

66981000.000 52.58000 656810000.000 74.82

66988000.000 52.58110 656880000.000 74.83

66995000.000 52.58220 656950000.000 74.83

67002000.000 52.58330 657020000.000 74.83

67009000.000 52.58440 657090000.000 74.83

67016000.000 52.58550 657160000.000 74.83

67023000.000 52.58660 657230000.000 74.83

67030000.000 52.58769 657300000.000 74.84

67037000.000 52.58879 657370000.000 74.84

67044000.000 52.58989 657440000.000 74.84

67051000.000 52.59099 657510000.000 74.84

67058000.000 52.59209 657580000.000 74.84

67065000.000 52.59318 657650000.000 74.84

67072000.000 52.59428 657720000.000 74.85

67079000.000 52.59538 657790000.000 74.85

67086000.000 52.59648 657860000.000 74.85

67093000.000 52.59758 657930000.000 74.85

67100000.000 52.59867 658000000.000 74.85

67107000.000 52.59977 658070000.000 74.85

67114000.000 52.60087 658140000.000 74.86

67121000.000 52.60197 658210000.000 74.86

67128000.000 52.60306 658280000.000 74.86

67135000.000 52.60416 658350000.000 74.86

67142000.000 52.60526 658420000.000 74.86

67149000.000 52.60635 658490000.000 74.86

67156000.000 52.60745 658560000.000 74.86

67163000.000 52.60855 658630000.000 74.87

67170000.000 52.60964 658700000.000 74.87

67177000.000 52.61074 658770000.000 74.87

67184000.000 52.61184 658840000.000 74.87

67191000.000 52.61293 658910000.000 74.87

67198000.000 52.61403 658980000.000 74.87

67205000.000 52.61512 659050000.000 74.88

67212000.000 52.61622 659120000.000 74.88

67219000.000 52.61732 659190000.000 74.88

67226000.000 52.61841 659260000.000 74.88

67233000.000 52.61951 659330000.000 74.88

67240000.000 52.62060 659400000.000 74.88

67247000.000 52.62170 659470000.000 74.89

67254000.000 52.62279 659540000.000 74.89

67261000.000 52.62389 659610000.000 74.89

67268000.000 52.62499 659680000.000 74.89

67275000.000 52.62608 659750000.000 74.89

67282000.000 52.62718 659820000.000 74.89

67289000.000 52.62827 659890000.000 74.89

67296000.000 52.62937 659960000.000 74.90

67303000.000 52.63046 660030000.000 74.90

67310000.000 52.63156 660100000.000 74.90

67317000.000 52.63265 660170000.000 74.90

67324000.000 52.63374 660240000.000 74.90

67331000.000 52.63484 660310000.000 74.90

67338000.000 52.63593 660380000.000 74.91

67345000.000 52.63703 660450000.000 74.91

67352000.000 52.63812 660520000.000 74.91

67359000.000 52.63922 660590000.000 74.91

67366000.000 52.64031 660660000.000 74.91

67373000.000 52.64140 660730000.000 74.91

67380000.000 52.64250 660800000.000 74.92

67387000.000 52.64359 660870000.000 74.92

67394000.000 52.64469 660940000.000 74.92

67401000.000 52.64578 661010000.000 74.92

67408000.000 52.64687 661080000.000 74.92

67415000.000 52.64797 661150000.000 74.92

67422000.000 52.64906 661220000.000 74.93

67429000.000 52.65015 661290000.000 74.93

67436000.000 52.65125 661360000.000 74.93
[truncated: 27,992 more chars]
